# High-Throughput Metabolomics and Diabetic Kidney Disease Progression: Evidence from the Chronic Renal Insufficiency (CRIC) Study

**DOI:** 10.1159/000521940

**Published:** 2022-02-23

**Authors:** Jing Zhang, Tobias Fuhrer, Hongping Ye, Brian Kwan, Daniel Montemayor, Jana Tumova, Manjula Darshi, Farsad Afshinnia, Julia J. Scialla, Amanda Anderson, Anna C. Porter, Jonathan J. Taliercio, Hernan Rincon-Choles, Panduranga Rao, Dawei Xie, Harold Feldman, Uwe Sauer, Kumar Sharma, Loki Natarajan

**Affiliations:** a Moores Cancer Center, University of California, San Diego, CA, USA;; b Institute of Molecular Systems Biology, ETH Zurich, Zurich, Switzerland;; c Department of Medicine, Center for Renal Precision Medicine, University of Texas Health Science Center at San Antonio, San Antonio, TX, USA;; d Division of Biostatistics and Bioinformatics, Herbert Wertheim School of Public Health and Human Longevity Science, University of California, San Diego, CA, USA;; e Division of Nephrology, Department of Internal Medicine, University of Michigan, Medical School, Ann Arbor, MI, USA;; f Departments of Medicine and Public Health Sciences, University of Virginia School of Medicine, Charlottesville, VA, USA;; g Department of Epidemiology, Tulane University School of Public Health and Tropical Medicine, New Orleans, LA, USA;; h Department of Biostatistics, Epidemiology and Informatics, Perelman School of Medicine, University of Pennsylvania, Philadelphia, PA, USA;; i Jesse Brown VA Medical Center, University of Illinois at Chicago, Chicago, IL, USA;; j Cleveland Clinic Foundation, Glickman Urological & Kidney Institute, Department of Nephrology, Cleveland, OH, USA;; k Center for Clinical Epidemiology and Biostatistics, Perelman School of Medicine at the University of Pennsylvania, Philadelphia, PA, USA

**Keywords:** Diabetes, Kidney disease, Metabolomics, Prognostic modeling, Lasso, Random forest, Pathways

## Abstract

**Introduction:**

Metabolomics could offer novel prognostic biomarkers and elucidate mechanisms of diabetic kidney disease (DKD) progression. Via metabolomic analysis of urine samples from 995 CRIC participants with diabetes and stateof-the-art statistical modeling, we aimed to identify metabolites prognostic to DKD progression.

**Methods:**

Urine samples (*N* = 995) were assayed for relative metabolite abundance by untargeted flow-injection mass spectrometry, and stringent statistical criteria were used to eliminate noisy compounds, resulting in 698 annotated metabolite ions. Utilizing the 698 metabolites’ ion abundance along with clinical data (demographics, blood pressure, HbA1c, eGFR, and albuminuria), we developed univariate and multivariate models for the eGFR slope using penalized (lasso) and random forest models. Final models were tested on time-to-ESKD (end-stage kidney disease) via cross-validated C-statistics. We also conducted pathway enrichment analysis and a targeted analysis of a subset of metabolites.

**Results:**

Six eGFR slope models selected 9–30 variables. In the adjusted ESKD model with highest C-statistic, valine (or betaine) and 3-(4-methyl-3-pentenyl) thiophene were associated (*p* < 0.05) with 44% and 65% higher hazard of ESKD per doubling of metabolite abundance, respectively. Also, 13 (of 15) prognostic amino acids, including valine and betaine, were confirmed in the targeted analysis. Enrichment analysis revealed pathways implicated in kidney and cardiometabolic disease.

**Conclusions:**

Using the diverse CRIC sample, a high-throughput untargeted assay, followed by targeted analysis, and rigorous statistical analysis to reduce false discovery, we identified several novel metabolites implicated in DKD progression. If replicated in independent cohorts, our findings could inform risk stratification and treatment strategies for patients with DKD.

## Introduction

In the USA, 25% of diabetic patients have chronic kidney disease, a major precursor to kidney failure [1]. Nearly half of the patients with kidney failure have diabetes [2]. Thus, it is important to identify DKD progression risk factors, so that kidney disease can be detected and treated early. While clinical factors, such as microalbuminuria, are generally prognostic for DKD, there is still large variability in DKD risk for patients with similar risk profiles [3–5], and furthermore, clinical risk factors do not offer molecular insights into disease pathology. Thus, there is a need to explore new biomarkers which may add prognostic power and more importantly identify biological mechanisms of DKD [6].

Metabolites, low-weight intermediates and end products of cellular pathways, could uncover physiological or pathological changes in chronic diseases [7]. Recent research emphasizes the importance of and need for further research on the human urine metabolome in kidney disease [8, 9].

Previous urine metabolome in DKD studies has largely been cross-sectional and aimed at identifying metabolites distinguishing DKD from healthy or suitable comparison controls [10–12]. Studies examining metabolomics for DKD progression contained several limitations: evaluating specific compounds, serum/plasma only assays, restriction to type I diabetes or specific subpopulations, small sample size, or short follow-up [13–17]. Highlighting recent work, the branched-chain amino acid pathway in type 1 diabetes [18] and lipogenesis in type 2 diabetes among American Indians [13] have been linked to DKD progression. Herein, we aimed to expand on prior works by using an extensive panel of urine metabolites in a large, diverse longitudinal cohort of 995 patients with DKD and had a median 8 years of follow-up. Previously [19], we evaluated 13 a priori metabolites and their association with DKD progression. Here, using a high-throughput untargeted platform, we examined relative abundance of 698 annotated metabolite ions and implemented machine learning and rigorous statistical approaches to identify markers of DKD progression, rate of annual eGFR change, and ESKD. Further, we conducted a targeted metabolomics analysis on a subset of 15 amino acids. By implementing a robust multifaceted statistical approach, two metabolomic assays, and two clinically important outcomes, we aimed to identify potentially multiple sets of novel features prognostic for DKD progression and/or elucidate biological pathways of DKD.

## Methods

### Study Cohort

We used a metabolomics substudy of the Chronic Renal Insufficiency Cohort (CRIC). The parent CRIC study recruited (from 2003 on) a racially diverse group aged 21–74 years, ~50% diabetic, and with a broad range of kidney function [20]. Informed consent was obtained from participants; protocols were approved by the IRBs and Scientific and Data Coordinating Center (approval # 807882). The current study analyzed the urine metabolome at study entry (baseline) of 995 randomly selected CRIC participants with diabetes across CKD stages 3a, 3b, and 4, eGFR 45–60, 30–45, and 20–30 mL/min/1.73 m^2^, respectively.

### Disease Outcomes

We evaluated two outcomes: annual rate of eGFR change (eGFR slope) and time-to-ESKD. The eGFR slope was estimated via mixed models using serial eGFR measures as described previously [19]. Time-to-ESKD was the time from entry to the CRIC study to incident kidney failure with need for renal replacement therapy or kidney transplantation; drop-out or death before kidney failure was considered censoring events.

We used the CKD-EPI equation to calculate eGFR [21]. Results were consistent (data not shown) when we repeated analyses using the CRIC-eGFR equations [22], so we report findings using the widely used CKD-EPI equation.

### Statistical Analysis

#### Overview of Analytic Steps (Fig. 1)

Details of the assay, sample processing, and feature extraction are given in online supplementary materials (for all online suppl. material, see www.karger.com/doi/10.1159/000521940). We implemented stringent filtering to exclude metabolite ions with high noise or low biological variability. We then tested single metabolite associations with DKD outcomes, correcting for multiple comparisons, with and without clinical variables adjustment. Next, we developed multivariate metabolite models, using statistical and machine learning methods to identify marker signatures associated with eGFR slope, and further tested these models on time-to-ESKD. A unique aspect of our approach is that we did not train models on the ESKD outcome; thus, ESKD results serve as “internal replication” for a long-term clinical outcome. Finally, we conducted pathway enrichment analyses to investigate biological underpinnings of selected metabolites and conducted a targeted analysis of a subset of metabolites.

#### Filtering Metabolite Ions

Leveraging technical replicates, we used QC and CRIC samples to eliminate ions with poor reliability in the untargeted analysis (online suppl. materials). Of 1,899 annotated metabolite ions, the 698 which passed filtering criteria are the final metabolite ion set for all subsequent analyses. A single ion could annotate multiple metabolites; we will clarify these resulting ambiguities as appropriate.

#### Associations of Single Metabolite with Outcomes

We tested associations between each of the 698 log2-transformed metabolite ions and outcomes, with and without adjustment for clinical variables: age, gender, race, smoking, baseline BMI, mean arterial pressure, HbA1c, eGFR, and albuminuria [19]. For the eGFR slope, we calculated Pearson correlations and fit linear models; for ESKD, we fit Cox models. We used the Benjamini-Hochberg false discovery rate to correct for multiple comparisons [23].

#### Multivariate Models for the eGFR Slope

Using eGFR slopes as outcome, penalized regression (via lasso) and machine learning (via random forest) models were developed to elicit multivariate prognostic metabolomic signatures. The lasso reduces overfitting by imposing a penalty (λ) [24]. We considered two λ values chosen by 10-fold cross-validation: λ.min, the value yielding the lowest prediction error; and λ.1se, the value within one SD of lowest prediction error. Four lasso models were fit; each included 698 ions and 9 clinical variables as covariates. Two models forced all 9 clinical variables to be included, utilizing either λ.min or λ.1se. The other two models did not force the clinical variables to be included. For random forests, we used percent increase of mean squared error to order variable importance [24]; for comparability, we selected the same number of variables as the corresponding lasso models (without forcing clinical variables). Thus, we fit six multiple metabolite ion models: four lasso and two random forest models, each of which selected an optimal predictor set. As a final sensitivity analysis, we also fit four elastic net models [24], which can select groups of correlated features and may better mimic biological pathways.

#### Internal Replication on ESKD Outcome

To evaluate the models on time-to-ESKD, we fit six Cox models, in which predictors were variables selected in the six eGFR slope outcome analyses. We used likelihood ratio tests to compare each Cox model to its corresponding model of only clinical variables. To quantify model performance, we used 5-fold cross validation repeated 100 times to estimate mean and 95% CI of the C-statistic. Predictors used in these six Cox models were the predictors selected in the corresponding eGFR slope models; no tuning or variable selection was used in the Cox models. We intentionally avoided further training on ESKD to assess if predictors of the eGFR slope were also predictive of long-term outcomes (i.e., ESKD).

#### Enrichment Analysis

Definitions of well-known biological pathways by their respective involved metabolites were obtained from HMDB [25]. We considered 743 pathways and performed a hypergeometric test for each pathway definition via

$$p\;\text{value}=f(x|M,K,N)=\frac{\left(\begin{array}{l} K\\ x \end{array}\right)\left(\begin{array}{c} M-K\\ N-x \end{array}\right)}{\left(\begin{array}{l} M\\ N \end{array}\right)},$$

where M=# of measured metabolite ions, K=# of measured compounds in the pathway definition, N=# of “hits,” i.e., selected/prognostic metabolite ions, and x=# of “hits” that mapped to the pathway. The p values were corrected for multiple testing using the Benjamini-Hochberg method.

#### Assay Validation

Using a targeted assay, we validated selection and annotation of 15 metabolites detected by untargeted analysis, which were prognostic in the ESKD analysis. Again, we fit Cox models, adjusted for 9 clinical variables. To compare untargeted and targeted analyses, we present standardized hazard ratios (HRs).

## Results

### Participant Characteristics

At entry (Table 1), participants (*n* = 995) were mean 59.9 years, 56% male, 44% white, and 42% black; on average (mean [SD]), they were obese (BMI 34.2 [7.9]), had poor diabetes control (HbA1c 7.6 [1.5]%), and had moderate-to-poor kidney function (eGFR 40.6 [11.2] mL/min/1.73 m^2^). The eGFR decline (slope) averaged 1.8 (SD = 1.9) mL/min/1.73 m^2^/year; 36% (*N* = 360) had ESKD during the 10-year study (range: 2–10 years). We excluded subjects with missing clinical variables (<2%).

### Associations between Single Metabolites and Disease Outcomes

Of 698 ions, 89 were significantly correlated with eGFR slopes without adjustment for clinical variables (false discovery rate corrected *p* < 0.05). In adjusted analyses, 6 ions remained significant with β-coefficients from −0.45 to 0.3 (online suppl. Table S1). Also, 123 (unadjusted) and 99 (adjusted) ions were significantly associated with ESKD in Cox regression. After adjustment, HRs ranged from 1.12 to 1.84 (online suppl. Table S2). The prognostic ion set contained several amino acids (valine, isoleucine, and tryptophan) and other compounds, e.g., hydroxybutanoic acid (online suppl. Table S2). Only one ion, annotated as 3-(4-methyl-3-pentenyl)thiophene (Ion Index 1098, online suppl. Tables S1, S2), was associated with both eGFR slope and ESKD in adjusted models with coefficients (95% CI) of −0.44 (−0.68, −0.21) for the eGFR slope and HR (95% CI) 1.84 (1.45, 2.32), indicating that higher abundance of this compound might be associated with worse DKD progression. Complete results are in online supplementary Tables S1, S2. Henceforth, the 99 ESKD-associated metabolites will be denoted the 99-ESKD-associated set.

### Multivariable Prognostic Metabolites for eGFR Slope Outcome

Each of the lasso or random forest models (online suppl. Table S3) selected 9–30 variables resulting in 49 (out of 698) ions across the 6 prognostic models (online suppl. Table S4), denoting the 49-eGFR-associated set. Baseline albuminuria, blood pressure, and HbA1c were selected in all 6 models, and unsurprisingly, higher levels of these clinical markers were associated with steeper eGFR decline; race was also selected in all 6 models; 3,4-dicaffeoyl-1,5-quinolactone was selected in all models except model 1 (clinical only model). Nine ions, annotated as 3,4-dicaffeoyl-1,5-quinolactone, butynal, 3-(4-methyl-3-pentenyl)thiophene, C10:3, zalcitibine, asparaginyl-hydroxyproline, valine (or betaine), argynil-glutamine, and pentose (or di-isopropyl disulfide), were selected in at least 3 models; an additional 13 ions were selected in at least 2 models (online suppl. Table S4). Elastic net models had similar C-statistics (data not shown), so the more parsimonious lasso models were retained.

### Internal Replication

The final selected features from each of the 6 eGFR slope models (online suppl. Table S3) were tested on the ESKD outcome in Cox models. The likelihood ratio test p values were < 0.0001 when each of the 6 models was compared to the corresponding model with only clinical features, i.e., adding metabolite ions improved model fit significantly. Several ESKD models had similar median C-statistics (online suppl. Fig. S1), ranging from 0.82 to 0.85. The best (model 2, online suppl. Table S3) with 29 variables had a cross-validated median (95% CI) C-statistic of 0.85 (0.85, 0.86). This model selected 20 metabolite ions via lasso; 14 were significantly associated with the eGFR slope as evidenced by their bootstrap 95% CIs which excluded 0 (Table 2). Of greater interest are the five ions significantly (5% level) associated with time-to-ES-KD. Higher abundance of valine (or betaine) and 3-(4-methyl-3-pentenyl)thiophene was each associated with increased risk of ESKD (HR 1.44 and 1.65, respectively); higher asparaginyl-hydroxyproline abundance was associated with lower ESKD risk (HR = 0.7). Two other significantly associated compounds were pipazethate and aminophylline. Importantly, since our lasso model was not trained on ESKD outcome, we expect the Cox model results to be valid and not influenced by model selection. We also ran sensitivity analysis including ACE inhibitor or ARB use in Cox regression for model 2. There were negligible changes in the Cox model coefficients for the 29 variables in model 2; the HR (95% CI) for ACE inhibitor or ARB use was 1.22 (0.92, 1.63, *p* = 0.17), and this was not statistically significant at 5% significance level.

### Enrichment Analysis

We conducted pathway enrichment based on the 49-eGFR-associated (online suppl. Tables S3, S4), the 99-ESKD-associated (online suppl. Table S2), and the combined 49- and 99-sets (=131 ions). Thus, in equation (1), *M* = 698; *N* = 49 or 99 or 131; K varied by pathway; and x=# of ions in a pathway and also in the prognostic 49-, 99-, or 131-set.

Pathways (online suppl. Fig. S2; Table S5) consistently and significantly enriched across both eGFR slope and ESKD models were enzyme deficiencies, acidurias and acidemias, and those related to amino acid metabolism. Of interest, the valine-leucine-isoleucine degradation pathway involved in insulin resistance, cardiometabolic risk, cardiomyopathy, and CKD [26, 27]; the 2-aminoadipic 2-oxoadipic aciduria and lysine degradation pathways implicated in diabetes and kidney disease [28, 29]; and the tryptophan pathway [30–32] was enriched in the 99-ESKD and combined 131-ion sets (online suppl. Fig. S2; Table S5).

### Validation by Targeted CE-MS Approach

**Fig. 2.**
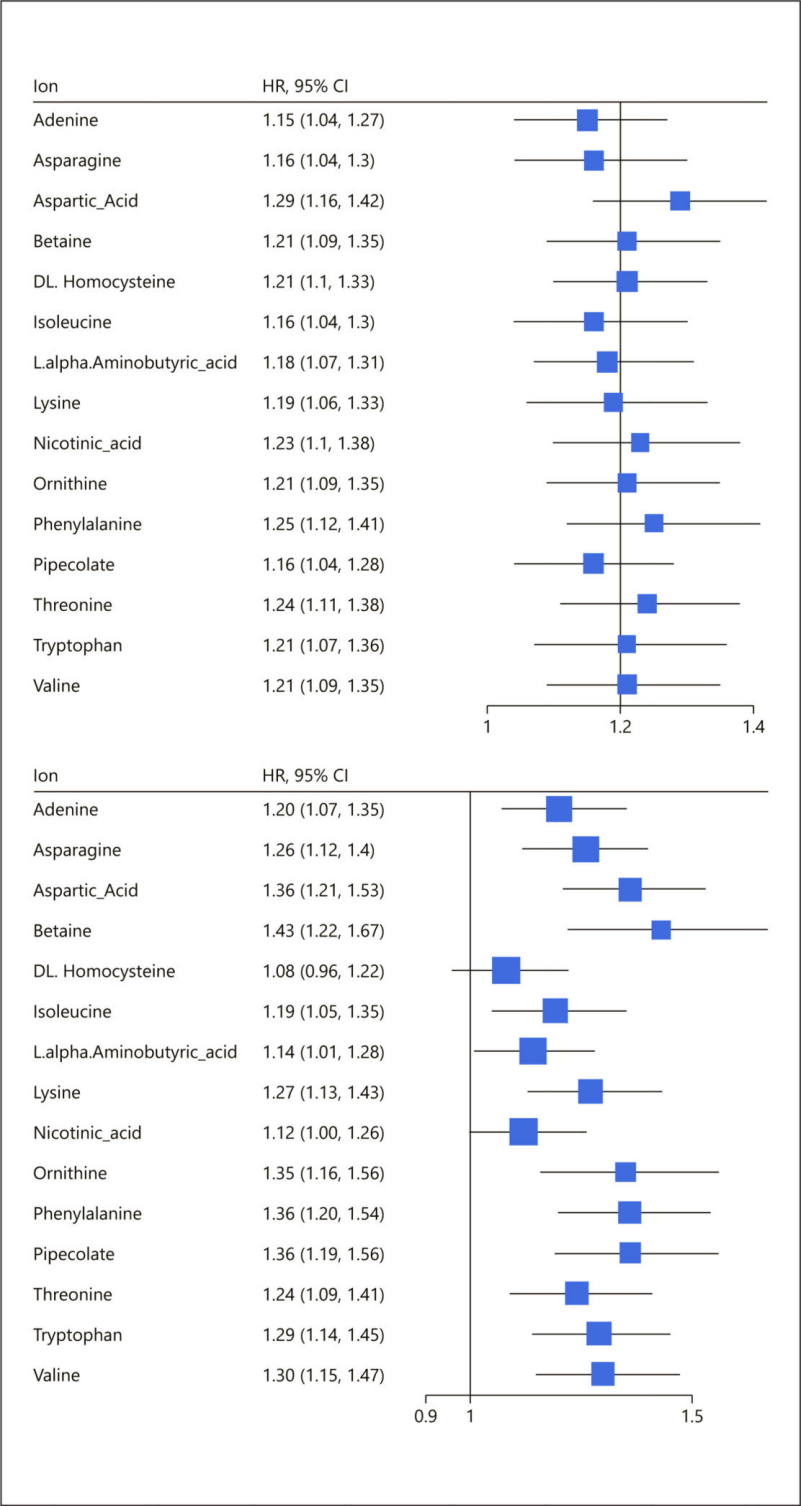
Forest plot (hazard ratios with 95% CIs) of ESKD risk of 15 untargeted and targeted metabolites. Above: untargeted assay; Below: targeted assay. The metabolite abundances were log2 transformed and then standardized by dividing by their SDs (standard deviation). Cox regression models are fitted using time to ESKD as outcome, each metabolite entered in the models separately with adjustment for 9 clinical variables. Note: In the untargeted assay (above), valine and betaine represent the same ion, due to ambiguities in annotation assignments. In the targeted quantitative analysis (below), these two compounds have separate annotations.

In the targeted validation analysis, 13 of 15 metabolites were significantly associated with ESKD (Fig. 2). HR (95% CI) in the untargeted and targeted analysis was comparable.

## Conclusions and Discussion

High-throughput metabolomics offers great potential, yet the scale and measurement errors raise analytic and inferential challenges. To address these challenges, we implemented two steps: (1) identifying a sparse prognostic set of metabolites after adjusting for known clinical factors and (2) using pathway analysis to elicit biological meaning. First, using an untargeted assay, we tested single metabolite associations with eGFR slope and ESKD. After adjusting for clinical variables, 6 and 99 (online suppl. Tables S1, S2) metabolite ions were found to exhibit non-null associations with eGFR slope and ESKD, respectively. An interesting finding is that for the eGFR slope, adjusting for clinical factors greatly reduced the number of non-null associations (from 89 to 6 ions), whereas for ESKD, the number of prognostic metabolites was relatively similar with and without adjustment (123 unadjusted to 99 adjusted), suggesting that clinical factors may be more critical for proximal (e.g., eGFR slope) versus long-term (e.g., ESKD) outcomes. Of note, differences in prognostic factors for these outcomes have also been previously reported [33].

As a next step, we conducted a quantitative targeted assay of 15 amino acid metabolism metabolites from the 99 (untargeted) ions and validated the prognostic value of 13 (of 15). Amino acid metabolism is known to be associated with DKD in cross-sectional analysis [12], and in a few prospective studies [7, 11, 34], but these studies have generally had small sample sizes (<100) and/or evaluated specific subgroups (e.g., type I diabetes). Thus, our finding of the prognostic value of urine amino acids in the large CRIC study even after adjustment for clinical factors adds to the literature and suggests that additional amino aciduria, independent of albuminuria, may be a risk factor for DKD progression.

In multivariable analysis, we fit 6 multiple metabolite models (adjusted for clinical factors) using robust penalized regression and machine learning. These modeling approaches are ideally suited to BigData with a large number of possibly correlated predictors. We identified sets of 6–25 prognostic metabolite ions (online suppl. Tables 2, S3). Nine unique ions were selected in ≥3 models. Two of these ions, asparginyl-hydroxyproline, implicated in iron deficiency and Crohn’s disease [35], and arginyl glutamine, a dipeptide shown to be helpful for treating hypoxia-induced small intestine injury [36], are likely novel findings in DKD research. In addition, we identified valine, a glucogenic branched chain amino acid, and C10:3, an acyl-carnitine whose blood levels were associated with heart failure and maternal and newborn metabolic health [37, 38]. The rest were food or drug derivatives [39, 40], e.g., 3-(4-methyl-3-pentenyl)thiophene found in alcoholic beverages. For ESKD, the 6 models had similar C-statistics (range 0.82–0.85), comparable to the 0.84 C-statistic of the clinical variables-only model, i.e., adding metabolites did not improve model discrimination. The fact that multiple models had similar discrimination is not surprising given the complexity of DKD. It is likely that there are many biological pathways in DKD, and thus potentially multiple “equally” prognostic models encompassing metabolite subsets from each of these pathways. Importantly, in the multiple adjusted Cox model with highest C-statistic, we identified several interesting prognostic (*p* < 0.05) metabolite ions. Valine (or betaine) and 3-(4-methyl3-pentenyl)thiophene were associated with 44% and 65% higher hazard of ESKD, respectively, per doubling of ion abundance. Importantly, the Cox model analysis, albeit on the same cohort, did not involve any training or variable selection, and hence HRs are likely less biased.

To systematically leverage the biological content of prognostic metabolite ions, we conducted enrichment analysis, using pathway, disease, and function definitions from the HMDB. We focused on metabolic pathway enrichment, since pathway definitions are less prone to curation anomalies, more finite, and well defined compared to disease and function definitions. We identified branched-chain amino acid pathways, namely, valine-leucine-isoleucine degradation, isovaleric acidemia/aciduria, propionic acidemia, and methylmalonic acidemia, all implicated in CKD [18, 26–29]. The tryptophan pathway was previously investigated in DKD primarily in serum/plasma samples [30–32] though findings were mixed. Our study found that higher levels of tryptophan were associated with higher ESKD risk in both untargeted and targeted analyses, and the tryptophan pathway was significantly enriched in our set of ESKD-related metabolites. To our knowledge, these findings are novel, and further investigation of urine tryptophan is warranted.

Our study fills several gaps in current DKD research. Previous urine metabolomics studies were cross-sectional, had small sample sizes, examined few single compounds, or have been restricted to subpopulations. In this work, we addressed these shortcomings. Our CRIC DKD sample is one of the largest in the USA, with comprehensive clinical data and long-term follow-up with annual assessments of kidney function. We conducted untargeted high-throughput metabolomics which identified 1,899 annotated ions, used stringent filtering to reduce to a reliable subset of 698 metabolite ions, and used rigorous paradigms to build cross-validated models to select metabolite ions prognostic for eGFR decline. The selected ions were then tested for association with ESKD without further training. While it could be argued that prognostic predictor sets may vary between the eGFR slope and ESKD outcomes [33], our approach of replicating findings on the ESKD outcome, rather than fitting separate ESKD models, is more statistically stringent. Of note, models were not trained on the ESKD outcome, which should reduce overfitting. Also, identifying predictors of short-term outcomes which also foretell long-term outcomes could be more impactful in the clinic for planning long-term disease management. However, we acknowledge that focusing feature selection solely on the ESKD outcome could be of value, if results could be validated, and we aim to pursue this line in future work in independent cohorts. Finally, we used a targeted CE-MS assay to validate and quantify a subset of “hits” identified in our untargeted analysis, illustrating the value of an untargeted high-throughput metabolomics approach despite the sample complexity of human urine.

There are limitations to our work. First, although we adjusted for known clinical factors, residual confounding and measurement error could still impact our findings. Results may also vary by clinical subgroups; we leave such investigation to future studies. Second, while our enrichment analysis revealed potential DKD-related pathways, our selected metabolites were also enriched for deficiencies associated with other diseases. Thus, the systematic pathway enrichment may not be specific to DKD, a limitation of pathway analyses when a given pathway could overlap multiple diseases. Finally, while we followed strict protocols for biospecimen storage, processing, and quality control, the age of our archived samples might also introduce errors. Also, while we validated a subset of metabolites with existing targeted approaches, it is necessary to develop quantitative assays to compare with our untargeted findings [41]. We are developing targeted assays for other prognostic markers for future validation. In addition, confirming (or refuting) our findings in independent cohorts using different assays will enhance generalizability.

In summary, we conducted large-scale untargeted and targeted metabolomics analysis using a diverse cohort and identified several metabolites implicated in DKD. We used robust statistical models incorporating rigorous techniques to reduce overfitting: cross-validation for feature selection, replicating results on a long-term clinical outcome, and validation using a targeted assay. While we highlighted and validated a few prognostic metabolites and related pathways, the most important aspect of our work is that we identified 131 potentially prognostic metabolites in our untargeted analysis, several of which are not addressed before. We hope that providing this list will spur further investigation of these promising metabolites and enhance understanding of DKD, eventually leading to better treatments and disease management.

## Supplementary Material

Zhang J Neph 2022 Metabolites

Zhang J Neph Methods and Table

## Figures and Tables

**Fig. 1.**
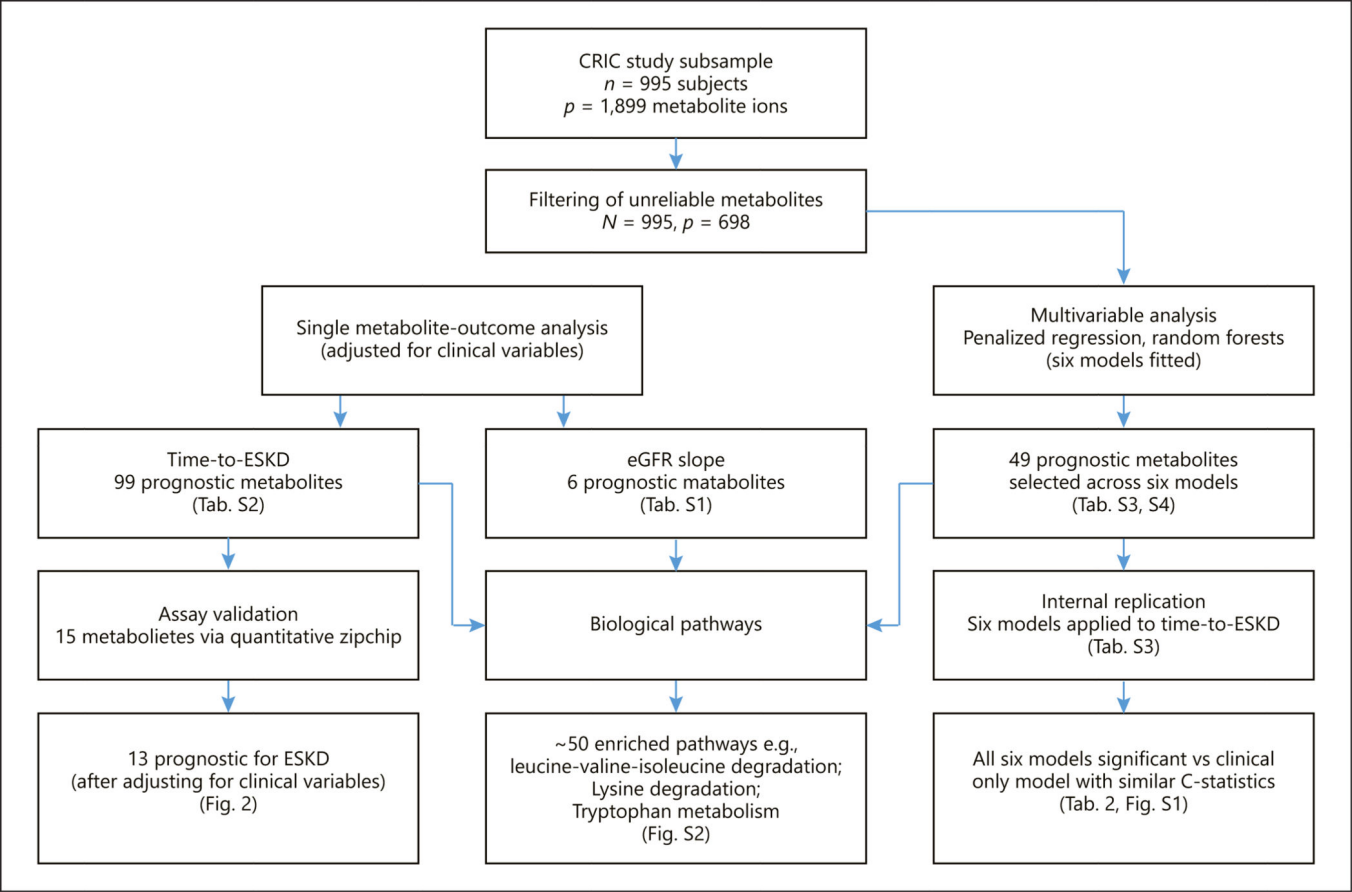
Flow diagram for analysis steps and brief overview of results.

**Table 1.** Baseline clinical characteristics of 995 metabolomics substudy participants with diabetes in the chronic renal insufficiency cohort (CRIC) study

Clinical variable	Mean ± SD/*N* (%)

Age, years	59.9±9.4
Sex	
Male	561 (56)
Female	434 (44)
Race	
White	440 (44)
Black	420 (42)
Others	135 (14)
Smoked 100 cigarettes	
Yes	565 (57)
No	430 (43)
BMI, kg/m^2^	34.2±7.9
Mean arterial pressure, mm Hg	90.0±13.4
eGFR, mL/min/1.73^2^	40.6±11.2
HbAlc, %	7.6±1.5
UACR, mg/d	
≤30	292 (30)
30–300	272 (28)
≥300	420 (43)
Continuous UACR, median (IQR),* mg/day	0.16 (0.92)
ACE inhibitor or ARB use	
Yes	799 (81)
No	193 (19)

BMI, body mass index; eGFR, estimated glomerular filtration rate; HbA1c, hemoglobin A1c; UACR, urine albumin-to-creatinine ratio; ACE, angiotensin-converting enzyme; ARB, angiotensin-receptor blocker.

* Continuous UACR is summarized using median (IQR, interquartile range) because of its skewed distribution. All other continuous variables are summarized using mean ± SD.

**Table 2.** Associations with DKD outcomes for the model with the largest C-statistic (model 2, online suppl. Table S3)

Predictor^§^: name or ion number	Linear lasso regression model outcome: eGFR slope*, ^†^		Cox model outcome: time-to-ESKD^†^		Metabolite ion annotation name
			
	selection percent	median (95% CI) of coefficients	hazard ratio (95% CI)	p value	

Age per 1 yr older	-	−0.0014 (−0.0144, 0.0108)	0.98 (0.97, 1)	0.006	
UACR, mg/g					
≤30	-	−0.8894 (−1.0449, −0.7312)	Ref	Ref	
30–300			3.86 (2.18, 6.81)	0^‡^	
≥300			11.57 (6.72, 19.91)	0^‡^	
BMI, per 1 kg/m^2^ greater	-	0.0007 (−0.0128, 0.0141)	0.99 (0.97,1)	0.098	
Baseline eGFR, per 1 mL/min/1.73 m^2^ greater	–	−0.0063 (−0.0184, 0.0076)	0.93 (0.91, 0.94)	0^‡^	
HbA1_c_, per 1 percentage point	–	−0.083 (−0.1599, −0.0071)	1.02 (0.95, 1.1)	0.56	
MAP, per 1 mm Hg greater	-	−0.0164 (−0.0247, −0.008)	1.02 (1.01, 1.03)	0^‡^	
Female sex	-	−0.4058 (−0.6916, −0.1411)	1.03 (0.8, 1.34)	0.802	
Smoke >100 cigarettes	-	−0.0344 (−0.259, 0.1859)	0.97 (0.77, 1.23)	0.806	
Race					
White	-	−0.1738 (−0.3528, −0.0153)	Ref	Ref	
Black			1.72 (1.28, 2.3)	0	
Others			1.39 (0.97, 1.97)	0.07	
**1,098**	**95%**	**−0.1296 (−0.2861, −0.0159)**	**1.65 (1.23, 2.2)**	**0.001**	**3-(4-Methyl-3-pentenyl)thiophene**
1,099	72%	−0.0878 (−0.2445, −0.001)	0.78 (0.58, 1.04)	0.089	C10:3
2,202	45%	−0.0447 (−0.2028, 0.0088)	1 (0.83,1.21)	0.988	Zalcitabine
2,513	49%	−0.0194 (−0.1078, 0.0557)	1.05 (0.91, 1.22)	0.518	4-(2-Amino-3-hydroxyphenyl)-2,4-dioxobutanoic acid
255	57%	0.1007 (0.0014, 0.3437)	1.05 (0.9, 1.22)	0.539	Aminophenol
281	56%	0.104 (0.0017, 0.2771)	1.12 (0.65, 1.93)	0.69	Furoic acid
30	43%	0.0932 (0.0043, 0.252)	0.64 (0.34, 1.23)	0.179	Butynal
**3,117**	**61%**	**−0.0666 (−0.2161, −9e−04)**	**0.71 (0.52, 0.98)**	**0.035**	**Asparaginyl-hydroxyproline**
3,165	52%	0.073 (7e−04, 0.28)	0.97 (0.91, 1.03)	0.339	2-Hydroxy-acetaminophen sulfate
**344**	**21%**	**−0.0126 (−0.124, 0.1478)**	**1.44 (1.12, 1.86)**	**0.005**	**Valine; betaine**
3,756	57%	−0.0578 (−0.2077, 0.0072)	1 (0.87, 1.15)	1	Neuraminic acid or adenosine
4,207	36%	−0.0353 (−0.1515, 0.0901)	1.16 (0.9, 1.5)	0.25	6-Thioinosinic acid
4,754	68%	−0.107 (−0.3121, −0.001)	1.08 (0.86, 1.36)	0.503	Arginyl-glutamine
5,388	92%	−0.1318 (−0.3354, −0.0077)	1.11 (0.92, 1.33)	0.278	Hydroxyhexamide
**7,244**	**59%**	**0.073 (3e−04, 0.2625)**	**0.88 (0.78, 0.99)**	**0.035**	**Pipazethate**
**7,671**	**72%**	**−0.0756 (−0.2443, −0.0023)**	**1.3 (1.01, 1.66)**	**0.044**	**Aminophylline**
798	85%	−0.1393 (−0.3178, −0.0023)	1.17 (0.84, 1.62)	0.353	Diisopropyl disulfide or pentose
8,590	54%	−0.0871 (−0.25, **−**3e−04)	1.03 (0.77, 1.39)	0.827	Dolichyl b-D-glucosyl phosphate
9,178	88%	0.1298 (0.0125, 0.3207)	0.99 (0.86, 1.15)	0.894	3,4-Dicaffeoyl-1,5-quinolactone
9,355	22%	−0.0296 (−0.1636, 0.111)	1.08 (0.79, 1.48)	0.626	Argatroban or 2-(2,4-dihydroxy-phenyl)-3-(3,7-dimethylocta-2,6-dien-1-yl)-5,7dihydroxy-6-(4-hydroxy-3-methylbut-2en-1-yl)-3,4-dihydro-2H-1-benzopyran-4one

Boldface indicates ions significant at the 5% level in the Cox model. A single ion can correspond to multiple metabolites, which resulted in some ambiguities for identifying metabolites, as noted in the last column.

* Coefficient from linear LASSO models. Bootstrapping resampling (500 times) was used to calculate 95% CIs; selection percent is the 100 × (# of times feature was selected/500); all clinical variables were forced into the model, and hence selection probability is not provided.

† Linear regression coefficients are per unit increase in (log_2_)-metabolite abundance. Hazard ratios are per doubling of metabolite abundance.

‡ p value of 0 means highly significant, i.e., <0.001.

§ Models were also adjusted for race.

## Data Availability

The data that support the findings of this study are available on request from the CRIC study at cristudy.org upon reasonable request.
