# Supplementary material for: High-Throughput Metabolomics and Diabetic Kidney Disease Progression: Evidence from the Chronic Renal Insufficiency (CRIC) Study
Source: Am J Nephrol. Author manuscript; Available in PMC 2023 Dec 7. (PMC9116599; doi:10.1159/000521940)
Supplement: Zhang J Neph 2022 Metabolites [file NIHMS1800823-supplement-Zhang_J_Neph_2022_Metabolites.pdf]

Ion Index	Database ID	Top-Name	Sum Formula	Detected Mass	Reliable.Ion
1	HMDB0041822; C01471; CHEBI:15368	Acrolein	C3H4O	55.01823771	No
6	HMDB0001659; C00207; CHEBI:15347	Acetone	C3H6O	57.03406158	No
6	HMDB0003366; C00479; CHEBI:17153	Propanal	C3H6O	57.03406158	No
6	HMDB0031558; C15508; CHEBI:38685	Methyloxirane	C3H6O	57.03406158	No
6	HMDB0031652; C02001; CHEBI:16605	Allyl alcohol	C3H6O	57.03406158	No
11	HMDB0001122; C11489; CHEBI:7438	N-Methylformamide	C2H5NO	58.02895255	No
11	HMDB0003656; C02658; CHEBI:50718	Acetaldehyde oxime	C2H5NO	58.02895255	No
11	HMDB0031645; C06244; CHEBI:27856	Acetamide	C2H5NO	58.02895255	No
12	HMDB0001842; C17349; CHEBI:42820	Guanidine	CH5N3	58.04033157	No
15	HMDB0000042; C00033; CHEBI:15366	Acetic acid	C2H4O2	59.01330173	Yes
15	HMDB0003344; C00266; CHEBI:17071	Glycolaldehyde	C2H4O2	59.01330173	Yes
18	HMDB0000820; C05979; CHEBI:28831	Propyl alcohol	C3H8O	59.04952873	No
18	HMDB0000863; C01845; CHEBI:17824	Isopropyl alcohol	C3H8O	59.04952873	No
23	HMDB0003538; C01353; CHEBI:28976	Carbonic acid	CH2O3	60.99255249	No
29	HMDB0031323	3-Butenenitrile	C4H5N	66.03463864	No
29	HMDB0035924; C19907; CHEBI:19203	Pyrrole	C4H5N	66.03463864	No
30	HMDB0006853; C06145; CHEBI:28180	3-Butyn-1-al	C4H4O	67.01860567	Yes
30	HMDB0013785; C00089; CHEBI:17992	Furan	C4H4O	67.01860567	Yes
31	HMDB0001525; C01589; CHEBI:16069	Imidazole	C3H4N2	67.02982178	No
39	HMDB0034233	(E)-2-Butenal	C4H6O	69.03439741	No
39	HMDB0061873; CHEBI:48058	3-Buten-2-one	C4H6O	69.03439741	No
39	HMDB0061874	Methacrolein	C4H6O	69.03439741	No
40	HMDB0004296; C01659; CHEBI:28619	Acrylamide	C3H5NO	70.02953081	No
42	HMDB0001167; C00546; CHEBI:17158	Pyruvaldehyde	C3H4O2	71.01354279	No
42	HMDB0006112; C19440; CHEBI:566274	Malondialdehyde	C3H4O2	71.01354279	No
42	HMDB0031647; C00511; CHEBI:18308	Acrylic acid	C3H4O2	71.01354279	No
43	HMDB0000246; CHEBI:26911	Tetrahydrofuran	C4H8O	71.0498917	No
43	HMDB0000474; C02845; CHEBI:28398	Butanone	C4H8O	71.0498917	No
43	HMDB0003543; C01412; CHEBI:15743	Butanal	C4H8O	71.0498917	No
43	HMDB0031243; C03219; CHEBI:48943	2-Methylpropanal	C4H8O	71.0498917	No
43	HMDB0031324	3-Buten-1-ol	C4H8O	71.0498917	No
43	HMDB0033858	2-Buten-1-ol	C4H8O	71.0498917	No
43	HMDB0034431	2,2-Dimethyloxirane	C4H8O	71.0498917	No
47	HMDB0001106; C05665; CHEBI:18090	3-Aminopropionaldehyde	C3H7NO	72.04524358	No
47	HMDB0001888; C03134; CHEBI:17741	N,N-Dimethylformamide	C3H7NO	72.04524358	No
47	HMDB0002134; C01888; CHEBI:17906	Aminoacetone	C3H7NO	72.04524358	No
47	HMDB0142435	2-(methylamino)acetaldehyde	C3H7NO	72.04524358	No
48	HMDB0000119; C00048; CHEBI:16891	Glyoxylic acid	C2H2O3	72.99287163	No
49	HMDB0031635	2-Propene-1-thiol	C3H6S	73.01148359	No
49	HMDB0040345	1-Propene-1-thiol	C3H6S	73.01148359	No
50	HMDB0000237; C00163; CHEBI:30768	Propionic acid	C3H6O2	73.02933072	No
50	HMDB0003052; C00424; CHEBI:18041	Lactaldehyde	C3H6O2	73.02933072	No
50	HMDB0003453; C00969; CHEBI:17871	3-Hydroxypropanal	C3H6O2	73.02933072	No
50	HMDB0006458; C00937; CHEBI:17167	D-Lactaldehyde	C3H6O2	73.02933072	No
50	HMDB0006961; C05235; CHEBI:27957	Hydroxyacetone	C3H6O2	73.02933072	No
50	HMDB0031229; CHEBI:52342	Ethyl formate	C3H6O2	73.02933072	No
50	HMDB0031523; C17530	Methyl acetate	C3H6O2	73.02933072	No
52	HMDB0000123; C00037; CHEBI:15428	Glycine	C2H5NO2	74.02406512	No
52	HMDB0014691; C06808; CHEBI:27777	Acetohydroxamic Acid	C2H5NO2	74.02406512	No
52	HMDB0031239	Ethyl nitrite	C2H5NO2	74.02406512	No
54	HMDB0000115; C00160; CHEBI:17497	Glycolic acid	C2H4O3	75.00856935	No
54	HMDB0031608	Peracetic acid	C2H4O3	75.00856935	No
57	HMDB0002991; C01678; CHEBI:17141	Cysteamine	C2H7NS	76.02257138	No
62	HMDB0001505; C01407; CHEBI:16716	Benzene	C6H6	77.03899612	No
68	HMDB0000926; C00747; CHEBI:16227	Pyridine	C5H5N	78.03480809	No
79	HMDB0001033; C11481; CHEBI:17137	Hydrogen sulfite	HO3S	79.9572718	No
86	HMDB0013749; CHEBI:969439	2-Methylfuran	C5H6O	81.03446392	No
86	HMDB0031597	2,4-Pentadienal	C5H6O	81.03446392	No
95	HMDB0004363; C06195; CHEBI:51023	Imidazolone	C3H4N2O	83.02511764	No

98	HMDB0001184	Methyl propenyl ketone	C5H8O	83.05016892	No
98	HMDB0012157; C07330; CHEBI:15825	3-Methyl-2-butenal	C5H8O	83.05016892	No
98	HMDB0031407; C00557; CHEBI:16486	Cyclopentanone	C5H8O	83.05016892	No
98	HMDB0031512; CHEBI:88419	Tiglic aldehyde	C5H8O	83.05016892	No
98	HMDB0031530	3-Methyl-3-buten-2-one	C5H8O	83.05016892	No
98	HMDB0031601	2-Pentenal	C5H8O	83.05016892	No
98	HMDB0031606; CHEBI:87508	4-Penten-2-one	C5H8O	83.05016892	No
98	HMDB0031607	1-Penten-3-one	C5H8O	83.05016892	No
98	HMDB0032458	4-Pentenal	C5H8O	83.05016892	No
98	HMDB0037788; CHEBI:51679	xi-2,3-Dihydro-3-methylfuran	C5H8O	83.05016892	No
98	HMDB0040584; CHEBI:51678	2,3-Dihydro-4-methylfuran	C5H8O	83.05016892	No
101	HMDB0002039; C11118; CHEBI:36592	2-Pyrrolidinone	C4H7NO	84.04550518	No
101	HMDB0060427; CHEBI:15348	Acetone cyanohydrin	C4H7NO	84.04550518	No
105	HMDB0033875	2,3-Dihydrothiophene	C4H6S	85.01271236	No
105	HMDB0033922	Divinyl sulfide	C4H6S	85.01271236	No
106	HMDB0000549; C01770; CHEBI:42639	Gamma-Butyrolactone	C4H6O2	85.02930871	No
106	HMDB0002523	Oxolan-3-one	C4H6O2	85.02930871	No
106	HMDB0003407; C00741; CHEBI:16583	Diacetyl	C4H6O2	85.02930871	No
106	HMDB0010720; C01771; CHEBI:41131	But-2-enoic acid	C4H6O2	85.02930871	No
106	HMDB0031209; C19309; CHEBI:46916	Ethenyl acetate	C4H6O2	85.02930871	No
106	HMDB0033977; C19443	Methyl acrylate	C4H6O2	85.02930871	No
106	HMDB0034439; CHEBI:36253	Isocrotonic acid	C4H6O2	85.02930871	No
107	HMDB0006478; C07329; CHEBI:16638	Iso-Valeraldehyde	C5H10O	85.06583185	No
107	HMDB0030124; C01390; CHEBI:16019	Prenol	C5H10O	85.06583185	No
107	HMDB0030126; CHEBI:62898	3-Methyl-3-buten-1-ol	C5H10O	85.06583185	No
107	HMDB0031206; CHEBI:121517	Pentanal	C5H10O	85.06583185	No
107	HMDB0031525; CHEBI:88414	(S)-2-Methylbutanal	C5H10O	85.06583185	No
107	HMDB0031526; C02223; CHEBI:16182	(±)-2-Methylbutanal	C5H10O	85.06583185	No
107	HMDB0031604	(E)-2-Penten-1-ol	C5H10O	85.06583185	No
107	HMDB0031605	1-Penten-3-ol	C5H10O	85.06583185	No
107	HMDB0033854; CHEBI:132752	Isoprenyl alcohol	C5H10O	85.06583185	No
107	HMDB0034235; C01949; CHEBI:16472	2-Pentanone	C5H10O	85.06583185	No
107	HMDB0034465	2-Methyl-2-buten-1-ol	C5H10O	85.06583185	No
107	HMDB0039779	xi-3-Methyl-3-buten-2-ol	C5H10O	85.06583185	No
110	HMDB0003609; C02218; CHEBI:17123	2-Aminoacrylic acid	C3H5NO2	86.02464259	No
110	HMDB0039426	3-Isoxazolidinone	C3H5NO2	86.02464259	No
112	HMDB0001080; C00555; CHEBI:17769	4-Aminobutyraldehyde	C4H9NO	86.06119331	No
112	HMDB0031215; CHEBI:87370	N-Ethylacetamide	C4H9NO	86.06119331	No
112	HMDB0031581; C14452; CHEBI:34856	Morpholine	C4H9NO	86.06119331	No
112	HMDB0033870; CHEBI:50724	Butyramide	C4H9NO	86.06119331	No
112	HMDB0142479	2-(dimethylamino)acetaldehyde	C4H9NO	86.06119331	No
114	HMDB0000243; C00022; CHEBI:32816	Pyruvic acid	C3H4O3	87.00871685	Yes
114	HMDB0011111; C00222; CHEBI:17960	Malonic semialdehyde	C3H4O3	87.00871685	Yes
114	HMDB0040261	Glucosereductone	C3H4O3	87.00871685	Yes
114	HMDB0062676; CHEBI:86354	2-hydroxyacrylic Acid	C3H4O3	87.00871685	Yes
115	HMDB0000039; C00246; CHEBI:30772	Butyric acid	C4H8O2	87.04513658	No
115	HMDB0001873; C02632; CHEBI:16135	Isobutyric acid	C4H8O2	87.04513658	No
115	HMDB0003243; C00466; CHEBI:15688	Acetoin	C4H8O2	87.04513658	No
115	HMDB0030062	Methyl propionate	C4H8O2	87.04513658	No
115	HMDB0031217; C00849; CHEBI:27750	Ethyl acetate	C4H8O2	87.04513658	No
115	HMDB0031507	1-Hydroxy-2-butanone	C4H8O2	87.04513658	No
115	HMDB0040253	Propyl formate	C4H8O2	87.04513658	No
115	HMDB0040579	Isopropyl formate	C4H8O2	87.04513658	No
116	HMDB0143055	(hydroxymethylidene)carbamic acid	C2H3NO3	88.00376349	No
118	HMDB0000056; C00099; CHEBI:16958	Beta-Alanine	C3H7NO2	88.04057358	No
118	HMDB0000161; C00041; CHEBI:16977	L-Alanine	C3H7NO2	88.04057358	No
118	HMDB0000271; C00213; CHEBI:15611	Sarcosine	C3H7NO2	88.04057358	No
118	HMDB0001310; C00133; CHEBI:15570	D-Alanine	C3H7NO2	88.04057358	No
118	HMDB0031219; C01537; CHEBI:17967	Ethyl carbamate	C3H7NO2	88.04057358	No
118	HMDB0062251; C01401; CHEBI:16449	(alpha-D-mannosyl)7-beta-D-mannosyl-diacetylchitobi	C3H7NO2	88.04057358	No
119	HMDB0002329; C00209; CHEBI:16995	Oxalic acid	C2H2O4	88.98799467	No

120	HMDB0030025	1-Mercapto-2-propanone	C3H6OS	89.00674682	No
120	HMDB0031189; CHEBI:51280	S-Methyl thioacetate	C3H6OS	89.00674682	No
120	HMDB0031636	(E)-1-Propene-1-sulfenic acid	C3H6OS	89.00674682	No
120	HMDB0031718; CHEBI:50716	(Methylthio)acetaldehyde	C3H6OS	89.00674682	No
120	HMDB0040346; C08389; CHEBI:8475	Thial-1-Propene-1-thiol S-oxide	C3H6OS	89.00674682	No
122	HMDB0000190; HMDB0062492; C00186; CHI	L-Lactic acid	C3H6O3	89.0242228	No
122	HMDB0000700; C01013; CHEBI:33404	Hydroxypropionic acid	C3H6O3	89.0242228	No
122	HMDB0001051; C02154; CHEBI:5445	Glyceraldehyde	C3H6O3	89.0242228	No
122	HMDB0001311; C00256; CHEBI:341	D-Lactic acid	C3H6O3	89.0242228	No
122	HMDB0001882; C00184; CHEBI:16016	Dihydroxyacetone	C3H6O3	89.0242228	No
122	HMDB0029580; CHEBI:36596	Dimethyl carbonate	C3H6O3	89.0242228	No
122	HMDB0031232; CHEBI:50849	Monoethyl carbonate	C3H6O3	89.0242228	No
122	HMDB0041929; CHEBI:132098	Methoxyacetic acid	C3H6O3	89.0242228	No
122	HMDB0144295	2-hydroxypropanoic acid	C3H6O3	89.0242228	No
123	HMDB0031245	2-Methyl-1-propanethiol	C4H10S	89.04395	No
123	HMDB0031322	1-Butanethiol	C4H10S	89.04395	No
123	HMDB0031666; C02272; CHEBI:27710	Diethyl sulfide	C4H10S	89.04395	No
123	HMDB0040576	2-(Methylthio)propane	C4H10S	89.04395	No
123	HMDB0061871	1-(Methylthio)-propane	C4H10S	89.04395	No
126	HMDB0034168; C01455; CHEBI:17578	Toluene	C7H8	91.05507469	No
128	HMDB0003012; C00292; CHEBI:17296	Aniline	C6H7N	92.05082483	No
128	HMDB0061887; CHEBI:39922	3-Methylpyridine	C6H7N	92.05082483	No
128	HMDB0061888; C14447; CHEBI:50415	2-Methylpyridine	C6H7N	92.05082483	No
132	HMDB0000228; C00146; CHEBI:15882	Phenol	C6H6O	93.03465738	Yes
133	HMDB0033112	Methylpyrazine	C5H6N2	93.04544107	No
136	HMDB0013751; C02502; CHEBI:16540	2-Hydroxypyridine	C5H5NO	94.03005126	Yes
136	HMDB0036057; CHEBI:59978	1H-Pyrrole-2-carboxaldehyde	C5H5NO	94.03005126	Yes
144	HMDB0032914; C14279	2-Furancarboxaldehyde	C5H4O2	95.01383545	No
144	HMDB0060503; CHEBI:28906	Protoanemonin	C5H4O2	95.01383545	No
148	HMDB0031545	2-Methyl-2-cyclopenten-1-one	C6H8O	95.05022735	No
148	HMDB0031546	3-Methyl-2-cyclopenten-1-one	C6H8O	95.05022735	No
148	HMDB0032965	2,4-Dimethylfuran	C6H8O	95.05022735	No
148	HMDB0033182	2,5-Dimethylfuran	C6H8O	95.05022735	No
148	HMDB0034729	(E,E)-2,4-Hexadienal	C6H8O	95.05022735	No
148	HMDB0040587	2-Ethylfuran	C6H8O	95.05022735	No
148	HMDB0040588	3-Ethylfuran	C6H8O	95.05022735	No
157	HMDB0031339; CHEBI:1178473	1-Cyano-2-hydroxy-3-butene	C5H7NO	96.04548441	No
157	HMDB0032968	2,4-Dimethyloxazole	C5H7NO	96.04548441	No
157	HMDB0032969	2,5-Dimethyloxazole	C5H7NO	96.04548441	No
157	HMDB0032970	4,5-Dimethyloxazole	C5H7NO	96.04548441	No
167	HMDB0013742; CHEBI:207496	2-Furanmethanol	C5H6O2	97.02953838	Yes
167	HMDB0029609; CHEBI:36436	5-Methyl-2(3H)-furanone	C5H6O2	97.02953838	Yes
167	HMDB0040145	5-Hydroxy-4-pentenoic acid d-lactone	C5H6O2	97.02953838	Yes
179	HMDB0005843; C19317; CHEBI:494804	Allyl isothiocyanate	C4H5NS	98.00713126	No
179	HMDB0029755	3-Thiocyanato-1-propene	C4H5NS	98.00713126	No
179	HMDB0033116; CHEBI:35626	4-Methylthiazole	C4H5NS	98.00713126	No
179	HMDB0033117	5-Methylthiazole	C4H5NS	98.00713126	No
179	HMDB0038624	Thiiraneacetonitrile	C4H5NS	98.00713126	No
179	HMDB0061872	Isothiocyanatocyclopropane	C4H5NS	98.00713126	No
181	HMDB0030276	(R)-Dihydromaleimide	C4H5NO2	98.02484942	No
184	HMDB0011749; CHEBI:110273	2-Piperidinone	C5H9NO	98.0610988	No
184	HMDB0034587	1-Pyrrolidinecarboxaldehyde	C5H9NO	98.0610988	No
184	HMDB0040518	2,5-Dihydro-2,4-dimethyloxazole	C5H9NO	98.0610988	No
184	HMDB0060309; C18796; CHEBI:60908	(2R)-2-Hydroxy-2-methylbutanenitrile	C5H9NO	98.0610988	No
196	HMDB0029555	4,5-Dihydro-2-methylthiazole	C4H7NS	100.0226254	No
197	HMDB0029387; C02926; CHEBI:17289	L-3-Aminodihydro-2(3H)-furanone	C4H7NO2	100.0405938	No
197	HMDB0029615; CHEBI:38108	(S)-2-Azetidinecarboxylic acid	C4H7NO2	100.0405938	No
197	HMDB0036458; C01234; CHEBI:18053	1-Aminocyclopropanecarboxylic acid	C4H7NO2	100.0405938	No
199	HMDB0012815; C12455	5-Aminopentanal	C5H11NO	100.0766898	No
199	HMDB0062600; CHEBI:84287	N-butylformamide	C5H11NO	100.0766898	No
204	HMDB0000005; C00109; CHEBI:30831	2-Ketobutyric acid	C4H6O3	101.0245281	No

204	HMDB0000060; C00164; CHEBI:15344	Acetoacetic acid	C4H6O3	101.0245281	No
204	HMDB0001172; C00349; CHEBI:16256	2-Methyl-3-oxopropanoic acid	C4H6O3	101.0245281	No
204	HMDB0001259; C00232; CHEBI:16265	Succinic acid semialdehyde	C4H6O3	101.0245281	No
204	HMDB0002217; C06002; CHEBI:27821	(S)-Methylmalonic acid semialdehyde	C4H6O3	101.0245281	No
204	HMDB0003381; CHEBI:553775	4-Hydroxycrotonic acid	C4H6O3	101.0245281	No
204	HMDB0031646; CHEBI:36610	Acetic anhydride	C4H6O3	101.0245281	No
205	HMDB0000718; C08262; CHEBI:28484	Isovaleric acid	C5H10O2	101.060761	No
205	HMDB0000892; C00803; CHEBI:17418	Valeric acid	C5H10O2	101.060761	No
205	HMDB0002176; C18319; CHEBI:37070	Ethylmethylacetic acid	C5H10O2	101.060761	No
205	HMDB0030058; CHEBI:167460	Ethyl propionate	C5H10O2	101.060761	No
205	HMDB0031175; CHEBI:137944	Tetrahydro-2-furanmethanol	C5H10O2	101.060761	No
205	HMDB0031241; CHEBI:1132558	Isopropyl acetate	C5H10O2	101.060761	No
205	HMDB0031247	2-Methylpropyl formate	C5H10O2	101.060761	No
205	HMDB0031249; CHEBI:73689	Methyl isobutyrate	C5H10O2	101.060761	No
205	HMDB0031516	3-Hydroxy-2-pentanone	C5H10O2	101.060761	No
205	HMDB0032230	2,4-Dimethyl-1,3-dioxolane	C5H10O2	101.060761	No
205	HMDB0032263; CHEBI:32026	Ethylene oxide/propylene oxide copolymer	C5H10O2	101.060761	No
205	HMDB0033742; CHEBI:38655	(S)-2-Methylbutanoic acid	C5H10O2	101.060761	No
205	HMDB0033890	Methyl butyrate	C5H10O2	101.060761	No
205	HMDB0034237; CHEBI:40116	Propyl acetate	C5H10O2	101.060761	No
205	HMDB0039799	Tetrahydro-2-methyl-3-furanol	C5H10O2	101.060761	No
205	HMDB0040575; CHEBI:88514	Butyl formate	C5H10O2	101.060761	No
205	HMDB0041992; CHEBI:45133	Pivalic acid	C5H10O2	101.060761	No
205	HMDB0059678	1-Hydroxy-2-pentanone	C5H10O2	101.060761	No
215	HMDB0000092; C01026; CHEBI:17724	Dimethylglycine	C4H9NO2	102.0559958	Yes
215	HMDB0000112; C00334; CHEBI:16865	Gamma-Aminobutyric acid	C4H9NO2	102.0559958	Yes
215	HMDB0000452; C02356; CHEBI:35619	L-Alpha-aminobutyric acid	C4H9NO2	102.0559958	Yes
215	HMDB0000650; C02261; CHEBI:28797	D-Alpha-aminobutyric acid	C4H9NO2	102.0559958	Yes
215	HMDB0001906; C03665; CHEBI:27971	2-Aminoisobutyric acid	C4H9NO2	102.0559958	Yes
215	HMDB0002166; C03284; CHEBI:33094	(S)-b-aminoisobutyric acid	C4H9NO2	102.0559958	Yes
215	HMDB0002299; C01205; CHEBI:16320	(R)-b-aminoisobutyric acid	C4H9NO2	102.0559958	Yes
215	HMDB0003911; C05145; CHEBI:27389	3-Aminoisobutanoic acid	C4H9NO2	102.0559958	Yes
215	HMDB0031329	Butyl nitrite	C4H9NO2	102.0559958	Yes
215	HMDB0031654; CHEBI:37081	3-Aminobutanoic acid	C4H9NO2	102.0559958	Yes
215	HMDB0038394	O-Acetylethanolamine	C4H9NO2	102.0559958	Yes
215	HMDB0041945; C11735; CHEBI:15620	N-Ethylglycine	C4H9NO2	102.0559958	Yes
215	HMDB0094692; CHEBI:17519	N-Methylalanine	C4H9NO2	102.0559958	Yes
222	HMDB0000691; C00383; CHEBI:30794	Malonic acid	C3H4O4	103.0036482	No
222	HMDB0001352; C00168; CHEBI:30841	Hydroxypyruvic acid	C3H4O4	103.0036482	No
222	HMDB0006938; C01146; CHEBI:16992	Tartronate semialdehyde	C3H4O4	103.0036482	No
226	HMDB0000008; C05984; CHEBI:1148	2-Hydroxybutyric acid	C4H8O3	103.0400162	Yes
226	HMDB0000011; C01089; CHEBI:17066	(R)-3-Hydroxybutyric acid	C4H8O3	103.0400162	Yes
226	HMDB0000023; C06001; CHEBI:37373	(S)-3-Hydroxyisobutyric acid	C4H8O3	103.0400162	Yes
226	HMDB0000336	(R)-3-Hydroxyisobutyric acid	C4H8O3	103.0400162	Yes
226	HMDB0000357; C01089; CHEBI:20067	3-Hydroxybutyric acid	C4H8O3	103.0400162	Yes
226	HMDB0000442; C03197; CHEBI:17290	(S)-3-Hydroxybutyric acid	C4H8O3	103.0400162	Yes
226	HMDB0000710; C00989; CHEBI:30830	4-Hydroxybutyric acid	C4H8O3	103.0400162	Yes
226	HMDB0000729; CHEBI:50129	Alpha-Hydroxyisobutyric acid	C4H8O3	103.0400162	Yes
226	HMDB0031212; CHEBI:741468	Ethoxyacetic acid	C4H8O3	103.0400162	Yes
226	HMDB0059748	2-Methyl-3-hydroxypropanoate	C4H8O3	103.0400162	Yes
226	HMDB0126088	3-hydroxy-2-methylpropanoic acid	C4H8O3	103.0400162	Yes
227	HMDB0002006; C03401; CHEBI:16303	2,3-Diaminopropionic acid	C3H8N2O2	103.0509394	No
228	HMDB0032457	1-Pentanethiol	C5H12S	103.0592049	No
228	HMDB0033902	Isopentyl mercaptan	C5H12S	103.0592049	No
228	HMDB0035418	2-Methyl-1-butanethiol	C5H12S	103.0592049	No
228	HMDB0036180	3-Methyl-2-butanethiol	C5H12S	103.0592049	No
228	HMDB0041010	(±)-2-Pentanethiol	C5H12S	103.0592049	No
232	HMDB0000187; C00065; CHEBI:17115	L-Serine	C3H7NO3	104.0352484	No
232	HMDB0003406; C00740; CHEBI:16523	D-Serine	C3H7NO3	104.0352484	No
232	HMDB0062263; C00716; CHEBI:17822	Protein serine	C3H7NO3	104.0352484	No
236	HMDB0031520; CHEBI:47872	2-Mercaptopropanoic acid	C3H6O2S	105.0014371	No

236	HMDB0041604; CHEBI:44111	3-Mercaptopropanoic acid	C3H6O2S	105.0014371	No
237	HMDB0000139; C00258; CHEBI:32398	Glyceric acid	C3H6O4	105.0191881	No
237	HMDB0006372; CHEBI:74324	L-Glyceric acid	C3H6O4	105.0191881	No
237	HMDB0031818; CHEBI:33508	D-2,3-Dihydroxypropanoic acid	C3H6O4	105.0191881	No
238	HMDB0006115; C00193; CHEBI:17169	Benzaldehyde	C7H6O	105.0346381	No
240	HMDB0034778; CHEBI:131388	(2R*,3R*)-1,2,3-Butanetriol	C4H10O3	105.055726	No
240	HMDB0061944	Trihydroxybutane	C4H10O3	105.055726	No
241	HMDB0059810; C07208; CHEBI:28488	m-Xylene	C8H10	105.0709884	No
241	HMDB0059851; C07212; CHEBI:28063	o-Xylene	C8H10	105.0709884	No
241	HMDB0059905; C07111; CHEBI:16101	Ethylbenzene	C8H10	105.0709884	No
241	HMDB0059924; C06756; CHEBI:27417	p-Xylene	C8H10	105.0709884	No
245	HMDB0029734; CHEBI:127904	3-Ethylpyridine	C7H9N	106.0663087	No
245	HMDB0032244	2,4-Dimethylpyridine	C7H9N	106.0663087	No
245	HMDB0032972; CHEBI:32548	2,6-Dimethylpyridine	C7H9N	106.0663087	No
245	HMDB0033871; C15562; CHEBI:40538	Benzylamine	C7H9N	106.0663087	No
245	HMDB0041965; C14403; CHEBI:66892	O-Toluidine	C7H9N	106.0663087	No
247	HMDB0003364; C00472; CHEBI:16509	Quinone	C6H4O2	107.0138181	No
247	HMDB0012133; C02351; CHEBI:17253	1,2-Benzoquinone	C6H4O2	107.0138181	No
250	HMDB0001858; C01468; CHEBI:17847	p-Cresol	C7H8O	107.0503114	Yes
250	HMDB0002048; C01467; CHEBI:17231	m-Cresol	C7H8O	107.0503114	Yes
250	HMDB0002055; C01542; CHEBI:28054	o-Cresol	C7H8O	107.0503114	Yes
250	HMDB0003119; C00556; CHEBI:17987	Benzyl alcohol	C7H8O	107.0503114	Yes
250	HMDB0033895; C01403; CHEBI:16579	Anisole	C7H8O	107.0503114	Yes
255	HMDB0001169; C02372; CHEBI:17602	4-Aminophenol	C6H7NO	108.0457737	Yes
255	HMDB0035882; CHEBI:59981	2-Acetylpyrrole	C6H7NO	108.0457737	Yes
255	HMDB0040582	1-Methyl-2-pyrrolecarboxaldehyde	C6H7NO	108.0457737	Yes
257	HMDB0029722	1,2,5-Trimethyl-1H-pyrrole	C7H11N	108.0819608	No
262	HMDB0029726	2-Vinylthiophene	C6H6S	109.0121418	No
262	HMDB0033746; CHEBI:48498	Benzenethiol	C6H6S	109.0121418	No
264	HMDB0000957; C00090; CHEBI:18135	Pyrocatechol	C6H6O2	109.0296735	Yes
264	HMDB0002434; C00530; CHEBI:17594	Hydroquinone	C6H6O2	109.0296735	Yes
264	HMDB0031180	(E,E)-2,4-Hexadienedial	C6H6O2	109.0296735	Yes
264	HMDB0032037; C01751; CHEBI:27810	1,3-Benzenediol	C6H6O2	109.0296735	Yes
264	HMDB0033002; C11115	5-Methyl-2-furancarboxaldehyde	C6H6O2	109.0296735	Yes
264	HMDB0033127; CHEBI:59983	2-Acetylfuran	C6H6O2	109.0296735	Yes
267	HMDB0029721	2,3,5-Trimethylfuran	C7H10O	109.0659084	No
267	HMDB0029728	2-Ethyl-5-methylfuran	C7H10O	109.0659084	No
267	HMDB0031541	3-Methyl-2-cyclohexen-1-one	C7H10O	109.0659084	No
267	HMDB0040277	2-Propylfuran	C7H10O	109.0659084	No
267	HMDB0040278	2-Isopropylfuran	C7H10O	109.0659084	No
267	HMDB0061891	(E,Z)-2,4-Heptadienal	C7H10O	109.0659084	No
271	HMDB0004230; C05942; CHEBI:36751	Pyrrole-2-carboxylic acid	C5H5NO2	110.0252333	Yes
271	HMDB0038175	2-Acetyloxazole	C5H5NO2	110.0252333	Yes
274	HMDB0031163	N-Acetyl-2,3-dihydro-1H-pyrrole	C6H9NO	110.0614554	No
274	HMDB0031308; CHEBI:67125	5-Acetyl-3,4-dihydro-2H-pyrrole	C6H9NO	110.0614554	No
274	HMDB0037837	2-Ethyl-4-methyloxazole	C6H9NO	110.0614554	No
274	HMDB0037838	4-Ethyl-2-methyloxazole	C6H9NO	110.0614554	No
274	HMDB0037858	2-Ethyl-5-methyloxazole	C6H9NO	110.0614554	No
274	HMDB0037859	5-Ethyl-2-methyloxazole	C6H9NO	110.0614554	No
274	HMDB0037863	5-Ethyl-4-methyloxazole	C6H9NO	110.0614554	No
274	HMDB0039584	5-Imino-2-methyl-1-cyclopenten-1-ol	C6H9NO	110.0614554	No
274	HMDB0040148	Trimethyloxazole	C6H9NO	110.0614554	No
275	HMDB0000870; C00388; CHEBI:18295	Histamine	C5H9N3	110.0722345	Yes
275	HMDB0014417; CHEBI:59170	Betazole	C5H9N3	110.0722345	Yes
275	HMDB0060263	Histamium	C5H9N3	110.0722345	Yes
280	HMDB0029717; CHEBI:246417	2-Thiophenecarboxaldehyde	C5H4OS	110.9910167	No
281	HMDB0000444; CHEBI:30846	3-Furoic acid	C5H4O3	111.0087886	Yes
281	HMDB0000617; C01546; CHEBI:30845	2-Furoic acid	C5H4O3	111.0087886	Yes
281	HMDB0032994	3-Hydroxy-2H-pyran-2-one	C5H4O3	111.0087886	Yes
281	HMDB0039437	2,3-Dihydroxy-2,4-cyclopentadien-1-one	C5H4O3	111.0087886	Yes
282	HMDB0000300; C00106; CHEBI:17568	Uracil	C4H4N2O2	111.0199841	Yes

282	HMDB0060760	4-Carboxypyrazole	C4H4N2O2	111.0199841	Yes
284	HMDB0001164; C04221; CHEBI:10702	trans-1,2-Dihydrobenzene-1,2-diol	C6H8O2	111.0453147	No
284	HMDB0029581; CHEBI:38358	(2E,4E)-2,4-Hexadienoic acid	C6H8O2	111.0453147	No
284	HMDB0031344; C06105; CHEBI:41674	1,2-Cyclohexanedione	C6H8O2	111.0453147	No
284	HMDB0031543	3-Methyl-1,2-cyclopentanedione	C6H8O2	111.0453147	No
284	HMDB0031759	(E)-4-Oxo-2-hexen-1-al	C6H8O2	111.0453147	No
284	HMDB0032233	2,5-Dimethyl-3(2H)-furanone	C6H8O2	111.0453147	No
284	HMDB0033569	Syoyualdehyde	C6H8O2	111.0453147	No
284	HMDB0034247	2-(Methoxymethyl)furan	C6H8O2	111.0453147	No
284	HMDB0039778	xi-3,5-Dimethyl-2(5H)-furanone	C6H8O2	111.0453147	No
284	HMDB0040260	5,5-Dimethyl-2(5H)-furanone	C6H8O2	111.0453147	No
287	HMDB0031483	(Z)-4-Heptenal	C7H12O	111.0815186	No
287	HMDB0031486	3-Hepten-2-one	C7H12O	111.0815186	No
287	HMDB0031487; CHEBI:87582	(E)-4-Hepten-2-one	C7H12O	111.0815186	No
287	HMDB0031488	2-Hepten-4-one	C7H12O	111.0815186	No
287	HMDB0031539	2-Methylcyclohexanone	C7H12O	111.0815186	No
287	HMDB0031540	4-Methylcyclohexanone	C7H12O	111.0815186	No
287	HMDB0031550	5-Methyl-3-hexen-2-one	C7H12O	111.0815186	No
287	HMDB0031551	5-Methyl-5-hexen-2-one	C7H12O	111.0815186	No
287	HMDB0032301	trans-2-trans-4-Heptadien-1-ol	C7H12O	111.0815186	No
287	HMDB0033667	(R)-3-Methylcyclohexanone	C7H12O	111.0815186	No
287	HMDB0033827	(2E)-2-Heptenal	C7H12O	111.0815186	No
287	HMDB0034372	xi-3-Methyl-3-cyclohexen-1-ol	C7H12O	111.0815186	No
287	HMDB0040339	2,5-Heptadien-1-ol	C7H12O	111.0815186	No
295	HMDB0032974	2,4-Dimethylthiazole	C5H7NS	112.0230472	No
295	HMDB0032975	2,5-Dimethylthiazole	C5H7NS	112.0230472	No
295	HMDB0032976	4,5-Dimethylthiazole	C5H7NS	112.0230472	No
295	HMDB0033867	4-Isothiocyanato-1-butene	C5H7NS	112.0230472	No
295	HMDB0038460	Thiiranepropanenitrile	C5H7NS	112.0230472	No
295	HMDB0040036	2-Ethylthiazole	C5H7NS	112.0230472	No
297	HMDB0001301; C04322; CHEBI:1372	1-Pyrroline-5-carboxylic acid	C5H7NO2	112.0392746	No
297	HMDB0006875; C03564; CHEBI:36761	1-Pyrroline-2-carboxylic acid	C5H7NO2	112.0392746	No
298	HMDB0000562; C00791; CHEBI:16737	Creatinine	C4H7N3O	112.0515817	No
303	HMDB0060293; CHEBI:5587	Hydroxidodioxidosulfidosulfate	H2O3S2	112.9373088	No
307	HMDB0032915	2-Furanmethanethiol	C5H6OS	113.0064262	No
307	HMDB0032993	2-Thiophenemethanol	C5H6OS	113.0064262	No
307	HMDB0036611	2-Methyl-3-furanthiol	C5H6OS	113.0064262	No
307	HMDB0039798	5-Methyl-3-furanthiol	C5H6OS	113.0064262	No
307	HMDB0041504	3-Thiophenemethanol	C5H6OS	113.0064262	No
309	HMDB0031859; CHEBI:74456	Norfuraneol	C5H6O3	113.0244052	Yes
309	HMDB0060461; CHEBI:28993	cis-Acetylacrylate	C5H6O3	113.0244052	Yes
310	HMDB0000076; C00429; CHEBI:15901	Dihydrouracil	C4H6N2O2	113.0355858	Yes
310	HMDB0003646; C02565; CHEBI:16354	N-Methylhydantoin	C4H6N2O2	113.0355858	Yes
310	HMDB0060245; C02512; CHEBI:16934	L-3-Cyanoalanine	C4H6N2O2	113.0355858	Yes
313	HMDB0003671; C08380; CHEBI:5672	2-Heptanone	C7H14O	113.097112	No
313	HMDB0004814	4-Heptanone	C7H14O	113.097112	No
313	HMDB0005841	Ethyl isobutyl ketone	C7H14O	113.097112	No
313	HMDB0031405	(Z)-4-Hepten-1-ol	C7H14O	113.097112	No
313	HMDB0031429; CHEBI:87754	2,4-Dimethyl-3-pentanone	C7H14O	113.097112	No
313	HMDB0031475; C14390	Heptanal	C7H14O	113.097112	No
313	HMDB0031482; CHEBI:50139	3-Heptanone	C7H14O	113.097112	No
313	HMDB0031538	3-Methylcyclohexanol	C7H14O	113.097112	No
313	HMDB0031549	5-Methyl-2-hexanone	C7H14O	113.097112	No
313	HMDB0032405; CHEBI:88822	3-Methylhexanal	C7H14O	113.097112	No
313	HMDB0038052	1-Ethoxy-3-methyl-2-butene	C7H14O	113.097112	No
313	HMDB0040191	(±)-2-Methylhexanal	C7H14O	113.097112	No
313	HMDB0041320	(2R,4Z)-4-Hepten-2-ol	C7H14O	113.097112	No
313	HMDB0059693	1-Methylcyclohexanol	C7H14O	113.097112	No
313	HMDB0061896	2-Methyl-3-hexanone	C7H14O	113.097112	No
322	HMDB0029172; CHEBI:19680	3-keto-2-Methylbutyrate	C5H7O3	114.0348459	No
323	HMDB0031328; CHEBI:50534	1-Isothiocyanatobutane	C5H9NS	114.0389472	No

323	HMDB0032345	Isobutyl isothiocyanate	C5H9NS	114.0389472	No
323	HMDB0040331	xi-2,5-Dihydro-2,4-dimethylthiazole	C5H9NS	114.0389472	No
324	HMDB0000162; C00148; CHEBI:17203	L-Proline	C5H9NO2	114.0559188	No
324	HMDB0003411; C00763; CHEBI:16313	D-Proline	C5H9NO2	114.0559188	No
324	HMDB0012880; C18170; CHEBI:30322	Acetamidopropanal	C5H9NO2	114.0559188	No
324	HMDB0030409	4-Amino-2-methylenebutanoic acid	C5H9NO2	114.0559188	No
324	HMDB0034208	Pterolactam	C5H9NO2	114.0559188	No
325	HMDB0031651	N-(3-Methylbutyl)acetamide	C6H13NO	114.0924738	No
325	HMDB0034203	N-(2-Methylpropyl)acetamide	C6H13NO	114.0924738	No
330	HMDB0000134; C00122; CHEBI:18012	Fumaric acid	C4H4O4	115.0037642	Yes
330	HMDB0000176; C01384; CHEBI:18300	Maleic acid	C4H4O4	115.0037642	Yes
333	HMDB0000019; C00141; CHEBI:16530	Alpha-ketoisovaleric acid	C5H8O3	115.0401488	Yes
333	HMDB0000310	Methylacetoacetic acid	C5H8O3	115.0401488	Yes
333	HMDB0000720; CHEBI:45630	Levulinic acid	C5H8O3	115.0401488	Yes
333	HMDB0001865; C06255; CHEBI:33033	2-Oxovaleric acid	C5H8O3	115.0401488	Yes
333	HMDB0003771; CHEBI:37079	2-Methylacetoacetic acid	C5H8O3	115.0401488	Yes
333	HMDB0012233; C03273; CHEBI:39153	Glutarate semialdehyde	C5H8O3	115.0401488	Yes
333	HMDB0031643	Ethyl pyruvate	C5H8O3	115.0401488	Yes
333	HMDB0034466	Acetoxyacetone	C5H8O3	115.0401488	Yes
333	HMDB0128916	4-hydroxy-3-methylbut-2-enoic acid	C5H8O3	115.0401488	Yes
333	HMDB0128917	dimethyloxirane-2-carboxylic acid	C5H8O3	115.0401488	Yes
333	HMDB0128943	(2E)-4-hydroxy-3-methylbut-2-enoic acid	C5H8O3	115.0401488	Yes
333	HMDB0129096	(2Z)-2-(hydroxymethyl)but-2-enoic acid	C5H8O3	115.0401488	Yes
333	HMDB0129097	4-hydroxy-2-methylbut-2-enoic acid	C5H8O3	115.0401488	Yes
333	HMDB0129098	2,3-dimethyloxirane-2-carboxylic acid	C5H8O3	115.0401488	Yes
333	HMDB0132477	2-(hydroxymethyl)but-2-enoic acid	C5H8O3	115.0401488	Yes
335	HMDB0000535; C01585; CHEBI:30776	Caproic acid	C6H12O2	115.0763976	No
335	HMDB0000689; CHEBI:74903	Isocaproic acid	C6H12O2	115.0763976	No
335	HMDB0029762	Methyl (S)-2-Methylbutanoate	C6H12O2	115.0763976	No
335	HMDB0030027	Methyl 3-methylbutanoate	C6H12O2	115.0763976	No
335	HMDB0030056	Isopropyl propionate	C6H12O2	115.0763976	No
335	HMDB0030059	Propyl propionate	C6H12O2	115.0763976	No
335	HMDB0031207	Methyl pentanoate	C6H12O2	115.0763976	No
335	HMDB0031221; CHEBI:410868	2-Ethylbutanoic acid	C6H12O2	115.0763976	No
335	HMDB0031246; CHEBI:50569	2-Methylpropyl acetate	C6H12O2	115.0763976	No
335	HMDB0031248; CHEBI:167422	Ethyl 2-methylpropanoate	C6H12O2	115.0763976	No
335	HMDB0031325; C12304; CHEBI:31328	n-Butyl acetate	C6H12O2	115.0763976	No
335	HMDB0031511; CHEBI:55381	Diacetone alcohol	C6H12O2	115.0763976	No
335	HMDB0031580	(±)-2-Methylpentanoic acid	C6H12O2	115.0763976	No
335	HMDB0033774	3-Methylpentanoic acid	C6H12O2	115.0763976	No
335	HMDB0033889	Ethyl butyrate	C6H12O2	115.0763976	No
335	HMDB0034163; C12293; CHEBI:31726	3-Methylbutyl formate	C6H12O2	115.0763976	No
335	HMDB0037266	2,2,4-Trimethyl-1,3-dioxolane	C6H12O2	115.0763976	No
335	HMDB0040279; CHEBI:87572	2,4,5-Trimethyl-1,3-dioxolane	C6H12O2	115.0763976	No
335	HMDB0040327	Pentyl formate	C6H12O2	115.0763976	No
341	HMDB0002287	Homocysteine thiolactone	C4H7NOS	116.0174696	No
342	HMDB0000532; CHEBI:40410	Acetylglycine	C4H7NO3	116.0352377	No
342	HMDB0006454; C03508; CHEBI:40673	L-2-Amino-3-oxobutanoic acid	C4H7NO3	116.0352377	No
342	HMDB0012249; C00441; CHEBI:18051	L-Aspartate-semialdehyde	C4H7NO3	116.0352377	No
343	HMDB0000738; C00463; CHEBI:16881	Indole	C8H7N	116.0505866	Yes
343	HMDB0034171; C16074; CHEBI:25979	Benzeneacetonitrile	C8H7N	116.0505866	Yes
344	HMDB0000043; C00719; CHEBI:17750	Betaine	C5H11NO2	116.0715056	Yes
344	HMDB0000883; C00183; CHEBI:16414	L-Valine	C5H11NO2	116.0715056	Yes
344	HMDB0001382; C07457; CHEBI:2691	Vaporole	C5H11NO2	116.0715056	Yes
344	HMDB0002141; CHEBI:134261	N-Methyl-a-aminoisobutyric acid	C5H11NO2	116.0715056	Yes
344	HMDB0003355; C00431; CHEBI:15887	5-Aminopentanoic acid	C5H11NO2	116.0715056	Yes
344	HMDB0013716; C01799; CHEBI:28804	Norvaline	C5H11NO2	116.0715056	Yes
344	HMDB0015550; C07457; CHEBI:55344	Amyl Nitrite	C5H11NO2	116.0715056	Yes
348	HMDB0039786	Dihydro-4-mercapto-3(2H)-furanone	C4H6O2S	117.0014062	No
350	HMDB0000202; C02170; CHEBI:30860	Methylmalonic acid	C4H6O4	117.0194662	No
350	HMDB0000254; C00042; CHEBI:15741	Succinic acid	C4H6O4	117.0194662	No

350	HMDB0000349; CHEBI:290972	Erythrono-1,4-lactone	C4H6O4	117.0194662	No
350	HMDB0000940; CHEBI:71176	Threonolactone	C4H6O4	117.0194662	No
350	HMDB0031204	4-Hydroxy-2-oxobutanoic acid	C4H6O4	117.0194662	No
350	HMDB0039324	xi-3-Hydroxy-2-oxobutanoic acid	C4H6O4	117.0194662	No
350	HMDB0130020	3-methoxy-3-oxopropanoic acid	C4H6O4	117.0194662	No
351	HMDB0030968	2,4,6-Octatriyn-1-ol	C8H6O	117.0341437	No
351	HMDB0032929; C14512; CHEBI:35260	Benzofuran	C8H6O	117.0341437	No
351	HMDB0038998	3,5,7-Octatriyn-1-ol	C8H6O	117.0341437	No
351	HMDB0040549	xi-2,3-Octadiene-5,7-diyn-1-ol	C8H6O	117.0341437	No
352	HMDB0033576	7-Azaindolizine	C7H6N2	117.0458628	No
352	HMDB0040040	5H-Cyclopentapyrazine	C7H6N2	117.0458628	No
353	HMDB0000351	3-Hydroxy-2-methyl-[R-(R,S)]-butanoic acid	C5H10O3	117.0556028	Yes
353	HMDB0000354; CHEBI:37051	2-Methyl-3-hydroxybutyric acid	C5H10O3	117.0556028	Yes
353	HMDB0000396; CHEBI:741550	2-Ethylhydracrylic acid	C5H10O3	117.0556028	Yes
353	HMDB0000407; CHEBI:60645	2-Hydroxy-3-methylbutyric acid	C5H10O3	117.0556028	Yes
353	HMDB0000410	3-Hydroxy-2-methyl-[S-(R,R)]-butanoic acid	C5H10O3	117.0556028	Yes
353	HMDB0000531	3-Hydroxyvaleric acid	C5H10O3	117.0556028	Yes
353	HMDB0000642	Erythronilic acid	C5H10O3	117.0556028	Yes
353	HMDB0000754; CHEBI:37084	3-Hydroxyisovaleric acid	C5H10O3	117.0556028	Yes
353	HMDB0001863; CHEBI:60647	2-Hydroxyvaleric acid	C5H10O3	117.0556028	Yes
353	HMDB0001987; CHEBI:68454	2-Hydroxy-2-methylbutyric acid	C5H10O3	117.0556028	Yes
353	HMDB0002011; CHEBI:384411	4-Hydroxyisovaleric acid	C5H10O3	117.0556028	Yes
353	HMDB0040735; CHEBI:78321	Ethyl lactate	C5H10O3	117.0556028	Yes
353	HMDB0041603	Methyl 3-hydroxybutyrate	C5H10O3	117.0556028	Yes
353	HMDB0059844	Diethyl carbonate	C5H10O3	117.0556028	Yes
353	HMDB0061927; C02804; CHEBI:45564	5-Hydroxypentanoic acid	C5H10O3	117.0556028	Yes
353	HMDB0062584; CHEBI:60631	(S)-2-hydroxy-3-methylbutyric Acid	C5H10O3	117.0556028	Yes
353	HMDB0130836	4-hydroxy-2-methylbutanoic acid	C5H10O3	117.0556028	Yes
354	HMDB0059837; CHEBI:37911	Indane	C9H10	117.0710574	No
354	HMDB0059899; C14395	alpha-Methylstyrene	C9H10	117.0710574	No
358	HMDB0001147; C00872; CHEBI:17475	Aminomalonic acid	C3H5NO4	118.0141636	No
358	HMDB0034259; C05669; CHEBI:16348	Bovinocidin	C3H5NO4	118.0141636	No
360	HMDB0062270; CHEBI:53806	1,2-Diacylglycerol-LD-PI-pool	C7H5NO	118.0299475	Yes
362	HMDB0000167; C00188; CHEBI:16857	L-Threonine	C4H9NO3	118.0509218	Yes
362	HMDB0000719; C00263; CHEBI:15699	L-Homoserine	C4H9NO3	118.0509218	Yes
362	HMDB0004041; C05519; CHEBI:28718	L-Allothreonine	C4H9NO3	118.0509218	Yes
362	HMDB0061148	Hydroxyethyl glycine	C4H9NO3	118.0509218	Yes
362	HMDB0061877; CHEBI:16080	4-Amino-3-hydroxybutyrate	C4H9NO3	118.0509218	Yes
368	HMDB0001368; C00957; CHEBI:16208	3-Mercaptopyruvic acid	C3H4O3S	118.9805911	No
369	HMDB0001527; C08276; CHEBI:1438	3-Methylthiopropionic acid	C4H8O2S	119.0170694	No
369	HMDB0035646; C03173; CHEBI:47870	Methyl 2-(methylthio)acetate	C4H8O2S	119.0170694	No
369	HMDB0112255	Allyl methyl sulfone	C4H8O2S	119.0170694	No
371	HMDB0000337; CHEBI:86371	(S)-3,4-Dihydroxybutyric acid	C4H8O4	119.0348558	Yes
371	HMDB0000360; CHEBI:86348	2,4-Dihydroxybutanoic acid	C4H8O4	119.0348558	Yes
371	HMDB0000498	4-Deoxyerythronic acid	C4H8O4	119.0348558	Yes
371	HMDB0002453; CHEBI:86391	4-Deoxythreonic acid	C4H8O4	119.0348558	Yes
371	HMDB0002601; CHEBI:36532	A,b-Dihydroxyisobutyric acid	C4H8O4	119.0348558	Yes
371	HMDB0002649; C01796; CHEBI:23956	Erythrose	C4H8O4	119.0348558	Yes
371	HMDB0006293; C02045; CHEBI:27913	L-Erythrulose	C4H8O4	119.0348558	Yes
372	HMDB0004072; C05627; CHEBI:1883	4-Hydroxystyrene	C8H8O	119.0499415	Yes
372	HMDB0006236; C00601; CHEBI:16424	Phenylacetaldehyde	C8H8O	119.0499415	Yes
372	HMDB0013815; CHEBI:87607	2,3-Dihydrobenzofuran	C8H8O	119.0499415	Yes
372	HMDB0029636; C07214; CHEBI:27434	2-Methylbenzaldehyde	C8H8O	119.0499415	Yes
372	HMDB0029637; C07209; CHEBI:28476	3-Methylbenzaldehyde	C8H8O	119.0499415	Yes
372	HMDB0029638; C06758; CHEBI:28617	4-Methylbenzaldehyde	C8H8O	119.0499415	Yes
372	HMDB0033910; C07113; CHEBI:27632	Acetophenone	C8H8O	119.0499415	Yes
372	HMDB0038997	Lentialexin	C8H8O	119.0499415	Yes
372	HMDB0062765; C02083; CHEBI:17907	Styrene Oxide	C8H8O	119.0499415	Yes
372	HMDB0124943	3-ethenylphenol	C8H8O	119.0499415	Yes
373	HMDB0013733; C14533; CHEBI:34039	1,2,4-Trimethylbenzene	C9H12	119.0867278	No
373	HMDB0034029; C14396; CHEBI:34656	Isopropylbenzene	C9H12	119.0867278	No

373	HMDB0040458	1,2,4-Tris(methylene)cyclohexane	C9H12	119.0867278	No
373	HMDB0041924; C14508; CHEBI:34833	Mesitylene	C9H12	119.0867278	No
373	HMDB0059819; C14572; CHEBI:34276	o-Ethyltoluene	C9H12	119.0867278	No
373	HMDB0059848; C14522; CHEBI:77512	m-Ethyl_toluene	C9H12	119.0867278	No
373	HMDB0059877; CHEBI:42630	Propylbenzene	C9H12	119.0867278	No
373	HMDB0059901; C14518; CHEBI:34037	Hemimellitene	C9H12	119.0867278	No
381	HMDB0000574; C00097; CHEBI:17561	L-Cysteine	C3H7NO2S	120.0124373	No
381	HMDB0003417; C00793; CHEBI:16375	D-Cysteine	C3H7NO2S	120.0124373	No
385	HMDB0004461; C09815; CHEBI:28179	Benzamide	C7H7NO	120.0458101	Yes
385	HMDB0033131	3-Acetylpyridine	C7H7NO	120.0458101	Yes
385	HMDB0035281	2-Acetylpyridine	C7H7NO	120.0458101	Yes
387	HMDB0001020; C02846; CHEBI:16269	N,N-Dimethylaniline	C8H11N	120.0821703	No
387	HMDB0002017; C02455; CHEBI:670	1-Phenylethylamine	C8H11N	120.0821703	No
387	HMDB0012275; C05332; CHEBI:18397	Phenylethylamine	C8H11N	120.0821703	No
387	HMDB0029729; CHEBI:481701	5-Ethyl-2-methylpyridine	C8H11N	120.0821703	No
387	HMDB0032497	2-Propylpyridine	C8H11N	120.0821703	No
387	HMDB0040041	2-Ethyl-5-methylpyridine	C8H11N	120.0821703	No
387	HMDB0060276	2-Phenylethanaminium	C8H11N	120.0821703	No
387	HMDB0060677; C11004; CHEBI:28738	2,6-Dimethylaniline	C8H11N	120.0821703	No
394	HMDB0002127; C05823	3-Mercaptolactic acid	C3H6O3S	120.996193	No
397	HMDB0001870; C00180; CHEBI:30746	Benzoic acid	C7H6O2	121.0291257	Yes
397	HMDB0011718; C00633; CHEBI:17597	4-Hydroxybenzaldehyde	C7H6O2	121.0291257	Yes
397	HMDB0032918	3-(2-Furanyl)-2-propenal	C7H6O2	121.0291257	Yes
397	HMDB0034170; C06202; CHEBI:16008	2-Hydroxybenzaldehyde	C7H6O2	121.0291257	Yes
398	HMDB0001406; C00153; CHEBI:17154	Niacinamide	C6H6N2O	121.0407299	No
398	HMDB0031861	2-Acetylpyrazine	C6H6N2O	121.0407299	No
399	HMDB0002994; C00503; CHEBI:17113	Erythritol	C4H10O4	121.0517622	No
399	HMDB0004136; C16884; CHEBI:48300	D-Threitol	C4H10O4	121.0517622	No
399	HMDB0094689; CHEBI:48299	1,2,3,4-Tetrahydroxybutane	C4H10O4	121.0517622	No
400	HMDB0029306; C13637; CHEBI:49584	4-Ethylphenol	C8H10O	121.0660887	No
400	HMDB0030540; CHEBI:421375	2,5-Dimethylphenol	C8H10O	121.0660887	No
400	HMDB0032074	1-Methoxy-2-methylbenzene	C8H10O	121.0660887	No
400	HMDB0032076	1-Methoxy-4-methylbenzene	C8H10O	121.0660887	No
400	HMDB0032148	2,3-Dimethylphenol	C8H10O	121.0660887	No
400	HMDB0032150	2,6-Dimethylphenol	C8H10O	121.0660887	No
400	HMDB0032151; CHEBI:39839	3,4-Dimethylphenol	C8H10O	121.0660887	No
400	HMDB0032619; C07112; CHEBI:669	1-Phenylethanol	C8H10O	121.0660887	No
400	HMDB0033944; C05853; CHEBI:49000	2-Phenylethanol	C8H10O	121.0660887	No
400	HMDB0041609; C06757; CHEBI:1895	4-Methylbenzyl alcohol	C8H10O	121.0660887	No
400	HMDB0059873; C14386	3-Ethylphenol	C8H10O	121.0660887	No
410	HMDB0001488; C00253; CHEBI:15940	Nicotinic acid	C6H5NO2	122.0250649	Yes
410	HMDB0002243; C10164; CHEBI:28747	Picolinic acid	C6H5NO2	122.0250649	Yes
410	HMDB0031713	2-Hydroxy-4-imino-2,5-cyclohexadienone	C6H5NO2	122.0250649	Yes
410	HMDB0041950; C06813; CHEBI:27798	Nitrobenzene	C6H5NO2	122.0250649	Yes
410	HMDB0060665; C07446; CHEBI:6032	Isonicotinic acid	C6H5NO2	122.0250649	Yes
413	HMDB0029300; C19326; CHEBI:163384	p-Anisidine	C7H9NO	122.061245	Yes
413	HMDB0032492	2-Propionylpyrrole	C7H9NO	122.061245	Yes
413	HMDB0032578	4-Hydroxybenzylamine	C7H9NO	122.061245	Yes
413	HMDB0036058	1-Ethyl-1H-pyrrole-2-carboxaldehyde	C7H9NO	122.061245	Yes
413	HMDB0040360; CHEBI:59982	2-Acetyl-1-methylpyrrole	C7H9NO	122.061245	Yes
418	HMDB0000873; C06730; CHEBI:17254	4-Methylcatechol	C7H8O2	123.0458593	No
418	HMDB0001398; C01502; CHEBI:28591	Guaiacol	C7H8O2	123.0458593	No
418	HMDB0011724; C17467; CHEBI:437856	4-Hydroxybenzyl alcohol	C7H8O2	123.0458593	No
418	HMDB0029696	Mequinol	C7H8O2	123.0458593	No
418	HMDB0032917	1-(2-Furanyl)-2-propanone	C7H8O2	123.0458593	No
418	HMDB0034877	2-Acetyl-3-methylfuran	C7H8O2	123.0458593	No
418	HMDB0035226; CHEBI:562752	2-Acetyl-5-methylfuran	C7H8O2	123.0458593	No
418	HMDB0038963	4,6-Heptadiyne-1,3-diol	C7H8O2	123.0458593	No
418	HMDB0040280	1-(2-Furanyl)-1-propanone	C7H8O2	123.0458593	No
418	HMDB0059709; C02323; CHEBI:16464	2-Hydroxybenzyl alcohol	C7H8O2	123.0458593	No
418	HMDB0059712; C03351; CHEBI:17069	3-Hydroxybenzyl alcohol	C7H8O2	123.0458593	No

418	HMDB0135619	3-methoxyphenol	C7H8O2	123.0458593	No
419	HMDB0004181; C05827; CHEBI:28104	Methylimidazole acetaldehyde	C6H8N2O	123.0565686	Yes
419	HMDB0031852	2-Methoxy-3-methylpyrazine	C6H8N2O	123.0565686	Yes
419	HMDB0040141	2-Methoxy-5-methylpyrazine	C6H8N2O	123.0565686	Yes
419	HMDB0040143	2-Methoxy-6-methylpyrazine	C6H8N2O	123.0565686	Yes
423	HMDB0000251; C00245; CHEBI:15891	Taurine	C2H7NO3S	124.0075296	No
424	HMDB0011747; C03557; CHEBI:15573	Ciliatine	C2H8NO3P	124.0163938	Yes
429	HMDB0030345; CHEBI:59533	6-Acetyl-1,2,3,4-tetrahydropyridine	C7H11NO	124.0758716	No
429	HMDB0034883	3,4-Dihydro-5-propanoyl-2H-pyrrole	C7H11NO	124.0758716	No
429	HMDB0037853	2-Methyl-4-propyloxazole	C7H11NO	124.0758716	No
429	HMDB0037854	4-Methyl-2-propyloxazole	C7H11NO	124.0758716	No
429	HMDB0037860	2-Methyl-5-propyloxazole	C7H11NO	124.0758716	No
429	HMDB0037861	5-Methyl-2-propyloxazole	C7H11NO	124.0758716	No
429	HMDB0037865	2-Ethyl-4,5-dimethyloxazole	C7H11NO	124.0758716	No
429	HMDB0037866	4-Ethyl-2,5-dimethyloxazole	C7H11NO	124.0758716	No
429	HMDB0037867	5-Ethyl-2,4-dimethyloxazole	C7H11NO	124.0758716	No
429	HMDB0038288	4-Butyloxazole	C7H11NO	124.0758716	No
429	HMDB0038289	5-Butyloxazole	C7H11NO	124.0758716	No
429	HMDB0039583	2-Ethyl-5-imino-1-cyclopenten-1-ol	C7H11NO	124.0758716	No
431	HMDB0013780; C08372	Dimethyl trisulfide	C2H6S3	124.9545292	No
433	HMDB0003903; C05123; CHEBI:1157	2-Hydroxyethanesulfonate	C2H6O4S	124.9914303	Yes
433	HMDB0031233	Ethyl hydrogen sulfate	C2H6O4S	124.9914303	Yes
434	HMDB0013674; C01108; CHEBI:16164	1,2,3-Trihydroxybenzene	C6H6O3	125.0244561	Yes
434	HMDB0013675; C02183; CHEBI:16204	1,3,5-Trihydroxybenzene	C6H6O3	125.0244561	Yes
434	HMDB0029750	Methyl 2-furoate	C6H6O3	125.0244561	Yes
434	HMDB0030776; C11918; CHEBI:142577	Maltol	C6H6O3	125.0244561	Yes
434	HMDB0031256	4-Hydroxy-2H-pyran-3-carboxaldehyde	C6H6O3	125.0244561	Yes
434	HMDB0032651	(Z)-Tamarindienal	C6H6O3	125.0244561	Yes
434	HMDB0034355; C11101; CHEBI:412516	5-Hydroxymethyl-2-furancarboxaldehyde	C6H6O3	125.0244561	Yes
434	HMDB0059735	5-Methylfuran-2-carboxylic acid	C6H6O3	125.0244561	Yes
434	HMDB0124831	benzene-1,2,4-triol	C6H6O3	125.0244561	Yes
436	HMDB0002128; CHEBI:804916	2,4-Diamino-6-hydroxypyrimidine	C4H6N4O	125.0470921	No
436	HMDB0003192; C04051; CHEBI:2030	5-Aminoimidazole-4-carboxamide	C4H6N4O	125.0470921	No
437	HMDB0029582; CHEBI:515741	Methyl sorbate	C7H10O2	125.0600368	No
437	HMDB0031222	3-Ethyl-1,2-cyclopentanedione	C7H10O2	125.0600368	No
437	HMDB0031223	Enol-3-Ethyl-1,2-cyclopentanedione	C7H10O2	125.0600368	No
437	HMDB0032067	Allyl crotonate	C7H10O2	125.0600368	No
437	HMDB0032333; CHEBI:52939	4-Hydroxy-4-methyl-5-hexenoic acid gamma lactone	C7H10O2	125.0600368	No
437	HMDB0033629	xi-4-Hydroxy-4-methyl-2-cyclohexen-1-one	C7H10O2	125.0600368	No
437	HMDB0034080	3-Methyl-1,2-cyclohexanedione	C7H10O2	125.0600368	No
437	HMDB0034835	3-(3-Ethyloxiranyl)-2-propenal	C7H10O2	125.0600368	No
437	HMDB0037026	3,4-Dimethyl-1,2-cyclopentanedione	C7H10O2	125.0600368	No
437	HMDB0037027	3,5-Dimethyl-1,2-cyclopentanedione	C7H10O2	125.0600368	No
437	HMDB0040445	2-(Ethoxymethyl)furan	C7H10O2	125.0600368	No
437	HMDB0041474	xi-5-Isopropyl-2(5H)-furanone	C7H10O2	125.0600368	No
440	HMDB0013809; CHEBI:61748	(E)-2-octenal	C8H14O	125.0971931	No
440	HMDB0030961	2-Octenal	C8H14O	125.0971931	No
440	HMDB0030966	(3xi,5Z)-1,5-Octadien-3-ol	C8H14O	125.0971931	No
440	HMDB0031197	2,4,4-Trimethylcyclopentanone	C8H14O	125.0971931	No
440	HMDB0031301	2-Octen-4-one	C8H14O	125.0971931	No
440	HMDB0031309	1-Octen-3-one	C8H14O	125.0971931	No
440	HMDB0031590	3-Methyl-3-hepten-2-one	C8H14O	125.0971931	No
440	HMDB0031591	5-Methyl-5-hepten-2-one	C8H14O	125.0971931	No
440	HMDB0032403	6-Methyl-3-hepten-2-one, trans-	C8H14O	125.0971931	No
440	HMDB0032451	4-Octen-3-one	C8H14O	125.0971931	No
440	HMDB0033547	3-Octen-2-one	C8H14O	125.0971931	No
440	HMDB0035242	(S,E)-Filbertone	C8H14O	125.0971931	No
440	HMDB0035390	5-Octen-2-one	C8H14O	125.0971931	No
440	HMDB0035915; C07287; CHEBI:16310	Sulcatone	C8H14O	125.0971931	No
440	HMDB0037152	2-Ethylidenehexanal	C8H14O	125.0971931	No
440	HMDB0039769	6-Octenal	C8H14O	125.0971931	No

440	HMDB0039770	(Z)-5-Octenal	C8H14O	125.0971931	No
440	HMDB0040146	2,5-Octadien-1-ol	C8H14O	125.0971931	No
440	HMDB0040149	(3E,5Z)-3,5-Octadien-1-ol	C8H14O	125.0971931	No
440	HMDB0040151	(2E,4E)-2,4-Octadien-1-ol	C8H14O	125.0971931	No
440	HMDB0061945; CHEBI:88838	2-Ethyl-2-hexenal	C8H14O	125.0971931	No
440	HMDB0062471	glutathionyl-3-hydroxykynurenine glucoside	C8H14O	125.0971931	No
440	HMDB0062529	Sialyl-3-paragloboside	C8H14O	125.0971931	No
440	HMDB0062536	trans-3-cis-8,11,14-eicosatetraenoyl-CoA	C8H14O	125.0971931	No
443	HMDB0034368	Hydroxyminaline	C5H5NO3	126.0198204	Yes
444	HMDB0060659; C16621	1-Methyl-4-nitroimidazole	C4H5N3O2	126.0308194	Yes
445	HMDB0001084; C04092; CHEBI:30912	D-1-Piperideine-2-carboxylic acid	C6H9NO2	126.0560214	No
445	HMDB0012130; CHEBI:49015	(S)-2,3,4,5-Tetrahydropiperidine-2-carboxylate	C6H9NO2	126.0560214	No
445	HMDB0029434	(±)-4-Methylene-2-pyrrolidinecarboxylic acid	C6H9NO2	126.0560214	No
445	HMDB0059657; C00450; CHEBI:49014	(S)-2,3,4,5-tetrahydropyridine-2-carboxylate	C6H9NO2	126.0560214	No
447	HMDB0031667	Nor-psi-tropine	C7H13NO	126.092474	No
447	HMDB0040030	1-(1-Pyrrolidinyl)-2-propanone	C7H13NO	126.092474	No
454	HMDB0059784; CHEBI:86395	5-Hydroxy-2-furoic acid	C5H4O4	127.0040153	No
455	HMDB0041833; C00813; CHEBI:16294	Barbituric acid	C4H4N2O3	127.0146309	No
456	HMDB0032916	2-(Methylthiomethyl)furan	C6H8OS	127.0228171	No
456	HMDB0036610	2-Methyl-3-(methylthio)furan	C6H8OS	127.0228171	No
456	HMDB0039586	2-(1-Mercaptoethyl)furan	C6H8OS	127.0228171	No
456	HMDB0039800	5-Methyl-2-furanmethanethiol	C6H8OS	127.0228171	No
456	HMDB0040197	2,5-Dimethyl-3-furanthiol	C6H8OS	127.0228171	No
456	HMDB0041566	2-Methyl-5-(methylthio)furan	C6H8OS	127.0228171	No
458	HMDB0031303	Osmundalactone	C6H8O3	127.0401707	Yes
458	HMDB0031306	3-Hydroxy-4,5-dimethyl-2(5H)-furanone	C6H8O3	127.0401707	Yes
458	HMDB0031847	2-Ethyl-3,4-dihydroxyfuran	C6H8O3	127.0401707	Yes
458	HMDB0033285	L-erythro-5-(1-Hydroxyethyl)-2(5H)-furanone	C6H8O3	127.0401707	Yes
458	HMDB0033517	5,6-Dihydro-4-methoxy-2H-pyran-2-one	C6H8O3	127.0401707	Yes
458	HMDB0038072	xi-5-Acetyltetrahydro-2(3H)-furanone	C6H8O3	127.0401707	Yes
458	HMDB0040594; CHEBI:76247	(±)-Furaneol	C6H8O3	127.0401707	Yes
458	HMDB0040732	2,7-Oxepanedione	C6H8O3	127.0401707	Yes
459	HMDB0000079; C00906; CHEBI:27468	Dihydrothymine	C5H8N2O2	127.0512895	Yes
459	HMDB0029874; CHEBI:565546	Squamolone	C5H8N2O2	127.0512895	Yes
459	HMDB0031547	L-Cyclo(alanylglycyl)	C5H8N2O2	127.0512895	Yes
459	HMDB0062558; CHEBI:133469	Pyroglutamine	C5H8N2O2	127.0512895	Yes
460	HMDB0029165	1,3-Diacetylpropane	C7H12O2	127.0764893	Yes
460	HMDB0029314	Isopentenyl acetate	C7H12O2	127.0764893	Yes
460	HMDB0030330	1-Methoxy-3-methylene-2-pentanone	C7H12O2	127.0764893	Yes
460	HMDB0031174	4-Methyl-5-hexanolide	C7H12O2	127.0764893	Yes
460	HMDB0031342; C09822; CHEBI:36096	Cyclohexanecarboxylic acid	C7H12O2	127.0764893	Yes
460	HMDB0031351	Cyclohexyl formate	C7H12O2	127.0764893	Yes
460	HMDB0031476	2,3-Heptanedione	C7H12O2	127.0764893	Yes
460	HMDB0031477	3,4-Heptanedione	C7H12O2	127.0764893	Yes
460	HMDB0031484; CHEBI:38364	2-Heptenoic acid	C7H12O2	127.0764893	Yes
460	HMDB0031500	Methyl 2E-hexenoate	C7H12O2	127.0764893	Yes
460	HMDB0031504	Methyl (Z)-3-hexenoate	C7H12O2	127.0764893	Yes
460	HMDB0031593	5-Methyl-2,3-hexanedione	C7H12O2	127.0764893	Yes
460	HMDB0031603	Ethyl 4-pentenoate	C7H12O2	127.0764893	Yes
460	HMDB0031681	Dihydro-5-propyl-2(3H)-furanone	C7H12O2	127.0764893	Yes
460	HMDB0032391	3-Methyl-3-butenyl acetate	C7H12O2	127.0764893	Yes
460	HMDB0032460; CHEBI:87515	4-Pentenyl acetate	C7H12O2	127.0764893	Yes
460	HMDB0033793	4-Heptenoic acid	C7H12O2	127.0764893	Yes
460	HMDB0038271	2-Hexenyl formate	C7H12O2	127.0764893	Yes
460	HMDB0038962; C08487	Ethyl tiglate	C7H12O2	127.0764893	Yes
460	HMDB0039819	2,4-Dimethyl-2-pentenoic acid	C7H12O2	127.0764893	Yes
460	HMDB0040214	cis-3-Hexenyl formate	C7H12O2	127.0764893	Yes
460	HMDB0040591	Allyl butyrate	C7H12O2	127.0764893	Yes
467	HMDB0033561	2-Acetyl-4,5-dihydrothiazole	C5H7NOS	128.0176917	No
467	HMDB0037178	5-Methoxy-2-methylthiazole	C5H7NOS	128.0176917	No
467	HMDB0041282	2-Ethoxythiazole	C5H7NOS	128.0176917	No

470	HMDB0000267; C01879; CHEBI:18183	Pyroglutamic acid	C5H7NO3	128.0354268	Yes
470	HMDB0000805; C02237; CHEBI:16010	Pyrrolidonecarboxylic acid	C5H7NO3	128.0354268	Yes
470	HMDB0001369; C04281; CHEBI:27391	Pyrroline hydroxycarboxylic acid	C5H7NO3	128.0354268	Yes
470	HMDB0001843	N-Acryloylglycine	C5H7NO3	128.0354268	Yes
470	HMDB0002234; C04282; CHEBI:16352	1-Pyrroline-4-hydroxy-2-carboxylate	C5H7NO3	128.0354268	Yes
470	HMDB0060262	5-Oxoprolinate	C5H7NO3	128.0354268	Yes
470	HMDB0061093	dimethadione	C5H7NO3	128.0354268	Yes
470	HMDB0062585; C04281; CHEBI:6151	(3R,5S)-1-pyrroline-3-hydroxy-5-carboxylic Acid	C5H7NO3	128.0354268	Yes
471	HMDB0032341	Isoamyl isothiocyanate	C6H11NS	128.05439	Yes
471	HMDB0038433	1-Isothiocyanatopentane	C6H11NS	128.05439	Yes
471	HMDB0038973	2,5-Dihydro-2,4,5-trimethylthiazole	C6H11NS	128.05439	Yes
473	HMDB0000070; C00408; CHEBI:17964	Pipecolic acid	C6H11NO2	128.0717386	Yes
473	HMDB0000716; C00408; CHEBI:30913	L-Pipecolic acid	C6H11NO2	128.0717386	Yes
473	HMDB0004226; C05936; CHEBI:7386	N4-Acetylaminobutanal	C6H11NO2	128.0717386	Yes
473	HMDB0005960; CHEBI:41582	D-Pipecolic acid	C6H11NO2	128.0717386	Yes
473	HMDB0015212; C07500; CHEBI:63638	Vigabatrin	C6H11NO2	128.0717386	Yes
473	HMDB0029435	L-trans-4-Methyl-2-pyrrolidinecarboxylic acid	C6H11NO2	128.0717386	Yes
473	HMDB0029444	2-Pyrrolidineacetic acid	C6H11NO2	128.0717386	Yes
473	HMDB0059649	3-Acetamidobutanal	C6H11NO2	128.0717386	Yes
473	HMDB0062225; C03969; CHEBI:40547	(2E)-Decenoyl-ACP	C6H11NO2	128.0717386	Yes
473	HMDB0094696; CHEBI:90344	N-Methyl proline	C6H11NO2	128.0717386	Yes
473	HMDB0094697	1-Piperidine carboxylic acid	C6H11NO2	128.0717386	Yes
476	HMDB0001921; C07151; CHEBI:6801	1,1-Dimethylbiguanide	C4H11N5	128.0943377	No
484	HMDB0031853	2-Thiophenemethanethiol	C5H6S2	128.9846514	No
484	HMDB0032939	3-(Methylthio)thiophene	C5H6S2	128.9846514	No
484	HMDB0039787	2-Methyl-3-thiophenethiol	C5H6S2	128.9846514	No
484	HMDB0039788	5-Methyl-3-thiophenethiol	C5H6S2	128.9846514	No
486	HMDB0033548	4-Hydroxy-5-methyl-3(2H)-thiophenone	C5H6O2S	129.0015062	No
487	HMDB0000620; C02214; CHEBI:15670	Glutaconic acid	C5H6O4	129.0194392	Yes
487	HMDB0000634; C02226; CHEBI:17626	Citraconic acid	C5H6O4	129.0194392	Yes
487	HMDB0000749; C01732; CHEBI:16600	Mesaconic acid	C5H6O4	129.0194392	Yes
487	HMDB0002092; C00490; CHEBI:30838	Itaconic acid	C5H6O4	129.0194392	Yes
487	HMDB0013233; C02800; CHEBI:17204	Gamma-delta-Dioxovaleric acid	C5H6O4	129.0194392	Yes
487	HMDB0033809	Methyl hydrogen fumarate	C5H6O4	129.0194392	Yes
487	HMDB0059743; CHEBI:74524	2-Hydroxyglutaric acid lactone	C5H6O4	129.0194392	Yes
487	HMDB0060365; CHEBI:17415	2,5-Dioxopentanoate	C5H6O4	129.0194392	Yes
487	HMDB0061185	2-Pentendioate	C5H6O4	129.0194392	Yes
489	HMDB0031370	2-[(Methylthio)methyl]-2-butenal	C6H10OS	129.0389316	No
489	HMDB0037493	S-2-Propenyl propanethioate	C6H10OS	129.0389316	No
489	HMDB0040238	2-Ethyldihydro-3(2H)-thiophenone	C6H10OS	129.0389316	No
490	HMDB0000408; CHEBI:86365	2-Methyl-3-ketovaleric acid	C6H10O3	129.0557235	Yes
490	HMDB0000491; C03465; CHEBI:35932	3-Methyl-2-oxovaleric acid	C6H10O3	129.0557235	Yes
490	HMDB0000695; C00233; CHEBI:48430	Ketoleucine	C6H10O3	129.0557235	Yes
490	HMDB0001864; C00902; CHEBI:17308	2-Ketohexanoic acid	C6H10O3	129.0557235	Yes
490	HMDB0006024	Mevalonolactone	C6H10O3	129.0557235	Yes
490	HMDB0010717; C02122; CHEBI:28422	3-Oxohexanoic acid	C6H10O3	129.0557235	Yes
490	HMDB0012882; C06102; CHEBI:2490	Adipate semialdehyde	C6H10O3	129.0557235	Yes
490	HMDB0031214; CHEBI:87307	5-Ethoxy-4,5-dihydro-2(3H)furanone	C6H10O3	129.0557235	Yes
490	HMDB0031216	Ethyl acetoacetate	C6H10O3	129.0557235	Yes
490	HMDB0031218; C03500; CHEBI:386429	Ethyl 3-oxobutanoate	C6H10O3	129.0557235	Yes
490	HMDB0033286	Sherry lactone	C6H10O3	129.0557235	Yes
490	HMDB0033302	(4S,6S)-3,4,5,6-Tetrahydro-4-hydroxy-6-methyl-2H-pyr	C6H10O3	129.0557235	Yes
490	HMDB0033698	Acetoin acetate	C6H10O3	129.0557235	Yes
490	HMDB0041602	Methyl levulinate	C6H10O3	129.0557235	Yes
490	HMDB0059876	Pantolactone	C6H10O3	129.0557235	Yes
490	HMDB0061881; C02129; CHEBI:15888	4-Acetylbutyrate	C6H10O3	129.0557235	Yes
491	HMDB0029396	L-cis-3-Amino-2-pyrrolidinecarboxylic acid	C5H10N2O2	129.0670823	No
491	HMDB0030405	D-1-Amino-2-pyrrolidinecarboxylic acid	C5H10N2O2	129.0670823	No
492	HMDB0031537	3-Methylcyclohexanethiol	C7H14S	129.0745765	No
493	HMDB0000666; C17714; CHEBI:45571	Heptanoic acid	C7H14O2	129.0920709	No
493	HMDB0030028; C12290	Ethyl isovalerate	C7H14O2	129.0920709	No

493	HMDB0030052	Butyl propionate	C7H14O2	129.0920709	No
493	HMDB0031528; C12296; CHEBI:31725	Isopentyl acetate	C7H14O2	129.0920709	No
493	HMDB0031594	2-Methylhexanoic acid	C7H14O2	129.0920709	No
493	HMDB0031595	5-Methylhexanoic acid	C7H14O2	129.0920709	No
493	HMDB0031600	(±)-2-Pentanol acetate	C7H14O2	129.0920709	No
493	HMDB0032204	cis- and trans-2-Isopropyl-4-methyl-1,3-dioxolane	C7H14O2	129.0920709	No
493	HMDB0032874	Hexyl formate	C7H14O2	129.0920709	No
493	HMDB0033745	Ethyl 2-methylbutyrate	C7H14O2	129.0920709	No
493	HMDB0034166; CHEBI:50585	2-Methylbutyl acetate	C7H14O2	129.0920709	No
493	HMDB0034239	2-Methylpropyl propanoate	C7H14O2	129.0920709	No
493	HMDB0035238; CHEBI:77322	Methyl hexanoate	C7H14O2	129.0920709	No
493	HMDB0036209	Propyl 2-methylpropanoate	C7H14O2	129.0920709	No
493	HMDB0036237	Methyl (±)-2-methylpentanoate	C7H14O2	129.0920709	No
493	HMDB0036238	Methyl 4-methylpentanoate	C7H14O2	129.0920709	No
493	HMDB0038227	Isopropyl 2-methylpropanoate	C7H14O2	129.0920709	No
493	HMDB0039095	Pentyl acetate	C7H14O2	129.0920709	No
493	HMDB0039618	Propyl butyrate	C7H14O2	129.0920709	No
493	HMDB0039619	Isopropyl butyrate	C7H14O2	129.0920709	No
493	HMDB0040297	Ethyl pentanoate	C7H14O2	129.0920709	No
494	HMDB0002064; C02714; CHEBI:17768	N-Acetylputrescine	C6H14N2O	129.1032891	No
494	HMDB0060268	N-Acetylputrescinium	C6H14N2O	129.1032891	No
503	HMDB0000725; C01157; CHEBI:18095	4-Hydroxyproline	C5H9NO3	130.0510919	Yes
503	HMDB0000766; CHEBI:40992	N-Acetyl-L-alanine	C5H9NO3	130.0510919	Yes
503	HMDB0000783; CHEBI:89836	Propionylglycine	C5H9NO3	130.0510919	Yes
503	HMDB0001149; C00430; CHEBI:17549	5-Aminolevulinic acid	C5H9NO3	130.0510919	Yes
503	HMDB0002104; C01165; CHEBI:17232	L-Glutamic gamma-semialdehyde	C5H9NO3	130.0510919	Yes
503	HMDB0002113; C04397; CHEBI:87840	3-Hydroxy-L-proline	C5H9NO3	130.0510919	Yes
503	HMDB0006055; C01015; CHEBI:18240	4-Hydroxy-L-proline	C5H9NO3	130.0510919	Yes
503	HMDB0006272; C01110; CHEBI:49268	5-Amino-2-oxopentanoic acid	C5H9NO3	130.0510919	Yes
503	HMDB0036576; CHEBI:20392	4-Hydroxy-2-pyrrolidinecarboxylic acid	C5H9NO3	130.0510919	Yes
503	HMDB0059659; C05147; CHEBI:16889	Trans-3-hydroxy-L-proline	C5H9NO3	130.0510919	Yes
503	HMDB0060460; C03440; CHEBI:16231	cis-4-Hydroxy-D-proline	C5H9NO3	130.0510919	Yes
503	HMDB0061880; C01073; CHEBI:16682	N-Acetyl-beta-alanine	C5H9NO3	130.0510919	Yes
503	HMDB0240251; CHEBI:28397	cis-4-Hydroxyproline	C5H9NO3	130.0510919	Yes
504	HMDB0000064; C00300; CHEBI:16919	Creatine	C4H9N3O2	130.062635	Yes
504	HMDB0013222; C03065; CHEBI:15968	Beta-Guanidinopropionic acid	C4H9N3O2	130.062635	Yes
505	HMDB0040058	(±)-2-Propylthiazolidine	C6H13NS	130.069356	Yes
506	HMDB0000172; C00407; CHEBI:17191	L-Isoleucine	C6H13NO2	130.0872156	Yes
506	HMDB0000557; CHEBI:43433	L-Alloisoleucine	C6H13NO2	130.0872156	Yes
506	HMDB0000687; C00123; CHEBI:15603	L-Leucine	C6H13NO2	130.0872156	Yes
506	HMDB0001645; C01933; CHEBI:18347	L-Norleucine	C6H13NO2	130.0872156	Yes
506	HMDB0001901; C02378; CHEBI:16586	Aminocaproic acid	C6H13NO2	130.0872156	Yes
506	HMDB0003640; C02486; CHEBI:15604	Beta-Leucine	C6H13NO2	130.0872156	Yes
506	HMDB0013773; C01570; CHEBI:28225	D-Leucine	C6H13NO2	130.0872156	Yes
506	HMDB0029168	3-Aminocaproic acid	C6H13NO2	130.0872156	Yes
506	HMDB0033923; C16434; CHEBI:38264	(±)-erythro-Isoleucine	C6H13NO2	130.0872156	Yes
506	HMDB0036382	6-Deoxyfagomine	C6H13NO2	130.0872156	Yes
506	HMDB0060650; CHEBI:67144	N-(2-Hydroxyethyl)-morpholine	C6H13NO2	130.0872156	Yes
506	HMDB0061716; CHEBI:44212	N-methylvaline	C6H13NO2	130.0872156	Yes
506	HMDB0062203; C16439; CHEBI:25017	3,3,5-triiodo-L-thyronine-beta-D-glucuronoside	C6H13NO2	130.0872156	Yes
517	HMDB0000223; C00036; CHEBI:30744	Oxalacetic acid	C4H4O5	130.9987305	No
517	HMDB0039791	2,3-Dihydro-5-methyl-3-thiophenethiol	C5H8S2	130.9987305	No
517	HMDB0039792	4,5-Dihydro-2-methyl-3-thiophenethiol	C5H8S2	130.9987305	No
517	HMDB0039796	4,5-Dihydro-5-methyl-3-thiophenethiol	C5H8S2	130.9987305	No
521	HMDB0000576	Monoethyl malonic acid	C5H8O4	131.0353042	Yes
521	HMDB0000622; CHEBI:741548	Ethylmalonic acid	C5H8O4	131.0353042	Yes
521	HMDB0000661; C00489; CHEBI:17859	Glutaric acid	C5H8O4	131.0353042	Yes
521	HMDB0001844; C08645; CHEBI:29003	Methylsuccinic acid	C5H8O4	131.0353042	Yes
521	HMDB0002001; CHEBI:111517	Dimethylmalonic acid	C5H8O4	131.0353042	Yes
521	HMDB0006833; C00900; CHEBI:16444	2-Acetolactate	C5H8O4	131.0353042	Yes
521	HMDB0006855; C06010; CHEBI:18409	(S)-2-Acetolactate	C5H8O4	131.0353042	Yes

521	HMDB0029884	2-C-Methyl-1,4-erythrono-D-lactone	C5H8O4	131.0353042	Yes
521	HMDB0033958	2-Deoxy-L-ribono-1,4-lactone	C5H8O4	131.0353042	Yes
521	HMDB0040531	2-Hydroxy-4-oxopentanoic acid	C5H8O4	131.0353042	Yes
521	HMDB0061879; CHEBI:75146	(S)-Methylbutanethioic acid	C5H8O4	131.0353042	Yes
522	HMDB0000026; C02642; CHEBI:18261	Ureidopropionic acid	C4H8N2O3	131.0462522	Yes
522	HMDB0000168; C00152; CHEBI:17196	L-Asparagine	C4H8N2O3	131.0462522	Yes
522	HMDB0011733; C02037; CHEBI:17201	Glycyl-glycine	C4H8N2O3	131.0462522	Yes
522	HMDB0012265; C01043; CHEBI:15737	N-Carbamoylsarcosine	C4H8N2O3	131.0462522	Yes
522	HMDB0033780; C01905; CHEBI:28159	D-Asparagine	C4H8N2O3	131.0462522	Yes
523	HMDB0031519; CHEBI:77856	4-Mercapto-4-methyl-2-pentanone	C6H12OS	131.0534828	No
523	HMDB0032237	2,5-Dimethyl-3-mercaptotetrahydrofuran	C6H12OS	131.0534828	No
523	HMDB0032430	4-(Methylthio)-2-pentanone	C6H12OS	131.0534828	No
523	HMDB0035176	3-Mercapto-2-methylpentanal	C6H12OS	131.0534828	No
523	HMDB0039843	S-Methyl 3-methylthiobutyrate	C6H12OS	131.0534828	No
524	HMDB0033171	1-Methylpyrrolo[1,2-a]pyrazine	C8H8N2	131.0615965	No
524	HMDB0033173	4-Methylpyrrolo[1,2-a]pyrazine	C8H8N2	131.0615965	No
525	HMDB0000317	2-Hydroxy-3-methylpentanoic acid	C6H12O3	131.0715086	Yes
525	HMDB0000409; CHEBI:78842	(5R)-5-Hydroxyhexanoic acid	C6H12O3	131.0715086	Yes
525	HMDB0000525; CHEBI:131434	5-Hydroxyhexanoic acid	C6H12O3	131.0715086	Yes
525	HMDB0000624; C03264; CHEBI:55534	D-Leucic acid	C6H12O3	131.0715086	Yes
525	HMDB0000665; CHEBI:59783	Leucinic acid	C6H12O3	131.0715086	Yes
525	HMDB0000746; CHEBI:44510	Hydroxyisocaproic acid	C6H12O3	131.0715086	Yes
525	HMDB0001624; CHEBI:86542	2-Hydroxycaproic acid	C6H12O3	131.0715086	Yes
525	HMDB0001975	2-Ethyl-2-Hydroxybutyric acid	C6H12O3	131.0715086	Yes
525	HMDB0010718	(R)-3-Hydroxyhexanoic acid	C6H12O3	131.0715086	Yes
525	HMDB0012843; C06103; CHEBI:17869	6-Hydroxyhexanoic acid	C6H12O3	131.0715086	Yes
525	HMDB0029166	2-Methyl-3-hydroxyvaleric acid	C6H12O3	131.0715086	Yes
525	HMDB0032158	(+/-)-1-Acetoxy-1-ethoxyethane	C6H12O3	131.0715086	Yes
525	HMDB0032456; C07834; CHEBI:27909	Paraldehyde	C6H12O3	131.0715086	Yes
525	HMDB0033851	4,4-Dimethoxy-2-butanone	C6H12O3	131.0715086	Yes
525	HMDB0040409; CHEBI:87685	Ethyl (±)-3-hydroxybutyrate	C6H12O3	131.0715086	Yes
525	HMDB0041844	Butoxyacetic acid	C6H12O3	131.0715086	Yes
525	HMDB0059770	Threo-3-Hydroxy-2-methylbutyric acid	C6H12O3	131.0715086	Yes
525	HMDB0061652; CHEBI:37035	3-hydroxyhexanoic acid	C6H12O3	131.0715086	Yes
526	HMDB0000214; C00077; CHEBI:15729	Ornithine	C5H12N2O2	131.0827168	Yes
526	HMDB0003374; C00515; CHEBI:16176	D-Ornithine	C5H12N2O2	131.0827168	Yes
531	HMDB0038442	1-Isothiocyanato-2-(methylthio)ethane	C4H7NS2	131.9949988	No
534	HMDB0000191; C00049; CHEBI:17053	L-Aspartic acid	C4H7NO4	132.0302478	Yes
534	HMDB0006483; C00402; CHEBI:17364	D-Aspartic acid	C4H7NO4	132.0302478	Yes
534	HMDB0011753; C19911; CHEBI:24786	Iminodiacetic acid	C4H7NO4	132.0302478	Yes
534	HMDB0062186; C16433; CHEBI:22660	1-deoxy-1-(N6-lysino)-D-fructose	C4H7NO4	132.0302478	Yes
535	HMDB0004094; C05658; CHEBI:17840	Indoxyl	C8H7NO	132.0455529	Yes
535	HMDB0029757; C03766; CHEBI:16667	4-Hydroxybenzeneacetonitrile	C8H7NO	132.0455529	Yes
535	HMDB0032390; CHEBI:51603	2-Methyl-4,5-benzoxazole	C8H7NO	132.0455529	Yes
535	HMDB0034666; CHEBI:16910	(±)-2-Hydroxy-2-phenylacetonitrile	C8H7NO	132.0455529	Yes
535	HMDB0059805; CHEBI:89649	5-Hydroxyindole	C8H7NO	132.0455529	Yes
535	HMDB0060486; C00561; CHEBI:18450	Mandelonitrile	C8H7NO	132.0455529	Yes
535	HMDB0061918; C12312; CHEBI:31697	1,3-Dihydro-(2H)-indol-2-one	C8H7NO	132.0455529	Yes
536	HMDB0031658	L-2-Amino-5-hydroxypentanoic acid	C5H11NO3	132.0666827	No
536	HMDB0032356	N-Lactoyl ethanolamine	C5H11NO3	132.0666827	No
536	HMDB0039499	1,4-Dideoxy-1,4-imino-D-ribitol	C5H11NO3	132.0666827	No
538	HMDB0012489; CHEBI:114116	1,2,3,4-Tetrahydroisoquinoline	C9H11N	132.0820604	No
538	HMDB0014890; C07155	Tranylcypromine	C9H11N	132.0820604	No
548	HMDB0000156; C00149; CHEBI:30797	L-Malic acid	C4H6O5	133.0144021	Yes
548	HMDB0000744; C00711; CHEBI:6650	Malic acid	C4H6O5	133.0144021	Yes
548	HMDB0031518; C00497; CHEBI:30796	D-Malic acid	C4H6O5	133.0144021	Yes
548	HMDB0032872	Velcorin	C4H6O5	133.0144021	Yes
548	HMDB0038974	3,3-Dimethyl-1,2-dithiolane	C5H10S2	133.0144021	Yes
548	HMDB0039789	Tetrahydro-2-methyl-2-thiophenethiol	C5H10S2	133.0144021	Yes
548	HMDB0039790	Tetrahydro-2-methyl-3-thiophenethiol	C5H10S2	133.0144021	Yes
549	HMDB0032469; C09921; CHEBI:479525	Phthalide	C8H6O2	133.0296238	Yes

549	HMDB0061916	Phenylglyoxal	C8H6O2	133.0296238	Yes
549	HMDB0129378	1-benzofuran-4-ol	C8H6O2	133.0296238	Yes
551	HMDB0000421	2,3-Dihydroxyvaleric acid	C5H10O4	133.0505423	Yes
551	HMDB0001292; C06257	1-Deoxy-D-xylulose	C5H10O4	133.0505423	Yes
551	HMDB0003224; C01801; CHEBI:28816	Deoxyribose	C5H10O4	133.0505423	Yes
551	HMDB0012141; C04272; CHEBI:15684	(R)-2,3-Dihydroxy-isovalerate	C5H10O4	133.0505423	Yes
551	HMDB0012485	(R)-glycerol 1-acetate	C5H10O4	133.0505423	Yes
551	HMDB0029576	2,3-Dihydroxy-2-methylbutanoic acid	C5H10O4	133.0505423	Yes
551	HMDB0127615	3-hydroxy-2-(hydroxymethyl)-2-methylpropanoic acid	C5H10O4	133.0505423	Yes
552	HMDB0029639	(4-Methylphenyl)acetaldehyde	C9H10O	133.065938	No
552	HMDB0029697; C02394; CHEBI:33226	Cinnamyl alcohol	C9H10O	133.065938	No
552	HMDB0029698; C02394; CHEBI:33227	trans-Cinnamyl alcohol	C9H10O	133.065938	No
552	HMDB0030839	(Z)-4-(1-Propenyl)phenol	C9H10O	133.065938	No
552	HMDB0031626	2-Phenylpropanal	C9H10O	133.065938	No
552	HMDB0032024	4-Ethylbenzaldehyde	C9H10O	133.065938	No
552	HMDB0032142	2,4-Dimethylbenzaldehyde	C9H10O	133.065938	No
552	HMDB0032608	4''-Methylacetophenone	C9H10O	133.065938	No
552	HMDB0032623; CHEBI:425902	1-Phenyl-1-propanone	C9H10O	133.065938	No
552	HMDB0033716	3-Phenylpropanal	C9H10O	133.065938	No
552	HMDB0034107; C16930; CHEBI:50158	Chavicol	C9H10O	133.065938	No
552	HMDB0034563	7-Nonene-3,5-diyn-1-ol	C9H10O	133.065938	No
552	HMDB0036441	o-Vinylanisole	C9H10O	133.065938	No
552	HMDB0059601; C01710; CHEBI:16697	Indan-1-ol	C9H10O	133.065938	No
552	HMDB0059895	m-Methylacetophenone	C9H10O	133.065938	No
557	HMDB0032930; CHEBI:45993	Benzothiazole	C7H5NS	134.0071387	No
559	HMDB0032931	2-Benzoxazolol	C7H5NO2	134.0249231	No
561	HMDB0000034; C00147; CHEBI:16708	Adenine	C5H5N5	134.0474863	Yes
562	HMDB0001250; C07565; CHEBI:28884	N-Acetylarylamine	C8H9NO	134.061187	No
562	HMDB0010715; C02505; CHEBI:16562	2-Phenylacetamide	C8H9NO	134.061187	No
562	HMDB0032628	2-Aminoacetophenone	C8H9NO	134.061187	No
562	HMDB0032630; CHEBI:91110	2''-Aminoacetophenone	C8H9NO	134.061187	No
562	HMDB0037071	2-Acetyl-4-methylpyridine	C8H9NO	134.061187	No
562	HMDB0037072	2-Acetyl-6-methylpyridine	C8H9NO	134.061187	No
562	HMDB0040008	2,3-Dihydro-1H-pyrrolizine-5-carboxaldehyde	C8H9NO	134.061187	No
562	HMDB0040013	5-(2-Furanyl)-3,4-dihydro-2H-pyrrole	C8H9NO	134.061187	No
562	HMDB0040222	2-Acetyl-5-methylpyridine	C8H9NO	134.061187	No
562	HMDB0040223	4-Acetyl-2-methylpyridine	C8H9NO	134.061187	No
562	HMDB0040224	4-Acetyl-3-methylpyridine	C8H9NO	134.061187	No
576	HMDB0000613; CHEBI:37655	Erythronic acid	C4H8O5	135.0300431	Yes
576	HMDB0000943; C01620; CHEBI:49059	Threonic acid	C4H8O5	135.0300431	Yes
576	HMDB0031160	1-Pentanesulfenothioic acid	C5H12S2	135.0300431	Yes
576	HMDB0033053	Ethyl propyl disulfide	C5H12S2	135.0300431	Yes
576	HMDB0033054	Ethyl isopropyl disulfide	C5H12S2	135.0300431	Yes
576	HMDB0062620; C01620; CHEBI:15908	L-threonic Acid	C4H8O5	135.0300431	Yes
577	HMDB0000209; C07086; CHEBI:30745	Phenylacetic acid	C8H8O2	135.0451893	No
577	HMDB0002340; C07215; CHEBI:36632	2-Methylbenzoic acid	C8H8O2	135.0451893	No
577	HMDB0003767; C03765; CHEBI:15621	4-Hydroxyphenylacetaldehyde	C8H8O2	135.0451893	No
577	HMDB0029635; C01454; CHEBI:36635	4-Methylbenzoic acid	C8H8O2	135.0451893	No
577	HMDB0029686; C10761; CHEBI:366673	4-Methoxybenzaldehyde	C8H8O2	135.0451893	No
577	HMDB0031459	3-Methoxybenzaldehyde	C8H8O2	135.0451893	No
577	HMDB0032400	3-(5-Methyl-2-furyl)prop-2-enal	C8H8O2	135.0451893	No
577	HMDB0032568	2''-Hydroxyacetophenone	C8H8O2	135.0451893	No
577	HMDB0032603	2-Hydroxy-4-methylbenzaldehyde	C8H8O2	135.0451893	No
577	HMDB0033128	4-(2-Furanyl)-3-buten-2-one	C8H8O2	135.0451893	No
577	HMDB0033766	2-Methoxybenzaldehyde	C8H8O2	135.0451893	No
577	HMDB0033968; CHEBI:72775	Methyl benzoate	C8H8O2	135.0451893	No
577	HMDB0038176	alpha-Methyl-2-furanacrolein	C8H8O2	135.0451893	No
577	HMDB0040638	3-(3-Furanyl)-2-methyl-2-propenal	C8H8O2	135.0451893	No
577	HMDB0040733; C00548; CHEBI:8082	Phenyl acetate	C8H8O2	135.0451893	No
577	HMDB0041485; C05613	Benzyl formate	C8H8O2	135.0451893	No
577	HMDB0062810; C07211; CHEBI:10589	M-toluic Acid	C8H8O2	135.0451893	No

577	HMDB0124942	4-ethenylbenzene-1,2-diol	C8H8O2	135.0451893	No
577	HMDB0125535	5-ethenylbenzene-1,3-diol	C8H8O2	135.0451893	No
577	HMDB0126385	4-ethenylbenzene-1,3-diol	C8H8O2	135.0451893	No
578	HMDB0003152; CHEBI:64399	N-Methylnicotinamide	C7H8N2O	135.0565198	Yes
578	HMDB0030001	2-Acetyl-3-methylpyrazine	C7H8N2O	135.0565198	Yes
578	HMDB0033947; C17512	2-Aminobenzamide	C7H8N2O	135.0565198	Yes
578	HMDB0037819	4-Acetyl-2-methylpyrimidine	C7H8N2O	135.0565198	Yes
578	HMDB0059711; CHEBI:68440	2-Methylnicotinamide	C7H8N2O	135.0565198	Yes
578	HMDB0062267	1,2-Diacylglycerol-Bile-PC-pool	C7H8N2O	135.0565198	Yes
578	HMDB0062268	1,2-Diacylglycerol-LD-PC-pool	C7H8N2O	135.0565198	Yes
580	HMDB0029667	2,3,6-Trimethylphenol	C9H12O	135.0803559	No
580	HMDB0031312	Benzyl ethyl ether	C9H12O	135.0803559	No
580	HMDB0031446	p-Isopropylphenol	C9H12O	135.0803559	No
580	HMDB0031627	1-Phenyl-1-propanol	C9H12O	135.0803559	No
580	HMDB0031628	2-Phenyl-1-propanol	C9H12O	135.0803559	No
580	HMDB0032029; CHEBI:38506	2-Isopropylphenol	C9H12O	135.0803559	No
580	HMDB0032149	1-Methoxy-2,4-dimethylbenzene	C9H12O	135.0803559	No
580	HMDB0032441	Nona-2,4,6-trienal	C9H12O	135.0803559	No
580	HMDB0032544	2,4,6-Trimethylphenol	C9H12O	135.0803559	No
580	HMDB0032621	(2-Methoxyethyl)benzene	C9H12O	135.0803559	No
580	HMDB0032624	2-Propylphenol	C9H12O	135.0803559	No
580	HMDB0032625; C14311	4-Propylphenol	C9H12O	135.0803559	No
580	HMDB0033962	3-Phenyl-1-propanol	C9H12O	135.0803559	No
580	HMDB0034378	(±)-1-(4-Methylphenyl)ethanol	C9H12O	135.0803559	No
580	HMDB0038984	4-Ethyl-2-methylphenol	C9H12O	135.0803559	No
580	HMDB0038985	3-Ethyl-5-methylphenol	C9H12O	135.0803559	No
580	HMDB0038986	3-Ethyl-4-methylphenol	C9H12O	135.0803559	No
580	HMDB0038987	2-Ethyl-5-methylphenol	C9H12O	135.0803559	No
580	HMDB0038988	2-Ethyl-4-methylphenol	C9H12O	135.0803559	No
580	HMDB0039782	2-(1-Pentenyl)furan	C9H12O	135.0803559	No
580	HMDB0059896	6-Ethyl-o-cresol	C9H12O	135.0803559	No
580	HMDB0125105	1-ethyl-3-methoxybenzene	C9H12O	135.0803559	No
581	HMDB0014918; C07430; CHEBI:248018	Phenelzine	C8H12N2	135.0929174	No
581	HMDB0015644; CHEBI:35677	Betahistine	C8H12N2	135.0929174	No
581	HMDB0029727; CHEBI:254553	5-Ethyl-2,3-dimethylpyrazine	C8H12N2	135.0929174	No
581	HMDB0032276	2-Ethyl-3,(5 or 6)-dimethylpyrazine	C8H12N2	135.0929174	No
581	HMDB0036584; CHEBI:133246	Tetramethylpyrazine	C8H12N2	135.0929174	No
581	HMDB0036808	2,5-Diethylpyrazine	C8H12N2	135.0929174	No
581	HMDB0036809	2,6-Diethylpyrazine	C8H12N2	135.0929174	No
581	HMDB0037151	2-Isopropyl-5-methylpyrazine	C8H12N2	135.0929174	No
581	HMDB0040000	2-Methyl-5-propylpyrazine	C8H12N2	135.0929174	No
581	HMDB0040299	2-Ethyl-3,5-dimethylpyrazine	C8H12N2	135.0929174	No
581	HMDB0041252	2-Methyl-3-propylpyrazine	C8H12N2	135.0929174	No
581	HMDB0041253	2,3-Diethylpyrazine	C8H12N2	135.0929174	No
592	HMDB0000875; C01004; CHEBI:18123	Trigonelline	C7H7NO2	136.0405969	Yes
592	HMDB0001123; C00108; CHEBI:30754	2-Aminobenzoic acid	C7H7NO2	136.0405969	Yes
592	HMDB0001392; C00568; CHEBI:30753	p-Aminobenzoic acid	C7H7NO2	136.0405969	Yes
592	HMDB0001538; CHEBI:1220323	3-Pyridylacetic acid	C7H7NO2	136.0405969	Yes
592	HMDB0001891; CHEBI:30761	m-Aminobenzoic acid	C7H7NO2	136.0405969	Yes
592	HMDB0015687; CHEBI:32114	Salicylamide	C7H7NO2	136.0405969	Yes
592	HMDB0029806; CHEBI:451700	Methyl nicotinate	C7H7NO2	136.0405969	Yes
592	HMDB0060722	2-Pyridylacetic acid	C7H7NO2	136.0405969	Yes
596	HMDB0000306; C00483; CHEBI:15760	Tyramine	C8H11NO	136.0768766	No
596	HMDB0001065; C02735; CHEBI:16343	2-Hydroxyphenethylamine	C8H11NO	136.0768766	No
596	HMDB0001466; C01183; CHEBI:17735	Dimethylaniline-N-oxide	C8H11NO	136.0768766	No
596	HMDB0004989; CHEBI:341165	m-Tyramine	C8H11NO	136.0768766	No
596	HMDB0029759	2-Acetyl-1-ethylpyrrole	C8H11NO	136.0768766	No
596	HMDB0039657	1,2,3,4,5,6-Hexahydro-7H-cyclopenta[b]pyridin-7-one	C8H11NO	136.0768766	No
596	HMDB0040059	(±)-2-(2-Furanyl)pyrrolidine	C8H11NO	136.0768766	No
596	HMDB0060681; C16570; CHEBI:55545	4-Hydroxy-2,6-dimethylaniline	C8H11NO	136.0768766	No
604	HMDB0036573; CHEBI:39196	1,3,5-Trithiane	C3H6S3	136.9561755	No

604	HMDB0040452; CHEBI:39194	1,2,3-Trithiane	C3H6S3	136.9561755	No
611	HMDB0000500; C00156; CHEBI:30763	4-Hydroxybenzoic acid	C7H6O3	137.0244702	No
611	HMDB0001895; C00805; CHEBI:16914	Salicylic acid	C7H6O3	137.0244702	No
611	HMDB0002466; C00587; CHEBI:30764	3-Hydroxybenzoic acid	C7H6O3	137.0244702	No
611	HMDB0004062; C05585; CHEBI:28508	Gentisate aldehyde	C7H6O3	137.0244702	No
611	HMDB0032576	2-Methoxy-1,4-benzoquinone	C7H6O3	137.0244702	No
611	HMDB0032920	alpha-Furyl methyl diketone	C7H6O3	137.0244702	No
611	HMDB0033812; C10832	Sesamol	C7H6O3	137.0244702	No
611	HMDB0059965; C16700; CHEBI:50205	3,4-Dihydroxybenzaldehyde	C7H6O3	137.0244702	No
611	HMDB0062484; CHEBI:50198	keratan sulfate II (core 2-linked), degradation product	C7H6O3	137.0244702	No
612	HMDB0012182	8-Hydroxypurine	C5H6N4O	137.0475774	Yes
613	HMDB0004284; C06044; CHEBI:1879	Tyrosol	C8H10O2	137.0607603	Yes
613	HMDB0029563	3-Acetyl-2,5-dimethylfuran	C8H10O2	137.0607603	Yes
613	HMDB0029671; CHEBI:1167379	1,4-Dimethoxybenzene	C8H10O2	137.0607603	Yes
613	HMDB0029672	1,3-Dimethoxybenzene	C8H10O2	137.0607603	Yes
613	HMDB0032036	4-Ethoxyphenol	C8H10O2	137.0607603	Yes
613	HMDB0032136	2-Methoxy-4-methylphenol	C8H10O2	137.0607603	Yes
613	HMDB0032137	2,6-Dimethyl-1,4-benzenediol	C8H10O2	137.0607603	Yes
613	HMDB0032139; CHEBI:59114	1,2-Dimethoxybenzene	C8H10O2	137.0607603	Yes
613	HMDB0032159	2-Acetyl-3,5-dimethylfuran	C8H10O2	137.0607603	Yes
613	HMDB0032290	1-(2-Furyl)butan-3-one	C8H10O2	137.0607603	Yes
613	HMDB0034241	4-Methoxybenzyl alcohol	C8H10O2	137.0607603	Yes
613	HMDB0039802	1-(2-Furanyl)-1-butanone	C8H10O2	137.0607603	Yes
613	HMDB0040174	4-Ethyl-1,2-benzenediol	C8H10O2	137.0607603	Yes
613	HMDB0040177; C06728; CHEBI:16572	3-Ethyl-1,2-benzenediol	C8H10O2	137.0607603	Yes
613	HMDB0041607; CHEBI:64275	2-Phenoxyethanol	C8H10O2	137.0607603	Yes
613	HMDB0125590	5-ethylbenzene-1,3-diol	C8H10O2	137.0607603	Yes
613	HMDB0126388	4-ethylbenzene-1,3-diol	C8H10O2	137.0607603	Yes
614	HMDB0031850	2-Ethoxy-3-methylpyrazine	C7H10N2O	137.0718913	No
614	HMDB0040054	3-Ethyl-2-methoxypyrazine	C7H10N2O	137.0718913	No
614	HMDB0040142	2-Ethoxy-5-methylpyrazine	C7H10N2O	137.0718913	No
614	HMDB0040144	2-Ethoxy-6-methylpyrazine	C7H10N2O	137.0718913	No
614	HMDB0040911	2-Methoxy-3,5-dimethylpyrimidine	C7H10N2O	137.0718913	No
628	HMDB0012153	3,4-Dihydroxybenzylamine	C7H9NO2	138.056225	Yes
628	HMDB0038174	5-Acetyl-2,4-dimethyloxazole	C7H9NO2	138.056225	Yes
628	HMDB0059856	Ethyl 2-pyrrolecarboxylate	C7H9NO2	138.056225	Yes
641	HMDB0032571	2-Methoxybenzenethiol	C7H8OS	139.0232497	Yes
641	HMDB0032922	Kahweofuran	C7H8OS	139.0232497	Yes
641	HMDB0033130	2-Acetyl-5-methylthiophene	C7H8OS	139.0232497	Yes
641	HMDB0033159	2-Propanoylthiophene	C7H8OS	139.0232497	Yes
641	HMDB0041281	2-(Methylthio)phenol	C7H8OS	139.0232497	Yes
641	HMDB0041502	2-Acetyl-3-methylthiophene	C7H8OS	139.0232497	Yes
643	HMDB0002144; CHEBI:108884	1,3-Dimethyluracil	C6H8N2O2	139.0490997	Yes
643	HMDB0002271; CHEBI:232595	Imidazolepropionic acid	C6H8N2O2	139.0490997	Yes
643	HMDB0002820; C05828	Methylimidazoleacetic acid	C6H8N2O2	139.0490997	Yes
643	HMDB0004988	Pi-Methylimidazoleacetic acid	C6H8N2O2	139.0490997	Yes
644	HMDB0040236	3,4-Diethylthiophene	C8H12S	139.0589662	Yes
645	HMDB0032311	2,4-Hexadienyl acetate	C8H12O2	139.0750888	Yes
645	HMDB0036176	3-Ethyl-2-hydroxy-4-methyl-2-cyclopenten-1-one	C8H12O2	139.0750888	Yes
645	HMDB0037167	3-Ethyl-5-methyl-1,2-cyclopentanedione	C8H12O2	139.0750888	Yes
645	HMDB0037196	Ethyl 2-methyl-3,4-pentadienoate	C8H12O2	139.0750888	Yes
645	HMDB0037625	Allyl tiglate	C8H12O2	139.0750888	Yes
645	HMDB0039651	3-Propyl-1,2-cyclopentanedione	C8H12O2	139.0750888	Yes
645	HMDB0039652	2-Hydroxy-3,4,5-trimethyl-2-cyclopenten-1-one	C8H12O2	139.0750888	Yes
645	HMDB0039673	4-Ethyl-2-hydroxy-3-methyl-2-cyclopenten-1-one	C8H12O2	139.0750888	Yes
645	HMDB0039680	3,5,5-Trimethyl-1,2-cyclopentanedione	C8H12O2	139.0750888	Yes
645	HMDB0039681	3-Isopropyl-1,2-cyclopentanedione	C8H12O2	139.0750888	Yes
645	HMDB0040463	Ethyl sorbate	C8H12O2	139.0750888	Yes
645	HMDB0040526	3,4,4-Trimethyl-1,2-cyclopentanedione	C8H12O2	139.0750888	Yes
645	HMDB0060682; C16656; CHEBI:80642	2-Propyl-2,4-pentadienoic acid	C8H12O2	139.0750888	Yes
648	HMDB0030957	(3Z,6Z)-3,6-Nonadien-1-ol	C9H16O	139.1128068	No

648	HMDB0030958	(Z)-6-Nonenal	C9H16O	139.1128068	No
648	HMDB0031262	2,6-Nonadien-1-ol	C9H16O	139.1128068	No
648	HMDB0031269	2-Nonenal	C9H16O	139.1128068	No
648	HMDB0031834	(±)-2,6-Dimethyl-5-heptenal	C9H16O	139.1128068	No
648	HMDB0032535	trans-4-Nonenal	C9H16O	139.1128068	No
648	HMDB0033554	2-Methyl-2-octenal	C9H16O	139.1128068	No
648	HMDB0033794	(±)-2,2,6-Trimethylcyclohexanone	C9H16O	139.1128068	No
648	HMDB0034758	8-Nonen-3-one	C9H16O	139.1128068	No
648	HMDB0034761	5-Nonen-2-one	C9H16O	139.1128068	No
648	HMDB0034764	3-Nonen-2-one	C9H16O	139.1128068	No
648	HMDB0034845	2,4-Nonadien-1-ol	C9H16O	139.1128068	No
648	HMDB0035916	(E)-2-Nonen-4-one	C9H16O	139.1128068	No
648	HMDB0035926	8-Nonen-2-one	C9H16O	139.1128068	No
648	HMDB0035927	1-Nonen-4-one	C9H16O	139.1128068	No
648	HMDB0037001	Santene hydrate	C9H16O	139.1128068	No
648	HMDB0037150	2-Ethyl-2-heptenal	C9H16O	139.1128068	No
648	HMDB0037170	3-Methylene-2-octanone	C9H16O	139.1128068	No
648	HMDB0038067	(3E,6Z)-3,6-Nonadien-1-ol	C9H16O	139.1128068	No
648	HMDB0038400	(2xi,4xi)-2,4-Nonadien-1-ol	C9H16O	139.1128068	No
648	HMDB0039461	7-Methyl-3-octen-2-one	C9H16O	139.1128068	No
648	HMDB0059828	3-Nonen-5-one	C9H16O	139.1128068	No
648	HMDB0062671; CHEBI:84226	(3E)-2,3,4-trimethylhex-3-enal	C9H16O	139.1128068	No
654	HMDB0001096; C00169; CHEBI:17672	Carbamoyl phosphate	CH4NO5P	139.9756645	No
661	HMDB0014731; C07505; CHEBI:4887	Ethosuximide	C7H11NO2	140.0717535	No
661	HMDB0029427; C08287	L-Hypoglycin A	C7H11NO2	140.0717535	No
661	HMDB0030352; C10128; CHEBI:226311	Arecaidine	C7H11NO2	140.0717535	No
662	HMDB0003431; C00860; CHEBI:16255	L-Histidinol	C6H11N3O	140.0828606	No
663	HMDB0030324	(R)-Pelletierine	C8H15NO	140.1083952	No
663	HMDB0030325; C06182	(±)-Pelletierine	C8H15NO	140.1083952	No
663	HMDB0030338; C10864	Physoperuvine	C8H15NO	140.1083952	No
663	HMDB0033364	Conhydrinone	C8H15NO	140.1083952	No
663	HMDB0039839	1-(1-Pyrrolidinyl)-2-butanone	C8H15NO	140.1083952	No
663	HMDB0039840	3-(1-Pyrrolidinyl)-2-butanone	C8H15NO	140.1083952	No
669	HMDB0029719	Methyl thiophene-2-carboxylate	C6H6O2S	141.001827	No
669	HMDB0037731	S-(2-Furanylmethyl) methanethioate	C6H6O2S	141.001827	No
669	HMDB0037762	Methyl 2-thiofuroate	C6H6O2S	141.001827	No
670	HMDB0002349; CHEBI:27036	trans-trans-Muconic acid	C6H6O4	141.020043	Yes
670	HMDB0002432; CHEBI:563718	Sumiki''s acid	C6H6O4	141.020043	Yes
670	HMDB0006331; C02480; CHEBI:16508	cis,cis-Muconic acid	C6H6O4	141.020043	Yes
670	HMDB0032923; C14516; CHEBI:34805	Kojic acid	C6H6O4	141.020043	Yes
670	HMDB0032988	5-Hydroxymaltol	C6H6O4	141.020043	Yes
670	HMDB0059762	2,3-Methylenesuccinic acid	C6H6O4	141.020043	Yes
670	HMDB0125218	benzene-1,2,3,5-tetrol	C6H6O4	141.020043	Yes
670	HMDB0134002	benzene-1,2,3,4-tetrol	C6H6O4	141.020043	Yes
674	HMDB0033551	5-Ethyl-3-hydroxy-4-methyl-2(5H)-furanone	C7H10O3	141.0558597	No
674	HMDB0033736	Homofureanol	C7H10O3	141.0558597	No
674	HMDB0034230	Mesifurane	C7H10O3	141.0558597	No
674	HMDB0041426	Erinapyrone A	C7H10O3	141.0558597	No
674	HMDB0041427	Erinapyrone B	C7H10O3	141.0558597	No
676	HMDB0032860; C14082; CHEBI:50717	1-Methylnaphthalene	C11H10	141.0709926	No
678	HMDB0000392	2-Octenoic acid	C8H14O2	141.0920176	Yes
678	HMDB0001568; CHEBI:86544	trans-2-Octenoic acid	C8H14O2	141.0920176	Yes
678	HMDB0013897; C16648; CHEBI:410867	4-ene-Valproic acid	C8H14O2	141.0920176	Yes
678	HMDB0013902; C16653; CHEBI:288752	2-ene-Valproic acid	C8H14O2	141.0920176	Yes
678	HMDB0013903; C16654	(3Z)-2-Propylpent-3-enoic acid	C8H14O2	141.0920176	Yes
678	HMDB0013904	(3E)-2-Propylpent-3-enoic acid	C8H14O2	141.0920176	Yes
678	HMDB0029761	Ethyl (±)-2-methyl-4-pentenoate	C8H14O2	141.0920176	Yes
678	HMDB0031293	2,3-Octanedione	C8H14O2	141.0920176	Yes
678	HMDB0031343	Methyl cyclohexanecarboxylate	C8H14O2	141.0920176	Yes
678	HMDB0031352; C12297	Cyclohexyl acetate	C8H14O2	141.0920176	Yes
678	HMDB0031403; CHEBI:37277	Cyclohexaneacetic acid	C8H14O2	141.0920176	Yes

678	HMDB0032164	Allyl valerate	C8H14O2	141.0920176	Yes
678	HMDB0032207	cis-5-Octenoic acid	C8H14O2	141.0920176	Yes
678	HMDB0032266	Ethyl (E)-3-hexenoate	C8H14O2	141.0920176	Yes
678	HMDB0032267; CHEBI:87299	Ethyl 5-hexenoate	C8H14O2	141.0920176	Yes
678	HMDB0032268	Ethyl 2E-hexenoate	C8H14O2	141.0920176	Yes
678	HMDB0032278	Ethyl trans-2-methyl-2-pentenoate	C8H14O2	141.0920176	Yes
678	HMDB0032533	trans-3-Hexenyl acetate	C8H14O2	141.0920176	Yes
678	HMDB0035422	4-Butyl-gamma-butyrolactone	C8H14O2	141.0920176	Yes
678	HMDB0036212	2-Methylpropyl (2E)-butenoate	C8H14O2	141.0920176	Yes
678	HMDB0036236	2-Methylallyl butyrate	C8H14O2	141.0920176	Yes
678	HMDB0037622	Ethyl 2-methyl-3-pentenoate	C8H14O2	141.0920176	Yes
678	HMDB0037624	Isopropyl tiglate	C8H14O2	141.0920176	Yes
678	HMDB0037630	xi-Tetrahydro-3-propyl-2H-pyran-2-one	C8H14O2	141.0920176	Yes
678	HMDB0038036; C19318	2-Propenyl 3-methylbutanoate	C8H14O2	141.0920176	Yes
678	HMDB0038310	xi-Tetrahydro-6-propyl-2H-pyran-2-one	C8H14O2	141.0920176	Yes
678	HMDB0039793; CHEBI:37316	(E)-4-Octenoic acid	C8H14O2	141.0920176	Yes
678	HMDB0039872	Methyl 2-heptenoate	C8H14O2	141.0920176	Yes
678	HMDB0040212	2-Hexenyl acetate	C8H14O2	141.0920176	Yes
678	HMDB0040215; C19757; CHEBI:61316	cis-3-Hexenyl acetate	C8H14O2	141.0920176	Yes
678	HMDB0059908; CHEBI:87560	Ethyl-3-hexanoate	C8H14O2	141.0920176	Yes
679	HMDB0013821	2-Methyl-4-heptanone	C9H18O	141.1285556	No
679	HMDB0029557; CHEBI:59065	Dihydroisophorol	C9H18O	141.1285556	No
679	HMDB0030959	(Z)-3-Nonen-1-ol	C9H18O	141.1285556	No
679	HMDB0031266; CHEBI:77927	2-Nonanone	C9H18O	141.1285556	No
679	HMDB0031267	3-Nonanone	C9H18O	141.1285556	No
679	HMDB0031268	Amyl propyl ketone	C9H18O	141.1285556	No
679	HMDB0031417	2,6-Dimethyl-4-heptanone	C9H18O	141.1285556	No
679	HMDB0031684	2-Methyloctanal	C9H18O	141.1285556	No
679	HMDB0031730	3,5,5-Trimethylhexanal	C9H18O	141.1285556	No
679	HMDB0034152	1-Nonen-3-ol	C9H18O	141.1285556	No
679	HMDB0037304	(±)-2,6-Dimethyl-6-hepten-1-ol	C9H18O	141.1285556	No
679	HMDB0040581	(Z)-6-Nonen-1-ol	C9H18O	141.1285556	No
679	HMDB0041498	2-Nonen-1-ol	C9H18O	141.1285556	No
679	HMDB0041499	(Z)-2-Nonen-1-ol	C9H18O	141.1285556	No
679	HMDB0041601	(E)-3-Nonen-1-ol	C9H18O	141.1285556	No
679	HMDB0059835; CHEBI:84268	Nonanal	C9H18O	141.1285556	No
679	HMDB0059887	(E)-6-Nonenol	C9H18O	141.1285556	No
690	HMDB0000894; CHEBI:74435	Vinylacetylglycine	C6H9NO3	142.0511672	No
690	HMDB0014491; CHEBI:131804	Trimethadione	C6H9NO3	142.0511672	No
690	HMDB0032161; CHEBI:415036	Acrylamide-acrylic acid resin	C6H9NO3	142.0511672	No
690	HMDB0061082	5-ethyl-5-methyl-2,4-oxazolidinedione	C6H9NO3	142.0511672	No
690	HMDB0061705; CHEBI:87998	6-Oxopiperidine-2-carboxylic acid	C6H9NO3	142.0511672	No
692	HMDB0033115	6-Methylquinoline	C10H9N	142.066062	Yes
692	HMDB0041802; C02227; CHEBI:27878	2-Aminonaphthalene	C10H9N	142.066062	Yes
692	HMDB0042004; CHEBI:132813	Quinaldine	C10H9N	142.066062	Yes
694	HMDB0004827; C10172; CHEBI:35280	Proline betaine	C7H13NO2	142.0872731	No
694	HMDB0030410	L-2-Amino-3-methylenehexanoic acid	C7H13NO2	142.0872731	No
694	HMDB0038949	3beta,6beta-Dihydroxynortropane	C7H13NO2	142.0872731	No
703	HMDB0032429	3-(Methylthio)methylthiophene	C6H8S2	142.9987484	No
703	HMDB0034901	2-Vinyl-4H-1,3-dithiine	C6H8S2	142.9987484	No
703	HMDB0040259	2-Methyl-5-(methylthio)thiophene	C6H8S2	142.9987484	No
703	HMDB0062781; CHEBI:16810	2-oxoglutarate(2-)	C5H4O5	142.9987484	No
705	HMDB0000393; CHEBI:86952	3-Hexenedioic acid	C6H8O4	143.0349614	Yes
705	HMDB0000522; CHEBI:37245	3-Methylglutaconic acid	C6H8O4	143.0349614	Yes
705	HMDB0002266	(E)-2-Methylglutaconic acid	C6H8O4	143.0349614	Yes
705	HMDB0013311; CHEBI:49296	trans-2-Hexenedioic acid	C6H8O4	143.0349614	Yes
705	HMDB0029171	3-Hydroxyadipic acid 3,6-lactone	C6H8O4	143.0349614	Yes
705	HMDB0031257; CHEBI:35460	Dimethyl fumarate	C6H8O4	143.0349614	Yes
705	HMDB0036232; C00922; CHEBI:23812	Maleic acid homopolymer	C6H8O4	143.0349614	Yes
705	HMDB0040207	Ethyl hydrogen fumarate	C6H8O4	143.0349614	Yes
706	HMDB0041946; C19485	N-Nitrosoproline	C5H8N2O3	143.04645	No

707	HMDB0006215; CHEBI:52431	Naphthalene epoxide	C10H8O	143.0503285	No
707	HMDB0012138; C11714; CHEBI:10319	1-Naphthol	C10H8O	143.0503285	No
707	HMDB0012322; C11713; CHEBI:10432	2-Naphthol	C10H8O	143.0503285	No
707	HMDB0031001	(Z)-2-Decene-4,6,8-triyn-1-ol	C10H8O	143.0503285	No
707	HMDB0060301; C14787; CHEBI:34001	(1S,2R)-Naphthalene 1,2-oxide	C10H8O	143.0503285	No
709	HMDB0001988; CHEBI:16817	4-Hydroxycyclohexylcarboxylic acid	C7H12O3	143.0714134	Yes
709	HMDB0031176	Tetrahydrofurfuryl acetate	C7H12O3	143.0714134	Yes
709	HMDB0036594	Botryodiplodin	C7H12O3	143.0714134	Yes
709	HMDB0037114	Methyl (R)-3-methyl-2-oxopentanoate	C7H12O3	143.0714134	Yes
709	HMDB0040433	Ethyl levulinate	C7H12O3	143.0714134	Yes
711	HMDB0000482; C06423; CHEBI:28837	Caprylic acid	C8H16O2	143.1077039	No
711	HMDB0001877; C07185; CHEBI:39867	Valproic acid	C8H16O2	143.1077039	No
711	HMDB0029980; CHEBI:87510	Hexyl acetate	C8H16O2	143.1077039	No
711	HMDB0030368	(3R,5Z)-5-Octene-1,3-diol	C8H16O2	143.1077039	No
711	HMDB0031230; CHEBI:744024	2-Ethylhexanoic acid	C8H16O2	143.1077039	No
711	HMDB0031290	1-Hydroxy-3-octanone	C8H16O2	143.1077039	No
711	HMDB0031478	Methyl heptanoate	C8H16O2	143.1077039	No
711	HMDB0031579	Ethyl (±)-2-methylpentanoate	C8H16O2	143.1077039	No
711	HMDB0031587	2-Methylheptanoic acid	C8H16O2	143.1077039	No
711	HMDB0031588	6-Methylheptanoic acid	C8H16O2	143.1077039	No
711	HMDB0031596	Methyl 5-methylhexanoate	C8H16O2	143.1077039	No
711	HMDB0031638	Pentyl propanoate	C8H16O2	143.1077039	No
711	HMDB0031703	2-Ethylbutyl acetate	C8H16O2	143.1077039	No
711	HMDB0032046	Isopropyl 3-methylbutanoate	C8H16O2	143.1077039	No
711	HMDB0032047	Propyl 3-methylbutanoate	C8H16O2	143.1077039	No
711	HMDB0032203	cis- and trans-2-Isobutyl-4-methyl-1,3-dioxolane	C8H16O2	143.1077039	No
711	HMDB0032225	1,1-Dimethoxy-trans-2-hexene	C8H16O2	143.1077039	No
711	HMDB0032272	Ethyl 4-methylpentanoate	C8H16O2	143.1077039	No
711	HMDB0032335	3-Hydroxy-2-octanone	C8H16O2	143.1077039	No
711	HMDB0032551	Valeraldehyde propyleneglycol acetal	C8H16O2	143.1077039	No
711	HMDB0034161; CHEBI:87683	2-Methylpropyl butanoate	C8H16O2	143.1077039	No
711	HMDB0034164; CHEBI:87419	3-Methylbutyl propanoate	C8H16O2	143.1077039	No
711	HMDB0034234	Ethyl (±)-3-methylpentanoate	C8H16O2	143.1077039	No
711	HMDB0034459	Heptyl formate	C8H16O2	143.1077039	No
711	HMDB0035457	(±)-5-Hydroxy-4-octanone	C8H16O2	143.1077039	No
711	HMDB0036165	3-(Hydroxymethyl)-2-heptanone	C8H16O2	143.1077039	No
711	HMDB0039217	Isopropyl 2-methylbutanoate	C8H16O2	143.1077039	No
711	HMDB0039620; CHEBI:87429	Butyl butyrate	C8H16O2	143.1077039	No
711	HMDB0040209; CHEBI:86055	Ethyl hexanoate	C8H16O2	143.1077039	No
711	HMDB0040251	Butyl isobutyrate	C8H16O2	143.1077039	No
711	HMDB0040252	Isobutyl isobutyrate	C8H16O2	143.1077039	No
711	HMDB0040296	Propyl pentanoate	C8H16O2	143.1077039	No
711	HMDB0040298	Ethyl 2-ethylbutanoate	C8H16O2	143.1077039	No
711	HMDB0061931; CHEBI:88826	Tetrahydro-2,5-dimethyl-2H-pyranmethanol	C8H16O2	143.1077039	No
718	HMDB0001552; C00940; CHEBI:30882	2-Keto-glutaramic acid	C5H7NO4	144.0291855	Yes
719	HMDB0029737; C08493; CHEBI:343043	1H-Indole-3-carboxaldehyde	C9H7NO	144.0455038	Yes
721	HMDB0000730; CHEBI:70979	Isobutyrylglycine	C6H11NO3	144.066675	Yes
721	HMDB0000808; CHEBI:89963	N-Butyrylglycine	C6H11NO3	144.066675	Yes
721	HMDB0001263; C01475; CHEBI:17027	Allysine	C6H11NO3	144.066675	Yes
721	HMDB0003681; C02946; CHEBI:17645	4-Acetamidobutanoic acid	C6H11NO3	144.066675	Yes
721	HMDB0012131; C03656; CHEBI:27713	(S)-5-Amino-3-oxohexanoate	C6H11NO3	144.066675	Yes
721	HMDB0012151; C03239; CHEBI:17534	2-Keto-6-aminocaproate	C6H11NO3	144.066675	Yes
721	HMDB0015127; CHEBI:724125	Methyl aminolevulinate	C6H11NO3	144.066675	Yes
721	HMDB0029425	L-cis-4-(Hydroxymethyl)-2-pyrrolidinecarboxylic acid	C6H11NO3	144.066675	Yes
721	HMDB0029426; CHEBI:74048	L-trans-5-Hydroxy-2-piperidinecarboxylic acid	C6H11NO3	144.066675	Yes
721	HMDB0059595; C04076; CHEBI:17917	(S)-2-amino-6-oxohexanoate	C6H11NO3	144.066675	Yes
721	HMDB0060265	4-Acetamidobutanoate	C6H11NO3	144.066675	Yes
721	HMDB0061156	N-(2-Carboxymethyl)-morpholine	C6H11NO3	144.066675	Yes
721	HMDB0094698	N-Propionylalanine	C6H11NO3	144.066675	Yes
723	HMDB0006831; C05543; CHEBI:16244	3-Dehydroxycarnitine	C7H15NO2	144.1029963	No
723	HMDB0094649; CHEBI:64304	2-Aminoheptanoate	C7H15NO2	144.1029963	No

733	HMDB0000208; C00026; CHEBI:30915	Oxoglutaric acid	C5H6O5	145.0144049	Yes
733	HMDB0013701	3-Oxoglutaric acid	C5H6O5	145.0144049	Yes
733	HMDB0033966; C08369; CHEBI:4488	Di-2-propenyl disulfide, 9CI	C6H10S2	145.0144049	Yes
733	HMDB0041390	(E,E)-Di-1-propenyl disulfide	C6H10S2	145.0144049	Yes
733	HMDB0041393	(E)-1-Propenyl 2-propenyl disulfide	C6H10S2	145.0144049	Yes
733	HMDB0062739; CHEBI:87810	3-methylmalate(2-)	C5H6O5	145.0144049	Yes
734	HMDB0001218; C05851; CHEBI:28794	Coumarin	C9H6O2	145.029875	No
734	HMDB0002359	Phenylpropiolic acid	C9H6O2	145.029875	No
734	HMDB0032938; CHEBI:72013	Chromone	C9H6O2	145.029875	No
734	HMDB0033183	2-Benzofurancarboxaldehyde	C9H6O2	145.029875	No
736	HMDB0062599; CHEBI:84282	2-methyl-1,3-thiazolidine-2-carboxamide	C5H10N2OS	145.0463489	No
737	HMDB0000422; CHEBI:68567	2-Methylglutaric acid	C6H10O4	145.0508371	No
737	HMDB0000448; C06104; CHEBI:30832	Adipic acid	C6H10O4	145.0508371	No
737	HMDB0000752; CHEBI:68566	Methylglutaric acid	C6H10O4	145.0508371	No
737	HMDB0000858; CHEBI:1007322	Monomethyl glutaric acid	C6H10O4	145.0508371	No
737	HMDB0002074; CHEBI:86537	2,2-Dimethylsuccinic acid	C6H10O4	145.0508371	No
737	HMDB0002173; CHEBI:89760	Solerol	C6H10O4	145.0508371	No
737	HMDB0006900; C06006; CHEBI:27681	(S)-2-Aceto-2-hydroxybutanoic acid	C6H10O4	145.0508371	No
737	HMDB0033837	Dimethyl succinate	C6H10O4	145.0508371	No
737	HMDB0061930	4-Ethoxy-4-oxobutanoic acid	C6H10O4	145.0508371	No
738	HMDB0000641; C00064; CHEBI:18050	L-Glutamine	C5H10N2O3	145.061903	Yes
738	HMDB0002031; C05100; CHEBI:1670	Ureidoisobutyric acid	C5H10N2O3	145.061903	Yes
738	HMDB0003423; C00819; CHEBI:17061	D-Glutamine	C5H10N2O3	145.061903	Yes
738	HMDB0006899; CHEBI:185249	Alanylglycine	C5H10N2O3	145.061903	Yes
738	HMDB0028687	Alanyl-Glycine	C5H10N2O3	145.061903	Yes
740	HMDB0000182; C00047; CHEBI:18019	L-Lysine	C6H14N2O2	145.0982932	Yes
740	HMDB0003405; C00739; CHEBI:16855	D-Lysine	C6H14N2O2	145.0982932	Yes
740	HMDB0012114; C01142; CHEBI:15613	(3S)-3,6-Diaminohexanoate	C6H14N2O2	145.0982932	Yes
740	HMDB0012115; C01186; CHEBI:15616	(3S,5S)-3,5-Diaminohexanoate	C6H14N2O2	145.0982932	Yes
740	HMDB0142894	2,6-diaminohexanoic acid	C6H14N2O2	145.0982932	Yes
741	HMDB0029359	(R)-1,3-Octanediol	C8H18O2	145.1236908	No
741	HMDB0037635; CHEBI:87383	xi-1-Ethoxy-1-butoxyethane	C8H18O2	145.1236908	No
741	HMDB0038679	1-Methoxy-1-pentyloxyethane	C8H18O2	145.1236908	No
748	HMDB0034370	Ibervirin	C5H9NS2	146.0101141	No
749	HMDB0006779; C05579; CHEBI:27406	Indole-5,6-quinone	C8H5NO2	146.0249145	No
749	HMDB0030481	Agrocybin	C8H5NO2	146.0249145	No
749	HMDB0061933; C11129; CHEBI:27539	1H-Indole-2,3-dione	C8H5NO2	146.0249145	No
750	HMDB0000148; C00025; CHEBI:16015	L-Glutamic acid	C5H9NO4	146.0458988	Yes
750	HMDB0002393; C12269; CHEBI:31882	N-Methyl-D-aspartic acid	C5H9NO4	146.0458988	Yes
750	HMDB0002931; CHEBI:45441	N-Acetylserine	C5H9NO4	146.0458988	Yes
750	HMDB0003011; C00979; CHEBI:17981	O-Acetylserine	C5H9NO4	146.0458988	Yes
750	HMDB0003339; C00217; CHEBI:15966	D-Glutamic acid	C5H9NO4	146.0458988	Yes
750	HMDB0006556; C05938; CHEBI:27809	L-4-Hydroxyglutamate semialdehyde	C5H9NO4	146.0458988	Yes
750	HMDB0033550	3-(Carboxymethylamino)propanoic acid	C5H9NO4	146.0458988	Yes
750	HMDB0060475; CHEBI:18237	DL-Glutamate	C5H9NO4	146.0458988	Yes
750	HMDB0062183	N-lactoyl-Glycine	C5H9NO4	146.0458988	Yes
753	HMDB0005785; CHEBI:24814	Indole-3-carbinol	C9H9NO	146.0617282	Yes
753	HMDB0030204	1-(2-Furanylmethyl)-1H-pyrrole	C9H9NO	146.0617282	Yes
753	HMDB0040009	4-(2-Furanylmethylene)-3,4-dihydro-2H-pyrrole	C9H9NO	146.0617282	Yes
753	HMDB0040011	3-(2-Furanylmethyl)-1H-pyrrole	C9H9NO	146.0617282	Yes
754	HMDB0029449; CHEBI:435538	(2R,3R,4R)-2-Amino-4-hydroxy-3-methylpentanoic acic	C6H13NO3	146.0824666	No
754	HMDB0033453; C10144; CHEBI:4969	Fagomine	C6H13NO3	146.0824666	No
754	HMDB0061157	N-(2-Hydroxyethyl)-morpholine N-oxide	C6H13NO3	146.0824666	No
754	HMDB0142963	2-amino-6-hydroxyhexanoic acid	C6H13NO3	146.0824666	No
764	HMDB0001553; C01180; CHEBI:33574	2-Oxo-4-methylthiobutanoic acid	C5H8O3S	147.0148177	No
766	HMDB0000426; C00815; CHEBI:15584	Citramalic acid	C5H8O5	147.0300553	Yes
766	HMDB0000428	3-Hydroxyglutaric acid	C5H8O5	147.0300553	Yes
766	HMDB0000606; C01087; CHEBI:32796	D-2-Hydroxyglutaric acid	C5H8O5	147.0300553	Yes
766	HMDB0000694; C03196; CHEBI:32797	L-2-Hydroxyglutaric acid	C5H8O5	147.0300553	Yes
766	HMDB0001900; C02674; CHEBI:707706	Ribonolactone	C5H8O5	147.0300553	Yes
766	HMDB0011676; C02266; CHEBI:15867	D-Xylono-1,5-lactone	C5H8O5	147.0300553	Yes

766	HMDB0033912	2-Propenyl propyl disulfide	C6H12S2	147.0300553	Yes
766	HMDB0038890	Methyl 3-methyl-1-butenyl disulfide	C6H12S2	147.0300553	Yes
766	HMDB0041392	1-Propenyl propyl disulfide	C6H12S2	147.0300553	Yes
766	HMDB0059655; C02630; CHEBI:17084	2-Hydroxyglutarate	C5H8O5	147.0300553	Yes
767	HMDB0032332; C03124; CHEBI:52794	Hydroxylated lecithin	C4H8N2O4	147.0426907	Yes
769	HMDB0000227; C00418; CHEBI:25351	Mevalonic acid	C6H12O4	147.0663556	Yes
769	HMDB0012140; C06007; CHEBI:27512	(R) 2,3-Dihydroxy-3-methylvalerate	C6H12O4	147.0663556	Yes
769	HMDB0031639	Glycerol 1-propanoate	C6H12O4	147.0663556	Yes
769	HMDB0059629; C00418; CHEBI:17710	(R)-mevalonate	C6H12O4	147.0663556	Yes
769	HMDB0059705; CHEBI:67255	(2-Methoxyethoxy)propanoic acid	C6H12O4	147.0663556	Yes
771	HMDB0059834	Pentylbenzene	C11H16	147.1179992	No
771	HMDB0061809; CHEBI:88583	1-Methyl-4-(1-methylpropyl)-benzene	C11H16	147.1179992	No
771	HMDB0061810	2,4-Dimethyl-1-(1-methylethyl)-benzene	C11H16	147.1179992	No
780	HMDB0039817	Dihydro-4,6-dimethyl-4H-1,3,5-dithiazine	C5H11NS2	148.0261313	No
781	HMDB0004058; C05578; CHEBI:27404	5,6-Dihydroxyindole	C8H7NO2	148.0404382	Yes
781	HMDB0060318; C03742; CHEBI:16660	(S)-4-Hydroxymandelonitrile	C8H7NO2	148.0404382	Yes
781	HMDB0060946; CHEBI:29132	NAPQI	C8H7NO2	148.0404382	Yes
783	HMDB0002099; C08434; CHEBI:28871	6-Methyladenine	C6H7N5	148.0629223	No
783	HMDB0011599; C02216; CHEBI:201302	1-Methyladenine	C6H7N5	148.0629223	No
783	HMDB0011600; C00913; CHEBI:38635	3-Methyladenine	C6H7N5	148.0629223	No
783	HMDB0011614; C02241; CHEBI:201389	7-Methyladenine	C6H7N5	148.0629223	No
785	HMDB0030175	Venoterpine	C9H11NO	148.0769454	No
785	HMDB0039662	2,3,6,7-Tetrahydrocyclopent[b]azepin-8(1H)-one	C9H11NO	148.0769454	No
785	HMDB0040016	2-(2-Furanyl)-3,4,5,6-tetrahydropyridine	C9H11NO	148.0769454	No
785	HMDB0040029	2,3-Dihydro-5-methyl-1H-pyrrolizine-7-carboxaldehyde	C9H11NO	148.0769454	No
785	HMDB0040043	3-(2-Furanylmethylene)pyrrolidine	C9H11NO	148.0769454	No
785	HMDB0040047	2,3-Dihydro-6-methyl-1H-pyrrolizine-5-carboxaldehyde	C9H11NO	148.0769454	No
785	HMDB0040050	3,4-Dihydro-5-(5-methyl-2-furanyl)-2H-pyrrole	C9H11NO	148.0769454	No
785	HMDB0040051	5-Acetyl-2,3-dihydro-1H-pyrrolizine	C9H11NO	148.0769454	No
787	HMDB0014337; C07438; CHEBI:8080	Phentermine	C10H15N	148.1131355	No
787	HMDB0015517; CHEBI:6809	Methamphetamine	C10H15N	148.1131355	No
787	HMDB0032243	N,N-Dimethylphenethylamine	C10H15N	148.1131355	No
787	HMDB0032782; C11001	2,6-Diethylaniline	C10H15N	148.1131355	No
787	HMDB0034886	3-Pentylpyridine	C10H15N	148.1131355	No
787	HMDB0034893	2-Pentylpyridine	C10H15N	148.1131355	No
791	HMDB0029611; C01892; CHEBI:18091	Asparagusic acid	C4H6O2S2	148.973271	No
793	HMDB0042032; C14872; CHEBI:35012	Thiodiacetic acid	C4H6O4S	148.9911825	No
794	HMDB0029878; C02107; CHEBI:15672	D-Tartaric acid	C4H6O6	149.0094833	No
794	HMDB0038889	2-Methyl-2-(methyldithio)propanal	C5H10OS2	149.0094833	No
794	HMDB0059916; CHEBI:15674	2,3-Dihydroxybutanedioic acid	C4H6O6	149.0094833	No
798	HMDB0000098; C00181; CHEBI:53455	D-Xylose	C5H10O5	149.0456745	Yes
798	HMDB0000283; C00121; CHEBI:16988	D-Ribose	C5H10O5	149.0456745	Yes
798	HMDB0000366; CHEBI:86350	2-Deoxyribonic acid	C5H10O5	149.0456745	Yes
798	HMDB0000621; C00309; CHEBI:17173	D-Ribulose	C5H10O5	149.0456745	Yes
798	HMDB0000646; C00259; CHEBI:17535	L-Arabinose	C5H10O5	149.0456745	Yes
798	HMDB0000751; C00312; CHEBI:17399	L-Threo-2-pentulose	C5H10O5	149.0456745	Yes
798	HMDB0001644; C00310; CHEBI:17140	D-Xylulose	C5H10O5	149.0456745	Yes
798	HMDB0003371; C00310; CHEBI:16880	L-Ribulose	C5H10O5	149.0456745	Yes
798	HMDB0012194; C08353; CHEBI:27476	Beta-D-ribopyranose	C5H10O5	149.0456745	Yes
798	HMDB0012325; C06115; CHEBI:6178	Arabinofuranose	C5H10O5	149.0456745	Yes
798	HMDB0029578	Diisopropyl disulfide	C6H14S2	149.0456745	Yes
798	HMDB0029941; C01488; CHEBI:27672	D-Apiose	C5H10O5	149.0456745	Yes
798	HMDB0029942; CHEBI:46983	D-Arabinose	C5H10O5	149.0456745	Yes
798	HMDB0031161	Methyl pentyl disulfide	C6H14S2	149.0456745	Yes
798	HMDB0031472; C08373; CHEBI:45758	Dipropyl disulfide	C6H14S2	149.0456745	Yes
798	HMDB0031709	1,6-Hexanedithiol	C6H14S2	149.0456745	Yes
798	HMDB0032180	Butyl ethyl disulfide	C6H14S2	149.0456745	Yes
798	HMDB0032408	Methyl isopentyl disulfide	C6H14S2	149.0456745	Yes
798	HMDB0040056	xi-1-(Propylthio)-1-propanethiol	C6H14S2	149.0456745	Yes
798	HMDB0059753; CHEBI:89890	2-Deoxypentonic acid	C5H10O5	149.0456745	Yes
798	HMDB0060254; CHEBI:15936	Aldehydo-D-xylose	C5H10O5	149.0456745	Yes

798	HMDB0094694; CHEBI:28272	beta-Arabinose	C5H10O5	149.0456745	Yes
798	HMDB0127617	3-hydroxy-2,2-bis(hydroxymethyl)propanoic acid	C5H10O5	149.0456745	Yes
799	HMDB0000764; C05629; CHEBI:28631	Hydrocinnamic acid	C9H10O2	149.0604276	No
799	HMDB0002097; CHEBI:262555	4-Ethylbenzoic acid	C9H10O2	149.0604276	No
799	HMDB0002222; CHEBI:329287	3-Methylphenylacetic acid	C9H10O2	149.0604276	No
799	HMDB0002237; CHEBI:64818	3,4-Dimethylbenzoic acid	C9H10O2	149.0604276	No
799	HMDB0003654; C02646; CHEBI:64555	4-Coumaryl alcohol	C9H10O2	149.0604276	No
799	HMDB0011743; CHEBI:48526	2-Phenylpropionate	C9H10O2	149.0604276	No
799	HMDB0013744; C17883; CHEBI:42438	2-Methoxy-4-vinylphenol	C9H10O2	149.0604276	No
799	HMDB0031310; C15513; CHEBI:52051	Benzyl acetate	C9H10O2	149.0604276	No
799	HMDB0032073; CHEBI:389009	o-Tolyl acetate	C9H10O2	149.0604276	No
799	HMDB0032075; C01963	p-Tolyl acetate	C9H10O2	149.0604276	No
799	HMDB0032569	2''-Methoxyacetophenone	C9H10O2	149.0604276	No
799	HMDB0032570; CHEBI:86567	4''-Methoxyacetophenone	C9H10O2	149.0604276	No
799	HMDB0032592	2''-Hydroxy-5''-methylacetophenone	C9H10O2	149.0604276	No
799	HMDB0032617	Methyl phenylacetate	C9H10O2	149.0604276	No
799	HMDB0033967; C01839; CHEBI:32807	Ethyl benzoate	C9H10O2	149.0604276	No
799	HMDB0033970	4-Ethoxybenzaldehyde	C9H10O2	149.0604276	No
799	HMDB0037715	1-Phenylethyl formate	C9H10O2	149.0604276	No
799	HMDB0038355	2-Ethyl-4-(2-furanyl)-2-propenal	C9H10O2	149.0604276	No
799	HMDB0040428; CHEBI:87417	2-Phenylethyl formate	C9H10O2	149.0604276	No
799	HMDB0040925	2-(2-Furanyl)-3-methyl-2-butenal	C9H10O2	149.0604276	No
799	HMDB0059824	4-Hydroxy-3-methylacetophenone	C9H10O2	149.0604276	No
799	HMDB0062349	3''-carboxy-alpha-chromanol	C9H10O2	149.0604276	No
799	HMDB0125532	3-ethenyl-5-methoxyphenol	C9H10O2	149.0604276	No
799	HMDB0134026	3-(2-hydroxyphenyl)propanal	C9H10O2	149.0604276	No
799	HMDB0134027	2-(3-hydroxyprop-1-en-1-yl)phenol	C9H10O2	149.0604276	No
799	HMDB0135239	4-(prop-2-en-1-yl)benzene-1,2-diol	C9H10O2	149.0604276	No
799	HMDB0135276	2-hydroxy-3-phenylpropanal	C9H10O2	149.0604276	No
799	HMDB0135277	3-hydroxy-3-phenylpropanal	C9H10O2	149.0604276	No
799	HMDB0135278	3-(3-hydroxyphenyl)propanal	C9H10O2	149.0604276	No
799	HMDB0135279	3-(4-hydroxyphenyl)propanal	C9H10O2	149.0604276	No
799	HMDB0135280	3-[(1E)-3-hydroxyprop-1-en-1-yl]phenol	C9H10O2	149.0604276	No
799	HMDB0135281	(3-phenyloxiran-2-yl)methanol	C9H10O2	149.0604276	No
799	HMDB0135644	4-(3-hydroxyprop-1-en-1-yl)phenol	C9H10O2	149.0604276	No
800	HMDB0013704; CHEBI:88954	6-Methylnicotinamide	C8H10N2O	149.0718283	No
800	HMDB0029410	2-Acetyl-3-ethylpyrazine	C8H10N2O	149.0718283	No
800	HMDB0038513	7,8-Dihydro-3-methylpyrrolo[1,2-a]pyrimidin-2(6H)-on	C8H10N2O	149.0718283	No
800	HMDB0039998	2-Acetyl-3,6-dimethylpyrazine	C8H10N2O	149.0718283	No
800	HMDB0039999	2-Acetyl-3,5-dimethylpyrazine	C8H10N2O	149.0718283	No
802	HMDB0001878; C09908; CHEBI:27607	Thymol	C10H14O	149.0973127	No
802	HMDB0003647; C02576; CHEBI:15421	Perillyl aldehyde	C10H14O	149.0973127	No
802	HMDB0004487; C11383; CHEBI:15399	(+)-(S)-Carvone	C10H14O	149.0973127	No
802	HMDB0029652	2-(4-Methylphenyl)-2-propanol	C10H14O	149.0973127	No
802	HMDB0031570; CHEBI:37001	2-Methyl-1-phenyl-2-propanol	C10H14O	149.0973127	No
802	HMDB0031613	4-Phenyl-2-butanol	C10H14O	149.0973127	No
802	HMDB0031817; C06576	Cuminyl alcohol	C10H14O	149.0973127	No
802	HMDB0032063; C14200; CHEBI:34444	4-tert-Butylphenol	C10H14O	149.0973127	No
802	HMDB0032213	2-trans-4-trans-7-cis-Decatrienal	C10H14O	149.0973127	No
802	HMDB0032626	1-Methoxy-4-propylbenzene	C10H14O	149.0973127	No
802	HMDB0033544	(2E,4Z,7Z)-2,4,7-Decatrienal	C10H14O	149.0973127	No
802	HMDB0034846	2-(4-Methylphenyl)-1-propanol	C10H14O	149.0973127	No
802	HMDB0034921	Teresantalal	C10H14O	149.0973127	No
802	HMDB0035089; C01767; CHEBI:15400	(R)-Carvone	C10H14O	149.0973127	No
802	HMDB0035172	2,6,6-Trimethyl-1,4-cyclohexadiene-1-carboxaldehyde	C10H14O	149.0973127	No
802	HMDB0035250; C11939	Myrtenal	C10H14O	149.0973127	No
802	HMDB0035591	3-Caren-5-one	C10H14O	149.0973127	No
802	HMDB0035761; C09884; CHEBI:28996	(-)-Pinocarvone	C10H14O	149.0973127	No
802	HMDB0035770; C09840; CHEBI:3440	5-Isopropyl-2-methylphenol	C10H14O	149.0973127	No
802	HMDB0035824; CHEBI:38265	Carvone	C10H14O	149.0973127	No
802	HMDB0036061; C17062; CHEBI:53169	Safranal	C10H14O	149.0973127	No

802	HMDB0036070	1-Acetyl-4-isopropenylcyclopentene	C10H14O	149.0973127	No
802	HMDB0036089; C09868; CHEBI:50542	(R)-Menthofuran	C10H14O	149.0973127	No
802	HMDB0036999; C01951; CHEBI:17304	Piperitenone	C10H14O	149.0973127	No
802	HMDB0037561	Linden ether	C10H14O	149.0973127	No
802	HMDB0038913	4,6-Decadiyn-1-ol	C10H14O	149.0973127	No
802	HMDB0061788	(S)-2-methyl-5-(1-Methylethenyl)-2-cyclohexen-1-one	C10H14O	149.0973127	No
802	HMDB0133616	2-methyl-3-phenylpropan-1-ol	C10H14O	149.0973127	No
809	HMDB0034413; CHEBI:167099	1,2-Benzisothiazol-3(2H)-one	C7H5NOS	150.0021229	No
809	HMDB0040578	4-Thiocyanatophenol	C7H5NOS	150.0021229	No
814	HMDB0001859; CHEBI:46195	Acetaminophen	C8H9NO2	150.0557935	No
814	HMDB0002210; CHEBI:55484	2-Phenylglycine	C8H9NO2	150.0557935	No
814	HMDB0012219; CHEBI:74684	Dopamine quinone	C8H9NO2	150.0557935	No
814	HMDB0012992; C17756	Leukoaminochrome	C8H9NO2	150.0557935	No
814	HMDB0029703; CHEBI:1017870	Methyl 2-aminobenzoate	C8H9NO2	150.0557935	No
814	HMDB0032609; C03005; CHEBI:16394	2-(Methylamino)benzoic acid	C8H9NO2	150.0557935	No
814	HMDB0033903	3alpha,4,7,7alpha-Tetrahydro-1H-isoindole-1,3(2H)-dic	C8H9NO2	150.0557935	No
814	HMDB0059846	Ethyl nicotinate	C8H9NO2	150.0557935	No
814	HMDB0060680; C16571	2-Amino-3-methylbenzoate	C8H9NO2	150.0557935	No
814	HMDB0061919	2''-Hydroxyacetanilide	C8H9NO2	150.0557935	No
825	HMDB0015167; CHEBI:2208	Mercaptopurine	C5H4N4S	151.0074132	Yes
826	HMDB0000292; C00385; CHEBI:17712	Xanthine	C5H4N4O2	151.0263773	Yes
826	HMDB0000786; C07599; CHEBI:28315	Oxypurinol	C5H4N4O2	151.0263773	Yes
826	HMDB0001182	6,8-Dihydroxypurine	C5H4N4O2	151.0263773	Yes
827	HMDB0000020; C00642; CHEBI:18101	p-Hydroxyphenylacetic acid	C8H8O3	151.0401438	Yes
827	HMDB0000440; C05593; CHEBI:17445	3-Hydroxyphenylacetic acid	C8H8O3	151.0401438	Yes
827	HMDB0000669; C05852; CHEBI:28478	Ortho-Hydroxyphenylacetic acid	C8H8O3	151.0401438	Yes
827	HMDB0000703; C01984; CHEBI:32800	Mandelic acid	C8H8O3	151.0401438	Yes
827	HMDB0001101; C02519; CHEBI:40813	p-Anisic acid	C8H8O3	151.0401438	Yes
827	HMDB0002390; C14088; CHEBI:20141	3-Cresotinic acid	C8H8O3	151.0401438	Yes
827	HMDB0003791; C04043; CHEBI:27978	3,4-Dihydroxyphenylacetaldehyde	C8H8O3	151.0401438	Yes
827	HMDB0004815; CHEBI:85239	4-Hydroxy-3-methylbenzoic acid	C8H8O3	151.0401438	Yes
827	HMDB0012308; C00755; CHEBI:18346	Vanillin	C8H8O3	151.0401438	Yes
827	HMDB0029657; C13635; CHEBI:35164	2,4''-Dihydroxyacetophenone	C8H8O3	151.0401438	Yes
827	HMDB0029658	2'',3''-Dihydroxyacetophenone	C8H8O3	151.0401438	Yes
827	HMDB0029659; C03663; CHEBI:18414	2'',4''-Dihydroxyacetophenone	C8H8O3	151.0401438	Yes
827	HMDB0029660; CHEBI:559127	2'',6''-Dihydroxyacetophenone	C8H8O3	151.0401438	Yes
827	HMDB0029661; C10675; CHEBI:491876	3'',4''-Dihydroxyacetophenone	C8H8O3	151.0401438	Yes
827	HMDB0029662; CHEBI:574890	3'',5''-Dihydroxyacetophenone	C8H8O3	151.0401438	Yes
827	HMDB0031609; C02181; CHEBI:8075	Phenoxyacetic acid	C8H8O3	151.0401438	Yes
827	HMDB0032399	Methyl furfuracrylate	C8H8O3	151.0401438	Yes
827	HMDB0032572; CHEBI:31835	Methylparaben	C8H8O3	151.0401438	Yes
827	HMDB0032594; C10804	2-(Hydroxymethyl)benzoic acid	C8H8O3	151.0401438	Yes
827	HMDB0032604; CHEBI:421840	2-Methoxybenzoic acid	C8H8O3	151.0401438	Yes
827	HMDB0032606	3-Methoxybenzoic acid	C8H8O3	151.0401438	Yes
827	HMDB0032629	2'',5''-Dihydroxyacetophenone	C8H8O3	151.0401438	Yes
827	HMDB0032919	Ethyl 2-furanyl diketone	C8H8O3	151.0401438	Yes
827	HMDB0033003	1-(5-Methyl-2-furanyl)-1,2-propanedione	C8H8O3	151.0401438	Yes
827	HMDB0034172; C12305; CHEBI:31832	Methyl 2-hydroxybenzoate	C8H8O3	151.0401438	Yes
827	HMDB0036907	2-Propenyl 2-furancarboxylate	C8H8O3	151.0401438	Yes
827	HMDB0041283	1-(2-Furanyl)-1,3-butanedione	C8H8O3	151.0401438	Yes
827	HMDB0060390; CHEBI:31128	4-Hydroxyphenyl acetate	C8H8O3	151.0401438	Yes
827	HMDB0062635; C01983; CHEBI:17656	(R)-mandelic Acid	C8H8O3	151.0401438	Yes
827	HMDB0124926	2-hydroxy-2-phenylacetic acid	C8H8O3	151.0401438	Yes
827	HMDB0125528	5-ethenylbenzene-1,2,3-triol	C8H8O3	151.0401438	Yes
827	HMDB0137457	4-[(E)-2-hydroxyethenyl]benzene-1,2-diol	C8H8O3	151.0401438	Yes
828	HMDB0004193; C05842; CHEBI:27410	N1-Methyl-2-pyridone-5-carboxamide	C7H8N2O2	151.0512271	Yes
828	HMDB0004194; C05843; CHEBI:27838	N1-Methyl-4-pyridone-3-carboxamide	C7H8N2O2	151.0512271	Yes
830	HMDB0032132	2-Isopropyl-1,4-benzenediol	C9H12O2	151.0764748	Yes
830	HMDB0032599	3-(4-Hydroxyphenyl)-1-propanol	C9H12O2	151.0764748	Yes
830	HMDB0036169	3-(5-Methyl-2-furanyl)butanal	C9H12O2	151.0764748	Yes
830	HMDB0036227	2-Propenyl 2,4-hexadienoate	C9H12O2	151.0764748	Yes

830	HMDB0039801	1-(2-Furanyl)-1-pentanone	C9H12O2	151.0764748	Yes
830	HMDB0039956	4-(Ethoxymethyl)phenol	C9H12O2	151.0764748	Yes
830	HMDB0040175	4-Ethyl-2-methoxyphenol	C9H12O2	151.0764748	Yes
830	HMDB0040216	2,6,6-Trimethyl-2-cyclohexene-1,4-dione	C9H12O2	151.0764748	Yes
830	HMDB0040449	2-(Ethoxymethyl)phenol	C9H12O2	151.0764748	Yes
830	HMDB0041326	(Dimethoxymethyl)benzene	C9H12O2	151.0764748	Yes
830	HMDB0041804; CHEBI:38547	2-Isopropoxyphenol	C9H12O2	151.0764748	Yes
830	HMDB0059853	3,5-Dimethoxytoluene	C9H12O2	151.0764748	Yes
830	HMDB0125537	3-ethyl-5-methoxyphenol	C9H12O2	151.0764748	Yes
832	HMDB0032381	2-Methoxy-(3 or 5 or 6)-isopropylpyrazine	C8H12N2O	151.0872666	No
832	HMDB0040052	2-Isopropyl-5-methoxypyrazine	C8H12N2O	151.0872666	No
832	HMDB0040053	2-Isopropyl-6-methoxypyrazine	C8H12N2O	151.0872666	No
832	HMDB0040340	2-Isopropyl-3-methoxypyrazine	C8H12N2O	151.0872666	No
833	HMDB0003450; C00964; CHEBI:15389	(-)-trans-Carveol	C10H16O	151.1129193	No
833	HMDB0003634; C02452; CHEBI:15420	Perillyl alcohol	C10H16O	151.1129193	No
833	HMDB0003667; C02759; CHEBI:29060	Alpha-Pinene-oxide	C10H16O	151.1129193	No
833	HMDB0030892	p-Menth-3-en-9-al	C10H16O	151.1129193	No
833	HMDB0030893	Isocitral	C10H16O	151.1129193	No
833	HMDB0031854	3-Methyl-5-propyl-2-cyclohexen-1-one	C10H16O	151.1129193	No
833	HMDB0032120	2,6-Dimethyl-1,7-octadien-3-one	C10H16O	151.1129193	No
833	HMDB0032242	(E)-3,7-Dimethyl-1,5,7-octatrien-3-ol	C10H16O	151.1129193	No
833	HMDB0032536	2-(trans-2-Pentenyl)cyclopentanone	C10H16O	151.1129193	No
833	HMDB0034715	p-Mentha-1(6),8-dien-3-ol	C10H16O	151.1129193	No
833	HMDB0034916	cis-2-Thujen-4-ol	C10H16O	151.1129193	No
833	HMDB0034973; CHEBI:48697	(R)-Campholenic aldehyde	C10H16O	151.1129193	No
833	HMDB0034974	(R)-Carvotanacetone	C10H16O	151.1129193	No
833	HMDB0034975; CHEBI:48933	Piperitone	C10H16O	151.1129193	No
833	HMDB0034985; C09859; CHEBI:4999	(+)-Fenchone	C10H16O	151.1129193	No
833	HMDB0035054	(1S,4R)-p-Mentha-2,8-dien-1-ol	C10H16O	151.1129193	No
833	HMDB0035077	(1R,4R)-p-Mentha-2,8-dien-1-ol	C10H16O	151.1129193	No
833	HMDB0035078; C01499; CHEBI:16980	Geranial	C10H16O	151.1129193	No
833	HMDB0035092; C09847; CHEBI:29020	cis-Citral	C10H16O	151.1129193	No
833	HMDB0035100; C11938	2-Pinen-10-ol	C10H16O	151.1129193	No
833	HMDB0035120	Dehydrolinalool	C10H16O	151.1129193	No
833	HMDB0035125	Darwinol	C10H16O	151.1129193	No
833	HMDB0035158; C07271; CHEBI:16431	1,2-Epoxy-p-menth-8-ene	C10H16O	151.1129193	No
833	HMDB0035203	(S)-9,10-Cyclo-p-menth-1-en-4-ol	C10H16O	151.1129193	No
833	HMDB0035241	(Z)-2-Methyl-6-methylene-2,7-octadien-1-ol	C10H16O	151.1129193	No
833	HMDB0035277	(2S,4R)-p-Mentha-1(7),5-dien-2-ol	C10H16O	151.1129193	No
833	HMDB0035280	Teresantalol	C10H16O	151.1129193	No
833	HMDB0035600	(S)-Phellandral	C10H16O	151.1129193	No
833	HMDB0035604; C09893; CHEBI:26381	(R)-p-Menth-4(8)-en-3-one	C10H16O	151.1129193	No
833	HMDB0035622; C11408; CHEBI:232	(+)-cis-Carveol	C10H16O	151.1129193	No
833	HMDB0035656	3-Pinanone	C10H16O	151.1129193	No
833	HMDB0035706	alpha-Cyclocitral	C10H16O	151.1129193	No
833	HMDB0035737; CHEBI:48934	(R)-Piperitone	C10H16O	151.1129193	No
833	HMDB0035738; C09885; CHEBI:41	(S)-Piperitone	C10H16O	151.1129193	No
833	HMDB0035741; CHEBI:37046	(-)-trans-Isopulegone	C10H16O	151.1129193	No
833	HMDB0035743; C11941; CHEBI:26137	Pinocarveol	C10H16O	151.1129193	No
833	HMDB0035744	(-)-Isopinocamphone	C10H16O	151.1129193	No
833	HMDB0036044	Marmelo oxide A	C10H16O	151.1129193	No
833	HMDB0036079; C18018; CHEBI:23733	Dihydrocarvone	C10H16O	151.1129193	No
833	HMDB0036080; C11415; CHEBI:168	(1S,4S)-Dihydrocarvone	C10H16O	151.1129193	No
833	HMDB0036082; C11395; CHEBI:227	(-)-cis-Carveol	C10H16O	151.1129193	No
833	HMDB0036083; CHEBI:23046	Carveol	C10H16O	151.1129193	No
833	HMDB0036085	Dehydro-1,8-cineole	C10H16O	151.1129193	No
833	HMDB0036087	(R)-p-Mentha-1,8-dien-7-ol	C10H16O	151.1129193	No
833	HMDB0036088; C02452; CHEBI:10782	(S)-p-Mentha-1,8-dien-7-ol	C10H16O	151.1129193	No
833	HMDB0036113; C20260; CHEBI:50045	(+)-3-Thujone	C10H16O	151.1129193	No
833	HMDB0036114; CHEBI:50046	(-)-3-Thujone	C10H16O	151.1129193	No
833	HMDB0036115; C09906; CHEBI:9577	(-)-3-Isothujone	C10H16O	151.1129193	No

833	HMDB0036127	(-)-Pinocamphone	C10H16O	151.1129193	No
833	HMDB0036128	(-)-trans-Pinocarveol	C10H16O	151.1129193	No
833	HMDB0036129	Verbenol	C10H16O	151.1129193	No
833	HMDB0036598	(E,E)-2,4-Decadienal	C10H16O	151.1129193	No
833	HMDB0037008	6,8-Epoxy-p-menth-2-ene	C10H16O	151.1129193	No
833	HMDB0037009	Terpinolene oxide	C10H16O	151.1129193	No
833	HMDB0037010	(2S,4R)-p-Mentha-1(7),8-dien-2-ol	C10H16O	151.1129193	No
833	HMDB0037015	p-Mentha-1,8-dien-4-ol	C10H16O	151.1129193	No
833	HMDB0037017	(R)-p-Mentha-1,8-dien-10-ol	C10H16O	151.1129193	No
833	HMDB0037024	4-Acetyl-1,4-dimethyl-1-cyclohexene	C10H16O	151.1129193	No
833	HMDB0037025	Hop ether	C10H16O	151.1129193	No
833	HMDB0037172	Tetrahydro-5-isopropenyl-2-methyl-2-vinylfuran	C10H16O	151.1129193	No
833	HMDB0037280	Junionone	C10H16O	151.1129193	No
833	HMDB0037302	xi-p-Mentha-3,8-dien-1-ol	C10H16O	151.1129193	No
833	HMDB0037303	xi-p-Mentha-1(7),2-dien-4-ol	C10H16O	151.1129193	No
833	HMDB0038251	xi-Pinol	C10H16O	151.1129193	No
833	HMDB0038290	Photocitral A	C10H16O	151.1129193	No
833	HMDB0038557	xi-3,6-Dihydro-4-methyl-2-(2-methyl-1-propenyl)-2H-p	C10H16O	151.1129193	No
833	HMDB0039008	3,4-Epoxy-p-menth-1(7)-ene	C10H16O	151.1129193	No
833	HMDB0039816	2-Hexylfuran	C10H16O	151.1129193	No
833	HMDB0040766	Anethofuran	C10H16O	151.1129193	No
833	HMDB0041011; CHEBI:53177	beta-Cyclocitral	C10H16O	151.1129193	No
833	HMDB0041013	Isocyclocitral	C10H16O	151.1129193	No
833	HMDB0041631; CHEBI:50040	3-Thujanone	C10H16O	151.1129193	No
833	HMDB0059608; C11409; CHEBI:15388	(+)-trans-Carveol	C10H16O	151.1129193	No
833	HMDB0059761; CHEBI:89872	4-(1-Methylethyl)-1-cyclohexene-4-carboxaldehyde	C10H16O	151.1129193	No
833	HMDB0059827	3-Cyclohexene-1-carboxaldehyde, 1,3,4-trimethyl-	C10H16O	151.1129193	No
833	HMDB0059838; CHEBI:15396	Camphor	C10H16O	151.1129193	No
833	HMDB0059875	Carvotanacetone	C10H16O	151.1129193	No
833	HMDB0059885; CHEBI:87568	p-Menth-1-en-9-al	C10H16O	151.1129193	No
833	HMDB0061794; C09893; CHEBI:35596	Pulegone	C10H16O	151.1129193	No
839	HMDB0003585; C01962; CHEBI:28839	Thiocysteine	C3H7NO2S2	151.9849148	No
840	HMDB0000996; C00606; CHEBI:41721	3-Sulfinoalanine	C3H7NO4S	152.0009575	No
840	HMDB0060179; C00606; CHEBI:16345	3-Sulfinato-L-alaninate	C3H7NO4S	152.0009575	No
843	HMDB0001476; C00632; CHEBI:15793	3-Hydroxyanthranilic acid	C7H7NO3	152.0353106	Yes
843	HMDB0001972; CHEBI:1162549	3-Aminosalicylic acid	C7H7NO3	152.0353106	Yes
843	HMDB0014378; C02518; CHEBI:27565	Aminosalicylic Acid	C7H7NO3	152.0353106	Yes
843	HMDB0014389; CHEBI:6775	Mesalazine	C7H7NO3	152.0353106	Yes
843	HMDB0059749	6-Methoxy-pyridine-3-carboxylic acid	C7H7NO3	152.0353106	Yes
843	HMDB0059811	2-Nitro-p-cresol	C7H7NO3	152.0353106	Yes
848	HMDB0000073; C03758; CHEBI:18243	Dopamine	C8H11NO2	152.0714674	No
848	HMDB0004825; C04227; CHEBI:44715	p-Octopamine	C8H11NO2	152.0714674	No
848	HMDB0012309; C16666; CHEBI:46958	Vanillylamine	C8H11NO2	152.0714674	No
851	HMDB0034580; C10865	psi-Pelletierine	C9H15NO	152.1080091	No
851	HMDB0037856	2-Methyl-4-pentyloxazole	C9H15NO	152.1080091	No
851	HMDB0037857	4-Methyl-2-pentyloxazole	C9H15NO	152.1080091	No
851	HMDB0037862	5-Butyl-2-ethyloxazole	C9H15NO	152.1080091	No
851	HMDB0037872	2-Ethyl-4-methyl-5-propyloxazole	C9H15NO	152.1080091	No
851	HMDB0037873	4-Ethyl-2-methyl-5-propyloxazole	C9H15NO	152.1080091	No
851	HMDB0037874	5-Ethyl-4-methyl-2-(1-methylethyl)oxazole	C9H15NO	152.1080091	No
851	HMDB0037875	2-Butyl-4,5-dimethyloxazole	C9H15NO	152.1080091	No
851	HMDB0037876	5-Ethyl-2-methyl-4-propyloxazole	C9H15NO	152.1080091	No
851	HMDB0037878	4-Ethyl-5-methyl-2-(1-methylethyl)oxazole	C9H15NO	152.1080091	No
851	HMDB0037879	4-Butyl-2,5-dimethyloxazole	C9H15NO	152.1080091	No
851	HMDB0037880	2-Isobutyl-4,5-dimethyloxazole	C9H15NO	152.1080091	No
851	HMDB0037881	4-Ethyl-5-methyl-2-propyloxazole	C9H15NO	152.1080091	No
851	HMDB0037882	5-Butyl-2,4-dimethyloxazole	C9H15NO	152.1080091	No
860	HMDB0041079	(E)-S-1-Propenyl thiosulfate	C3H6O3S2	152.9690593	No
861	HMDB0031871	Methyl propyl trisulfide	C4H10S3	152.9862545	No
861	HMDB0032547	2,4,6-Trithiaheptane	C4H10S3	152.9862545	No
861	HMDB0033047	Ethyl (methylthio)methyl disulfide	C4H10S3	152.9862545	No

861	HMDB0039524	Diethyl trisulfide	C4H10S3	152.9862545	No
861	HMDB0040239	1,1''-Thiobisethanethiol	C4H10S3	152.9862545	No
864	HMDB0062694; CHEBI:57730	2-hydroxy-4-nitrophenolate	C6H4NO4	153.007708	No
865	HMDB0000152; C00628; CHEBI:17189	Gentisic acid	C7H6O4	153.0184507	Yes
865	HMDB0000397; C00196; CHEBI:18026	2-Pyrocatechuic acid	C7H6O4	153.0184507	Yes
865	HMDB0001856; C00230; CHEBI:36062	Protocatechuic acid	C7H6O4	153.0184507	Yes
865	HMDB0013676; CHEBI:545917	2,6-Dihydroxybenzoic acid	C7H6O4	153.0184507	Yes
865	HMDB0013677; CHEBI:258254	3,5-Dihydroxybenzoic acid	C7H6O4	153.0184507	Yes
865	HMDB0029666; CHEBI:248101	2,4-Dihydroxybenzoic acid	C7H6O4	153.0184507	Yes
865	HMDB0034299; C16748	Patulin	C7H6O4	153.0184507	Yes
865	HMDB0125090	2,4,6-trihydroxybenzaldehyde	C7H6O4	153.0184507	Yes
866	HMDB0062719	5-(2-carboxylatoethyl)-4-oxo-4,5-dihydro-1H-imidazol-	C6H6N2O3	153.0300544	Yes
867	HMDB0032935	1-(2-Thienyl)-1-butanone	C8H10OS	153.0385939	No
867	HMDB0040231	1-(5-Methyl-2-thienyl)-1-propanone	C8H10OS	153.0385939	No
867	HMDB0040589	3-Acetyl-2,5-dimethylthiophene	C8H10OS	153.0385939	No
869	HMDB0005784; CHEBI:68889	Hydroxytyrosol	C8H10O3	153.0557002	Yes
869	HMDB0030570	Sylvopinol	C8H10O3	153.0557002	Yes
869	HMDB0032012; C06317; CHEBI:18353	4-Hydroxy-3-methoxybenzenemethanol	C8H10O3	153.0557002	Yes
869	HMDB0034158; C10787; CHEBI:955	2,6-Dimethoxyphenol	C8H10O3	153.0557002	Yes
869	HMDB0037724	Propyl 2-furoate	C8H10O3	153.0557002	Yes
869	HMDB0037728	2-Furanylmethyl propanoate	C8H10O3	153.0557002	Yes
869	HMDB0059966	3,5-Dimethoxyphenol	C8H10O3	153.0557002	Yes
869	HMDB0125534	5-ethylbenzene-1,2,3-triol	C8H10O3	153.0557002	Yes
869	HMDB0126617	2,4-dimethylbenzene-1,3,5-triol	C8H10O3	153.0557002	Yes
869	HMDB0131231	5-methoxy-4-methylbenzene-1,3-diol	C8H10O3	153.0557002	Yes
869	HMDB0131232	5-methoxy-2-methylbenzene-1,3-diol	C8H10O3	153.0557002	Yes
869	HMDB0131479	2,3-dimethoxyphenol	C8H10O3	153.0557002	Yes
869	HMDB0135785	1-(4-hydroxyphenyl)ethane-1,2-diol	C8H10O3	153.0557002	Yes
872	HMDB0031302	Methyl 2-octynoate	C9H14O2	153.0920033	Yes
872	HMDB0032314	2,4-Hexadienyl propionate	C9H14O2	153.0920033	Yes
872	HMDB0032442	2-Nonenoic acid gamma-lactone	C9H14O2	153.0920033	Yes
872	HMDB0035135	(E)-2,6-Dimethyl-2,5-heptadienoic acid	C9H14O2	153.0920033	Yes
872	HMDB0036177	3,5,5-Trimethyl-1,2-cyclohexanedione	C9H14O2	153.0920033	Yes
872	HMDB0036239	Enol-3,5,5-Trimethyl-1,2-cyclohexanedione	C9H14O2	153.0920033	Yes
872	HMDB0037276	Octahydro-2H-1-benzopyran-2-one	C9H14O2	153.0920033	Yes
872	HMDB0038268	Allyl hexenoate	C9H14O2	153.0920033	Yes
872	HMDB0038697	xi-2,2,6-Trimethyl-1,4-cyclohexanedione	C9H14O2	153.0920033	Yes
872	HMDB0040285	Anapear	C9H14O2	153.0920033	Yes
872	HMDB0060285; CHEBI:58972	4-Oxo-2-nonenal	C9H14O2	153.0920033	Yes
882	HMDB0002916; C02235; CHEBI:16318	4-Nitrocatechol	C6H5NO4	154.0149184	No
882	HMDB0006200	2,4-Dihydroxy-nitrophenol	C6H5NO4	154.0149184	No
884	HMDB0041484	5-Acetyl-2,4-dimethylthiazole	C7H9NOS	154.0340684	No
888	HMDB0000177; C00135; CHEBI:15971	L-Histidine	C6H9N3O2	154.0622628	Yes
888	HMDB0034267	L-2-Amino-3-(1-pyrazolyl)propanoic acid	C6H9N3O2	154.0622628	Yes
889	HMDB0030353; C10129; CHEBI:101022	Arecoline	C8H13NO2	154.087434	No
898	HMDB0124940	2-(sulfooxy)acetic acid	C2H4O6S	154.9655016	No
899	HMDB0000816; C00988; CHEBI:17150	Phosphoglycolic acid	C2H5O6P	154.9752927	No
900	HMDB0041066	S-Propyl thiosulfate	C3H8O3S2	154.9845729	No
902	HMDB0004812; CHEBI:84212	2,5-Furandicarboxylic acid	C6H4O5	154.9988326	Yes
902	HMDB0040939	Methyl phenyl disulfide	C7H8S2	154.9988326	Yes
904	HMDB0000226; C00295; CHEBI:16742	Orotic acid	C5H4N2O4	155.0099536	No
905	HMDB0032160	3-(Acetylthio)-2-methylfuran	C7H8O2S	155.0176539	No
905	HMDB0032334	1-(3-Hydroxy-5-methyl-2-thienyl)ethanone	C7H8O2S	155.0176539	No
905	HMDB0035088	Furfuryl thioacetate	C7H8O2S	155.0176539	No
909	HMDB0029610	Ascladiol	C7H8O4	155.0349571	No
909	HMDB0059731; CHEBI:89910	2,3-Methyleneglutaric acid	C7H8O4	155.0349571	No
909	HMDB0125064	4-methoxybenzene-1,2,3-triol	C7H8O4	155.0349571	No
909	HMDB0125077	2-methoxybenzene-1,3,5-triol	C7H8O4	155.0349571	No
909	HMDB0140890	6-methoxybenzene-1,2,4-triol	C7H8O4	155.0349571	No
909	HMDB0140891	5-methoxybenzene-1,2,3-triol	C7H8O4	155.0349571	No
910	HMDB0000544	5-Hydroxymethyl-4-methyluracil	C6H8N2O3	155.0463128	Yes

910	HMDB0001014; C03680; CHEBI:27384	4-Imidazolone-5-propionic acid	C6H8N2O3	155.0463128	Yes
910	HMDB0002320; C05132; CHEBI:27487	Imidazolelactic acid	C6H8N2O3	155.0463128	Yes
913	HMDB0031101	8-Hydroxy-5,6-octadienoic acid	C8H12O3	155.0713638	Yes
913	HMDB0032232	2,5-Dimethyl-4-ethoxy-3(2H)-furanone	C8H12O3	155.0713638	Yes
915	HMDB0004362; CHEBI:58968	4-Hydroxynonenal	C9H16O2	155.1078878	Yes
915	HMDB0031263	2,4-Nonanedione	C9H16O2	155.1078878	Yes
915	HMDB0031271	2-Nonenoic acid	C9H16O2	155.1078878	Yes
915	HMDB0031514	6-Butyltetrahydro-2H-pyran-2-one	C9H16O2	155.1078878	Yes
915	HMDB0031531	Dihydro-5-pentyl-2(3H)-furanone	C9H16O2	155.1078878	Yes
915	HMDB0032265	cis-Ethyl 4-heptenoate	C9H16O2	155.1078878	Yes
915	HMDB0032307	Hept-trans-2-en-1-yl acetate	C9H16O2	155.1078878	Yes
915	HMDB0032413	Methyl 2-octenoate	C9H16O2	155.1078878	Yes
915	HMDB0032491; CHEBI:206353	Prenyl isobutyrate	C9H16O2	155.1078878	Yes
915	HMDB0033167	Ethyl cyclohexanecarboxylate	C9H16O2	155.1078878	Yes
915	HMDB0034716	4-Oxononanal	C9H16O2	155.1078878	Yes
915	HMDB0035307	Quercuslactone a	C9H16O2	155.1078878	Yes
915	HMDB0036208	2-Propenyl 2-ethylbutanoate	C9H16O2	155.1078878	Yes
915	HMDB0036222	Cyclohexyl propionate	C9H16O2	155.1078878	Yes
915	HMDB0037023	2-Hydroxy-2,6,6-trimethylcyclohexanone	C9H16O2	155.1078878	Yes
915	HMDB0037496	Methyl (E)-2-octenoate	C9H16O2	155.1078878	Yes
915	HMDB0037618	(E)-3-Heptenyl acetate	C9H16O2	155.1078878	Yes
915	HMDB0037629	Isobutyl angelate	C9H16O2	155.1078878	Yes
915	HMDB0038272; CHEBI:87682	2-Hexenyl propionate	C9H16O2	155.1078878	Yes
915	HMDB0038275	cis-3-Hexenyl propanoate	C9H16O2	155.1078878	Yes
915	HMDB0039736	6-Hydroxy-4,6-dimethyl-3-hepten-2-one	C9H16O2	155.1078878	Yes
915	HMDB0039794	Methyl 4Z-octenoate	C9H16O2	155.1078878	Yes
915	HMDB0040210	2-Propenyl hexanoate	C9H16O2	155.1078878	Yes
915	HMDB0059728	Methyl 3-Octenoate	C9H16O2	155.1078878	Yes
915	HMDB0059817	(Z)-Oak lactone	C9H16O2	155.1078878	Yes
915	HMDB0059840	(E)-2-Ethyl heptenoate	C9H16O2	155.1078878	Yes
915	HMDB0059894	(E)-Oak lactone	C9H16O2	155.1078878	Yes
915	HMDB0060286	3,4-Epoxynonanal	C9H16O2	155.1078878	Yes
928	HMDB0000459; CHEBI:68499	3-Methylcrotonylglycine	C7H11NO3	156.0668001	Yes
928	HMDB0000959; CHEBI:73018	Tiglylglycine	C7H11NO3	156.0668001	Yes
928	HMDB0014755; C07411; CHEBI:250989	Paramethadione	C7H11NO3	156.0668001	Yes
928	HMDB0094701; CHEBI:21560	N-Acetylproline	C7H11NO3	156.0668001	Yes
931	HMDB0014447; C12535; CHEBI:48669	Tranexamic Acid	C8H15NO2	156.1031327	No
931	HMDB0033433; C08283	(S)-Homostachydrine	C8H15NO2	156.1031327	No
935	HMDB0041065	Dimethyl tetrasulfide	C2H6S4	156.926051	No
941	HMDB0031210	Zymonic acid	C6H6O5	157.0150889	No
941	HMDB0037759; C06035	2-Methylene-4-oxopentanedioic acid	C6H6O5	157.0150889	No
941	HMDB0060348; CHEBI:1184	2-Maleylacetate	C6H6O5	157.0150889	No
941	HMDB0137818	benzene-1,2,3,4,5-pentol	C6H6O5	157.0150889	No
942	HMDB0000462; C01551; CHEBI:15676	Allantoin	C4H6N4O3	157.0370714	No
943	HMDB0000635; CHEBI:87897	Succinylacetone	C7H10O4	157.050688	No
943	HMDB0012241; C02631; CHEBI:17275	Isopropylmaleate	C7H10O4	157.050688	No
943	HMDB0036380	xi-2,3-Dihydro-3,5-dihydroxy-6-methyl-4H-pyran-4-one	C7H10O4	157.050688	No
943	HMDB0059815	Ethyl 5-oxotetrahydro-2-furancarboxylate	C7H10O4	157.050688	No
945	HMDB0031670	1-(Hydroxymethyl)-5,5-dimethyl-2,4-imidazolidinedion	C6H10N2O3	157.0615015	No
946	HMDB0032861	2-Methoxynaphthalene	C11H10O	157.0655256	No
946	HMDB0060303; CHEBI:27615	(2-Naphthyl)methanol	C11H10O	157.0655256	No
946	HMDB0060322	1-Hydroxymethylnaphthalene	C11H10O	157.0655256	No
949	HMDB0000451; CHEBI:89344	cis-4-Hydroxycyclohexylacetic acid	C8H14O3	157.0870153	No
949	HMDB0000909; CHEBI:88508	trans-4-Hydroxycyclohexylacetic acid	C8H14O3	157.0870153	No
949	HMDB0010721; CHEBI:44680	3-Oxooctanoic acid	C8H14O3	157.0870153	No
949	HMDB0013211	Alpha-Ketooctanoic acid	C8H14O3	157.0870153	No
949	HMDB0030303	6-Ethyl-1-methyl-2,7,8-trioxabicyclo[3.2.1]octane	C8H14O3	157.0870153	No
949	HMDB0031177	Tetrahydrofurfuryl propionate	C8H14O3	157.0870153	No
949	HMDB0031307; C02975; CHEBI:18119	Ethyl 3-oxohexanoate	C8H14O3	157.0870153	No
949	HMDB0036230	1-Methyl-2-oxopropyl butyrate	C8H14O3	157.0870153	No
949	HMDB0036395	2-Methylpropyl 3-oxobutanoate	C8H14O3	157.0870153	No

949	HMDB0038305	3-Methylbutyl 2-oxopropanoate	C8H14O3	157.0870153	No
949	HMDB0040447	Butyl acetoacetate	C8H14O3	157.0870153	No
949	HMDB0041616	Propyl levulinate	C8H14O3	157.0870153	No
949	HMDB0059938	5-Butyl-1,4-dioxan-2-one	C8H14O3	157.0870153	No
949	HMDB0059939	6-Butyl-1,4-dioxan-2-one	C8H14O3	157.0870153	No
949	HMDB0060683; C16655	2-n-Propyl-4-oxopentanoic acid	C8H14O3	157.0870153	No
949	HMDB0060685; C16652; CHEBI:80638	3-Oxovalproic acid	C8H14O3	157.0870153	No
949	HMDB0062788; CHEBI:84261	Isobutyric Acid Anhydride	C8H14O3	157.0870153	No
950	HMDB0032178; C19575; CHEBI:53405	Butadiene-styrene rubber	C12H14	157.1018603	No
951	HMDB0000798; CHEBI:86618	Oenanthic ether	C9H18O2	157.1234425	No
951	HMDB0000847; C01601; CHEBI:29019	Pelargonic acid	C9H18O2	157.1234425	No
951	HMDB0030331	xi-2-Ethylheptanoic acid	C9H18O2	157.1234425	No
951	HMDB0031291; CHEBI:87432	Methyl caprylate	C9H18O2	157.1234425	No
951	HMDB0031480	Heptyl acetate	C9H18O2	157.1234425	No
951	HMDB0031557	4-Methyloctanoic acid	C9H18O2	157.1234425	No
951	HMDB0032042	Butyl 3-methylbutanoate	C9H18O2	157.1234425	No
951	HMDB0032309	3-Heptyl acetate	C9H18O2	157.1234425	No
951	HMDB0032406	5-Methylhexyl acetate	C9H18O2	157.1234425	No
951	HMDB0032454	3-Octyl formate	C9H18O2	157.1234425	No
951	HMDB0034162	Pentyl butanoate	C9H18O2	157.1234425	No
951	HMDB0034165; CHEBI:87365	Propyl hexanoate	C9H18O2	157.1234425	No
951	HMDB0034606; CHEBI:87549	Hexyl propionate	C9H18O2	157.1234425	No
951	HMDB0036234; CHEBI:87537	3-Methylbutyl 2-methylpropanoate	C9H18O2	157.1234425	No
951	HMDB0037143	1-Hydroxy-3-nonanone	C9H18O2	157.1234425	No
951	HMDB0037164	3-(Hydroxymethyl)-2-octanone	C9H18O2	157.1234425	No
951	HMDB0037288	4-Methyl-2-pentyl-1,3-dioxolane	C9H18O2	157.1234425	No
951	HMDB0038040	2-Methylpropyl 3-methylbutanoate	C9H18O2	157.1234425	No
951	HMDB0038603; CHEBI:87496	Octyl formate	C9H18O2	157.1234425	No
951	HMDB0039216	Butyl 2-methylbutanoate	C9H18O2	157.1234425	No
951	HMDB0039219	1-Methylbutyl butanoate	C9H18O2	157.1234425	No
951	HMDB0040221; CHEBI:87422	Isoamyl butyrate	C9H18O2	157.1234425	No
951	HMDB0040295	Butyl pentanoate	C9H18O2	157.1234425	No
951	HMDB0040430	Isopropyl hexanoate	C9H18O2	157.1234425	No
951	HMDB0059822	Ethyl 5-methylhexanoate	C9H18O2	157.1234425	No
965	HMDB0029433; C00651; CHEBI:48029	L-2-Amino-4-methylenepentanedioic acid	C6H9NO4	158.0460434	Yes
965	HMDB0034442	L-trans-alpha-Amino-2-carboxycyclopropaneacetic acic	C6H9NO4	158.0460434	Yes
970	HMDB0000339; CHEBI:240943	2-Methylbutyrylglycine	C7H13NO3	158.0824781	Yes
970	HMDB0000678; CHEBI:70984	Isovalerylglycine	C7H13NO3	158.0824781	Yes
970	HMDB0000927	Valerylglycine	C7H13NO3	158.0824781	Yes
970	HMDB0011757	N-Acetylvaline	C7H13NO3	158.0824781	Yes
970	HMDB0012154; C02636; CHEBI:57885	3-Dehydrocarnitine	C7H13NO3	158.0824781	Yes
970	HMDB0012175; C03087	5-Acetamidovalerate	C7H13NO3	158.0824781	Yes
970	HMDB0029230	4-Hydroxystachydrine	C7H13NO3	158.0824781	Yes
970	HMDB0029409; CHEBI:85534	Turicine	C7H13NO3	158.0824781	Yes
970	HMDB0029412; C08269	Betonicine	C7H13NO3	158.0824781	Yes
970	HMDB0031345	Calystegine A6	C7H13NO3	158.0824781	Yes
970	HMDB0036384	Calystegine A7	C7H13NO3	158.0824781	Yes
970	HMDB0038593	Calystegin A3	C7H13NO3	158.0824781	Yes
970	HMDB0038625	Medicanine	C7H13NO3	158.0824781	Yes
970	HMDB0061678; CHEBI:70830	Methyl 5-(hydroxymethyl)pyrrolidine-3-carboxylate	C7H13NO3	158.0824781	Yes
972	HMDB0000991; CHEBI:75145	DL-2-Aminooctanoic acid	C8H17NO2	158.1187401	No
972	HMDB0014375; CHEBI:64356	Pregabalin	C8H17NO2	158.1187401	No
981	HMDB0035901	Thiacremonone	C6H8O3S	159.0117358	No
983	HMDB0000225; C00322; CHEBI:15753	Oxoadipic acid	C6H8O5	159.0304985	Yes
983	HMDB0000398; C00846; CHEBI:37440	3-Oxoadipic acid	C6H8O5	159.0304985	Yes
983	HMDB0039447	2-Methyl-4-oxopentanedioic acid	C6H8O5	159.0304985	Yes
983	HMDB0059737; CHEBI:17325	3-Methyl-3-hydroxypentanedioate	C6H8O5	159.0304985	Yes
984	HMDB0030412; C08298; CHEBI:529821	Tricholomic acid	C5H8N2O4	159.0414001	No
984	HMDB0042037; CHEBI:29128	Thymine glycol	C5H8N2O4	159.0414001	No
985	HMDB0032394; CHEBI:563586	6-Methylcoumarin	C10H8O2	159.0453046	No
985	HMDB0036073	Viburtinal	C10H8O2	159.0453046	No

985	HMDB0041009	7-Methyl-2-benzofurancarboxaldehyde	C10H8O2	159.0453046	No
985	HMDB0060497; CHEBI:17435	Naphthalene-1,2-diol	C10H8O2	159.0453046	No
986	HMDB0000555	3-Methyladipic acid	C7H12O4	159.0662726	Yes
986	HMDB0000857; C02656; CHEBI:30531	Pimelic acid	C7H12O4	159.0662726	Yes
986	HMDB0002441; CHEBI:1129201	3,3-Dimethylglutaric acid	C7H12O4	159.0662726	Yes
986	HMDB0029167	2-Methyladipic acid	C7H12O4	159.0662726	Yes
986	HMDB0029573; CHEBI:391281	Diethyl malonate	C7H12O4	159.0662726	Yes
986	HMDB0041605	Propyleneglycol diacetate	C7H12O4	159.0662726	Yes
986	HMDB0059722; CHEBI:70855	Mono-methyl-adipate	C7H12O4	159.0662726	Yes
986	HMDB0059738	2-Ethylglutaric acid	C7H12O4	159.0662726	Yes
986	HMDB0059893	Ethyl methyl_succinate	C7H12O4	159.0662726	Yes
986	HMDB0061676; CHEBI:68459	2,2-Dimethylglutaric acid	C7H12O4	159.0662726	Yes
987	HMDB0031572; CHEBI:87335	2-Methyl-4-propyl-1,3-oxathiane	C8H16OS	159.0850371	No
987	HMDB0032426	(+/-)-3-(Methylthio)heptanal	C8H16OS	159.0850371	No
988	HMDB0000486	7-Hydroxyoctanoic acid	C8H16O3	159.1027358	No
988	HMDB0000711; CHEBI:86543	Hydroxyoctanoic acid	C8H16O3	159.1027358	No
988	HMDB0001954; CHEBI:37098	3-Hydroxyoctanoic acid	C8H16O3	159.1027358	No
988	HMDB0002264	(R)-2-Hydroxycaprylic acid	C8H16O3	159.1027358	No
988	HMDB0010722; CHEBI:37099	(R)-3-Hydroxyoctanoic acid	C8H16O3	159.1027358	No
988	HMDB0013898; C16650	5-Hydroxyvalproic acid	C8H16O3	159.1027358	No
988	HMDB0013899; C16651	3-Hydroxyvalproic acid	C8H16O3	159.1027358	No
988	HMDB0013900; C16649	4-Hydroxyvalproic acid	C8H16O3	159.1027358	No
988	HMDB0031509; CHEBI:23997	Ethyl (±)-3-hydroxyhexanoate	C8H16O3	159.1027358	No
988	HMDB0032270	(+/-)-Ethyl 2-hydroxy-3-methylvalerate	C8H16O3	159.1027358	No
988	HMDB0032827	Peroxyoctanoic acid	C8H16O3	159.1027358	No
988	HMDB0059825; CHEBI:87534	Isoamyl lactate	C8H16O3	159.1027358	No
988	HMDB0059867	Ethyl 2-hydroxy-4-methylpentanoate	C8H16O3	159.1027358	No
988	HMDB0062596; CHEBI:78949	(7R)-7-hydroxyoctanoic Acid	C8H16O3	159.1027358	No
989	HMDB0002038; C02728; CHEBI:17604	N(6)-Methyllysine	C7H16N2O2	159.1140004	Yes
989	HMDB0006009	Isoputreanine	C7H16N2O2	159.1140004	Yes
990	HMDB0061813	1-(1-Methylethenyl)-4-(1-methylethyl)benzene	C12H16	159.118041	No
990	HMDB0061814	1-(1-methylethenyl)-3-(1-methylethyl)-benzene	C12H16	159.118041	No
998	HMDB0002273; C03079; CHEBI:16338	4-Hydroxy-L-glutamic acid	C5H7NO5	160.0258281	No
998	HMDB0002467	A-Ketoglutaric acid oxime	C5H7NO5	160.0258281	No
998	HMDB0033764	Erucin	C6H11NS2	160.0258281	No
998	HMDB0038446	1-Thiocyanato-4-(methylthio)butane	C6H11NS2	160.0258281	No
998	HMDB0060495; C01044; CHEBI:48429	N-Formyl-L-aspartate	C5H7NO5	160.0258281	No
999	HMDB0002285; CHEBI:130363	2-Indolecarboxylic acid	C9H7NO2	160.0402492	Yes
999	HMDB0003320; C19837; CHEBI:24809	Indole-3-carboxylic acid	C9H7NO2	160.0402492	Yes
999	HMDB0004077; C05639; CHEBI:28799	4,6-Dihydroxyquinoline	C9H7NO2	160.0402492	Yes
999	HMDB0031172; CHEBI:563251	3-Formyl-6-hydroxyindole	C9H7NO2	160.0402492	Yes
999	HMDB0060289; CHEBI:28883	Quinoline-4,8-diol	C9H7NO2	160.0402492	Yes
1000	HMDB0000510; C00956; CHEBI:37024	Aminoadipic acid	C6H11NO4	160.061571	Yes
1000	HMDB0029423; CHEBI:7671	Acetylhomoserine	C6H11NO4	160.061571	Yes
1000	HMDB0033747; C03210; CHEBI:857	(±)-2,2''-Iminobispropanoic acid	C6H11NO4	160.061571	Yes
1000	HMDB0061715	Glutamate, gamma-methyl ester	C6H11NO4	160.061571	Yes
1000	HMDB0062557; CHEBI:45826	N-Acetylthreonine	C6H11NO4	160.061571	Yes
1000	HMDB0062660; C01046; CHEBI:16440	N-methyl-L-glutamic Acid	C6H11NO4	160.061571	Yes
1000	HMDB0094716	hydroxybutyrylglycine	C6H11NO4	160.061571	Yes
1001	HMDB0003447; C00955; CHEBI:17890	Tryptophanol	C10H11NO	160.0767608	Yes
1001	HMDB0030267; C09915; CHEBI:3157	(R)-Boschniakine	C10H11NO	160.0767608	Yes
1001	HMDB0040028; CHEBI:134433	1-(2,3-Dihydro-1H-pyrrolizin-5-yl)-2-propen-1-one	C10H11NO	160.0767608	Yes
1001	HMDB0040042; CHEBI:134432	3-[(5-Methyl-2-furanyl)methyl]-1H-pyrrole	C10H11NO	160.0767608	Yes
1001	HMDB0040048; CHEBI:134434	3,4-Dihydro-4-[(5-methyl-2-furanyl)methylene]-2H-pyr	C10H11NO	160.0767608	Yes
1003	HMDB0000062; C00318; CHEBI:11060	L-Carnitine	C7H15NO3	160.0979234	No
1003	HMDB0062496; CHEBI:17126	Malonyl-Carnitin	C7H15NO3	160.0979234	No
1005	HMDB0006078; CHEBI:89478	Putreanine	C7H17N2O2	160.1212858	No
1005	HMDB0015154; C06850; CHEBI:3084	Bethanechol	C7H17N2O2	160.1212858	No
1018	HMDB0012134; C15606; CHEBI:49252	1,2-Dihydroxy-3-keto-5-methylthiopentene	C6H10O3S	161.0290006	Yes
1018	HMDB0036204	1-(Methylsulfanyl)-1-oxopropan-2-yl acetate	C6H10O3S	161.0290006	Yes
1019	HMDB0000321; C02360; CHEBI:17023	2-Hydroxyadipic acid	C6H10O5	161.0456518	Yes

1019	HMDB0000345	3-Hydroxyadipic acid	C6H10O5	161.0456518	Yes
1019	HMDB0000355; C03761; CHEBI:16831	3-Hydroxymethylglutaric acid	C6H10O5	161.0456518	Yes
1019	HMDB0000368; CHEBI:55541	2(R)-Hydroxyadipic acid	C6H10O5	161.0456518	Yes
1019	HMDB0000640; CHEBI:30997	Levoglucosan	C6H10O5	161.0456518	Yes
1019	HMDB0029934	D-1-Deoxy-erythro-hexo-2,3-diulose	C6H10O5	161.0456518	Yes
1019	HMDB0032873; C11592; CHEBI:59051	Diethyl dicarbonate	C6H10O5	161.0456518	Yes
1019	HMDB0034426	(-)-1-Methylpropyl 1-propenyl disulfide	C7H14S2	161.0456518	Yes
1019	HMDB0041561	D-1,5-Anhydrofructose	C6H10O5	161.0456518	Yes
1019	HMDB0059758; C02488	2-Hydroxy-2-ethylsuccinic acid	C6H10O5	161.0456518	Yes
1020	HMDB0029714	4,5-Dihydro-1-benzoxepin-3(2H)-one	C10H10O2	161.0592347	Yes
1020	HMDB0032611; C10475	3-(4-Methoxyphenyl)-2-propenal	C10H10O2	161.0592347	Yes
1020	HMDB0033591; C10490	Safrole	C10H10O2	161.0592347	Yes
1020	HMDB0033830	Cassiastearoptene	C10H10O2	161.0592347	Yes
1020	HMDB0033833	Methyl cinnamate	C10H10O2	161.0592347	Yes
1020	HMDB0038893	3,6-Dimethyl-2(3H)-benzofuranone	C10H10O2	161.0592347	Yes
1020	HMDB0040577	Cinnamyl formate	C10H10O2	161.0592347	Yes
1020	HMDB0040592	Allyl benzoate	C10H10O2	161.0592347	Yes
1020	HMDB0060335; CHEBI:28516	1,2-Dihydronaphthalene-1,2-diol	C10H10O2	161.0592347	Yes
1020	HMDB0133504	4-(3-hydroxyphenyl)but-3-en-2-one	C10H10O2	161.0592347	Yes
1020	HMDB0133594	(2E)-2-methyl-3-phenylprop-2-enoic acid	C10H10O2	161.0592347	Yes
1020	HMDB0133595	(2Z)-2-(hydroxymethyl)-3-phenylprop-2-enal	C10H10O2	161.0592347	Yes
1020	HMDB0133596	(2Z)-3-(3-hydroxyphenyl)-2-methylprop-2-enal	C10H10O2	161.0592347	Yes
1020	HMDB0133597	(2Z)-3-(4-hydroxyphenyl)-2-methylprop-2-enal	C10H10O2	161.0592347	Yes
1020	HMDB0133598	2-methyl-3-phenyloxirane-2-carbaldehyde	C10H10O2	161.0592347	Yes
1020	HMDB0133599	(2Z)-3-(2-hydroxyphenyl)-2-methylprop-2-enal	C10H10O2	161.0592347	Yes
1020	HMDB0133747	(3E)-1-hydroxy-4-phenylbut-3-en-2-one	C10H10O2	161.0592347	Yes
1020	HMDB0133748	(3E)-4-(3-hydroxyphenyl)but-3-en-2-one	C10H10O2	161.0592347	Yes
1020	HMDB0133749	(3E)-4-(4-hydroxyphenyl)but-3-en-2-one	C10H10O2	161.0592347	Yes
1020	HMDB0133750	1-(3-phenyloxiran-2-yl)ethan-1-one	C10H10O2	161.0592347	Yes
1020	HMDB0133751	(3E)-4-(2-hydroxyphenyl)but-3-en-2-one	C10H10O2	161.0592347	Yes
1020	HMDB0133752	(3Z)-3-hydroxy-4-phenylbut-3-en-2-one	C10H10O2	161.0592347	Yes
1020	HMDB0133755	1-phenylbutane-2,3-dione	C10H10O2	161.0592347	Yes
1020	HMDB0135643	3-(4-methoxyphenyl)prop-2-enal	C10H10O2	161.0592347	Yes
1020	HMDB0141046	4-(4-hydroxyphenyl)but-3-en-2-one	C10H10O2	161.0592347	Yes
1020	HMDB0141158	1-hydroxy-4-phenylbut-3-en-2-one	C10H10O2	161.0592347	Yes
1021	HMDB0001297; CHEBI:89406	Norcotinine	C9H10N2O	161.0717116	No
1022	HMDB0059918	2-(1-Ethoxyethoxy)propanoic acid	C7H14O4	161.0818906	Yes
1023	HMDB0000450; C16741; CHEBI:18040	5-Hydroxylysine	C6H14N2O3	161.0932471	No
1024	HMDB0001934; C00745; CHEBI:17688	L(-)-Nicotine	C10H14N2	161.1079084	No
1024	HMDB0004350; C06180; CHEBI:28986	Anabasine	C10H14N2	161.1079084	No
1024	HMDB0014330; C16150; CHEBI:18723	Nicotine	C10H14N2	161.1079084	No
1024	HMDB0039982	Trimethyl-2-propenylpyrazine	C10H14N2	161.1079084	No
1026	HMDB0013806	1,3-Diisopropylbenzene	C12H18	161.1336889	No
1026	HMDB0036759	Pregeijerene	C12H18	161.1336889	No
1026	HMDB0061815	Hexylbenzene	C12H18	161.1336889	No
1034	HMDB0033585; CHEBI:83501	Acesulfame	C4H5NO4S	161.9867454	No
1036	HMDB0001890; C06809; CHEBI:28939	Acetylcysteine	C5H9NO3S	162.0239964	Yes
1037	HMDB0038445; CHEBI:351346	1-Isothiocyanato-2-phenylethane	C9H9NS	162.0359729	No
1037	HMDB0040034	2-Ethylbenzothiazole	C9H9NS	162.0359729	No
1039	HMDB0001424	4-(3-Pyridyl)-3-butenoic acid	C9H9NO2	162.0560012	Yes
1039	HMDB0004186; C05834; CHEBI:28254	3-Methyldioxyindole	C9H9NO2	162.0560012	Yes
1039	HMDB0062406; C19567; CHEBI:66879	4-Oxo-1-(3-pyridyl)-1-butanone	C9H9NO2	162.0560012	Yes
1042	HMDB0011727; CHEBI:39066	Bicine	C6H13NO4	162.0763519	No
1042	HMDB0032862	2-Amino-4-ethoxy-3-hydroxybutanoic acid	C6H13NO4	162.0763519	No
1042	HMDB0035359; C16843	1-Deoxynojirimycin	C6H13NO4	162.0763519	No
1057	HMDB0000205; C00166; CHEBI:30851	Phenylpyruvic acid	C9H8O3	163.0399119	No
1057	HMDB0001713; C12621; CHEBI:32357	m-Coumaric acid	C9H8O3	163.0399119	No
1057	HMDB0002035; C00811; CHEBI:32374	4-Hydroxycinnamic acid	C9H8O3	163.0399119	No
1057	HMDB0002641; C01772; CHEBI:18125	2-Hydroxycinnamic acid	C9H8O3	163.0399119	No
1057	HMDB0012225; C02763; CHEBI:16815	Enol-phenylpyruvate	C9H8O3	163.0399119	No
1057	HMDB0030677; C06738; CHEBI:17450	cis-p-Coumaric acid	C9H8O3	163.0399119	No

1057	HMDB0041592; C05838; CHEBI:28873	Coumaric acid	C9H8O3	163.0399119	No
1057	HMDB0062605; CHEBI:84256	Methyl Phenylglyoxalate	C9H8O3	163.0399119	No
1057	HMDB0125104	3-(3-hydroxyphenyl)prop-2-enoic acid	C9H8O3	163.0399119	No
1057	HMDB0126545	3-phenyloxirane-2-carboxylic acid	C9H8O3	163.0399119	No
1057	HMDB0128076	3-(4-hydroxyphenyl)prop-2-enoic acid	C9H8O3	163.0399119	No
1057	HMDB0129375	4-methoxy-1-benzofuran-6-ol	C9H8O3	163.0399119	No
1057	HMDB0134028	3-(2-hydroxyphenyl)prop-2-enoic acid	C9H8O3	163.0399119	No
1057	HMDB0134029	3-(2,3-dihydroxyphenyl)prop-2-enal	C9H8O3	163.0399119	No
1057	HMDB0134030	3-(2,5-dihydroxyphenyl)prop-2-enal	C9H8O3	163.0399119	No
1057	HMDB0134031	3-(2-hydroxyphenyl)oxirane-2-carbaldehyde	C9H8O3	163.0399119	No
1057	HMDB0141767	(2E)-3-(3,4-dihydroxyphenyl)prop-2-enal	C9H8O3	163.0399119	No
1057	HMDB0141768	3-(4-hydroxyphenyl)oxirane-2-carbaldehyde	C9H8O3	163.0399119	No
1059	HMDB0000174; C01019; CHEBI:2181	L-Fucose	C6H12O5	163.0612871	Yes
1059	HMDB0000849; C00507; CHEBI:62346	Rhamnose	C6H12O5	163.0612871	Yes
1059	HMDB0002712; C07326; CHEBI:16070	1,5-Anhydrosorbitol	C6H12O5	163.0612871	Yes
1059	HMDB0003081; C02095; CHEBI:27442	Beta-D-Fucose	C6H12O5	163.0612871	Yes
1059	HMDB0005876	3-Deoxyfructose	C6H12O5	163.0612871	Yes
1059	HMDB0010207; C00861; CHEBI:17897	L-Rhamnulose	C6H12O5	163.0612871	Yes
1059	HMDB0012327; C02781	2-Deoxygalactopyranose	C6H12O5	163.0612871	Yes
1059	HMDB0029196; C01018; CHEBI:42564	D-Fucose	C6H12O5	163.0612871	Yes
1059	HMDB0033050	Butyl isopropyl disulfide	C7H16S2	163.0612871	Yes
1059	HMDB0033051	Butyl propyl disulfide	C7H16S2	163.0612871	Yes
1059	HMDB0033821	2-O-Methyl-D-xylose	C6H12O5	163.0612871	Yes
1059	HMDB0039783	1-(Methylthio)-1-hexanethiol	C7H16S2	163.0612871	Yes
1059	HMDB0059624; CHEBI:42548	Alpha-L-fucose	C6H12O5	163.0612871	Yes
1059	HMDB0059625; CHEBI:42589	beta-L-fucose	C6H12O5	163.0612871	Yes
1059	HMDB0060267; C01721; CHEBI:17617	L-Fuculose	C6H12O5	163.0612871	Yes
1059	HMDB0062477	2-Deoxyglucose	C6H12O5	163.0612871	Yes
1059	HMDB0062489; CHEBI:48204	L-Fucose-1P	C6H12O5	163.0612871	Yes
1059	HMDB0124735	6-methyloxane-2,3,4,5-tetrol	C6H12O5	163.0612871	Yes
1060	HMDB0000543; CHEBI:41500	Benzenebutanoic acid	C10H12O2	163.0766013	No
1060	HMDB0001955	3-Phenylbutyric acid	C10H12O2	163.0766013	No
1060	HMDB0005802; C10469; CHEBI:50545	Isoeugenol	C10H12O2	163.0766013	No
1060	HMDB0005809; C10453; CHEBI:4917	Eugenol	C10H12O2	163.0766013	No
1060	HMDB0030060	Methyl 3-phenylpropanoate	C10H12O2	163.0766013	No
1060	HMDB0030063	Benzyl propionate	C10H12O2	163.0766013	No
1060	HMDB0031761	Propyl benzoate	C10H12O2	163.0766013	No
1060	HMDB0032038	Isopropyl benzoate	C10H12O2	163.0766013	No
1060	HMDB0032336	3-Hydroxy-4-phenylbutan-2-one	C10H12O2	163.0766013	No
1060	HMDB0032586	3-(4-Methoxyphenyl)-2-propen-1-ol	C10H12O2	163.0766013	No
1060	HMDB0032618	Ethyl phenylacetate	C10H12O2	163.0766013	No
1060	HMDB0032620	1-Phenylethyl acetate	C10H12O2	163.0766013	No
1060	HMDB0032891	1-(4-Methoxyphenyl)-2-propanone	C10H12O2	163.0766013	No
1060	HMDB0033723; CHEBI:68656	4-(4-Hydroxyphenyl)-2-butanone	C10H12O2	163.0766013	No
1060	HMDB0033945; C12303; CHEBI:31988	2-Phenylethyl acetate	C10H12O2	163.0766013	No
1060	HMDB0034471	3-Phenylpropyl formate	C10H12O2	163.0766013	No
1060	HMDB0034615	Epoxyanethole	C10H12O2	163.0766013	No
1060	HMDB0034732	Thymoquinone	C10H12O2	163.0766013	No
1060	HMDB0035213; C09904; CHEBI:10447	beta-Thujaplicin	C10H12O2	163.0766013	No
1060	HMDB0035268; C06578; CHEBI:28122	4-Isopropylbenzoic acid	C10H12O2	163.0766013	No
1060	HMDB0035414	Lepalone	C10H12O2	163.0766013	No
1060	HMDB0036098	Isoegomaketone	C10H12O2	163.0766013	No
1060	HMDB0037160	4-Methyl-2-phenyl-1,3-dioxolane	C10H12O2	163.0766013	No
1060	HMDB0037174	p-Mentha-2,4(8)-dien-9,3-olide	C10H12O2	163.0766013	No
1060	HMDB0038184	Egomaketone	C10H12O2	163.0766013	No
1060	HMDB0040359	1,2-Dimethoxy-4-vinylbenzene	C10H12O2	163.0766013	No
1060	HMDB0040460	4-Methylbenzyl alcohol acetate	C10H12O2	163.0766013	No
1060	HMDB0041472; C07213	2-Methylbenzyl alcohol acetate	C10H12O2	163.0766013	No
1060	HMDB0041473; C07216	(3-Methylphenyl)methyl acetate	C10H12O2	163.0766013	No
1060	HMDB0041612	Phenyl butyrate	C10H12O2	163.0766013	No
1060	HMDB0127494	1-ethenyl-3,5-dimethoxybenzene	C10H12O2	163.0766013	No

1060	HMDB0133507	4-(3-hydroxyphenyl)butan-2-one	C10H12O2	163.0766013	No
1060	HMDB0133600	2-hydroxy-2-methyl-3-phenylpropanal	C10H12O2	163.0766013	No
1060	HMDB0133601	2-benzyl-3-hydroxypropanal	C10H12O2	163.0766013	No
1060	HMDB0133603	3-hydroxy-2-methyl-3-phenylpropanal	C10H12O2	163.0766013	No
1060	HMDB0133604	3-(3-hydroxyphenyl)-2-methylpropanal	C10H12O2	163.0766013	No
1060	HMDB0133605	3-(4-hydroxyphenyl)-2-methylpropanal	C10H12O2	163.0766013	No
1060	HMDB0133606	2-(phenylmethylidene)propane-1,3-diol	C10H12O2	163.0766013	No
1060	HMDB0133607	3-[(1E)-3-hydroxy-2-methylprop-1-en-1-yl]phenol	C10H12O2	163.0766013	No
1060	HMDB0133608	4-[(1E)-3-hydroxy-2-methylprop-1-en-1-yl]phenol	C10H12O2	163.0766013	No
1060	HMDB0133609	(2-methyl-3-phenyloxiran-2-yl)methanol	C10H12O2	163.0766013	No
1060	HMDB0133610	2-[(1E)-3-hydroxy-2-methylprop-1-en-1-yl]phenol	C10H12O2	163.0766013	No
1060	HMDB0133617	2-methyl-3-phenylpropanoic acid	C10H12O2	163.0766013	No
1060	HMDB0133725	(3E)-4-phenylbut-3-ene-1,2-diol	C10H12O2	163.0766013	No
1060	HMDB0133727	3-[(1E)-3-hydroxybut-1-en-1-yl]phenol	C10H12O2	163.0766013	No
1060	HMDB0133729	1-(3-phenyloxiran-2-yl)ethan-1-ol	C10H12O2	163.0766013	No
1060	HMDB0133753	1-hydroxy-4-phenylbutan-2-one	C10H12O2	163.0766013	No
1060	HMDB0133754	4-hydroxy-4-phenylbutan-2-one	C10H12O2	163.0766013	No
1060	HMDB0135737	3-(4-methoxyphenyl)propanal	C10H12O2	163.0766013	No
1060	HMDB0135738	3-(4-methoxyphenyl)prop-2-en-1-ol	C10H12O2	163.0766013	No
1061	HMDB0012246; CHEBI:130677	Kynuramine	C9H12N2O	163.0873513	No
1061	HMDB0034900	2-(1,2-Diamino-1-propenyl)phenol	C9H12N2O	163.0873513	No
1062	HMDB0062666; CHEBI:84233	Triethylene Glycol Monomethyl Ether	C7H16O4	163.0969515	No
1063	HMDB0029653	1-(1-Methoxy-1-methylethyl)-4-methylbenzene	C11H16O	163.1129607	No
1063	HMDB0031311	Benzyl butyl ether	C11H16O	163.1129607	No
1063	HMDB0031454; CHEBI:88585	trans-Jasmone	C11H16O	163.1129607	No
1063	HMDB0031624	5-Phenyl-1-pentanol	C11H16O	163.1129607	No
1063	HMDB0031625	1-Phenyl-2-pentanol	C11H16O	163.1129607	No
1063	HMDB0031866	2-Methyl-4-phenyl-2-butanol	C11H16O	163.1129607	No
1063	HMDB0032060	2-Butyl-4-methylphenol	C11H16O	163.1129607	No
1063	HMDB0035601; C08490	(Z)-3-Methyl-2-(2-pentenyl)-2-cyclopenten-1-one	C11H16O	163.1129607	No
1063	HMDB0035989	Thymol methyl ether	C11H16O	163.1129607	No
1063	HMDB0036719	Norbicycloekasantalal	C11H16O	163.1129607	No
1063	HMDB0036814	Santalone	C11H16O	163.1129607	No
1063	HMDB0036816	Norecasantalal	C11H16O	163.1129607	No
1063	HMDB0037294	2-Methyl-3-(2-pentenyl)-2-cyclopenten-1-one	C11H16O	163.1129607	No
1074	HMDB0041820; C20155; CHEBI:44605	8-Oxoguanine	C5H3N5O2	164.0216373	Yes
1075	HMDB0003454; C00971; CHEBI:16871	4-Pyridoxolactone	C8H7NO3	164.0354879	Yes
1075	HMDB0004089; C05653; CHEBI:36575	Formylanthranilic acid	C8H7NO3	164.0354879	Yes
1075	HMDB0004291; C06052; CHEBI:2124	5-Pyridoxolactone	C8H7NO3	164.0354879	Yes
1075	HMDB0013030	Noradrenochrome	C8H7NO3	164.0354879	Yes
1075	HMDB0036582	6-Methoxy-2(3H)-benzoxazolone	C8H7NO3	164.0354879	Yes
1075	HMDB0038318; C15769; CHEBI:63559	(R)-2-Hydroxy-2H-1,4-benzoxazin-3(4H)-one	C8H7NO3	164.0354879	Yes
1077	HMDB0000897; C02242; CHEBI:28664	7-Methylguanine	C6H7N5O	164.0577897	Yes
1077	HMDB0001566; C02230; CHEBI:27564	3-Methylguanine	C6H7N5O	164.0577897	Yes
1077	HMDB0003282; C04152; CHEBI:21803	1-Methylguanine	C6H7N5O	164.0577897	Yes
1077	HMDB0006040; C04153	N2-Methylguanine	C6H7N5O	164.0577897	Yes
1079	HMDB0000159; C00079; CHEBI:17295	L-Phenylalanine	C9H11NO2	164.0716265	Yes
1079	HMDB0001007	3-Pyridinebutanoic acid	C9H11NO2	164.0716265	Yes
1079	HMDB0004992; C07527; CHEBI:116735	Benzocaine	C9H11NO2	164.0716265	Yes
1079	HMDB0006044; CHEBI:110006	Norsalsolinol	C9H11NO2	164.0716265	Yes
1079	HMDB0033485	Gentiatibetine	C9H11NO2	164.0716265	Yes
1079	HMDB0033589	Ethyl 2-aminobenzoate	C9H11NO2	164.0716265	Yes
1079	HMDB0034169	Methyl N-methylanthranilate	C9H11NO2	164.0716265	Yes
1079	HMDB0059934	Benzyl glycinate	C9H11NO2	164.0716265	Yes
1079	HMDB0062402; C19565	4-Hydroxy-1-(3-pyridinyl)-1-butanone	C9H11NO2	164.0716265	Yes
1079	HMDB0062407; C19578; CHEBI:82572	5-(3-Pyridyl)-2-hydroxytetrahydrofuran	C9H11NO2	164.0716265	Yes
1080	HMDB0001943; C02765; CHEBI:51209	Pseudoephedrine	C10H15NO	164.1080703	No
1080	HMDB0004366; C06199; CHEBI:5764	Hordenine	C10H15NO	164.1080703	No
1080	HMDB0015451; C01575; CHEBI:15407	Ephedrine	C10H15NO	164.1080703	No
1080	HMDB0035652	Perilla sugar	C10H15NO	164.1080703	No
1080	HMDB0038597	Nepetalactam	C10H15NO	164.1080703	No

1080	HMDB0039665	5-Ethyl-1,2,3,4,5,6-hexahydro-7H-cyclopenta[b]pyridin	C10H15NO	164.1080703	No
1080	HMDB0039666	6-Ethyl-1,2,3,4,5,6-hexahydro-7H-cyclopenta[b]pyridin	C10H15NO	164.1080703	No
1080	HMDB0039678	3-Methyl-2-(1-pyrrolidinyl)-2-cyclopenten-1-one	C10H15NO	164.1080703	No
1080	HMDB0039679	5-Methyl-2-(1-pyrrolidinyl)-2-cyclopenten-1-one	C10H15NO	164.1080703	No
1080	HMDB0039682	2,3,4,5,6,7-Hexahydro-7-methylcyclopent[b]azepin-8(1	C10H15NO	164.1080703	No
1080	HMDB0039836	2-(5-Methyl-2-furanyl)piperidine	C10H15NO	164.1080703	No
1080	HMDB0040049	1-[(5-Methyl-2-furanyl)methyl]pyrrolidine	C10H15NO	164.1080703	No
1080	HMDB0060767	4-Hydroxymethamphetamine	C10H15NO	164.1080703	No
1092	HMDB0002107; C01606; CHEBI:29069	Phthalic acid	C8H6O4	165.0196468	No
1092	HMDB0002334	Benzoquinoneacetic acid	C8H6O4	165.0196468	No
1092	HMDB0002428; C06337; CHEBI:15702	Terephthalic acid	C8H6O4	165.0196468	No
1092	HMDB0032613	3,4-Methylenedioxybenzoic acid	C8H6O4	165.0196468	No
1093	HMDB0060412; C16614; CHEBI:28279	6-Methylmercaptopurine	C6H6N4S	165.0244019	No
1093	HMDB0136691	[(3-methylbut-2-en-1-yl)oxy]sulfonic acid	C5H10O4S	165.0244019	No
1094	HMDB0001886; C16357; CHEBI:62207	3-Methylxanthine	C6H6N4O2	165.041798	Yes
1094	HMDB0001991; C16353; CHEBI:48991	7-Methylxanthine	C6H6N4O2	165.041798	Yes
1094	HMDB0010738; CHEBI:336244	1-Methylxanthine	C6H6N4O2	165.041798	Yes
1094	HMDB0034394; CHEBI:91021	S-Propyl 1-propanesulfinothioate	C6H14OS2	165.041798	Yes
1094	HMDB0059716; CHEBI:287361	9-Methylxanthine	C6H6N4O2	165.041798	Yes
1095	HMDB0000375; C11457; CHEBI:1427	3-(3-Hydroxyphenyl)propanoic acid	C9H10O3	165.0555638	No
1095	HMDB0000563; C05607; CHEBI:8100	D-Phenyllactic acid	C9H10O3	165.0555638	No
1095	HMDB0000748; C05607; CHEBI:32978	L-3-Phenyllactic acid	C9H10O3	165.0555638	No
1095	HMDB0000779; C01479; CHEBI:25998	Phenyllactic acid	C9H10O3	165.0555638	No
1095	HMDB0002072; CHEBI:55501	4-Methoxyphenylacetic acid	C9H10O3	165.0555638	No
1095	HMDB0002199; C01744; CHEBI:32980	Desaminotyrosine	C9H10O3	165.0555638	No
1095	HMDB0002229	3-Phenoxypropionic acid	C9H10O3	165.0555638	No
1095	HMDB0005175; C05581; CHEBI:28111	Homovanillin	C9H10O3	165.0555638	No
1095	HMDB0029665; CHEBI:48408	Ethyl vanillin	C9H10O3	165.0555638	No
1095	HMDB0029817	Ethyl salicylate	C9H10O3	165.0555638	No
1095	HMDB0031132	3,4-Dihydroxyphenylacetone	C9H10O3	165.0555638	No
1095	HMDB0032030	Guaicyl acetate	C9H10O3	165.0555638	No
1095	HMDB0032138; C02201; CHEBI:17098	3,4-Dimethoxybenzaldehyde	C9H10O3	165.0555638	No
1095	HMDB0032573; CHEBI:31575	Ethylparaben	C9H10O3	165.0555638	No
1095	HMDB0032605	Methyl 2-methoxybenzoate	C9H10O3	165.0555638	No
1095	HMDB0032639; CHEBI:86903	Methyl 4-methoxybenzoate	C9H10O3	165.0555638	No
1095	HMDB0033752; C01198; CHEBI:16104	3-(2-Hydroxyphenyl)propanoic acid	C9H10O3	165.0555638	No
1095	HMDB0034243	Ipomeanine	C9H10O3	165.0555638	No
1095	HMDB0034993	4-Methoxybenzyl formate	C9H10O3	165.0555638	No
1095	HMDB0040645	3-Hydroxy-1-(4-hydroxyphenyl)-1-propanone	C9H10O3	165.0555638	No
1095	HMDB0041611	Ethyl 2-furanacrylate	C9H10O3	165.0555638	No
1095	HMDB0041683; C03080	4-Hydroxyphenyl-2-propionic acid	C9H10O3	165.0555638	No
1095	HMDB0059969	3-Methoxyphenylacetic acid	C9H10O3	165.0555638	No
1095	HMDB0062348	3,5-Dimethoxybenzaldehyde	C9H10O3	165.0555638	No
1095	HMDB0062590; C01456; CHEBI:30765	Tropate	C9H10O3	165.0555638	No
1095	HMDB0124925	3-hydroxy-3-phenylpropanoic acid	C9H10O3	165.0555638	No
1095	HMDB0125519	4-ethenyl-6-methoxybenzene-1,3-diol	C9H10O3	165.0555638	No
1095	HMDB0125525	5-ethenyl-3-methoxybenzene-1,2-diol	C9H10O3	165.0555638	No
1095	HMDB0125539	4-ethenyl-2-methoxybenzene-1,3-diol	C9H10O3	165.0555638	No
1095	HMDB0134032	3-(2,3-dihydroxyphenyl)propanal	C9H10O3	165.0555638	No
1095	HMDB0134033	3-(2,5-dihydroxyphenyl)propanal	C9H10O3	165.0555638	No
1095	HMDB0134034	2-hydroxy-3-(2-hydroxyphenyl)propanal	C9H10O3	165.0555638	No
1095	HMDB0134035	3-hydroxy-3-(2-hydroxyphenyl)propanal	C9H10O3	165.0555638	No
1095	HMDB0134036	3-(3-hydroxyprop-1-en-1-yl)benzene-1,2-diol	C9H10O3	165.0555638	No
1095	HMDB0134037	2-[3-(hydroxymethyl)oxiran-2-yl]phenol	C9H10O3	165.0555638	No
1095	HMDB0134038	2-(3-hydroxyprop-1-en-1-yl)benzene-1,4-diol	C9H10O3	165.0555638	No
1095	HMDB0137472	4-[(E)-2-hydroxyethenyl]-2-methoxyphenol	C9H10O3	165.0555638	No
1095	HMDB0141765	4-[(1E)-3-hydroxyprop-1-en-1-yl]benzene-1,2-diol	C9H10O3	165.0555638	No
1095	HMDB0141766	4-[3-(hydroxymethyl)oxiran-2-yl]phenol	C9H10O3	165.0555638	No
1095	HMDB0142136	2-(2-hydroxyphenyl)propanoic acid	C9H10O3	165.0555638	No
1095	HMDB0142137	2-hydroxy-2-phenylpropanoic acid	C9H10O3	165.0555638	No
1097	HMDB0034294	1,3,11-Tridecatriene-5,7,9-triyne	C13H10	165.0703778	Yes

1097	HMDB0039104	(3E,5Z)-1,3,5-Tridecatriene-7,9,11-triyne	C13H10	165.0703778	Yes
1098	HMDB0038183	3-(4-Methyl-3-pentenyl)thiophene	C10H14S	165.0745258	Yes
1099	HMDB0004586; C11924; CHEBI:36999	Perillic acid	C10H14O2	165.0921939	Yes
1099	HMDB0029691	Wine lactone	C10H14O2	165.0921939	Yes
1099	HMDB0031085; CHEBI:66729	6-Pentyl-2H-pyran-2-one	C10H14O2	165.0921939	Yes
1099	HMDB0032062; CHEBI:78886	2-tert-Butyl-1,4-benzenediol	C10H14O2	165.0921939	Yes
1099	HMDB0032135	2-Methoxy-4-propylphenol	C10H14O2	165.0921939	Yes
1099	HMDB0032462; C02088; CHEBI:9642	Pentyl 2-furyl ketone	C10H14O2	165.0921939	Yes
1099	HMDB0032615	1,1-Dimethoxy-2-phenylethane	C10H14O2	165.0921939	Yes
1099	HMDB0034922	alpha-Teresantalic acid	C10H14O2	165.0921939	Yes
1099	HMDB0035063	Epinepetalactone	C10H14O2	165.0921939	Yes
1099	HMDB0035235	2,4,7-Decatrienoic acid	C10H14O2	165.0921939	Yes
1099	HMDB0035328	Perilla ketone	C10H14O2	165.0921939	Yes
1099	HMDB0036042	Marmelolactone A	C10H14O2	165.0921939	Yes
1099	HMDB0036084	Isomintlactone	C10H14O2	165.0921939	Yes
1099	HMDB0036878	1,4-Epidioxy-p-mentha-2,8-diene	C10H14O2	165.0921939	Yes
1099	HMDB0036879	1,2:3,4-Diepoxy-p-menth-8-ene	C10H14O2	165.0921939	Yes
1099	HMDB0037011	5-Hydroxy-p-mentha-6,8-dien-2-one	C10H14O2	165.0921939	Yes
1099	HMDB0037012	2-Hydroxy-p-mentha-1,8-dien-6-one	C10H14O2	165.0921939	Yes
1099	HMDB0037044	Rotundifolone	C10H14O2	165.0921939	Yes
1099	HMDB0039382	(1xi,4xi,6xi)-Carvone oxide	C10H14O2	165.0921939	Yes
1099	HMDB0040176	4-Ethyl-1,2-dimethoxybenzene	C10H14O2	165.0921939	Yes
1099	HMDB0041000	Karahana lactone	C10H14O2	165.0921939	Yes
1099	HMDB0041584	3-Hydroxy-p-mentha-1,8-dien-7-al	C10H14O2	165.0921939	Yes
1099	HMDB0062603; CHEBI:84225	2-(4-methoxyphenyl)propan-2-ol	C10H14O2	165.0921939	Yes
1099	HMDB0127496	1-ethyl-3,5-dimethoxybenzene	C10H14O2	165.0921939	Yes
1099	HMDB0135742	3-(4-methoxyphenyl)propan-1-ol	C10H14O2	165.0921939	Yes
1100	HMDB0029742; CHEBI:256036	3-Isopropyl-2-methoxy-5-methylpyrazine	C9H14N2O	165.1031056	No
1100	HMDB0031860	2-Methoxy-3-(2-methylpropyl)pyrazine	C9H14N2O	165.1031056	No
1100	HMDB0032933	2-Methoxy-3-(1-methylpropyl)pyrazine	C9H14N2O	165.1031056	No
1102	HMDB0030002; CHEBI:525644	(-)-Nopol	C11H18O	165.1285788	No
1102	HMDB0030252	Limonene aldehyde	C11H18O	165.1285788	No
1102	HMDB0031359	2,4-Undecadienal	C11H18O	165.1285788	No
1102	HMDB0031565	Dihydrojasmone	C11H18O	165.1285788	No
1102	HMDB0031728	2-Hexylidenecyclopentanone	C11H18O	165.1285788	No
1102	HMDB0032540	2,6,6-Trimethyl-1-cyclohexen-1-acetaldehyde	C11H18O	165.1285788	No
1102	HMDB0033546	2,5-Undecadienal	C11H18O	165.1285788	No
1102	HMDB0036190	2-Heptylfuran	C11H18O	165.1285788	No
1102	HMDB0036815	Norecasantalol	C11H18O	165.1285788	No
1108	HMDB0030524; C14437	2(3H)-Benzothiazolethione	C7H5NS2	165.979219	No
1111	HMDB0006462	Homocysteinesulfinic acid	C4H9NO4S	166.0179804	No
1114	HMDB0030383	Zapotidine	C7H9N3S	166.0451799	Yes
1115	HMDB0001545; C00250; CHEBI:17310	Pyridoxal	C8H9NO3	166.0510044	No
1115	HMDB0004290; C06051; CHEBI:17889	Isopyridoxal	C8H9NO3	166.0510044	No
1115	HMDB0034887	3alpha,4,5,7alpha-Tetrahydro-5-hydroxy-1H-isoindole-	C8H9NO3	166.0510044	No
1115	HMDB0034889	3alpha,4,7,7alpha-Tetrahydro-4-hydroxy-1H-isoindole-	C8H9NO3	166.0510044	No
1115	HMDB0060374; CHEBI:27440	3-Methoxyanthranilate	C8H9NO3	166.0510044	No
1117	HMDB0000022; C05587; CHEBI:742324	3-Methoxytyramine	C9H13NO2	166.0873193	No
1117	HMDB0002182; C07441; CHEBI:8093	Phenylephrine	C9H13NO2	166.0873193	No
1117	HMDB0004826; C04548; CHEBI:29081	p-Synephrine	C9H13NO2	166.0873193	No
1117	HMDB0012162; CHEBI:1016357	4-Methoxytyramine	C9H13NO2	166.0873193	No
1117	HMDB0014748; C07146; CHEBI:6794	Metaraminol	C9H13NO2	166.0873193	No
1117	HMDB0015165; C07832; CHEBI:4884	Ethinamate	C9H13NO2	166.0873193	No
1117	HMDB0032020; C07453	4-(beta-Methylaminoethyl)catechol	C9H13NO2	166.0873193	No
1117	HMDB0038869	Norpandamarilactonine A	C9H13NO2	166.0873193	No
1117	HMDB0039837	2-(2-Furanyl)-3-piperidinol	C9H13NO2	166.0873193	No
1117	HMDB0041888; C07453	Epinine	C9H13NO2	166.0873193	No
1117	HMDB0060592	4-Hydroxynorephedrine	C9H13NO2	166.0873193	No
1117	HMDB0060807	a-Methyldopamine	C9H13NO2	166.0873193	No
1117	HMDB0062266; C19582; CHEBI:82576	1-(3-Pyridinyl)-1,4-butanediol	C9H13NO2	166.0873193	No
1117	HMDB0062811; C01869; CHEBI:119	D-synephrine	C9H13NO2	166.0873193	No

1119	HMDB0030371	Myrtine	C10H17NO	166.1236925	No
1119	HMDB0036030	Buccoxime	C10H17NO	166.1236925	No
1119	HMDB0037864	4-Ethyl-5-pentyloxazole	C10H17NO	166.1236925	No
1119	HMDB0037883	2,4-Diethyl-5-propyloxazole	C10H17NO	166.1236925	No
1119	HMDB0037884	2-Butyl-5-ethyl-4-methyloxazole	C10H17NO	166.1236925	No
1119	HMDB0037885	5-Butyl-4-ethyl-2-methyloxazole	C10H17NO	166.1236925	No
1119	HMDB0037886	2-Butyl-4-ethyl-5-methyloxazole	C10H17NO	166.1236925	No
1119	HMDB0037887	4,5-Dimethyl-2-pentyloxazole	C10H17NO	166.1236925	No
1119	HMDB0037888	4-Butyl-2-ethyl-5-methyloxazole	C10H17NO	166.1236925	No
1124	HMDB0000263; C00074; CHEBI:44897	Phosphoenolpyruvic acid	C3H5O6P	166.9753138	No
1127	HMDB0033042	Methyl 1-(methylthio)propyl disulfide	C5H12S3	167.003725	Yes
1127	HMDB0033046	Ethyl 1-(methylthio)ethyl disulfide	C5H12S3	167.003725	Yes
1127	HMDB0033056	Ethyl (ethylthio)methyl disulfide	C5H12S3	167.003725	Yes
1127	HMDB0033057	1-(Ethylthio)ethyl methyl disulfide	C5H12S3	167.003725	Yes
1128	HMDB0000289; C00366; CHEBI:17775	Uric acid	C5H4N4O3	167.0214313	Yes
1130	HMDB0030929	2,4,6,8-Tridecatetrayne	C13H12	167.0869155	Yes
1130	HMDB0032610	4-Methylbiphenyl	C13H12	167.0869155	Yes
1130	HMDB0038754	(E,E,E)-1,3,5,11-Tridecatetraene-7,9-diyne	C13H12	167.0869155	Yes
1132	HMDB0013105	trans-4,5-epoxy-2(E)-decenal	C10H16O2	167.1081043	Yes
1132	HMDB0031156	Ethyl (4Z)-4,7-octadienoate	C10H16O2	167.1081043	Yes
1132	HMDB0031273; CHEBI:51749	Methyl octynecarboxylate	C10H16O2	167.1081043	Yes
1132	HMDB0032219	(+/-)-Dihydromintlactone	C10H16O2	167.1081043	Yes
1132	HMDB0032312	2,4-Hexadienyl butyrate	C10H16O2	167.1081043	Yes
1132	HMDB0032313	2,4-Hexadienyl isobutyrate	C10H16O2	167.1081043	Yes
1132	HMDB0032319	cis-3-Hexenyl crotonate	C10H16O2	167.1081043	Yes
1132	HMDB0032434; CHEBI:25038	(+/-)-2-(5-Methyl-5-vinyltetrahydrofuran-2-yl)propiona	C10H16O2	167.1081043	Yes
1132	HMDB0033700	(-)-(Z)-Tetrahydro-6-(2-pentenyl)-2H-pyran-2-one	C10H16O2	167.1081043	Yes
1132	HMDB0034452	5,6-Dihydro-6-pentyl-2H-pyran-2-one	C10H16O2	167.1081043	Yes
1132	HMDB0034670	6-Hydroxy-2,6-dimethyl-2,7-octadien-4-one	C10H16O2	167.1081043	Yes
1132	HMDB0035714	Epoxyartemisia ketone	C10H16O2	167.1081043	Yes
1132	HMDB0035766; C09836; CHEBI:2866	Ascaridole	C10H16O2	167.1081043	Yes
1132	HMDB0035829	Dihydronepetalactone	C10H16O2	167.1081043	Yes
1132	HMDB0036067	Epoxycampholenic aldehyde	C10H16O2	167.1081043	Yes
1132	HMDB0036103; C16461	Geranic acid	C10H16O2	167.1081043	Yes
1132	HMDB0037014	gamma-Diosphenol	C10H16O2	167.1081043	Yes
1132	HMDB0037175	4-Isopropyl-3-cyclohexene-1-carboxylic acid	C10H16O2	167.1081043	Yes
1132	HMDB0037821; C02108; CHEBI:488	xi-1,8,8-Trimethyl-2-oxabicyclo[3.2.1]octan-3-one	C10H16O2	167.1081043	Yes
1132	HMDB0040639	(xi)-(Z)-5-(3-Hexenyl)dihydro-2(3H)-furanone	C10H16O2	167.1081043	Yes
1149	HMDB0000370; C05672; CHEBI:244030	2-Amino-3-phosphonopropionic acid	C3H8NO5P	168.0068753	No
1151	HMDB0000439; CHEBI:613373	2-Furoylglycine	C7H7NO4	168.0299565	Yes
1151	HMDB0012247; C03340; CHEBI:48052	L-2,3-Dihydrodipicolinate	C7H7NO4	168.0299565	Yes
1153	HMDB0000001; C01152; CHEBI:50599	1-Methylhistidine	C7H11N3O2	168.0779119	Yes
1153	HMDB0000479; C01152; CHEBI:27596	3-Methylhistidine	C7H11N3O2	168.0779119	Yes
1165	HMDB0060176; C11499; CHEBI:16712	(S)-3-Sulfonatolactate	C3H6O6S	168.9813432	Yes
1167	HMDB0005807; C01424; CHEBI:30778	Gallic acid	C7H6O5	169.0135704	Yes
1167	HMDB0029649	2,4,6-Trihydroxybenzoic acid	C7H6O5	169.0135704	Yes
1167	HMDB0040590	Benzyl methyl disulfide	C8H10S2	169.0135704	Yes
1167	HMDB0059964	2,3,4-Trihydroxybenzoic acid	C7H6O5	169.0135704	Yes
1167	HMDB0126491	2,4,5-trihydroxybenzoic acid	C7H6O5	169.0135704	Yes
1167	HMDB0130402	2,3,4,6-tetrahydroxybenzaldehyde	C7H6O5	169.0135704	Yes
1169	HMDB0032234	2,5-Dimethyl-3-furanthiol acetate	C8H10O2S	169.0335079	Yes
1169	HMDB0037732	S-(2-Furanylmethyl) propanethioate	C8H10O2S	169.0335079	Yes
1171	HMDB0014690; C07569; CHEBI:8502	Propylthiouracil	C7H10N2OS	169.0436725	No
1172	HMDB0000318; C05576; CHEBI:1387	3,4-Dihydroxyphenylglycol	C8H10O4	169.0506125	Yes
1172	HMDB0032966	Furaneol acetate	C8H10O4	169.0506125	Yes
1172	HMDB0038760	Hericerin III	C8H10O4	169.0506125	Yes
1172	HMDB0038761	Hericerin IV	C8H10O4	169.0506125	Yes
1172	HMDB0059756; CHEBI:89898	3,4-Methyleneadipic acid	C8H10O4	169.0506125	Yes
1172	HMDB0132906	3,5-dimethoxybenzene-1,2-diol	C8H10O4	169.0506125	Yes
1172	HMDB0132907	2,6-dimethoxybenzene-1,4-diol	C8H10O4	169.0506125	Yes
1172	HMDB0133971	3,4-dimethoxybenzene-1,2-diol	C8H10O4	169.0506125	Yes

1172	HMDB0133972	2,3-dimethoxybenzene-1,4-diol	C8H10O4	169.0506125	Yes
1172	HMDB0133982	4,5-dimethoxybenzene-1,3-diol	C8H10O4	169.0506125	Yes
1175	HMDB0032412; CHEBI:52364	Methyl beta-naphthyl ketone	C12H10O	169.0660181	Yes
1175	HMDB0032582; C02499; CHEBI:17043	2-Biphenylol	C12H10O	169.0660181	Yes
1175	HMDB0034446; C07733; CHEBI:39258	Diphenyl ether	C12H10O	169.0660181	Yes
1177	HMDB0030471	1,4-Ipomeadiol	C9H14O3	169.086806	Yes
1177	HMDB0038276	cis-3-Hexenyl pyruvate	C9H14O3	169.086806	Yes
1178	HMDB0015333; C07841; CHEBI:352567	Levetiracetam	C8H14N2O2	169.0981886	No
1178	HMDB0033926	(3xi,6xi)-Cyclo(alanylvalyl)	C8H14N2O2	169.0981886	No
1179	HMDB0059701	1,6,7-Trimethylnaphthalene	C13H14	169.1031253	No
1179	HMDB0059733	1,4,5-Trimethyl-naphtalene	C13H14	169.1031253	No
1180	HMDB0004980; CHEBI:32380	cis-4-Decenoic acid	C10H18O2	169.1233716	No
1180	HMDB0010726; CHEBI:50467	trans-Dec-2-enoic acid	C10H18O2	169.1233716	No
1180	HMDB0012183; C18202	8-Methylnonenoate	C10H18O2	169.1233716	No
1180	HMDB0029358	5Z-Octenyl acetate	C10H18O2	169.1233716	No
1180	HMDB0029859	Methyl (xi)-3-nonenoate	C10H18O2	169.1233716	No
1180	HMDB0030031	(±)-6-Methyl-5-hepten-2-yl acetate	C10H18O2	169.1233716	No
1180	HMDB0031002	3-Decenoic acid	C10H18O2	169.1233716	No
1180	HMDB0031003; CHEBI:32381	9-Decenoic acid	C10H18O2	169.1233716	No
1180	HMDB0031297	2-Octenyl acetate	C10H18O2	169.1233716	No
1180	HMDB0031362	Cyclohexylethyl acetate	C10H18O2	169.1233716	No
1180	HMDB0031440	Linalool oxide (trans-pyranoid)	C10H18O2	169.1233716	No
1180	HMDB0031441	Linalool oxide III	C10H18O2	169.1233716	No
1180	HMDB0032119	4-Hydroxy-2,6-dimethyl-7-octen-3-one	C10H18O2	169.1233716	No
1180	HMDB0032275	Ethyl 3-octenoate	C10H18O2	169.1233716	No
1180	HMDB0032393	2-Methylbutyl 3-methyl-2-butenoate	C10H18O2	169.1233716	No
1180	HMDB0032452	1-Octen-3-yl acetate	C10H18O2	169.1233716	No
1180	HMDB0033203	6-Decanolide	C10H18O2	169.1233716	No
1180	HMDB0033377	cis-3-Hexenyl butyrate	C10H18O2	169.1233716	No
1180	HMDB0034427	Cyclohexyl butanoate	C10H18O2	169.1233716	No
1180	HMDB0034581	2-Hexenyl butanoate	C10H18O2	169.1233716	No
1180	HMDB0034880	3-Methyl-2,4-nonanedione	C10H18O2	169.1233716	No
1180	HMDB0035122	(S,E)-2,6-Dimethyl-5,7-octadiene-2,3-diol	C10H18O2	169.1233716	No
1180	HMDB0035365	Cnidiol C	C10H18O2	169.1233716	No
1180	HMDB0035837; C16462	Citronellic acid	C10H18O2	169.1233716	No
1180	HMDB0035839	(E)-3-(Tetrahydro-5,5-dimethyl-2-furanyl)-2-buten-1-ol	C10H18O2	169.1233716	No
1180	HMDB0035907; CHEBI:88534	Linalyl oxide	C10H18O2	169.1233716	No
1180	HMDB0036099	Lilac alcohol	C10H18O2	169.1233716	No
1180	HMDB0036211	Hexyl crotonate	C10H18O2	169.1233716	No
1180	HMDB0036990	2,6-Dimethyl-3,7-octadiene-2,6-diol	C10H18O2	169.1233716	No
1180	HMDB0036992	1-Cyclopropyl-4-methyl-1,3-cyclohexanediol	C10H18O2	169.1233716	No
1180	HMDB0037021	trans-p-Menth-2-ene-1,4-diol	C10H18O2	169.1233716	No
1180	HMDB0037116; CHEBI:87327	delta-Decalactone	C10H18O2	169.1233716	No
1180	HMDB0037217	xi-5-Hexyldihydro-2(3H)-furanone	C10H18O2	169.1233716	No
1180	HMDB0037497	Ethyl (E)-2-octenoate	C10H18O2	169.1233716	No
1180	HMDB0037632	xi-Tetrahydro-3-pentyl-2H-pyran-2-one	C10H18O2	169.1233716	No
1180	HMDB0038744	9-Hydroxygeraniol	C10H18O2	169.1233716	No
1180	HMDB0039149	(+)-6-Hydroxy-2,6-dimethyl-7-octen-4-one	C10H18O2	169.1233716	No
1180	HMDB0039768	5-Decenoic acid	C10H18O2	169.1233716	No
1180	HMDB0039795	Ethyl 4Z-octenoate	C10H18O2	169.1233716	No
1180	HMDB0040194	Methyl 2-nonenoate	C10H18O2	169.1233716	No
1180	HMDB0040208	2-Propenyl heptanoate	C10H18O2	169.1233716	No
1180	HMDB0040213	cis-3-Hexenyl isobutyrate	C10H18O2	169.1233716	No
1180	HMDB0040330; C18066; CHEBI:50243	Menthone lactone	C10H18O2	169.1233716	No
1180	HMDB0040530	Isopentyl 3-methyl-2-butenoate	C10H18O2	169.1233716	No
1180	HMDB0040726; CHEBI:88903	(±)-cis-Linalyl oxide	C10H18O2	169.1233716	No
1180	HMDB0040727	(±)-trans-Linalyl oxide	C10H18O2	169.1233716	No
1180	HMDB0041012	6-Decenoic acid	C10H18O2	169.1233716	No
1180	HMDB0041630	(+)-cis-Linalool 3,7-oxide	C10H18O2	169.1233716	No
1180	HMDB0059610; CHEBI:16271	2-Exo-hydroxy-1,8-cineole	C10H18O2	169.1233716	No
1180	HMDB0059852; C20326	2-Hydroxycineol	C10H18O2	169.1233716	No

1192	HMDB0032420	2-(4-Methyl-5-thiazolyl)ethyl formate	C7H9NO2S	170.0282438	No
1194	HMDB0012289; C03972; CHEBI:32976	Tetrahydrodipicolinate	C7H9NO4	170.0459069	No
1195	HMDB0015052; C07203; CHEBI:6909	Metronidazole	C6H9N3O3	170.0581767	No
1199	HMDB0005015; CHEBI:42797	Gabapentin	C9H17NO2	170.1187497	No
1219	HMDB0000126; C00093; CHEBI:15978	Glycerol 3-phosphate	C3H9O6P	171.0066155	Yes
1219	HMDB0002520; C02979; CHEBI:17270	Beta-Glycerophosphoric acid	C3H9O6P	171.0066155	Yes
1220	HMDB0059933; C06677; CHEBI:27849	p-Toluenesulfonic acid	C7H8O3S	171.0122857	No
1221	HMDB0031868	Monomethyl phenylphosphonate	C7H9O3P	171.0213463	No
1224	HMDB0001212; C05565	Hydantoin-5-propionic acid	C6H8N2O4	171.0409808	Yes
1224	HMDB0034322	L-alpha-Amino-5-oxo-2(5H)-isoxazolepropanoic acid	C6H8N2O4	171.0409808	Yes
1225	HMDB0001892; C05377; CHEBI:28869	Menadione	C11H8O2	171.0450091	No
1225	HMDB0033765; C08446	Methyl (Z)-2-decene-4,6,8-triynoate	C11H8O2	171.0450091	No
1226	HMDB0037296	3-Acetyl-2,7-naphthyridine	C10H8N2O	171.0564385	No
1226	HMDB0038462	4-Hydroxy-1H-indole-3-acetonitrile	C10H8N2O	171.0564385	No
1227	HMDB0000341; CHEBI:89192	2-Octenedioic acid	C8H12O4	171.0664559	No
1227	HMDB0004982	cis-4-Octenedioic acid	C8H12O4	171.0664559	No
1227	HMDB0013312	trans-3-Octenedioic acid	C8H12O4	171.0664559	No
1227	HMDB0032352	3-Isopropenylpentanedioic acid	C8H12O4	171.0664559	No
1227	HMDB0034851	Ethyl 2,4-dioxohexanoate	C8H12O4	171.0664559	No
1227	HMDB0038601; CHEBI:68508	Diethyl fumarate	C8H12O4	171.0664559	No
1228	HMDB0000721; CHEBI:356660	Glycylproline	C7H12N2O3	171.0776209	Yes
1228	HMDB0011178; CHEBI:356329	L-prolyl-L-glycine	C7H12N2O3	171.0776209	Yes
1229	HMDB0029688	2-Ethoxynaphthalene	C12H12O	171.0815097	Yes
1229	HMDB0031621	Cinnamylideneacetone	C12H12O	171.0815097	Yes
1230	HMDB0031699	S-(3-Methyl-2-butenyl) 2-methylpropanethioate	C9H16OS	171.084631	No
1230	HMDB0039467	Allyl thiohexanoate	C9H16OS	171.084631	No
1231	HMDB0036188	Tetrahydrofurfuryl butyrate	C9H16O3	171.1026671	Yes
1231	HMDB0036213	cis-3-Hexenyl lactate	C9H16O3	171.1026671	Yes
1231	HMDB0036396	3-Methylbutyl 3-oxobutanoate	C9H16O3	171.1026671	Yes
1231	HMDB0040165	Butyl levulinate	C9H16O3	171.1026671	Yes
1231	HMDB0040202	Butyl 3-hydroxy-2-methylidenebutanoate	C9H16O3	171.1026671	Yes
1231	HMDB0040203	2-Methylpropyl 3-hydroxy-2-methylidenebutanoate	C9H16O3	171.1026671	Yes
1231	HMDB0059936	5-Pentyl-1,4-dioxan-2-one	C9H16O3	171.1026671	Yes
1231	HMDB0059937	6-Pentyl-1,4-dioxan-2-one	C9H16O3	171.1026671	Yes
1231	HMDB0060287	4-Hydroperoxy-2-nonenal	C9H16O3	171.1026671	Yes
1231	HMDB0094711; CHEBI:78700	9-Oxo-nonanoic acid	C9H16O3	171.1026671	Yes
1246	HMDB0000842; C06325; CHEBI:18386	Quinaldic acid	C10H7NO2	172.040614	No
1246	HMDB0062188; C14040; CHEBI:34104	1-nitronaphthalene	C10H7NO2	172.040614	No
1248	HMDB0006488; C01250; CHEBI:16319	N-Acetyl-L-glutamate 5-semialdehyde	C7H11NO4	172.0616402	No
1249	HMDB0004225; C03771; CHEBI:58489	2-Oxoarginine	C6H11N3O3	172.0727551	No
1252	HMDB0000701; CHEBI:64390	Hexanoylglycine	C8H15NO3	172.0979188	No
1252	HMDB0000747	Isovalerylalanine	C8H15NO3	172.0979188	No
1252	HMDB0002087	Isovalerylsarcosine	C8H15NO3	172.0979188	No
1252	HMDB0011756; C02710; CHEBI:17786	N-Acetylleucine	C8H15NO3	172.0979188	No
1252	HMDB0061684; CHEBI:84056	N-Acetylisoleucine	C8H15NO3	172.0979188	No
1268	HMDB0060015; C00850; CHEBI:27905	Phenol sulphate	C6H6O4S	172.9917773	Yes
1269	HMDB0000072; C00417; CHEBI:32805	cis-Aconitic acid	C6H6O6	173.0083949	Yes
1269	HMDB0000958; C02341; CHEBI:32806	trans-Aconitic acid	C6H6O6	173.0083949	Yes
1269	HMDB0001264; C00425; CHEBI:17242	Dehydroascorbic acid	C6H6O6	173.0083949	Yes
1271	HMDB0003070; C00493; CHEBI:16119	Shikimic acid	C7H10O5	173.0458291	Yes
1271	HMDB0012149; C04236; CHEBI:1467	2-Isopropyl-3-oxosuccinate	C7H10O5	173.0458291	Yes
1271	HMDB0061388	Dimethyl 2-oxoglutarate	C7H10O5	173.0458291	Yes
1271	HMDB0062194	2,6-dimethyl-trans-2-heptenoyl-CoA	C7H10O5	173.0458291	Yes
1271	HMDB0062377	4(R),8-dimethyl-trans-2-nonenoyl-CoA	C7H10O5	173.0458291	Yes
1271	HMDB0130150	3,4,5-trihydroxycyclohex-1-ene-1-carboxylic acid	C7H10O5	173.0458291	Yes
1272	HMDB0031845	3-Propylidene-1(3H)-isobenzofuranone	C11H10O2	173.0602628	Yes
1272	HMDB0032727	4-Methoxy-1-naphthol	C11H10O2	173.0602628	Yes
1273	HMDB0000893; C08278; CHEBI:9300	Suberic acid	C8H14O4	173.0821894	Yes
1273	HMDB0002023	Ethyladipic acid	C8H14O4	173.0821894	Yes
1273	HMDB0033838	Diethyl succinate	C8H14O4	173.0821894	Yes
1273	HMDB0041606; C14570	Dimethyl adipate	C8H14O4	173.0821894	Yes

1273	HMDB0059727; CHEBI:89933	2,4-Dimethyladipic acid	C8H14O4	173.0821894	Yes
1273	HMDB0059757	3-Methylpimelic acid	C8H14O4	173.0821894	Yes
1273	HMDB0060684; C16658; CHEBI:80644	2-Propylglutaric acid	C8H14O4	173.0821894	Yes
1274	HMDB0003357; C00437; CHEBI:16543	N-Acetylornithine	C7H14N2O3	173.0933897	Yes
1274	HMDB0028854	Glycyl-Valine	C7H14N2O3	173.0933897	Yes
1274	HMDB0029127	Valyl-Glycine	C7H14N2O3	173.0933897	Yes
1274	HMDB0034365	L-Theanine	C7H14N2O3	173.0933897	Yes
1274	HMDB0062764; C01047; CHEBI:17394	N(5)-ethyl-L-glutamine	C7H14N2O3	173.0933897	Yes
1274	HMDB0094702	Gly-Norvaline	C7H14N2O3	173.0933897	Yes
1275	HMDB0031622	6-Phenyl-3-hexen-2-one	C12H14O	173.0989478	Yes
1275	HMDB0037281	4-Methyl-2-phenyl-2-pentenal	C12H14O	173.0989478	Yes
1275	HMDB0039153	(R)-11,12,13-Trinor-1(5),6,9-guaiatrien-8-one	C12H14O	173.0989478	Yes
1275	HMDB0039466	Hexanethioic acid S-propyl ester	C9H18OS	173.0989478	Yes
1275	HMDB0040823	(S)-11,12,13-Trinor-7-calamenone	C12H14O	173.0989478	Yes
1277	HMDB0031513	(±)-3-Hydroxynonanoic acid	C9H18O3	173.1194703	Yes
1277	HMDB0034040	2,4,6-Triethyl-1,3,5-trioxane	C9H18O3	173.1194703	Yes
1278	HMDB0013287; C05545	Ne,Ne dimethyllysine	C8H18N2O2	173.1298235	Yes
1293	HMDB0000812; C01042; CHEBI:21547	N-Acetyl-L-aspartic acid	C6H9NO5	174.0408111	Yes
1293	HMDB0003470; C01045; CHEBI:48309	N-Formyl-L-glutamic acid	C6H9NO5	174.0408111	Yes
1293	HMDB0031577	Berteroin	C7H13NS2	174.0408111	Yes
1293	HMDB0039163	D-N-(Carboxyacetyl)alanine	C6H9NO5	174.0408111	Yes
1293	HMDB0060273; CHEBI:28095	2-Amino-3-oxoadipate	C6H9NO5	174.0408111	Yes
1294	HMDB0000197; C00954; CHEBI:16411	Indoleacetic acid	C10H9NO2	174.0559159	Yes
1294	HMDB0004073; C05634; CHEBI:50157	5-Hydroxyindoleacetaldehyde	C10H9NO2	174.0559159	Yes
1294	HMDB0038628	Hydroxymethyl indol-3-yl ketone	C10H9NO2	174.0559159	Yes
1294	HMDB0040972	1-Methoxy-1H-indole-3-carboxaldehyde	C10H9NO2	174.0559159	Yes
1295	HMDB0002201	N-Carboxyethyl-g-aminobutyric acid	C7H13NO4	174.0771607	No
1295	HMDB0034252; CHEBI:64305	2-Aminoheptanedioic acid	C7H13NO4	174.0771607	No
1295	HMDB0036383	Calystegine B5	C7H13NO4	174.0771607	No
1295	HMDB0038594; C10851	Calystegine B2	C7H13NO4	174.0771607	No
1295	HMDB0094717	hydroxyvalerylglycine	C7H13NO4	174.0771607	No
1317	HMDB0000044; C00072; CHEBI:17208	Ascorbic acid	C6H8O6	175.0251535	No
1317	HMDB0006355; C02670; CHEBI:18268	D-Glucurono-6,3-lactone	C6H8O6	175.0251535	No
1317	HMDB0031193; C19806; CHEBI:45969	1,2,3-Propanetricarboxylic acid	C6H8O6	175.0251535	No
1318	HMDB0000402; C02504; CHEBI:35128	2-Isopropylmalic acid	C7H12O5	175.0613898	Yes
1318	HMDB0002025	2,3-Dimethyl-3-hydroxyglutaric acid	C7H12O5	175.0613898	Yes
1318	HMDB0012156; C04411; CHEBI:35114	3-Isopropylmalate	C7H12O5	175.0613898	Yes
1318	HMDB0029169	3-Hydroxy-2-methylglutarate	C7H12O5	175.0613898	Yes
1318	HMDB0031712	(±)-Glycerol 1,2-diacetate	C7H12O5	175.0613898	Yes
1319	HMDB0028696	Alanyl-Serine	C6H12N2O4	175.0722575	Yes
1319	HMDB0028851	Glycyl-Threonine	C6H12N2O4	175.0722575	Yes
1319	HMDB0029032	Serinyl-Alanine	C6H12N2O4	175.0722575	Yes
1319	HMDB0029061	Threoninyl-Glycine	C6H12N2O4	175.0722575	Yes
1320	HMDB0002706; C00308; CHEBI:405237	Canavanine	C5H12N4O3	175.0835523	No
1337	HMDB0001015; C03145; CHEBI:16552	N-Formyl-L-methionine	C6H11NO3S	176.0387115	Yes
1337	HMDB0029417	Cycloalliin	C6H11NO3S	176.0387115	Yes
1337	HMDB0029441; C08295	Isoalliin	C6H11NO3S	176.0387115	Yes
1337	HMDB0033592; C08265	(R)C(S)S-Alliin	C6H11NO3S	176.0387115	Yes
1337	HMDB0033843	Polyvidone	C6H13NOP2	176.0387115	Yes
1343	HMDB0014968; CHEBI:775159	Phenmetrazine	C11H15NO	176.1073025	No
1343	HMDB0033484; C09991	(S)-Valerianine	C11H15NO	176.1073025	No
1343	HMDB0039664	7-Ethyl-2,3,6,7-tetrahydrocyclopent[b]azepin-8(1H)-or	C11H15NO	176.1073025	No
1343	HMDB0039667	2,3,6,7-Tetrahydro-6,7-dimethylcyclopent[b]azepin-8(	C11H15NO	176.1073025	No
1343	HMDB0040014	1-(2,3-Dihydro-1H-pyrrolizin-5-yl)-1-butanone	C11H15NO	176.1073025	No
1343	HMDB0040017	2,3-Dihydro-7-methyl-5-propanoyl-1H-pyrrolizine	C11H15NO	176.1073025	No
1343	HMDB0040018	2,3-Dihydro-6-methyl-5-propanoyl-1H-pyrrolizine	C11H15NO	176.1073025	No
1343	HMDB0040019	1-(2,3-Dihydro-5-ethyl-1H-pyrrolizin-7-yl)ethanone	C11H15NO	176.1073025	No
1343	HMDB0040020	5-Acetyl-2,3-dihydro-6,7-dimethyl-1H-pyrrolizine	C11H15NO	176.1073025	No
1343	HMDB0040027	1-(2,3-Dihydro-5,6-dimethyl-1H-pyrrolizin-7-yl)ethanoı	C11H15NO	176.1073025	No
1343	HMDB0041923; CHEBI:59331	Mephedrone	C11H15NO	176.1073025	No
1344	HMDB0014362; CHEBI:37937	Bethanidine	C10H15N3	176.1175502	No

1356	HMDB0031154; CHEBI:302136	Allitridin	C6H10S3	176.9873554	No
1361	HMDB0031162; CHEBI:1093323	3,3''-Thiobispropanoic acid	C6H10O4S	177.0219518	Yes
1363	HMDB0000150; C00198; CHEBI:16217	Gluconolactone	C6H10O6	177.0405783	Yes
1363	HMDB0001353; C01216; CHEBI:17028	2-Keto-3-deoxy-D-gluconic acid	C6H10O6	177.0405783	Yes
1363	HMDB0001385; C05394; CHEBI:27453	3-Keto-b-D-galactose	C6H10O6	177.0405783	Yes
1363	HMDB0002541; C03383; CHEBI:15895	Galactonolactone	C6H10O6	177.0405783	Yes
1363	HMDB0003466; C01040; CHEBI:17587	L-Gulonolactone	C6H10O6	177.0405783	Yes
1363	HMDB0029932; CHEBI:52417	D-Arabino-hexos-2-ulose	C6H10O6	177.0405783	Yes
1363	HMDB0031714	Methylthiomethyl 2-methylbutanethiolate	C7H14OS2	177.0405783	Yes
1364	HMDB0002040; CHEBI:260249	4-Methoxycinnamic acid	C10H10O3	177.0556439	Yes
1364	HMDB0030903	3-(4-Methylphenyl)oxiranecarboxylic acid	C10H10O3	177.0556439	Yes
1364	HMDB0031055	(Z)-10-Hydroxy-8-decene-4,6-diynoic acid	C10H10O3	177.0556439	Yes
1364	HMDB0033248	Di-alpha-furfuryl ether	C10H10O3	177.0556439	Yes
1364	HMDB0038712	(S)-Isosclerone	C10H10O3	177.0556439	Yes
1364	HMDB0038965	(E)-10-Hydroxy-2-decene-4,6-diynoic acid	C10H10O3	177.0556439	Yes
1364	HMDB0039471	3-(4-Hydroxy-2-methoxyphenyl)-2-propenal	C10H10O3	177.0556439	Yes
1364	HMDB0062273; C14784; CHEBI:34048	1,2-Dihydroxy-3,4-epoxy-1,2,3,4-tetrahydronaphthalen	C10H10O3	177.0556439	Yes
1364	HMDB0062729; CHEBI:84260	Ethyl Phenylglyoxylate	C10H10O3	177.0556439	Yes
1364	HMDB0125103	3-(3-methoxyphenyl)prop-2-enoic acid	C10H10O3	177.0556439	Yes
1364	HMDB0125518	(2E)-3-(3-methoxyphenyl)prop-2-enoic acid	C10H10O3	177.0556439	Yes
1364	HMDB0131168	methyl (2E)-3-(4-hydroxyphenyl)prop-2-enoate	C10H10O3	177.0556439	Yes
1364	HMDB0133457	4-(3,4-dihydroxyphenyl)but-3-en-2-one	C10H10O3	177.0556439	Yes
1364	HMDB0133611	(2Z)-2-(hydroxymethyl)-3-phenylprop-2-enoic acid	C10H10O3	177.0556439	Yes
1364	HMDB0133612	(2E)-3-(3-hydroxyphenyl)-2-methylprop-2-enoic acid	C10H10O3	177.0556439	Yes
1364	HMDB0133613	(2E)-3-(4-hydroxyphenyl)-2-methylprop-2-enoic acid	C10H10O3	177.0556439	Yes
1364	HMDB0133614	2-methyl-3-phenyloxirane-2-carboxylic acid	C10H10O3	177.0556439	Yes
1364	HMDB0133615	(2E)-3-(2-hydroxyphenyl)-2-methylprop-2-enoic acid	C10H10O3	177.0556439	Yes
1364	HMDB0134055	3-(2-hydroxy-3-methoxyphenyl)prop-2-enal	C10H10O3	177.0556439	Yes
1364	HMDB0135647	(2Z)-2-hydroxy-3-(4-methoxyphenyl)prop-2-enal	C10H10O3	177.0556439	Yes
1364	HMDB0135649	3-(2-hydroxy-4-methoxyphenyl)prop-2-enal	C10H10O3	177.0556439	Yes
1364	HMDB0135650	3-(4-methoxyphenyl)oxirane-2-carbaldehyde	C10H10O3	177.0556439	Yes
1364	HMDB0135651	3-(4-methoxyphenyl)prop-2-enoic acid	C10H10O3	177.0556439	Yes
1364	HMDB0141076	4-(2,4-dihydroxyphenyl)but-3-en-2-one	C10H10O3	177.0556439	Yes
1364	HMDB0141077	1-[3-(4-hydroxyphenyl)oxiran-2-yl]ethan-1-one	C10H10O3	177.0556439	Yes
1364	HMDB0141078	1-hydroxy-4-(4-hydroxyphenyl)but-3-en-2-one	C10H10O3	177.0556439	Yes
1364	HMDB0141782	(2E)-3-(4-hydroxy-3-methoxyphenyl)prop-2-enal	C10H10O3	177.0556439	Yes
1365	HMDB0001267	4-Oxo-4-(3-pyridyl)-butanamide	C9H10N2O2	177.0665037	Yes
1365	HMDB0004145	BOX B	C9H10N2O2	177.0665037	Yes
1365	HMDB0004146	BOX A	C9H10N2O2	177.0665037	Yes
1365	HMDB0015253; C07428; CHEBI:183000	Phenacemide	C9H10N2O2	177.0665037	Yes
1366	HMDB0033187	2,12-Tetradecadiene-4,6,8,10-tetrayne	C14H10	177.0715657	No
1367	HMDB0029569	Dibutyl disulfide	C8H18S2	177.0768652	No
1367	HMDB0033789	2-O-Methyl-L-fucose	C7H14O5	177.0768652	No
1367	HMDB0040348	1,8-Octanedithiol	C8H18S2	177.0768652	No
1368	HMDB0002043; CHEBI:40131	5-Phenylvaleric acid	C11H14O2	177.0922971	No
1368	HMDB0031250	Phenylmethyl 2-methylpropanoate	C11H14O2	177.0922971	No
1368	HMDB0031864; C10454; CHEBI:4918	Methyleugenol	C11H14O2	177.0922971	No
1368	HMDB0033376	Phenylmethyl butanoate	C11H14O2	177.0922971	No
1368	HMDB0033386; C17663; CHEBI:81257	3-Phenylpropyl acetate	C11H14O2	177.0922971	No
1368	HMDB0033597	4-(4-Methoxyphenyl)-2-butanone	C11H14O2	177.0922971	No
1368	HMDB0035013	2-Phenylethyl propanoate	C11H14O2	177.0922971	No
1368	HMDB0036235	alpha,alpha-Dimethylphenethyl formate	C11H14O2	177.0922971	No
1368	HMDB0036385	Methyl 4-phenylbutanoate	C11H14O2	177.0922971	No
1368	HMDB0037680	2-Methylphenyl 2-methylpropanoate	C11H14O2	177.0922971	No
1368	HMDB0037708	p-Tolyl isobutyrate	C11H14O2	177.0922971	No
1368	HMDB0038179	3-(2-Hydroxy-4-methylphenyl)-2-butanone	C11H14O2	177.0922971	No
1368	HMDB0040287	1-Phenylethyl propanoate	C11H14O2	177.0922971	No
1368	HMDB0040356	Osmorhizole	C11H14O2	177.0922971	No
1368	HMDB0040411	Ethyl 3-phenylpropanoate	C11H14O2	177.0922971	No
1368	HMDB0040425	Propyl phenylacetate	C11H14O2	177.0922971	No
1368	HMDB0040426	Isopropyl phenylacetate	C11H14O2	177.0922971	No

1368	HMDB0040583; CHEBI:87500	2-Methylpropyl benzoate	C11H14O2	177.0922971	No
1368	HMDB0040800	2-Ethoxy-5-(1-propenyl)phenol	C11H14O2	177.0922971	No
1368	HMDB0041553; C10478; CHEBI:50550	Methylisoeugenol	C11H14O2	177.0922971	No
1368	HMDB0126149	2-(3-methylbut-2-en-1-yl)benzene-1,3-diol	C11H14O2	177.0922971	No
1368	HMDB0126449	2-[(1E)-3-methylbut-1-en-1-yl]benzene-1,3-diol	C11H14O2	177.0922971	No
1368	HMDB0132981	1-(4-hydroxyphenyl)pentan-3-one	C11H14O2	177.0922971	No
1368	HMDB0133181	4-(3-methylbut-2-en-1-yl)benzene-1,3-diol	C11H14O2	177.0922971	No
1368	HMDB0133469	4-(3-methoxyphenyl)butan-2-one	C11H14O2	177.0922971	No
1368	HMDB0133682	3-hydroxy-3-methyl-4-phenylbutan-2-one	C11H14O2	177.0922971	No
1368	HMDB0133683	4-hydroxy-3-methyl-4-phenylbutan-2-one	C11H14O2	177.0922971	No
1368	HMDB0133684	4-(3-hydroxyphenyl)-3-methylbutan-2-one	C11H14O2	177.0922971	No
1368	HMDB0133685	4-(4-hydroxyphenyl)-3-methylbutan-2-one	C11H14O2	177.0922971	No
1368	HMDB0133686	3-benzyl-4-hydroxybutan-2-one	C11H14O2	177.0922971	No
1368	HMDB0133687	1-hydroxy-3-methyl-4-phenylbutan-2-one	C11H14O2	177.0922971	No
1369	HMDB0001240; CHEBI:37753	Pseudooxynicotine	C10H14N2O	177.1033552	No
1369	HMDB0001329	2''-Hydroxynicotine	C10H14N2O	177.1033552	No
1369	HMDB0001497; CHEBI:30734	Nicotine-1''-N-oxide	C10H14N2O	177.1033552	No
1369	HMDB0060679; C16569; CHEBI:357241	Glycinexylidide	C10H14N2O	177.1033552	No
1370	HMDB0014956; CHEBI:44915	Propofol	C12H18O	177.1285908	No
1370	HMDB0031194	Tricycloekasantal	C12H18O	177.1285908	No
1370	HMDB0031568	4-Methyl-1-phenyl-2-pentanol	C12H18O	177.1285908	No
1370	HMDB0031731	3-Methyl-1-phenyl-3-pentanol	C12H18O	177.1285908	No
1370	HMDB0032579	2,5-Diisopropylphenol	C12H18O	177.1285908	No
1370	HMDB0035055	11-Methyl-7-oxatetracyclo[6.3.1.01,6.04,11]dodecane	C12H18O	177.1285908	No
1370	HMDB0036681	7-Isopropyl-5-methylbicyclo[2.2.2]oct-5-en-2-one	C12H18O	177.1285908	No
1370	HMDB0037272	2-Ethoxy-1-methyl-4-(1-methylethyl)benzene	C12H18O	177.1285908	No
1370	HMDB0038107	Quinceoxepine	C12H18O	177.1285908	No
1370	HMDB0038194	Geijerone	C12H18O	177.1285908	No
1381	HMDB0029415; CHEBI:971853	S-Carboxymethyl-L-cysteine	C5H9NO4S	178.0183496	No
1383	HMDB0032581	1-(Isothiocyanatomethyl)-4-methoxybenzene	C9H9NOS	178.0321148	No
1384	HMDB0000714; C01586; CHEBI:18089	Hippuric acid	C9H9NO3	178.05085	Yes
1384	HMDB0000992; C19569; CHEBI:66951	3-Succinoylpyridine	C9H9NO3	178.05085	Yes
1384	HMDB0012884; CHEBI:838058	Adrenochrome	C9H9NO3	178.05085	Yes
1384	HMDB0032398	Methyl n-formylanthranilate	C9H9NO3	178.05085	Yes
1384	HMDB0032595	1-(4-Methoxyphenyl)-2-nitroethylene	C9H9NO3	178.05085	Yes
1390	HMDB0014621; C07236; CHEBI:193858	Rimantadine	C12H21N	178.1610713	Yes
1390	HMDB0015177; C13736; CHEBI:64312	Memantine	C12H21N	178.1610713	Yes
1426	HMDB0031671	2,4,6-Trimethyl-1,3,5-trithiane	C6H12S3	179.0033758	No
1426	HMDB0032115	1-(Methylthio)ethyl 2-propenyl disulfide	C6H12S3	179.0033758	No
1431	HMDB0001087; C03089; CHEBI:16895	5-Methylthioribose	C6H12O4S	179.0370916	Yes
1432	HMDB0003269; C05380; CHEBI:7563	Nicotinuric acid	C8H8N2O3	179.0464806	Yes
1432	HMDB0041912; C16623; CHEBI:80617	Isonicotinylglycine	C8H8N2O3	179.0464806	Yes
1432	HMDB0059766; CHEBI:467315	Picolinoylglycine	C8H8N2O3	179.0464806	Yes
1433	HMDB0000122; C00031; CHEBI:4167	D-Glucose	C6H12O6	179.0570607	No
1433	HMDB0000143; C00984; CHEBI:28061	D-Galactose	C6H12O6	179.0570607	No
1433	HMDB0000169; C00159; CHEBI:4208	D-Mannose	C6H12O6	179.0570607	No
1433	HMDB0000211; C00137; CHEBI:17268	myo-Inositol	C6H12O6	179.0570607	No
1433	HMDB0000346	3-Deoxyarabinohexonic acid	C6H12O6	179.0570607	No
1433	HMDB0000516; C00221; CHEBI:15903	Beta-D-Glucose	C6H12O6	179.0570607	No
1433	HMDB0000660; C02336; CHEBI:28645	D-Fructose	C6H12O6	179.0570607	No
1433	HMDB0001151; C01487; CHEBI:37741	Allose	C6H12O6	179.0570607	No
1433	HMDB0001266; C00247; CHEBI:10295	L-Sorbose	C6H12O6	179.0570607	No
1433	HMDB0001860; C13747; CHEBI:25858	Paraxanthine	C7H8N4O2	179.0570607	No
1433	HMDB0001889; C07130; CHEBI:28177	Theophylline	C7H8N4O2	179.0570607	No
1433	HMDB0002825; C07480; CHEBI:28946	Theobromine	C7H8N4O2	179.0570607	No
1433	HMDB0003345; C00267; CHEBI:17925	Alpha-D-Glucose	C6H12O6	179.0570607	No
1433	HMDB0003418; C00795; CHEBI:47693	D-Tagatose	C6H12O6	179.0570607	No
1433	HMDB0003449; C00962; CHEBI:27667	Beta-D-Galactose	C6H12O6	179.0570607	No
1433	HMDB0006088; C06153; CHEBI:10642	scyllo-Inositol	C6H12O6	179.0570607	No
1433	HMDB0012326; C15923; CHEBI:37704	L-Gulose	C6H12O6	179.0570607	No
1433	HMDB0032222	Dihydroxyacetone (dimer)	C6H12O6	179.0570607	No

1433	HMDB0033704; C01825; CHEBI:37619	L-Galactose	C6H12O6	179.0570607	No
1433	HMDB0034220; C06151; CHEBI:27374	Levoinositol	C6H12O6	179.0570607	No
1433	HMDB0061922; CHEBI:37744	Alpha-D-Glucopyranoside	C6H12O6	179.0570607	No
1433	HMDB0062138; C06152; CHEBI:27987	muco-Inositol	C6H12O6	179.0570607	No
1433	HMDB0062170; C00738	D-Glucopyranoside	C6H12O6	179.0570607	No
1433	HMDB0062202; CHEBI:40656	3(S)-hydroxy-all-cis-8,11,14,17-eicosatetraenoyl-CoA	C6H12O6	179.0570607	No
1433	HMDB0062473; CHEBI:37675	Glycoprotein-phospho-D-mannose	C6H12O6	179.0570607	No
1433	HMDB0062538; C10906; CHEBI:48095	Fructose-1P	C6H12O6	179.0570607	No
1433	HMDB0125599	2,5-bis(hydroxymethyl)oxolane-2,3,4-triol	C6H12O6	179.0570607	No
1433	HMDB0240207; CHEBI:23927	epi-Inositol	C6H12O6	179.0570607	No
1433	HMDB0240208; CHEBI:25492	neo-inositol	C6H12O6	179.0570607	No
1433	HMDB0240209; C06150; CHEBI:27372	D-chiro-inositol	C6H12O6	179.0570607	No
1433	HMDB0240210; CHEBI:23311	cis-inositol	C6H12O6	179.0570607	No
1433	HMDB0240211; CHEBI:22357	allo-inositol	C6H12O6	179.0570607	No
1437	HMDB0031181	Homodihydrojasmone	C12H20O	179.1442642	No
1437	HMDB0032531	2-trans-6-cis-Dodecadienal	C12H20O	179.1442642	No
1437	HMDB0038108	cis-Quinceoxepane	C12H20O	179.1442642	No
1437	HMDB0039339	Tricycloekasantalol	C12H20O	179.1442642	No
1437	HMDB0039590	(2E,4E)-2,4-Dodecadienal	C12H20O	179.1442642	No
1450	HMDB0006954; C06050	2-Methyl-3-hydroxy-5-formylpyridine-4-carboxylate	C8H7NO4	180.0293523	Yes
1450	HMDB0033733; C15770; CHEBI:63558	2,4-Dihydroxy-2H-1,4-benzoxazin-3(4H)-one	C8H7NO4	180.0293523	Yes
1450	HMDB0038054	(R)-2,7-Dihydroxy-2H-1,4-benzoxazin-3(4H)-one	C8H7NO4	180.0293523	Yes
1452	HMDB0000158; C00082; CHEBI:17895	L-Tyrosine	C9H11NO3	180.0663265	No
1452	HMDB0001119; C19579	4-Hydroxy-4-(3-pyridyl)-butanoic acid	C9H11NO3	180.0663265	No
1452	HMDB0002184	L-Threo-3-Phenylserine	C9H11NO3	180.0663265	No
1452	HMDB0003831; C04368; CHEBI:16939	Beta-Tyrosine	C9H11NO3	180.0663265	No
1452	HMDB0006050; CHEBI:278475	o-Tyrosine	C9H11NO3	180.0663265	No
1452	HMDB0059720; CHEBI:44303	Meta-Tyrosine	C9H11NO3	180.0663265	No
1452	HMDB0060280	4,6,7-Trihydroxy-1,2,3,4-tetrahydroisoquinoline	C9H11NO3	180.0663265	No
1456	HMDB0033868	beta-O-Methylynephrine	C10H15NO2	180.1030481	No
1456	HMDB0035195	cis-6-Nitro-p-mentha-1(7),2-diene	C10H15NO2	180.1030481	No
1456	HMDB0036090	(R)-2-Nitro-p-mentha-1,5-diene	C10H15NO2	180.1030481	No
1456	HMDB0039784	2,5-Dimethyl-4-(1-pyrrolidinyl)-3(2H)-furanone	C10H15NO2	180.1030481	No
1456	HMDB0039838	2-(5-Methyl-2-furanyl)-3-piperidinol	C10H15NO2	180.1030481	No
1456	HMDB0040038	5-(1-Pyrrolidinylmethyl)-2-furanmethanol	C10H15NO2	180.1030481	No
1456	HMDB0041806; CHEBI:136995	3,4-Dimethoxyphenylethylamine	C10H15NO2	180.1030481	No
1456	HMDB0060748	3-O-Methyl-a-methyldopamine	C10H15NO2	180.1030481	No
1466	HMDB0032825	Dipropyl trisulfide	C6H14S3	181.0180501	Yes
1466	HMDB0033055	Ethyl 1-(ethylthio)ethyl disulfide	C6H14S3	181.0180501	Yes
1466	HMDB0039654	Ethyl 1-(methylthio)propyl disulfide	C6H14S3	181.0180501	Yes
1466	HMDB0040235	1,1''-Thiobis-1-propanethiol	C6H14S3	181.0180501	Yes
1466	HMDB0126547	[(oxolan-2-yl)methoxy]sulfonic acid	C5H10O5S	181.0180501	Yes
1467	HMDB0001970; CHEBI:149031	3-Methyluric acid	C6H6N4O3	181.0368939	Yes
1467	HMDB0001973	9-Methyluric acid	C6H6N4O3	181.0368939	Yes
1467	HMDB0003099; C16359; CHEBI:149558	1-Methyluric acid	C6H6N4O3	181.0368939	Yes
1467	HMDB0011107; C16355; CHEBI:149744	7-Methyluric acid	C6H6N4O3	181.0368939	Yes
1468	HMDB0000118; C05582; CHEBI:545959	Homovanillic acid	C9H10O4	181.0502237	Yes
1468	HMDB0000333; CHEBI:823536	Isohomovanillic acid	C9H10O4	181.0502237	Yes
1468	HMDB0000423; C10447; CHEBI:48400	3,4-Dihydroxyhydrocinnamic acid	C9H10O4	181.0502237	Yes
1468	HMDB0000755; C03672; CHEBI:17385	Hydroxyphenyllactic acid	C9H10O4	181.0502237	Yes
1468	HMDB0002643; CHEBI:86369	3-(3-Hydroxyphenyl)-3-hydroxypropanoic acid	C9H10O4	181.0502237	Yes
1468	HMDB0004061; C05583	3-Methoxy-4-hydroxyphenylglycolaldehyde	C9H10O4	181.0502237	Yes
1468	HMDB0029232	3-Hydroxyphenyllactate	C9H10O4	181.0502237	Yes
1468	HMDB0029273; CHEBI:545935	2,6-Dimethoxybenzoic acid	C9H10O4	181.0502237	Yes
1468	HMDB0029646; C10680; CHEBI:562351	2'',6''-Dihydroxy-4''-methoxyacetophenone	C9H10O4	181.0502237	Yes
1468	HMDB0033624	(±)-2-Hydroxy-3-(2-hydroxyphenyl)propanoic acid	C9H10O4	181.0502237	Yes
1468	HMDB0037274	Maltol propionate	C9H10O4	181.0502237	Yes
1468	HMDB0041270	2'',4''-Dihydroxy-6''-methoxyacetophenone	C9H10O4	181.0502237	Yes
1468	HMDB0059763; CHEBI:296881	3,4-Dimethoxybenzoic acid	C9H10O4	181.0502237	Yes
1468	HMDB0062595; CHEBI:78336	Meta-hydroxyphenylhydracrylic Acid	C9H10O4	181.0502237	Yes
1468	HMDB0124923	3-hydroxy-3-(4-hydroxyphenyl)propanoic acid	C9H10O4	181.0502237	Yes

1468	HMDB0125533	3-(3,5-dihydroxyphenyl)propanoic acid	C9H10O4	181.0502237	Yes
1468	HMDB0125591	4-ethenyl-6-methoxybenzene-1,2,3-triol	C9H10O4	181.0502237	Yes
1468	HMDB0125595	5-ethenyl-3-methoxybenzene-1,2,4-triol	C9H10O4	181.0502237	Yes
1468	HMDB0126386	3-(2,4-dihydroxyphenyl)propanoic acid	C9H10O4	181.0502237	Yes
1468	HMDB0127495	3,5-dimethoxybenzoic acid	C9H10O4	181.0502237	Yes
1468	HMDB0129348	2-(3,4-dihydroxyphenyl)propanoic acid	C9H10O4	181.0502237	Yes
1468	HMDB0131428	2-(3-hydroxy-5-methoxyphenyl)acetic acid	C9H10O4	181.0502237	Yes
1468	HMDB0133494	2-hydroxy-2-(3-methoxyphenyl)acetic acid	C9H10O4	181.0502237	Yes
1468	HMDB0133788	2-(2,4-dihydroxyphenyl)propanoic acid	C9H10O4	181.0502237	Yes
1468	HMDB0134042	3-(2,3-dihydroxyphenyl)propanoic acid	C9H10O4	181.0502237	Yes
1468	HMDB0134043	3-(2,5-dihydroxyphenyl)propanoic acid	C9H10O4	181.0502237	Yes
1468	HMDB0140294	2-hydroxy-2-(4-methoxyphenyl)acetic acid	C9H10O4	181.0502237	Yes
1468	HMDB0140892	2-hydroxy-3-(3-hydroxyphenyl)propanoic acid	C9H10O4	181.0502237	Yes
1469	HMDB0000107; C01697; CHEBI:16813	Galactitol	C6H14O6	181.0720257	Yes
1469	HMDB0000247; C00794; CHEBI:17924	Sorbitol	C6H14O6	181.0720257	Yes
1469	HMDB0000765; C00392; CHEBI:16899	Mannitol	C6H14O6	181.0720257	Yes
1469	HMDB0011632; C01507; CHEBI:18202	L-Iditol	C6H14O6	181.0720257	Yes
1483	HMDB0029723; C12283; CHEBI:32111	Saccharin	C7H5NO3S	181.9918506	No
1484	HMDB0062578; CHEBI:994	Cis,cis-2-amino-3-(3-oxoprop-1-enyl)but-2-enedioate	C7H5NO5	182.0070258	Yes
1486	HMDB0000017; C00847; CHEBI:17405	4-Pyridoxic acid	C8H9NO4	182.046045	Yes
1546	HMDB0015435	Polystyrene sulfonate	C8H8O3S	183.0116641	No
1553	HMDB0036117	(1''R)-Nepetalic acid	C10H16O3	183.1027614	No
1553	HMDB0036998	(S)-Oleuropeic acid	C10H16O3	183.1027614	No
1553	HMDB0039052	(4S,8R)-8,9-Dihydroxy-p-menth-1(6)-en-2-one	C10H16O3	183.1027614	No
1553	HMDB0039710	1-Acetylcyclohexyl acetate	C10H16O3	183.1027614	No
1553	HMDB0040883	(E)-10-Oxo-8-decenoic acid	C10H16O3	183.1027614	No
1554	HMDB0061384; CHEBI:133185	N-(3-acetamidopropyl)pyrrolidin-2-one	C9H16N2O2	183.1138862	Yes
1555	HMDB0031062	(Z)-3-Methyl-3-decenoic acid	C11H20O2	183.1392619	No
1555	HMDB0031063	(Z)-3-Methyl-4-decenoic acid	C11H20O2	183.1392619	No
1555	HMDB0031087	trans-2-Hexyl-1-cyclopropaneacetic acid	C11H20O2	183.1392619	No
1555	HMDB0031272	Ethyl (E)-2-nonenoate	C11H20O2	183.1392619	No
1555	HMDB0031406	Ethyl 3-cyclohexylpropionate	C11H20O2	183.1392619	No
1555	HMDB0031691	cis-3-Hexenyl pentanoate	C11H20O2	183.1392619	No
1555	HMDB0031843	2,3-Undecanedione	C11H20O2	183.1392619	No
1555	HMDB0032308; CHEBI:48944	(E)-3-Heptenyl 2-methylpropanoate	C11H20O2	183.1392619	No
1555	HMDB0032320	3-Hexenyl 2-methylbutyrate	C11H20O2	183.1392619	No
1555	HMDB0032396	Methyl 2-decenoate	C11H20O2	183.1392619	No
1555	HMDB0032404	6-Methyl-5-hepten-2-one propyleneglycol acetal	C11H20O2	183.1392619	No
1555	HMDB0032453	cis-3-Octenyl propionate	C11H20O2	183.1392619	No
1555	HMDB0032461	Pent-2-enyl hexanoate	C11H20O2	183.1392619	No
1555	HMDB0032489; C13422; CHEBI:31676	Prenyl caproate	C11H20O2	183.1392619	No
1555	HMDB0032534	trans-2-Hexenyl 2-methylbutyrate	C11H20O2	183.1392619	No
1555	HMDB0033371; C12295	Citronellyl formate	C11H20O2	183.1392619	No
1555	HMDB0033724; C13910; CHEBI:35045	Undecylenic acid	C11H20O2	183.1392619	No
1555	HMDB0034428	Cyclohexyl 3-methylbutanoate	C11H20O2	183.1392619	No
1555	HMDB0034429	Cyclohexyl pentanoate	C11H20O2	183.1392619	No
1555	HMDB0036140	5-Hexyldihydro-5-methyl-2(3H)-furanone	C11H20O2	183.1392619	No
1555	HMDB0036784	6-Hexyltetrahydro-2H-pyran-2-one	C11H20O2	183.1392619	No
1555	HMDB0037106	(x)-1-Nonen-3-yl acetate	C11H20O2	183.1392619	No
1555	HMDB0037185	Rhodinyl formate	C11H20O2	183.1392619	No
1555	HMDB0037225	Methyl citronellate	C11H20O2	183.1392619	No
1555	HMDB0037309	5-Hexyldihydro-4-methyl-2(3H)-furanone	C11H20O2	183.1392619	No
1555	HMDB0038273	2-Hexenyl valerate	C11H20O2	183.1392619	No
1555	HMDB0038274	2-Hexenyl isovalerate	C11H20O2	183.1392619	No
1555	HMDB0038277	cis-3-Hexenyl 2-methylbutanoate	C11H20O2	183.1392619	No
1555	HMDB0038278	cis-3-Hexenyl 3-methylbutanoate	C11H20O2	183.1392619	No
1555	HMDB0038311	xi-gamma-Undecalactone	C11H20O2	183.1392619	No
1555	HMDB0038906	5-Octenyl propanoate	C11H20O2	183.1392619	No
1555	HMDB0040593	2-Propenyl octanoate	C11H20O2	183.1392619	No
1556	HMDB0031019; C14996	2-Dodecanone	C12H24O	183.1751003	No
1556	HMDB0031734	2-Methylundecanal	C12H24O	183.1751003	No

1556	HMDB0032543	(+/-)-2,4,8-Trimethyl-7-nonen-2-ol	C12H24O	183.1751003	No
1556	HMDB0033933; C02278; CHEBI:27836	Lauric aldehyde	C12H24O	183.1751003	No
1566	HMDB0032986	5-(2-Hydroxyethyl)-4-methylthiazole acetate	C8H11NO2S	184.043744	No
1569	HMDB0034912	(S)-N-(4,5-Dihydro-1-methyl-4-oxo-1H-imidazol-2-yl)ala	C7H11N3O3	184.0728841	No
1572	HMDB0006348; C12449	Pseudoecgonine	C9H15NO3	184.097919	Yes
1572	HMDB0006548; C10858; CHEBI:708641	Ecgonine	C9H15NO3	184.097919	Yes
1572	HMDB0094728	2-Hepteneoylglycine	C9H15NO3	184.097919	Yes
1572	HMDB0094729	3-Hepteneoylglycine	C9H15NO3	184.097919	Yes
1572	HMDB0094730	4-Hepteneoylglycine	C9H15NO3	184.097919	Yes
1572	HMDB0094731	5-Hepteneoylglycine	C9H15NO3	184.097919	Yes
1589	HMDB0060371; CHEBI:1506	3-Fumarylpyruvate	C7H6O6	185.0101898	No
1589	HMDB0125352	2,3,4,5-tetrahydroxybenzoic acid	C7H6O6	185.0101898	No
1589	HMDB0130404	2,3,4,6-tetrahydroxybenzoic acid	C7H6O6	185.0101898	No
1592	HMDB0014533; C07615; CHEBI:617099	Carbimazole	C7H10N2O2S	185.041108	No
1594	HMDB0061890	Pyroglutamylglycine	C7H10N2O4	185.0570881	Yes
1595	HMDB0032708; C13014; CHEBI:32918	1-Naphthaleneacetic acid	C12H10O2	185.0608235	No
1597	HMDB0040973	1-Methoxy-1H-indole-3-acetonitrile	C11H10N2O	185.072205	No
1598	HMDB0030988	5-(2-Methylpropyl)tetrahydro-2-oxo-3-furancarboxylic	C9H14O4	185.0821905	No
1598	HMDB0030992	5-Butyltetrahydro-2-oxo-3-furancarboxylic acid	C9H14O4	185.0821905	No
1599	HMDB0028695	Alanyl-Proline	C8H14N2O3	185.0931636	No
1599	HMDB0029010	Prolyl-Alanine	C8H14N2O3	185.0931636	No
1600	HMDB0032205	cis- and trans-L-Mercapto-p-menthan-3-one	C10H18OS	185.1008661	No
1600	HMDB0037385	8-Mercapto-p-menthan-3-one	C10H18OS	185.1008661	No
1601	HMDB0010724; CHEBI:37157	3-Oxodecanoic acid	C10H18O3	185.1181884	Yes
1601	HMDB0030367	Ethyl (3R,5Z)-3-hydroxy-5-octenoate	C10H18O3	185.1181884	Yes
1601	HMDB0030369	3-Hydroxy-5Z-octenyl acetate	C10H18O3	185.1181884	Yes
1601	HMDB0033010	(1S,2S,4S,5R)-1,8-Epoxy-p-menthane-2,5-diol	C10H18O3	185.1181884	Yes
1601	HMDB0038692	6-Methyl-2-methylene-6-octene-1,3,8-triol	C10H18O3	185.1181884	Yes
1601	HMDB0038978	(1beta,2beta,5beta)-p-Menth-3-ene-1,2,5-triol	C10H18O3	185.1181884	Yes
1601	HMDB0039533	(E)-10-Hydroxy-8-decenoic acid	C10H18O3	185.1181884	Yes
1601	HMDB0039582	6-Hexyl-1,4-dioxan-2-one	C10H18O3	185.1181884	Yes
1601	HMDB0039587	5-Hexyl-1,4-dioxan-2-one	C10H18O3	185.1181884	Yes
1601	HMDB0040204	2-Methylbutyl 3-hydroxy-2-methylidenebutanoate	C10H18O3	185.1181884	Yes
1601	HMDB0040205	3-Methylbutyl 3-hydroxy-2-methylidenebutanoate	C10H18O3	185.1181884	Yes
1601	HMDB0041376; C17620	exo,exo-1,8-Epoxy-p-menthane-2,6-diol	C10H18O3	185.1181884	Yes
1601	HMDB0041545	(1R,2R,4R)-1,8-Epoxy-p-menthane-2,4-diol	C10H18O3	185.1181884	Yes
1602	HMDB0000947; C17715; CHEBI:32368	Undecanoic acid	C11H22O2	185.1546103	No
1602	HMDB0002373; CHEBI:63864	4,6-Dimethylnonanoic acid	C11H22O2	185.1546103	No
1602	HMDB0032044	Hexyl 3-methylbutanoate	C11H22O2	185.1546103	No
1602	HMDB0032325	2-Hexyl-4,5-dimethyl-1,3-dioxolane	C11H22O2	185.1546103	No
1602	HMDB0032448	Octanal propyleneglycol acetal	C11H22O2	185.1546103	No
1602	HMDB0033618; CHEBI:87542	3-Methylbutyl hexanoate	C11H22O2	185.1546103	No
1602	HMDB0033848	Methyl decanoate	C11H22O2	185.1546103	No
1602	HMDB0034134	Octyl propanoate	C11H22O2	185.1546103	No
1602	HMDB0034456	9-Methyldecanoic acid	C11H22O2	185.1546103	No
1602	HMDB0034458	Isobutyl enanthate	C11H22O2	185.1546103	No
1602	HMDB0034460	Heptyl butanoate	C11H22O2	185.1546103	No
1602	HMDB0036034	6,6-Dimethoxy-2,5,5-trimethyl-2-hexene	C11H22O2	185.1546103	No
1602	HMDB0036229	Pentyl hexanoate	C11H22O2	185.1546103	No
1602	HMDB0038237	xi-8-Methyldecanoic acid	C11H22O2	185.1546103	No
1602	HMDB0038267	7,7-Diethoxy-3-heptene	C11H22O2	185.1546103	No
1602	HMDB0039215	Hexyl 2-methylbutanoate	C11H22O2	185.1546103	No
1602	HMDB0040171	Heptyl 2-methylpropanoate	C11H22O2	185.1546103	No
1602	HMDB0040193; CHEBI:87501	Ethyl nonanoate	C11H22O2	185.1546103	No
1602	HMDB0040266; CHEBI:87511	Nonyl acetate	C11H22O2	185.1546103	No
1602	HMDB0041559	Butyl heptanoate	C11H22O2	185.1546103	No
1602	HMDB0059831; CHEBI:87374	Propyl octanoate	C11H22O2	185.1546103	No
1602	HMDB0059860	2-Methylbutyl hexanoate	C11H22O2	185.1546103	No
1602	HMDB0062119	Hexyl valerate	C11H22O2	185.1546103	No
1602	HMDB0094659	1,1-Dimethylethyl heptanoic acid	C11H22O2	185.1546103	No
1615	HMDB0031700	1-(Malonylamino)cyclopropanecarboxylic acid	C7H9NO5	186.0423935	No

1616	HMDB0000734; CHEBI:90333	Indoleacrylic acid	C11H9NO2	186.0554719	No
1618	HMDB0012150; C05548	2-Keto-6-acetamidocaproate	C8H13NO4	186.077088	No
1621	HMDB0013010; CHEBI:74433	N-Heptanoylglycine	C9H17NO3	186.1135618	No
1621	HMDB0037790; C01092; CHEBI:15830	Polyethylene glycol	C9H17NO3	186.1135618	No
1623	HMDB0001276; C00612; CHEBI:17927	N1-Acetylspermidine	C9H21N3O	186.1610855	No
1623	HMDB0002189; C01029; CHEBI:27911	N8-Acetylspermidine	C9H21N3O	186.1610855	No
1623	HMDB0060288	N(8)-Acetylspermidinium	C9H21N3O	186.1610855	No
1630	HMDB0011635; CHEBI:82914	p-Cresol sulfate	C7H8O4S	187.0075585	No
1630	HMDB0034782	Benzyl sulfate	C7H8O4S	187.0075585	No
1632	HMDB0000784; C08261; CHEBI:48131	Azelaic acid	C9H16O4	187.0981662	No
1632	HMDB0011717; CHEBI:88404	Nonate	C9H16O4	187.0981662	No
1632	HMDB0032200	cis- and trans-Ethyl 2,4-dimethyl-1,3-dioxolane-2-aceta	C9H16O4	187.0981662	No
1632	HMDB0032258	(+/-)-Ethyl 3-acetoxy-2-methylbutyrate	C9H16O4	187.0981662	No
1632	HMDB0032387	(+/-)-Methyl 5-acetoxyhexanoate	C9H16O4	187.0981662	No
1632	HMDB0036233	Butyl ethyl malonate	C9H16O4	187.0981662	No
1632	HMDB0059760; CHEBI:89874	2,4-Dimethylpimelic acid	C9H16O4	187.0981662	No
1632	HMDB0059783	3-Methylsuberic acid	C9H16O4	187.0981662	No
1632	HMDB0059814	Diethyl methylsuccinate	C9H16O4	187.0981662	No
1632	HMDB0059879; CHEBI:87319	Diethyl glutarate	C9H16O4	187.0981662	No
1633	HMDB0000670; C01924; CHEBI:27747	Homo-L-arginine	C7H16N4O2	187.1193102	No
1633	HMDB0029416; C03884; CHEBI:28229	L-Targinine	C7H16N4O2	187.1193102	No
1651	HMDB0004823	Lanthionine ketimine	C6H7NO4S	188.0029798	Yes
1651	HMDB0061116; CHEBI:133186	2-aminophenol sulphate	C6H7NO4S	188.0029798	Yes
1653	HMDB0000590	Glutarylglycine	C7H11NO5	188.0564266	No
1653	HMDB0001138; C00624; CHEBI:12575	N-Acetylglutamic acid	C7H11NO5	188.0564266	No
1653	HMDB0031574	1-Isothiocyanato-6-(methylthio)hexane	C8H15NS2	188.0564266	No
1653	HMDB0032499	Pyrrolidino-[1,2E]-4H-2,4-dimethyl-1,3,5-dithiazine	C8H15NS2	188.0564266	No
1656	HMDB0036604	N-Methylcalystegine B2	C8H15NO4	188.092977	Yes
1656	HMDB0062181	N-lactoyl-Valine	C8H15NO4	188.092977	Yes
1656	HMDB0094718	1-hydroxyhexanoylglycine	C8H15NO4	188.092977	Yes
1656	HMDB0094719	2-hydroxyhexanoylglycine	C8H15NO4	188.092977	Yes
1656	HMDB0094720	3-hydroxyhexanoylglycine	C8H15NO4	188.092977	Yes
1656	HMDB0094721	4-hydroxyhexanoylglycine	C8H15NO4	188.092977	Yes
1656	HMDB0094722	5-hydroxyhexanoylglycine	C8H15NO4	188.092977	Yes
1665	HMDB0059724; HMDB0061713; CHEBI:6850	Pyrocatechol sulfate	C6H6O5S	188.9866147	Yes
1666	HMDB0061120	3-Hydroxy-4-aminopyridine sulfate	C5H6N2O4S	188.9984844	Yes
1669	HMDB0032944	Coumarin-4-carboxylic acid	C10H6O4	189.0199539	No
1669	HMDB0039045	8H-1,3-Dioxolo[4,5-h][1]benzopyran-8-one	C10H6O4	189.0199539	No
1669	HMDB0039147	6-Formylumbelliferone	C10H6O4	189.0199539	No
1669	HMDB0128616	1-oxo-1H-isochromene-3-carboxylic acid	C10H6O4	189.0199539	No
1669	HMDB0128617	7-hydroxy-1-oxo-1H-isochromene-3-carbaldehyde	C10H6O4	189.0199539	No
1669	HMDB0128618	6-hydroxy-1-oxo-1H-isochromene-3-carbaldehyde	C10H6O4	189.0199539	No
1669	HMDB0128619	5-hydroxy-1-oxo-1H-isochromene-3-carbaldehyde	C10H6O4	189.0199539	No
1670	HMDB0012710; C00944; CHEBI:17947	3-Dehydroquinate	C7H10O6	189.0407561	Yes
1670	HMDB0037815	5-(Methylthio)-2-[(methylthio)methyl]-2-pentenal	C8H14OS2	189.0407561	Yes
1670	HMDB0059927	2-Hydroxy-2-(2-oxopropyl)butanedioic acid	C7H10O6	189.0407561	Yes
1672	HMDB0032991	7-Methoxy-6-methyl-2H-1-benzopyran-2-one	C11H10O3	189.055174	Yes
1672	HMDB0039359	Methyl (Z,Z)-10-hydroxy-2,8-decadiene-4,6-diynoate	C11H10O3	189.055174	Yes
1672	HMDB0040326	7-Hydroxy-2,5-dimethyl-4H-1-benzopyran-4-one	C11H10O3	189.055174	Yes
1672	HMDB0040731	Avenalumic acid	C11H10O3	189.055174	Yes
1672	HMDB0040938; C17916; CHEBI:81379	Coixinden A	C11H10O3	189.055174	Yes
1672	HMDB0041122	(S)-2,3-Dihydro-5-hydroxy-2-methyl-1,4-naphthoquino	C11H10O3	189.055174	Yes
1672	HMDB0129361	1-(4-methoxy-1-benzofuran-5-yl)ethan-1-one	C11H10O3	189.055174	Yes
1673	HMDB0031173; CHEBI:563250	6-Hydroxy-1H-indole-3-acetamide	C10H10N2O2	189.0673558	Yes
1674	HMDB0000325; CHEBI:70766	3-Hydroxysuberic acid	C8H14O5	189.0769549	Yes
1674	HMDB0040220; CHEBI:53781	Diethyl L-malate	C8H14O5	189.0769549	Yes
1674	HMDB0061681; CHEBI:84086	6-(2-Hydroxyethoxy)-6-oxohexanoic acid	C8H14O5	189.0769549	Yes
1676	HMDB0001370; C00666; CHEBI:16026	Diaminopimelic acid	C7H14N2O4	189.0880342	Yes
1676	HMDB0028697	Alanyl-Threonine	C7H14N2O4	189.0880342	Yes
1676	HMDB0029054	Threoninyl-Alanine	C7H14N2O4	189.0880342	Yes
1676	HMDB0031412	2-Amino-4-[(2-hydroxy-1-oxopropyl)amino]butanoic ac	C7H14N2O4	189.0880342	Yes

1676	HMDB0039222	N-(gamma-Glutamyl)ethanolamine	C7H14N2O4	189.0880342	Yes
1677	HMDB0030905	1-(4-Methoxyphenyl)-1-penten-3-one	C12H14O2	189.0922696	Yes
1677	HMDB0032027	3-(2-Methylpropylidene)-3alpha,4-dihydro-1(3H)-isobe	C12H14O2	189.0922696	Yes
1677	HMDB0032064; C17854	(S)-3-Butyl-1(3H)-isobenzofuranone	C12H14O2	189.0922696	Yes
1677	HMDB0032177	Benzyl trans-2-methyl-2-butenoate	C12H14O2	189.0922696	Yes
1677	HMDB0032488; C03885	Prenyl benzoate	C12H14O2	189.0922696	Yes
1677	HMDB0033033	(R)-3,7-Dimethyl-5-indanecarboxylic acid	C12H14O2	189.0922696	Yes
1677	HMDB0034277	Ligustilide	C12H14O2	189.0922696	Yes
1677	HMDB0037627	Benzyl tiglate	C12H14O2	189.0922696	Yes
1677	HMDB0037700; C06360	Propyl cinnamate	C12H14O2	189.0922696	Yes
1677	HMDB0037701	Isopropyl cinnamate	C12H14O2	189.0922696	Yes
1677	HMDB0041315	Cinnamyl propionate	C12H14O2	189.0922696	Yes
1677	HMDB0132934	1-(4-hydroxyphenyl)-4-methylpent-1-en-3-one	C12H14O2	189.0922696	Yes
1677	HMDB0132983	5-(4-methoxyphenyl)pent-1-en-3-one	C12H14O2	189.0922696	Yes
1678	HMDB0032346	(+/-)-Isobutyl 3-methylthiobutyrate	C9H18O2S	189.0957142	Yes
1678	HMDB0032372	3-Mercaptoheptyl acetate	C9H18O2S	189.0957142	Yes
1678	HMDB0032427	(S)-3-Methylthiohexyl acetate	C9H18O2S	189.0957142	Yes
1680	HMDB0013804; CHEBI:67251	beta-Damascenone	C13H18O	189.1289504	No
1680	HMDB0036178	2-(3-Phenylpropyl)tetrahydrofuran	C13H18O	189.1289504	No
1680	HMDB0036572	3,5,8-Megastigmatrien-7-one	C13H18O	189.1289504	No
1680	HMDB0036685	2,2,6,7-Tetramethylbicyclo[4.3.0]nona-1(9),4-dien-8-o	C13H18O	189.1289504	No
1680	HMDB0037763	xi-3-(4-Isopropylphenyl)-2-methylpropanal	C13H18O	189.1289504	No
1680	HMDB0059904	dehydro-beta-Ionone	C13H18O	189.1289504	No
1680	HMDB0059906	Megastigmatrienone	C13H18O	189.1289504	No
1692	HMDB0062803; CHEBI:57441	2,3-diketogulonate	C6H7O7	190.0115737	Yes
1695	HMDB0000763; C05635; CHEBI:27823	5-Hydroxyindoleacetic acid	C10H9NO3	190.0510752	Yes
1695	HMDB0035514; CHEBI:133221	xi-2,3-Dihydro-2-oxo-1H-indole-3-acetic acid	C10H9NO3	190.0510752	Yes
1695	HMDB0060400; C16596; CHEBI:80594	5-Phenyl-1,3-oxazinane-2,4-dione	C10H9NO3	190.0510752	Yes
1696	HMDB0031346	Calystegine C1	C7H13NO5	190.071495	Yes
1696	HMDB0038443	1-Isothiocyanato-4-phenylbutane	C11H13NS	190.071495	Yes
1696	HMDB0040031	2-Butylbenzothiazole	C11H13NS	190.071495	Yes
1696	HMDB0040332	Dihydro-6-isopropyl-2,4-dimethyl-4H-1,3,5-dithiazine	C8H17NS2	190.071495	Yes
1696	HMDB0040333	Dihydro-4,6-dimethyl-2-(1-methylethyl)-4H-1,3,5-dithi	C8H17NS2	190.071495	Yes
1698	HMDB0001896; CHEBI:114833	5-Methoxytryptophol	C11H13NO2	190.0875977	No
1698	HMDB0033560	(E)-5-(3,4,5,6-Tetrahydro-3-pyridylidenemethyl)-2-fura	C11H13NO2	190.0875977	No
1715	HMDB0000094; C00158; CHEBI:30769	Citric acid	C6H8O7	191.0200273	Yes
1715	HMDB0000193; C00311; CHEBI:30887	Isocitric acid	C6H8O7	191.0200273	Yes
1715	HMDB0001874; C00451; CHEBI:151	D-threo-Isocitric acid	C6H8O7	191.0200273	Yes
1715	HMDB0005971; C04575; CHEBI:15622	Diketogulonic acid	C6H8O7	191.0200273	Yes
1715	HMDB0006511; C04575; CHEBI:15622	2,3-Diketo-L-gulonate	C6H8O7	191.0200273	Yes
1715	HMDB0033717; C04617; CHEBI:160	(1R,2R)-Isocitric acid	C6H8O7	191.0200273	Yes
1715	HMDB0041862; CHEBI:85805	D-Glucaro-1,4-lactone	C6H8O7	191.0200273	Yes
1716	HMDB0003072; C06746; CHEBI:17521	Quinic acid	C7H12O6	191.0561831	No
1718	HMDB0001004	Oxoamide	C10H12N2O2	191.0821315	No
1718	HMDB0001324	N''-Hydroxymethylnorcotinine	C10H12N2O2	191.0821315	No
1718	HMDB0001390	Hydroxycotinine	C10H12N2O2	191.0821315	No
1718	HMDB0001411; CHEBI:89087	Cotinine N-oxide	C10H12N2O2	191.0821315	No
1718	HMDB0001427	5''-Hydroxycotinine	C10H12N2O2	191.0821315	No
1719	HMDB0031710	1,9-Nonanedithiol	C9H20S2	191.0926413	Yes
1720	HMDB0031571	2-Methyl-1-phenyl-2-propanyl acetate	C12H16O2	191.1077627	No
1720	HMDB0031614	4-Phenyl-2-butyl acetate	C12H16O2	191.1077627	No
1720	HMDB0031618	Ethyl 4-phenylbutanoate	C12H16O2	191.1077627	No
1720	HMDB0032043	Benzyl 3-methylbutanoate	C12H16O2	191.1077627	No
1720	HMDB0033380	3-Methylbutyl benzoate	C12H16O2	191.1077627	No
1720	HMDB0035010	2-Methylpropyl phenylacetate	C12H16O2	191.1077627	No
1720	HMDB0035014; CHEBI:87413	2-Phenylethyl butanoate	C12H16O2	191.1077627	No
1720	HMDB0035015; CHEBI:87409	2-Phenylethyl 2-methylpropanoate	C12H16O2	191.1077627	No
1720	HMDB0035447	5-Isopropyl-2-methylphenol acetate	C12H16O2	191.1077627	No
1720	HMDB0036388	3-Phenylpropyl propanoate	C12H16O2	191.1077627	No
1720	HMDB0036734	11,12,13-Trinor-1(10)-spirovetivene-2,7-dione	C12H16O2	191.1077627	No
1720	HMDB0037165	4-Methyl-2-(1-phenylethyl)-1,3-dioxolane, 9CI	C12H16O2	191.1077627	No

1720	HMDB0037709	4-Methylphenyl 3-methylbutanoate	C12H16O2	191.1077627	No
1720	HMDB0037716	1-Phenylethyl isobutyrate	C12H16O2	191.1077627	No
1720	HMDB0037717	1-Phenylethyl butyrate	C12H16O2	191.1077627	No
1720	HMDB0037792	1-Ethoxy-2-methoxy-4-(1-propenyl)benzene	C12H16O2	191.1077627	No
1720	HMDB0038180	2-(2-Hydroxy-4-methylphenyl)-3-pentanone	C12H16O2	191.1077627	No
1720	HMDB0039496	2-Ethoxy-1-methoxy-4-(1-propenyl)benzene	C12H16O2	191.1077627	No
1720	HMDB0039643	11,12,13-Trinor-1,3,5-bisabolatrien-10-oic acid	C12H16O2	191.1077627	No
1720	HMDB0039841	2-Benzyl-4,5-dimethyl-1,3-dioxolane	C12H16O2	191.1077627	No
1720	HMDB0040427	Butyl phenylacetate	C12H16O2	191.1077627	No
1720	HMDB0132942	1-(4-hydroxyphenyl)-4-methylpentan-3-one	C12H16O2	191.1077627	No
1720	HMDB0132971	1-(4-methoxyphenyl)pentan-3-one	C12H16O2	191.1077627	No
1720	HMDB0132979	(1E)-1-(4-methoxyphenyl)pent-1-en-3-ol	C12H16O2	191.1077627	No
1739	HMDB0000821; C05598; CHEBI:27480	Phenylacetylglycine	C10H11NO3	192.0664338	Yes
1739	HMDB0000859; CHEBI:468391	Methyl hippurate	C10H11NO3	192.0664338	Yes
1739	HMDB0011723; C01586; CHEBI:613303	2-Methylhippuric acid	C10H11NO3	192.0664338	Yes
1739	HMDB0013245; CHEBI:613449	m-Methylhippuric acid	C10H11NO3	192.0664338	Yes
1739	HMDB0013292; CHEBI:103973	p-Methylhippuric acid	C10H11NO3	192.0664338	Yes
1739	HMDB0032388; C06332; CHEBI:16803	Methyl n-acetylanthranilate	C10H11NO3	192.0664338	Yes
1739	HMDB0034250	Betamipron	C10H11NO3	192.0664338	Yes
1739	HMDB0060366; C16587; CHEBI:80586	3-Carbamoyl-2-phenylpropionaldehyde	C10H11NO3	192.0664338	Yes
1739	HMDB0060389; C16595	4-Hydroxy-5-phenyltetrahydro-1,3-oxazin-2-one	C10H11NO3	192.0664338	Yes
1739	HMDB0060758	4-Anilino-4-oxobutanoic acid	C10H11NO3	192.0664338	Yes
1743	HMDB0003626; CHEBI:88955	(R)-N-Methylsalsolinol	C11H15NO2	192.1026161	No
1743	HMDB0003892	(S)-N-Methylsalsolinol	C11H15NO2	192.1026161	No
1743	HMDB0012469; C09640; CHEBI:761542	(-)-Salsoline	C11H15NO2	192.1026161	No
1743	HMDB0012484	(R)-1,2-dimethyl-5,6-dihydroxy-tetrahydroisoquinoline	C11H15NO2	192.1026161	No
1743	HMDB0031797; C18418; CHEBI:38505	2-Isopropylphenyl methylcarbamate	C11H15NO2	192.1026161	No
1743	HMDB0035072	2-Methylpropyl 2-aminobenzoate	C11H15NO2	192.1026161	No
1743	HMDB0036906; CHEBI:86192	Butyl 2-aminobenzoate	C11H15NO2	192.1026161	No
1743	HMDB0037698	Ethyl N-ethylanthranilate	C11H15NO2	192.1026161	No
1761	HMDB0000127; C00191; CHEBI:42717	D-Glucuronic acid	C6H10O7	193.035759	Yes
1761	HMDB0002545; C08348; CHEBI:47962	Galacturonic acid	C6H10O7	193.035759	Yes
1761	HMDB0002704; C06472; CHEBI:24769	Iduronic acid	C6H10O7	193.035759	Yes
1761	HMDB0003363; C00470; CHEBI:33885	Pectic acid	C6H10O7	193.035759	Yes
1761	HMDB0003402; C08348; CHEBI:47954	Pectin	C6H10O7	193.035759	Yes
1761	HMDB0006334; C00618; CHEBI:16142	3-Dehydro-L-gulonate	C6H10O7	193.035759	Yes
1761	HMDB0011731; C01062	5-Keto-D-gluconate	C6H10O7	193.035759	Yes
1761	HMDB0011732; C15673	2-Keto-L-gluconate	C6H10O7	193.035759	Yes
1761	HMDB0033245	6-(Hydroxymethyl)-2,4(1H,3H)-pteridinedione	C7H6N4O3	193.035759	Yes
1761	HMDB0038491	L-Altruronic acid	C6H10O7	193.035759	Yes
1761	HMDB0127846	3,4,5,6-tetrahydroxyoxane-2-carboxylic acid	C6H10O7	193.035759	Yes
1762	HMDB0000954; C01494; CHEBI:17620	trans-Ferulic acid	C10H10O4	193.050635	No
1762	HMDB0000955; C10470; CHEBI:493263	Isoferulic acid	C10H10O4	193.050635	No
1762	HMDB0002120; CHEBI:70973	Monoethyl phthalate	C10H10O4	193.050635	No
1762	HMDB0029663; CHEBI:86956	Vanillin acetate	C10H10O4	193.050635	No
1762	HMDB0030765	(-)-trans-3,4-Dihydro-4,8-dihydroxy-3-methyl-1H-2-ber	C10H10O4	193.050635	No
1762	HMDB0032614	Piperonyl acetate	C10H10O4	193.050635	No
1762	HMDB0032652	Meconine	C10H10O4	193.050635	No
1762	HMDB0037644	3-(2-Hydroxy-3-methoxyphenyl)-2-propenoic acid	C10H10O4	193.050635	No
1762	HMDB0039606	Repandiol	C10H10O4	193.050635	No
1762	HMDB0059721; CHEBI:84093	mono-Benzyl malonate	C10H10O4	193.050635	No
1762	HMDB0061686; CHEBI:84089	2,5-Dihydroxycinnamic acid methyl ester	C10H10O4	193.050635	No
1762	HMDB0125096	3-(4-hydroxy-3-methoxyphenyl)prop-2-enoic acid	C10H10O4	193.050635	No
1762	HMDB0125524	(2E)-3-(3-hydroxy-5-methoxyphenyl)prop-2-enoic acid	C10H10O4	193.050635	No
1762	HMDB0125588	3-(3-hydroxy-5-methoxyphenyl)prop-2-enoic acid	C10H10O4	193.050635	No
1762	HMDB0127500	3-(4-methoxyphenyl)-2-oxopropanoic acid	C10H10O4	193.050635	No
1762	HMDB0131137	3-(3-hydroxy-4-methoxyphenyl)prop-2-enoic acid	C10H10O4	193.050635	No
1762	HMDB0131171	(2Z)-2-hydroxy-3-(4-methoxyphenyl)prop-2-enoic acid	C10H10O4	193.050635	No
1762	HMDB0131172	(2E)-3-(2-hydroxy-4-methoxyphenyl)prop-2-enoic acid	C10H10O4	193.050635	No
1762	HMDB0131173	3-(4-methoxyphenyl)oxirane-2-carboxylic acid	C10H10O4	193.050635	No
1762	HMDB0134088	3-(2-hydroxy-3-methoxyphenyl)prop-2-enoic acid	C10H10O4	193.050635	No

1762	HMDB0135741	3-(2-hydroxy-4-methoxyphenyl)prop-2-enoic acid	C10H10O4	193.050635	No
1762	HMDB0137510	3-(4-hydroxy-2-methoxyphenyl)prop-2-enoic acid	C10H10O4	193.050635	No
1762	HMDB0140957	(2E)-3-(3-hydroxy-2-methoxyphenyl)prop-2-enoic acid	C10H10O4	193.050635	No
1762	HMDB0141263	(2Z)-2-hydroxy-3-(3-methoxyphenyl)prop-2-enoic acid	C10H10O4	193.050635	No
1762	HMDB0141265	3-(3-methoxyphenyl)-2-oxopropanoic acid	C10H10O4	193.050635	No
1762	HMDB0142175	2-benzylpropanedioic acid	C10H10O4	193.050635	No
1763	HMDB0001867; CHEBI:104011	4-Aminohippuric acid	C9H10N2O3	193.0617684	Yes
1763	HMDB0059723; CHEBI:68456	Pyridylacetylglycine	C9H10N2O3	193.0617684	Yes
1764	HMDB0029915; C06352; CHEBI:18266	D-4-O-Methyl-myo-inositol	C7H14O6	193.0706163	No
1764	HMDB0029916; C06353; CHEBI:28762	4-O-Methyl-myo-inositol	C7H14O6	193.0706163	No
1764	HMDB0029965; CHEBI:113524	Methyl beta-D-glucopyranoside	C7H14O6	193.0706163	No
1764	HMDB0031437; C03659; CHEBI:18427	(-)-Bornesitol	C7H14O6	193.0706163	No
1764	HMDB0031449; C03365; CHEBI:15975	Sequoyitol	C7H14O6	193.0706163	No
1764	HMDB0033816	Mytilitol	C7H14O6	193.0706163	No
1764	HMDB0034219; C03844; CHEBI:28548	D-Pinitol	C7H14O6	193.0706163	No
1764	HMDB0034221; C08257; CHEBI:111	L-Quebrachitol	C7H14O6	193.0706163	No
1764	HMDB0034222; CHEBI:37209	L-Pinitol	C7H14O6	193.0706163	No
1764	HMDB0037295	3-[(2-Mercapto-1-methylpropyl)thio]-2-butanol	C8H18OS2	193.0706163	No
1764	HMDB0062564	1D-5-O-methyl-chiro-inositol	C7H14O6	193.0706163	No
1768	HMDB0014018	4-Hydroxypropofol	C12H18O2	193.1233721	No
1768	HMDB0031840	(S,Z)-Lyratol acetate	C12H18O2	193.1233721	No
1768	HMDB0032567	Hexylresorcinol	C12H18O2	193.1233721	No
1768	HMDB0034450; C17002	Neocnidilide	C12H18O2	193.1233721	No
1768	HMDB0035126	(±)-Myrtenyl acetate	C12H18O2	193.1233721	No
1768	HMDB0035375	Ethyl (2E,4E,7Z)-Decatrienoate	C12H18O2	193.1233721	No
1768	HMDB0035376	Ethyl (2E,4Z,7Z)-Decatrienoate	C12H18O2	193.1233721	No
1768	HMDB0035801	(2S,4R)-p-Mentha-1(7),5-dien-2-ol acetate	C12H18O2	193.1233721	No
1768	HMDB0037018	(S)-p-Mentha-1,8-dien-10-yl acetate	C12H18O2	193.1233721	No
1768	HMDB0037231	Perillyl acetate	C12H18O2	193.1233721	No
1768	HMDB0037389	(R)-2-Acetoxy-p-mentha-1,8-diene	C12H18O2	193.1233721	No
1768	HMDB0038046	Carvyl acetate	C12H18O2	193.1233721	No
1768	HMDB0038292	Menthadienyl acetate	C12H18O2	193.1233721	No
1768	HMDB0039685	8-Ocimenyl acetate	C12H18O2	193.1233721	No
1768	HMDB0040439	1-(2-Furanyl)-1-octanone	C12H18O2	193.1233721	No
1768	HMDB0041497	(S)-Neolyratyl acetate	C12H18O2	193.1233721	No
1768	HMDB0041610	(2,2-Diethoxyethyl)benzene	C12H18O2	193.1233721	No
1768	HMDB0132986	1-(4-methoxyphenyl)pentan-3-ol	C12H18O2	193.1233721	No
1783	HMDB0000840; C07588; CHEBI:9008	Salicyluric acid	C9H9NO4	194.0460029	No
1783	HMDB0001229; C00822; CHEBI:16852	Dopaquinone	C9H9NO4	194.0460029	No
1783	HMDB0002016; CHEBI:242989	4-Carboxyphenylglycine	C9H9NO4	194.0460029	No
1783	HMDB0002404; CHEBI:68451	Alpha-Hydroxyhippuric acid	C9H9NO4	194.0460029	No
1783	HMDB0004067; C05604; CHEBI:60872	Leucodopachrome	C9H9NO4	194.0460029	No
1783	HMDB0006116; CHEBI:594277	3-Hydroxyhippuric acid	C9H9NO4	194.0460029	No
1783	HMDB0013678; CHEBI:613375	4-Hydroxyhippuric acid	C9H9NO4	194.0460029	No
1783	HMDB0030726	2-Hydroxy-6,7-dimethoxybenzoxazole	C9H9NO4	194.0460029	No
1783	HMDB0038055	2-Hydroxy-7-methoxy-2H-1,4-benzoxazin-3(4H)-one	C9H9NO4	194.0460029	No
1783	HMDB0039119	L-Dopaquinone	C9H9NO4	194.0460029	No
1783	HMDB0060602	N-acetyl-5-aminosalicylic acid	C9H9NO4	194.0460029	No
1784	HMDB0029743; CHEBI:749489	9H-Carbazole-3-carboxaldehyde	C13H9NO	194.0617238	No
1786	HMDB0060953	2-hydroxymexiletine	C11H17NO2	194.1187908	Yes
1786	HMDB0060954	p-hydroxymexiletine	C11H17NO2	194.1187908	Yes
1840	HMDB0128930	3-methyl-4-(sulfooxy)but-2-enoic acid	C5H8O6S	194.9967849	Yes
1840	HMDB0129117	(2Z)-2-[(sulfooxy)methyl]but-2-enoic acid	C5H8O6S	194.9967849	Yes
1840	HMDB0129120	2-methyl-4-(sulfooxy)but-2-enoic acid	C5H8O6S	194.9967849	Yes
1842	HMDB0030800	3-Methoxy-4,5-methylenedioxybenzoic acid	C9H8O5	195.0321552	Yes
1842	HMDB0033044	1-(Methylthio)propyl propyl disulfide	C7H16S3	195.0321552	Yes
1842	HMDB0033045	Ethyl 1-(ethylthio)propyl disulfide	C7H16S3	195.0321552	Yes
1842	HMDB0059809; CHEBI:89654	4-Hydroxy-benzenepropanedioate	C9H8O5	195.0321552	Yes
1842	HMDB0125373	3-(3,4-dihydroxyphenyl)-2-oxopropanoic acid	C9H8O5	195.0321552	Yes
1842	HMDB0125523	(2E)-3-(3,4,5-trihydroxyphenyl)prop-2-enoic acid	C9H8O5	195.0321552	Yes
1842	HMDB0125584	3-(3,4,5-trihydroxyphenyl)prop-2-enoic acid	C9H8O5	195.0321552	Yes

1842	HMDB0126031	(2E)-3-(2,3,4-trihydroxyphenyl)prop-2-enoic acid	C9H8O5	195.0321552	Yes
1842	HMDB0126041	(2E)-3-(2,4,5-trihydroxyphenyl)prop-2-enoic acid	C9H8O5	195.0321552	Yes
1842	HMDB0131758	3-(2,4-dihydroxyphenyl)-2-oxopropanoic acid	C9H8O5	195.0321552	Yes
1842	HMDB0132449	3-(3,5-dihydroxyphenyl)-2-oxopropanoic acid	C9H8O5	195.0321552	Yes
1842	HMDB0132521	3-(2,4,5-trihydroxyphenyl)prop-2-enoic acid	C9H8O5	195.0321552	Yes
1842	HMDB0137120	2-(2-hydroxy-4-methoxyphenyl)-2-oxoacetic acid	C9H8O5	195.0321552	Yes
1842	HMDB0137166	3-(2,3,4-trihydroxyphenyl)prop-2-enoic acid	C9H8O5	195.0321552	Yes
1843	HMDB0000565; C00880; CHEBI:16534	Galactonic acid	C6H12O7	195.0516488	Yes
1843	HMDB0000625; C00257; CHEBI:33198	Gluconic acid	C6H12O7	195.0516488	Yes
1843	HMDB0001857; CHEBI:149714	1,3-Dimethyluric acid	C7H8N4O3	195.0516488	Yes
1843	HMDB0001982; CHEBI:149724	3,7-Dimethyluric acid	C7H8N4O3	195.0516488	Yes
1843	HMDB0002026	1,9-Dimethyluric acid	C7H8N4O3	195.0516488	Yes
1843	HMDB0003290; C00800; CHEBI:16154	Gulonic acid	C6H12O7	195.0516488	Yes
1843	HMDB0004308	7,9-Dimethyluric acid	C7H8N4O3	195.0516488	Yes
1843	HMDB0011103; CHEBI:149713	1,7-Dimethyluric acid	C7H8N4O3	195.0516488	Yes
1843	HMDB0059704	3,9-Dimethyluric acid	C7H8N4O3	195.0516488	Yes
1856	HMDB0062692; CHEBI:57590	O-phosphonato-L-homoserine(2-)	C4H8NO6P	196.0002313	No
1861	HMDB0033179	N-(4-hydroxyphenyl)ethoxycarbothioamide	C9H11NO2S	196.0441709	No
1863	HMDB0032055; C02997; CHEBI:16437	N-Acetylhistidine	C8H11N3O3	196.0721764	No
1864	HMDB0033188; C14416	3-Amino-1-methyl-5H-pyrido[4,3-b]indole	C12H11N3	196.0908217	No
1865	HMDB0004063; C05588; CHEBI:144365	Metanephrine	C10H15NO3	196.0980113	No
1865	HMDB0036074; C08511	Tenuazonic acid	C10H15NO3	196.0980113	No
1865	HMDB0060558; CHEBI:73248	Desglymidodrine	C10H15NO3	196.0980113	No
1865	HMDB0062493; C05588; CHEBI:6270	L-Metanephrine	C10H15NO3	196.0980113	No
1865	HMDB0094780	1,2-octadienoylglycine	C10H15NO3	196.0980113	No
1865	HMDB0094781	1,3-octadienoylglycine	C10H15NO3	196.0980113	No
1865	HMDB0094782	1,4-octadienoylglycine	C10H15NO3	196.0980113	No
1865	HMDB0094783	1,5-octadienoylglycine	C10H15NO3	196.0980113	No
1865	HMDB0094784	1,6-octadienoylglycine	C10H15NO3	196.0980113	No
1865	HMDB0094785	1,7-octadienoylglycine	C10H15NO3	196.0980113	No
1865	HMDB0094786	2,3-octadienoylglycine	C10H15NO3	196.0980113	No
1865	HMDB0094787	2,4-octadienoylglycine	C10H15NO3	196.0980113	No
1865	HMDB0094788	2,5-octadienoylglycine	C10H15NO3	196.0980113	No
1865	HMDB0094789	2,6-octadienoylglycine	C10H15NO3	196.0980113	No
1865	HMDB0094790	2,7-octadienoylglycine	C10H15NO3	196.0980113	No
1865	HMDB0094791	3,4-octadienoylglycine	C10H15NO3	196.0980113	No
1865	HMDB0094792	3,5-octadienoylglycine	C10H15NO3	196.0980113	No
1865	HMDB0094793	3,6-octadienoylglycine	C10H15NO3	196.0980113	No
1865	HMDB0094794	3,7-octadienoylglycine	C10H15NO3	196.0980113	No
1865	HMDB0094795	4,5-octadienoylglycine	C10H15NO3	196.0980113	No
1865	HMDB0094796	4,6-octadienoylglycine	C10H15NO3	196.0980113	No
1865	HMDB0094797	4,7-octadienoylglycine	C10H15NO3	196.0980113	No
1865	HMDB0094798	5,6-octadienoylglycine	C10H15NO3	196.0980113	No
1865	HMDB0094799	5,7-octadienoylglycine	C10H15NO3	196.0980113	No
1865	HMDB0094800	6,7-octadienoylglycine	C10H15NO3	196.0980113	No
1866	HMDB0036173	2-(3-Phenylpropyl)pyridine	C14H15N	196.1133142	No
1876	HMDB0040585	(±)-Sulfobutanedioic acid	C4H6O7S	196.9762045	No
1878	HMDB0062563; CHEBI:18034	4-fumarylacetoacetate(2-)	C8H6O6	197.0105506	No
1878	HMDB0130847	2-[(sulfooxy)methyl]butanoic acid	C5H10O6S	197.0105506	No
1878	HMDB0130850	2-methyl-4-(sulfooxy)butanoic acid	C5H10O6S	197.0105506	No
1878	HMDB0143265	5-(sulfooxy)pentanoic acid	C5H10O6S	197.0105506	No
1878	HMDB0143268	2-(sulfooxy)pentanoic acid	C5H10O6S	197.0105506	No
1882	HMDB0000291; C05584; CHEBI:1127735	Vanillylmandelic acid	C9H10O5	197.0476993	Yes
1882	HMDB0002085; C10833; CHEBI:454990	Syringic acid	C9H10O5	197.0476993	Yes
1882	HMDB0003503; C01207; CHEBI:17807	3-(3,4-Dihydroxyphenyl)lactic acid	C9H10O5	197.0476993	Yes
1882	HMDB0029170	3-Hydroxy-4-methoxymandelate	C9H10O5	197.0476993	Yes
1882	HMDB0033836; CHEBI:87247	Ethyl gallate	C9H10O5	197.0476993	Yes
1882	HMDB0041662	3,4-O-Dimethylgallic acid	C9H10O5	197.0476993	Yes
1882	HMDB0125024	2-(2,4,5-trihydroxyphenyl)propanoic acid	C9H10O5	197.0476993	Yes
1882	HMDB0125529	3-(3,4,5-trihydroxyphenyl)propanoic acid	C9H10O5	197.0476993	Yes
1882	HMDB0126478	3-(2,4-dihydroxyphenyl)-3-hydroxypropanoic acid	C9H10O5	197.0476993	Yes

1882	HMDB0126489	3-(2,4,5-trihydroxyphenyl)propanoic acid	C9H10O5	197.0476993	Yes
1882	HMDB0130476	2-(2,5-dihydroxy-4-methoxyphenyl)acetic acid	C9H10O5	197.0476993	Yes
1882	HMDB0131426	2-(3,4-dihydroxy-5-methoxyphenyl)acetic acid	C9H10O5	197.0476993	Yes
1882	HMDB0133489	2-hydroxy-2-(4-hydroxy-3-methoxyphenyl)acetic acid	C9H10O5	197.0476993	Yes
1882	HMDB0137136	2-hydroxy-2-(2-hydroxy-4-methoxyphenyl)acetic acid	C9H10O5	197.0476993	Yes
1882	HMDB0137823	2-(2,3,4-trihydroxyphenyl)propanoic acid	C9H10O5	197.0476993	Yes
1882	HMDB0140947	3-hydroxy-2,4-dimethoxybenzoic acid	C9H10O5	197.0476993	Yes
1882	HMDB0142084	2-hydroxy-3,4-dimethoxybenzoic acid	C9H10O5	197.0476993	Yes
1882	HMDB0142087	4-hydroxy-2,3-dimethoxybenzoic acid	C9H10O5	197.0476993	Yes
1883	HMDB0015188; C04771; CHEBI:29063	Mimosine	C8H10N2O4	197.055855	Yes
1884	HMDB0004400; C16366	5-Acetylamino-6-amino-3-methyluracil	C7H10N4O3	197.0681276	Yes
1884	HMDB0059771; CHEBI:89861	6-amino-5[N-methylformylamino]-1-methyluracil	C7H10N4O3	197.0681276	Yes
1885	HMDB0004998; CHEBI:200157	Guaifenesin	C10H14O4	197.0819365	Yes
1885	HMDB0030991	5-(3E-Pentenyl)tetrahydro-2-oxo-3-furancarboxylic aci	C10H14O4	197.0819365	Yes
1885	HMDB0033093; CHEBI:125366	3-(4-Hydroxy-3-methoxyphenyl)-1,2-propanediol	C10H14O4	197.0819365	Yes
1885	HMDB0034099	(2E,4E)-2,7-Dimethyl-2,4-octadienedioic acid	C10H14O4	197.0819365	Yes
1885	HMDB0059768; CHEBI:89887	3,4-Methylene suberic acid	C10H14O4	197.0819365	Yes
1885	HMDB0059779; CHEBI:89869	2,3-Methylene suberic acid	C10H14O4	197.0819365	Yes
1885	HMDB0131265	2,3,5-trimethoxy-6-methylphenol	C10H14O4	197.0819365	Yes
1885	HMDB0135755	4-ethyl-2,6-dimethoxybenzene-1,3-diol	C10H14O4	197.0819365	Yes
1904	HMDB0003484; C01102; CHEBI:15961	O-Phosphohomoserine	C4H10NO6P	198.0171191	No
1904	HMDB0011185; C12147; CHEBI:37525	O-Phosphothreonine	C4H10NO6P	198.0171191	No
1904	HMDB0060483; C12215	Iminoerythrose 4-phosphate	C4H10NO6P	198.0171191	No
1907	HMDB0014904; C06662; CHEBI:3736	Clavulanate	C8H9NO5	198.0410862	No
1907	HMDB0029414	(S)-alpha-Amino-4-carboxy-3-furanpropanoic acid	C8H9NO5	198.0410862	No
1908	HMDB0030376; C08289	(S)-Isowillardiine	C7H9N3O4	198.0507493	No
1909	HMDB0032424	2-(4-Methyl-5-thiazolyl)ethyl propionate	C9H13NO2S	198.0598174	Yes
1913	HMDB0006406; C12448; CHEBI:708640	Ecgonine methyl ester	C10H17NO3	198.1120614	Yes
1913	HMDB0031820	Neotussilagine	C10H17NO3	198.1120614	Yes
1913	HMDB0094801	2-octenoylglycine	C10H17NO3	198.1120614	Yes
1913	HMDB0094802	3-octenoylglycine	C10H17NO3	198.1120614	Yes
1913	HMDB0094803	4-octenoylglycine	C10H17NO3	198.1120614	Yes
1913	HMDB0094804	5-octenoylglycine	C10H17NO3	198.1120614	Yes
1913	HMDB0094805	6-octenoylglycine	C10H17NO3	198.1120614	Yes
1913	HMDB0094806	7-octenoylglycine	C10H17NO3	198.1120614	Yes
1929	HMDB0062775; CHEBI:82931	4-Vinylphenol sulfate	C8H8O4S	199.0074284	Yes
1929	HMDB0124988	(3-ethenylphenyl)oxidanesulfonic acid	C8H8O4S	199.0074284	Yes
1935	HMDB0030009	3,6-Dihydro-4-(4-methyl-3-pentenyl)-1,2-dithiin	C10H16S2	199.0626223	Yes
1936	HMDB0014738; C14244; CHEBI:34380	Monobenzone	C13H12O2	199.0738122	Yes
1936	HMDB0030646	(Z)-3-(1-Butenyl)-1H-2-benzopyran-1-one	C13H12O2	199.0738122	Yes
1936	HMDB0036460; C08458	Safynol	C13H12O2	199.0738122	Yes
1937	HMDB0029838; C06537; CHEBI:27943	Harmalol	C12H12N2O	199.0862848	Yes
1938	HMDB0000603	cis-4-Decenedioic acid	C10H16O4	199.0979435	Yes
1938	HMDB0013227	cis-5-Decenedioic acid	C10H16O4	199.0979435	Yes
1938	HMDB0030989	5-Pentyltetrahydro-2-oxo-3-furancarboxylic acid	C10H16O4	199.0979435	Yes
1938	HMDB0030990	alpha-Carboxy-delta-nonalactone	C10H16O4	199.0979435	Yes
1938	HMDB0034491	(±)-Camphoric acid	C10H16O4	199.0979435	Yes
1938	HMDB0034971	(1R,2R,3S,1''R)-Nepetalinic acid	C10H16O4	199.0979435	Yes
1938	HMDB0036715	Matsutakic acid A	C10H16O4	199.0979435	Yes
1938	HMDB0060606	Hydralazine acetone hydrazone	C11H12N4	199.0979435	Yes
1939	HMDB0032662; CHEBI:132854	(S)-9-Hydroxy-10-undecenoic acid	C11H20O3	199.1341091	Yes
1939	HMDB0036029	Clarycet	C11H20O3	199.1341091	Yes
1939	HMDB0037179	3-Nonanon-1-yl acetate	C11H20O3	199.1341091	Yes
1940	HMDB0013076	Spermine dialdehyde	C10H20N2O2	199.1452846	No
1940	HMDB0041901	Hexamethylene bisacetamide	C10H20N2O2	199.1452846	No
1941	HMDB0000638; C02679; CHEBI:30805	Dodecanoic acid	C12H24O2	199.1704016	No
1941	HMDB0030998; CHEBI:87430	Ethyl decanoate	C12H24O2	199.1704016	No
1941	HMDB0032310; CHEBI:201659	2-Heptyl butyrate	C12H24O2	199.1704016	No
1941	HMDB0032440	Nonanal propyleneglycol acetal	C12H24O2	199.1704016	No
1941	HMDB0033619	Hexyl hexanoate	C12H24O2	199.1704016	No
1941	HMDB0034128	Isopropyl nonanoate	C12H24O2	199.1704016	No

1941	HMDB0034136	Octyl butanoate	C12H24O2	199.1704016	No
1941	HMDB0034137	Octyl 2-methylpropanoate	C12H24O2	199.1704016	No
1941	HMDB0035410	(R)-Dihydrocitronellol acetate	C12H24O2	199.1704016	No
1941	HMDB0036225	Pentyl heptanoate	C12H24O2	199.1704016	No
1941	HMDB0040166	Decyl acetate	C12H24O2	199.1704016	No
1941	HMDB0059868	Isobutyl octanoate	C12H24O2	199.1704016	No
1949	HMDB0000731; C05824; CHEBI:27891	Cysteine-S-sulfate	C3H7NO5S2	199.9691555	No
1956	HMDB0062715; CHEBI:61509	N-acetyl-L-2-aminoadipate(2-)	C8H11NO5	200.0562975	No
1960	HMDB0240212	(alpha-keto-dimethyl-delta-N,N-Dimethylguanidynol) v	C8H15N3O3	200.103391	Yes
1961	HMDB0000832; CHEBI:74099	Capryloylglycine	C10H19NO3	200.1293606	Yes
1961	HMDB0013116	Valproylglycine	C10H19NO3	200.1293606	Yes
1961	HMDB0041540	N-(5-Methyl-3-oxohexyl)alanine	C10H19NO3	200.1293606	Yes
1961	HMDB0059745; CHEBI:89901	N-Acetylaminooctanoic acid	C10H19NO3	200.1293606	Yes
1968	HMDB0039431	1,2,4,5,7-Pentathiocane	C3H6S5	200.9001326	No
1976	HMDB0062551; CHEBI:82932	4-ethylphenylsulfate	C8H10O4S	201.0234058	Yes
1976	HMDB0062721; CHEBI:82935	3-ethylphenyl Sulfate	C8H10O4S	201.0234058	Yes
1978	HMDB0029436	Penmacric acid	C7H10N2O5	201.0516508	No
1979	HMDB0032706; C18750; CHEBI:50397	(2-Naphthalenyloxy)acetic acid	C12H10O3	201.0559944	No
1979	HMDB0032713	3,4'',5-Biphenyltriol	C12H10O3	201.0559944	No
1979	HMDB0034663	5-Hydroxy-2,3-dimethyl-1,4-naphthoquinone	C12H10O3	201.0559944	No
1979	HMDB0038115	Setarin	C12H10O3	201.0559944	No
1981	HMDB0032306; C04067; CHEBI:16879	4-Heptenal diethyl acetal	C9H14O5	201.0771782	No
1981	HMDB0033092	3-(1-Hydroxymethyl-1-propenyl)pentanedioic acid	C9H14O5	201.0771782	No
1983	HMDB0000792; C08277; CHEBI:41865	Sebacic acid	C10H18O4	201.1127162	Yes
1983	HMDB0031510	R-2-Hydroxy-3-methylbutanoic acid 3-Methylbutanoyl	C10H18O4	201.1127162	Yes
1983	HMDB0040196	Oxalic acid dibutyl ester	C10H18O4	201.1127162	Yes
1983	HMDB0059708; CHEBI:68453	2-Ethylsuberic acid	C10H18O4	201.1127162	Yes
1983	HMDB0059719; CHEBI:70747	Heptylmalonic acid	C10H18O4	201.1127162	Yes
1983	HMDB0059754; CHEBI:89889	3-Methylazelaic acid	C10H18O4	201.1127162	Yes
1983	HMDB0062180	N-lactoyl-Isoeucine	C10H18O4	201.1127162	Yes
1984	HMDB0001539; C03626; CHEBI:17929	Asymmetric dimethylarginine	C8H18N4O2	201.1355317	Yes
1984	HMDB0003334; CHEBI:61914	Symmetric dimethylarginine	C8H18N4O2	201.1355317	Yes
1985	HMDB0032443	3-Hydroxynonyl acetate	C11H22O3	201.1501607	No
1985	HMDB0061654	3-hydroxyundecanoic acid	C11H22O3	201.1501607	No
1986	HMDB0013224; CHEBI:74096	Caproylcholine	C11H24NO2	201.1737379	No
1987	HMDB0001256; C00750; CHEBI:15746	Spermine	C10H26N4	201.2091818	No
2004	HMDB0060484; C00331; CHEBI:29750	Indolepyruvate	C11H9NO3	202.0514188	Yes
2005	HMDB0028840	Glycyl-Glutamate	C7H11N2O5	202.0592714	No
2007	HMDB0038440	1-Isothiocyanato-7-(methylthio)heptane	C9H17NS2	202.0721487	Yes
2008	HMDB0000201; C02571	L-Acetylcarnitine	C9H17NO4	202.1088784	Yes
2008	HMDB0062176	N-lactoyl-Leucine	C9H17NO4	202.1088784	Yes
2009	HMDB0028846	Glycyl-Lysine	C8H17N3O3	202.1188616	No
2009	HMDB0028951	Lysyl-Glycine	C8H17N3O3	202.1188616	No
2023	HMDB0060013; CHEBI:133460	O-methoxycatechol-O-sulphate	C7H8O5S	203.0023881	No
2024	HMDB0041031	3,5,6-Trihydroxy-5-(hydroxymethyl)-2-methoxy-2-cyclo	C8H12O6	203.0547614	Yes
2026	HMDB0011162	L-beta-aspartyl-L-alanine	C7H12N2O5	203.0667386	Yes
2026	HMDB0011667; HMDB0029149; CHEBI:7384!	gamma-Glutamylglycine	C7H12N2O5	203.0667386	Yes
2026	HMDB0014986; C07070; CHEBI:484948	Levamisole	C11H12N2S	203.0667386	Yes
2026	HMDB0028683	Alanyl-Aspartate	C7H12N2O5	203.0667386	Yes
2026	HMDB0028746	Aspartyl-Alanine	C7H12N2O5	203.0667386	Yes
2026	HMDB0028819; CHEBI:73505	Glutamylglycine	C7H12N2O5	203.0667386	Yes
2027	HMDB0033303	Anofinic acid	C12H12O3	203.0714251	No
2027	HMDB0035095	7-Ethoxy-4-methyl-2H-1-benzopyran-2-one	C12H12O3	203.0714251	No
2028	HMDB0000929; C00078; CHEBI:16828	L-Tryptophan	C11H12N2O2	203.0825683	Yes
2028	HMDB0013609; C00525; CHEBI:16296	D-Tryptophan	C11H12N2O2	203.0825683	Yes
2028	HMDB0013840	3-Hydroxymethylantipyrine	C11H12N2O2	203.0825683	Yes
2028	HMDB0014892; C07839; CHEBI:4888	Ethotoin	C11H12N2O2	203.0825683	Yes
2028	HMDB0030396; C00806; CHEBI:27897	(±)-Tryptophan	C11H12N2O2	203.0825683	Yes
2028	HMDB0060533	Nirvanol	C11H12N2O2	203.0825683	Yes
2028	HMDB0060878	4-Hydroxyantipyrine	C11H12N2O2	203.0825683	Yes
2028	HMDB0060967	S-nirvanol	C11H12N2O2	203.0825683	Yes

2030	HMDB0029052	Serinyl-Valine	C8H16N2O4	203.1040415	Yes
2030	HMDB0029136	Valyl-Serine	C8H16N2O4	203.1040415	Yes
2030	HMDB0033891	N6-Acetyl-5S-hydroxy-L-lysine	C8H16N2O4	203.1040415	Yes
2030	HMDB0039948	3,4-Dihydroxy-2-hydroxymethyl-1-pyrrolidinepropanar	C8H16N2O4	203.1040415	Yes
2030	HMDB0094706; CHEBI:74823	Serylvaline	C8H16N2O4	203.1040415	Yes
2031	HMDB0033217	(2xi,6xi)-7-Methyl-3-methylene-1,2,6,7-octanetetrol	C10H20O4	203.1290484	Yes
2031	HMDB0033642	(2xi,3xi,6E)-3,7-Dimethyl-6-octene-1,2,3,8-tetrol	C10H20O4	203.1290484	Yes
2031	HMDB0035133	3,7-Dimethyl-3-octene-1,2,6,7-tetrol	C10H20O4	203.1290484	Yes
2031	HMDB0039053	(1alpha,2alpha,4betaH,6alpha,8R)-p-Menthane-2,6,8,9	C10H20O4	203.1290484	Yes
2031	HMDB0039974	(1S,2S,4R,8R)-p-Menthane-1,2,8,9-tetrol	C10H20O4	203.1290484	Yes
2041	HMDB0000881; C02470; CHEBI:217069	Xanthurenic acid	C10H7NO4	204.0310993	Yes
2041	HMDB0032963	Zeanic acid	C10H7NO4	204.0310993	Yes
2041	HMDB0033528	4,6-Dihydroxy-2-quinolinecarboxylic acid	C10H7NO4	204.0310993	Yes
2044	HMDB0000671; C02043; CHEBI:24813	Indolelactic acid	C11H11NO3	204.0670545	Yes
2044	HMDB0004096; C05660; CHEBI:235589	5-Methoxyindoleacetate	C11H11NO3	204.0670545	Yes
2044	HMDB0011621; CHEBI:613590	Cinnamoylglycine	C11H11NO3	204.0670545	Yes
2044	HMDB0030179	Edulitine	C11H11NO3	204.0670545	Yes
2044	HMDB0032755	Methyl 1-methoxy-1H-indole-3-carboxylate	C11H11NO3	204.0670545	Yes
2044	HMDB0059765; CHEBI:89877	3-Indolehydracrylic acid	C11H11NO3	204.0670545	Yes
2046	HMDB0036394	N-Methylcalystegine C1	C8H15NO5	204.0871273	Yes
2046	HMDB0040334	Dihydro-2,4-dimethyl-6-(2-methylpropyl)-4H-1,3,5-dith	C9H19NS2	204.0871273	Yes
2046	HMDB0040335	Dihydro-4,6-dimethyl-2-(2-methylpropyl)-4H-1,3,5-dith	C9H19NS2	204.0871273	Yes
2046	HMDB0040336	Dihydro-2,4-dimethyl-6-(1-methylpropyl)-4H-1,3,5-dith	C9H19NS2	204.0871273	Yes
2046	HMDB0040337	Dihydro-4,6-dimethyl-2-(1-methylpropyl)-4H-1,3,5-dith	C9H19NS2	204.0871273	Yes
2047	HMDB0040046	1-(2,3-Dihydro-1H-pyrrolizin-5-yl)-1,4-pentanedione	C12H15NO2	204.1024766	No
2049	HMDB0004231; C05944; CHEBI:895821	Pantothenol	C9H19NO4	204.1233211	No
2057	HMDB0059752	5-Sulfoxymethylfurfural	C6H6O6S	204.9816526	Yes
2057	HMDB0060016; CHEBI:89613	Pyrogallol-1-O-sulphate	C6H6O6S	204.9816526	Yes
2057	HMDB0060018; CHEBI:89615	Pyrogallol-2-O-sulphate	C6H6O6S	204.9816526	Yes
2060	HMDB0000379; CHEBI:30835	2-Methylcitric acid	C7H10O7	205.0360237	Yes
2060	HMDB0001451; C00725; CHEBI:30314	(R)-lipoic acid	C8H14O2S2	205.0360237	Yes
2060	HMDB0003518; C01251; CHEBI:52222	Homocitric acid	C7H10O7	205.0360237	Yes
2060	HMDB0006471; C04593; CHEBI:15607	Methylisocitric acid	C7H10O7	205.0360237	Yes
2060	HMDB0014312; CHEBI:43796	(S)-lipoic acid	C8H14O2S2	205.0360237	Yes
2060	HMDB0059932	3-(Carboxymethyl)-3-hydroxypentanedioic acid	C7H10O7	205.0360237	Yes
2064	HMDB0033584	Diethyl tartrate	C8H14O6	205.0716537	No
2064	HMDB0038791	2,4,6-Triethyl-1,3,5-oxadithiane	C9H18OS2	205.0716537	No
2066	HMDB0029049	Serinyl-Threonine	C7H14N2O5	205.0829662	Yes
2066	HMDB0029070	Threoninyl-Serine	C7H14N2O5	205.0829662	Yes
2070	HMDB0013825	4-(1,1,3,3-Tetramethylbutyl)-phenol	C14H22O	205.1599728	Yes
2070	HMDB0031737	delta-Methylionone	C14H22O	205.1599728	Yes
2070	HMDB0031738	3-Methyl-4-(2,6,6-trimethyl-2-cyclohexen-1-yl)-3-buter	C14H22O	205.1599728	Yes
2070	HMDB0032397	Methyl-delta-ionone	C14H22O	205.1599728	Yes
2070	HMDB0032528; C14205	alpha-(p-(1,1,3,3-Tetramethylbutyl)phenyl)-omega-hyc	C14H22O	205.1599728	Yes
2070	HMDB0035245	1-(2,6,6-Trimethyl-2-cyclohexen-1-yl)-1-penten-3-one	C14H22O	205.1599728	Yes
2070	HMDB0035631; C09690; CHEBI:10284	alpha-Irone	C14H22O	205.1599728	Yes
2070	HMDB0036023	Etaspirene	C14H22O	205.1599728	Yes
2070	HMDB0036024	10-Isopropyl-2,7-dimethyl-1-oxaspiro[4.5]deca-3,6-die	C14H22O	205.1599728	Yes
2070	HMDB0038130; CHEBI:88921	1-(2,6,6-Trimethyl-1-cyclohexen-1-yl)-1-penten-3-one	C14H22O	205.1599728	Yes
2070	HMDB0061831; CHEBI:88618	(Z)-alpha-Irone	C14H22O	205.1599728	Yes
2070	HMDB0133179	2-benzylheptan-1-ol	C14H22O	205.1599728	Yes
2092	HMDB0000978; C01252; CHEBI:17442	4-(2-Aminophenyl)-2,4-dioxobutanoic acid	C10H9NO4	206.0461199	Yes
2092	HMDB0060328; CHEBI:34099	1-Nitro-5,6-dihydroxy-dihydronaphthalene	C10H9NO4	206.0461199	Yes
2094	HMDB0060664; C16624; CHEBI:80618	Isoniazid pyruvate	C9H9N3O3	206.0574199	Yes
2094	HMDB0061058	triazolopropionic acid	C9H9N3O3	206.0574199	Yes
2095	HMDB0000985; C00579; CHEBI:17694	Dihydrolipoamide	C8H17NOS2	206.0681586	No
2095	HMDB0029939; CHEBI:681033	6-Amino-9H-purine-9-propanoic acid	C8H9N5O2	206.0681586	No
2097	HMDB0000512; C03519; CHEBI:16259	N-Acetyl-L-phenylalanine	C11H13NO3	206.0816784	Yes
2097	HMDB0000860; CHEBI:266653	Phenylpropionylglycine	C11H13NO3	206.0816784	Yes
2097	HMDB0002042	3-Phenylpropionylglycine	C11H13NO3	206.0816784	Yes
2097	HMDB0060608	N-isopropylterephthalamic acid	C11H13NO3	206.0816784	Yes

2097	HMDB0061682; CHEBI:84085	2-Acetamido-4-methylphenyl acetate	C11H13NO3	206.0816784	Yes
2098	HMDB0014634; C07708; CHEBI:545681	Miglitol	C8H17NO5	206.1034077	No
2100	HMDB0015319; CHEBI:453011	Ciclopirox	C12H17NO2	206.118816	No
2100	HMDB0029370; CHEBI:769975	O-Methylcorypalline	C12H17NO2	206.118816	No
2100	HMDB0037697	Isobutyl N-methylanthranilate	C12H17NO2	206.118816	No
2100	HMDB0039939	Synephrine acetonide	C12H17NO2	206.118816	No
2100	HMDB0040010	1-(2,3-Dihydro-6,7-dimethyl-1H-pyrrolizin-5-yl)-2-hydr	C12H17NO2	206.118816	No
2100	HMDB0094662	Phenyl-Leucine	C12H17NO2	206.118816	No
2112	HMDB0032653	(Z)-[3-(Methylsulfinyl)-1-propenyl] 2-propenyl disulfide	C7H12OS3	206.9971336	Yes
2115	HMDB0012210; C02147; CHEBI:18047	Dihydrolipoate	C8H16O2S2	207.0510454	Yes
2115	HMDB0034759	Methyl 5-(1-Propynyl)-2-thiophenepropanoate	C11H12O2S	207.0510454	Yes
2115	HMDB0060589	Aminofurantoin	C8H8N4O3	207.0510454	Yes
2117	HMDB0012127; C09816; CHEBI:1220624	(R)-2-Benzylsuccinate	C11H12O4	207.066411	Yes
2117	HMDB0029185; CHEBI:400905	5-(3'',4''-Dihydroxyphenyl)-gamma-valerolactone	C11H12O4	207.066411	Yes
2117	HMDB0034315; CHEBI:86549	3-(3,4-Dimethoxyphenyl)-2-propenoic acid	C11H12O4	207.066411	Yes
2117	HMDB0036055	Furapiole	C11H12O4	207.066411	Yes
2117	HMDB0037308	1-(2-Methoxy-3,4-methylenedioxyphenyl)-1-propanon	C11H12O4	207.066411	Yes
2117	HMDB0038510; C02381; CHEBI:16252	6-Methoxymellein	C11H12O4	207.066411	Yes
2117	HMDB0040892	3-(3-Methoxy-4,5-methylenedioxyphenyl)-2-propen-1-	C11H12O4	207.066411	Yes
2117	HMDB0041461	Anthriscinol	C11H12O4	207.066411	Yes
2117	HMDB0041692	5-(3'',5''-Dihydroxyphenyl)-gamma-valerolactone	C11H12O4	207.066411	Yes
2117	HMDB0127491	3-(3,5-dimethoxyphenyl)prop-2-enoic acid	C11H12O4	207.066411	Yes
2117	HMDB0131169	methyl (2E)-3-(2-hydroxy-4-methoxyphenyl)prop-2-en	C11H12O4	207.066411	Yes
2117	HMDB0133456	(3Z)-3-hydroxy-4-(4-hydroxy-3-methoxyphenyl)but-3-e	C11H12O4	207.066411	Yes
2117	HMDB0133458	4-(3,4-dihydroxy-5-methoxyphenyl)but-3-en-2-one	C11H12O4	207.066411	Yes
2117	HMDB0133459	4-(2,4-dihydroxy-5-methoxyphenyl)but-3-en-2-one	C11H12O4	207.066411	Yes
2117	HMDB0133460	4-(2,4-dihydroxy-3-methoxyphenyl)but-3-en-2-one	C11H12O4	207.066411	Yes
2117	HMDB0133461	1-[3-(4-hydroxy-3-methoxyphenyl)oxiran-2-yl]ethan-1-	C11H12O4	207.066411	Yes
2117	HMDB0133462	1-hydroxy-4-(4-hydroxy-3-methoxyphenyl)but-3-en-2-	C11H12O4	207.066411	Yes
2117	HMDB0133474	1-(4-hydroxy-3-methoxyphenyl)butane-2,3-dione	C11H12O4	207.066411	Yes
2117	HMDB0138526	3-(3,4-dimethoxyphenyl)prop-2-enoic acid	C11H12O4	207.066411	Yes
2117	HMDB0142179	2-benzylbutanedioic acid	C11H12O4	207.066411	Yes
2119	HMDB0003066; C01484; CHEBI:48965	Chalcone	C15H12O	207.0826038	Yes
2119	HMDB0060323; CHEBI:27753	1-Methoxyphenanthrene	C15H12O	207.0826038	Yes
2119	HMDB0135598	1,3-diphenylprop-2-en-1-one	C15H12O	207.0826038	Yes
2120	HMDB0029968	Ethyl beta-D-glucopyranoside	C8H16O6	207.0876408	Yes
2120	HMDB0033942	Dambonitol	C8H16O6	207.0876408	Yes
2123	HMDB0029867; C16975	Isoelemicin	C12H16O3	207.1020804	No
2123	HMDB0029872; C17821	gamma-Asarone	C12H16O3	207.1020804	No
2123	HMDB0030150	Jasmine ketolactone	C12H16O3	207.1020804	No
2123	HMDB0030197	(Z)-3-Oxo-2-(2-pentenyl)-1-cyclopenteneacetic acid	C12H16O3	207.1020804	No
2123	HMDB0030681	5-Deoxydiplosporin	C12H16O3	207.1020804	No
2123	HMDB0031469; C17846	trans-Isoasarone	C12H16O3	207.1020804	No
2123	HMDB0033778; C10451	Elemicin	C12H16O3	207.1020804	No
2123	HMDB0034991	4-Methoxybenzyl butanoate	C12H16O3	207.1020804	No
2123	HMDB0035224	3,4-Dihydro-6-methoxy-3,7-dimethyl-1H-2-benzopyrar	C12H16O3	207.1020804	No
2123	HMDB0038604	Amyl salicylate	C12H16O3	207.1020804	No
2123	HMDB0040218	2-Phenoxyethyl isobutyrate	C12H16O3	207.1020804	No
2123	HMDB0040225	Isoamyl salicylate	C12H16O3	207.1020804	No
2123	HMDB0041411	3,4-Dihydro-6-methoxy-2,2-dimethyl-2H-1-benzopyrar	C12H16O3	207.1020804	No
2123	HMDB0124910	5-methoxy-2-(3-methylbut-2-en-1-yl)benzene-1,3-diol	C12H16O3	207.1020804	No
2123	HMDB0126246	4-methoxy-2-(3-methylbut-2-en-1-yl)benzene-1,3-diol	C12H16O3	207.1020804	No
2123	HMDB0132980	4-hydroxy-1-(4-methoxyphenyl)pentan-3-one	C12H16O3	207.1020804	No
2123	HMDB0132982	1-hydroxy-5-(4-methoxyphenyl)pentan-3-one	C12H16O3	207.1020804	No
2123	HMDB0132984	2-hydroxy-1-(4-methoxyphenyl)pentan-3-one	C12H16O3	207.1020804	No
2123	HMDB0132985	1-hydroxy-1-(4-methoxyphenyl)pentan-3-one	C12H16O3	207.1020804	No
2123	HMDB0133263	4-methoxy-6-(3-methylbut-2-en-1-yl)benzene-1,3-diol	C12H16O3	207.1020804	No
2124	HMDB0015217; C07474; CHEBI:8207	Pilocarpine	C11H16N2O2	207.1140593	No
2124	HMDB0060813	Descarbonyl-lacosamide	C11H16N2O2	207.1140593	No
2127	HMDB0029668	Eremopetasinorol	C13H20O2	207.139144	No
2127	HMDB0031869	(S,E)-Lyratol propanoate	C13H20O2	207.139144	No

2127	HMDB0034672	(5alpha,8beta,9beta)-5,9-Epoxy-3,6-megastigmadien-8	C13H20O2	207.139144	No
2127	HMDB0034984	(5R,6S)-5,6-Epoxy-7-megastigmen-9-one	C13H20O2	207.139144	No
2127	HMDB0035228	Theaspirone A	C13H20O2	207.139144	No
2127	HMDB0036686	2,2,6,7-Tetramethylbicyclo[4.3.0]nona-1(9),4-diene-7,8	C13H20O2	207.139144	No
2127	HMDB0036821	3-Hydroxy-beta-ionone	C13H20O2	207.139144	No
2127	HMDB0036824	Acetal R	C13H20O2	207.139144	No
2127	HMDB0037776	5,9:6,9-Diepoxy-3-megastigmene	C13H20O2	207.139144	No
2127	HMDB0038047	Carvyl propionate	C13H20O2	207.139144	No
2127	HMDB0038529	5,9:6,9-Diepoxy-4-megastigmene	C13H20O2	207.139144	No
2127	HMDB0040233	2,3,5,6,8,8a-Hexahydro-2,5,5,8a-tetramethyl-7H-1-ber	C13H20O2	207.139144	No
2127	HMDB0041636	(3R,8E)-3-Hydroxy-5,8-megastigmadien-7-one	C13H20O2	207.139144	No
2127	HMDB0132947	1-(4-methoxyphenyl)-4-methylpentan-3-ol	C13H20O2	207.139144	No
2145	HMDB0000735; C05596; CHEBI:28595	Hydroxyphenylacetylglycine	C10H11NO4	208.0617471	Yes
2145	HMDB0060367; C16591; CHEBI:80589	3-Carbamoyl-2-phenylpropionic acid	C10H11NO4	208.0617471	Yes
2145	HMDB0126552	2-{[hydroxy(2-methoxyphenyl)methylidene]amino}ace	C10H11NO4	208.0617471	Yes
2145	HMDB0127991	2-{[hydroxy(3-methoxyphenyl)methylidene]amino}ace	C10H11NO4	208.0617471	Yes
2145	HMDB0131190	2-[(4-methoxyphenyl)formamido]acetic acid	C10H11NO4	208.0617471	Yes
2147	HMDB0032757	3-Methyl-9H-carbazole-9-carboxaldehyde	C14H11NO	208.0767522	No
2147	HMDB0033000	10-Methylacridone	C14H11NO	208.0767522	No
2147	HMDB0060652; C16604	2-Hydroxyiminostilbene	C14H11NO	208.0767522	No
2148	HMDB0015255; C11181; CHEBI:8452	Proflavine	C13H11N3	208.0856176	No
2173	HMDB0000639; C00879; CHEBI:30852	Galactaric acid	C6H10O8	209.0307692	Yes
2173	HMDB0000663; C00818; CHEBI:16002	Glucaric acid	C6H10O8	209.0307692	Yes
2173	HMDB0029881; C00818; CHEBI:1139229	D-Glucaric acid	C6H10O8	209.0307692	Yes
2174	HMDB0011714	Vanilpyruvic acid	C10H10O5	209.0456742	Yes
2174	HMDB0035484; C05619	3-(3,4-Dihydroxy-5-methoxy)-2-propenoic acid	C10H10O5	209.0456742	Yes
2174	HMDB0125514	(2E)-3-(2,4-dihydroxy-5-methoxyphenyl)prop-2-enoic a	C10H10O5	209.0456742	Yes
2174	HMDB0125515	(2E)-3-(2,4-dihydroxy-3-methoxyphenyl)prop-2-enoic a	C10H10O5	209.0456742	Yes
2174	HMDB0125516	3-(4-hydroxy-3-methoxyphenyl)oxirane-2-carboxylic ac	C10H10O5	209.0456742	Yes
2174	HMDB0127490	3-(3,4-dihydroxy-5-methoxyphenyl)prop-2-enoic acid	C10H10O5	209.0456742	Yes
2174	HMDB0127946	3-(2,4-dihydroxy-5-methoxyphenyl)prop-2-enoic acid	C10H10O5	209.0456742	Yes
2174	HMDB0127947	3-(2,4-dihydroxy-3-methoxyphenyl)prop-2-enoic acid	C10H10O5	209.0456742	Yes
2174	HMDB0132221	3-(2,3-dihydroxy-4-methoxyphenyl)prop-2-enoic acid	C10H10O5	209.0456742	Yes
2174	HMDB0132228	3-(2,5-dihydroxy-4-methoxyphenyl)prop-2-enoic acid	C10H10O5	209.0456742	Yes
2174	HMDB0140909	3-(3-hydroxy-4-methoxyphenyl)oxirane-2-carboxylic ac	C10H10O5	209.0456742	Yes
2174	HMDB0141258	(2Z)-2-hydroxy-3-(4-hydroxy-3-methoxyphenyl)prop-2-	C10H10O5	209.0456742	Yes
2174	HMDB0141331	3-(3,5-dihydroxy-4-methoxyphenyl)prop-2-enoic acid	C10H10O5	209.0456742	Yes
2174	HMDB0142176	2-[(2-hydroxyphenyl)methyl]propanedioic acid	C10H10O5	209.0456742	Yes
2174	HMDB0142177	2-[hydroxy(phenyl)methyl]propanedioic acid	C10H10O5	209.0456742	Yes
2174	HMDB0142178	2-[(4-hydroxyphenyl)methyl]propanedioic acid	C10H10O5	209.0456742	Yes
2174	HMDB0142180	2-benzyl-2-hydroxypropanedioic acid	C10H10O5	209.0456742	Yes
2174	HMDB0142181	2-[(3-hydroxyphenyl)methyl]propanedioic acid	C10H10O5	209.0456742	Yes
2175	HMDB0031813	N-Carbamoyl-2-amino-2-(4-hydroxyphenyl)acetic acid	C9H10N2O4	209.0569617	Yes
2175	HMDB0061683; CHEBI:68589	N-(5-Amino-2-hydroxybenzoyl)glycine	C9H10N2O4	209.0569617	Yes
2177	HMDB0002123; CHEBI:691622	1,3,7-Trimethyluric acid	C8H10N4O3	209.0679976	No
2177	HMDB0036431	1,3,9-Trimethyluric acid	C8H10N4O3	209.0679976	No
2180	HMDB0015441; C07826; CHEBI:2791	Aprobarbital	C10H14N2O3	209.0929773	No
2182	HMDB0032797; C08491; CHEBI:18292	Jasmonic acid	C12H18O3	209.1183296	No
2182	HMDB0032871	Sedanonic acid	C12H18O3	209.1183296	No
2182	HMDB0033102	Dihydro-3-(2-octenyl)-2,5-furandione	C12H18O3	209.1183296	No
2182	HMDB0037104	(R)-8-Acetoxycarvotanacetone	C12H18O3	209.1183296	No
2182	HMDB0037135	1-Benzyloxy-1-(2-methoxyethoxy)ethane	C12H18O3	209.1183296	No
2182	HMDB0037642	4-(Butoxymethyl)-2-methoxyphenol	C12H18O3	209.1183296	No
2182	HMDB0037735	Isoamyl 2-furonpropionate	C12H18O3	209.1183296	No
2182	HMDB0037816	Dihydro-3-(1-octenyl)-2,5-furandione	C12H18O3	209.1183296	No
2182	HMDB0041573	3-Ethenyl-2,5-dimethyl-4-oxohex-5-en-2-yl acetate	C12H18O3	209.1183296	No
2183	HMDB0034276	L,L-Cyclo(leucylprolyl)	C11H18N2O2	209.1299654	No
2184	HMDB0030425	Linalyl propionate	C13H22O2	209.1551076	No
2184	HMDB0031014	Methyl (2E,6Z)-dodecadienoate	C13H22O2	209.1551076	No
2184	HMDB0032052	alpha-Terpineol propanoate	C13H22O2	209.1551076	No
2184	HMDB0032098	Cepanone	C13H22O2	209.1551076	No

2184	HMDB0037286	Citral propylene glycol acetal	C13H22O2	209.1551076	No
2184	HMDB0037307	Propyl 2,4-decadienoate	C13H22O2	209.1551076	No
2184	HMDB0037390	Tsibulin 1	C13H22O2	209.1551076	No
2184	HMDB0038249	Isobornyl propionate	C13H22O2	209.1551076	No
2184	HMDB0038258	Neryl propionate	C13H22O2	209.1551076	No
2184	HMDB0038291	2-Propenyl cyclohexanebutanoate	C13H22O2	209.1551076	No
2184	HMDB0040232	Octahydro-2,5,5,8a-tetramethyl-7H-1-benzopyran-7-or	C13H22O2	209.1551076	No
2184	HMDB0041554	Artemisyl propionate	C13H22O2	209.1551076	No
2196	HMDB0011639; CHEBI:36076	Topaquinone	C9H9NO5	210.0410179	Yes
2196	HMDB0029842; C08538; CHEBI:27483	Betalamic acid	C9H9NO5	210.0410179	Yes
2196	HMDB0034864; C04720; CHEBI:18048	2,4-Dihydroxy-7-methoxy-2H-1,4-benzoxazin-3(4H)-on	C9H9NO5	210.0410179	Yes
2196	HMDB0059999	Gentisuric acid	C9H9NO5	210.0410179	Yes
2196	HMDB0124989	2-{[(3,4-dihydroxyphenyl)(hydroxy)methylidene]aminc	C9H9NO5	210.0410179	Yes
2196	HMDB0126443	2-{[(2,4-dihydroxyphenyl)(hydroxy)methylidene]aminc	C9H9NO5	210.0410179	Yes
2196	HMDB0126654	2-{[(3,5-dihydroxyphenyl)(hydroxy)methylidene]aminc	C9H9NO5	210.0410179	Yes
2196	HMDB0134101	2-{[(2,3-dihydroxyphenyl)(hydroxy)methylidene]aminc	C9H9NO5	210.0410179	Yes
2197	HMDB0030216; CHEBI:715293	Mukonal	C13H9NO2	210.056035	Yes
2198	HMDB0060410; CHEBI:61126	6-Carboxy-5,6,7,8-tetrahydropterin	C7H9N5O3	210.060735	Yes
2199	HMDB0014380; C07224; CHEBI:50693	Milrinone	C12H9N3O	210.066335	Yes
2201	HMDB0001434	3-Methoxytyrosine	C10H13NO4	210.0773637	Yes
2201	HMDB0011754; C07194; CHEBI:61058	Methyldopa	C10H13NO4	210.0773637	Yes
2201	HMDB0060747; CHEBI:82913	3-O-Methyl-a-methyldopa	C10H13NO4	210.0773637	Yes
2202	HMDB0015078; C07207; CHEBI:10101	Zalcitabine	C9H13N3O3	210.0884906	Yes
2203	HMDB0014861; C07513; CHEBI:111775	Methoxamine	C11H17NO3	210.1125129	No
2203	HMDB0014954; C07144; CHEBI:144647	Orciprenaline	C11H17NO3	210.1125129	No
2203	HMDB0015197; C07056; CHEBI:64317	Isoproterenol	C11H17NO3	210.1125129	No
2220	HMDB0015045; C07504; CHEBI:10127	Zonisamide	C8H8N2O3S	211.018618	No
2222	HMDB0013696; CHEBI:1050862	Urolithin B	C13H8O3	211.0395444	Yes
2222	HMDB0032997	2-Hydroxyxanthone	C13H8O3	211.0395444	Yes
2222	HMDB0032999	4-Hydroxyxanthone	C13H8O3	211.0395444	Yes
2225	HMDB0000913; CHEBI:1240674	Vanillactic acid	C10H12O5	211.0614535	Yes
2225	HMDB0031704; C10674	Danielone	C10H12O5	211.0614535	Yes
2225	HMDB0033835; C11155	Propyl gallate	C10H12O5	211.0614535	Yes
2225	HMDB0033839; CHEBI:454991	Eudesmic acid	C10H12O5	211.0614535	Yes
2225	HMDB0034493; C09801	Valtrate	C10H12O5	211.0614535	Yes
2225	HMDB0041286	2,3-Dihydroxy-1-(4-hydroxy-3-methoxyphenyl)-1-propa	C10H12O5	211.0614535	Yes
2225	HMDB0041661	3,4-Dihydroxyphenyllactic acid methyl ester	C10H12O5	211.0614535	Yes
2225	HMDB0041663	3-(3,4-Dihydroxyphenyl)-2-methoxypropionic acid	C10H12O5	211.0614535	Yes
2225	HMDB0041665	3-Hydroxy-4-methoxyphenyllactic acid	C10H12O5	211.0614535	Yes
2225	HMDB0060557	beta-(2-Methoxyphenoxy)-lactic acid	C10H12O5	211.0614535	Yes
2225	HMDB0125013	2-(2,5-dihydroxy-4-methoxyphenyl)propanoic acid	C10H12O5	211.0614535	Yes
2225	HMDB0125520	3-(2,4-dihydroxy-5-methoxyphenyl)propanoic acid	C10H12O5	211.0614535	Yes
2225	HMDB0125526	3-(3,4-dihydroxy-5-methoxyphenyl)propanoic acid	C10H12O5	211.0614535	Yes
2225	HMDB0125540	3-(2,4-dihydroxy-3-methoxyphenyl)propanoic acid	C10H12O5	211.0614535	Yes
2225	HMDB0129354	2-(2,3-dihydroxy-4-methoxyphenyl)propanoic acid	C10H12O5	211.0614535	Yes
2225	HMDB0131424	2-(4-hydroxy-3,5-dimethoxyphenyl)acetic acid	C10H12O5	211.0614535	Yes
2225	HMDB0133486	3-hydroxy-3-(4-hydroxy-3-methoxyphenyl)propanoic a	C10H12O5	211.0614535	Yes
2225	HMDB0142083	2,3,4-trimethoxybenzoic acid	C10H12O5	211.0614535	Yes
2226	HMDB0014814; C12537; CHEBI:41237	Benzyl Benzoate	C14H12O2	211.0771388	No
2226	HMDB0032039; C01408; CHEBI:17682	Benzoin	C14H12O2	211.0771388	No
2226	HMDB0037153	S-2,5-Dimethyl-3-furanyl 3-methylbutanethioate	C11H16O2S	211.0771388	No
2227	HMDB0028843	Glycyl-Histidine	C8H12N4O3	211.0838352	Yes
2227	HMDB0028885	Histidinyl-Glycine	C8H12N4O3	211.0838352	Yes
2228	HMDB0030311; C06538; CHEBI:28121	Harmine	C13H12N2O	211.0904388	Yes
2228	HMDB0032066; CHEBI:41320	Carbanilide	C13H12N2O	211.0904388	Yes
2229	HMDB0029707; C19254	2-Amino-3,4-dimethylimidazo[4,5-f]quinoline	C12H12N4	211.0967159	Yes
2229	HMDB0059744; CHEBI:89902	3,4-Methyleneazelaic acid	C11H16O4	211.0967159	Yes
2232	HMDB0029388	Cucurbic acid	C12H20O3	211.1338448	Yes
2232	HMDB0033601	Dihydrojasmonic acid	C12H20O3	211.1338448	Yes
2232	HMDB0035085	Vulgarole	C12H20O3	211.1338448	Yes
2232	HMDB0037326; C16309; CHEBI:19144	Traumatin	C12H20O3	211.1338448	Yes

2232	HMDB0041572	3-Ethenyl-4-hydroxy-2,5-dimethylhex-5-en-2-yl acetate	C12H20O3	211.1338448	Yes
2233	HMDB0031017	10-Undecenyl acetate	C13H24O2	211.1704971	No
2233	HMDB0031028	Methyl (E)-2-dodecenoate	C13H24O2	211.1704971	No
2233	HMDB0031741	Coniferan	C13H24O2	211.1704971	No
2233	HMDB0032792	(±)-(E)-3-Methyl-4-decen-1-yl acetate	C13H24O2	211.1704971	No
2233	HMDB0034286	Ethyl 10-undecenoate	C13H24O2	211.1704971	No
2233	HMDB0037187	Rhodinyl propionate	C13H24O2	211.1704971	No
2233	HMDB0037226	Citronellyl propionate	C13H24O2	211.1704971	No
2233	HMDB0037626	1-Ethylhexyl tiglate	C13H24O2	211.1704971	No
2233	HMDB0037907	2,6,10,10-Tetramethyl-1-oxaspiro[4.5]decan-6-ol	C13H24O2	211.1704971	No
2233	HMDB0038078	1,1-Diethoxy-2,6-nonadiene	C13H24O2	211.1704971	No
2233	HMDB0039805	4-(3-Hydroxybutyl)-3,3,5-trimethylcyclohexanone	C13H24O2	211.1704971	No
2233	HMDB0040451	Oxacyclotetradecan-2-one	C13H24O2	211.1704971	No
2242	HMDB0012250; C03082	L-Aspartyl-4-phosphate	C4H8NO7P	211.996728	No
2243	HMDB0000682; CHEBI:43355	Indoxyl sulfate	C8H7NO4S	212.0023698	Yes
2246	HMDB0094807	2-nonenoylglycine	C11H19NO3	212.1294527	No
2246	HMDB0094808	3-nonenoylglycine	C11H19NO3	212.1294527	No
2246	HMDB0094809	4-nonenoylglycine	C11H19NO3	212.1294527	No
2246	HMDB0094810	5-nonenoylglycine	C11H19NO3	212.1294527	No
2246	HMDB0094811	6-nonenoylglycine	C11H19NO3	212.1294527	No
2246	HMDB0094812	7-nonenoylglycine	C11H19NO3	212.1294527	No
2246	HMDB0094813	8-nonenoylglycine	C11H19NO3	212.1294527	No
2267	HMDB0006801; C06054; CHEBI:27951	2-Oxo-3-hydroxy-4-phosphobutanoic acid	C4H7O8P	212.9822353	Yes
2271	HMDB0135284	{[(2E)-3-phenylprop-2-en-1-yl]oxy}sulfonic acid	C9H10O4S	213.0223733	Yes
2272	HMDB0014772; CHEBI:63845	Sulfacetamide	C8H10N2O3S	213.0340961	No
2273	HMDB0128186	2-hydroxy-3-(2,4,5-trihydroxyphenyl)propanoic acid	C9H10O6	213.0407769	No
2273	HMDB0135756	2,4-dihydroxy-3,5-dimethoxybenzoic acid	C9H10O6	213.0407769	No
2273	HMDB0141548	2-hydroxy-3-(3,4,5-trihydroxyphenyl)propanoic acid	C9H10O6	213.0407769	No
2274	HMDB0032018; C14163; CHEBI:34918	Phenyl salicylate	C13H10O3	213.0555203	No
2274	HMDB0032583	4''-Hydroxy-2-biphenylcarboxylic acid	C13H10O3	213.0555203	No
2274	HMDB0041807; CHEBI:72631	3-Phenoxybenzoic acid	C13H10O3	213.0555203	No
2274	HMDB0130049	3-[(1E)-buta-1,3-dien-1-yl]-5-hydroxy-1H-isochromen-1	C13H10O3	213.0555203	No
2276	HMDB0004822; CHEBI:89480	Methyl bisnorbiotinyl ketone	C9H14N2O2S	213.0727562	No
2278	HMDB0012111; C16015; CHEBI:50014	(+)-(1R,2R)-1,2-Diphenylethane-1,2-diol	C14H14O2	213.0916647	No
2278	HMDB0029553	(E)-2-(2,4-Hexadiynylidene)-1,6-dioxaspiro[4.5]dec-3-e	C14H14O2	213.0916647	No
2278	HMDB0032709	Ethyl 1-naphthylacetic acid	C14H14O2	213.0916647	No
2278	HMDB0135186	1,2-diphenylethane-1,2-diol	C14H14O2	213.0916647	No
2279	HMDB0030984	5-Hexyltetrahydro-2-oxo-3-furancarboxylic acid	C11H18O4	213.1135613	No
2279	HMDB0030985	alpha-Carboxy-delta-decalactone	C11H18O4	213.1135613	No
2279	HMDB0030986	2-Carboxy-5,7-dimethyl-4-octanolide	C11H18O4	213.1135613	No
2281	HMDB0010727; C02367; CHEBI:18037	3-Oxododecanoic acid	C12H22O3	213.1482777	No
2281	HMDB0037398	4-Acetoxy-2-hexyltetrahydrofuran	C12H22O3	213.1482777	No
2282	HMDB0000910; C17076; CHEBI:45919	Tridecanoic acid	C13H26O2	213.1863241	No
2282	HMDB0029552	Ethyl undecanoate	C13H26O2	213.1863241	No
2282	HMDB0031018; CHEBI:87494	Methyl dodecanoate	C13H26O2	213.1863241	No
2282	HMDB0032211	Decanal propyleneglycol acetal	C13H26O2	213.1863241	No
2282	HMDB0034140	Octyl 3-methylbutanoate	C13H26O2	213.1863241	No
2282	HMDB0036217; C04780; CHEBI:15720	Pentyl octanoate	C13H26O2	213.1863241	No
2282	HMDB0036219	Octyl 2-methylbutyrate	C13H26O2	213.1863241	No
2282	HMDB0037311	Decyl propionate	C13H26O2	213.1863241	No
2282	HMDB0038729; CHEBI:87536	3-Methylbutyl octanoate	C13H26O2	213.1863241	No
2282	HMDB0059900; CHEBI:86973	Propyl decanoate	C13H26O2	213.1863241	No
2282	HMDB0061828	9-Methyldodecanoic acid	C13H26O2	213.1863241	No
2282	HMDB0061829	Isopropyl dodecanoic acid	C13H26O2	213.1863241	No
2298	HMDB0000114; C01233	Glycerylphosphorylethanolamine	C5H14NO6P	214.0487698	No
2298	HMDB0059660; CHEBI:16929	sn-glycero-3-Phosphoethanolamine	C5H14NO6P	214.0487698	No
2305	HMDB0013124; CHEBI:86076	Propenoylcarnitine	C10H17NO4	214.10861	No
2305	HMDB0094732	1-hydroxyoct-2-enoylglycine	C10H17NO4	214.10861	No
2305	HMDB0094733	1-hydroxyoct-3-enoylglycine	C10H17NO4	214.10861	No
2305	HMDB0094734	1-hydroxyoct-4-enoylglycine	C10H17NO4	214.10861	No
2305	HMDB0094735	1-hydroxyoct-5-enoylglycine	C10H17NO4	214.10861	No

2305	HMDB0094736	1-hydroxyoct-6-enoylglycine	C10H17NO4	214.10861	No
2305	HMDB0094737	1-hydroxyoct-7-enoylglycine	C10H17NO4	214.10861	No
2305	HMDB0094738	2-hydroxyoct-1-enoylglycine	C10H17NO4	214.10861	No
2305	HMDB0094739	2-hydroxyoct-2-enoylglycine	C10H17NO4	214.10861	No
2305	HMDB0094740	2-hydroxyoct-3-enoylglycine	C10H17NO4	214.10861	No
2305	HMDB0094741	2-hydroxyoct-4-enoylglycine	C10H17NO4	214.10861	No
2305	HMDB0094742	2-hydroxyoct-5-enoylglycine	C10H17NO4	214.10861	No
2305	HMDB0094743	2-hydroxyoct-6-enoylglycine	C10H17NO4	214.10861	No
2305	HMDB0094744	2-hydroxyoct-7-enoylglycine	C10H17NO4	214.10861	No
2305	HMDB0094745	3-hydroxyoct-1-enoylglycine	C10H17NO4	214.10861	No
2305	HMDB0094746	3-hydroxyoct-2-enoylglycine	C10H17NO4	214.10861	No
2305	HMDB0094747	3-hydroxyoct-3-enoylglycine	C10H17NO4	214.10861	No
2305	HMDB0094748	3-hydroxyoct-4-enoylglycine	C10H17NO4	214.10861	No
2305	HMDB0094749	3-hydroxyoct-5-enoylglycine	C10H17NO4	214.10861	No
2305	HMDB0094750	3-hydroxyoct-6-enoylglycine	C10H17NO4	214.10861	No
2305	HMDB0094751	3-hydroxyoct-7-enoylglycine	C10H17NO4	214.10861	No
2305	HMDB0094752	4-hydroxyoct-1-enoylglycine	C10H17NO4	214.10861	No
2305	HMDB0094753	4-hydroxyoct-2-enoylglycine	C10H17NO4	214.10861	No
2305	HMDB0094754	4-hydroxyoct-3-enoylglycine	C10H17NO4	214.10861	No
2305	HMDB0094755	4-hydroxyoct-4-enoylglycine	C10H17NO4	214.10861	No
2305	HMDB0094756	4-hydroxyoct-5-enoylglycine	C10H17NO4	214.10861	No
2305	HMDB0094757	4-hydroxyoct-6-enoylglycine	C10H17NO4	214.10861	No
2305	HMDB0094758	4-hydroxyoct-7-enoylglycine	C10H17NO4	214.10861	No
2305	HMDB0094759	5-hydroxyoct-1-enoylglycine	C10H17NO4	214.10861	No
2305	HMDB0094760	5-hydroxyoct-2-enoylglycine	C10H17NO4	214.10861	No
2305	HMDB0094761	5-hydroxyoct-3-enoylglycine	C10H17NO4	214.10861	No
2305	HMDB0094762	5-hydroxyoct-4-enoylglycine	C10H17NO4	214.10861	No
2305	HMDB0094763	5-hydroxyoct-5-enoylglycine	C10H17NO4	214.10861	No
2305	HMDB0094764	5-hydroxyoct-6-enoylglycine	C10H17NO4	214.10861	No
2305	HMDB0094765	5-hydroxyoct-7-enoylglycine	C10H17NO4	214.10861	No
2305	HMDB0094766	6-hydroxyoct-1-enoylglycine	C10H17NO4	214.10861	No
2305	HMDB0094767	6-hydroxyoct-2-enoylglycine	C10H17NO4	214.10861	No
2305	HMDB0094768	6-hydroxyoct-3-enoylglycine	C10H17NO4	214.10861	No
2305	HMDB0094769	6-hydroxyoct-4-enoylglycine	C10H17NO4	214.10861	No
2305	HMDB0094770	6-hydroxyoct-5-enoylglycine	C10H17NO4	214.10861	No
2305	HMDB0094771	6-hydroxyoct-6-enoylglycine	C10H17NO4	214.10861	No
2305	HMDB0094772	6-hydroxyoct-7-enoylglycine	C10H17NO4	214.10861	No
2305	HMDB0094773	7-hydroxyoct-1-enoylglycine	C10H17NO4	214.10861	No
2305	HMDB0094774	7-hydroxyoct-2-enoylglycine	C10H17NO4	214.10861	No
2305	HMDB0094775	7-hydroxyoct-3-enoylglycine	C10H17NO4	214.10861	No
2305	HMDB0094776	7-hydroxyoct-4-enoylglycine	C10H17NO4	214.10861	No
2305	HMDB0094777	7-hydroxyoct-5-enoylglycine	C10H17NO4	214.10861	No
2305	HMDB0094778	7-hydroxyoct-6-enoylglycine	C10H17NO4	214.10861	No
2305	HMDB0094779	7-hydroxyoct-7-enoylglycine	C10H17NO4	214.10861	No
2309	HMDB0013279; CHEBI:74439	N-Nonanoylglycine	C11H21NO3	214.1451075	No
2329	HMDB0013989	O-Desmethylnaproxen	C13H12O3	215.0715745	No
2329	HMDB0030647	Artemidinol	C13H12O3	215.0715745	No
2329	HMDB0032035	6-(3,4-Methylenedioxyphenyl)-3,5-hexadien-2-one	C13H12O3	215.0715745	No
2329	HMDB0032656	(Z)-6-(2-Methoxyvinyl)-7-methyl-2H-1-benzopyran-2-o	C13H12O3	215.0715745	No
2329	HMDB0037723	2-Phenylethyl 2-furancarboxylate	C13H12O3	215.0715745	No
2329	HMDB0038017	Ethyl 2-phenyl-3-furancarboxylate	C13H12O3	215.0715745	No
2329	HMDB0130028	3-[(1E)-3-hydroxybut-1-en-1-yl]-1H-isochromen-1-one	C13H12O3	215.0715745	No
2329	HMDB0130029	3-[(1E)-but-1-en-1-yl]-7-hydroxy-1H-isochromen-1-one	C13H12O3	215.0715745	No
2329	HMDB0130030	3-[(1E)-but-1-en-1-yl]-6-hydroxy-1H-isochromen-1-one	C13H12O3	215.0715745	No
2329	HMDB0130031	3-(3-ethyloxiran-2-yl)-1H-isochromen-1-one	C13H12O3	215.0715745	No
2329	HMDB0130032	3-[(1E)-but-1-en-1-yl]-5-hydroxy-1H-isochromen-1-one	C13H12O3	215.0715745	No
2329	HMDB0130033	3-[(1E)-4-hydroxybut-1-en-1-yl]-1H-isochromen-1-one	C13H12O3	215.0715745	No
2332	HMDB0029027	Prolyl-Threonine	C9H16N2O4	215.1038441	Yes
2332	HMDB0029069	Threoninyl-Proline	C9H16N2O4	215.1038441	Yes
2332	HMDB0038239	Sakacin P	C9H16N2O4	215.1038441	Yes
2333	HMDB0029837	Nb-Acetyl-Nb-methyltryptamine	C13H16N2O	215.1210638	Yes

2333	HMDB0034292	Nb-Methyltetrahydroharmol	C13H16N2O	215.1210638	Yes
2333	HMDB0034910	(R)-2-Amino-N-(2,2,4,4-tetramethyl-3-thietanyl)propar	C10H20N2OS	215.1210638	Yes
2334	HMDB0000888; CHEBI:73713	Undecanedioic acid	C11H20O4	215.1290091	Yes
2334	HMDB0037137	Butyl butyryllactate	C11H20O4	215.1290091	Yes
2336	HMDB0029140	Valyl-Valine	C10H20N2O3	215.140246	Yes
2338	HMDB0000387; CHEBI:36206	3-Hydroxydodecanoic acid	C12H24O3	215.1665662	No
2338	HMDB0002059; C08317; CHEBI:39567	12-Hydroxydodecanoic acid	C12H24O3	215.1665662	No
2338	HMDB0010728; CHEBI:43197	(R)-3-Hydroxydodecanoic acid	C12H24O3	215.1665662	No
2338	HMDB0040169	xi-5-Hydroxydodecanoic acid	C12H24O3	215.1665662	No
2356	HMDB0006409; CHEBI:133530	Tyramine-O-sulfate	C8H11NO4S	216.0343398	No
2358	HMDB0015328; CHEBI:3380	Captopril	C9H15NO3S	216.0706014	No
2360	HMDB0038447	1-Isothiocyanato-8-(methylthio)octane	C10H19NS2	216.0880083	Yes
2361	HMDB0000856; C15532; CHEBI:49006	N-a-Acetylcitrulline	C8H15N3O4	216.0992152	No
2361	HMDB0028685	Alanyl-Glutamine	C8H15N3O4	216.0992152	No
2361	HMDB0028701	Alanyl-Gamma-glutamate	C8H15N3O4	216.0992152	No
2361	HMDB0028790	Glutaminylalanine	C8H15N3O4	216.0992152	No
2361	HMDB0029189	5-Hydroxysebacate	C10H17O5	216.0992152	No
2363	HMDB0000824; C03017; CHEBI:28867	Propionylcarnitine	C10H19NO4	216.12427	Yes
2363	HMDB0062514; CHEBI:53210	O-propanoyl-carnitine	C10H19NO4	216.12427	Yes
2380	HMDB0011725; C16199; CHEBI:480324	5-Sulfosalicylic acid	C7H6O6S	216.9813061	Yes
2380	HMDB0059968	3-hydroxybenzoic acid-3-O-sulphate	C7H6O6S	216.9813061	Yes
2380	HMDB0059982; CHEBI:88700	4-hydroxybenzoic acid-4-O-sulphate	C7H6O6S	216.9813061	Yes
2382	HMDB0041785	Tyrosol 4-sulfate	C8H10O5S	217.0175859	Yes
2382	HMDB0124986	(5-ethyl-2-hydroxyphenyl)oxidanesulfonic acid	C8H10O5S	217.0175859	Yes
2387	HMDB0003764; HMDB0028812; CHEBI:7384	Glutamylalanine	C8H14N2O5	217.082561	Yes
2387	HMDB0006248; HMDB0029142; HMDB0061	gamma-Glutamylalanine	C8H14N2O5	217.082561	Yes
2387	HMDB0028872	Hydroxyprolyl-Serine	C8H14N2O5	217.082561	Yes
2387	HMDB0029040	Serinyl-Hydroxyproline	C8H14N2O5	217.082561	Yes
2387	HMDB0036301; CHEBI:50621	N-gamma-L-Glutamyl-D-alanine	C8H14N2O5	217.082561	Yes
2388	HMDB0000350; CHEBI:89182	3-Hydroxysebacic acid	C10H18O5	217.1084562	Yes
2388	HMDB0000424	2-Hydroxydecanedioic acid	C10H18O5	217.1084562	Yes
2399	HMDB0006492; CHEBI:35422	4-Nitrophenyl sulfate	C6H5NO6S	217.9774443	Yes
2407	HMDB0028740	Asparaginyl-Serine	C7H13N3O5	218.0775146	Yes
2407	HMDB0029034	Serinyl-Asparagine	C7H13N3O5	218.0775146	Yes
2407	HMDB0060827	ID11614	C11H13N3S	218.0775146	Yes
2407	HMDB0061056	benzisothiazole piperazine	C11H13N3S	218.0775146	Yes
2408	HMDB0013988; CHEBI:235459	N-Deschlorobenzoyl indomethacin	C12H13NO3	218.0826929	Yes
2408	HMDB0033436	Nigellimine N-oxide	C12H13NO3	218.0826929	Yes
2408	HMDB0038338	alpha-Hydroxy-1-methyl-1H-indole-3-propanoic acid	C12H13NO3	218.0826929	Yes
2408	HMDB0038339	alpha-Methoxy-1H-indole-3-propanoic acid	C12H13NO3	218.0826929	Yes
2409	HMDB0000210; C00864; CHEBI:7916	Pantothenic acid	C9H17NO5	218.1039901	Yes
2409	HMDB0012204; C00371; CHEBI:16522	Cis-zeatin	C10H13N5O	218.1039901	Yes
2409	HMDB0062717; C00864; CHEBI:46905	(R)-pantothenic Acid	C9H17NO5	218.1039901	Yes
2423	HMDB0030789	Dracunculin	C11H8O5	219.0327181	No
2423	HMDB0035086	Buntansin A	C11H8O5	219.0327181	No
2423	HMDB0036346	7-Hydroxy-2-methyl-4-oxo-4H-1-benzopyran-5-carbox	C11H8O5	219.0327181	No
2423	HMDB0038967	1-Propenyl 1-(1-propenylthio)propyl disulfide	C9H16S3	219.0327181	No
2423	HMDB0039820	8-Methoxy-6,7-methylenedioxycoumarin	C11H8O5	219.0327181	No
2423	HMDB0129373	2-(4-methoxy-1-benzofuran-5-yl)-2-oxoacetic acid	C11H8O5	219.0327181	No
2424	HMDB0034857	2-(2-Thienylmethylene)-1,6-dioxaspiro[4.4]non-3-ene	C12H12O2S	219.0461182	No
2424	HMDB0035149	Chrycorin	C12H12O2S	219.0461182	No
2424	HMDB0041942; C11490	N-Acetyl-S-(N-methylcarbamoyl)cysteine	C7H12N2O4S	219.0461182	No
2425	HMDB0059775; CHEBI:89858	3-Hydroxy-3-carboxymethyl-adipic acid	C8H12O7	219.0510522	Yes
2426	HMDB0029467	Eugenitin	C12H12O4	219.0662662	Yes
2426	HMDB0029473	Isoeugenitin	C12H12O4	219.0662662	Yes
2426	HMDB0041442	Hispolone	C12H12O4	219.0662662	Yes
2427	HMDB0000472; C01017; CHEBI:28171	5-Hydroxy-L-tryptophan	C11H12N2O3	219.0774383	Yes
2427	HMDB0015571; C00643; CHEBI:17780	Oxitriptan	C11H12N2O3	219.0774383	Yes
2427	HMDB0061174	p-Hydroxyl-ethotoin	C11H12N2O3	219.0774383	Yes
2428	HMDB0000472; C01017; CHEBI:28171	5-Hydroxy-L-tryptophan	C11H12N2O3	219.0800079	No
2428	HMDB0013139; C14519	1-Hydroxypyrene	C16H12O	219.0800079	No

2428	HMDB0015571; C00643; CHEBI:17780	Oxitriptan	C11H12N2O3	219.0800079	No
2428	HMDB0028693	Alanyl-Methionine	C8H16N2O3S	219.0800079	No
2428	HMDB0028788	Cysteinyl-Valine	C8H16N2O3S	219.0800079	No
2428	HMDB0028966	Methionyl-Alanine	C8H16N2O3S	219.0800079	No
2428	HMDB0029124	Valyl-Cysteine	C8H16N2O3S	219.0800079	No
2428	HMDB0061174	p-Hydroxyl-ethotoin	C11H12N2O3	219.0800079	No
2429	HMDB0060724	2,4-Diamino-6,7-dimethoxyquinazoline	C10H12N4O2	219.0870669	Yes
2430	HMDB0032589	1-(5-Acetyl-2-hydroxyphenyl)-3-methyl-1-butanone	C13H16O3	219.1022084	Yes
2430	HMDB0037492	Ethyl methyl-p-tolylglycidate	C13H16O3	219.1022084	Yes
2430	HMDB0040424	Ethyl 2-benzylacetoacetate	C13H16O3	219.1022084	Yes
2430	HMDB0061823; CHEBI:88591	3-Hexenyl salicylic acid	C13H16O3	219.1022084	Yes
2430	HMDB0061942	cis-3-Hexenyl salicylate	C13H16O3	219.1022084	Yes
2430	HMDB0132933	(1Z)-2-hydroxy-1-(4-methoxyphenyl)-4-methylpent-1-e	C13H16O3	219.1022084	Yes
2430	HMDB0132935	1-(2-hydroxy-4-methoxyphenyl)-4-methylpent-1-en-3-	C13H16O3	219.1022084	Yes
2430	HMDB0132936	1-[3-(4-methoxyphenyl)oxiran-2-yl]-2-methylpropan-1	C13H16O3	219.1022084	Yes
2430	HMDB0132937	5-hydroxy-1-(4-methoxyphenyl)-4-methylpent-1-en-3-	C13H16O3	219.1022084	Yes
2430	HMDB0132939	4-hydroxy-1-(4-methoxyphenyl)-4-methylpent-1-en-3-	C13H16O3	219.1022084	Yes
2430	HMDB0132948	1-(4-methoxyphenyl)-4-methylpentane-2,3-dione	C13H16O3	219.1022084	Yes
2436	HMDB0013688; C17915	Nootkatol	C15H24O	219.1744669	No
2436	HMDB0030232; C09720	(2R,6S,7S,10Z)-beta-Santala-3(15),10-dien-12-ol	C15H24O	219.1744669	No
2436	HMDB0032220	Dihydronootkatone	C15H24O	219.1744669	No
2436	HMDB0033826; C14693; CHEBI:34247	2,6-Di-tert-butyl-4-methylphenol	C15H24O	219.1744669	No
2436	HMDB0034661	alpha-Valerenol	C15H24O	219.1744669	No
2436	HMDB0034718	Isospathulenol	C15H24O	219.1744669	No
2436	HMDB0034940	alpha-Santal-10-en-12-ol	C15H24O	219.1744669	No
2436	HMDB0035020	Acolamone	C15H24O	219.1744669	No
2436	HMDB0035026; C16945	alpha-Cyperol	C15H24O	219.1744669	No
2436	HMDB0035097	beta-Costol	C15H24O	219.1744669	No
2436	HMDB0035306	Epishyobunone	C15H24O	219.1744669	No
2436	HMDB0035391	Preisocalamendiol	C15H24O	219.1744669	No
2436	HMDB0035645	Epoxyguaiene	C15H24O	219.1744669	No
2436	HMDB0035704	Acorenone	C15H24O	219.1744669	No
2436	HMDB0035718; C16974	Isocyperol	C15H24O	219.1744669	No
2436	HMDB0035720	Isoacolamone	C15H24O	219.1744669	No
2436	HMDB0035739	(R)-2,7(14),9-Bisabolatrien-11-ol	C15H24O	219.1744669	No
2436	HMDB0035793	Fukinone	C15H24O	219.1744669	No
2436	HMDB0036118	(3S,6E)-6-Caryophyllen-15-al	C15H24O	219.1744669	No
2436	HMDB0036192	Apritone	C15H24O	219.1744669	No
2436	HMDB0036402	alpha-Bergamotenol	C15H24O	219.1744669	No
2436	HMDB0036420	Spathulenol	C15H24O	219.1744669	No
2436	HMDB0036716; C09720; CHEBI:10441	beta-Santalol	C15H24O	219.1744669	No
2436	HMDB0036717	trans-beta-Santalol	C15H24O	219.1744669	No
2436	HMDB0036788	beta-Betulenol	C15H24O	219.1744669	No
2436	HMDB0036789; C16908	Caryophyllene alpha-oxide	C15H24O	219.1744669	No
2436	HMDB0036793	3,15-Epoxy-6-caryophyllene	C15H24O	219.1744669	No
2436	HMDB0036798	8alpha-3-Copaen-8-ol	C15H24O	219.1744669	No
2436	HMDB0037069	Acoragermacrone	C15H24O	219.1744669	No
2436	HMDB0037392	4(15)-Copaen-11-ol	C15H24O	219.1744669	No
2436	HMDB0037395	11-Copaen-4-ol	C15H24O	219.1744669	No
2436	HMDB0037811	Vetiverol	C15H24O	219.1744669	No
2436	HMDB0038119	Eremofukinone	C15H24O	219.1744669	No
2436	HMDB0038123	1,5-Epoxy-4(14)-salvialene	C15H24O	219.1744669	No
2436	HMDB0038134	Bisabolene oxide	C15H24O	219.1744669	No
2436	HMDB0038146	Calacone	C15H24O	219.1744669	No
2436	HMDB0038160	7(14)-Isodaucen-10-one	C15H24O	219.1744669	No
2436	HMDB0038193	Isoshyobunone	C15H24O	219.1744669	No
2436	HMDB0038208	Humuladienone	C15H24O	219.1744669	No
2436	HMDB0038209	Humulene epoxide I	C15H24O	219.1744669	No
2436	HMDB0038210	Humulene epoxide II	C15H24O	219.1744669	No
2436	HMDB0038211	Humulenol I	C15H24O	219.1744669	No
2436	HMDB0038212	Humulenol II	C15H24O	219.1744669	No

2436	HMDB0038512	Bisacurol	C15H24O	219.1744669	No
2436	HMDB0038982; C14550; CHEBI:34440	4-Nonylphenol	C15H24O	219.1744669	No
2436	HMDB0039532	(6Z,9Z,12Z)-6,9,12-Pentadecatrien-2-one	C15H24O	219.1744669	No
2436	HMDB0039711	Aromadendrene epoxide	C15H24O	219.1744669	No
2436	HMDB0040763	Italicene ether	C15H24O	219.1744669	No
2436	HMDB0059836	Cabreuva oxide D	C15H24O	219.1744669	No
2436	HMDB0060356; CHEBI:15894	2-trans,6-trans-Farnesal	C15H24O	219.1744669	No
2436	HMDB0134152	2-benzyloctan-1-ol	C15H24O	219.1744669	No
2453	HMDB0002135	S-(3-oxo-3-carboxy-n-propyl)cysteine	C7H11NO5S	220.0285316	No
2456	HMDB0038990	Methyl 5-hydroxyoxindole-3-acetate	C11H11NO4	220.0636227	Yes
2456	HMDB0038991	Methyl dioxindole-3-acetate	C11H11NO4	220.0636227	Yes
2456	HMDB0062182	N-lactoyl-Methionine	C8H15NO4S	220.0636227	Yes
2459	HMDB0000389	2''-Deoxysepiapterin	C9H11N5O2	220.084792	Yes
2462	HMDB0030305; C10142	Dioscorine	C13H19NO2	220.1362859	No
2462	HMDB0060850	N,N,O-Tridesmethyl-tramadol	C13H19NO2	220.1362859	No
2476	HMDB0014957; C06805; CHEBI:27690	Acetazolamide	C4H6N4O3S2	220.9813616	Yes
2480	HMDB0128662	3-(6-hydroxy-7-methoxy-2H-1,3-benzodioxol-5-yl)prop	C11H10O5	221.0434554	Yes
2480	HMDB0128663	3-(7-hydroxy-6-methoxy-2H-1,3-benzodioxol-5-yl)prop	C11H10O5	221.0434554	Yes
2480	HMDB0132247	8-hydroxy-6,7-dimethoxy-2H-chromen-2-one	C11H10O5	221.0434554	Yes
2480	HMDB0132248	5-hydroxy-6,7-dimethoxy-2H-chromen-2-one	C11H10O5	221.0434554	Yes
2480	HMDB0136673	6-hydroxy-5,7-dimethoxy-2H-chromen-2-one	C11H10O5	221.0434554	Yes
2481	HMDB0010325; CHEBI:87248	Ethyl glucuronide	C8H14O7	221.0670782	No
2481	HMDB0125202	(3,4,5,6-tetrahydroxyoxan-2-yl)methyl acetate	C8H14O7	221.0670782	No
2481	HMDB0131286	6-ethoxy-3,4,5-trihydroxyoxane-2-carboxylic acid	C8H14O7	221.0670782	No
2513	HMDB0004083; C05645	4-(2-Amino-3-hydroxyphenyl)-2,4-dioxobutanoic acid	C10H9NO5	222.0410817	Yes
2513	HMDB0038941	(R)-2,3-Dihydro-3,5-dihydroxy-2-oxo-3-indoleacetic aci	C10H9NO5	222.0410817	Yes
2513	HMDB0039495; C03147; CHEBI:52430	2-(Malonylamino)benzoic acid	C10H9NO5	222.0410817	Yes
2513	HMDB0129380	2-{[(2H-1,3-benzodioxol-5-yl)(hydroxy)methylidene]am	C10H9NO5	222.0410817	Yes
2518	HMDB0062375; C19602	4-(Methylnitrosamino)-1-(1-oxido-3-pyridinyl)-1-butan	C10H13N3O3	222.0890052	No
2518	HMDB0062382; C19563; CHEBI:82561	4-[(Hydroxymethyl)nitrosoamino]-1-(3-pyridinyl)-1-but	C10H13N3O3	222.0890052	No
2518	HMDB0062404; C19566	4-Hydroxy-4-(methylnitrosoamino)-1-(3-pyridinyl)-1-bu	C10H13N3O3	222.0890052	No
2520	HMDB0015168; C12058; CHEBI:171741	Cerulenin	C12H17NO3	222.1139505	Yes
2521	HMDB0004087; C05201; CHEBI:16804	1-Benzyl-1,2,3,4-tetrahydroisoquinoline	C16H17N	222.1282309	No
2522	HMDB0029828	Dihydrodioscorine	C13H21NO2	222.1501942	No
2522	HMDB0029875; C10868	Tigloidine	C13H21NO2	222.1501942	No
2522	HMDB0030268	Dumetorine	C13H21NO2	222.1501942	No
2536	HMDB0028772	Cysteinyl-Cysteine	C6H12N2O3S2	223.0226226	Yes
2536	HMDB0036314	Dehydrochorismic acid	C10H8O6	223.0226226	Yes
2542	HMDB0000732; C02794; CHEBI:1547	Hydroxykynurenine	C10H12N2O4	223.0716543	No
2542	HMDB0011631; C03227; CHEBI:17380	L-3-Hydroxykynurenine	C10H12N2O4	223.0716543	No
2542	HMDB0012819; C05651; CHEBI:2076	5-Hydroxykynurenine	C10H12N2O4	223.0716543	No
2542	HMDB0014787; C07312; CHEBI:63581	Stavudine	C10H12N2O4	223.0716543	No
2542	HMDB0029397	L-Nicotianine	C10H12N2O4	223.0716543	No
2543	HMDB0004328; CHEBI:336245	Temurin	C9H12N4O3	223.0810528	Yes
2544	HMDB0030379	N-Methoxy-1-vinyl-beta-carboline	C14H12N2O	223.0872528	No
2545	HMDB0030680	Diplosporin	C12H16O4	223.0968423	Yes
2545	HMDB0030703	Dhelwangin	C12H16O4	223.0968423	Yes
2545	HMDB0032552	Vanillin 1,2-butylene glycol acetal	C12H16O4	223.0968423	Yes
2545	HMDB0032663	Acoramone	C12H16O4	223.0968423	Yes
2545	HMDB0036203	2-Ethoxy-4-(4-methyl-1,3-dioxolan-2-yl)phenol	C12H16O4	223.0968423	Yes
2545	HMDB0040629	Isoacoramone	C12H16O4	223.0968423	Yes
2545	HMDB0040890	3'',4'',5''-Trimethoxycinnamyl alcohol	C12H16O4	223.0968423	Yes
2545	HMDB0124917	2-(4-hydroxy-3-methylbut-2-en-1-yl)-5-methoxybenzer	C12H16O4	223.0968423	Yes
2545	HMDB0124919	6-methoxy-3-(3-methylbut-2-en-1-yl)benzene-1,2,4-tri	C12H16O4	223.0968423	Yes
2545	HMDB0124920	2-[(3,3-dimethyloxiran-2-yl)methyl]-5-methoxybenzen	C12H16O4	223.0968423	Yes
2545	HMDB0125801	7-methoxy-2,2-dimethyl-3,4-dihydro-2H-1-benzopyran	C12H16O4	223.0968423	Yes
2545	HMDB0127761	5-(4-hydroxy-3-methoxyphenyl)pentanoic acid	C12H16O4	223.0968423	Yes
2545	HMDB0127765	4-hydroxy-5-(3-methoxyphenyl)pentanoic acid	C12H16O4	223.0968423	Yes
2545	HMDB0135709	4-(4-hydroxy-3,5-dimethoxyphenyl)butan-2-one	C12H16O4	223.0968423	Yes
2546	HMDB0014386; CHEBI:102524	Butalbital	C11H16N2O3	223.108904	No
2547	HMDB0029670	Epoxyeremopetasinorol	C13H20O3	223.1340789	Yes

2547	HMDB0032679	Annuionone A	C13H20O3	223.1340789	Yes
2547	HMDB0032689	Annuionone C	C13H20O3	223.1340789	Yes
2547	HMDB0034564	13-Oxo-9,11-tridecadienoic acid	C13H20O3	223.1340789	Yes
2547	HMDB0036583; C11512; CHEBI:15929	Methyl jasmonate	C13H20O3	223.1340789	Yes
2547	HMDB0037244	(8alpha,10beta,11beta)-3-Hydroxy-4,15-dinor-1(5)-xar	C13H20O3	223.1340789	Yes
2547	HMDB0037726	Octyl 2-furoate	C13H20O3	223.1340789	Yes
2547	HMDB0037730	Furfuryl octanoate	C13H20O3	223.1340789	Yes
2547	HMDB0037736	3-Methylbutyl 2-furanbutanoate	C13H20O3	223.1340789	Yes
2547	HMDB0112084	3-Methyl-5-pentyl-2-furanpropanoic acid	C13H20O3	223.1340789	Yes
2563	HMDB0014752; C07999; CHEBI:5195	Furazolidone	C8H7N3O5	224.0322445	No
2563	HMDB0032558	Dinitolmide	C8H7N3O5	224.0322445	No
2564	HMDB0037264	2-Hydroxy-4,7-dimethoxy-2H-1,4-benzoxazin-3(4H)-on	C10H11NO5	224.0566803	No
2564	HMDB0060026	Vanilloylglycine	C10H11NO5	224.0566803	No
2564	HMDB0128032	2-{[hydroxy(3-hydroxy-5-methoxyphenyl)methylidene]	C10H11NO5	224.0566803	No
2564	HMDB0131194	2-{[hydroxy(2-hydroxy-4-methoxyphenyl)methylidene]	C10H11NO5	224.0566803	No
2564	HMDB0140926	2-{[hydroxy(3-hydroxy-4-methoxyphenyl)methylidene]	C10H11NO5	224.0566803	No
2565	HMDB0029744; CHEBI:581298	Methyl 3-carbazolecarboxylate	C14H11NO2	224.0716563	Yes
2565	HMDB0032983	1-Hydroxy-10-methylacridone	C14H11NO2	224.0716563	Yes
2565	HMDB0038638	6-Methoxy-9H-carbazole-3-carboxaldehyde	C14H11NO2	224.0716563	Yes
2565	HMDB0038849; C09228	Murrayanine	C14H11NO2	224.0716563	Yes
2565	HMDB0041443	Mukolidine	C14H11NO2	224.0716563	Yes
2566	HMDB0014925; C06810; CHEBI:2453	Aciclovir	C8H11N5O3	224.0791146	Yes
2570	HMDB0032141; C03764; CHEBI:17903	4-(Dimethylamino)azobenzene	C14H15N3	224.1194854	No
2570	HMDB0034853; C10914	Cyprodinil	C14H15N3	224.1194854	No
2571	HMDB0015009; C07129; CHEBI:683322	Terbutaline	C12H19NO3	224.1294819	No
2593	HMDB0012199; C00251; CHEBI:17333	Chorismate	C10H10O6	225.0385539	Yes
2593	HMDB0012283; C00254; CHEBI:16666	Prephenate	C10H10O6	225.0385539	Yes
2593	HMDB0033842	3,4-Dimethoxy-1,2-benzenedicarboxylic acid	C10H10O6	225.0385539	Yes
2593	HMDB0033893	4,5-Dimethoxy-1,2-benzenedicarboxylic acid	C10H10O6	225.0385539	Yes
2593	HMDB0125585	3-(2,3,4-trihydroxy-5-methoxyphenyl)prop-2-enoic aci	C10H10O6	225.0385539	Yes
2593	HMDB0125586	3-(2,4,5-trihydroxy-3-methoxyphenyl)prop-2-enoic aci	C10H10O6	225.0385539	Yes
2593	HMDB0125587	3-(3,4-dihydroxy-5-methoxyphenyl)oxirane-2-carboxyl	C10H10O6	225.0385539	Yes
2593	HMDB0126510	3-(3,4-dihydroxy-5-methoxyphenyl)-2-oxopropanoic ac	C10H10O6	225.0385539	Yes
2594	HMDB0032998	2-Methoxyxanthone	C14H10O3	225.0559499	No
2594	HMDB0033925	Oroselone	C14H10O3	225.0559499	No
2595	HMDB0011105; CHEBI:32643	5-Acetylamino-6-formylamino-3-methyluracil	C8H10N4O4	225.0634698	No
2596	HMDB0033251	Phenylmethyl benzeneacetate	C15H14O2	225.0926071	Yes
2596	HMDB0033946	2-Phenylethyl benzoate	C15H14O2	225.0926071	Yes
2596	HMDB0041564	p-Tolyl phenylacetate	C15H14O2	225.0926071	Yes
2596	HMDB0130334	4-[(2E)-3-phenylprop-2-en-1-yl]benzene-1,3-diol	C15H14O2	225.0926071	Yes
2596	HMDB0130984	3-methoxy-5-[(E)-2-phenylethenyl]phenol	C15H14O2	225.0926071	Yes
2596	HMDB0135209	3-hydroxy-1,3-diphenylpropan-1-one	C15H14O2	225.0926071	Yes
2596	HMDB0135210	1-(3-hydroxyphenyl)-3-phenylpropan-1-one	C15H14O2	225.0926071	Yes
2596	HMDB0135211	3-(3-hydroxyphenyl)-1-phenylpropan-1-one	C15H14O2	225.0926071	Yes
2596	HMDB0135212	1-(4-hydroxyphenyl)-3-phenylpropan-1-one	C15H14O2	225.0926071	Yes
2596	HMDB0135213	3-(4-hydroxyphenyl)-1-phenylpropan-1-one	C15H14O2	225.0926071	Yes
2596	HMDB0135214	1-(2-hydroxyphenyl)-3-phenylpropan-1-one	C15H14O2	225.0926071	Yes
2596	HMDB0135215	3-(2-hydroxyphenyl)-1-phenylpropan-1-one	C15H14O2	225.0926071	Yes
2596	HMDB0135540	1-hydroxy-1,3-diphenylpropan-2-one	C15H14O2	225.0926071	Yes
2596	HMDB0135541	1-(3-hydroxyphenyl)-3-phenylpropan-2-one	C15H14O2	225.0926071	Yes
2596	HMDB0135542	1-(4-hydroxyphenyl)-3-phenylpropan-2-one	C15H14O2	225.0926071	Yes
2596	HMDB0135543	1-(2-hydroxyphenyl)-3-phenylpropan-2-one	C15H14O2	225.0926071	Yes
2596	HMDB0135589	4-(3-hydroxy-3-phenylprop-1-en-1-yl)phenol	C15H14O2	225.0926071	Yes
2596	HMDB0141210	3-phenyl-3,4-dihydro-2H-1-benzopyran-7-ol	C15H14O2	225.0926071	Yes
2597	HMDB0040705	Allixin	C12H18O4	225.1128525	Yes
2597	HMDB0059729; CHEBI:89935	3,4-Methylenesebacic acid	C12H18O4	225.1128525	Yes
2598	HMDB0014457; C07422; CHEBI:7983	Pentobarbital	C11H18N2O3	225.1249618	Yes
2598	HMDB0015440; C07536; CHEBI:2673	Amobarbital	C11H18N2O3	225.1249618	Yes
2599	HMDB0031740; CHEBI:89741	Methyl dihydrojasmonate	C13H22O3	225.1500027	No
2599	HMDB0037562	Riesling acetal	C13H22O3	225.1500027	No
2599	HMDB0040615	4,5-Dihydrovomifoliol	C13H22O3	225.1500027	No

2601	HMDB0000499	5-Tetradecenoic acid	C14H26O2	225.1860159	No
2601	HMDB0000521; CHEBI:70716	5Z-Tetradecenoic acid	C14H26O2	225.1860159	No
2601	HMDB0002000; C08322; CHEBI:27781	Myristoleic acid	C14H26O2	225.1860159	No
2601	HMDB0005051	Tsuzuic acid	C14H26O2	225.1860159	No
2601	HMDB0010732; CHEBI:37271	trans-Tetra-dec-2-enoic acid	C14H26O2	225.1860159	No
2601	HMDB0030921	(R)-14-Methyloxacyclotetradecan-2-one	C14H26O2	225.1860159	No
2601	HMDB0031029	Ethyl (E)-2-dodecenoate	C14H26O2	225.1860159	No
2601	HMDB0031073	12-Methyl-13-tridecanolide	C14H26O2	225.1860159	No
2601	HMDB0032152	Acetaldehyde di-cis-3-hexenyl acetal	C14H26O2	225.1860159	No
2601	HMDB0032321	2-Hexenyl octanoate	C14H26O2	225.1860159	No
2601	HMDB0034561; C16947	Houttuynin	C14H26O2	225.1860159	No
2601	HMDB0037188	Rhodinyl butyrate	C14H26O2	225.1860159	No
2601	HMDB0037189	Rhodinyl isobutyrate	C14H26O2	225.1860159	No
2601	HMDB0037227	Citronellyl butyrate	C14H26O2	225.1860159	No
2601	HMDB0037228	Citronellyl isobutyrate	C14H26O2	225.1860159	No
2601	HMDB0037369	Butyl 2-decenoate	C14H26O2	225.1860159	No
2601	HMDB0037633	xi-Tetrahydro-3-nonyl-2H-pyran-2-one	C14H26O2	225.1860159	No
2601	HMDB0038056	delta-Tetradecalactone	C14H26O2	225.1860159	No
2601	HMDB0040228	1,1-Diethoxy-3,7-dimethyl-2,6-octadiene	C14H26O2	225.1860159	No
2601	HMDB0062243; CHEBI:53206	7Z-tetradecenoic acid	C14H26O2	225.1860159	No
2601	HMDB0062248; CHEBI:131381	9E-tetradecenoic acid	C14H26O2	225.1860159	No
2601	HMDB0094671	Heptyl cyclohexane carboxylic acid	C14H26O2	225.1860159	No
2618	HMDB0030218	Lansine	C13H9NO3	226.0505534	No
2618	HMDB0030319	Mukeic acid	C13H9NO3	226.0505534	No
2643	HMDB0001343; C01107; CHEBI:17436	Mevalonic acid-5P	C6H13O7P	227.0298269	No
2644	HMDB0013695; CHEBI:1280327	Urolithin A	C13H8O4	227.0352837	No
2644	HMDB0030723; C10054; CHEBI:411468	1,5-Dihydroxyxanthone	C13H8O4	227.0352837	No
2644	HMDB0030724; C10061; CHEBI:475123	Euxanthone	C13H8O4	227.0352837	No
2644	HMDB0142418	3,9-dihydroxy-6H-benzo[c]chromen-6-one	C13H8O4	227.0352837	No
2644	HMDB0142421	3,4-dihydroxy-6H-benzo[c]chromen-6-one	C13H8O4	227.0352837	No
2644	HMDB0142422	1,3-dihydroxy-6H-benzo[c]chromen-6-one	C13H8O4	227.0352837	No
2646	HMDB0125034	2-(2,3,5-trihydroxy-4-methoxyphenyl)propanoic acid	C10H12O6	227.0563284	Yes
2646	HMDB0125049	2-(2,3,6-trihydroxy-4-methoxyphenyl)propanoic acid	C10H12O6	227.0563284	Yes
2646	HMDB0125592	3-(2,3,4-trihydroxy-5-methoxyphenyl)propanoic acid	C10H12O6	227.0563284	Yes
2646	HMDB0125596	3-(2,4,5-trihydroxy-3-methoxyphenyl)propanoic acid	C10H12O6	227.0563284	Yes
2646	HMDB0141334	2-hydroxy-3,4,5-trimethoxybenzoic acid	C10H12O6	227.0563284	Yes
2646	HMDB0142089	6-hydroxy-2,3,4-trimethoxybenzoic acid	C10H12O6	227.0563284	Yes
2647	HMDB0000012; C00526; CHEBI:16450	Deoxyuridine	C9H12N2O5	227.068287	Yes
2647	HMDB0015023; CHEBI:775149	Pemirolast	C10H8N6O	227.068287	Yes
2649	HMDB0006695; CHEBI:74767	Prolylhydroxyproline	C10H16N2O4	227.1040799	No
2649	HMDB0028871	Hydroxyprolyl-Proline	C10H16N2O4	227.1040799	No
2649	HMDB0029018	Prolyl-Hydroxyproline	C10H16N2O4	227.1040799	No
2649	HMDB0040573	Tetraacetylethylenediamine	C10H16N2O4	227.1040799	No
2649	HMDB0094651; CHEBI:132991	Pyroglutamylvaline	C10H16N2O4	227.1040799	No
2651	HMDB0000933; C16308; CHEBI:545687	Traumatic acid	C12H20O4	227.1293552	Yes
2651	HMDB0030994	5-Heptyltetrahydro-2-oxo-3-furancarboxylic acid	C12H20O4	227.1293552	Yes
2653	HMDB0035881	11-Hydroxy-9-tridecenoic acid	C13H24O3	227.1655073	No
2653	HMDB0036139	Menthone 1,2-glyceryl ketal	C13H24O3	227.1655073	No
2653	HMDB0037212	Menthyl lactate	C13H24O3	227.1655073	No
2653	HMDB0040004	Menthone 1,3-glyceryl ketal	C13H24O3	227.1655073	No
2654	HMDB0000806; C06424; CHEBI:28875	Myristic acid	C14H28O2	227.2018038	No
2654	HMDB0002221	2,6,10-Trimethylundecanoic acid	C14H28O2	227.2018038	No
2654	HMDB0031072; CHEBI:43722	12-Methyltridecanoic acid	C14H28O2	227.2018038	No
2654	HMDB0032318; CHEBI:542954	Hexanal octane-1,3-diol acetal	C14H28O2	227.2018038	No
2654	HMDB0032548	Undecanal propyleneglycol acetal	C14H28O2	227.2018038	No
2654	HMDB0033788; CHEBI:87427	Ethyl dodecanoate	C14H28O2	227.2018038	No
2654	HMDB0034461	Heptyl heptanoate	C14H28O2	227.2018038	No
2654	HMDB0036216; C13798; CHEBI:34491	Hexyl octanoate	C14H28O2	227.2018038	No
2654	HMDB0037312; CHEBI:23580	Decyl butanoate	C14H28O2	227.2018038	No
2654	HMDB0037495	3-Methylbutyl nonanoate	C14H28O2	227.2018038	No
2654	HMDB0038034	Nonyl isovalerate	C14H28O2	227.2018038	No

2654	HMDB0038969	Dodecyl acetate	C14H28O2	227.2018038	No
2654	HMDB0059865; CHEBI:87570	Isobutyl decanoate	C14H28O2	227.2018038	No
2654	HMDB0061781; CHEBI:90062	10-Methyltridecanoic acid	C14H28O2	227.2018038	No
2665	HMDB0014847; C07065; CHEBI:63577	Lamivudine	C8H11N3O3S	228.0448205	No
2673	HMDB0013126	Butenylcarnitine	C11H19NO4	228.1237582	No
2675	HMDB0033531	2,4,6,8-Decatetraenoic acid dehydropiperidide	C15H19NO	228.1395865	No
2675	HMDB0037770	Neopellitorine A	C15H19NO	228.1395865	No
2675	HMDB0039106	2,4-Undecadiene-8,10-diynoic acid isobutylamide	C15H19NO	228.1395865	No
2691	HMDB0000618; C00199; CHEBI:17363	D-Ribulose 5-phosphate	C5H11O8P	229.0093739	No
2691	HMDB0000868; C00231; CHEBI:16332	Xylulose 5-phosphate	C5H11O8P	229.0093739	No
2691	HMDB0001489; C00620; CHEBI:16300	Ribose 1-phosphate	C5H11O8P	229.0093739	No
2691	HMDB0001548; C00117; CHEBI:52742	D-Ribose 5-phosphate	C5H11O8P	229.0093739	No
2691	HMDB0006534; CHEBI:71687	D-Xylulose 1-phosphate	C5H11O8P	229.0093739	No
2691	HMDB0011734; C01112; CHEBI:16241	D-Arabinose 5-phosphate	C5H11O8P	229.0093739	No
2691	HMDB0012195; C03906; CHEBI:15807	Beta-L-arabinose 1-phosphate	C5H11O8P	229.0093739	No
2692	HMDB0127980	(4-ethenyl-2-methoxyphenyl)oxidanesulfonic acid	C9H10O5S	229.0178394	Yes
2692	HMDB0134048	{[3-(2-hydroxyphenyl)prop-2-en-1-yl]oxy}sulfonic acid	C9H10O5S	229.0178394	Yes
2692	HMDB0135258	[2-hydroxy-5-(prop-2-en-1-yl)phenyl]oxidanesulfonic a	C9H10O5S	229.0178394	Yes
2692	HMDB0135295	[(1-oxo-3-phenylpropan-2-yl)oxy]sulfonic acid	C9H10O5S	229.0178394	Yes
2692	HMDB0135297	(3-oxo-1-phenylpropoxy)sulfonic acid	C9H10O5S	229.0178394	Yes
2692	HMDB0135299	[3-(3-oxopropyl)phenyl]oxidanesulfonic acid	C9H10O5S	229.0178394	Yes
2692	HMDB0135301	[4-(3-oxopropyl)phenyl]oxidanesulfonic acid	C9H10O5S	229.0178394	Yes
2692	HMDB0135303	{3-[(1E)-3-hydroxyprop-1-en-1-yl]phenyl}oxidanesulfor	C9H10O5S	229.0178394	Yes
2692	HMDB0135304	{[(2E)-3-(3-hydroxyphenyl)prop-2-en-1-yl]oxy}sulfonic	C9H10O5S	229.0178394	Yes
2692	HMDB0135306	{4-[(1E)-3-hydroxyprop-1-en-1-yl]phenyl}oxidanesulfor	C9H10O5S	229.0178394	Yes
2692	HMDB0135307	{[(2E)-3-(4-hydroxyphenyl)prop-2-en-1-yl]oxy}sulfonic	C9H10O5S	229.0178394	Yes
2692	HMDB0135311	[(3-phenyloxiran-2-yl)methoxy]sulfonic acid	C9H10O5S	229.0178394	Yes
2692	HMDB0135656	[4-(3-hydroxyprop-1-en-1-yl)phenyl]oxidanesulfonic ac	C9H10O5S	229.0178394	Yes
2692	HMDB0135657	{[3-(4-hydroxyphenyl)prop-2-en-1-yl]oxy}sulfonic acid	C9H10O5S	229.0178394	Yes
2695	HMDB0039670	3,3''-Dithiobis[4,5-dihydro-2-methylfuran]	C10H14O2S2	229.0362623	No
2696	HMDB0062720; CHEBI:82911	Thymol Sulfate	C10H14O4S	229.0543441	Yes
2696	HMDB0133674	(2-methyl-3-phenylpropoxy)sulfonic acid	C10H14O4S	229.0543441	Yes
2698	HMDB0001923; C01517; CHEBI:7476	Naproxen	C14H14O3	229.0868968	Yes
2698	HMDB0033184	Pondaplin	C14H14O3	229.0868968	Yes
2698	HMDB0034130; C18080	Osthenol	C14H14O3	229.0868968	Yes
2698	HMDB0034212; C09947	(R)-Kawain	C14H14O3	229.0868968	Yes
2698	HMDB0039097; C10253	Demethylbatatasin IV	C14H14O3	229.0868968	Yes
2698	HMDB0129921	5-[2-(3-hydroxyphenyl)ethyl]benzene-1,3-diol	C14H14O3	229.0868968	Yes
2698	HMDB0131063	7-hydroxy-3-(2-methylbut-3-en-2-yl)-2H-chromen-2-or	C14H14O3	229.0868968	Yes
2698	HMDB0142155	1-(2-hydroxyphenyl)-2-phenylethane-1,2-diol	C14H14O3	229.0868968	Yes
2698	HMDB0142156	1-(3-hydroxyphenyl)-2-phenylethane-1,2-diol	C14H14O3	229.0868968	Yes
2698	HMDB0142157	1-(4-hydroxyphenyl)-2-phenylethane-1,2-diol	C14H14O3	229.0868968	Yes
2700	HMDB0037942	(1xi,3xi)-1,2,3,4-Tetrahydro-1-methyl-beta-carboline-3	C13H14N2O2	229.0977242	No
2700	HMDB0060811	cyclic Melatonin	C13H14N2O2	229.0977242	No
2701	HMDB0041448	Dicyclohexyl disulfide	C12H22S2	229.1085319	Yes
2702	HMDB0028876	Hydroxyprolyl-Valine	C10H18N2O4	229.1197539	Yes
2702	HMDB0029128	Valyl-Hydroxyproline	C10H18N2O4	229.1197539	Yes
2704	HMDB0000623; C02678; CHEBI:4676	Dodecanedioic acid	C12H22O4	229.1431515	Yes
2704	HMDB0030143	Talaromycin A	C12H22O4	229.1431515	Yes
2704	HMDB0041613; C14523	Bis(1-methylethyl) hexanedioate	C12H22O4	229.1431515	Yes
2704	HMDB0041614	Dipropyl hexanedioate	C12H22O4	229.1431515	Yes
2705	HMDB0028920	Isoleucyl-Valine	C11H22N2O3	229.1556753	Yes
2705	HMDB0028942	Leucyl-Valine	C11H22N2O3	229.1556753	Yes
2705	HMDB0029130	Valyl-Isoleucine	C11H22N2O3	229.1556753	Yes
2705	HMDB0029131	Valyl-Leucine	C11H22N2O3	229.1556753	Yes
2706	HMDB0036133	3-[[5-Methyl-2-(1-methylethyl)cyclohexyl]oxy]-1,2-pro	C13H26O3	229.1807337	No
2706	HMDB0061655	3-hydroxytridecanoic acid	C13H26O3	229.1807337	No
2717	HMDB0059911; CHEBI:32635	Paracetamol sulfate	C8H9NO5S	230.013503	No
2717	HMDB0059994; CHEBI:88751	Benzeneacetamide-4-O-sulphate	C8H9NO5S	230.013503	No
2718	HMDB0028744	Asparaginyl-Valine	C9H17N3O4	230.1150178	Yes
2718	HMDB0029122	Valyl-Asparagine	C9H17N3O4	230.1150178	Yes

2720	HMDB0001959; CHEBI:89308	Gamma-Aminobutyryl-lysine	C10H21N3O3	230.1500701	No
2755	HMDB0041646	2,4-Dihydroxyacetophenone 5-sulfate	C8H8O6S	230.9972104	Yes
2755	HMDB0041789	Vanillin 4-sulfate	C8H8O6S	230.9972104	Yes
2755	HMDB0125163	2-[3-(sulfooxy)phenyl]acetic acid	C8H8O6S	230.9972104	Yes
2755	HMDB0128010	(4-ethenyl-2,6-dihydroxyphenyl)oxidanesulfonic acid	C8H8O6S	230.9972104	Yes
2755	HMDB0132500	2-[4-(sulfooxy)phenyl]acetic acid	C8H8O6S	230.9972104	Yes
2757	HMDB0030813; CHEBI:549602	Cassiachromone	C13H12O4	231.0662012	No
2757	HMDB0033330	Dihydrocoriandrin	C13H12O4	231.0662012	No
2757	HMDB0038911	(2S,3S,4S)-5,7,9,11-Tridecatetrayne-1,2,3,4-tetrol	C13H12O4	231.0662012	No
2757	HMDB0041325	Salfredin B11	C13H12O4	231.0662012	No
2757	HMDB0128959	7-hydroxy-6-(3-oxobutyl)-2H-chromen-2-one	C13H12O4	231.0662012	No
2757	HMDB0130044	5-hydroxy-3-[(1E)-3-hydroxybut-1-en-1-yl]-1H-isochror	C13H12O4	231.0662012	No
2757	HMDB0130045	3-[(1E)-but-1-en-1-yl]-5,6-dihydroxy-1H-isochromen-1-	C13H12O4	231.0662012	No
2757	HMDB0130046	3-[(1E)-but-1-en-1-yl]-5,7-dihydroxy-1H-isochromen-1-	C13H12O4	231.0662012	No
2757	HMDB0130047	3-(3-ethyloxiran-2-yl)-5-hydroxy-1H-isochromen-1-one	C13H12O4	231.0662012	No
2757	HMDB0130048	5-hydroxy-3-[(1E)-4-hydroxybut-1-en-1-yl]-1H-isochror	C13H12O4	231.0662012	No
2757	HMDB0130065	3-(1,2-dihydroxybut-3-en-1-yl)-1H-isochromen-1-one	C13H12O4	231.0662012	No
2757	HMDB0135794	7-(hydroxymethyl)-6-[(E)-2-methoxyethenyl]-2H-chron	C13H12O4	231.0662012	No
2757	HMDB0135795	6-(3-methoxyoxiran-2-yl)-7-methyl-2H-chromen-2-one	C13H12O4	231.0662012	No
2757	HMDB0135796	8-hydroxy-6-[(E)-2-methoxyethenyl]-7-methyl-2H-chro	C13H12O4	231.0662012	No
2757	HMDB0135797	5-hydroxy-6-[(E)-2-methoxyethenyl]-7-methyl-2H-chro	C13H12O4	231.0662012	No
2757	HMDB0135798	3-hydroxy-6-[(E)-2-methoxyethenyl]-7-methyl-2H-chro	C13H12O4	231.0662012	No
2758	HMDB0014917; C05079; CHEBI:100147	Nalidixic Acid	C12H12N2O3	231.0780012	No
2758	HMDB0015305; CHEBI:8069	Phenobarbital	C12H12N2O3	231.0780012	No
2759	HMDB0031640	Glycerol 1-propanoate diacetate	C10H16O6	231.0868708	Yes
2760	HMDB0001199; C03415; CHEBI:27574	N2-Succinyl-L-ornithine	C9H16N2O5	231.0990294	No
2760	HMDB0012161; C15767; CHEBI:49260	4-(Glutamylamino) butanoate	C9H16N2O5	231.0990294	No
2760	HMDB0028766	Aspartyl-Valine	C9H16N2O5	231.0990294	No
2760	HMDB0028873	Hydroxyprolyl-Threonine	C9H16N2O5	231.0990294	No
2760	HMDB0029062	Threoninyl-Hydroxyproline	C9H16N2O5	231.0990294	No
2760	HMDB0029123	Valyl-Aspartate	C9H16N2O5	231.0990294	No
2760	HMDB0029393; CHEBI:102630	L-N-(3-Carboxypropyl)glutamine	C9H16N2O5	231.0990294	No
2761	HMDB0001389; C01598; CHEBI:16796	Melatonin	C13H16N2O2	231.1128613	No
2761	HMDB0014501; C07617; CHEBI:2654	Aminoglutethimide	C13H16N2O2	231.1128613	No
2764	HMDB0013075	Spermic acid 2	C10H20N2O4	231.1354314	No
2764	HMDB0028917	Isoleucyl-Threonine	C10H20N2O4	231.1354314	No
2764	HMDB0028939	Leucyl-Threonine	C10H20N2O4	231.1354314	No
2764	HMDB0029064	Threoninyl-Isoleucine	C10H20N2O4	231.1354314	No
2764	HMDB0029065	Threoninyl-Leucine	C10H20N2O4	231.1354314	No
2788	HMDB0029038	Serinyl-Glutamate	C8H13N2O6	232.0696217	No
2790	HMDB0035923; CHEBI:91152	Hirsutin	C10H19NOS2	232.083836	No
2791	HMDB0139876	3-(4-hydroxyphenyl)-N-(4-oxobutyl)prop-2-enimidic ac	C13H15NO3	232.099853	No
2792	HMDB0040033	2-Heptylbenzothiazole	C14H19NS	232.1157619	No
2793	HMDB0028960	Lysyl-Serine	C9H19N3O4	232.1298606	No
2793	HMDB0029044	Serinyl-Lysine	C9H19N3O4	232.1298606	No
2793	HMDB0029447	Lysinoalanine	C9H19N3O4	232.1298606	No
2795	HMDB0011140	Hypusine	C10H23N3O3	232.1679446	No
2812	HMDB0033566; C16757	Ajoene	C9H14OS3	233.0125445	Yes
2812	HMDB0128030	(4-ethyl-2,6-dihydroxyphenyl)oxidanesulfonic acid	C8H10O6S	233.0125445	Yes
2816	HMDB0032438	1-Isopropyl citrate	C9H14O7	233.0656682	Yes
2816	HMDB0032660	(2Z,4''Z)-2-(5-Methylthio-4-penten-2-ynylidene)-1,6-di	C13H14O2S	233.0656682	Yes
2816	HMDB0038083	2-Isopropyl citrate	C9H14O7	233.0656682	Yes
2817	HMDB0011169	L-beta-aspartyl-L-threonine	C8H14N2O6	233.0807682	No
2817	HMDB0028763	Aspartyl-Threonine	C8H14N2O6	233.0807682	No
2817	HMDB0028828; CHEBI:73509	Glutamylserine	C8H14N2O6	233.0807682	No
2817	HMDB0029057	Threoninyl-Aspartate	C8H14N2O6	233.0807682	No
2817	HMDB0029158	gamma-Glutamylserine	C8H14N2O6	233.0807682	No
2817	HMDB0030486	(S)-2,3-Dihydro-6-hydroxy-5-(hydroxyacetyl)-2-isoprop	C13H14O4	233.0807682	No
2817	HMDB0030648	Artemidiol	C13H14O4	233.0807682	No
2817	HMDB0033109	Methyl (2R*,3S*)-2,3-dihydro-3-hydroxy-2-isopropeny	C13H14O4	233.0807682	No
2817	HMDB0035712	Aloesol	C13H14O4	233.0807682	No

2817	HMDB0040906; C17917; CHEBI:81380	Coixinden B	C13H14O4	233.0807682	No
2817	HMDB0041005	1''-Acetoxychavicol acetate	C13H14O4	233.0807682	No
2817	HMDB0041006; C10426; CHEBI:469	1''-Acetoxychavicol	C13H14O4	233.0807682	No
2819	HMDB0030763	(10S,11S)-Pterosin C	C14H18O3	233.1183592	No
2819	HMDB0036046	Dehydrooreadone	C14H18O3	233.1183592	No
2819	HMDB0036607	(S)-Pterosin P	C14H18O3	233.1183592	No
2819	HMDB0038218	Pterosin N	C14H18O3	233.1183592	No
2819	HMDB0133171	(2Z)-3-hydroxy-2-(phenylmethylidene)heptanoic acid	C14H18O3	233.1183592	No
2819	HMDB0133172	(2Z)-2-[(3-hydroxyphenyl)methylidene]heptanoic acid	C14H18O3	233.1183592	No
2819	HMDB0133173	(2Z)-2-[(4-hydroxyphenyl)methylidene]heptanoic acid	C14H18O3	233.1183592	No
2819	HMDB0133174	(2Z)-6-hydroxy-2-(phenylmethylidene)heptanoic acid	C14H18O3	233.1183592	No
2819	HMDB0133175	2-pentyl-3-phenyloxirane-2-carboxylic acid	C14H18O3	233.1183592	No
2819	HMDB0133176	(2Z)-7-hydroxy-2-(phenylmethylidene)heptanoic acid	C14H18O3	233.1183592	No
2819	HMDB0133177	(2Z)-2-[(2-hydroxyphenyl)methylidene]heptanoic acid	C14H18O3	233.1183592	No
2820	HMDB0033461	4-Coumaroylputrescine	C13H18N2O2	233.129497	No
2820	HMDB0139874	N-(4-aminobutyl)-3-(4-hydroxyphenyl)prop-2-enimidic	C13H18N2O2	233.129497	No
2822	HMDB0030016; C09743	Valerenic acid	C15H22O2	233.1548196	No
2822	HMDB0031349	Epiacoronene	C15H22O2	233.1548196	No
2822	HMDB0032081	4,10-Longipinanedione	C15H22O2	233.1548196	No
2822	HMDB0033252	Curcumanolide A	C15H22O2	233.1548196	No
2822	HMDB0033253; C17492	Curcumenone	C15H22O2	233.1548196	No
2822	HMDB0033960; C16942; CHEBI:80811	Curcumenol	C15H22O2	233.1548196	No
2822	HMDB0034562	9-Pentadecene-12,14-diyne-1,11-diol	C15H22O2	233.1548196	No
2822	HMDB0034742	Saussurea lactone	C15H22O2	233.1548196	No
2822	HMDB0034956	Oxysolavetivone	C15H22O2	233.1548196	No
2822	HMDB0035202	7-Hydroxycostal	C15H22O2	233.1548196	No
2822	HMDB0035407; C16949	Dehydrocurdione	C15H22O2	233.1548196	No
2822	HMDB0035408	1,10-Epoxygermacrone	C15H22O2	233.1548196	No
2822	HMDB0035413	(4alpha,5alpha)-11-Eremophilene-2,9-dione	C15H22O2	233.1548196	No
2822	HMDB0035639	Petasalbin	C15H22O2	233.1548196	No
2822	HMDB0035794	beta-Costic acid	C15H22O2	233.1548196	No
2822	HMDB0035869	alpha-Rotunol	C15H22O2	233.1548196	No
2822	HMDB0035889; C17489	Germacrone 4,5-epoxide	C15H22O2	233.1548196	No
2822	HMDB0035935	Dihydroisoalantolactone	C15H22O2	233.1548196	No
2822	HMDB0036035	Marasmene	C15H22O2	233.1548196	No
2822	HMDB0036390	3-Phenylpropyl hexanoate	C15H22O2	233.1548196	No
2822	HMDB0036392	(Z)-alpha-Bergamotenoic acid	C15H22O2	233.1548196	No
2822	HMDB0036427	Procurcumenol	C15H22O2	233.1548196	No
2822	HMDB0036449	(1alpha,4beta,5beta)-4-Hydroxy-7(11),10(14)-guaiadie	C15H22O2	233.1548196	No
2822	HMDB0036466	Zerumbone oxide	C15H22O2	233.1548196	No
2822	HMDB0036647	Isocurcumenol	C15H22O2	233.1548196	No
2822	HMDB0037038	Cyclodehydroisolubimin	C15H22O2	233.1548196	No
2822	HMDB0037391	p-Hydroxynonanophenone	C15H22O2	233.1548196	No
2822	HMDB0037640	2-Methyl-4-phenyl-2-butyl 2-methylpropanoate	C15H22O2	233.1548196	No
2822	HMDB0037710	4-Methylphenyl octanoate	C15H22O2	233.1548196	No
2822	HMDB0037739	7-(1,3-Cyclohexadienyl)-5-hydroxy-2,6-dimethyl-2-hep	C15H22O2	233.1548196	No
2822	HMDB0038190	Rishitinol	C15H22O2	233.1548196	No
2822	HMDB0038198	3,7-Bisaboladiene-2,8-dione	C15H22O2	233.1548196	No
2822	HMDB0038698	xi-2-Hydroxy-1,3,5-bisabolatrien-9-one	C15H22O2	233.1548196	No
2822	HMDB0038795	7,9-Illudadiene-3,14-diol	C15H22O2	233.1548196	No
2822	HMDB0038796	2,7(14)-Illudadiene-10,15-diol	C15H22O2	233.1548196	No
2822	HMDB0038914	4,6-Decadiyn-1-ol isovalerate	C15H22O2	233.1548196	No
2822	HMDB0039621	beta-Santalic acid	C15H22O2	233.1548196	No
2822	HMDB0041036; C17504	Sugeonol	C15H22O2	233.1548196	No
2822	HMDB0134131	2-[hydroxy(phenyl)methyl]octanal	C15H22O2	233.1548196	No
2822	HMDB0134132	2-[(3-hydroxyphenyl)methyl]octanal	C15H22O2	233.1548196	No
2822	HMDB0134133	2-[(4-hydroxyphenyl)methyl]octanal	C15H22O2	233.1548196	No
2822	HMDB0134134	2-benzyl-7-hydroxyoctanal	C15H22O2	233.1548196	No
2822	HMDB0134135	2-benzyl-8-hydroxyoctanal	C15H22O2	233.1548196	No
2822	HMDB0134136	2-benzyl-2-hydroxyoctanal	C15H22O2	233.1548196	No
2822	HMDB0134137	2-[(2-hydroxyphenyl)methyl]octanal	C15H22O2	233.1548196	No

2822	HMDB0134138	2-(phenylmethylidene)octane-1,3-diol	C15H22O2	233.1548196	No
2822	HMDB0134139	3-[(1Z)-2-hexyl-3-hydroxyprop-1-en-1-yl]phenol	C15H22O2	233.1548196	No
2822	HMDB0134140	4-[(1Z)-2-hexyl-3-hydroxyprop-1-en-1-yl]phenol	C15H22O2	233.1548196	No
2822	HMDB0134141	(2Z)-2-(phenylmethylidene)octane-1,7-diol	C15H22O2	233.1548196	No
2822	HMDB0134142	(2-hexyl-3-phenyloxiran-2-yl)methanol	C15H22O2	233.1548196	No
2822	HMDB0134143	(2Z)-2-(phenylmethylidene)octane-1,8-diol	C15H22O2	233.1548196	No
2822	HMDB0134153	2-benzyloctanoic acid	C15H22O2	233.1548196	No
2839	HMDB0013204	6-Succinoaminopurine	C9H9N5O3	234.063768	No
2845	HMDB0060851	N,O-Didesmethyltramadol	C14H21NO2	234.1497497	No
2863	HMDB0028750	Aspartyl-Cysteine	C7H12N2O5S	235.039875	Yes
2863	HMDB0028771	Cysteinyl-Aspartate	C7H12N2O5S	235.039875	Yes
2865	HMDB0001200; C02406; CHEBI:18377	N''-Formylkynurenine	C11H12N2O4	235.0726739	No
2865	HMDB0060485; C02700; CHEBI:30249	L-Formylkynurenine	C11H12N2O4	235.0726739	No
2866	HMDB0037683	Ethyl vanillin isobutyrate	C13H16O4	235.0980711	Yes
2866	HMDB0060564; CHEBI:133199	Carboxy-ibuprofen	C13H16O4	235.0980711	Yes
2866	HMDB0124833	4-hydroxy-2-methoxy-3-(3-methylbut-2-en-1-yl)benzoi	C13H16O4	235.0980711	Yes
2866	HMDB0135908	2-hydroxy-2-[4-hydroxy-3-(3-methylbut-2-en-1-yl)pher	C13H16O4	235.0980711	Yes
2866	HMDB0140425	4-hydroxy-3-methoxy-5-(3-methylbut-2-en-1-yl)benzoi	C13H16O4	235.0980711	Yes
2868	HMDB0034462; C14718; CHEBI:17879	Heptyl 4-hydroxybenzoate	C14H20O3	235.1332752	No
2868	HMDB0040778	Eremopetasidione	C14H20O3	235.1332752	No
2869	HMDB0014859; C07375; CHEBI:8430	Procaine	C13H20N2O2	235.1450745	Yes
2869	HMDB0139525	N-(4-aminobutyl)-3-(4-hydroxyphenyl)propanimidic ac	C13H20N2O2	235.1450745	Yes
2870	HMDB0036468	1,5,5,8-Tetramethyl-12-thiabicyclo[9.1.0]dodeca-3,7-d	C15H24S	235.1530502	No
2870	HMDB0036469	3,7,7,10-Tetramethyl-12-thiabicyclo[9.1.0]dodeca-3,7-	C15H24S	235.1530502	No
2870	HMDB0038151	Mintsulfide	C15H24S	235.1530502	No
2871	HMDB0002352; C09627; CHEBI:28283	Capsidiol	C15H24O2	235.1702352	No
2871	HMDB0030891	(2E,6E)-1-Hydroxy-2,6,10-farnesatrien-9-one	C15H24O2	235.1702352	No
2871	HMDB0030916	2-Hydroxyacorenone	C15H24O2	235.1702352	No
2871	HMDB0030920	Acorusnol	C15H24O2	235.1702352	No
2871	HMDB0031348	Epiacorone	C15H24O2	235.1702352	No
2871	HMDB0032048	2,6-Di-tert-butyl-4-hydroxymethylphenol	C15H24O2	235.1702352	No
2871	HMDB0032292; CHEBI:545749	Geranyl tiglate	C15H24O2	235.1702352	No
2871	HMDB0032681	10alpha-4,5-Seco-11-eudesmene-4,5-dione	C15H24O2	235.1702352	No
2871	HMDB0033083	(4S)-Dihydrocurcumenone	C15H24O2	235.1702352	No
2871	HMDB0033104	Calamensesquiterpinenol	C15H24O2	235.1702352	No
2871	HMDB0034714; C17493	Curdione	C15H24O2	235.1702352	No
2871	HMDB0034722	3-Hydroxy-1,10-bisaboladien-9-one	C15H24O2	235.1702352	No
2871	HMDB0035032	Epilubimin	C15H24O2	235.1702352	No
2871	HMDB0035040	7-Hydroxycostol	C15H24O2	235.1702352	No
2871	HMDB0035127	(-)-Myrtenyl isovalerate	C15H24O2	235.1702352	No
2871	HMDB0035147	Rishitinone	C15H24O2	235.1702352	No
2871	HMDB0035301	4alpha,5alpha-Epoxy-11-eudesmen-3a-ol	C15H24O2	235.1702352	No
2871	HMDB0035648	Isolubimin	C15H24O2	235.1702352	No
2871	HMDB0035827	Aubergenone	C15H24O2	235.1702352	No
2871	HMDB0036446	beta-Kessyl ketone	C15H24O2	235.1702352	No
2871	HMDB0036549	Apotrichothecene	C15H24O2	235.1702352	No
2871	HMDB0036668	Humulene-8-hydroperoxide	C15H24O2	235.1702352	No
2871	HMDB0036680; C16946	Cyperolone	C15H24O2	235.1702352	No
2871	HMDB0036687	Tanavulgarol	C15H24O2	235.1702352	No
2871	HMDB0037393	(3xi,7(11)Z)-7(11)-Copaene-4,12-diol	C15H24O2	235.1702352	No
2871	HMDB0037906	Hernandulcin	C15H24O2	235.1702352	No
2871	HMDB0038027	beta-Ionyl acetate	C15H24O2	235.1702352	No
2871	HMDB0038122	Curcumol	C15H24O2	235.1702352	No
2871	HMDB0038144	Petasitolone	C15H24O2	235.1702352	No
2871	HMDB0038178	Davanone	C15H24O2	235.1702352	No
2871	HMDB0039525	Dihydro-alpha-santalic acid	C15H24O2	235.1702352	No
2871	HMDB0039688	Tuberonone	C15H24O2	235.1702352	No
2871	HMDB0040712	Humulene diepoxide A	C15H24O2	235.1702352	No
2887	HMDB0034251	N-Benzoylaspartic acid	C11H11NO5	236.0590286	Yes
2887	HMDB0038942	Methyl 2,3-dihydro-3,5-dihydroxy-2-oxo-3-indoleaceti	C11H11NO5	236.0590286	Yes
2890	HMDB0013067	Salsoline-1-carboxylate	C12H15NO4	236.0922087	Yes

2890	HMDB0032775	3-Hydroxy-carbofuran	C12H15NO4	236.0922087	Yes
2890	HMDB0062175	N-lactoyl-Phenylalanine	C12H15NO4	236.0922087	Yes
2890	HMDB0139939	3-(3,4-dihydroxyphenyl)-N-(3-oxopropyl)propanimidic	C12H15NO4	236.0922087	Yes
2918	HMDB0014836; CHEBI:1039679	Nitrofurantoin	C8H6N4O5	237.0266663	No
2920	HMDB0033073	1-Propenyl 1-(propylsulfinyl)propyl disulfide	C9H18OS3	237.0424096	No
2920	HMDB0033074	1-(1-Propenylsulfinyl)propyl propyl disulfide	C9H18OS3	237.0424096	No
2920	HMDB0036370	1-(2-Propenylsulfinyl)propyl propyl disulfide	C9H18OS3	237.0424096	No
2920	HMDB0036375	2-Propenyl 1-(propylsulfinyl)propyl disulfide	C9H18OS3	237.0424096	No
2920	HMDB0039494	Benzoylmalic acid	C11H10O6	237.0424096	No
2920	HMDB0128679	3-(6-hydroxy-7-methoxy-2H-1,3-benzodioxol-5-yl)prop	C11H10O6	237.0424096	No
2920	HMDB0128680	3-(7-hydroxy-6-methoxy-2H-1,3-benzodioxol-5-yl)prop	C11H10O6	237.0424096	No
2922	HMDB0002399	Cystathionine sulfoxide	C7H14N2O5S	237.053762	No
2922	HMDB0031816; C01495; CHEBI:5078	3-Hydroxyflavone	C15H10O3	237.053762	No
2922	HMDB0130492	7-hydroxy-3-phenyl-4H-chromen-4-one	C15H10O3	237.053762	No
2922	HMDB0137519	6-hydroxy-2-(phenylmethylidene)-2,3-dihydro-1-benzo	C15H10O3	237.053762	No
2922	HMDB0141529	2-(2-hydroxyphenyl)-4H-chromen-4-one	C15H10O3	237.053762	No
2922	HMDB0141530	6-hydroxy-2-phenyl-4H-chromen-4-one	C15H10O3	237.053762	No
2922	HMDB0141531	7-hydroxy-2-phenyl-4H-chromen-4-one	C15H10O3	237.053762	No
2922	HMDB0141532	2-(3-hydroxyphenyl)-4H-chromen-4-one	C15H10O3	237.053762	No
2922	HMDB0141533	2-(4-hydroxyphenyl)-4H-chromen-4-one	C15H10O3	237.053762	No
2922	HMDB0141534	5-hydroxy-2-phenyl-4H-chromen-4-one	C15H10O3	237.053762	No
2923	HMDB0131294	3,4,5-trihydroxy-6-(2-hydroxyethoxy)oxane-2-carboxyl	C8H14O8	237.061862	No
2924	HMDB0002511	3,4,5-Trimethoxycinnamic acid	C12H14O5	237.0768782	Yes
2924	HMDB0011721	Trans-2, 3, 4-Trimethoxycinnamate	C12H14O5	237.0768782	Yes
2924	HMDB0031771	1-(2,4,5-Trimethoxyphenyl)-1,2-propanedione	C12H14O5	237.0768782	Yes
2924	HMDB0124869	3-(3,4,5-trimethoxyphenyl)prop-2-enoic acid	C12H14O5	237.0768782	Yes
2924	HMDB0128658	3-(6,7-dimethoxy-2H-1,3-benzodioxol-5-yl)propanal	C12H14O5	237.0768782	Yes
2924	HMDB0128659	3-(6,7-dimethoxy-2H-1,3-benzodioxol-5-yl)prop-2-en-1	C12H14O5	237.0768782	Yes
2924	HMDB0128697	3-(4-hydroxy-2,3,5-trimethoxyphenyl)prop-2-enal	C12H14O5	237.0768782	Yes
2925	HMDB0015084; C07501; CHEBI:4995	Felbamate	C11H14N2O4	237.0861021	No
2925	HMDB0028853	Glycyl-Tyrosine	C11H14N2O4	237.0861021	No
2925	HMDB0029105	Tyrosyl-Glycine	C11H14N2O4	237.0861021	No
2925	HMDB0029451	gamma-Glutaminyl-4-hydroxybenzene	C11H14N2O4	237.0861021	No
2925	HMDB0040988	Falimint	C11H14N2O4	237.0861021	No
2926	HMDB0029700; CHEBI:724725	Cinnamyl benzoate	C16H14O2	237.0920021	No
2926	HMDB0032585	4''-Methoxychalcone	C16H14O2	237.0920021	No
2926	HMDB0040286	Benzyl cinnamate	C16H14O2	237.0920021	No
2926	HMDB0062295; CHEBI:42334	15(R)-hydroperoxy-EPE	C8H18N2O4S	237.0920021	No
2927	HMDB0033065	(x)-1,2-Propanediol 1-O-b-D-glucopyranoside	C9H18O7	237.0980006	No
2929	HMDB0014562; CHEBI:9073	Secobarbital	C12H18N2O3	237.124898	No
2929	HMDB0139940	N-(3-aminopropyl)-3-(3,4-dihydroxyphenyl)propanimic	C12H18N2O3	237.124898	No
2931	HMDB0038256	Geranyl acetoacetate	C14H22O3	237.1492653	No
2931	HMDB0112083	3,4-Dimethyl-5-pentyl-2-furanpropanoic acid	C14H22O3	237.1492653	No
2932	HMDB0029350	Geranyl 3-methylbutanoate	C15H26O2	237.1860885	No
2932	HMDB0030428	Linalyl isovalerate	C15H26O2	237.1860885	No
2932	HMDB0031383	(3S,4S,6R,7S)-1,10-Bisaboladiene-3,4-diol	C15H26O2	237.1860885	No
2932	HMDB0031384	beta-Kessyl alcohol	C15H26O2	237.1860885	No
2932	HMDB0032054	alpha-Terpinyl pentanoate	C15H26O2	237.1860885	No
2932	HMDB0032209	Citronellyl trans-2-methyl-2-butenoate	C15H26O2	237.1860885	No
2932	HMDB0032291	Geranyl 2-methylbutyrate	C15H26O2	237.1860885	No
2932	HMDB0032343	Isobornyl 2-methylbutyrate	C15H26O2	237.1860885	No
2932	HMDB0033228	Guaidiol	C15H26O2	237.1860885	No
2932	HMDB0034673	Calamendiol	C15H26O2	237.1860885	No
2932	HMDB0035287; C09652	Daucol	C15H26O2	237.1860885	No
2932	HMDB0035887	Germacrenone	C15H26O2	237.1860885	No
2932	HMDB0036032	3beta-7-Drimene-3,11-diol	C15H26O2	237.1860885	No
2932	HMDB0036730	Tricyclohumuladiol	C15H26O2	237.1860885	No
2932	HMDB0036880	1,6,9-Farnesatriene-3,11-diol	C15H26O2	237.1860885	No
2932	HMDB0037195	2-Propenyl cyclohexanehexanoate	C15H26O2	237.1860885	No
2932	HMDB0037279	2,10-Bisaboladiene-1,4-diol	C15H26O2	237.1860885	No
2932	HMDB0037284	5(1->10)-Abeo-1,12-patchoulanediol	C15H26O2	237.1860885	No

2932	HMDB0038136	Curcumadiol	C15H26O2	237.1860885	No
2932	HMDB0038138	alpha-Bisabolol oxide C	C15H26O2	237.1860885	No
2932	HMDB0038196; C16773	alpha-Bisabolol oxide A	C15H26O2	237.1860885	No
2932	HMDB0038197; C16774; CHEBI:80720	alpha-Bisabolol oxide B	C15H26O2	237.1860885	No
2932	HMDB0038247	Bornyl valerate	C15H26O2	237.1860885	No
2932	HMDB0038248	Bornyl isovalerate	C15H26O2	237.1860885	No
2932	HMDB0038250	Isobornyl isovalerate	C15H26O2	237.1860885	No
2932	HMDB0039130	Methyl (Z,Z)-5,8-tetradecadienoate	C15H26O2	237.1860885	No
2932	HMDB0039745	(2E,6E,9xi)-Farnesol	C15H26O2	237.1860885	No
2932	HMDB0040273	Terpenyl isovalerate	C15H26O2	237.1860885	No
2932	HMDB0040281	Geranyl valerate	C15H26O2	237.1860885	No
2932	HMDB0041589	alpha-Kessyl alcohol	C15H26O2	237.1860885	No
2933	HMDB0034181	3-Methylcyclopentadecanone	C16H30O	237.2228095	No
2933	HMDB0037544	(±)-2-Dodecylcyclobutanone	C16H30O	237.2228095	No
2933	HMDB0060482; CHEBI:17585	Hexadecenal	C16H30O	237.2228095	No
2952	HMDB0060805	9-Carboxymethoxymethylguanine	C8H9N5O4	238.058373	No
2956	HMDB0033525	2,4-Undecadiene-8,10-diynoic acid 2,3-dehydropiperid	C16H17NO	238.1244227	No
2958	HMDB0001937; C11770; CHEBI:8746	Salbutamol	C13H21NO3	238.144864	No
2958	HMDB0014366; C07053; CHEBI:775164	Isoetharine	C13H21NO3	238.144864	No
2958	HMDB0030516	Petasinine	C13H21NO3	238.144864	No
2958	HMDB0033454	3alpha-Tigloyloxytropane N-oxide	C13H21NO3	238.144864	No
2958	HMDB0041935	Moprolol	C13H21NO3	238.144864	No
2977	HMDB0128682	3-(6-hydroxy-7-methoxy-2H-1,3-benzodioxol-5-yl)prop	C11H12O6	239.0563309	No
2977	HMDB0128684	3-(7-hydroxy-6-methoxy-2H-1,3-benzodioxol-5-yl)prop	C11H12O6	239.0563309	No
2977	HMDB0132101	2-benzyl-2,3-dihydroxybutanedioic acid	C11H12O6	239.0563309	No
2977	HMDB0135751	3-(2,4-dihydroxy-3,5-dimethoxyphenyl)prop-2-enoic ac	C11H12O6	239.0563309	No
2977	HMDB0135752	3-(4-hydroxy-3,5-dimethoxyphenyl)oxirane-2-carboxyl	C11H12O6	239.0563309	No
2978	HMDB0030254; CHEBI:583580	3-(3,4,5-Trimethoxyphenyl)propanoic acid	C12H16O5	239.093021	Yes
2978	HMDB0038627	Ethyl 3,4,5-trimethoxybenzoate	C12H16O5	239.093021	Yes
2978	HMDB0041756	Isopropyl 3-(3,4-dihydroxyphenyl)-2-hydroxypropanoa	C12H16O5	239.093021	Yes
2978	HMDB0061112; HMDB0062776; CHEBI:4125	3-Carboxy-4-methyl-5-propyl-2-furanpropionic acid	C12H16O5	239.093021	Yes
2978	HMDB0125802	7-methoxy-2,2-dimethyl-3,4-dihydro-2H-1-benzopyran	C12H16O5	239.093021	Yes
2978	HMDB0125804	2-(hydroxymethyl)-7-methoxy-2-methyl-3,4-dihydro-2	C12H16O5	239.093021	Yes
2978	HMDB0127748	4-hydroxy-5-(4-hydroxy-3-methoxyphenyl)pentanoic a	C12H16O5	239.093021	Yes
2978	HMDB0127826	4-hydroxy-5-(3-hydroxy-4-methoxyphenyl)pentanoic a	C12H16O5	239.093021	Yes
2978	HMDB0128672	3-(6,7-dimethoxy-2H-1,3-benzodioxol-5-yl)propan-1-ol	C12H16O5	239.093021	Yes
2978	HMDB0128705	3-(4-hydroxy-2,3,5-trimethoxyphenyl)propanal	C12H16O5	239.093021	Yes
2978	HMDB0128721	4-(3-hydroxyprop-1-en-1-yl)-2,3,6-trimethoxyphenol	C12H16O5	239.093021	Yes
2978	HMDB0142074	3-(2,3,4-trimethoxyphenyl)propanoic acid	C12H16O5	239.093021	Yes
2979	HMDB0000194; C01262; CHEBI:18323	Anserine	C10H16N4O3	239.115147	Yes
2979	HMDB0000745; C00884; CHEBI:491179	Homocarnosine	C10H16N4O3	239.115147	Yes
2979	HMDB0005769	Balenine	C10H16N4O3	239.115147	Yes
2983	HMDB0032369	L-Menthyl acetoacetate	C14H24O3	239.1660436	Yes
2985	HMDB0029765	9-Pentadecenoic acid	C15H28O2	239.2018971	No
2985	HMDB0032347; CHEBI:46643	Isobutyl 10-undecenoate	C15H28O2	239.2018971	No
2985	HMDB0034455	Exaltolide	C15H28O2	239.2018971	No
2985	HMDB0036426	Butyl undecylenate	C15H28O2	239.2018971	No
2985	HMDB0037190	Rhodinyl isovalerate	C15H28O2	239.2018971	No
2985	HMDB0037211	Menthyl isovalerate	C15H28O2	239.2018971	No
2985	HMDB0037229	Citronellyl pentanoate	C15H28O2	239.2018971	No
2985	HMDB0038954	Citronellyl isovalerate	C15H28O2	239.2018971	No
2985	HMDB0039715	Auberganol	C15H28O2	239.2018971	No
2985	HMDB0040997	15,5-Farnesanolide	C15H28O2	239.2018971	No
2987	HMDB0001551; C00517; CHEBI:17600	Palmitaldehyde	C16H32O	239.2377895	No
2987	HMDB0031052	2-Hexadecanone	C16H32O	239.2377895	No
2998	HMDB0060002; CHEBI:89621	Indole-3-carboxilic acid-O-sulphate	C9H7NO5S	239.9975208	Yes
3001	HMDB0033527	Dehydroisochalciporone	C16H19NO	240.1396306	No
3001	HMDB0060535	N-Desmethyldiphenhydramine	C16H19NO	240.1396306	No
3001	HMDB0061165	N,N-Didemethyl orphenadrine	C16H19NO	240.1396306	No
3002	HMDB0038588	Dioscoretine	C13H23NO3	240.1587557	No
3018	HMDB0029953	D-erythro-D-galacto-octitol	C8H18O8	241.0907529	No

3022	HMDB0010730; CHEBI:37270	3-Oxotetradecanoic acid	C14H26O3	241.1819031	No
3022	HMDB0030982	6-Ketomyristic acid	C14H26O3	241.1819031	No
3022	HMDB0032370	L-Menthyl (R,S)-3-hydroxybutyrate	C14H26O3	241.1819031	No
3024	HMDB0000826; C16537	Pentadecanoic acid	C15H30O2	241.2174439	No
3024	HMDB0030469; CHEBI:447965	Methyl tetradecanoate	C15H30O2	241.2174439	No
3024	HMDB0032250	Dodecyl propionate	C15H30O2	241.2174439	No
3024	HMDB0032328; CHEBI:32361	Hexyl nonanoate	C15H30O2	241.2174439	No
3024	HMDB0036218	Heptyl octanoate	C15H30O2	241.2174439	No
3024	HMDB0036220	Octyl heptanoate	C15H30O2	241.2174439	No
3024	HMDB0037306	Pentyl decanoate	C15H30O2	241.2174439	No
3024	HMDB0038907	3-Methylbutyl decanoate	C15H30O2	241.2174439	No
3024	HMDB0040998	7(14)-Farnesene-9,12-diol	C15H30O2	241.2174439	No
3024	HMDB0041588	2,6,10-Trimethyldodecanoic acid	C15H30O2	241.2174439	No
3024	HMDB0059820	Isopropyl_laurate	C15H30O2	241.2174439	No
3024	HMDB0059833	Ethyl tridecanoate	C15H30O2	241.2174439	No
3024	HMDB0061707; CHEBI:39250	13-Methylmyristic acid	C15H30O2	241.2174439	No
3044	HMDB0000089; C00475; CHEBI:17562	Cytidine	C9H13N3O5	242.0800096	Yes
3044	HMDB0015122; C02961; CHEBI:28680	Cytarabine	C9H13N3O5	242.0800096	Yes
3044	HMDB0060478; C05711; CHEBI:10565	gamma-Glutamyl-beta-cyanoalanine	C9H13N3O5	242.0800096	Yes
3047	HMDB0028805	Glutaminylproline	C10H17N3O4	242.1136293	Yes
3047	HMDB0029015	Prolyl-Glutamine	C10H17N3O4	242.1136293	Yes
3047	HMDB0029031	Prolyl-Gamma-glutamate	C10H17N3O4	242.1136293	Yes
3049	HMDB0015133; CHEBI:350328	Frovatriptan	C14H17N3O	242.1301394	Yes
3050	HMDB0028959	Lysyl-Proline	C11H21N3O3	242.1489394	No
3050	HMDB0029022	Prolyl-Lysine	C11H21N3O3	242.1489394	No
3051	HMDB0033526	Chalciporone	C16H21NO	242.1549883	No
3052	HMDB0013286; CHEBI:74438	N-Undecanoylglycine	C13H25NO3	242.176781	No
3072	HMDB0124975	(2E)-3-[3-(sulfooxy)phenyl]prop-2-enoic acid	C9H8O6S	242.9970342	Yes
3072	HMDB0125166	(2E)-3-[4-(sulfooxy)phenyl]prop-2-enoic acid	C9H8O6S	242.9970342	Yes
3072	HMDB0127985	3-[3-(sulfooxy)phenyl]prop-2-enoic acid	C9H8O6S	242.9970342	Yes
3072	HMDB0128093	3-[4-(sulfooxy)phenyl]prop-2-enoic acid	C9H8O6S	242.9970342	Yes
3072	HMDB0134054	[2-hydroxy-3-(3-oxoprop-1-en-1-yl)phenyl]oxidanesulf	C9H8O6S	242.9970342	Yes
3076	HMDB0059983; CHEBI:88699	4-phenylbutanic acid-O-sulphate	C10H12O5S	243.0331643	No
3076	HMDB0133637	(2-benzyl-3-oxopropoxy)sulfonic acid	C10H12O5S	243.0331643	No
3076	HMDB0133640	(2-methyl-3-oxo-1-phenylpropoxy)sulfonic acid	C10H12O5S	243.0331643	No
3076	HMDB0133642	[3-(2-methyl-3-oxopropyl)phenyl]oxidanesulfonic acid	C10H12O5S	243.0331643	No
3076	HMDB0133644	[4-(2-methyl-3-oxopropyl)phenyl]oxidanesulfonic acid	C10H12O5S	243.0331643	No
3076	HMDB0133646	[(2E)-3-hydroxy-2-(phenylmethylidene)propoxy]sulfon	C10H12O5S	243.0331643	No
3076	HMDB0133648	{3-[(1E)-3-hydroxy-2-methylprop-1-en-1-yl]phenyl}oxid	C10H12O5S	243.0331643	No
3076	HMDB0133649	{[(2E)-3-(3-hydroxyphenyl)-2-methylprop-2-en-1-yl]oxy	C10H12O5S	243.0331643	No
3076	HMDB0133651	{4-[(1E)-3-hydroxy-2-methylprop-1-en-1-yl]phenyl}oxid	C10H12O5S	243.0331643	No
3076	HMDB0133652	{[(2E)-3-(4-hydroxyphenyl)-2-methylprop-2-en-1-yl]oxy	C10H12O5S	243.0331643	No
3076	HMDB0133656	[(2-methyl-3-phenyloxiran-2-yl)methoxy]sulfonic acid	C10H12O5S	243.0331643	No
3076	HMDB0133658	{[(2E)-3-(2-hydroxyphenyl)-2-methylprop-2-en-1-yl]oxy	C10H12O5S	243.0331643	No
3076	HMDB0133734	{[(3E)-2-hydroxy-4-phenylbut-3-en-1-yl]oxy}sulfonic ac	C10H12O5S	243.0331643	No
3076	HMDB0133735	{[(3E)-1-hydroxy-4-phenylbut-3-en-2-yl]oxy}sulfonic ac	C10H12O5S	243.0331643	No
3076	HMDB0133737	{3-[(1E)-3-hydroxybut-1-en-1-yl]phenyl}oxidanesulfoni	C10H12O5S	243.0331643	No
3076	HMDB0133738	{[(3E)-4-(3-hydroxyphenyl)but-3-en-2-yl]oxy}sulfonic a	C10H12O5S	243.0331643	No
3076	HMDB0133740	{4-[(1E)-3-hydroxybut-1-en-1-yl]phenyl}oxidanesulfoni	C10H12O5S	243.0331643	No
3076	HMDB0133741	{[(3E)-4-(4-hydroxyphenyl)but-3-en-2-yl]oxy}sulfonic a	C10H12O5S	243.0331643	No
3076	HMDB0133745	[1-(3-phenyloxiran-2-yl)ethoxy]sulfonic acid	C10H12O5S	243.0331643	No
3076	HMDB0133766	[(3-oxo-1-phenylbutan-2-yl)oxy]sulfonic acid	C10H12O5S	243.0331643	No
3076	HMDB0133768	(2-oxo-4-phenylbutoxy)sulfonic acid	C10H12O5S	243.0331643	No
3076	HMDB0133770	(3-oxo-1-phenylbutoxy)sulfonic acid	C10H12O5S	243.0331643	No
3076	HMDB0133772	[3-(3-oxobutyl)phenyl]oxidanesulfonic acid	C10H12O5S	243.0331643	No
3076	HMDB0133774	[4-(3-oxobutyl)phenyl]oxidanesulfonic acid	C10H12O5S	243.0331643	No
3076	HMDB0135245	[2-methoxy-4-(prop-2-en-1-yl)phenyl]oxidanesulfonic a	C10H12O5S	243.0331643	No
3076	HMDB0135653	{[3-(4-methoxyphenyl)prop-2-en-1-yl]oxy}sulfonic acid	C10H12O5S	243.0331643	No
3078	HMDB0039177	1-O-Galloylglycerol	C10H12O7	243.0530025	Yes
3079	HMDB0000296; C00299; CHEBI:16704	Uridine	C9H12N2O6	243.0626163	Yes
3079	HMDB0000767; C02067; CHEBI:17802	Pseudouridine	C9H12N2O6	243.0626163	Yes

3080	HMDB0006005	Indolylacryloylglycine	C13H12N2O3	243.0774132	No
3080	HMDB0040734	Haematopodin	C13H12N2O3	243.0774132	No
3081	HMDB0031237	threo-Syringoylglycerol	C11H16O6	243.0871883	Yes
3082	HMDB0014336; CHEBI:3395	Carbidopa	C10H16N2O5	243.0984127	Yes
3082	HMDB0028827; CHEBI:73508	Glutamylproline	C10H16N2O5	243.0984127	Yes
3082	HMDB0028864	Hydroxyprolyl-Hydroxyproline	C10H16N2O5	243.0984127	Yes
3082	HMDB0029157	gamma-Glutamylproline	C10H16N2O5	243.0984127	Yes
3082	HMDB0029439	(2S,4S)-Pinnatanine	C10H16N2O5	243.0984127	Yes
3084	HMDB0014437; C07522; CHEBI:4910	Etomidate	C14H16N2O2	243.1112031	Yes
3085	HMDB0032472	Polyethylene, oxidized	C12H20O5	243.1242472	Yes
3086	HMDB0028866	Hydroxyprolyl-Isoleucine	C11H20N2O4	243.1352339	Yes
3086	HMDB0028867	Hydroxyprolyl-Leucine	C11H20N2O4	243.1352339	Yes
3086	HMDB0028908	Isoleucyl-Hydroxyproline	C11H20N2O4	243.1352339	Yes
3086	HMDB0028930	Leucyl-Hydroxyproline	C11H20N2O4	243.1352339	Yes
3088	HMDB0028910	Isoleucyl-Isoleucine	C12H24N2O3	243.1711957	No
3088	HMDB0028911	Isoleucyl-Leucine	C12H24N2O3	243.1711957	No
3088	HMDB0028932	Leucyl-Isoleucine	C12H24N2O3	243.1711957	No
3088	HMDB0028933; CHEBI:6418	Leucyl-Leucine	C12H24N2O3	243.1711957	No
3089	HMDB0002261; CHEBI:59270	2-Hydroxymyristic acid	C14H28O3	243.1963815	No
3089	HMDB0010731; CHEBI:42539	(R)-3-Hydroxy-tetradecanoic acid	C14H28O3	243.1963815	No
3089	HMDB0032417	3-(L-Menthoxy)-2-methylpropane-1,2-diol	C14H28O3	243.1963815	No
3089	HMDB0059850	Ethyl 3-hydroxydodecanoate	C14H28O3	243.1963815	No
3089	HMDB0061656; CHEBI:85148	3-hydroxytetradecanoic acid	C14H28O3	243.1963815	No
3117	HMDB0028732	Asparaginyl-Hydroxyproline	C9H15N3O5	244.094454	Yes
3117	HMDB0028858	Hydroxyprolyl-Asparagine	C9H15N3O5	244.094454	Yes
3121	HMDB0059773; CHEBI:89859	S-3-oxodecanoyl cysteamine	C12H23NO2S	244.1382426	Yes
3122	HMDB0060815	Desmethyl frovatriptan	C14H19N3O	244.1457488	No
3123	HMDB0000378; CHEBI:73026	2-Methylbutyroylcarnitine	C12H23NO4	244.1549867	Yes
3123	HMDB0000688; CHEBI:73025	Isovalerylcarnitine	C12H23NO4	244.1549867	Yes
3123	HMDB0013128	Valerylcarnitine	C12H23NO4	244.1549867	Yes
3123	HMDB0041993	Pivaloylcarnitine	C12H23NO4	244.1549867	Yes
3137	HMDB0032822	5-Sulfo-1,3-benzenedicarboxylic acid	C8H6O7S	244.977229	Yes
3140	HMDB0094710; CHEBI:64414	3-[3-(Sulfooxy)phenyl]propanoic acid	C9H10O6S	245.0130299	Yes
3140	HMDB0125171	3-[4-(sulfooxy)phenyl]propanoic acid	C9H10O6S	245.0130299	Yes
3140	HMDB0128001	(4-ethenyl-2-hydroxy-6-methoxyphenyl)oxidanesulfoni	C9H10O6S	245.0130299	Yes
3140	HMDB0130016	2-[4-(sulfooxy)phenyl]propanoic acid	C9H10O6S	245.0130299	Yes
3140	HMDB0134064	[2-hydroxy-3-(3-oxopropyl)phenyl]oxidanesulfonic acic	C9H10O6S	245.0130299	Yes
3140	HMDB0134069	{[1-(2-hydroxyphenyl)-3-oxopropan-2-yl]oxy}sulfonic a	C9H10O6S	245.0130299	Yes
3140	HMDB0134071	[1-(2-hydroxyphenyl)-3-oxopropoxy]sulfonic acid	C9H10O6S	245.0130299	Yes
3140	HMDB0134074	[2-hydroxy-3-(3-hydroxyprop-1-en-1-yl)phenyl]oxidane	C9H10O6S	245.0130299	Yes
3140	HMDB0134080	{[3-(2-hydroxyphenyl)oxiran-2-yl]methoxy}sulfonic acic	C9H10O6S	245.0130299	Yes
3140	HMDB0134083	{[3-(2,5-dihydroxyphenyl)prop-2-en-1-yl]oxy}sulfonic a	C9H10O6S	245.0130299	Yes
3143	HMDB0135750	[3-(4-methoxyphenyl)propoxy]sulfonic acid	C10H14O5S	245.0491872	Yes
3145	HMDB0000497; CHEBI:23774	5,6-Dihydrouridine	C9H14N2O6	245.0781043	Yes
3145	HMDB0011160	L-alpha-Aspartyl-L-hydroxyproline	C9H14N2O6	245.0781043	Yes
3145	HMDB0028754	Aspartyl-Hydroxyproline	C9H14N2O6	245.0781043	Yes
3146	HMDB0013713; CHEBI:74640	N-acetyltryptophan	C13H14N2O3	245.0933478	No
3146	HMDB0014987; C07829; CHEBI:6758	Methylphenobarbital	C13H14N2O3	245.0933478	No
3146	HMDB0030269	Nigellicine	C13H14N2O3	245.0933478	No
3146	HMDB0060810	cyclic 6-Hydroxymelatonin	C13H14N2O3	245.0933478	No
3148	HMDB0011166	L-beta-aspartyl-L-leucine	C10H18N2O5	245.1147443	Yes
3148	HMDB0011172; HMDB0029162; CHEBI:6884	gamma-Glutamylvaline	C10H18N2O5	245.1147443	Yes
3148	HMDB0028756	Aspartyl-Isoleucine	C10H18N2O5	245.1147443	Yes
3148	HMDB0028757	Aspartyl-Leucine	C10H18N2O5	245.1147443	Yes
3148	HMDB0028832; HMDB0059717; CHEBI:6884	Glutamylvaline	C10H18N2O5	245.1147443	Yes
3148	HMDB0028903	Isoleucyl-Aspartate	C10H18N2O5	245.1147443	Yes
3148	HMDB0028925	Leucyl-Aspartate	C10H18N2O5	245.1147443	Yes
3150	HMDB0061115; C09213	Lenticin	C14H18N2O2	245.129667	Yes
3151	HMDB0000413	3-Hydroxydodecanedioic acid	C12H22O5	245.1396595	Yes
3151	HMDB0031696	Dibutyl malate	C12H22O5	245.1396595	Yes
3152	HMDB0036207	alpha-Amylcinnamyl acetate	C16H22O2	245.1543259	Yes

3152	HMDB0037705	Heptyl cinnamate	C16H22O2	245.1543259	Yes
3152	HMDB0041250	Demethoxyshogaol	C16H22O2	245.1543259	Yes
3153	HMDB0015096; C07528; CHEBI:6759	Mepivacaine	C15H22N2O	245.1660192	No
3153	HMDB0015602; CHEBI:521102	Milnacipran	C15H22N2O	245.1660192	No
3165	HMDB0062547; CHEBI:82942	2-Hydroxyacetaminophen sulfate	C8H9NO6S	246.0083	Yes
3167	HMDB0128628	2-{[hydroxy(1-oxo-1H-isochromen-3-yl)methylidene]ar	C12H9NO5	246.0397526	Yes
3172	HMDB0029060	Threoninyl-Glutamate	C9H15N2O6	246.0875497	Yes
3173	HMDB0002095; CHEBI:86047	Malonylcarnitine	C10H17NO6	246.0977028	Yes
3173	HMDB0033699; C01594; CHEBI:16441	Linamarin	C10H17NO6	246.0977028	Yes
3173	HMDB0062592; CHEBI:85470	O-malonyl-L-carnitine	C10H17NO6	246.0977028	Yes
3193	HMDB0041788; CHEBI:88847	Vanillic acid 4-sulfate	C8H8O7S	246.9921269	Yes
3193	HMDB0125151	2-[4-hydroxy-3-(sulfooxy)phenyl]acetic acid	C8H8O7S	246.9921269	Yes
3193	HMDB0140929	4-methoxy-3-(sulfooxy)benzoic acid	C8H8O7S	246.9921269	Yes
3196	HMDB0029229	4-Methoxyphenylethanol sulfate	C9H12O6S	247.0280911	Yes
3196	HMDB0128019	(4-ethyl-2-hydroxy-6-methoxyphenyl)oxidanesulfonic a	C9H12O6S	247.0280911	Yes
3198	HMDB0004185; C05832	5-Hydroxyindoleacetylglycine	C12H12N2O4	247.0721473	No
3198	HMDB0060537	p-Hydroxyphenobarbital	C12H12N2O4	247.0721473	No
3198	HMDB0060826	Hydroxynalidixic acid	C12H12N2O4	247.0721473	No
3199	HMDB0028829; CHEBI:73510	Glutamylthreonine	C9H16N2O6	247.0957061	No
3199	HMDB0029159; CHEBI:133747	gamma-Glutamylthreonine	C9H16N2O6	247.0957061	No
3199	HMDB0029575	(+)-cis-5,6-Dihydro-5-hydroxy-4-methoxy-6-(2-phenyle	C14H16O4	247.0957061	No
3199	HMDB0029774	7-Hydroxy-6-(methoxyacetyl)-2,2-dimethyl-2H-1-benzo	C14H16O4	247.0957061	No
3199	HMDB0033515	Citreovirenone	C14H16O4	247.0957061	No
3199	HMDB0041140; C10487; CHEBI:8393	Prenyl cis-caffeate	C14H16O4	247.0957061	No
3199	HMDB0128964	6-(3-hydroxybutyl)-7-methoxy-2H-chromen-2-one	C14H16O4	247.0957061	No
3199	HMDB0130532	7-hydroxy-6-(3-hydroxy-3-methylbutyl)-2H-chromen-2	C14H16O4	247.0957061	No
3199	HMDB0135890	3-[4-hydroxy-3-(4-hydroxy-3-methylbut-2-en-1-yl)pher	C14H16O4	247.0957061	No
3199	HMDB0135891	3-{4-hydroxy-3-[(2E)-4-hydroxy-3-methylbut-2-en-1-yl]	C14H16O4	247.0957061	No
3199	HMDB0135894	3-[3,4-dihydroxy-5-(3-methylbut-2-en-1-yl)phenyl]prop	C14H16O4	247.0957061	No
3199	HMDB0135895	3-[2,4-dihydroxy-3-(3-methylbut-2-en-1-yl)phenyl]prop	C14H16O4	247.0957061	No
3199	HMDB0135896	3-[2,4-dihydroxy-5-(3-methylbut-2-en-1-yl)phenyl]prop	C14H16O4	247.0957061	No
3200	HMDB0004081; C05643; CHEBI:308079	6-Hydroxymelatonin	C13H16N2O3	247.1082689	Yes
3200	HMDB0060069	cyclic 3-Hydroxymelatonin	C13H16N2O3	247.1082689	Yes
3200	HMDB0060721	2-Oxomelatonin	C13H16N2O3	247.1082689	Yes
3201	HMDB0031876; C09808	Prenyl glucoside	C11H20O6	247.1187917	Yes
3201	HMDB0033064	(E)-2-Methyl-2-buten-1-ol O-beta-D-Glucopyranoside	C11H20O6	247.1187917	Yes
3202	HMDB0001931	Gamma-CEHC	C15H20O3	247.1334827	No
3202	HMDB0030757	(S)-Pterosin A	C15H20O3	247.1334827	No
3202	HMDB0030888	Illudin C2	C15H20O3	247.1334827	No
3202	HMDB0030910	2alpha-Hydroxyalantolactone	C15H20O3	247.1334827	No
3202	HMDB0031378	4-Epiisoinuviscolide	C15H20O3	247.1334827	No
3202	HMDB0031386	(1(10)E,4beta,5alpha,8beta)-4,5-Epoxy-1(10),11(13)-ge	C15H20O3	247.1334827	No
3202	HMDB0031387	(1beta,4E,8beta,10alpha)-1,10-Epoxy-4,11(13)-germac	C15H20O3	247.1334827	No
3202	HMDB0031970	1beta-Hydroxyalantolactone	C15H20O3	247.1334827	No
3202	HMDB0032649	4,5-Epoxy-11(13)-guaien-12,8-olide	C15H20O3	247.1334827	No
3202	HMDB0033707	Heliannuol E	C15H20O3	247.1334827	No
3202	HMDB0034123	Eugenyl isovalerate	C15H20O3	247.1334827	No
3202	HMDB0035131	Heliannuol C	C15H20O3	247.1334827	No
3202	HMDB0035132	Heliannuol B	C15H20O3	247.1334827	No
3202	HMDB0035223	(S)-Pterosin D	C15H20O3	247.1334827	No
3202	HMDB0035503	Pseudoisoeugenol 2-methylbutanoate	C15H20O3	247.1334827	No
3202	HMDB0035708	8-Epiisoivangustin	C15H20O3	247.1334827	No
3202	HMDB0035903	Enokipodin A	C15H20O3	247.1334827	No
3202	HMDB0036005	Istanbulin B	C15H20O3	247.1334827	No
3202	HMDB0036040	Marasmen-3-one	C15H20O3	247.1334827	No
3202	HMDB0036641; C09301	Artabsin	C15H20O3	247.1334827	No
3202	HMDB0036770	Desacetyllaurenobiolide	C15H20O3	247.1334827	No
3202	HMDB0036772	Glechomafuran	C15H20O3	247.1334827	No
3202	HMDB0037022	Cichoralexin	C15H20O3	247.1334827	No
3202	HMDB0037557	1alpha-1-Hydroxy-2,4(18),11(13)-eudesmatrien-12-oic	C15H20O3	247.1334827	No
3202	HMDB0037800	Annuolide B	C15H20O3	247.1334827	No

3202	HMDB0038326	Annuolide E	C15H20O3	247.1334827	No
3202	HMDB0038794	2,12-Epoxy-7(14)-illudadiene-3,8-diol	C15H20O3	247.1334827	No
3202	HMDB0039859	Glandulone C	C15H20O3	247.1334827	No
3202	HMDB0134144	(2Z)-3-hydroxy-2-(phenylmethylidene)octanoic acid	C15H20O3	247.1334827	No
3202	HMDB0134145	(2Z)-2-[(3-hydroxyphenyl)methylidene]octanoic acid	C15H20O3	247.1334827	No
3202	HMDB0134146	(2Z)-2-[(4-hydroxyphenyl)methylidene]octanoic acid	C15H20O3	247.1334827	No
3202	HMDB0134147	(2Z)-7-hydroxy-2-(phenylmethylidene)octanoic acid	C15H20O3	247.1334827	No
3202	HMDB0134148	2-hexyl-3-phenyloxirane-2-carboxylic acid	C15H20O3	247.1334827	No
3202	HMDB0134149	(2Z)-8-hydroxy-2-(phenylmethylidene)octanoic acid	C15H20O3	247.1334827	No
3202	HMDB0134150	(2Z)-2-[(2-hydroxyphenyl)methylidene]octanoic acid	C15H20O3	247.1334827	No
3203	HMDB0015095; C07445; CHEBI:8214	Pindolol	C14H20N2O2	247.1453638	No
3221	HMDB0001915; C07752; CHEBI:2567	Alendronic acid	C4H13NO7P2	248.0088891	Yes
3222	HMDB0002062	Norepinephrine sulfate	C8H11NO6S	248.023921	Yes
3223	HMDB0028774	Cysteinyl-Glutamate	C8H13N2O5S	248.0462393	Yes
3224	HMDB0015028; CHEBI:132842	Sulfapyridine	C11H11N3O2S	248.0501993	No
3230	HMDB0060070	Melatonin radical	C13H17N2O3	248.1147645	No
3231	HMDB0094646	2-Pentanamido-3-phenylpropanoic acid	C14H19NO3	248.1288462	No
3232	HMDB0060531	Desmethylnortriptyline	C18H19N	248.1445132	No
3234	HMDB0015004; CHEBI:51211	Alprenolol	C15H23NO2	248.1655266	No
3234	HMDB0060852; CHEBI:83529	N,O-Didesmethylvenlafaxine	C15H23NO2	248.1655266	No
3234	HMDB0060997	O-Desmethyltramadol	C15H23NO2	248.1655266	No
3234	HMDB0061007	N-Desmethyltramadol	C15H23NO2	248.1655266	No
3248	HMDB0126639	3,5-dihydroxy-4-(sulfooxy)benzoic acid	C7H6O8S	248.9714595	Yes
3248	HMDB0130471	2,4-dihydroxy-3-(sulfooxy)benzoic acid	C7H6O8S	248.9714595	Yes
3253	HMDB0039464	(E)-2-Propenyl [3-(2-propenylthio)-2-propenyl] sulfate	C9H14O4S2	249.0265325	No
3257	HMDB0032353	Isopropyl citrate	C10H18O7	249.0982053	Yes
3260	HMDB0015443; C17725; CHEBI:588074	Heptabarbital	C13H18N2O3	249.1240203	Yes
3260	HMDB0029876; C03002	Paucine	C13H18N2O3	249.1240203	Yes
3260	HMDB0033462	4-Coumaroyl-2-hydroxyputrescine	C13H18N2O3	249.1240203	Yes
3260	HMDB0060742	3-Hydroxymelatonin	C13H18N2O3	249.1240203	Yes
3260	HMDB0139529	(2E)-N-(4-aminobutyl)-3-(3,4-dihydroxyphenyl)prop-2-	C13H18N2O3	249.1240203	Yes
3260	HMDB0139530	N-(4-aminobutyl)-3-(4-hydroxyphenyl)oxirane-2-carbo	C13H18N2O3	249.1240203	Yes
3260	HMDB0139613	N-(4-aminobutyl)-3-(3,4-dihydroxyphenyl)prop-2-enim	C13H18N2O3	249.1240203	Yes
3260	HMDB0139973	(2E)-N-(3-aminopropyl)-3-(4-hydroxy-3-methoxypheny	C13H18N2O3	249.1240203	Yes
3261	HMDB0015371; C07020; CHEBI:5296	Gemfibrozil	C15H22O3	249.1498519	No
3261	HMDB0030917	1-Hydroxyacorenone	C15H22O3	249.1498519	No
3261	HMDB0031901	Blennin A	C15H22O3	249.1498519	No
3261	HMDB0034721	Procurcumadiol	C15H22O3	249.1498519	No
3261	HMDB0035117	(3beta,6beta)-Furanoeremophilane-3,6-diol	C15H22O3	249.1498519	No
3261	HMDB0035137	Heliannuol D	C15H22O3	249.1498519	No
3261	HMDB0035148	(6beta,8alpha)-6-Hydroxy-7(11)-eremophilen-12,8-olid	C15H22O3	249.1498519	No
3261	HMDB0035358	Ketosantalic acid	C15H22O3	249.1498519	No
3261	HMDB0035760	3-Hydroxytrichothecene	C15H22O3	249.1498519	No
3261	HMDB0035798; C09711	Piperdial	C15H22O3	249.1498519	No
3261	HMDB0036036	3beta-Dihydroxymarasmene	C15H22O3	249.1498519	No
3261	HMDB0036037	13-Hydroxymarasmene	C15H22O3	249.1498519	No
3261	HMDB0036550	3-Ketoapotrichothecene	C15H22O3	249.1498519	No
3261	HMDB0036563	Valerenolic acid	C15H22O3	249.1498519	No
3261	HMDB0036664	Ketopelenolide a	C15H22O3	249.1498519	No
3261	HMDB0037064	FS4 toxin	C15H22O3	249.1498519	No
3261	HMDB0037529	Lactaronecatorin A	C15H22O3	249.1498519	No
3261	HMDB0037559	Cadabicilone	C15H22O3	249.1498519	No
3261	HMDB0039156	3beta-Hydroxycinnamolide	C15H22O3	249.1498519	No
3261	HMDB0039635	Abscisic alcohol	C15H22O3	249.1498519	No
3261	HMDB0039644	Heliannuol A	C15H22O3	249.1498519	No
3261	HMDB0040754	(3beta,8beta)-3-Hydroxy-7(11)-eremophilen-12,8-olide	C15H22O3	249.1498519	No
3261	HMDB0061839	Ethylhexyl salicylate	C15H22O3	249.1498519	No
3279	HMDB0029355	N-Phenylacetylaspartic acid	C12H13NO5	250.0723271	No
3279	HMDB0039102	N-Carboxyacetyl-D-phenylalanine	C12H13NO5	250.0723271	No
3279	HMDB0059747; C02564; CHEBI:17691	4-Hydroxy-3-methoxy-cinnamoylglycine	C12H13NO5	250.0723271	No
3309	HMDB0028781	Cysteinyl-Methionine	C8H16N2O3S2	251.0526647	Yes

3309	HMDB0028970	Methionyl-Cysteine	C8H16N2O3S2	251.0526647	Yes
3310	HMDB0004086; C05648; CHEBI:2065	5-Hydroxy-N-formylkynurenine	C11H12N2O5	251.0679293	Yes
3310	HMDB0035993	N5-(3,4-Dioxo-1,5-cyclohexadien-1-yl)-L-glutamine	C11H12N2O5	251.0679293	Yes
3311	HMDB0000071; C05512; CHEBI:28997	Deoxyinosine	C10H12N4O4	251.0788703	Yes
3311	HMDB0029956; C01736; CHEBI:139743	Nebularine	C10H12N4O4	251.0788703	Yes
3312	HMDB0030668	1-(2,4,6-Trimethoxyphenyl)-1,3-butanedione	C13H16O5	251.0927794	Yes
3312	HMDB0038551; CHEBI:86898	Methyl 3,4,5-trimethoxycinnamate	C13H16O5	251.0927794	Yes
3312	HMDB0125460	2-[2,4,6-trihydroxy-3-(3-methylbut-2-en-1-yl)phenyl]ac	C13H16O5	251.0927794	Yes
3312	HMDB0137142	2-[2,4-dihydroxy-3-(3-methylbut-2-en-1-yl)phenyl]-2-h	C13H16O5	251.0927794	Yes
3314	HMDB0014644; C06952; CHEBI:3699	Cimetidine	C10H16N6S	251.1084794	No
3314	HMDB0033317	(1Z,4Z)-1,5-bis(4-hydroxyphenyl)-1,4-pentadiene	C17H16O2	251.1084794	No
3314	HMDB0035018	2-Phenylethyl 3-phenyl-2-propenoate	C17H16O2	251.1084794	No
3314	HMDB0037707	Cinnamyl phenylacetate	C17H16O2	251.1084794	No
3315	HMDB0040822	2,3-Butanediol glucoside	C10H20O7	251.1137965	Yes
3316	HMDB0033197	2,2,4,4-Tetramethyl-6-(1-oxobutyl)-1,3,5-cyclohexanet	C14H20O4	251.1264481	Yes
3316	HMDB0036047	3alpha-Hydroxyoreadone	C14H20O4	251.1264481	Yes
3316	HMDB0059661; CHEBI:17976	QH(2)	C14H20O4	251.1264481	Yes
3317	HMDB0014451	Talbutal	C13H20N2O3	251.139883	Yes
3317	HMDB0139534	N-(4-aminobutyl)-3-(3,4-dihydroxyphenyl)propanimidi	C13H20N2O3	251.139883	Yes
3321	HMDB0030918	1-Hydroxyepiacorone	C15H24O3	251.1653901	No
3321	HMDB0030919	Acorusdiol	C15H24O3	251.1653901	No
3321	HMDB0032122	3(4->5)-Abeo-4,11:4,12-diepoxy-3-eudesmanol	C15H24O3	251.1653901	No
3321	HMDB0033149	(4R,5S,7R,11R)-11,12-Dihydroxy-1(10)-spirovetiven-2-c	C15H24O3	251.1653901	No
3321	HMDB0034574	Apotrichodiol	C15H24O3	251.1653901	No
3321	HMDB0035309	6alpha-Carissanol	C15H24O3	251.1653901	No
3321	HMDB0035597	alpha-Carissanol	C15H24O3	251.1653901	No
3321	HMDB0035613	Epioxylubimin	C15H24O3	251.1653901	No
3321	HMDB0035729	Dihydromyoporone	C15H24O3	251.1653901	No
3321	HMDB0035767	Piperalol	C15H24O3	251.1653901	No
3321	HMDB0036448	Zedoarondiol	C15H24O3	251.1653901	No
3321	HMDB0036663	Hydroxypelenolide	C15H24O3	251.1653901	No
3321	HMDB0037063	Toxin FS2	C15H24O3	251.1653901	No
3321	HMDB0037969	Urodiolenone	C15H24O3	251.1653901	No
3321	HMDB0038502	Bisacurone B	C15H24O3	251.1653901	No
3321	HMDB0040685	2,3-Dihydroabscisic alcohol	C15H24O3	251.1653901	No
3321	HMDB0112086	3-Methyl-5-pentyl-2-furanpentanoic acid	C15H24O3	251.1653901	No
3321	HMDB0112094	3-Methyl-5-propyl-2-furanheptanoic acid	C15H24O3	251.1653901	No
3323	HMDB0000477; CHEBI:86147	7Z,10Z-Hexadecadienoic acid	C16H28O2	251.2017014	No
3323	HMDB0029351	Geranyl hexanoate	C16H28O2	251.2017014	No
3323	HMDB0030429	Linalyl hexanoate	C16H28O2	251.2017014	No
3323	HMDB0031086	(Z)-7-Hexadecen-1,16-olide	C16H28O2	251.2017014	No
3323	HMDB0032340	Isoambrettolide	C16H28O2	251.2017014	No
3323	HMDB0037805	D6-Ambrettolide	C16H28O2	251.2017014	No
3323	HMDB0038255	Geranyl 2-ethylbutyrate	C16H28O2	251.2017014	No
3323	HMDB0039131	Ethyl (Z,Z)-5,8-tetradecadienoate	C16H28O2	251.2017014	No
3339	HMDB0015150; C07315; CHEBI:9332	Sulfamethoxazole	C10H11N3O3S	252.0452048	No
3341	HMDB0000727; CHEBI:49751	L-Threoneopterin	C9H11N5O4	252.0730104	No
3341	HMDB0000845; C05926	Neopterin	C9H11N5O4	252.0730104	No
3341	HMDB0000877	Umanopterin	C9H11N5O4	252.0730104	No
3341	HMDB0002109	Hydroxysepiapterin	C9H11N5O4	252.0730104	No
3341	HMDB0029895	D-erythro-Eritadenine	C9H11N5O4	252.0730104	No
3345	HMDB0001940; D00386; CHEBI:117411	Triamterene	C12H11N7	252.1003827	Yes
3345	HMDB0032758	Ethyl 3-methyl-9H-carbazole-9-carboxylate	C16H15NO2	252.1003827	Yes
3345	HMDB0041319	Cinnamyl anthranilate	C16H15NO2	252.1003827	Yes
3346	HMDB0014444; C07417; CHEBI:7956	Penciclovir	C10H15N5O3	252.1120827	No
3376	HMDB0060603	N-acetyl zonisamide	C10H10N2O4S	253.0291961	No
3377	HMDB0003312; C10208; CHEBI:28197	Daidzein	C15H10O4	253.0507345	No
3377	HMDB0004195; C05844	5-L-Glutamyl-taurine	C7H14N2O6S	253.0507345	No
3377	HMDB0030670; C10315; CHEBI:3687	Chrysophanol	C15H10O4	253.0507345	No
3377	HMDB0030699; C02168; CHEBI:1012277	5,7-Dihydroxyisoflavone	C15H10O4	253.0507345	No
3377	HMDB0030874	Phomarin	C15H10O4	253.0507345	No

3377	HMDB0033153; C08644; CHEBI:5731	(Z)-4'',6-Dihydroxyaurone	C15H10O4	253.0507345	No
3377	HMDB0036619; C10028	5,7-Dihydroxyflavone	C15H10O4	253.0507345	No
3377	HMDB0130498	6,7-dihydroxy-3-phenyl-4H-chromen-4-one	C15H10O4	253.0507345	No
3377	HMDB0130504	7-hydroxy-3-(3-hydroxyphenyl)-4H-chromen-4-one	C15H10O4	253.0507345	No
3377	HMDB0130510	7,8-dihydroxy-3-phenyl-4H-chromen-4-one	C15H10O4	253.0507345	No
3377	HMDB0130907	5,6-dihydroxy-2-phenyl-4H-chromen-4-one	C15H10O4	253.0507345	No
3377	HMDB0130927	6,7-dihydroxy-2-phenyl-4H-chromen-4-one	C15H10O4	253.0507345	No
3377	HMDB0133314	7-hydroxy-2-(3-hydroxyphenyl)-4H-chromen-4-one	C15H10O4	253.0507345	No
3377	HMDB0134545	3-hydroxy-2-(2-hydroxyphenyl)-4H-chromen-4-one	C15H10O4	253.0507345	No
3377	HMDB0134546	3,6-dihydroxy-2-phenyl-4H-chromen-4-one	C15H10O4	253.0507345	No
3377	HMDB0134547	3,7-dihydroxy-2-phenyl-4H-chromen-4-one	C15H10O4	253.0507345	No
3377	HMDB0134548	3-hydroxy-2-(3-hydroxyphenyl)-4H-chromen-4-one	C15H10O4	253.0507345	No
3377	HMDB0134549	3-hydroxy-2-(4-hydroxyphenyl)-4H-chromen-4-one	C15H10O4	253.0507345	No
3377	HMDB0134550	3,5-dihydroxy-2-phenyl-4H-chromen-4-one	C15H10O4	253.0507345	No
3377	HMDB0137521	5,6-dihydroxy-2-(phenylmethylidene)-2,3-dihydro-1-be	C15H10O4	253.0507345	No
3377	HMDB0137524	6,7-dihydroxy-2-(phenylmethylidene)-2,3-dihydro-1-be	C15H10O4	253.0507345	No
3377	HMDB0137527	4,6-dihydroxy-2-(phenylmethylidene)-2,3-dihydro-1-be	C15H10O4	253.0507345	No
3377	HMDB0137530	6-hydroxy-2-[(3-hydroxyphenyl)methylidene]-2,3-dihy	C15H10O4	253.0507345	No
3377	HMDB0137607	6-hydroxy-2-[(4-hydroxyphenyl)methylidene]-2,3-dihy	C15H10O4	253.0507345	No
3377	HMDB0140470	2-(3,5-dihydroxyphenyl)-4H-chromen-4-one	C15H10O4	253.0507345	No
3378	HMDB0029445	L-Agaridoxin	C11H14N2O5	253.0834559	Yes
3378	HMDB0060669; C16584; CHEBI:80584	p-Hydroxyfelbamate	C11H14N2O5	253.0834559	Yes
3378	HMDB0060670; C16582	2-Hydroxyfelbamate	C11H14N2O5	253.0834559	Yes
3379	HMDB0006790; C05401; CHEBI:15754	Galactosylglycerol	C9H18O8	253.0929559	Yes
3379	HMDB0014789; C07819; CHEBI:4728	Dyphylline	C10H14N4O4	253.0929559	Yes
3379	HMDB0038664	(2R)-1-O-beta-D-Galactopyranosylglycerol	C9H18O8	253.0929559	Yes
3380	HMDB0015285; C07846; CHEBI:9536	Thiamylal	C12H18N2O2S	253.1008275	Yes
3382	HMDB0014356; C07890; CHEBI:6933	Midodrine	C12H18N2O4	253.119784	Yes
3382	HMDB0029390	L-Furosine	C12H18N2O4	253.119784	Yes
3382	HMDB0029443	L-Pyridosine	C12H18N2O4	253.119784	Yes
3382	HMDB0033143	Pyrraline	C12H18N2O4	253.119784	Yes
3382	HMDB0037817	Aldosine	C12H18N2O4	253.119784	Yes
3383	HMDB0005767	Homoanserine	C11H18N4O3	253.1306602	Yes
3383	HMDB0028898	Histidinyl-Valine	C11H18N4O3	253.1306602	Yes
3383	HMDB0029129	Valyl-Histidine	C11H18N4O3	253.1306602	Yes
3383	HMDB0037180	(S)-Argpyrimidine	C11H18N4O3	253.1306602	Yes
3385	HMDB0029604	Lubiminol	C15H26O3	253.1811511	No
3385	HMDB0035780	8-Hydroxy-4(6)-lactarene-5,14-diol	C15H26O3	253.1811511	No
3385	HMDB0036053	(3beta,9beta)-7-Drimene-3,11,12-triol	C15H26O3	253.1811511	No
3385	HMDB0036866	7-Drimene-11,12,14-triol	C15H26O3	253.1811511	No
3385	HMDB0037197	Kessyl glycol	C15H26O3	253.1811511	No
3385	HMDB0040276	5-Acetoxydihydrotheaespirane	C15H26O3	253.1811511	No
3387	HMDB0002186; CHEBI:35465	Hypogeic acid	C16H30O2	253.2171588	No
3387	HMDB0003229; C08362; CHEBI:28716	Palmitoleic acid	C16H30O2	253.2171588	No
3387	HMDB0010735; CHEBI:37252	Trans-Hexa-dec-2-enoic acid	C16H30O2	253.2171588	No
3387	HMDB0012328; CHEBI:59265	Palmitelaidic acid	C16H30O2	253.2171588	No
3387	HMDB0031053	(E)-6-Hexadecenoic acid	C16H30O2	253.2171588	No
3387	HMDB0031145	5-Dodecyldihydro-2(3H)-furanone	C16H30O2	253.2171588	No
3387	HMDB0031711	15-Hexadecanolide	C16H30O2	253.2171588	No
3387	HMDB0032638	(Z)-5-Hexadecenoic acid	C16H30O2	253.2171588	No
3387	HMDB0033791	(E)-3-Hexadecenoic acid	C16H30O2	253.2171588	No
3387	HMDB0035877	(Z)-13-Hexadecenoic acid	C16H30O2	253.2171588	No
3387	HMDB0037647	(E)-11-Hexadecenoic acid	C16H30O2	253.2171588	No
3387	HMDB0038958	Citronellyl hexanoate	C16H30O2	253.2171588	No
3387	HMDB0041422	(Z)-14-Methyl-6-pentadecenoic acid	C16H30O2	253.2171588	No
3403	HMDB0125091	3,5,7-trihydroxy-2-phenyl-1??-chromen-1-ylium	C15H11O4	254.0612391	No
3404	HMDB0002275; C04874; CHEBI:17001	7,8-Dihydroneopterin	C9H13N5O4	254.0897992	Yes
3404	HMDB0015139; CHEBI:465284	Ganciclovir	C9H13N5O4	254.0897992	Yes
3421	HMDB0060649	Ascorbic acid-2-sulfate	C6H8O9S	254.9811836	Yes
3422	HMDB0030809; CHEBI:997963	(2R,3S)-Piscidic acid	C11H12O7	255.0511849	Yes
3423	HMDB0005760; CHEBI:75842	Dihydrodaidzein	C15H12O4	255.0665024	Yes

3423	HMDB0029462	(E)-2,4,4''-Trihydroxychalcone	C15H12O4	255.0665024	Yes
3423	HMDB0029519; C09762; CHEBI:536636	(2S)-Liquiritigenin	C15H12O4	255.0665024	Yes
3423	HMDB0030808; C09827; CHEBI:215489	(S)-Pinocembrin	C15H12O4	255.0665024	Yes
3423	HMDB0032577	4''-Methoxybenzophenone-2-carboxylic acid	C15H12O4	255.0665024	Yes
3423	HMDB0033904	7E-Mycosinyl acetate	C15H12O4	255.0665024	Yes
3423	HMDB0036457	Emodinanthranol	C15H12O4	255.0665024	Yes
3423	HMDB0037316; C08650	Isoliquiritigenin	C15H12O4	255.0665024	Yes
3423	HMDB0041647	2-Dehydro-O-desmethylangolensin	C15H12O4	255.0665024	Yes
3423	HMDB0061744; CHEBI:132612	Mono-benzyl phthalate	C15H12O4	255.0665024	Yes
3423	HMDB0128077	3-phenyl-1-(2,4,6-trihydroxyphenyl)prop-2-en-1-one	C15H12O4	255.0665024	Yes
3423	HMDB0129959	5,7-dihydroxy-3-phenyl-3,4-dihydro-2H-1-benzopyran-	C15H12O4	255.0665024	Yes
3423	HMDB0134575	5,8-dihydroxy-2-phenyl-3,4-dihydro-2H-1-benzopyran-	C15H12O4	255.0665024	Yes
3423	HMDB0137512	6-hydroxy-2-[(4-hydroxyphenyl)methyl]-2,3-dihydro-1-	C15H12O4	255.0665024	Yes
3423	HMDB0137518	2-[(4-hydroxyphenyl)methylidene]-2,3-dihydro-1-benz	C15H12O4	255.0665024	Yes
3423	HMDB0137526	2-benzyl-6,7-dihydroxy-2,3-dihydro-1-benzofuran-3-or	C15H12O4	255.0665024	Yes
3423	HMDB0138504	8,17-dioxatetracyclo[8.7.0.0²,?.0¹¹,¹?]heptadeca-2(7),3,	C15H12O4	255.0665024	Yes
3423	HMDB0141337	(2S)-5,7-dihydroxy-2-phenyl-3,4-dihydro-2H-1-benzopy	C15H12O4	255.0665024	Yes
3425	HMDB0141328	3-hydroxy-3-(3,4,5-trimethoxyphenyl)propanoic acid	C12H16O6	255.0880024	Yes
3425	HMDB0141330	2-hydroxy-3-(3,4,5-trimethoxyphenyl)propanoic acid	C12H16O6	255.0880024	Yes
3425	HMDB0142079	3-hydroxy-3-(2,3,4-trimethoxyphenyl)propanoic acid	C12H16O6	255.0880024	Yes
3425	HMDB0142082	2-hydroxy-3-(2,3,4-trimethoxyphenyl)propanoic acid	C12H16O6	255.0880024	Yes
3427	HMDB0011648; C15497; CHEBI:55458	1-(beta-D-Ribofuranosyl)-1,4-dihydronicotinamide	C11H16N2O5	255.0995025	No
3428	HMDB0015190; C06975; CHEBI:4753	Echothiophate	C9H23NO3PS	255.1066433	No
3429	HMDB0031772	1-Methoxy-1-(2,4,5-trimethoxyphenyl)-2-propanol	C13H20O5	255.1238258	Yes
3429	HMDB0040352	2-[4-(3-Hydroxypropyl)-2-methoxyphenoxy]-1,3-propa	C13H20O5	255.1238258	Yes
3434	HMDB0040904	Ipomeatetrahydrofuran	C15H28O3	255.1977407	No
3435	HMDB0000220; C00249; CHEBI:15756	Palmitic acid	C16H32O2	255.2330955	Yes
3435	HMDB0002396; CHEBI:58966	Trimethyltridecanoic acid	C16H32O2	255.2330955	Yes
3435	HMDB0031068; CHEBI:84890	Isopalmitic acid	C16H32O2	255.2330955	Yes
3435	HMDB0032065	Butyl dodecanoate	C16H32O2	255.2330955	Yes
3435	HMDB0032249	Dodecyl butyrate	C16H32O2	255.2330955	Yes
3435	HMDB0032324	Hexyl decanoate	C16H32O2	255.2330955	Yes
3435	HMDB0033166	Octyl octanoate	C16H32O2	255.2330955	Yes
3435	HMDB0034153; CHEBI:84849	Ethyl tetradecanoate	C16H32O2	255.2330955	Yes
3435	HMDB0036210	Dodecyl 2-methylpropanoate	C16H32O2	255.2330955	Yes
3459	HMDB0000982; CHEBI:20607	5-Methylcytidine	C10H15N3O5	256.093248	Yes
3459	HMDB0039229	Pyro-L-glutaminyl-L-glutamine	C10H15N3O5	256.093248	Yes
3464	HMDB0013161	2-Hexenoylcarnitine	C13H23NO4	256.1559059	No
3466	HMDB0014045; CHEBI:580449	3-Methoxymorphinan	C17H23NO	256.1707393	No
3466	HMDB0014992; C08014; CHEBI:119572	Levorphanol	C17H23NO	256.1707393	No
3466	HMDB0060552; CHEBI:29133	Dextrorphan	C17H23NO	256.1707393	No
3467	HMDB0013272; CHEBI:74441	N-Lauroylglycine	C14H27NO3	256.1921158	No
3484	HMDB0012282; C00404; CHEBI:39949	PPPi	H5O10P3	256.902591	Yes
3493	HMDB0131180	{4-[(1E)-3-methoxy-3-oxoprop-1-en-1-yl]phenyl}oxidar	C10H10O6S	257.0130187	Yes
3493	HMDB0133661	(2Z)-3-phenyl-2-[(sulfooxy)methyl]prop-2-enoic acid	C10H10O6S	257.0130187	Yes
3493	HMDB0133664	(2E)-2-methyl-3-[3-(sulfooxy)phenyl]prop-2-enoic acid	C10H10O6S	257.0130187	Yes
3493	HMDB0133667	(2E)-2-methyl-3-[4-(sulfooxy)phenyl]prop-2-enoic acid	C10H10O6S	257.0130187	Yes
3493	HMDB0135681	[2-hydroxy-5-(3-oxobut-1-en-1-yl)phenyl]oxidanesulfo	C10H10O6S	257.0130187	Yes
3501	HMDB0032527	1,1''-(Tetrahydro-6a-hydroxy-2,3a,5-trimethylfuro[2,3-	C12H18O6	257.1033649	Yes
3502	HMDB0030420	Strobilurin A	C16H18O3	257.116119	Yes
3502	HMDB0038614	gamma-L-Glutamyl-L-pipecolic acid	C11H18N2O5	257.116119	Yes
3502	HMDB0038676	(2S,2''S)-Pyrosaccharopine	C11H18N2O5	257.116119	Yes
3507	HMDB0000872; C11002; CHEBI:76308	Tetradecanedioic acid	C14H26O4	257.1758192	Yes
3507	HMDB0040429	Diethyl decanedioate	C14H26O4	257.1758192	Yes
3507	HMDB0041618; C14260	Diisobutyl adipate	C14H26O4	257.1758192	Yes
3507	HMDB0059946	2-Hydroxypropyl 2-isopropyl-5-methylcyclohexyl carbo	C14H26O4	257.1758192	Yes
3507	HMDB0059947	1-Hydroxypropan-2-yl 2-isopropyl-5-methylcyclohexyl	C14H26O4	257.1758192	Yes
3508	HMDB0061860	2-Acetyl-3,5,5,6,8,8-hexamethyl-5,6,7,8- tetrahydrona	C18H26O	257.1899593	No
3510	HMDB0031061	10-Hydroxymyristic acid methyl ester	C15H30O3	257.2128361	No
3510	HMDB0059866; CHEBI:87428	Ethyl 3-hydroxytridecanoate	C15H30O3	257.2128361	No
3510	HMDB0061657	3-hydroxypentadecanoic acid	C15H30O3	257.2128361	No

3530	HMDB0000592; C04132; CHEBI:743540	Glucosamine 6-sulfate	C6H13NO8S	258.0286088	Yes
3530	HMDB0001481; C01075; CHEBI:16702	N-Sulfo-D-glucosamine	C6H13NO8S	258.0286088	Yes
3534	HMDB0041934	Mizoribine	C9H13N3O6	258.0755421	Yes
3536	HMDB0028798	Glutaminylhydroxyproline	C10H17N3O5	258.1084222	No
3536	HMDB0028861	Hydroxyprolyl-Glutamine	C10H17N3O5	258.1084222	No
3536	HMDB0028877	Hydroxyprolyl-Gamma-glutamate	C10H17N3O5	258.1084222	No
3536	HMDB0029150	Gamma-glutamyl-Hydroxyproline	C10H17N3O5	258.1084222	No
3536	HMDB0038516; C05939; CHEBI:28004	Linatine	C10H17N3O5	258.1084222	No
3537	HMDB0001849; C07407; CHEBI:8499	Propranolol	C16H21NO2	258.1501589	No
3537	HMDB0015115; CHEBI:321962	Ramelteon	C16H21NO2	258.1501589	No
3537	HMDB0029829	Norchalciporyl propionate	C16H21NO2	258.1501589	No
3537	HMDB0041954	Norbutorphanol	C16H21NO2	258.1501589	No
3552	HMDB0041706; CHEBI:90242	Caffeic acid 3-sulfate	C9H8O7S	258.9919125	Yes
3552	HMDB0041708	Caffeic acid 4-sulfate	C9H8O7S	258.9919125	Yes
3552	HMDB0125409	2-oxo-3-[4-(sulfooxy)phenyl]propanoic acid	C9H8O7S	258.9919125	Yes
3552	HMDB0125831	3-[4-(sulfooxy)phenyl]oxirane-2-carboxylic acid	C9H8O7S	258.9919125	Yes
3552	HMDB0127962	3-[4-hydroxy-3-(sulfooxy)phenyl]prop-2-enoic acid	C9H8O7S	258.9919125	Yes
3552	HMDB0134087	3-[2-hydroxy-3-(sulfooxy)phenyl]prop-2-enoic acid	C9H8O7S	258.9919125	Yes
3552	HMDB0135272	(2E)-3-[2-hydroxy-3-(sulfooxy)phenyl]prop-2-enoic acid	C9H8O7S	258.9919125	Yes
3554	HMDB0135261	[2-hydroxy-6-methoxy-4-(prop-2-en-1-yl)phenyl]oxidar	C10H12O6S	259.0274247	No
3554	HMDB0135264	{2-methoxy-4-[(oxiran-2-yl)methyl]phenyl}oxidanesulf	C10H12O6S	259.0274247	No
3554	HMDB0135659	{[3-(2-hydroxy-4-methoxyphenyl)prop-2-en-1-yl]oxy}su	C10H12O6S	259.0274247	No
3554	HMDB0135663	{[3-(4-methoxyphenyl)oxiran-2-yl]methoxy}sulfonic aci	C10H12O6S	259.0274247	No
3554	HMDB0135705	[2-hydroxy-5-(3-oxobutyl)phenyl]oxidanesulfonic acid	C10H12O6S	259.0274247	No
3554	HMDB0135744	{[1-(4-methoxyphenyl)-3-oxopropan-2-yl]oxy}sulfonic a	C10H12O6S	259.0274247	No
3554	HMDB0135746	[1-(4-methoxyphenyl)-3-oxopropoxy]sulfonic acid	C10H12O6S	259.0274247	No
3557	HMDB0028730	Asparaginyl-Glutamate	C9H14N3O6	259.0788511	Yes
3559	HMDB0032857; CHEBI:88153	Glycerol tripropanoate	C12H20O6	259.119306	Yes
3560	HMDB0011170; HMDB0029152; CHEBI:6843	gamma-Glutamylisoleucine	C11H20N2O5	259.1302754	No
3560	HMDB0011171; HMDB0029153; CHEBI:6843	gamma-Glutamylleucine	C11H20N2O5	259.1302754	No
3560	HMDB0028822	Glutamylisoleucine	C11H20N2O5	259.1302754	No
3560	HMDB0028823; CHEBI:73506	Glutamylleucine	C11H20N2O5	259.1302754	No
3563	HMDB0014413; C07564; CHEBI:8888	Ropinirole	C16H24N2O	259.1819114	Yes
3563	HMDB0015070; C07363; CHEBI:142520	Oxymetazoline	C16H24N2O	259.1819114	Yes
3583	HMDB0062550; CHEBI:82944	2-Methoxyacetaminophen sulfate	C9H11NO6S	260.023806	Yes
3586	HMDB0013133	Methylmalonylcarnitine	C11H19NO6	260.114731	No
3586	HMDB0033865; C08334	Lotaustralin	C11H19NO6	260.114731	No
3586	HMDB0034777	Epidermin	C11H19NO6	260.114731	No
3586	HMDB0039082	Camelinin	C12H23NOS2	260.114731	No
3586	HMDB0061717	Succinylcarnitine	C11H19NO6	260.114731	No
3586	HMDB0062785; CHEBI:85473	O-methylmalonyl-L-carnitine	C11H19NO6	260.114731	No
3587	HMDB0002248	Gamma glutamyl ornithine	C10H19N3O5	260.1247387	Yes
3587	HMDB0004985	Aspartylysine	C10H19N3O5	260.1247387	Yes
3587	HMDB0004987; CHEBI:204036	Alpha-Aspartyl-lysine	C10H19N3O5	260.1247387	Yes
3587	HMDB0028758	Aspartyl-Lysine	C10H19N3O5	260.1247387	Yes
3587	HMDB0028947	Lysyl-Aspartate	C10H19N3O5	260.1247387	Yes
3587	HMDB0240241	Thenyldiamine	C14H19N3S	260.1247387	Yes
3590	HMDB0015556; C07790; CHEBI:130096	Phenindamine	C19H19N	260.1449388	No
3590	HMDB0060948; CHEBI:93101	desmethylcyclobenzaprine	C19H19N	260.1449388	No
3591	HMDB0062555	hydroxyisovaleroyl carnitine	C12H23NO5	260.1493388	No
3611	HMDB0011719; CHEBI:88405	Homovanillic acid sulfate	C9H10O7S	261.0079493	Yes
3611	HMDB0041648	2-Hydroxy-4-methoxyacetophenone 5-sulfate	C9H10O7S	261.0079493	Yes
3611	HMDB0041721	Dihydrocaffeic acid 3-sulfate	C9H10O7S	261.0079493	Yes
3611	HMDB0059967; CHEBI:88716	3-hydroxy-3-(3-hydroxyphenyl)propanoic acid-O-sulph	C9H10O7S	261.0079493	Yes
3611	HMDB0061117	3-(3,5-dihydroxyphenyl)-1-propanoic acid sulphate	C9H10O7S	261.0079493	Yes
3611	HMDB0132291	2-hydroxy-3-[4-(sulfooxy)phenyl]propanoic acid	C9H10O7S	261.0079493	Yes
3611	HMDB0132292	3-(4-hydroxyphenyl)-2-(sulfooxy)propanoic acid	C9H10O7S	261.0079493	Yes
3611	HMDB0134099	3-[2-hydroxy-3-(sulfooxy)phenyl]propanoic acid	C9H10O7S	261.0079493	Yes
3613	HMDB0032644; C09951	Maclurin	C13H10O6	261.0407137	No
3614	HMDB0031762	Carbadox	C11H10N4O4	261.0617517	No
3616	HMDB0030882	(S)-Rutaretin	C14H14O5	261.0757517	Yes

3616	HMDB0032838	Dorsteniol	C14H14O5	261.0757517	Yes
3616	HMDB0039027	Celereoin	C14H14O5	261.0757517	Yes
3616	HMDB0128944	9-hydroxy-8-(2-hydroxypropan-2-yl)-2H,8H,9H-furo[2,3	C14H14O5	261.0757517	Yes
3616	HMDB0128958	6-(1-hydroxy-3-oxobutyl)-7-methoxy-2H-chromen-2-or	C14H14O5	261.0757517	Yes
3616	HMDB0128960	8-hydroxy-7-methoxy-6-(3-oxobutyl)-2H-chromen-2-or	C14H14O5	261.0757517	Yes
3616	HMDB0128961	5-hydroxy-7-methoxy-6-(3-oxobutyl)-2H-chromen-2-or	C14H14O5	261.0757517	Yes
3616	HMDB0128962	6-(4-hydroxy-3-oxobutyl)-7-methoxy-2H-chromen-2-or	C14H14O5	261.0757517	Yes
3616	HMDB0128963	6-(2-hydroxy-3-oxobutyl)-7-methoxy-2H-chromen-2-or	C14H14O5	261.0757517	Yes
3616	HMDB0131630	3-hydroxy-2-(2-hydroxypropan-2-yl)-2H,3H,7H-furo[3,2	C14H14O5	261.0757517	Yes
3616	HMDB0131691	6-[2-(2H-1,3-benzodioxol-5-yl)ethyl]-4-hydroxy-5,6-dih	C14H14O5	261.0757517	Yes
3616	HMDB0138052	9,10-dihydroxy-8,8-dimethyl-2H,8H,9H,10H-pyrano[2,3	C14H14O5	261.0757517	Yes
3617	HMDB0031360	L-cis-Cyclo(aspartylphenylalanyl)	C13H14N2O4	261.088181	Yes
3619	HMDB0011177; CHEBI:356661	Phenylalanylproline	C14H18N2O3	261.1245019	Yes
3619	HMDB0011179; CHEBI:299684	L-prolyl-L-phenylalanine	C14H18N2O3	261.1245019	Yes
3619	HMDB0014617; C07844; CHEBI:102216	Methohexital	C14H18N2O3	261.1245019	Yes
3620	HMDB0031897	Phaseolic acid	C12H22O6	261.1347054	Yes
3622	HMDB0032297	Glyceryl 5-hydroxydecanoate	C13H26O5	261.1709355	No
3643	HMDB0029944	Ascorbalamic acid	C9H13NO8	262.0581349	No
3651	HMDB0014488; C07408; CHEBI:8597	Protriptyline	C19H21N	262.1606959	No
3651	HMDB0014680; C07274; CHEBI:7640	Nortriptyline	C19H21N	262.1606959	No
3651	HMDB0060672; C16660; CHEBI:80646	2-Ethyl-5-methyl-3,3-diphenyl-1-pyrroline	C19H21N	262.1606959	No
3651	HMDB0060993	demethylmaprotiline	C19H21N	262.1606959	No
3653	HMDB0013892; CHEBI:261282	N-Desmethylvenlafaxine	C16H25NO2	262.1813825	No
3653	HMDB0014339; C07153; CHEBI:238661	Tramadol	C16H25NO2	262.1813825	No
3653	HMDB0015646; CHEBI:261281	Desvenlafaxine	C16H25NO2	262.1813825	No
3653	HMDB0029567	Hydroxy-alpha-sanshool	C16H25NO2	262.1813825	No
3653	HMDB0060532	O-Desmethylvenlafaxine	C16H25NO2	262.1813825	No
3665	HMDB0060005; CHEBI:89624	Methylgallic acid-O-sulphate	C8H8O8S	262.9865678	Yes
3665	HMDB0128025	3-hydroxy-5-methoxy-4-(sulfooxy)benzoic acid	C8H8O8S	262.9865678	Yes
3665	HMDB0130454	2-[3,5-dihydroxy-4-(sulfooxy)phenyl]acetic acid	C8H8O8S	262.9865678	Yes
3669	HMDB0000559; CHEBI:1091015	3-Methoxy-4-hydroxyphenylethyleneglycol sulfate	C9H12O7S	263.023584	No
3669	HMDB0003332	3-Methoxy-4-Hydroxyphenylglycol sulfate	C9H12O7S	263.023584	No
3673	HMDB0036544	1''-Acetoxyeugenol acetate	C14H16O5	263.0926781	Yes
3674	HMDB0004259; C05642	Acetyl-N-formyl-5-methoxykynurenamine	C13H16N2O4	263.104004	No
3674	HMDB0006344; C04148; CHEBI:17884	Alpha-N-Phenylacetyl-L-glutamine	C13H16N2O4	263.104004	No
3674	HMDB0061136	di-Hydroxymelatonin	C13H16N2O4	263.104004	No
3676	HMDB0037543	(±)-(Z)-2-(5-Tetradecenyl)cyclobutanone	C18H32O	263.2377257	No
3694	HMDB0060663; C16625	Isoniazid alpha-ketoglutaric acid	C11H11N3O5	264.0629312	Yes
3697	HMDB0059732; CHEBI:89911	2-(2-Phenylacetoxy)propionylglycine	C13H15NO5	264.087368	Yes
3697	HMDB0059772	N-Phenylacetylglutamic acid	C13H15NO5	264.087368	Yes
3724	HMDB0033351; C08506; CHEBI:6841	Methoxybrassinin	C12H14N2OS2	265.0463038	No
3724	HMDB0040790	4-Methoxybrassinin	C12H14N2OS2	265.0463038	No
3726	HMDB0032437	Monoglyceride citrate	C9H14O9	265.0575643	Yes
3726	HMDB0125085	3-oxo-3-[(3,4,5,6-tetrahydroxyoxan-2-yl)methoxy]prop	C9H14O9	265.0575643	Yes
3729	HMDB0014383; C07263; CHEBI:63613	Nevirapine	C15H14N4O	265.1094428	Yes
3731	HMDB0015027; C08090; CHEBI:217108	Dienestrol	C18H18O2	265.1242045	Yes
3731	HMDB0036387	3-Phenylpropyl cinnamate	C18H18O2	265.1242045	Yes
3734	HMDB0001924; C13235; CHEBI:2904	Atenolol	C14H22N2O3	265.1551488	Yes
3734	HMDB0015411; C11696; CHEBI:258351	Practolol	C14H22N2O3	265.1551488	Yes
3734	HMDB0139570	N-(4-aminobutyl)-3-(4-hydroxy-3-methoxyphenyl)prop	C14H22N2O3	265.1551488	Yes
3735	HMDB0033917	4-Hydroxy-3-methoxy-2,10-bisaboladien-9-one	C16H26O3	265.1800579	No
3735	HMDB0060055; CHEBI:89569	Tetranor 12-HETE	C16H26O3	265.1800579	No
3735	HMDB0112085	3,4-Dimethyl-5-pentyl-2-furanpentanoic acid	C16H26O3	265.1800579	No
3735	HMDB0112088	5-Hexyl-2-furanhexanoic acid	C16H26O3	265.1800579	No
3735	HMDB0112089	3,4-Dimethyl-5-propyl-2-furanheptanoic acid	C16H26O3	265.1800579	No
3735	HMDB0112092	5-Pentyl-2-furanheptanoic acid	C16H26O3	265.1800579	No
3735	HMDB0112097	5-Butyl-2-furanoctanoic acid	C16H26O3	265.1800579	No
3736	HMDB0031050	Methyl 2E,4Z-hexadecadienoate	C17H30O2	265.2178304	No
3737	HMDB0030965; CHEBI:417823	9-Octadecenal	C18H34O	265.2536199	No
3737	HMDB0037517	2-Tetradecylcyclobutanone	C18H34O	265.2536199	No
3756	HMDB0000050; C00212; CHEBI:16335	Adenosine	C10H13N5O4	266.0878176	Yes

3756	HMDB0000085; C00330; CHEBI:17172	Deoxyguanosine	C10H13N5O4	266.0878176	Yes
3756	HMDB0000830; C06469; CHEBI:49022	Neuraminic acid	C9H17NO8	266.0878176	Yes
3756	HMDB0014340; C07195; CHEBI:45327	Vidarabine	C10H13N5O4	266.0878176	Yes
3756	HMDB0014638; C07210; CHEBI:10110	Zidovudine	C10H13N5O4	266.0878176	Yes
3756	HMDB0037842	N-(1-Deoxy-1-fructosyl)serine	C9H17NO8	266.0878176	Yes
3778	HMDB0000575; C01817; CHEBI:17485	DL-Homocystine	C8H16N2O4S2	267.0485364	Yes
3778	HMDB0000676; C01817; CHEBI:89698	L-Homocystine	C8H16N2O4S2	267.0485364	Yes
3778	HMDB0128678	3-(6,7-dimethoxy-2H-1,3-benzodioxol-5-yl)oxirane-2-ca	C12H12O7	267.0485364	Yes
3778	HMDB0128681	(2Z)-3-(6,7-dimethoxy-2H-1,3-benzodioxol-5-yl)-2-hydr	C12H12O7	267.0485364	Yes
3779	HMDB0000195; C00294; CHEBI:17596	Inosine	C10H12N4O5	267.0739435	Yes
3779	HMDB0000481; CHEBI:74074	Allopurinol riboside	C10H12N4O5	267.0739435	Yes
3779	HMDB0003040; CHEBI:705435	Arabinosylhypoxanthine	C10H12N4O5	267.0739435	Yes
3781	HMDB0029051	Serinyl-Tyrosine	C12H16N2O5	267.0969207	Yes
3781	HMDB0029114	Tyrosyl-Serine	C12H16N2O5	267.0969207	Yes
3782	HMDB0032056	Eugenyl benzoate	C17H16O3	267.1043208	Yes
3783	HMDB0012881	Acetylcarnosine	C11H16N4O4	267.1099979	Yes
3783	HMDB0014524; CHEBI:50223	Dexrazoxane	C11H16N4O4	267.1099979	Yes
3783	HMDB0014692; C02267	Pentostatin	C11H16N4O4	267.1099979	Yes
3783	HMDB0028865	Hydroxyprolyl-Histidine	C11H16N4O4	267.1099979	Yes
3783	HMDB0028886	Histidinyl-Hydroxyproline	C11H16N4O4	267.1099979	Yes
3784	HMDB0038935	Kamahine C	C14H20O5	267.1236405	Yes
3784	HMDB0061643	3-carboxy-4-methyl-5-pentyl-2-furanpropanoic acid	C14H20O5	267.1236405	Yes
3786	HMDB0036150	4,11,13,15-Tetrahydroridentin B	C15H24O4	267.1600664	Yes
3786	HMDB0038153	1-(3-Furanyl)-6,7-dihydroxy-4,8-dimethyl-1-nonanone	C15H24O4	267.1600664	Yes
3786	HMDB0038154	3,11,12-Trihydroxy-1(10)-spirovetiven-2-one	C15H24O4	267.1600664	Yes
3786	HMDB0038165	Acoric acid	C15H24O4	267.1600664	Yes
3786	HMDB0038503	Bisacurone epoxide	C15H24O4	267.1600664	Yes
3786	HMDB0060570	Deoxydihydro-artemisinin	C15H24O4	267.1600664	Yes
3786	HMDB0240213	Prostaglandin lactone-diol	C15H24O4	267.1600664	Yes
3788	HMDB0030997; C12100	Cyclohexaneundecanoic acid	C17H32O2	267.2324672	No
3788	HMDB0031046; C16536	(Z)-9-Heptadecenoic acid	C17H32O2	267.2324672	No
3788	HMDB0040888	2,4-Heptadecanedione	C17H32O2	267.2324672	No
3788	HMDB0060038; CHEBI:75094	10Z-Heptadecenoic acid	C17H32O2	267.2324672	No
3788	HMDB0061858	7-Hexadecenoic acid, methyl ester	C17H32O2	267.2324672	No
3788	HMDB0062437; CHEBI:84328	9Z-heptadecenoic acid	C17H32O2	267.2324672	No
3789	HMDB0002384; C01838; CHEBI:17034	Stearaldehyde	C18H36O	267.268957	No
3789	HMDB0029632	Oleyl alcohol	C18H36O	267.268957	No
3789	HMDB0062710; CHEBI:73504	(9Z)-octadecen-1-ol	C18H36O	267.268957	No
3801	HMDB0013852; CHEBI:53016	Sulfamethoxazole N4-hydroxylamine	C10H11N3O4S	268.0402122	No
3801	HMDB0060529	5-Hydroxysulfamethoxazole	C10H11N3O4S	268.0402122	No
3801	HMDB0060985	sulfamethoxazole hydroxylamine	C10H11N3O4S	268.0402122	No
3806	HMDB0015088; CHEBI:48273	Rizatriptan	C15H19N5	268.1560981	No
3808	HMDB0015304; C07935; CHEBI:7789	Orphenadrine	C18H23NO	268.1700241	No
3808	HMDB0144299	3-methyl-4-(methylamino)-1,2-diphenylbutan-2-ol	C18H23NO	268.1700241	No
3826	HMDB0002124; C01477; CHEBI:18388	Apigenin	C15H10O5	269.0466268	Yes
3826	HMDB0003217; C06563; CHEBI:28088	Genistein	C15H10O5	269.0466268	Yes
3826	HMDB0029372; C02495; CHEBI:27479	2''-Hydroxydaidzein	C15H10O5	269.0466268	Yes
3826	HMDB0029521; C10044; CHEBI:5262	Norizalpinin	C15H10O5	269.0466268	Yes
3826	HMDB0030829; C10294; CHEBI:161756	Aloeemodin	C15H10O5	269.0466268	Yes
3826	HMDB0031715; C14314	6-Hydroxydaidzein	C15H10O5	269.0466268	Yes
3826	HMDB0033790; C10379; CHEBI:7635	1,2,8-Trihydroxy-3-methylanthraquinone	C15H10O5	269.0466268	Yes
3826	HMDB0033860	8-Hydroxydaidzein	C15H10O5	269.0466268	Yes
3826	HMDB0034004; C10037	3,4'',7-Trihydroxyflavone	C15H10O5	269.0466268	Yes
3826	HMDB0034409	Rhababerone	C15H10O5	269.0466268	Yes
3826	HMDB0035214; C10343; CHEBI:42223	Emodin	C15H10O5	269.0466268	Yes
3826	HMDB0039449	Rheinanthrone	C15H10O5	269.0466268	Yes
3826	HMDB0041655; C14313; CHEBI:50399	3''-Hydroxydaidzein	C15H10O5	269.0466268	Yes
3826	HMDB0126019	5,7-dihydroxy-2-(3-hydroxyphenyl)-4H-chromen-4-one	C15H10O5	269.0466268	Yes
3826	HMDB0129338	5,7-dihydroxy-3-(3-hydroxyphenyl)-4H-chromen-4-one	C15H10O5	269.0466268	Yes
3826	HMDB0129963	5,6,7-trihydroxy-3-phenyl-4H-chromen-4-one	C15H10O5	269.0466268	Yes
3826	HMDB0130170	5,7,8-trihydroxy-2-phenyl-4H-chromen-4-one	C15H10O5	269.0466268	Yes

3826	HMDB0130780	5,7-dihydroxy-3-(2-hydroxyphenyl)-4H-chromen-4-one	C15H10O5	269.0466268	Yes
3826	HMDB0132880	6,7,8-trihydroxy-3-phenyl-4H-chromen-4-one	C15H10O5	269.0466268	Yes
3826	HMDB0132951	[4-(4-methyl-3-oxopent-1-en-1-yl)phenyl]oxidanesulfo	C12H14O5S	269.0466268	Yes
3826	HMDB0133311	2-(3,4-dihydroxyphenyl)-7-hydroxy-4H-chromen-4-one	C15H10O5	269.0466268	Yes
3826	HMDB0133316	6,7-dihydroxy-2-(3-hydroxyphenyl)-4H-chromen-4-one	C15H10O5	269.0466268	Yes
3826	HMDB0133990	6,7-dihydroxy-3-(3-hydroxyphenyl)-4H-chromen-4-one	C15H10O5	269.0466268	Yes
3826	HMDB0134890	5,6,7-trihydroxy-2-phenyl-4H-chromen-4-one	C15H10O5	269.0466268	Yes
3826	HMDB0137513	5,6-dihydroxy-2-[(4-hydroxyphenyl)methylidene]-2,3-c	C15H10O5	269.0466268	Yes
3826	HMDB0137514	6,7-dihydroxy-2-[(4-hydroxyphenyl)methylidene]-2,3-c	C15H10O5	269.0466268	Yes
3826	HMDB0137515	4,6-dihydroxy-2-[(4-hydroxyphenyl)methylidene]-2,3-c	C15H10O5	269.0466268	Yes
3826	HMDB0137516	2-[(3,4-dihydroxyphenyl)methylidene]-6-hydroxy-2,3-c	C15H10O5	269.0466268	Yes
3826	HMDB0137517	2-[(2,4-dihydroxyphenyl)methylidene]-6-hydroxy-2,3-c	C15H10O5	269.0466268	Yes
3826	HMDB0140468	2-(3,4,5-trihydroxyphenyl)-4H-chromen-4-one	C15H10O5	269.0466268	Yes
3827	HMDB0059806	Phenylglucuronide	C12H14O7	269.0670278	Yes
3827	HMDB0060014; CHEBI:64681	Phenol glucuronide	C12H14O7	269.0670278	Yes
3827	HMDB0135168	3,4,5-trihydroxy-6-phenoxyoxane-2-carboxylic acid	C12H14O7	269.0670278	Yes
3829	HMDB0028755	Aspartyl-Histidine	C10H14N4O5	269.0888369	No
3829	HMDB0028881	Histidinyl-Aspartate	C10H14N4O5	269.0888369	No
3829	HMDB0029955	D-erythro-L-galacto-Nonulose	C9H18O9	269.0888369	No
3836	HMDB0010733; CHEBI:37251	3-Oxohexadecanoic acid	C16H30O3	269.2124171	No
3836	HMDB0030972	8-Oxohexadecanoic acid	C16H30O3	269.2124171	No
3836	HMDB0030973	9-Oxohexadecanoic acid	C16H30O3	269.2124171	No
3836	HMDB0030978	11-Oxohexadecanoic acid	C16H30O3	269.2124171	No
3837	HMDB0002259; CHEBI:32365	Heptadecanoic acid	C17H34O2	269.2484881	No
3837	HMDB0031067; CHEBI:84874	(S)-14-Methylhexadecanoic acid	C17H34O2	269.2484881	No
3837	HMDB0034131	Nonyl octanoate	C17H34O2	269.2484881	No
3837	HMDB0037372; CHEBI:87343	3-Methylbutyl dodecanoate	C17H34O2	269.2484881	No
3837	HMDB0040392	Isopropyl tetradecanoate	C17H34O2	269.2484881	No
3837	HMDB0040598	(±)-11-Methylhexadecanoic acid	C17H34O2	269.2484881	No
3837	HMDB0059870	Ethyl pentadecanoate	C17H34O2	269.2484881	No
3837	HMDB0061859; C16995	Methyl hexadecanoic acid	C17H34O2	269.2484881	No
3855	HMDB0011654; C04692; CHEBI:16475	2-(3-Carboxy-3-(methylammonio)propyl)-L-histidine	C11H19N4O4	270.1348002	No
3859	HMDB0001920; C06947; CHEBI:4470	Dextromethorphan	C18H25NO	270.1859492	No
3860	HMDB0013317; CHEBI:74437	Tridecanoylglycine	C15H29NO3	270.2078076	No
3879	HMDB0128634	[(7-hydroxy-1-oxo-1H-isochromen-3-yl)methoxy]sulfor	C10H8O7S	270.9920928	Yes
3879	HMDB0128636	[(6-hydroxy-1-oxo-1H-isochromen-3-yl)methoxy]sulfor	C10H8O7S	270.9920928	Yes
3879	HMDB0128638	[(5-hydroxy-1-oxo-1H-isochromen-3-yl)methoxy]sulfor	C10H8O7S	270.9920928	Yes
3880	HMDB0059993; CHEBI:88750	5-(Hydroxyphenyl)-gamma-valerolactone-O-sulphate	C11H12O6S	271.0285929	Yes
3880	HMDB0126424	[(5-hydroxy-2-methyl-2H-chromen-2-yl)methoxy]sulfo	C11H12O6S	271.0285929	Yes
3880	HMDB0129400	[1-(4-methoxy-1-benzofuran-5-yl)ethoxy]sulfonic acid	C11H12O6S	271.0285929	Yes
3880	HMDB0135674	[2-methoxy-4-(3-oxobut-1-en-1-yl)phenyl]oxidanesulfo	C11H12O6S	271.0285929	Yes
3881	HMDB0002670; C00509; CHEBI:17846	Naringenin	C15H12O5	271.0615479	Yes
3881	HMDB0005897; C14458	Dihydrogenistein	C15H12O5	271.0615479	Yes
3881	HMDB0029631; C06561; CHEBI:15413	Chalconaringenin	C15H12O5	271.0615479	Yes
3881	HMDB0030873	1-Hydroxy-3,7-dimethoxyxanthone	C15H12O5	271.0615479	Yes
3881	HMDB0031824	4'',5,8-Trihydroxyflavanone	C15H12O5	271.0615479	Yes
3881	HMDB0033308	Moracin J	C15H12O5	271.0615479	Yes
3881	HMDB0033928; C03567; CHEBI:16035	(±)-2''-Hydroxydihydrodaidzein	C15H12O5	271.0615479	Yes
3881	HMDB0034002	3,4,9-Trihydroxypterocarpan	C15H12O5	271.0615479	Yes
3881	HMDB0034105; C01263; CHEBI:15649	Glycinol	C15H12O5	271.0615479	Yes
3881	HMDB0037254; C09751	(2R,3R)-3,4'',7-Trihydroxyflavanone	C15H12O5	271.0615479	Yes
3881	HMDB0038549; C17673; CHEBI:78029	Toralactone	C15H12O5	271.0615479	Yes
3881	HMDB0041656	3''-Hydroxydihydrodaidzein	C15H12O5	271.0615479	Yes
3881	HMDB0041698	6-Hydroxydihydrodaidzein	C15H12O5	271.0615479	Yes
3881	HMDB0041704	8-Hydroxydihydrodaidzein	C15H12O5	271.0615479	Yes
3881	HMDB0125364	3,5,7-trihydroxy-2-phenyl-3,4-dihydro-2H-1-benzopyra	C15H12O5	271.0615479	Yes
3881	HMDB0125365	3-phenyl-1-(2,4,6-trihydroxyphenyl)propane-1,2-dione	C15H12O5	271.0615479	Yes
3881	HMDB0126021	5,7-dihydroxy-2-(3-hydroxyphenyl)-3,4-dihydro-2H-1-b	C15H12O5	271.0615479	Yes
3881	HMDB0126022	3-(3-hydroxyphenyl)-1-(2,4,6-trihydroxyphenyl)prop-2-	C15H12O5	271.0615479	Yes
3881	HMDB0128074	5,7-dihydroxy-2-(4-hydroxyphenyl)-3,4-dihydro-2H-1-b	C15H12O5	271.0615479	Yes
3881	HMDB0128075	3-(4-hydroxyphenyl)-1-(2,4,6-trihydroxyphenyl)prop-2-	C15H12O5	271.0615479	Yes

3881	HMDB0129341	5,7-dihydroxy-3-(3-hydroxyphenyl)-3,4-dihydro-2H-1-b	C15H12O5	271.0615479	Yes
3881	HMDB0129965	5,6,7-trihydroxy-3-phenyl-3,4-dihydro-2H-1-benzopyra	C15H12O5	271.0615479	Yes
3881	HMDB0130173	5,7,8-trihydroxy-2-phenyl-3,4-dihydro-2H-1-benzopyra	C15H12O5	271.0615479	Yes
3881	HMDB0130182	3-phenyl-1-(2,3,4,6-tetrahydroxyphenyl)prop-2-en-1-o	C15H12O5	271.0615479	Yes
3881	HMDB0133545	2-benzyl-2,4,6-trihydroxy-2,3-dihydro-1-benzofuran-3-	C15H12O5	271.0615479	Yes
3881	HMDB0133993	6,7-dihydroxy-3-(3-hydroxyphenyl)-3,4-dihydro-2H-1-b	C15H12O5	271.0615479	Yes
3881	HMDB0133999	6,7,8-trihydroxy-3-phenyl-3,4-dihydro-2H-1-benzopyra	C15H12O5	271.0615479	Yes
3881	HMDB0134891	5,6,7-trihydroxy-2-phenyl-3,4-dihydro-2H-1-benzopyra	C15H12O5	271.0615479	Yes
3881	HMDB0134892	(2E)-3-phenyl-1-(2,3,4,6-tetrahydroxyphenyl)prop-2-er	C15H12O5	271.0615479	Yes
3881	HMDB0137525	6,7-dihydroxy-2-[(4-hydroxyphenyl)methyl]-2,3-dihydr	C15H12O5	271.0615479	Yes
3881	HMDB0137528	4,6-dihydroxy-2-[(4-hydroxyphenyl)methyl]-2,3-dihydr	C15H12O5	271.0615479	Yes
3881	HMDB0137531	2-[(3,4-dihydroxyphenyl)methyl]-6-hydroxy-2,3-dihydr	C15H12O5	271.0615479	Yes
3881	HMDB0138088	8,17-dioxatetracyclo[8.7.0.0²,?.0¹¹,¹?]heptadeca-2(7),3,	C15H12O5	271.0615479	Yes
3881	HMDB0138618	5-(6-hydroxy-5-methoxy-1-benzofuran-2-yl)benzene-1,	C15H12O5	271.0615479	Yes
3883	HMDB0029943; C06186; CHEBI:18305	Arbutin	C12H16O7	271.0823895	Yes
3884	HMDB0014632; C07309; CHEBI:63622	Sotalol	C12H20N2O3S	271.11245	Yes
3884	HMDB0060341; CHEBI:82517	1a,11b-Dihydro-4,9-dimethylbenz[a]anthra[3,4-b]oxire	C20H16O	271.11245	Yes
3884	HMDB0060419	7-Hydroxymethyl-12-methylbenz[a]anthracene	C20H16O	271.11245	Yes
3884	HMDB0060422; CHEBI:82590	7,12-Dimethylbenz[a]anthracene 5,6-oxide	C20H16O	271.11245	Yes
3888	HMDB0035918	7(14)-Bisabolene-2,3,10,11-tetrol	C15H28O4	271.1910848	Yes
3889	HMDB0010734; CHEBI:38247	(R)-3-Hydroxy-hexadecanoic acid	C16H32O3	271.2280483	No
3889	HMDB0031057; CHEBI:65101	(R)-2-Hydroxyhexadecanoic acid	C16H32O3	271.2280483	No
3889	HMDB0061658; CHEBI:37248	3-hydroxyhexadecanoic acid	C16H32O3	271.2280483	No
3889	HMDB0112184	5-Hydroxyhexadecanoic acid	C16H32O3	271.2280483	No
3889	HMDB0112185	7-Hydroxyhexadecanoic acid	C16H32O3	271.2280483	No
3889	HMDB0112186	8-Hydroxyhexadecanoic acid	C16H32O3	271.2280483	No
3889	HMDB0112187	9-Hydroxyhexadecanoic acid	C16H32O3	271.2280483	No
3889	HMDB0112188	10-Hydroxyhexadecanoic acid	C16H32O3	271.2280483	No
3889	HMDB0112189	11-Hydroxyhexadecanoic acid	C16H32O3	271.2280483	No
3889	HMDB0112190	12-Hydroxyhexadecanoic acid	C16H32O3	271.2280483	No
3889	HMDB0112191	13-Hydroxyhexadecanoic acid	C16H32O3	271.2280483	No
3889	HMDB0112192	6-Hydroxyhexadecanoic acid	C16H32O3	271.2280483	No
3901	HMDB0062800; CHEBI:58759	6-phosphonatooxy-D-gluconate	C6H10O10P	271.9939305	Yes
3902	HMDB0015492	Stepronin	C10H11NO4S2	272.0044604	No
3906	HMDB0014468; C07949; CHEBI:63630	Tolcapone	C14H11NO5	272.0546661	Yes
3909	HMDB0000667; CHEBI:30662	L-Thyronine	C15H15NO4	272.0900062	No
3909	HMDB0062356; CHEBI:30661	3''-monoiodo-L-thyronine	C15H15NO4	272.0900062	No
3912	HMDB0030187; CHEBI:520668	(E,E)-Piperlonguminine	C16H19NO3	272.1295715	No
3912	HMDB0038834	(2E)-Piperamide-C5:1	C16H19NO3	272.1295715	No
3916	HMDB0013238; CHEBI:74303	Heptanoylcarnitine	C14H27NO4	272.1865508	No
3918	HMDB0031184	N-Isobutyl-2,4,8,10,12-tetradecapentaenamide	C18H27NO	272.2022429	No
3940	HMDB0029200; CHEBI:133508	Ferulic acid 4-sulfate	C10H10O7S	273.0078497	No
3940	HMDB0041748	Isoferulic acid 3-sulfate	C10H10O7S	273.0078497	No
3940	HMDB0127955	3-[3-methoxy-4-(sulfooxy)phenyl]prop-2-enoic acid	C10H10O7S	273.0078497	No
3940	HMDB0131141	3-[4-methoxy-3-(sulfooxy)phenyl]prop-2-enoic acid	C10H10O7S	273.0078497	No
3943	HMDB0059981; CHEBI:88703	4-Hydroxy-5-(phenyl)-valeric acid-O-sulphate	C11H14O6S	273.0444891	Yes
3943	HMDB0126242	{[4-(2,6-dihydroxyphenyl)-2-methylbut-2-en-1-yl]oxy}s	C11H14O6S	273.0444891	Yes
3943	HMDB0126373	{[(2E)-4-(2,6-dihydroxyphenyl)-2-methylbut-2-en-1-yl]c	C11H14O6S	273.0444891	Yes
3943	HMDB0133253	{[(2E)-4-(2,4-dihydroxyphenyl)-2-methylbut-2-en-1-yl]c	C11H14O6S	273.0444891	Yes
3943	HMDB0133255	{[4-(2,4-dihydroxyphenyl)-2-methylbut-2-en-1-yl]oxy}s	C11H14O6S	273.0444891	Yes
3943	HMDB0133259	[2,6-dihydroxy-3-(3-methylbut-2-en-1-yl)phenyl]oxidar	C11H14O6S	273.0444891	Yes
3943	HMDB0135676	[2-methoxy-4-(3-oxobutyl)phenyl]oxidanesulfonic acid	C11H14O6S	273.0444891	Yes
3943	HMDB0135698	[4-(3-hydroxybut-1-en-1-yl)-2-methoxyphenyl]oxidane	C11H14O6S	273.0444891	Yes
3943	HMDB0135699	{[4-(4-hydroxy-3-methoxyphenyl)but-3-en-2-yl]oxy}sul	C11H14O6S	273.0444891	Yes
3944	HMDB0013870; CHEBI:212535	cis,trans-5''-Hydroxythalidomide	C13H10N2O5	273.0538891	Yes
3944	HMDB0013871; CHEBI:405332	5-Hydroxythalidomide	C13H10N2O5	273.0538891	Yes
3944	HMDB0013872	Thalidomide arene oxide	C13H10N2O5	273.0538891	Yes
3944	HMDB0041120	4-Phenyl-1H,3H-naphtho[1,8-cd]pyran-1,3-dione	C18H10O3	273.0538891	Yes
3945	HMDB0133010	{[1-(4-methoxyphenyl)pentan-3-yl]oxy}sulfonic acid	C12H18O5S	273.0805836	No
3946	HMDB0032173	Bakers yeast extract	C19H14O2	273.0931793	Yes
3948	HMDB0028950	Lysyl-Glutamate	C11H20N3O5	273.1338794	Yes

3950	HMDB0000394	3-Hydroxytetradecanedioic acid	C14H26O5	273.171044	No
3953	HMDB0014441; C07532; CHEBI:8890	Ropivacaine	C17H26N2O	273.196513	No
3953	HMDB0037992	Cyanidin 3-(diferuloylsophoroside) 5-glucoside	C17H26N2O	273.196513	No
3954	HMDB0072858	MG(0:0/i-12:0/0:0)	C15H30O4	273.2062264	No
3954	HMDB0072860	MG(i-12:0/0:0/0:0)	C15H30O4	273.2062264	No
3954	HMDB0072863	MG(12:0/0:0/0:0)	C15H30O4	273.2062264	No
3954	HMDB0072869	MG(0:0/12:0/0:0)	C15H30O4	273.2062264	No
3975	HMDB0001786	Ethenodeoxyadenosine	C12H13N5O3	274.0939123	No
3975	HMDB0030697; C08340	Sarmentosin	C11H17NO7	274.0939123	No
3977	HMDB0041488	4,5-Dihydropiperlonguminine	C16H21NO3	274.1441473	No
3977	HMDB0060989	4''-hydroxypropanolol	C16H21NO3	274.1441473	No
3992	HMDB0041724	Dihydroferulic acid 4-sulfate	C10H12O7S	275.0228436	Yes
3992	HMDB0131144	3-[4-methoxy-3-(sulfooxy)phenyl]propanoic acid	C10H12O7S	275.0228436	Yes
3996	HMDB0033649; CHEBI:133763	O-Demethylfonsecin	C14H12O6	275.0573853	Yes
3996	HMDB0135717	[4-(3-hydroxybutyl)-2-methoxyphenyl]oxidanesulfonic	C11H16O6S	275.0573853	Yes
3996	HMDB0135718	{[4-(4-hydroxy-3-methoxyphenyl)butan-2-yl]oxy}sulfor	C11H16O6S	275.0573853	Yes
3999	HMDB0030764	Acetylpterosin C	C16H20O4	275.1291298	Yes
4000	HMDB0015586; CHEBI:3988	Cyclandelate	C17H24O3	275.1647156	No
4000	HMDB0031463; C10494	[8]-Shogaol	C17H24O3	275.1647156	No
4000	HMDB0038938	Panaquinquecol 2	C17H24O3	275.1647156	No
4000	HMDB0038994	Ginsenoyne C	C17H24O3	275.1647156	No
4000	HMDB0040375	Ginsenoyne K	C17H24O3	275.1647156	No
4000	HMDB0041201	Panaquinquecol 7	C17H24O3	275.1647156	No
4000	HMDB0137236	(4E)-1-(4-hydroxy-3-methoxyphenyl)dec-4-en-3-one	C17H24O3	275.1647156	No
4000	HMDB0137237	(1E)-1-(4-hydroxy-3-methoxyphenyl)dec-1-en-3-one	C17H24O3	275.1647156	No
4001	HMDB0015555; C07230; CHEBI:103799	Molindone	C16H24N2O2	275.1764175	No
4001	HMDB0061010	Noralfentanil	C16H24N2O2	275.1764175	No
4003	HMDB0002697; CHEBI:36412	19-Norandrosterone	C18H28O2	275.2021822	No
4003	HMDB0004590; CHEBI:529214	19-Nor-5-androstenediol	C18H28O2	275.2021822	No
4003	HMDB0005886; CHEBI:36412	19-Noretiocholanolone	C18H28O2	275.2021822	No
4003	HMDB0006547; C16300; CHEBI:32389	Stearidonic acid	C18H28O2	275.2021822	No
4003	HMDB0032464	Phenethyl decanoate	C18H28O2	275.2021822	No
4003	HMDB0032672	4,8,12,15-Octadecatetraenoic acid	C18H28O2	275.2021822	No
4003	HMDB0034382	(E,E)-11,13-Octadecadien-9-ynoic acid	C18H28O2	275.2021822	No
4003	HMDB0062677; CHEBI:87333	17beta-hydroxy-5beta-estran-3-one	C18H28O2	275.2021822	No
4018	HMDB0001495; C01449; CHEBI:17433	Queuine	C12H15N5O3	276.1097584	Yes
4018	HMDB0006868; C04424; CHEBI:17577	S-(2-Methylpropionyl)-dihydrolipoamide-E	C12H23NO2S2	276.1097584	Yes
4018	HMDB0014585; CHEBI:473990	Entecavir	C12H15N5O3	276.1097584	Yes
4018	HMDB0038493	N-(1-Deoxy-1-fructosyl)proline	C11H19NO7	276.1097584	Yes
4020	HMDB0036325	Dinorcapsaicin	C16H23NO3	276.1609993	No
4021	HMDB0041944; CHEBI:60728	N-Acetylprocainamide	C15H23N3O2	276.1722325	No
4037	HMDB0124971	2-hydroxy-3-[4-hydroxy-3-(sulfooxy)phenyl]propanoic	C9H10O8S	277.0009085	Yes
4037	HMDB0124972	3-(3,4-dihydroxyphenyl)-2-(sulfooxy)propanoic acid	C9H10O8S	277.0009085	Yes
4037	HMDB0128014	3-[3,5-dihydroxy-4-(sulfooxy)phenyl]propanoic acid	C9H10O8S	277.0009085	Yes
4037	HMDB0131461	3,5-dimethoxy-4-(sulfooxy)benzoic acid	C9H10O8S	277.0009085	Yes
4038	HMDB0033209; C14420; CHEBI:34761	Fenthion	C10H15O3PS2	277.0119708	No
4042	HMDB0015522; C19530; CHEBI:102265	Sulfamethazine	C12H14N4O2S	277.0754847	Yes
4043	HMDB0028825; CHEBI:73507	Glutamylmethionine	C10H18N2O5S	277.0842682	No
4043	HMDB0034367; HMDB0029155; CHEBI:8296!	gamma-Glutamylmethionine	C10H18N2O5S	277.0842682	No
4044	HMDB0002091	Isovalerylglucuronide	C11H18O8	277.0926217	Yes
4044	HMDB0126546	3,4,5-trihydroxy-6-[(oxolan-2-yl)methoxy]oxane-2-carb	C11H18O8	277.0926217	Yes
4044	HMDB0130798	3,4,5-trihydroxy-6-[(3-methylbutanoyl)oxy]oxane-2-ca	C11H18O8	277.0926217	Yes
4045	HMDB0030766	11-Hydroxy-12-methoxydihydrokawain	C15H18O5	277.1084915	No
4045	HMDB0033262	Solanolone	C15H18O5	277.1084915	No
4045	HMDB0036049	Marasmone	C15H18O5	277.1084915	No
4045	HMDB0036052	Isomarasmone	C15H18O5	277.1084915	No
4045	HMDB0036662	2beta,9xi-Dihydroxy-8-oxo-1(10),4,11(13)-germacratri	C15H18O5	277.1084915	No
4045	HMDB0037325	Achimilic acid	C15H18O5	277.1084915	No
4045	HMDB0131686	6-[2-(2H-1,3-benzodioxol-5-yl)ethyl]-4-methoxyoxan-2	C15H18O5	277.1084915	No
4046	HMDB0011112; C05775; CHEBI:10329	N1-(alpha-D-ribosyl)-5,6-dimethyl-benzimidazole	C14H18N2O4	277.1184915	No
4046	HMDB0011176	L-phenylalanyl-L-hydroxyproline	C14H18N2O4	277.1184915	No

4046	HMDB0028996	Phenylalanyl-Hydroxyproline	C14H18N2O4	277.1184915	No
4046	HMDB0029029	Prolyl-Tyrosine	C14H18N2O4	277.1184915	No
4046	HMDB0029113	Tyrosyl-Proline	C14H18N2O4	277.1184915	No
4048	HMDB0001518; CHEBI:626842	Alpha-CEHC	C16H22O4	277.1439528	Yes
4048	HMDB0013248; C03343; CHEBI:17243	Monoethylhexyl phthalic acid	C16H22O4	277.1439528	Yes
4048	HMDB0013835; C15205; CHEBI:894546	Diisobutyl phthalate	C16H22O4	277.1439528	Yes
4048	HMDB0033244; C14214; CHEBI:535597	Dibutyl phthalate	C16H22O4	277.1439528	Yes
4048	HMDB0133045	2-(4-hydroxy-3-methylbut-2-en-1-yl)-4-(3-methylbut-2	C16H22O4	277.1439528	Yes
4048	HMDB0133047	bis(3-methylbut-2-en-1-yl)benzene-1,2,3,5-tetrol	C16H22O4	277.1439528	Yes
4048	HMDB0133048	2-[(3,3-dimethyloxiran-2-yl)methyl]-4-(3-methylbut-2-	C16H22O4	277.1439528	Yes
4048	HMDB0133084	4-(3,7-dimethylocta-2,6-dien-1-yl)benzene-1,2,3,5-tetr	C16H22O4	277.1439528	Yes
4048	HMDB0133085	2-{[3-methyl-3-(4-methylpent-3-en-1-yl)oxiran-2-yl]me	C16H22O4	277.1439528	Yes
4048	HMDB0133086	2-[5-(3,3-dimethyloxiran-2-yl)-3-methylpent-2-en-1-yl]	C16H22O4	277.1439528	Yes
4048	HMDB0133087	2-[(2Z)-4-hydroxy-3-(4-methylpent-3-en-1-yl)but-2-en-	C16H22O4	277.1439528	Yes
4048	HMDB0133088	2-(4-hydroxy-3,7-dimethylocta-2,6-dien-1-yl)benzene-	C16H22O4	277.1439528	Yes
4048	HMDB0133089	2-(5-hydroxy-3,7-dimethylocta-2,6-dien-1-yl)benzene-	C16H22O4	277.1439528	Yes
4048	HMDB0133090	2-[(6E)-8-hydroxy-3,7-dimethylocta-2,6-dien-1-yl]benz	C16H22O4	277.1439528	Yes
4048	HMDB0133091	2-(8-hydroxy-3,7-dimethylocta-2,6-dien-1-yl)benzene-	C16H22O4	277.1439528	Yes
4049	HMDB0013243; C11221; CHEBI:383437	Leucyl-phenylalanine	C15H22N2O3	277.1561516	No
4049	HMDB0028914	Isoleucyl-Phenylalanine	C15H22N2O3	277.1561516	No
4049	HMDB0028998	Phenylalanyl-Isoleucine	C15H22N2O3	277.1561516	No
4049	HMDB0062483; CHEBI:111173	keratan sulfate I	C15H22N2O3	277.1561516	No
4050	HMDB0035028	(x)-2-Heptanol glucoside	C13H26O6	277.1647743	No
4051	HMDB0014571; CHEBI:165539	Triprolidine	C19H22N2	277.1707516	No
4053	HMDB0060931	2-ethyl-1,5-dimethyl-3,3-diphenylpyrrolinium (EDDP)	C20H24N	277.1845473	No
4054	HMDB0001388; C06427; CHEBI:27432	Alpha-Linolenic acid	C18H30O2	277.2173802	No
4054	HMDB0003073; C06426; CHEBI:28661	Gamma-Linolenic acid	C18H30O2	277.2173802	No
4054	HMDB0030962; CHEBI:86148	Calendic acid	C18H30O2	277.2173802	No
4054	HMDB0030963; C08364; CHEBI:38383	Punicic acid	C18H30O2	277.2173802	No
4054	HMDB0030964	Linolenelaidic acid	C18H30O2	277.2173802	No
4070	HMDB0032807	Niazirin	C14H17NO5	278.1051245	No
4075	HMDB0013889	E-10-Hydroxynortriptyline	C19H21NO	278.1542806	No
4075	HMDB0015273; C06971; CHEBI:36691	Doxepin	C19H21NO	278.1542806	No
4077	HMDB0036326	N-[(4-Hydroxy-3-methoxyphenyl)methyl]octanamide	C16H25NO3	278.1764402	No
4097	HMDB0126113	6-(1-carboxy-1-methylethoxy)-3,4,5-trihydroxyoxane-2	C10H16O9	279.0703938	Yes
4097	HMDB0126114	3,4,5-trihydroxy-6-[(2-hydroxy-2-methylpropanoyl)oxy	C10H16O9	279.0703938	Yes
4097	HMDB0126115	6-(2-carboxy-2-methylethoxy)-3,4,5-trihydroxyoxane-2	C10H16O9	279.0703938	Yes
4097	HMDB0126116	3,4,5-trihydroxy-6-[(3-hydroxy-2-methylpropanoyl)oxy	C10H16O9	279.0703938	Yes
4097	HMDB0130019	1-methyl 3-(3,4,5,6-tetrahydroxyoxan-2-yl)methyl prop	C10H16O9	279.0703938	Yes
4097	HMDB0130129	6-[(1-carboxypropan-2-yl)oxy]-3,4,5-trihydroxyoxane-2	C10H16O9	279.0703938	Yes
4097	HMDB0130130	3,4,5-trihydroxy-6-[(3-hydroxybutanoyl)oxy]oxane-2-ca	C10H16O9	279.0703938	Yes
4097	HMDB0130132	6-(3-carboxypropoxy)-3,4,5-trihydroxyoxane-2-carboxy	C10H16O9	279.0703938	Yes
4097	HMDB0130133	3,4,5-trihydroxy-6-[(4-hydroxybutanoyl)oxy]oxane-2-ca	C10H16O9	279.0703938	Yes
4097	HMDB0130135	6-(1-carboxypropoxy)-3,4,5-trihydroxyoxane-2-carboxy	C10H16O9	279.0703938	Yes
4097	HMDB0130136	3,4,5-trihydroxy-6-[(2-hydroxybutanoyl)oxy]oxane-2-ca	C10H16O9	279.0703938	Yes
4099	HMDB0000706; CHEBI:385866	L-Aspartyl-L-phenylalanine	C13H16N2O5	279.0990882	Yes
4099	HMDB0011167	L-beta-aspartyl-L-phenylalanine	C13H16N2O5	279.0990882	Yes
4099	HMDB0015058; CHEBI:50567	Levosimendan	C14H12N6O	279.0990882	Yes
4099	HMDB0028760	Aspartyl-Phenylalanine	C13H16N2O5	279.0990882	Yes
4099	HMDB0028991	Phenylalanyl-Aspartate	C13H16N2O5	279.0990882	Yes
4102	HMDB0035620	Artabsinolide A	C15H20O5	279.1246217	No
4102	HMDB0036051	Dihydromarasmone	C15H20O5	279.1246217	No
4102	HMDB0036094	Nigellic acid	C15H20O5	279.1246217	No
4102	HMDB0036095	13-Hydroxyabscisic acid	C15H20O5	279.1246217	No
4102	HMDB0036695	Crispolide	C15H20O5	279.1246217	No
4102	HMDB0038545	(1beta,4alpha,5alpha,6beta,8alpha,10b)-1,10:4,5-Diep	C15H20O5	279.1246217	No
4102	HMDB0041003	Vulgarolide	C15H20O5	279.1246217	No
4102	HMDB0041144	8-Deoxy-11,13-dihydroxygrosheimin	C15H20O5	279.1246217	No
4102	HMDB0133288	2-hydroxy-3-[4-hydroxy-3-methoxy-5-(3-methylbut-2-	C15H20O5	279.1246217	No
4119	HMDB0003331; C02494; CHEBI:16020	1-Methyladenosine	C11H15N5O4	280.1025104	No
4119	HMDB0004044; CHEBI:21891	N6-Methyladenosine	C11H15N5O4	280.1025104	No

4119	HMDB0004326; C04779; CHEBI:119928	2''-O-Methyladenosine	C11H15N5O4	280.1025104	No
4119	HMDB0006023	3''-O-Methyladenosine	C11H15N5O4	280.1025104	No
4119	HMDB0037843	N-(1-Deoxy-1-fructosyl)threonine	C10H19NO8	280.1025104	No
4123	HMDB0032791	Methyl 15-cyanopentadecanoate	C17H31NO2	280.2289743	No
4126	HMDB0002117; C19670; CHEBI:116314	Oleamide	C18H35NO	280.2645396	No
4141	HMDB0040794	5-Hydroxy-4-methoxy-6-canthinone 3-N-oxide	C15H10N2O4	281.0588451	No
4141	HMDB0060868	Phenytoin quinone	C15H10N2O4	281.0588451	No
4142	HMDB0030698; CHEBI:205093	5,7-Dimethoxyisoflavone	C17H14O4	281.0816979	No
4142	HMDB0030719	5,6-Dimethoxyflavone	C17H14O4	281.0816979	No
4142	HMDB0036620; C10029; CHEBI:3684	5,7-Dimethoxyflavone	C17H14O4	281.0816979	No
4142	HMDB0037255	5-Hydroxy-7-methoxy-6-methylflavone	C17H14O4	281.0816979	No
4142	HMDB0130371	7-methoxy-3-(3-methoxyphenyl)-4H-chromen-4-one	C17H14O4	281.0816979	No
4142	HMDB0140467	2-(3,5-dimethoxyphenyl)-4H-chromen-4-one	C17H14O4	281.0816979	No
4143	HMDB0013911; CHEBI:155605	12-Hydroxynevirapine	C15H14N4O2	281.1017034	Yes
4143	HMDB0013912	2-Hydroxynevirapine	C15H14N4O2	281.1017034	Yes
4143	HMDB0013913	8-Hydroxynevirapine	C15H14N4O2	281.1017034	Yes
4143	HMDB0013914	3-Hydroxynevirapine	C15H14N4O2	281.1017034	Yes
4143	HMDB0125459	2-[4,6-dihydroxy-3-(4-hydroxy-3-methylbut-2-en-1-yl)-	C14H18O6	281.1017034	Yes
4143	HMDB0125461	2-[2,3,4-trihydroxy-6-methoxy-5-(3-methylbut-2-en-1-	C14H18O6	281.1017034	Yes
4143	HMDB0125462	2-{3-[(3,3-dimethyloxiran-2-yl)methyl]-4,6-dihydroxy-2	C14H18O6	281.1017034	Yes
4143	HMDB0125463	2-[4,6-dihydroxy-2-methoxy-3-(3-methylbut-2-en-1-yl)	C14H18O6	281.1017034	Yes
4148	HMDB0033375; CHEBI:83631	Octyl gallate	C15H22O5	281.1405233	Yes
4148	HMDB0034941	Artabsinolide D	C15H22O5	281.1405233	Yes
4148	HMDB0034983	Cynaratriol	C15H22O5	281.1405233	Yes
4148	HMDB0035846	Anguidol	C15H22O5	281.1405233	Yes
4148	HMDB0038660; C15971; CHEBI:23758	Dihydrophaseic acid	C15H22O5	281.1405233	Yes
4148	HMDB0038661	Epidihydrophaseic acid	C15H22O5	281.1405233	Yes
4148	HMDB0038989	Oxyhumulinic acid	C15H22O5	281.1405233	Yes
4148	HMDB0039241	Pisumic acid	C15H22O5	281.1405233	Yes
4148	HMDB0041324	Bisbynin	C15H22O5	281.1405233	Yes
4150	HMDB0061822; CHEBI:49793	Hexaethylene glycol	C12H26O7	281.1607021	No
4151	HMDB0031799; C19039	Malonoben	C18H22N2O	281.1658536	No
4151	HMDB0060992	2-hydroxydesipramine	C18H22N2O	281.1658536	No
4152	HMDB0033630	Lactapiperanol C	C16H26O4	281.1762169	Yes
4156	HMDB0000207; C00712; CHEBI:16196	Oleic acid	C18H34O2	281.2484002	Yes
4156	HMDB0000573; C00712; CHEBI:27997	Elaidic acid	C18H34O2	281.2484002	Yes
4156	HMDB0003231; C08367; CHEBI:28727	Vaccenic acid	C18H34O2	281.2484002	Yes
4156	HMDB0041480; CHEBI:82617	(Z)-13-Octadecenoic acid	C18H34O2	281.2484002	Yes
4156	HMDB0059871	Ethyl 9-hexadecenoate	C18H34O2	281.2484002	Yes
4156	HMDB0062218	(E)-13-Octadecenoic acid	C18H34O2	281.2484002	Yes
4156	HMDB0062242	7Z-octadecenoic acid	C18H34O2	281.2484002	Yes
4156	HMDB0062703; CHEBI:36021	Octadec-9-enoic Acid	C18H34O2	281.2484002	Yes
4156	HMDB0240219; CHEBI:50464	cis-Vaccenic acid	C18H34O2	281.2484002	Yes
4207	HMDB0060791	6-Thioinosinic acid	C10H12N4O4S	283.0495389	Yes
4208	HMDB0002338; C00814; CHEBI:17574	Biochanin A	C16H12O5	283.0618005	Yes
4208	HMDB0005781; C14536; CHEBI:542494	Glycitein	C16H12O5	283.0618005	Yes
4208	HMDB0029522; C11577; CHEBI:1272299	5,7-Dihydroxy-3-methoxyflavone	C16H12O5	283.0618005	Yes
4208	HMDB0030768; CHEBI:103729	2-(6-Hydroxy-2-methoxy-3,4-methylenedioxyphenyl)b	C16H12O5	283.0618005	Yes
4208	HMDB0031428	Farnisin	C16H12O5	283.0618005	Yes
4208	HMDB0031720; C02920; CHEBI:17678	Xenognosin B	C16H12O5	283.0618005	Yes
4208	HMDB0034127; C10521; CHEBI:8600	Prunetin	C16H12O5	283.0618005	Yes
4208	HMDB0036629; CHEBI:73029	(-)-Maackiain	C16H12O5	283.0618005	Yes
4208	HMDB0039166	Cajaquinone	C16H12O5	283.0618005	Yes
4208	HMDB0039578	1,5-Dihydroxy-2-methoxy-6-methylanthraquinone	C16H12O5	283.0618005	Yes
4208	HMDB0041758; C10504	Melanettin	C16H12O5	283.0618005	Yes
4208	HMDB0041776	Stevenin	C16H12O5	283.0618005	Yes
4208	HMDB0128584	5,7-dihydroxy-6-methoxy-2-phenyl-4H-chromen-4-one	C16H12O5	283.0618005	Yes
4208	HMDB0128588	6,7-dihydroxy-5-methoxy-2-phenyl-4H-chromen-4-one	C16H12O5	283.0618005	Yes
4208	HMDB0130320	5,7-dihydroxy-2-(3-methoxyphenyl)-4H-chromen-4-on	C16H12O5	283.0618005	Yes
4208	HMDB0130374	3-(3,4-dihydroxyphenyl)-7-methoxy-4H-chromen-4-on	C16H12O5	283.0618005	Yes
4208	HMDB0130379	7-hydroxy-3-(4-hydroxy-3-methoxyphenyl)-4H-chrome	C16H12O5	283.0618005	Yes

4208	HMDB0130929	7-hydroxy-2-(4-hydroxyphenyl)-6-methoxy-4H-chrome	C16H12O5	283.0618005	Yes
4208	HMDB0132455	7-hydroxy-2-(4-hydroxyphenyl)-5-methoxy-4H-chrome	C16H12O5	283.0618005	Yes
4208	HMDB0132457	5,7-dihydroxy-2-(4-methoxyphenyl)-4H-chromen-4-on	C16H12O5	283.0618005	Yes
4208	HMDB0132850	6,7-dihydroxy-3-(4-methoxyphenyl)-4H-chromen-4-on	C16H12O5	283.0618005	Yes
4208	HMDB0132873	7,8-dihydroxy-6-methoxy-3-phenyl-4H-chromen-4-one	C16H12O5	283.0618005	Yes
4208	HMDB0132886	5,7-dihydroxy-6-methoxy-3-phenyl-4H-chromen-4-one	C16H12O5	283.0618005	Yes
4208	HMDB0133330	7-hydroxy-2-(4-hydroxy-3-methoxyphenyl)-4H-chrome	C16H12O5	283.0618005	Yes
4208	HMDB0134889	5,6-dihydroxy-7-methoxy-2-phenyl-4H-chromen-4-one	C16H12O5	283.0618005	Yes
4208	HMDB0137557	6-hydroxy-2-[(4-hydroxy-3-methoxyphenyl)methylider	C16H12O5	283.0618005	Yes
4208	HMDB0140471	2-(3,5-dihydroxy-4-methoxyphenyl)-4H-chromen-4-on	C16H12O5	283.0618005	Yes
4208	HMDB0141945	7,8-dihydroxy-3-(4-methoxyphenyl)-4H-chromen-4-on	C16H12O5	283.0618005	Yes
4209	HMDB0000299; C01762; CHEBI:18107	Xanthosine	C10H12N4O6	283.0688092	Yes
4210	HMDB0011686; CHEBI:87986	p-Cresol glucuronide	C13H16O7	283.0824819	No
4210	HMDB0034618	2-O-Benzoyl-D-glucose	C13H16O7	283.0824819	No
4210	HMDB0038492	D-Vacciniin	C13H16O7	283.0824819	No
4212	HMDB0029819	2-Phenylethyl beta-D-glucopyranoside	C14H20O6	283.1180672	Yes
4213	HMDB0014564; C07379; CHEBI:8459	Promazine	C17H20N2S	283.1279273	Yes
4213	HMDB0015202; C07404; CHEBI:8461	Promethazine	C17H20N2S	283.1279273	Yes
4215	HMDB0060593	Alpha-dihydroartemisinin	C15H24O5	283.1556909	Yes
4215	HMDB0061088	Dihydroartemisinin (DHA)	C15H24O5	283.1556909	Yes
4220	HMDB0000827; C01530; CHEBI:28842	Stearic acid	C18H36O2	283.2643917	Yes
4220	HMDB0029811; CHEBI:84932	Ethyl hexadecanoate	C18H36O2	283.2643917	Yes
4220	HMDB0031066; CHEBI:1305874	16-Methylheptadecanoic acid	C18H36O2	283.2643917	Yes
4220	HMDB0061946	Hexyl dodecanoate	C18H36O2	283.2643917	Yes
4233	HMDB0006211; C04640; CHEBI:18413	2-(Formamido)-N1-(5-phospho-D-ribosyl)acetamidine	C6H12N3O8P	284.0277321	Yes
4234	HMDB0015166; C07372; CHEBI:177474	Probenecid	C13H19NO4S	284.0966901	Yes
4240	HMDB0013324	2-Octenoylcarnitine	C15H27NO4	284.1876253	No
4258	HMDB0132992	{[(4E)-5-(4-methoxyphenyl)-3-oxopent-4-en-2-yl]oxy}s	C12H14O6S	285.044585	No
4258	HMDB0132996	{[(4E)-5-(4-methoxyphenyl)-3-oxopent-4-en-1-yl]oxy}s	C12H14O6S	285.044585	No
4258	HMDB0140410	3-(3-methylbut-2-en-1-yl)-4-(sulfooxy)benzoic acid	C12H14O6S	285.044585	No
4259	HMDB0029498	3''-Methoxyfukiic acid	C12H14O8	285.0619746	Yes
4259	HMDB0041272	Uralenneoside	C12H14O8	285.0619746	Yes
4259	HMDB0059998; CHEBI:88743	Diphenol glucuronide	C12H14O8	285.0619746	Yes
4259	HMDB0124997	3,4,5-trihydroxy-6-(2-hydroxyphenoxy)oxane-2-carbox	C12H14O8	285.0619746	Yes
4259	HMDB0125471	3,4,5-trihydroxy-6-(3-hydroxyphenoxy)oxane-2-carbox	C12H14O8	285.0619746	Yes
4259	HMDB0135181	3,4,5-trihydroxy-6-(4-hydroxyphenoxy)oxane-2-carbox	C12H14O8	285.0619746	Yes
4261	HMDB0013895	3'',4''-Dihydrodiol	C15H14N2O4	285.0860352	No
4261	HMDB0060866	Phenytoin dihydrodiol	C15H14N2O4	285.0860352	No
4262	HMDB0006408; CHEBI:63799	4-Hydroxy tolbutamide	C12H18N2O4S	285.0914412	No
4262	HMDB0030840; CHEBI:745302	2-Methoxy-9-phenyl-1H-phenalen-1-one	C20H14O2	285.0914412	No
4262	HMDB0060438	Benzo[a]pyrene-7,8-diol	C20H14O2	285.0914412	No
4264	HMDB0030152	4''-Hydroxy-5,7-dimethoxyflavan	C17H18O4	285.1157853	No
4264	HMDB0030521; C10526	Sativan	C17H18O4	285.1157853	No
4264	HMDB0034024	Isosativan	C17H18O4	285.1157853	No
4264	HMDB0041168	Myrigalone G	C17H18O4	285.1157853	No
4264	HMDB0041169	Myrigalone H	C17H18O4	285.1157853	No
4264	HMDB0041671	4''-Hydroxy-3,4,5-trimethoxystilbene	C17H18O4	285.1157853	No
4264	HMDB0041680	4-Hydroxy-3,5,4''-trimethoxystilbene	C17H18O4	285.1157853	No
4264	HMDB0126611	3-phenyl-1-(2,4,6-trihydroxy-3,5-dimethylphenyl)propa	C17H18O4	285.1157853	No
4264	HMDB0130384	3-(3,4-dimethoxyphenyl)-3,4-dihydro-2H-1-benzopyra	C17H18O4	285.1157853	No
4264	HMDB0130572	6,8-dimethyl-2-phenyl-3,4-dihydro-2H-1-benzopyran-4	C17H18O4	285.1157853	No
4264	HMDB0132858	6-methoxy-3-(4-methoxyphenyl)-3,4-dihydro-2H-1-ber	C17H18O4	285.1157853	No
4264	HMDB0132908	1-(2-hydroxy-4,6-dimethoxyphenyl)-3-phenylpropan-1	C17H18O4	285.1157853	No
4264	HMDB0133974	1-(2-hydroxy-3,4-dimethoxyphenyl)-3-phenylpropan-1	C17H18O4	285.1157853	No
4269	HMDB0060995	10-alpha-methoxy-9,10-dihydrolysergol	C17H22N2O2	285.1614354	No
4270	HMDB0240222; CHEBI:104017	Thonzylamine	C16H22N4O	285.1711743	Yes
4272	HMDB0000672; C19615; CHEBI:73722	Hexadecanedioic acid	C16H30O4	285.2069682	No
4272	HMDB0041287	16-Hydroxy-10-oxohexadecanoic acid	C16H30O4	285.2069682	No
4272	HMDB0059777	2,2,4-Trimethyl-1,3-pentadienol diisobutyrate	C16H30O4	285.2069682	No
4292	HMDB0029914	Tetraphyllin B	C12H17NO7	286.0941124	No
4293	HMDB0002089; CHEBI:90055	N-Ribosylhistidine	C11H17N3O6	286.1050281	No

4295	HMDB0014812; C08526; CHEBI:5264	Galantamine	C17H21NO3	286.1450601	Yes
4295	HMDB0014887; C06991; CHEBI:4909	Etodolac	C17H21NO3	286.1450601	Yes
4295	HMDB0015005; C07239; CHEBI:147138	Ritodrine	C17H21NO3	286.1450601	Yes
4295	HMDB0033874	Piperanine	C17H21NO3	286.1450601	Yes
4295	HMDB0040699	Feruperine	C17H21NO3	286.1450601	Yes
4295	HMDB0041956	Nordihydrocodeine	C17H21NO3	286.1450601	Yes
4295	HMDB0060548; C11782	Dihydromorphine	C17H21NO3	286.1450601	Yes
4295	HMDB0060796	6alpha-Hydroxy-hydromorphone	C17H21NO3	286.1450601	Yes
4295	HMDB0060798	6beta-Hydroxy-hydromorphone	C17H21NO3	286.1450601	Yes
4295	HMDB0060854	Nordihydroisomorphine	C17H21NO3	286.1450601	Yes
4299	HMDB0000791; C02838; CHEBI:18102	L-Octanoylcarnitine	C15H29NO4	286.2027167	No
4301	HMDB0032358	Lauroyl diethanolamide	C16H33NO3	286.2391217	No
4324	HMDB0029191; CHEBI:134254	5''-(3'',4''-Dihydroxyphenyl)-gamma-valerolactone sulf	C11H12O7S	287.0237102	No
4324	HMDB0135678	{4-[(1Z)-2-hydroxy-3-oxobut-1-en-1-yl]-2-methoxypher	C11H12O7S	287.0237102	No
4324	HMDB0135688	[2-hydroxy-6-methoxy-4-(3-oxobut-1-en-1-yl)phenyl]o:	C11H12O7S	287.0237102	No
4324	HMDB0135693	[4-(3-acetyloxiran-2-yl)-2-methoxyphenyl]oxidanesulfo	C11H12O7S	287.0237102	No
4324	HMDB0135695	[4-(4-hydroxy-3-oxobut-1-en-1-yl)-2-methoxyphenyl]o:	C11H12O7S	287.0237102	No
4324	HMDB0135696	{[4-(4-hydroxy-3-methoxyphenyl)-2-oxobut-3-en-1-yl]c	C11H12O7S	287.0237102	No
4325	HMDB0000788; C01103; CHEBI:25722	Orotidine	C10H12N2O8	287.0525228	Yes
4329	HMDB0132970	{[1-(4-methoxyphenyl)-4-methylpentan-3-yl]oxy}sulfor	C13H20O5S	287.097721	Yes
4330	HMDB0034881	3-Carboxy-2,3,4,9-tetrahydro-1H-pyrido[3,4-b]indole-1	C15H16N2O4	287.1041808	No
4334	HMDB0034913; C11083	Imazamethabenz-methyl	C16H20N2O3	287.1402112	Yes
4334	HMDB0037554	(±)-Rollipyrrole	C16H20N2O3	287.1402112	Yes
4341	HMDB0037798; C08285	(S)-10,16-Dihydroxyhexadecanoic acid	C16H32O4	287.2228022	No
4341	HMDB0072841	MG(a-13:0/0:0/0:0)[rac]	C16H32O4	287.2228022	No
4341	HMDB0072843	MG(0:0/a-13:0/0:0)[rac]	C16H32O4	287.2228022	No
4341	HMDB0072845	MG(13:0/0:0/0:0)	C16H32O4	287.2228022	No
4341	HMDB0072861	MG(0:0/13:0/0:0)	C16H32O4	287.2228022	No
4341	HMDB0072870	MG(0:0/i-13:0/0:0)	C16H32O4	287.2228022	No
4355	HMDB0005765; CHEBI:84058	Ophthalmic acid	C11H19N3O6	288.1209498	Yes
4356	HMDB0061151	Hydroxylated N-acetyl desmethyl frovatriptan	C15H19N3O3	288.1353541	No
4357	HMDB0000552; CHEBI:70857	3-Methylglutarylcarnitine	C13H23NO6	288.1457541	No
4357	HMDB0061677; CHEBI:68568	O-Adipoylcarnitine	C13H23NO6	288.1457541	No
4360	HMDB0014783; C07881; CHEBI:4724	Dyclonine	C18H27NO2	288.1973612	No
4360	HMDB0031183	N-(2-Hydroxyisobutyl)-2,4,8,10,12-tetradecapentaenar	C18H27NO2	288.1973612	No
4376	HMDB0038459	5-(4-Acetoxy-3-oxo-1-butynyl)-2,2''-bithiophene	C14H10O3S2	289.0004666	Yes
4378	HMDB0001068; C00281; CHEBI:15721	D-Sedoheptulose 7-phosphate	C7H15O10P	289.0304466	Yes
4378	HMDB0060509; C06222; CHEBI:9082	Sedoheptulose 1-phosphate	C7H15O10P	289.0304466	Yes
4379	HMDB0059975; CHEBI:88682	4-Hydroxy-5-(3''-hydroxyphenyl)-valeric acid-3''-O-sulp	C11H14O7S	289.03889	Yes
4379	HMDB0059976; CHEBI:88683	4-Hydroxy-5-(4''-hydroxyphenyl)-valeric acid-4''-O-sulp	C11H14O7S	289.03889	Yes
4379	HMDB0125823	(5,7-dihydroxy-2,2-dimethyl-3,4-dihydro-2H-1-benzopy	C11H14O7S	289.03889	Yes
4379	HMDB0127756	5-(3-hydroxyphenyl)-4-(sulfooxy)pentanoic acid	C11H14O7S	289.03889	Yes
4379	HMDB0127760	5-[4-hydroxy-3-(sulfooxy)phenyl]pentanoic acid	C11H14O7S	289.03889	Yes
4379	HMDB0135701	[4-(1-hydroxy-3-oxobutyl)-2-methoxyphenyl]oxidanesu	C11H14O7S	289.03889	Yes
4379	HMDB0135702	[1-(4-hydroxy-3-methoxyphenyl)-3-oxobutoxy]sulfonic	C11H14O7S	289.03889	Yes
4379	HMDB0135708	[2-hydroxy-6-methoxy-4-(3-oxobutyl)phenyl]oxidanesu	C11H14O7S	289.03889	Yes
4379	HMDB0135711	[4-(4-hydroxy-3-oxobutyl)-2-methoxyphenyl]oxidanesu	C11H14O7S	289.03889	Yes
4379	HMDB0135712	[4-(4-hydroxy-3-methoxyphenyl)-2-oxobutoxy]sulfonic	C11H14O7S	289.03889	Yes
4379	HMDB0135714	[4-(2-hydroxy-3-oxobutyl)-2-methoxyphenyl]oxidanesu	C11H14O7S	289.03889	Yes
4379	HMDB0135715	{[1-(4-hydroxy-3-methoxyphenyl)-3-oxobutan-2-yl]oxy	C11H14O7S	289.03889	Yes
4380	HMDB0031779; C11111; CHEBI:6047	Isoprothiolane	C12H18O4S2	289.0564367	Yes
4381	HMDB0038666	L-N-(1H-Indol-3-ylacetyl)aspartic acid	C14H14N2O5	289.0827414	No
4381	HMDB0039500	N-Malonyltryptophan	C14H14N2O5	289.0827414	No
4382	HMDB0038722	5,6-Dihydro-11-methoxyyangonin	C16H18O5	289.1077869	Yes
4382	HMDB0127764	2-[2-hydroxy-3-(3-methoxyphenyl)propyl]benzene-1,3,	C16H18O5	289.1077869	Yes
4383	HMDB0014583; C01965; CHEBI:45924	Trimethoprim	C14H18N4O3	289.1308872	Yes
4383	HMDB0031767; C10896; CHEBI:3015	Benomyl	C14H18N4O3	289.1308872	Yes
4384	HMDB0032223; CHEBI:38708	Diisopentyl thiomalate	C14H26O4S	289.148677	No
4386	HMDB0032959	1-Octen-3-yl glucoside	C14H26O6	289.1661785	Yes
4402	HMDB0142153	(2S)-2-amino-3-[4-hydroxy-3-(sulfooxy)phenyl]-2-meth	C10H13NO7S	290.0344966	No
4406	HMDB0002354	Hawkinsin	C11H17NO6S	290.0703496	Yes

4406	HMDB0060609	Orciprenaline-3-O-sulfate	C11H17NO6S	290.0703496	Yes
4416	HMDB0015114; C06932; CHEBI:4024	Cyclopentolate	C17H25NO3	290.1757315	No
4416	HMDB0015341; C07914; CHEBI:6438	Levobunolol	C17H25NO3	290.1757315	No
4416	HMDB0036327	Norcapsaicin	C17H25NO3	290.1757315	No
4439	HMDB0124954	[2-hydroxy-5-(2-hydroxy-3-methoxy-3-oxopropyl)phen	C10H12O8S	291.0206624	Yes
4439	HMDB0124955	{[3-(3,4-dihydroxyphenyl)-1-methoxy-1-oxopropan-2-y	C10H12O8S	291.0206624	Yes
4439	HMDB0127788	2-hydroxy-3-[3-methoxy-4-(sulfooxy)phenyl]propanoic	C10H12O8S	291.0206624	Yes
4439	HMDB0127789	3-(4-hydroxy-3-methoxyphenyl)-2-(sulfooxy)propanoic	C10H12O8S	291.0206624	Yes
4439	HMDB0127843	2-hydroxy-3-[4-methoxy-3-(sulfooxy)phenyl]propanoic	C10H12O8S	291.0206624	Yes
4439	HMDB0127844	3-(3-hydroxy-4-methoxyphenyl)-2-(sulfooxy)propanoic	C10H12O8S	291.0206624	Yes
4439	HMDB0128005	3-[3-hydroxy-5-methoxy-4-(sulfooxy)phenyl]propanoic	C10H12O8S	291.0206624	Yes
4439	HMDB0133893	2-[2-hydroxy-4-methoxy-3-(sulfooxy)phenyl]propanoic	C10H12O8S	291.0206624	Yes
4441	HMDB0039404	3-Hydroxy-9-(4-hydroxyphenyl)-1H,3H-naphtho[1,8-cd	C18H12O4	291.0642625	No
4441	HMDB0039405	3-Hydroxy-4-(4-hydroxyphenyl)-1H,3H-naphtho[1,8-cd	C18H12O4	291.0642625	No
4442	HMDB0041269	Pantoyllactone glucoside	C12H20O8	291.1089679	Yes
4443	HMDB0028887	Histidinyl-Histidine	C12H16N6O3	291.1187387	No
4444	HMDB0030767; CHEBI:110179	11,12-Dimethoxydihydrokawain	C16H20O5	291.1250627	Yes
4444	HMDB0037774	Coriandrone A	C16H20O5	291.1250627	Yes
4444	HMDB0037775	Coriandrone B	C16H20O5	291.1250627	Yes
4444	HMDB0094645; CHEBI:132606	Mono-(2-ethyl-5-oxohexyl) phthalate	C16H20O5	291.1250627	Yes
4448	HMDB0032958	(S)-3-Octanol glucoside	C14H28O6	291.1814597	No
4460	HMDB0028994	Phenylalanyl-Glutamate	C14H17N2O5	292.1049729	No
4460	HMDB0029353	Galactosyl 4-hydroxyproline	C11H19NO8	292.1049729	No
4460	HMDB0029354	4-Hydroxyproline galactoside	C11H19NO8	292.1049729	No
4460	HMDB0034085	(3R)-3,4-Dihydroxy-3-(hydroxymethyl)butanenitrile 4-g	C11H19NO8	292.1049729	No
4460	HMDB0060493; C02713; CHEBI:21615	N-Acetylmuramate	C11H19NO8	292.1049729	No
4486	HMDB0135202	(2-hydroxy-1,2-diphenylethoxy)sulfonic acid	C14H14O5S	293.0492576	No
4488	HMDB0039825	Cyclocalopin F	C15H18O6	293.1035683	No
4489	HMDB0000594; HMDB0029562; CHEBI:8958	gamma-Glutamylphenylalanine	C14H18N2O5	293.1145683	Yes
4489	HMDB0001894; C11045; CHEBI:2877	Aspartame	C14H18N2O5	293.1145683	Yes
4489	HMDB0028875	Hydroxyprolyl-Tyrosine	C14H18N2O5	293.1145683	Yes
4489	HMDB0029106	Tyrosyl-Hydroxyproline	C14H18N2O5	293.1145683	Yes
4489	HMDB0029156; HMDB0028826	Glutamylphenylalanine	C14H18N2O5	293.1145683	Yes
4489	HMDB0031865	4''-tert-Butyl-2'',6''-dimethyl-3'',5''-dinitroacetophenor	C14H18N2O5	293.1145683	Yes
4489	HMDB0038514	L-gamma-Glutamyl-beta-phenyl-beta-L-alanine	C14H18N2O5	293.1145683	Yes
4492	HMDB0028919	Isoleucyl-Tyrosine	C15H22N2O4	293.1505881	No
4492	HMDB0028941	Leucyl-Tyrosine	C15H22N2O4	293.1505881	No
4492	HMDB0029108	Tyrosyl-Isoleucine	C15H22N2O4	293.1505881	No
4492	HMDB0029109	Tyrosyl-Leucine	C15H22N2O4	293.1505881	No
4492	HMDB0033464	Sinapoylputrescine	C15H22N2O4	293.1505881	No
4492	HMDB0139646	N-(4-aminobutyl)-3-(4-hydroxy-3,5-dimethoxyphenyl)p	C15H22N2O4	293.1505881	No
4493	HMDB0035443	(E,E)-1,6-bis(4-methoxyphenyl)-1,5-hexadiene	C20H22O2	293.1552085	No
4493	HMDB0038543	Longistylin A	C20H22O2	293.1552085	No
4495	HMDB0004668; C14765; CHEBI:902350	13-OxoODE	C18H30O3	293.2121629	No
4495	HMDB0004669; C14766	9-OxoODE	C18H30O3	293.2121629	No
4495	HMDB0010200	A-12(13)-EpODE	C18H30O3	293.2121629	No
4495	HMDB0010203; CHEBI:72641	13-HOTE	C18H30O3	293.2121629	No
4495	HMDB0010206	15(16)-EpODE	C18H30O3	293.2121629	No
4495	HMDB0010220	9(10)-EpODE	C18H30O3	293.2121629	No
4495	HMDB0010224; CHEBI:88438	9-HOTE	C18H30O3	293.2121629	No
4495	HMDB0011108; C16346	17-Hydroxylinolenic acid	C18H30O3	293.2121629	No
4495	HMDB0029786	10-Oxo-11-octadecen-13-olide	C18H30O3	293.2121629	No
4495	HMDB0029969	Squamostanal A	C18H30O3	293.2121629	No
4495	HMDB0030950	15,16-Epoxy-9,12-octadecadienoic acid	C18H30O3	293.2121629	No
4495	HMDB0030995; C19827; CHEBI:60956	(2''E,4''Z,8E)-Colneleic acid	C18H30O3	293.2121629	No
4495	HMDB0031088	12,13-Epoxy-9,15-octadecadienoic acid	C18H30O3	293.2121629	No
4495	HMDB0031103	2-Hydroxylinolenic acid	C18H30O3	293.2121629	No
4495	HMDB0031934; CHEBI:89388	(9S,10E,12Z,15Z)-9-Hydroxy-10,12,15-octadecatrienoi	C18H30O3	293.2121629	No
4495	HMDB0034586	(9Z,12Z,14E)-16-Hydroxy-9,12,14-octadecatrienoic aci	C18H30O3	293.2121629	No
4495	HMDB0036832	Sterebin D	C18H30O3	293.2121629	No
4495	HMDB0039603	3,4-Dimethyl-5-pentyl-2-furanheptanoic acid	C18H30O3	293.2121629	No

4495	HMDB0061644	3,4-Dimethyl-5-propyl-2-furannonanoic acid	C18H30O3	293.2121629	No
4495	HMDB0062288	12(13)-epoxy-6Z,9Z-octadecadienoic acid	C18H30O3	293.2121629	No
4495	HMDB0112093	5-Heptyl-2-furanheptanoic acid	C18H30O3	293.2121629	No
4495	HMDB0112099	5-Hexyl-2-furanoctanoic acid	C18H30O3	293.2121629	No
4495	HMDB0112102	5-pentyl-2-furannonanoic acid	C18H30O3	293.2121629	No
4495	HMDB0112103	5-Butyl-3-methyl-2-furannonanoic acid	C18H30O3	293.2121629	No
4514	HMDB0013854; CHEBI:116400	N4-Acetylsulfamethoxazole	C12H13N3O4S	294.0557075	No
4555	HMDB0031780; C18574	Dithianon	C14H4N2O2S2	294.9666336	No
4558	HMDB0130036	{[(3E)-4-(1-oxo-1H-isochromen-3-yl)but-3-en-2-yl]oxy}s	C13H12O6S	295.0284963	No
4558	HMDB0130043	{[(3E)-4-(1-oxo-1H-isochromen-3-yl)but-3-en-1-yl]oxy}s	C13H12O6S	295.0284963	No
4559	HMDB0041059	N-Methoxyspirobrassinol methyl ether	C13H16N2O2S2	295.058943	No
4562	HMDB0038120; C09674	Ovalicin	C16H24O5	295.1554241	Yes
4562	HMDB0039376	Methyl dihydrophaseate	C16H24O5	295.1554241	Yes
4562	HMDB0041551	3,8-Dihydroxy-6-methoxy-7(11)-eremophilen-12,8-olid	C16H24O5	295.1554241	Yes
4563	HMDB0001926; C07534; CHEBI:4903	17a-Ethynylestradiol	C20H24O2	295.1694194	Yes
4563	HMDB0015125; C08162; CHEBI:4953	Exemestane	C20H24O2	295.1694194	Yes
4563	HMDB0033293	5-Methoxy-1,7-diphenyl-3-heptanone	C20H24O2	295.1694194	Yes
4563	HMDB0061097	17-alpha-ethinyl estradiol	C20H24O2	295.1694194	Yes
4572	HMDB0013622; C00174; CHEBI:37527	Nonadeca-10(Z)-enoic acid	C19H36O2	295.2638443	No
4572	HMDB0035575	4,6-Nonadecanedione	C19H36O2	295.2638443	No
4594	HMDB0001563; C04545; CHEBI:19062	1-Methylguanosine	C11H15N5O5	296.0990768	No
4594	HMDB0005862; CHEBI:19702	2-Methylguanosine	C11H15N5O5	296.0990768	No
4594	HMDB0006038; CHEBI:70867	3''-O-Methylguanosine	C11H15N5O5	296.0990768	No
4594	HMDB0015401; CHEBI:63612	Nelarabine	C11H15N5O5	296.0990768	No
4618	HMDB0060834	N-Acetylserotonin sulfate	C12H14N2O5S	297.0554898	Yes
4619	HMDB0029512; CHEBI:1271609	4'',5-Dihydroxy-7-methoxy-6-methylflavone	C17H14O5	297.0745895	No
4619	HMDB0030718; C10527	Sayanedin	C17H14O5	297.0745895	No
4619	HMDB0038811	Alfalone	C17H14O5	297.0745895	No
4619	HMDB0128582	7-hydroxy-5,6-dimethoxy-2-phenyl-4H-chromen-4-one	C17H14O5	297.0745895	No
4619	HMDB0130361	3-(3-hydroxy-4-methoxyphenyl)-7-methoxy-4H-chrom	C17H14O5	297.0745895	No
4619	HMDB0130362	3-(3,4-dimethoxyphenyl)-7-hydroxy-4H-chromen-4-on	C17H14O5	297.0745895	No
4619	HMDB0130368	6-hydroxy-7-methoxy-3-(3-methoxyphenyl)-4H-chrom	C17H14O5	297.0745895	No
4619	HMDB0130392	8-hydroxy-7-methoxy-3-(3-methoxyphenyl)-4H-chrom	C17H14O5	297.0745895	No
4619	HMDB0130396	5-hydroxy-7-methoxy-3-(3-methoxyphenyl)-4H-chrom	C17H14O5	297.0745895	No
4619	HMDB0130763	6-hydroxy-5,7-dimethoxy-3-phenyl-4H-chromen-4-one	C17H14O5	297.0745895	No
4619	HMDB0130767	3-(4-hydroxyphenyl)-5,7-dimethoxy-4H-chromen-4-on	C17H14O5	297.0745895	No
4619	HMDB0130900	2-(2-hydroxyphenyl)-5,6-dimethoxy-4H-chromen-4-on	C17H14O5	297.0745895	No
4619	HMDB0132452	2-(4-hydroxyphenyl)-5,7-dimethoxy-4H-chromen-4-on	C17H14O5	297.0745895	No
4619	HMDB0132453	7-hydroxy-5-methoxy-2-(4-methoxyphenyl)-4H-chrom	C17H14O5	297.0745895	No
4619	HMDB0132454	5-hydroxy-7-methoxy-2-(4-methoxyphenyl)-4H-chrom	C17H14O5	297.0745895	No
4619	HMDB0132849	7-hydroxy-6-methoxy-3-(4-methoxyphenyl)-4H-chrom	C17H14O5	297.0745895	No
4619	HMDB0140449	2-(4-hydroxy-3,5-dimethoxyphenyl)-4H-chromen-4-on	C17H14O5	297.0745895	No
4619	HMDB0140450	2-(3-hydroxy-4,5-dimethoxyphenyl)-4H-chromen-4-on	C17H14O5	297.0745895	No
4619	HMDB0140455	2-(3,5-dimethoxyphenyl)-7-hydroxy-4H-chromen-4-on	C17H14O5	297.0745895	No
4621	HMDB0010350; C03033	2-Phenylethanol glucuronide	C14H18O7	297.0984896	Yes
4621	HMDB0124996	6-(3-ethylphenoxy)-3,4,5-trihydroxyoxane-2-carboxylic	C14H18O7	297.0984896	Yes
4621	HMDB0125173	6-(4-ethylphenoxy)-3,4,5-trihydroxyoxane-2-carboxylic	C14H18O7	297.0984896	Yes
4622	HMDB0004808; CHEBI:89489	7C-aglycone	C18H18O4	297.1138639	No
4622	HMDB0006101; CHEBI:562788	Enterolactone	C18H18O4	297.1138639	No
4622	HMDB0029543	(+)-Ligballinol	C18H18O4	297.1138639	No
4622	HMDB0030695	7-Hydroxy-5-methoxy-6,8-dimethylflavanone	C18H18O4	297.1138639	No
4622	HMDB0037376; CHEBI:70658	1-(2,4-Dihydroxy-6-methoxy-3,5-dimethylphenyl)-3-ph	C18H18O4	297.1138639	No
4622	HMDB0038453	5,7-Dimethoxy-6-methylflavanone	C18H18O4	297.1138639	No
4622	HMDB0131379	4-[5-(3-hydroxypropyl)-1-benzofuran-2-yl]-2-methoxyp	C18H18O4	297.1138639	No
4623	HMDB0036159	Toxin T2 tetrol	C15H22O6	297.1350897	Yes
4623	HMDB0037560	3,7,8,15-Scirpenetetrol	C15H22O6	297.1350897	Yes
4624	HMDB0015376; C07172; CHEBI:157833	Trimeprazine	C18H22N2S	297.1456897	Yes
4624	HMDB0035233	Olomoucine	C15H18N6O	297.1456897	Yes
4627	HMDB0014943; CHEBI:51038	Minaprine	C17H22N4O	297.1711773	Yes
4627	HMDB0015643; CHEBI:195280	Artemether	C16H26O5	297.1711773	Yes
4627	HMDB0061061	9''-desmethylgranisetron	C17H22N4O	297.1711773	Yes

4628	HMDB0060473; CHEBI:15949	Diphthamide	C13H24N5O3	297.1816362	Yes
4630	HMDB0010736; CHEBI:50576	3-Oxooctadecanoic acid	C18H34O3	297.2437323	No
4630	HMDB0030979	9-Oxooctadecanoic acid	C18H34O3	297.2437323	No
4630	HMDB0030980	10-Oxooctadecanoic acid	C18H34O3	297.2437323	No
4630	HMDB0030981	11-Oxooctadecanoic acid	C18H34O3	297.2437323	No
4630	HMDB0031127	5-Hexyltetrahydro-2-furanoctanoic acid	C18H34O3	297.2437323	No
4630	HMDB0034074	5-Oxooctadecanoic acid	C18H34O3	297.2437323	No
4630	HMDB0034297; C08365; CHEBI:28592	Ricinoleic acid	C18H34O3	297.2437323	No
4630	HMDB0061650; CHEBI:85661	9,10-epoxyoctadecanoic acid	C18H34O3	297.2437323	No
4631	HMDB0000772; C16535; CHEBI:39246	Nonadecanoic acid	C19H38O2	297.2798	No
4631	HMDB0000795; CHEBI:51340	Pristanic acid	C19H38O2	297.2798	No
4631	HMDB0004085; C16794	Tuberculostearic acid	C19H38O2	297.2798	No
4631	HMDB0034154	Methyl stearate	C19H38O2	297.2798	No
4631	HMDB0035474; CHEBI:31731	Isopropyl hexadecanoate	C19H38O2	297.2798	No
4631	HMDB0035674	4-Hydroxy-6-nonadecanone	C19H38O2	297.2798	No
4631	HMDB0035675	6-Hydroxy-4-nonadecanone	C19H38O2	297.2798	No
4631	HMDB0037397; CHEBI:133136	xi-17-Methyloctadecanoic acid	C19H38O2	297.2798	No
4631	HMDB0038070; CHEBI:84875	xi-16-Methyloctadecanoic acid	C19H38O2	297.2798	No
4654	HMDB0013300	Pentadecanoylglycine	C17H33NO3	298.2393685	No
4656	HMDB0000252; C00319; CHEBI:16393	Sphingosine	C18H37NO2	298.2756091	No
4656	HMDB0001480; C02934; CHEBI:17862	3-Dehydrosphinganine	C18H37NO2	298.2756091	No
4656	HMDB0002100; C16512; CHEBI:147261	Palmitoylethanolamide	C18H37NO2	298.2756091	No
4671	HMDB0128440	13,14,15-trihydroxy-9-oxo-8,17-dioxatetracyclo[8.7.0.(	C15H8O7	299.0175049	Yes
4671	HMDB0128443	5,13,14,15-tetrahydroxy-8,17-dioxatetracyclo[8.7.0.0²,	C15H8O7	299.0175049	Yes
4671	HMDB0128447	5,6,13,14-tetrahydroxy-8,17-dioxatetracyclo[8.7.0.0²,?	C15H8O7	299.0175049	Yes
4671	HMDB0128451	5,12,13,14-tetrahydroxy-8,17-dioxatetracyclo[8.7.0.0²,	C15H8O7	299.0175049	Yes
4671	HMDB0128455	3,5,13,14-tetrahydroxy-8,17-dioxatetracyclo[8.7.0.0²,?	C15H8O7	299.0175049	Yes
4672	HMDB0041849	Carboxytolbutamide	C12H16N2O5S	299.0714004	No
4674	HMDB0013938	Promazine 5-sulfoxide	C17H20N2OS	299.1212671	Yes
4709	HMDB0000781; C16265	N-Acetylgalactosamine 4-sulphate	C8H15NO9S	300.0396237	No
4709	HMDB0000814; C04132; CHEBI:28132	N-Acetylglucosamine 6-sulfate	C8H15NO9S	300.0396237	No
4709	HMDB0000841	N-Acetylgalactosamine 6-sulfate	C8H15NO9S	300.0396237	No
4745	HMDB0060031; CHEBI:89538	5-(3'',5''-Dihydroxyphenyl)-gamma-valerolactone-O-su	C12H14O7S	301.0400383	Yes
4745	HMDB0127769	{2-methoxy-4-[(5-oxooxolan-2-yl)methyl]phenyl}oxidar	C12H14O7S	301.0400383	Yes
4745	HMDB0127831	{2-methoxy-5-[(5-oxooxolan-2-yl)methyl]phenyl}oxidar	C12H14O7S	301.0400383	Yes
4747	HMDB0060017; CHEBI:89612	Pyrogallol-2-O-glucuronide	C12H14O9	301.0566906	Yes
4747	HMDB0125508	6-(2,4-dihydroxyphenoxy)-3,4,5-trihydroxyoxane-2-car	C12H14O9	301.0566906	Yes
4747	HMDB0125509	6-(2,5-dihydroxyphenoxy)-3,4,5-trihydroxyoxane-2-car	C12H14O9	301.0566906	Yes
4747	HMDB0125510	6-(3,4-dihydroxyphenoxy)-3,4,5-trihydroxyoxane-2-car	C12H14O9	301.0566906	Yes
4747	HMDB0125511	6-(2,6-dihydroxyphenoxy)-3,4,5-trihydroxyoxane-2-car	C12H14O9	301.0566906	Yes
4747	HMDB0125512	6-(2,3-dihydroxyphenoxy)-3,4,5-trihydroxyoxane-2-car	C12H14O9	301.0566906	Yes
4752	HMDB0028708	Arginyl-Glutamate	C11H20N5O5	301.1413108	Yes
4754	HMDB0028707	Arginyl-Glutamine	C11H22N6O4	301.1630409	Yes
4754	HMDB0028723	Arginyl-Gamma-glutamate	C11H22N6O4	301.1630409	Yes
4754	HMDB0028791	Glutaminylarginine	C11H22N6O4	301.1630409	Yes
4756	HMDB0015216; C07785; CHEBI:4779	Emedastine	C17H26N4O	301.2032158	Yes
4780	HMDB0013240; CHEBI:70811	Indoleacetyl glutamine	C15H17N3O4	302.114389	Yes
4782	HMDB0003573; C01851; CHEBI:16794	Scopolamine	C17H21NO4	302.1403068	No
4782	HMDB0015043; C01416; CHEBI:27958	Cocaine	C17H21NO4	302.1403068	No
4782	HMDB0015405; CHEBI:149226	Fenoterol	C17H21NO4	302.1403068	No
4782	HMDB0060543	6-Hydroxyetodolac	C17H21NO4	302.1403068	No
4782	HMDB0060544	7-Hydroxyetodolac	C17H21NO4	302.1403068	No
4782	HMDB0061074	alpha-noroxycodol	C17H21NO4	302.1403068	No
4782	HMDB0061075	beta-noroxycodol	C17H21NO4	302.1403068	No
4782	HMDB0061076	beta-oxymorphol	C17H21NO4	302.1403068	No
4798	HMDB0059985; CHEBI:88701	5-(3'',4'',5''-Trihydroxyphenyl)-gamma-valerolactone-3	C11H12O8S	303.0164851	Yes
4798	HMDB0059987; CHEBI:88693	5-(3'',4'',5''-Trihydroxyphenyl)-gamma-valerolactone-4	C11H12O8S	303.0164851	Yes
4798	HMDB0060020; CHEBI:89552	Sinapinic acid-O-sulphate	C11H12O8S	303.0164851	Yes
4798	HMDB0128711	{[3-(6-hydroxy-7-methoxy-2H-1,3-benzodioxol-5-yl)prc	C11H12O8S	303.0164851	Yes
4798	HMDB0128713	{[3-(7-hydroxy-6-methoxy-2H-1,3-benzodioxol-5-yl)prc	C11H12O8S	303.0164851	Yes
4801	HMDB0040631	Pratenol B	C15H12O7	303.0498166	Yes

4801	HMDB0125017	6,7-dihydroxy-3-(2,4,5-trihydroxyphenyl)-3,4-dihydro-2	C15H12O7	303.0498166	Yes
4801	HMDB0125208	2-(3,5-dihydroxyphenyl)-3,5,7-trihydroxy-3,4-dihydro-2	C15H12O7	303.0498166	Yes
4801	HMDB0125211	3-(3,5-dihydroxyphenyl)-1-(2,4,6-trihydroxyphenyl)pro	C15H12O7	303.0498166	Yes
4801	HMDB0125220	3,5,6,7-tetrahydroxy-2-(3-hydroxyphenyl)-3,4-dihydro-	C15H12O7	303.0498166	Yes
4801	HMDB0125366	3,5,6,7-tetrahydroxy-2-(4-hydroxyphenyl)-3,4-dihydro-	C15H12O7	303.0498166	Yes
4801	HMDB0125368	3-(4-hydroxyphenyl)-1-(2,3,4,6-tetrahydroxyphenyl)pro	C15H12O7	303.0498166	Yes
4801	HMDB0125371	2-(3,4-dihydroxyphenyl)-3,5,7-trihydroxy-3,4-dihydro-2	C15H12O7	303.0498166	Yes
4801	HMDB0125372	3-(3,4-dihydroxyphenyl)-1-(2,4,6-trihydroxyphenyl)pro	C15H12O7	303.0498166	Yes
4801	HMDB0126023	2-(3,4-dihydroxyphenyl)-5,6,7-trihydroxy-3,4-dihydro-2	C15H12O7	303.0498166	Yes
4801	HMDB0126025	(2E)-3-(3,4-dihydroxyphenyl)-1-(2,3,4,6-tetrahydroxypl	C15H12O7	303.0498166	Yes
4801	HMDB0126029	5,7-dihydroxy-2-(2,3,4-trihydroxyphenyl)-3,4-dihydro-2	C15H12O7	303.0498166	Yes
4801	HMDB0126030	(2E)-3-(2,3,4-trihydroxyphenyl)-1-(2,4,6-trihydroxyphe	C15H12O7	303.0498166	Yes
4801	HMDB0126033	5,7-dihydroxy-2-(3,4,5-trihydroxyphenyl)-3,4-dihydro-2	C15H12O7	303.0498166	Yes
4801	HMDB0126035	(2E)-1-(2,4,6-trihydroxyphenyl)-3-(3,4,5-trihydroxyphe	C15H12O7	303.0498166	Yes
4801	HMDB0126039	5,7-dihydroxy-2-(2,4,5-trihydroxyphenyl)-3,4-dihydro-2	C15H12O7	303.0498166	Yes
4801	HMDB0126040	(2E)-3-(2,4,5-trihydroxyphenyl)-1-(2,4,6-trihydroxyphe	C15H12O7	303.0498166	Yes
4801	HMDB0126565	3-(3-hydroxyphenyl)-1-(2,3,4,6-tetrahydroxyphenyl)pro	C15H12O7	303.0498166	Yes
4801	HMDB0128217	1-(2,4,6-trihydroxyphenyl)-3-(3,4,5-trihydroxyphenyl)p	C15H12O7	303.0498166	Yes
4801	HMDB0129344	3-(3,4-dihydroxyphenyl)-5,6,7-trihydroxy-3,4-dihydro-2	C15H12O7	303.0498166	Yes
4801	HMDB0129353	5,7-dihydroxy-3-(2,3,4-trihydroxyphenyl)-3,4-dihydro-2	C15H12O7	303.0498166	Yes
4801	HMDB0129359	5,7-dihydroxy-3-(2,4,5-trihydroxyphenyl)-3,4-dihydro-2	C15H12O7	303.0498166	Yes
4801	HMDB0130177	5,6,7,8-tetrahydroxy-2-(4-hydroxyphenyl)-3,4-dihydro-	C15H12O7	303.0498166	Yes
4801	HMDB0130180	3-(4-hydroxyphenyl)-1-(2,3,4,5,6-pentahydroxyphenyl)	C15H12O7	303.0498166	Yes
4801	HMDB0130332	3-(3,4-dihydroxyphenyl)-1-(2,3,4,6-tetrahydroxypheny	C15H12O7	303.0498166	Yes
4801	HMDB0131756	2-(2,4-dihydroxyphenyl)-3,5,7-trihydroxy-3,4-dihydro-2	C15H12O7	303.0498166	Yes
4801	HMDB0131926	2-(3,5-dihydroxyphenyl)-5,6,7-trihydroxy-3,4-dihydro-2	C15H12O7	303.0498166	Yes
4801	HMDB0133541	2,4,5,6-tetrahydroxy-2-[(4-hydroxyphenyl)methyl]-2,3-	C15H12O7	303.0498166	Yes
4801	HMDB0133542	2,4,6-trihydroxy-2-[hydroxy(4-hydroxyphenyl)methyl]-	C15H12O7	303.0498166	Yes
4801	HMDB0133543	2-[(3,4-dihydroxyphenyl)methyl]-2,4,6-trihydroxy-2,3-c	C15H12O7	303.0498166	Yes
4801	HMDB0137814	3-(2,4-dihydroxyphenyl)-5,7,8-trihydroxy-3,4-dihydro-2	C15H12O7	303.0498166	Yes
4802	HMDB0125817	(5-hydroxy-7-methoxy-2,2-dimethyl-3,4-dihydro-2H-1-	C12H16O7S	303.0543161	Yes
4802	HMDB0127785	5-(3-methoxyphenyl)-4-(sulfooxy)pentanoic acid	C12H16O7S	303.0543161	Yes
4802	HMDB0127792	5-[3-methoxy-4-(sulfooxy)phenyl]pentanoic acid	C12H16O7S	303.0543161	Yes
4804	HMDB0032943; C14324; CHEBI:34682	Diazinon	C12H21N2O3PS	303.0965262	No
4804	HMDB0034073; C08437	Vicine	C10H16N4O7	303.0965262	No
4804	HMDB0038665; CHEBI:136547	L-N-(1H-Indol-3-ylacetyl)glutamic acid	C15H16N2O5	303.0965262	No
4808	HMDB0006353; C00110	Dolichol phosphate	(C5H8)nC10H21O4I	303.1714564	No
4810	HMDB0000309	3a,16b-Dihydroxyandrostenone	C19H28O3	303.1947626	No
4810	HMDB0000322	16-Oxoandrostenediol	C19H28O3	303.1947626	No
4810	HMDB0000324	3a,16a-Dihydroxyandrostenone	C19H28O3	303.1947626	No
4810	HMDB0000352; C05139; CHEBI:27771	16a-Hydroxydehydroisoandrosterone	C19H28O3	303.1947626	No
4810	HMDB0000388	3a,16-Dihydroxyandrostenone	C19H28O3	303.1947626	No
4810	HMDB0003956; C05291	7a-Hydroxytestosterone	C19H28O3	303.1947626	No
4810	HMDB0004611; C18045; CHEBI:712194	7a-Hydroxydehydroepiandrosterone	C19H28O3	303.1947626	No
4810	HMDB0004624; CHEBI:712193	7b-Hydroxydehydroepiandrosterone	C19H28O3	303.1947626	No
4810	HMDB0006031; C14552	11-Ketoetiocholanolone	C19H28O3	303.1947626	No
4810	HMDB0006259; C14497; CHEBI:34477	6beta-Hydroxytestosterone	C19H28O3	303.1947626	No
4810	HMDB0006769; C05294	19-Hydroxytestosterone	C19H28O3	303.1947626	No
4810	HMDB0012533	11beta-Hydroxytestosterone	C19H28O3	303.1947626	No
4810	HMDB0012654	2beta-Hydroxytestosterone	C19H28O3	303.1947626	No
4810	HMDB0031464	cis-[8]-Shogaol	C19H28O3	303.1947626	No
4810	HMDB0039589	Ginsenoyne G	C19H28O3	303.1947626	No
4810	HMDB0039735	10-Acetoxy-8-heptadecene-4,6-diyn-3-ol	C19H28O3	303.1947626	No
4810	HMDB0060089; CHEBI:798	w Hydroxy testosterone	C19H28O3	303.1947626	No
4810	HMDB0060339; C18075; CHEBI:607670	11beta,17beta-Dihydroxy-4-androsten-3-one	C19H28O3	303.1947626	No
4812	HMDB0001043; C00219; CHEBI:15843	Arachidonic acid	C20H32O2	303.2328632	No
4812	HMDB0002177; CHEBI:71488	Cis-8,11,14,17-Eicosatetraenoic acid	C20H32O2	303.2328632	No
4812	HMDB0006036; D04947; CHEBI:529213	Mesterolone	C20H32O2	303.2328632	No
4812	HMDB0014996; C14605; CHEBI:34838	Drostanolone	C20H32O2	303.2328632	No
4812	HMDB0036702	Sideridiol	C20H32O2	303.2328632	No
4812	HMDB0036721	ent-17-Hydroxy-16beta-kauran-19-al	C20H32O2	303.2328632	No

4812	HMDB0036754	Yucalexin P21	C20H32O2	303.2328632	No
4812	HMDB0036829	Copalic acid	C20H32O2	303.2328632	No
4812	HMDB0036830	Junicedral	C20H32O2	303.2328632	No
4812	HMDB0037824	7,13-Eperudien-15-oic acid	C20H32O2	303.2328632	No
4812	HMDB0039687	Oryzalexin S	C20H32O2	303.2328632	No
4812	HMDB0039702; CHEBI:78259	Oryzalexin E	C20H32O2	303.2328632	No
4812	HMDB0060102	Arachidonate	C20H32O2	303.2328632	No
4827	HMDB0011691	Cytidine 2'',3''-cyclic phosphate	C9H12N3O7P	304.0341687	No
4827	HMDB0062545	2''-deoxycytidine 3''-monophosphate	C9H12N3O7P	304.0341687	No
4830	HMDB0034364	Cepharadione A	C18H11NO4	304.0591688	No
4831	HMDB0034364	Cepharadione A	C18H11NO4	304.0634121	No
4838	HMDB0002227; C06866; CHEBI:3374	Capsaicin	C18H27NO3	304.1921018	No
4855	HMDB0059978	4-Hydroxy-5-(dihydroxyphenyl)-valeric acid-O-sulphate	C11H14O8S	305.0340414	Yes
4855	HMDB0059979	4-Hydroxy-5-(dihydroxyphenyl)-valeric acid-O-sulphate	C11H14O8S	305.0340414	Yes
4855	HMDB0127746	4-hydroxy-5-[4-hydroxy-3-(sulfooxy)phenyl]pentanoic	C11H14O8S	305.0340414	Yes
4855	HMDB0127747	5-(3,4-dihydroxyphenyl)-4-(sulfooxy)pentanoic acid	C11H14O8S	305.0340414	Yes
4855	HMDB0128720	{[3-(4,5-dihydroxy-2,3-dimethoxyphenyl)prop-2-en-1-y	C11H14O8S	305.0340414	Yes
4862	HMDB0035781	Naematolone	C17H22O5	305.1397117	No
4862	HMDB0036487	Achillicin	C17H22O5	305.1397117	No
4862	HMDB0036643; C09499	Matricin	C17H22O5	305.1397117	No
4862	HMDB0134974	3,4-dihydroxy-2-(4-hydroxy-3,7-dimethylocta-2,6-dien-	C17H22O5	305.1397117	No
4862	HMDB0134975	3,4-dihydroxy-2-[(2Z)-4-hydroxy-3-(4-methylpent-3-en	C17H22O5	305.1397117	No
4862	HMDB0134976	3,4-dihydroxy-2-(8-hydroxy-3,7-dimethylocta-2,6-dien-	C17H22O5	305.1397117	No
4862	HMDB0134977	3,4-dihydroxy-2-[(6E)-8-hydroxy-3,7-dimethylocta-2,6-	C17H22O5	305.1397117	No
4862	HMDB0134979	3,4-dihydroxy-2-(5-hydroxy-3,7-dimethylocta-2,6-dien-	C17H22O5	305.1397117	No
4862	HMDB0134980	3,4-dihydroxy-2-{[3-methyl-3-(4-methylpent-3-en-1-yl)	C17H22O5	305.1397117	No
4862	HMDB0134982	2-(3,7-dimethylocta-2,6-dien-1-yl)-3,4,6-trihydroxyben	C17H22O5	305.1397117	No
4865	HMDB0031315	1-(beta-D-Glucopyranosyloxy)-3-octanone	C14H26O7	305.1609811	Yes
4865	HMDB0037107; C18325; CHEBI:75544	N1-trans-Feruloylagmatine	C15H22N4O3	305.1609811	Yes
4866	HMDB0012799	5''-Carboxy-gamma-chromanol	C18H26O4	305.1736542	No
4866	HMDB0034780; C20203	Capsiate	C18H26O4	305.1736542	No
4868	HMDB0000523	5-Androstene-3b,16b,17a-triol	C19H30O3	305.2124619	No
4868	HMDB0000540	5-Androstene-3b,16a,17a-triol	C19H30O3	305.2124619	No
4868	HMDB0000550	5-Androstenetriol	C19H30O3	305.2124619	No
4868	HMDB0002984; C14606	11-Hydroxyandrosterone	C19H30O3	305.2124619	No
4868	HMDB0013156	16-alpha-Hydroxyandrosterone	C19H30O3	305.2124619	No
4868	HMDB0013199	5-Androstene-3alpha-16b,17b-triol	C19H30O3	305.2124619	No
4868	HMDB0014759; C07346; CHEBI:7820	Oxandrolone	C19H30O3	305.2124619	No
4868	HMDB0040640	[8]-Paradol	C19H30O3	305.2124619	No
4869	HMDB0000523	5-Androstene-3b,16b,17a-triol	C19H30O3	305.2147157	No
4869	HMDB0000540	5-Androstene-3b,16a,17a-triol	C19H30O3	305.2147157	No
4869	HMDB0000550	5-Androstenetriol	C19H30O3	305.2147157	No
4869	HMDB0002984; C14606	11-Hydroxyandrosterone	C19H30O3	305.2147157	No
4869	HMDB0013156	16-alpha-Hydroxyandrosterone	C19H30O3	305.2147157	No
4869	HMDB0013199	5-Androstene-3alpha-16b,17b-triol	C19H30O3	305.2147157	No
4869	HMDB0014759; C07346; CHEBI:7820	Oxandrolone	C19H30O3	305.2147157	No
4869	HMDB0040640	[8]-Paradol	C19H30O3	305.2147157	No
4870	HMDB0002925; C03242; CHEBI:53486	8,11,14-Eicosatrienoic acid	C20H34O2	305.2484254	No
4870	HMDB0010378; CHEBI:1306412	5,8,11-Eicosatrienoic acid	C20H34O2	305.2484254	No
4870	HMDB0031058	Sciadonic acid	C20H34O2	305.2484254	No
4870	HMDB0036835	Sagittariol	C20H34O2	305.2484254	No
4870	HMDB0060039; C16522; CHEBI:53460	11,14,17-Eicosatrienoic acid	C20H34O2	305.2484254	No
4909	HMDB0001409; C00365; CHEBI:17622	dUMP	C9H13N2O8P	307.032954	Yes
4911	HMDB0033627	D-Erythroascorbic acid 1''-a-D-glucoside	C11H16O10	307.0679569	Yes
4912	HMDB0040692	4R,5R,6S-Trihydroxy-2-hydroxymethyl-2-cyclohexen-1-	C15H16O7	307.0829981	No
4912	HMDB0128179	4-[3-(3,4-dihydroxyphenyl)-2-hydroxypropyl]benzene-:	C15H16O7	307.0829981	No
4912	HMDB0128184	5-[2-hydroxy-3-(2,4,6-trihydroxyphenyl)propyl]benzen	C15H16O7	307.0829981	No
4912	HMDB0141543	5-[2-hydroxy-3-(2,4,6-trihydroxyphenyl)propyl]benzen	C15H16O7	307.0829981	No
4914	HMDB0037772	8-Acetylegelolide	C16H20O6	307.1186871	Yes
4914	HMDB0094647; CHEBI:132611	Mono-(2-ethyl-5-carboxypentyl) phthalate	C16H20O6	307.1186871	Yes
4916	HMDB0060990	8-Hydroxycarteolol	C16H24N2O4	307.1665972	Yes

4919	HMDB0005060; C16525; CHEBI:603631	Eicosadienoic acid	C20H36O2	307.2647777	No
4919	HMDB0036827; C09183; CHEBI:9053	Sclareol	C20H36O2	307.2647777	No
4919	HMDB0061864	Dihomo-linoleate (20:2n6)	C20H36O2	307.2647777	No
4919	HMDB0062432; CHEBI:78706	8Z,11Z-eicosadienoic acid	C20H36O2	307.2647777	No
4939	HMDB0015461; CHEBI:1178587	Glycodiazine	C13H15N3O4S	308.0715196	No
4940	HMDB0010319; C03033	Inodxyl glucuronide	C14H15NO7	308.0778533	No
4940	HMDB0040830	N-Feruloylaspartic acid	C14H15NO7	308.0778533	No
4940	HMDB0059804	Indoxyl glucuronide	C14H15NO7	308.0778533	No
4941	HMDB0033359	Cryptodorine	C18H15NO4	308.0930948	No
4941	HMDB0038584	Piperolactam C	C18H15NO4	308.0930948	No
4944	HMDB0028809	Glutaminyltyrosine	C14H19N3O5	308.1264898	Yes
4944	HMDB0029103	Tyrosyl-Glutamine	C14H19N3O5	308.1264898	Yes
4944	HMDB0029119	Tyrosyl-Gamma-glutamate	C14H19N3O5	308.1264898	Yes
4944	HMDB0030207	Koenigine	C19H19NO3	308.1264898	Yes
4947	HMDB0014484; CHEBI:51024	Metixene	C20H23NS	308.1485998	Yes
4949	HMDB0028963	Lysyl-Tyrosine	C15H23N3O4	308.1596899	Yes
4949	HMDB0029110	Tyrosyl-Lysine	C15H23N3O4	308.1596899	Yes
4977	HMDB0129951	{3-[2-(3,5-dihydroxyphenyl)ethyl]phenyl}oxidanesulfor	C14H14O6S	309.0438142	No
4984	HMDB0031060	(R)-2-Hydroxysterculic acid	C19H34O3	309.2438238	No
4985	HMDB0002231; C16526; CHEBI:32425	Eicosenoic acid	C20H38O2	309.279886	No
4985	HMDB0034296	11-Eicosenoic acid	C20H38O2	309.279886	No
4985	HMDB0034451; C03425	Ethyl oleate	C20H38O2	309.279886	No
4985	HMDB0035159; CHEBI:134479	Paullinic acid	C20H38O2	309.279886	No
4985	HMDB0035572; CHEBI:134474	6,8-Icosanedione	C20H38O2	309.279886	No
4985	HMDB0035573; CHEBI:134475	5,7-Icosanedione	C20H38O2	309.279886	No
4985	HMDB0035574	4,6-Icosanedione	C20H38O2	309.279886	No
4985	HMDB0037127	Oleyl acetate	C20H38O2	309.279886	No
4985	HMDB0062436; CHEBI:32419	9Z-eicosenoic acid	C20H38O2	309.279886	No
5001	HMDB0001961	1,7-Dimethylguanosine	C12H17N5O5	310.1155142	No
5001	HMDB0004824; CHEBI:19289	N2,N2-Dimethylguanosine	C12H17N5O5	310.1155142	No
5002	HMDB0028711	Arginyl-Histidine	C12H21N7O3	310.1641007	No
5002	HMDB0028879	Histidinyl-Arginine	C12H21N7O3	310.1641007	No
5031	HMDB0130051	{[(3E)-4-(5-hydroxy-1-oxo-1H-isochromen-3-yl)but-3-er	C13H12O7S	311.0228123	Yes
5031	HMDB0130058	{[(3E)-4-(5-hydroxy-1-oxo-1H-isochromen-3-yl)but-3-er	C13H12O7S	311.0228123	Yes
5031	HMDB0130093	{[2-hydroxy-1-(1-oxo-1H-isochromen-3-yl)but-3-en-1-y	C13H12O7S	311.0228123	Yes
5031	HMDB0130094	{[1-hydroxy-1-(1-oxo-1H-isochromen-3-yl)but-3-en-2-y	C13H12O7S	311.0228123	Yes
5031	HMDB0135800	({6-[(E)-2-methoxyethenyl]-2-oxo-2H-chromen-7-yl}me	C13H12O7S	311.0228123	Yes
5032	HMDB0013680; CHEBI:664875	Caftaric acid	C13H12O9	311.0396075	No
5032	HMDB0029276	Cis-Caffeoyl tartaric acid	C13H12O9	311.0396075	No
5034	HMDB0011677	Galactose-beta-1,4-xylose	C11H20O10	311.0982177	Yes
5034	HMDB0029883	Sambubiose	C11H20O10	311.0982177	Yes
5034	HMDB0029897	Serotinose	C11H20O10	311.0982177	Yes
5034	HMDB0038854; C07560	3-O-beta-D-Galactopyranosyl-L-arabinose	C11H20O10	311.0982177	Yes
5034	HMDB0038863	2-O-b-D-Galactopyranosyl-D-xylose	C11H20O10	311.0982177	Yes
5034	HMDB0038864	4-O-beta-D-Galactopyranosyl-D-xylose	C11H20O10	311.0982177	Yes
5034	HMDB0140855	6-({[3,4-dihydroxy-4-(hydroxymethyl)oxolan-2-yl]oxy}r	C11H20O10	311.0982177	Yes
5035	HMDB0028985	Methionyl-Tyrosine	C14H20N2O4S	311.1070177	Yes
5035	HMDB0029111	Tyrosyl-Methionine	C14H20N2O4S	311.1070177	Yes
5035	HMDB0060892	1-Naphtaldehyde	C22H16O2	311.1070177	Yes
5036	HMDB0004304; C06080	Nivalenol	C15H20O7	311.1144029	Yes
5036	HMDB0031724	Moringyne	C15H20O7	311.1144029	Yes
5036	HMDB0034861	3-Hydroxychavicol 1-glucoside	C15H20O7	311.1144029	Yes
5036	HMDB0135312	3,4,5-trihydroxy-6-(3-phenylpropoxy)oxane-2-carboxyl	C15H20O7	311.1144029	Yes
5040	HMDB0031976	Methyl (9Z)-10''-oxo-6,10''-diapo-6-carotenoate	C20H24O3	311.1632479	No
5040	HMDB0033294	5-Methoxy-7-(4-hydroxyphenyl)-1-phenyl-3-heptanone	C20H24O3	311.1632479	No
5040	HMDB0040761	4''-Hydroxy-5,5''-diisopropyl-2,2''-dimethyl-3,4-biphen	C20H24O3	311.1632479	No
5040	HMDB0061027	2-hydroxyethinylestradiol	C20H24O3	311.1632479	No
5041	HMDB0032549	N-Undecylbenzenesulfonic acid	C17H28O3S	311.168713	No
5043	HMDB0000995; C03207; CHEBI:18204	16-Dehydroprogesterone	C21H28O2	311.2016858	No
5043	HMDB0004626	Tetrahydrogestrinone	C21H28O2	311.2016858	No
5043	HMDB0014511; C08149; CHEBI:6443	Levonorgestrel	C21H28O2	311.2016858	No

5043	HMDB0014522; CHEBI:31527	Dydrogesterone	C21H28O2	311.2016858	No
5043	HMDB0060580; CHEBI:34749	Ethisterone	C21H28O2	311.2016858	No
5044	HMDB0003871; C04717; CHEBI:15655	13-L-Hydroperoxylinoleic acid	C18H32O4	311.2228034	No
5044	HMDB0004706; C14831; CHEBI:34485	8(R)-Hydroperoxylinoleic acid	C18H32O4	311.2228034	No
5044	HMDB0006940; C14827	9(S)-HPODE	C18H32O4	311.2228034	No
5044	HMDB0010201	12,13-DiHODE	C18H32O4	311.2228034	No
5044	HMDB0010208	15,16-DiHODE	C18H32O4	311.2228034	No
5044	HMDB0010221	9,10-DiHODE	C18H32O4	311.2228034	No
5044	HMDB0040900	(±)-(E)-13-Hydroxy-10-oxo-11-octadecenoic acid	C18H32O4	311.2228034	No
5044	HMDB0062281; C07338; CHEBI:15657	11-HpODE	C18H32O4	311.2228034	No
5044	HMDB0062434; C14827; CHEBI:34498	(10E,12Z)-(9S)-9-Hydroperoxyoctadeca-10,12-dienoic a	C18H32O4	311.2228034	No
5045	HMDB0000417	3D,7D,11D-Phytanic acid	C20H40O2	311.2956387	No
5045	HMDB0000553	3L,7D,11D-Phytanic acid	C20H40O2	311.2956387	No
5045	HMDB0000801; C01607; CHEBI:16285	Phytanic acid	C20H40O2	311.2956387	No
5045	HMDB0002212; C06425; CHEBI:28822	Arachidic acid	C20H40O2	311.2956387	No
5045	HMDB0031318	2,2,6,10,14-Pentamethylpentadecanoic acid	C20H40O2	311.2956387	No
5045	HMDB0034156; CHEBI:84936	Ethyl stearate	C20H40O2	311.2956387	No
5045	HMDB0035672	5-Hydroxy-7-eicosanone	C20H40O2	311.2956387	No
5045	HMDB0035673	4-Hydroxy-6-eicosanone	C20H40O2	311.2956387	No
5045	HMDB0062520	3,7R,11R,15-tetramethyl-hexadecanoic acid	C20H40O2	311.2956387	No
5062	HMDB0010328; C03033	Tyramine glucuronide	C14H19NO7	312.1091698	No
5093	HMDB0029508; CHEBI:90194	Laccaic acid D	C16H10O7	313.0325928	No
5093	HMDB0126141	12-hydroxy-5,7,9,20-tetraoxahexacyclo[11.7.0.0^{2},^{1}?.0^{3},	C16H10O7	313.0325928	No
5093	HMDB0126142	11,14-dihydroxy-6,8,19-trioxapentacyclo[10.7.0.0^{2},?.0	C16H10O7	313.0325928	No
5093	HMDB0128480	4,5,14-trihydroxy-13-methoxy-8,17-dioxatetracyclo[8.	C16H10O7	313.0325928	No
5093	HMDB0128481	5,14,15-trihydroxy-13-methoxy-8,17-dioxatetracyclo[8	C16H10O7	313.0325928	No
5093	HMDB0128482	5,6,14-trihydroxy-13-methoxy-8,17-dioxatetracyclo[8.	C16H10O7	313.0325928	No
5093	HMDB0128483	5,12,14-trihydroxy-13-methoxy-8,17-dioxatetracyclo[8	C16H10O7	313.0325928	No
5093	HMDB0128484	3,5,14-trihydroxy-13-methoxy-8,17-dioxatetracyclo[8.	C16H10O7	313.0325928	No
5094	HMDB0130068	[2-hydroxy-1-(1-oxo-1H-isochromen-3-yl)butoxy]sulfor	C13H14O7S	313.0393928	No
5094	HMDB0130069	{[1-hydroxy-1-(1-oxo-1H-isochromen-3-yl)butan-2-yl]o	C13H14O7S	313.0393928	No
5098	HMDB0015667; CHEBI:253330	Sulfaphenazole	C15H14N4O2S	313.0786913	No
5099	HMDB0029664	Vanilloside	C14H18O8	313.093113	No
5099	HMDB0033137	2-Methoxycarbonylphenyl beta-D-glucopyranoside	C14H18O8	313.093113	No
5099	HMDB0034060	6-O-Acetylarbutin	C14H18O8	313.093113	No
5099	HMDB0038333	2-Hydroxyphenylacetic acid O-b-D-glucoside	C14H18O8	313.093113	No
5099	HMDB0038335	(S)-Mandelic acid O-beta-D-Glucopyranoside	C14H18O8	313.093113	No
5099	HMDB0040720	2-O-Acetylarbutin	C14H18O8	313.093113	No
5099	HMDB0124984	6-(4-ethyl-2-hydroxyphenoxy)-3,4,5-trihydroxyoxane-2	C14H18O8	313.093113	No
5099	HMDB0124985	6-(5-ethyl-2-hydroxyphenoxy)-3,4,5-trihydroxyoxane-2	C14H18O8	313.093113	No
5099	HMDB0126441	6-(4-ethyl-3-hydroxyphenoxy)-3,4,5-trihydroxyoxane-2	C14H18O8	313.093113	No
5099	HMDB0126442	6-(2-ethyl-5-hydroxyphenoxy)-3,4,5-trihydroxyoxane-2	C14H18O8	313.093113	No
5100	HMDB0001930; D00673; CHEBI:8776	Ranitidine	C13H22N4O3S	313.1343427	No
5101	HMDB0029848	(-)-trans-Carveol glucoside	C16H26O6	313.1673871	Yes
5101	HMDB0029849	(+)-trans-Carveol glucoside	C16H26O6	313.1673871	Yes
5101	HMDB0033347; C17567	Lotusine	C19H24NO3	313.1673871	Yes
5101	HMDB0038706	Perilloside A	C16H26O6	313.1673871	Yes
5101	HMDB0040697	Oblongine	C19H24NO3	313.1673871	Yes
5102	HMDB0030263	Rhazidigenine Nb-oxide	C19H26N2O2	313.1925832	No
5103	HMDB0032995	2-Undecyl-4(1H)-quinolinone N-oxide	C20H28NO2	313.2073833	No
5105	HMDB0000782; CHEBI:133086	Octadecanedioic acid	C18H34O4	313.2387999	No
5105	HMDB0004704; C14828	9,10-DHOME	C18H34O4	313.2387999	No
5105	HMDB0004705; C14829	12,13-DHOME	C18H34O4	313.2387999	No
5105	HMDB0031679	(9xi,10xi,12xi)-9,10-Dihydroxy-12-octadecenoic acid	C18H34O4	313.2387999	No
5105	HMDB0041220	Dibutyl decanedioate	C18H34O4	313.2387999	No
5116	HMDB0015382	Mitiglinide	C19H25NO3	314.1764827	No
5116	HMDB0030185; CHEBI:713939	(E)-Piperolein A	C19H25NO3	314.1764827	No
5118	HMDB0094676	Val-Val-Val	C15H29N3O4	314.2082357	No
5138	HMDB0032013	Vanilloloside	C14H20O8	315.1088756	No
5138	HMDB0041024	Hydroxytyrosol 1-O-glucoside	C14H20O8	315.1088756	No
5140	HMDB0033643	10-Hydroxy-8-nor-2-fenchanone glucoside	C15H24O7	315.1461358	Yes

5140	HMDB0035136	Glucosyl (E)-2,6-Dimethyl-2,5-heptadienoate	C15H24O7	315.1461358	Yes
5141	HMDB0029566	Isopulegone caffeate	C19H24O4	315.1588496	No
5141	HMDB0134704	3-[8-hydroxy-2-methyl-2-(4-methylpent-3-en-1-yl)-2H-	C19H24O4	315.1588496	No
5143	HMDB0029346	Neryl glucoside	C16H28O6	315.1811922	Yes
5143	HMDB0029856	(S)-alpha-Terpinyl glucoside	C16H28O6	315.1811922	Yes
5143	HMDB0030418	D-Linalool 3-glucoside	C16H28O6	315.1811922	Yes
5143	HMDB0037147	5(6)-Butyl-1,4-dioxan-2-one	C16H28O6	315.1811922	Yes
5143	HMDB0040563	Perilloside C	C16H28O6	315.1811922	Yes
5143	HMDB0060004; CHEBI:89620	Menthol-glucoronide	C16H28O6	315.1811922	Yes
5147	HMDB0011532	MG(0:0/15:0/0:0)	C18H36O4	315.2541775	No
5147	HMDB0011563; CHEBI:710856	MG(15:0/0:0/0:0)	C18H36O4	315.2541775	No
5147	HMDB0031008	(+)-15,16-Dihydroxyoctadecanoic acid	C18H36O4	315.2541775	No
5147	HMDB0059633; C15988; CHEBI:49254	(9S,10S)-9,10-dihydroxyoctadecanoate	C18H36O4	315.2541775	No
5147	HMDB0072849	MG(0:0/i-15:0/0:0)	C18H36O4	315.2541775	No
5147	HMDB0072862	MG(0:0/a-15:0/0:0)[rac]	C18H36O4	315.2541775	No
5147	HMDB0072865	MG(a-15:0/0:0/0:0)[rac]	C18H36O4	315.2541775	No
5147	HMDB0072878	MG(i-15:0/0:0/0:0)	C18H36O4	315.2541775	No
5163	HMDB0037839	N-(1-Deoxy-1-fructosyl)histidine	C12H19N3O7	316.1125108	Yes
5164	HMDB0030341; C10166; CHEBI:564921	Piplartine	C17H19NO5	316.1197109	Yes
5164	HMDB0137022	3-(3,4-dihydroxyphenyl)-N-[2-(3,4-dihydroxyphenyl)etl	C17H19NO5	316.1197109	Yes
5170	HMDB0014869; C12508; CHEBI:146727	Nateglinide	C19H27NO3	316.1923209	No
5170	HMDB0015592; C11168; CHEBI:64028	Tetrabenazine	C19H27NO3	316.1923209	No
5189	HMDB0128688	{[3-(6,7-dimethoxy-2H-1,3-benzodioxol-5-yl)prop-2-en	C12H14O8S	317.0343549	No
5191	HMDB0004068; C05606	Melanin	C18H10N2O4	317.0574533	No
5192	HMDB0041451	Musanolone F	C20H14O4	317.0802533	No
5196	HMDB0006709; C00399; CHEBI:46372	Ubiquinone-2	C19H26O4	317.1742136	Yes
5196	HMDB0030064	Cohulupone	C19H26O4	317.1742136	Yes
5196	HMDB0031373	(1(10)E,4E,6a,9b)-9-(2-Methylpropanoyloxy)-1(10),4,1	C19H26O4	317.1742136	Yes
5196	HMDB0035500	Panaquinquecol 6	C19H26O4	317.1742136	Yes
5196	HMDB0039277	[8]-Dehydrogingerdione	C19H26O4	317.1742136	Yes
5196	HMDB0134951	3-[2-(3,7-dimethylocta-2,6-dien-1-yl)-3,4-dihydroxyphe	C19H26O4	317.1742136	Yes
5200	HMDB0001337; C00909; CHEBI:15651	Leukotriene A4	C20H30O3	317.212858	No
5200	HMDB0005081; CHEBI:72801	5-HEPE	C20H30O3	317.212858	No
5200	HMDB0006027; C14665	Oxymesterone	C20H30O3	317.212858	No
5200	HMDB0010202	12-HEPE	C20H30O3	317.212858	No
5200	HMDB0010205; CHEBI:137269	14,15-EpETE	C20H30O3	317.212858	No
5200	HMDB0010209	15-HEPE	C20H30O3	317.212858	No
5200	HMDB0010210; C04577; CHEBI:15559	15-KETE	C20H30O3	317.212858	No
5200	HMDB0010212; C13843; CHEBI:1263761	17,18-EpETE	C20H30O3	317.212858	No
5200	HMDB0010217; C14732; CHEBI:52449	5-KETE	C20H30O3	317.212858	No
5200	HMDB0012534; CHEBI:91264	11R-HEPE	C20H30O3	317.212858	No
5200	HMDB0012611	18R-HEPE	C20H30O3	317.212858	No
5200	HMDB0013633; C14807; CHEBI:34151	12-KETE	C20H30O3	317.212858	No
5200	HMDB0030101	Methyl [8]-Shogaol	C20H30O3	317.212858	No
5200	HMDB0035116	(ent-7alpha)-7-Hydroxy-8(14),15-pimaradien-19-oic ac	C20H30O3	317.212858	No
5200	HMDB0035311	Galanal A	C20H30O3	317.212858	No
5200	HMDB0036698; C09110; CHEBI:5920	Ineketone	C20H30O3	317.212858	No
5200	HMDB0036706	Steviol	C20H30O3	317.212858	No
5200	HMDB0036712	Yucalexin B20	C20H30O3	317.212858	No
5200	HMDB0036713	Yucalexin B22	C20H30O3	317.212858	No
5200	HMDB0036724	ent-17-Hydroxy-15-kauren-19-oic acid	C20H30O3	317.212858	No
5200	HMDB0036752	Yucalexin P17	C20H30O3	317.212858	No
5200	HMDB0036776	Yucalexin A19	C20H30O3	317.212858	No
5200	HMDB0036806	Aframodial	C20H30O3	317.212858	No
5200	HMDB0036839	(ent-15beta)-15-Hydroxy-19-trachylobanoic acid	C20H30O3	317.212858	No
5200	HMDB0036930	Grandiflorolic acid	C20H30O3	317.212858	No
5200	HMDB0037523; C17498	Galanolactone	C20H30O3	317.212858	No
5200	HMDB0038521	2-Hydroxy-6-(8-tridecenyl)benzoic acid	C20H30O3	317.212858	No
5200	HMDB0039012	(ent-15beta)-15,19-Dihydroxy-7-trachylobanone	C20H30O3	317.212858	No
5200	HMDB0039716	12alpha-12-Hydroxy-7,13-abietadien-18-oic acid	C20H30O3	317.212858	No
5200	HMDB0041058	Phytocassane C	C20H30O3	317.212858	No

5200	HMDB0060053	9-HEPE	C20H30O3	317.212858	No
5200	HMDB0061114	Atractyligenin	C20H30O3	317.212858	No
5200	HMDB0062222; C18177; CHEBI:81563	18R-hydroxy-5Z,8Z,11Z,14Z,16E-eicosapentaenoic acid	C20H30O3	317.212858	No
5200	HMDB0062293; CHEBI:91140	15R-hydroxy-5Z,8Z,11Z,13E,17Z-eicosapentaenoic acid	C20H30O3	317.212858	No
5200	HMDB0062296; CHEBI:88347	15S-hydroxy-5Z,8Z,11Z,13E,17Z-eicosapentaenoic acid	C20H30O3	317.212858	No
5200	HMDB0062408	5S-hydroxy-6E,8Z,11Z,14Z,17Z-eicosapentaenoic acid	C20H30O3	317.212858	No
5200	HMDB0137419	(4E)-1-(3,4-dihydroxyphenyl)tetradec-4-en-3-one	C20H30O3	317.212858	No
5202	HMDB0031461	Xylene	C24H30	317.2277007	No
5236	HMDB0135412	{2-hydroxy-5-[(2E)-3-phenylprop-2-enoyl]phenyl}oxida	C15H12O6S	319.0298584	No
5237	HMDB0062560; CHEBI:77843	thymidine 3''-monophosphate	C10H13N2O8P	319.0360584	No
5240	HMDB0029292	3-p-Coumaroyl-1,5-quinolactone	C16H16O7	319.0821471	No
5240	HMDB0029293	4-p-Coumaroyl-1,5-quinolactone	C16H16O7	319.0821471	No
5240	HMDB0041295	7-Hydroxy-6-methoxy-alpha-pyrufuran	C16H16O7	319.0821471	No
5240	HMDB0125015	5-(4,7-dihydroxy-6-methoxy-3,4-dihydro-2H-1-benzopy	C16H16O7	319.0821471	No
5240	HMDB0125016	1-(2,4-dihydroxy-5-methoxyphenyl)-2-(2,4,5-trihydroxy	C16H16O7	319.0821471	No
5240	HMDB0125018	3-(2,5-dihydroxy-4-methoxyphenyl)-3,4-dihydro-2H-1-	C16H16O7	319.0821471	No
5240	HMDB0125019	2-(2,5-dihydroxy-4-methoxyphenyl)-1-(2,4,5-trihydroxy	C16H16O7	319.0821471	No
5240	HMDB0128216	3-(3,4-dihydroxy-5-methoxyphenyl)-1-(2,4,6-trihydroxy	C16H16O7	319.0821471	No
5240	HMDB0129343	2-(3-hydroxy-4-methoxyphenyl)-1-(2,3,4,6-tetrahydrox	C16H16O7	319.0821471	No
5240	HMDB0129352	2-(2,3-dihydroxy-4-methoxyphenyl)-1-(2,4,6-trihydroxy	C16H16O7	319.0821471	No
5240	HMDB0129358	2-(2,5-dihydroxy-4-methoxyphenyl)-1-(2,4,6-trihydroxy	C16H16O7	319.0821471	No
5240	HMDB0130179	3-(4-methoxyphenyl)-1-(2,3,4,5,6-pentahydroxyphenyl	C16H16O7	319.0821471	No
5240	HMDB0130331	3-(4-hydroxy-3-methoxyphenyl)-1-(2,3,4,6-tetrahydrox	C16H16O7	319.0821471	No
5240	HMDB0133840	2-(2-hydroxy-4-methoxyphenyl)-1-(2,3,4,6-tetrahydrox	C16H16O7	319.0821471	No
5241	HMDB0041463	2,3-Dihydro-2,3-dihydroxy-4-(4-methoxyphenyl)-1H-pl	C20H16O4	319.0956685	No
5241	HMDB0126080	5-hydroxy-8,8-dimethyl-4-phenyl-2H,8H-pyrano[2,3-f](	C20H16O4	319.0956685	No
5243	HMDB0015159; CHEBI:168396	Mycophenolic acid	C17H20O6	319.119262	Yes
5243	HMDB0030742; CHEBI:475114	Helipyrone	C17H20O6	319.119262	Yes
5243	HMDB0037321	Oleacein	C17H20O6	319.119262	Yes
5243	HMDB0130121	5-hydroxy-8-(2-hydroxypropan-2-yl)-4-(1-hydroxyprop	C17H20O6	319.119262	Yes
5243	HMDB0130122	5-hydroxy-8-(2-hydroxypropan-2-yl)-4-(2-hydroxyprop	C17H20O6	319.119262	Yes
5243	HMDB0130123	8-(1,2-dihydroxypropan-2-yl)-5-hydroxy-4-propyl-2H,8	C17H20O6	319.119262	Yes
5243	HMDB0130124	5-hydroxy-8-(2-hydroxypropan-2-yl)-4-(3-hydroxyprop	C17H20O6	319.119262	Yes
5244	HMDB0000901	Valproic acid glucuronide	C14H24O8	319.1393926	Yes
5244	HMDB0010347; C03033	Octanoylglucuronide	C14H24O8	319.1393926	Yes
5245	HMDB0032698	Bis(2-methylpropanoyloxy)-9,10-epoxy-p-mentha-1,3,!	C18H24O5	319.1553187	Yes
5245	HMDB0041824; C14750	alpha-Zearalenol	C18H24O5	319.1553187	Yes
5245	HMDB0041838; C14751	beta-Zearalenol	C18H24O5	319.1553187	Yes
5245	HMDB0134978	2-[2-(3,7-dimethylocta-2,6-dien-1-yl)-3,4-dihydroxyphe	C18H24O5	319.1553187	Yes
5281	HMDB0061011	N-desmethylalmotriptan	C16H23N3O2S	320.1439502	No
5297	HMDB0128305	3,4-dihydroxy-5-(3,4,5-trihydroxybenzoyloxy)benzoic a	C14H10O9	321.0257334	No
5299	HMDB0136090	2-(3,4,5-trihydroxyphenyl)-3,4-dihydro-2H-1-benzopyr	C15H14O8	321.0625935	Yes
5300	HMDB0006834; C04079; CHEBI:18416	D-Pantothenoyl-L-cysteine	C12H22N2O6S	321.1125937	Yes
5300	HMDB0029530; CHEBI:603420	Glabrene	C20H18O4	321.1125937	Yes
5300	HMDB0034173; C10514	Phaseollin	C20H18O4	321.1125937	Yes
5300	HMDB0041167	(-)-Shinpterocarpin	C20H18O4	321.1125937	Yes
5300	HMDB0041399	2,3-Dihydro-4-(4-methoxyphenyl)-1H-phenalene-1,2,3-	C20H18O4	321.1125937	Yes
5300	HMDB0125839	3-(5-hydroxy-2,2-dimethyl-2H-chromen-6-yl)-1-(4-hydr	C20H18O4	321.1125937	Yes
5300	HMDB0131486	5,7-dihydroxy-8-(3-methylbut-2-en-1-yl)-4-phenyl-2H-(	C20H18O4	321.1125937	Yes
5302	HMDB0032702; C14752	Zeranol	C18H26O5	321.1724738	Yes
5316	HMDB0060619; CHEBI:73129	Tazarotenic acid	C19H17NO2S	322.0916262	Yes
5317	HMDB0015252; CHEBI:31654	Gliclazide	C15H21N3O3S	322.1234218	No
5336	HMDB0013880	S-6-Hydroxywarfarin	C19H16O5	323.0932487	Yes
5336	HMDB0013881	S-4''-Hydroxywarfarin	C19H16O5	323.0932487	Yes
5336	HMDB0013882	R-4''-Hydroxywarfarin	C19H16O5	323.0932487	Yes
5336	HMDB0013883	R-6-Hydroxywarfarin	C19H16O5	323.0932487	Yes
5336	HMDB0013884	R-10-Hydroxywarfarin	C19H16O5	323.0932487	Yes
5336	HMDB0013885	R-8-Hydroxywarfarin	C19H16O5	323.0932487	Yes
5336	HMDB0013886	R-7-Hydroxywarfarin	C19H16O5	323.0932487	Yes
5336	HMDB0041296	Moracin K	C19H16O5	323.0932487	Yes
5336	HMDB0041297	Moracin L	C19H16O5	323.0932487	Yes

5336	HMDB0140986	4-hydroxy-3-[1-(2-hydroxyphenyl)-3-oxobutyl]-2H-chro	C19H16O5	323.0932487	Yes
5336	HMDB0140987	4,6-dihydroxy-3-(3-oxo-1-phenylbutyl)-2H-chromen-2-	C19H16O5	323.0932487	Yes
5336	HMDB0140988	4,7-dihydroxy-3-(3-oxo-1-phenylbutyl)-2H-chromen-2-	C19H16O5	323.0932487	Yes
5336	HMDB0140989	4-hydroxy-3-[1-(4-hydroxyphenyl)-3-oxobutyl]-2H-chro	C19H16O5	323.0932487	Yes
5336	HMDB0140990	4,8-dihydroxy-3-(3-oxo-1-phenylbutyl)-2H-chromen-2-	C19H16O5	323.0932487	Yes
5336	HMDB0140991	4,5-dihydroxy-3-(3-oxo-1-phenylbutyl)-2H-chromen-2-	C19H16O5	323.0932487	Yes
5336	HMDB0140992	4-hydroxy-3-(2-hydroxy-3-oxo-1-phenylbutyl)-2H-chro	C19H16O5	323.0932487	Yes
5336	HMDB0140993	4-hydroxy-3-(4-hydroxy-3-oxo-1-phenylbutyl)-2H-chro	C19H16O5	323.0932487	Yes
5336	HMDB0140995	4-hydroxy-3-[1-(3-hydroxyphenyl)-3-oxobutyl]-2H-chro	C19H16O5	323.0932487	Yes
5336	HMDB0142561	2-hydroxy-3-[(1R)-2-hydroxy-3-oxo-1-phenylbutyl]-4H-	C19H16O5	323.0932487	Yes
5336	HMDB0142603	2,8-dihydroxy-3-(3-oxo-1-phenylbutyl)-4H-chromen-4-	C19H16O5	323.0932487	Yes
5337	HMDB0014558; C06806; CHEBI:28052	Acetohexamide	C15H20N2O4S	323.1072765	Yes
5340	HMDB0014611; C06526; CHEBI:15854	Quinine	C20H24N2O2	323.176513	No
5340	HMDB0015044; C06527; CHEBI:28593	Quinidine	C20H24N2O2	323.176513	No
5345	HMDB0031007; CHEBI:1011895	1-Acetoxy-2-hydroxy-16-heptadecyn-4-one	C19H32O4	323.2229139	No
5359	HMDB0002377	Urothion	C11H11N5O3S2	324.023211	No
5360	HMDB0059997; CHEBI:88744	Dihyroxy-1H-indole glucuronide I	C14H15NO8	324.0728541	Yes
5362	HMDB0015294; CHEBI:112465	Amdinocillin	C15H23N3O3S	324.1398403	No
5366	HMDB0005049; C13800	10-Nitrolinoleic acid	C18H31NO4	324.2185439	No
5366	HMDB0014443; CHEBI:51066	Dapiprazole	C19H27N5	324.2185439	No
5366	HMDB0014750; C06852; CHEBI:3127	Bisoprolol	C18H31NO4	324.2185439	No
5366	HMDB0033503	Small bacteriocin	C18H31NO4	324.2185439	No
5366	HMDB0041248	N-[[3-Hydroxy-2-(2-pentenyl)cyclopentyl]acetyl]isoleu	C18H31NO4	324.2185439	No
5367	HMDB0015170; C07750; CHEBI:9622	Tolterodine	C22H31NO	324.2315418	No
5384	HMDB0128974	{[4-(7-methoxy-2-oxo-2H-chromen-6-yl)but-3-en-2-yl]	C14H14O7S	325.0392111	Yes
5384	HMDB0132710	{[1-(7-hydroxy-2-oxo-2H-chromen-8-yl)-3-methylbut-3	C14H14O7S	325.0392111	Yes
5386	HMDB0039723	3-O-alpha-D-Glucopyranuronosyl-D-xylose	C11H18O11	325.0758937	Yes
5386	HMDB0040737	Aldobiouronic acid D3	C11H18O11	325.0758937	Yes
5386	HMDB0127654	6-[2-carboxy-2,2-bis(hydroxymethyl)ethoxy]-3,4,5-trih	C11H18O11	325.0758937	Yes
5386	HMDB0127655	3,4,5-trihydroxy-6-{[3-hydroxy-2,2-bis(hydroxymethyl)	C11H18O11	325.0758937	Yes
5388	HMDB0060610	Hydroxyhexamide	C15H22N2O4S	325.1235338	Yes
5389	HMDB0034755	1-Methoxy-3-(4-hydroxyphenyl)-2E-propenal 4''-gluco	C16H22O7	325.1292784	Yes
5389	HMDB0034863	4-(4-Hydroxyphenyl)-2-butanone glucoside	C16H22O7	325.1292784	Yes
5389	HMDB0038708	Citrusin C	C16H22O7	325.1292784	Yes
5389	HMDB0133673	3,4,5-trihydroxy-6-(2-methyl-3-phenylpropoxy)oxane-2	C16H22O7	325.1292784	Yes
5390	HMDB0038595	Alkaloid AQC2	C19H22N2O3	325.1557213	No
5392	HMDB0031031	2-Dodecylbenzenesulfonic acid	C18H30O3S	325.1856502	Yes
5392	HMDB0059915	4-Dodecylbenzenesulfonic Acid	C18H30O3S	325.1856502	Yes
5392	HMDB0061835; CHEBI:44748	Heptaethylene glycol	C14H30O8	325.1856502	Yes
5408	HMDB0010316; CHEBI:32636	Acetaminophen glucuronide	C14H17NO8	326.0885073	No
5408	HMDB0029344	Blepharin	C14H17NO8	326.0885073	No
5410	HMDB0062579; CHEBI:77016	(4Z,7Z,10Z,13Z,16Z,19Z)-docosahexaenoate	C22H31O2	326.2232965	No
5411	HMDB0062579; CHEBI:77016	(4Z,7Z,10Z,13Z,16Z,19Z)-docosahexaenoate	C22H31O2	326.2268965	No
5439	HMDB0033622	Cabbage identification factor 2	C15H12N4O3S	327.057901	No
5441	HMDB0029677	Zapotinin	C18H16O6	327.0856486	No
5441	HMDB0030596	Ugaxanthone	C18H16O6	327.0856486	No
5441	HMDB0030727; C09479	Betagarin	C18H16O6	327.0856486	No
5441	HMDB0030834	Americanin A	C18H16O6	327.0856486	No
5441	HMDB0033169	Garbogiol	C18H16O6	327.0856486	No
5441	HMDB0033318	Isoamericanin A	C18H16O6	327.0856486	No
5441	HMDB0034109; CHEBI:48201	Frenolicin B	C18H16O6	327.0856486	No
5441	HMDB0034186	Morusignin B	C18H16O6	327.0856486	No
5441	HMDB0037458	5-Hydroxy-4'',7,8-trimethoxyflavone	C18H16O6	327.0856486	No
5441	HMDB0039601	Americanin D	C18H16O6	327.0856486	No
5441	HMDB0040303	5-Hydroxy-4,4'',6-trimethoxyaurone	C18H16O6	327.0856486	No
5441	HMDB0040719	8-Hydroxy-4'',5,7-trimethoxyflavone	C18H16O6	327.0856486	No
5441	HMDB0040935	Methyl 2-(2-methoxy-4-hydroxyphenyl)-6-methoxy-3-b	C18H16O6	327.0856486	No
5441	HMDB0041544	7-Hydroxy-3,4'',8-trimethoxyflavone	C18H16O6	327.0856486	No
5441	HMDB0128575	7-hydroxy-5,6-dimethoxy-2-(4-methoxyphenyl)-4H-chr	C18H16O6	327.0856486	No
5441	HMDB0128576	6-hydroxy-5,7-dimethoxy-2-(4-methoxyphenyl)-4H-chr	C18H16O6	327.0856486	No
5441	HMDB0128577	5-hydroxy-6,7-dimethoxy-2-(4-methoxyphenyl)-4H-chr	C18H16O6	327.0856486	No

5441	HMDB0128578	2-(4-hydroxyphenyl)-5,6,7-trimethoxy-4H-chromen-4-c	C18H16O6	327.0856486	No
5441	HMDB0129574	5-hydroxy-3-(4-hydroxy-3-methoxyphenyl)-7-methoxy	C18H16O6	327.0856486	No
5441	HMDB0130360	3-(3,4-dimethoxyphenyl)-6-hydroxy-7-methoxy-4H-chr	C18H16O6	327.0856486	No
5441	HMDB0130363	3-(3,4-dimethoxyphenyl)-8-hydroxy-7-methoxy-4H-chr	C18H16O6	327.0856486	No
5441	HMDB0130364	3-(3,4-dimethoxyphenyl)-5-hydroxy-7-methoxy-4H-chr	C18H16O6	327.0856486	No
5441	HMDB0140447	7-hydroxy-2-(3,4,5-trimethoxyphenyl)-4H-chromen-4-c	C18H16O6	327.0856486	No
5441	HMDB0140448	6-hydroxy-2-(3,4,5-trimethoxyphenyl)-4H-chromen-4-c	C18H16O6	327.0856486	No
5441	HMDB0140451	5-hydroxy-2-(3,4,5-trimethoxyphenyl)-4H-chromen-4-c	C18H16O6	327.0856486	No
5445	HMDB0029777	Cymorcin monoglucoside	C16H24O7	327.1450388	No
5445	HMDB0032110	Litcubine	C19H22NO4	327.1450388	No
5445	HMDB0039385	3-Hydroxy-4-isopropylbenzyl alcohol 3-glucoside	C16H24O7	327.1450388	No
5445	HMDB0040465	Perilloside B	C16H24O7	327.1450388	No
5446	HMDB0014736; C07063; CHEBI:6343	Labetalol	C19H24N2O3	327.1711539	No
5450	HMDB0002183; C06429; CHEBI:28125	Docosahexaenoic acid	C22H32O2	327.233069	No
5450	HMDB0030053	Neogrifolin	C22H32O2	327.233069	No
5450	HMDB0030446; CHEBI:544843	Grifolin	C22H32O2	327.233069	No
5450	HMDB0035185; CHEBI:32095	Retinol acetate	C22H32O2	327.233069	No
5450	HMDB0038908	(Z,Z)-2-Methyl-5-(8,11,14-pentadecatrienyl)-1,3-benze	C22H32O2	327.233069	No
5452	HMDB0011534	MG(0:0/16:1(9Z)/0:0)	C19H36O4	327.2522646	No
5452	HMDB0011565	MG(16:1(9Z)/0:0/0:0)	C19H36O4	327.2522646	No
5452	HMDB0031043; CHEBI:967297	Avocadene 1-acetate	C19H36O4	327.2522646	No
5452	HMDB0031044	Avocadene 2-acetate	C19H36O4	327.2522646	No
5452	HMDB0031045	Avocadene 4-acetate	C19H36O4	327.2522646	No
5477	HMDB0000058; C00575; CHEBI:17489	Cyclic AMP	C10H12N5O6P	328.0452807	Yes
5477	HMDB0011616; C02353; CHEBI:27844	Adenosine 2'',3''-cyclic phosphate	C10H12N5O6P	328.0452807	Yes
5477	HMDB0031800; C18661; CHEBI:38718	Mecarbam	C10H20NO5PS2	328.0452807	Yes
5480	HMDB0032805	N-trans-Feruloyloctopamine	C18H19NO5	328.1179598	Yes
5480	HMDB0136405	N-[2-hydroxy-2-(4-hydroxyphenyl)ethyl]-3-(4-hydroxy-	C18H19NO5	328.1179598	Yes
5480	HMDB0137042	3-(4-hydroxy-3-methoxyphenyl)-N-[2-(4-hydroxypheny	C18H19NO5	328.1179598	Yes
5480	HMDB0137055	(2E)-N-[2-(3,4-dihydroxyphenyl)ethyl]-3-(4-hydroxy-3-r	C18H19NO5	328.1179598	Yes
5484	HMDB0013202	6-Keto-decanoylcarnitine	C17H31NO5	328.2118376	No
5504	HMDB0040621	3''-Glucosyl-2'',4'',6''-trihydroxyacetophenone	C14H18O9	329.0881569	Yes
5504	HMDB0128028	6-(5-ethyl-2,3-dihydroxyphenoxy)-3,4,5-trihydroxyoxa	C14H18O9	329.0881569	Yes
5504	HMDB0128029	6-(4-ethyl-2,6-dihydroxyphenoxy)-3,4,5-trihydroxyoxa	C14H18O9	329.0881569	Yes
5506	HMDB0005796	Picrocrocin	C16H26O7	329.1610638	Yes
5506	HMDB0031382	Portuloside A	C16H26O7	329.1610638	Yes
5506	HMDB0033111	Epijasminoside A	C16H26O7	329.1610638	Yes
5506	HMDB0033224	(1R,4R,5S)-5-Hydroxyfenchone glucoside	C16H26O7	329.1610638	Yes
5506	HMDB0033225	(1R,4S,6R)-6-Hydroxyfenchone glucoside	C16H26O7	329.1610638	Yes
5506	HMDB0033226	(1S,4R)-10-Hydroxyfenchone glucoside	C16H26O7	329.1610638	Yes
5506	HMDB0034558	6-Hydroxy-2-bornanone glucoside	C16H26O7	329.1610638	Yes
5506	HMDB0038979	(3b,4b,5b)-4,5-Epoxy-p-menth-1-en-3-ol 3-glucoside	C16H26O7	329.1610638	Yes
5506	HMDB0039056	(4S,6R)-p-Mentha-1,8-diene-6,7-diol 7-glucoside	C16H26O7	329.1610638	Yes
5507	HMDB0002121; C09069; CHEBI:468005	Carnosol	C20H26O4	329.1767038	Yes
5507	HMDB0036749	Momilactone B	C20H26O4	329.1767038	Yes
5507	HMDB0036753	Yucalexin P15	C20H26O4	329.1767038	Yes
5507	HMDB0036755	Yucalexin P8	C20H26O4	329.1767038	Yes
5507	HMDB0039613	3,3'',4,4''-Tetrahydroxy-5,5''-diisopropyl-2,2''-dimethyl	C20H26O4	329.1767038	Yes
5511	HMDB0004708; C14833	9,12,13-TriHOME	C18H34O5	329.232553	No
5511	HMDB0004710; C14835	9,10,13-TriHOME	C18H34O5	329.232553	No
5511	HMDB0030936	5,8,12-Trihydroxy-9-octadecenoic acid	C18H34O5	329.232553	No
5511	HMDB0038555	(9S,10E,12S,13S)-9,12,13-Trihydroxy-10-octadecenoic	C18H34O5	329.232553	No
5512	HMDB0011533; CHEBI:840954	MG(0:0/16:0/0:0)	C19H38O4	329.2698786	No
5512	HMDB0011564	MG(16:0/0:0/0:0)	C19H38O4	329.2698786	No
5512	HMDB0031074; CHEBI:718061	Glycerol 1-hexadecanoate	C19H38O4	329.2698786	No
5512	HMDB0072844	MG(i-16:0/0:0/0:0)	C19H38O4	329.2698786	No
5512	HMDB0072873	MG(0:0/i-16:0/0:0)	C19H38O4	329.2698786	No
5533	HMDB0000905; C00360; CHEBI:17713	Deoxyadenosine monophosphate	C10H14N5O6P	330.061926	Yes
5537	HMDB0014924; CHEBI:50662	Marimastat	C15H29N3O5	330.2032208	No
5561	HMDB0014831; CHEBI:31624	Fluorescein	C20H12O5	331.0616187	Yes
5562	HMDB0031721	Leonuriside A	C14H20O9	331.101722	Yes

5565	HMDB0028808	Glutaminyltryptophan	C16H20N4O4	331.1403244	Yes
5565	HMDB0029081	Tryptophyl-Glutamine	C16H20N4O4	331.1403244	Yes
5565	HMDB0029097	Tryptophyl-Gamma-glutamate	C16H20N4O4	331.1403244	Yes
5566	HMDB0030787	(R)-Marmin	C19H24O5	331.1550386	Yes
5566	HMDB0034987	Gibberellin A61	C19H24O5	331.1550386	Yes
5566	HMDB0035041; C11865; CHEBI:29599	Gibberellin A51	C19H24O5	331.1550386	Yes
5566	HMDB0035042	Gibberellin A45	C19H24O5	331.1550386	Yes
5566	HMDB0038014	Gibberellin A70	C19H24O5	331.1550386	Yes
5566	HMDB0134705	3-[8-hydroxy-2-(hydroxymethyl)-2-(4-methylpent-3-en	C19H24O5	331.1550386	Yes
5566	HMDB0134706	3-hydroxy-3-[8-hydroxy-2-methyl-2-(4-methylpent-3-e	C19H24O5	331.1550386	Yes
5566	HMDB0134707	3-[6,8-dihydroxy-2-methyl-2-(4-methylpent-3-en-1-yl)-	C19H24O5	331.1550386	Yes
5566	HMDB0134708	3-[7,8-dihydroxy-2-methyl-2-(4-methylpent-3-en-1-yl)-	C19H24O5	331.1550386	Yes
5566	HMDB0134709	3-{2-[2-(3,3-dimethyloxiran-2-yl)ethyl]-8-hydroxy-2-me	C19H24O5	331.1550386	Yes
5566	HMDB0134710	3-[8-hydroxy-5-methyl-5-(4-methylpent-3-en-1-yl)-3,6-	C19H24O5	331.1550386	Yes
5566	HMDB0134711	2-hydroxy-3-[8-hydroxy-2-methyl-2-(4-methylpent-3-e	C19H24O5	331.1550386	Yes
5566	HMDB0134712	3-[8-hydroxy-2-(2-hydroxy-4-methylpent-3-en-1-yl)-2-r	C19H24O5	331.1550386	Yes
5566	HMDB0134713	3-{8-hydroxy-2-[(3E)-5-hydroxy-4-methylpent-3-en-1-y	C19H24O5	331.1550386	Yes
5566	HMDB0134714	3-[8-hydroxy-2-(5-hydroxy-4-methylpent-3-en-1-yl)-2-r	C19H24O5	331.1550386	Yes
5566	HMDB0134715	3-[8-hydroxy-2-(1-hydroxy-4-methylpent-3-en-1-yl)-2-r	C19H24O5	331.1550386	Yes
5567	HMDB0028962	Lysyl-Tryptophan	C17H24N4O3	331.1767572	No
5567	HMDB0029088	Tryptophyl-Lysine	C17H24N4O3	331.1767572	No
5567	HMDB0029784	Unshuoside A	C16H28O7	331.1767572	No
5567	HMDB0033019	7-Hydroxyterpineol 8-glucoside	C16H28O7	331.1767572	No
5567	HMDB0033110	(1S,2S,4R)-1,8-Epoxy-p-menthan-2-ol glucoside	C16H28O7	331.1767572	No
5567	HMDB0033215	1,8-Epoxy-p-menthan-4-ol glucoside	C16H28O7	331.1767572	No
5567	HMDB0033220	(1R,2R,4S,6R)-2,6-Fenchanediol 2-O-b-D-glucoside	C16H28O7	331.1767572	No
5567	HMDB0033221	(1R,2S,4R,5S)-2,5-Fenchanediol 2-O-b-D-glucoside	C16H28O7	331.1767572	No
5567	HMDB0033644	(1S,2S,4S,5S)-2,4-Thujanediol 4-O-beta-D-Glucopyrano	C16H28O7	331.1767572	No
5567	HMDB0034772	(1R,3S,4S)-1,8-Epoxy-p-menthan-3-ol glucoside	C16H28O7	331.1767572	No
5567	HMDB0035025	6Z-8-Hydroxygeraniol 8-O-glucoside	C16H28O7	331.1767572	No
5567	HMDB0035634	Betulalbuside A	C16H28O7	331.1767572	No
5567	HMDB0038747	(2E,4E,7R)-2,7-Dimethyl-2,4-octadiene-1,8-diol 8-O-b-I	C16H28O7	331.1767572	No
5567	HMDB0038980	(4R,6S)-p-Menth-1-ene-4,6-diol 4-glucoside	C16H28O7	331.1767572	No
5567	HMDB0038981	(S)-p-Menth-1-ene-4,7-diol 4-glucoside	C16H28O7	331.1767572	No
5567	HMDB0039055	(1S,2R,4R)-p-Menth-8-ene-2,10-diol 2-glucoside	C16H28O7	331.1767572	No
5567	HMDB0060012; CHEBI:89610	Neomenthol-glucuronide	C16H28O7	331.1767572	No
5568	HMDB0030935	9,10,13-Trihydroxystearic acid	C18H36O5	331.2486752	No
5568	HMDB0034295; C19621	Floionolic acid	C18H36O5	331.2486752	No
5570	HMDB0002226; C16527; CHEBI:53487	Adrenic acid	C22H36O2	331.2642202	No
5570	HMDB0035676	1-Hydroxy-1-phenyl-3-hexadecanone	C22H36O2	331.2642202	No
5570	HMDB0035677	3-Hydroxy-1-phenyl-1-hexadecanone	C22H36O2	331.2642202	No
5570	HMDB0038910	2-Methyl-5-(8-pentadecenyl)-1,3-benzenediol	C22H36O2	331.2642202	No
5570	HMDB0062725; CHEBI:84873	Ethyl Arachidonate	C22H36O2	331.2642202	No
5610	HMDB0133344	[3-(7-hydroxy-4-oxo-4H-chromen-2-yl)phenyl]oxidanes	C15H10O7S	333.0079131	Yes
5610	HMDB0134560	[3-(3-hydroxy-4-oxo-4H-chromen-2-yl)phenyl]oxidanes	C15H10O7S	333.0079131	Yes
5610	HMDB0137537	{4-[(6-hydroxy-3-oxo-2,3-dihydro-1-benzofuran-2-ylide	C15H10O7S	333.0079131	Yes
5611	HMDB0060029; CHEBI:89551	5-(3'',4'',5''-Trihydroxyphenyl)-gamma-valerolactone-C	C12H14O9S	333.0290132	No
5611	HMDB0128699	[1-(6,7-dimethoxy-2H-1,3-benzodioxol-5-yl)-3-oxoprop	C12H14O9S	333.0290132	No
5611	HMDB0128707	{[1-(6,7-dimethoxy-2H-1,3-benzodioxol-5-yl)-3-oxopro	C12H14O9S	333.0290132	No
5611	HMDB0128717	{[3-(6,7-dimethoxy-2H-1,3-benzodioxol-5-yl)oxiran-2-y	C12H14O9S	333.0290132	No
5614	HMDB0014450; C06681; CHEBI:27504	Mitomycin	C15H18N4O5	333.1195962	Yes
5616	HMDB0037499	3''-N''-Acetylfusarochromanone	C17H22N2O5	333.1460534	Yes
5617	HMDB0040461	1,5-Dibutyl methyl hydroxycitrate	C15H26O8	333.1559083	Yes
5618	HMDB0032699	3-(2-Methylpropanoyloxy)-8-(2-methylbutanoyloxy)-9,	C19H26O5	333.1710935	No
5618	HMDB0032700	3-(2-Methylpropanoyloxy)-8-(3-methylbutanoyloxy)-9,	C19H26O5	333.1710935	No
5618	HMDB0134962	3-[3,4-dihydroxy-2-(4-hydroxy-3,7-dimethylocta-2,6-di	C19H26O5	333.1710935	No
5618	HMDB0134963	3-{3,4-dihydroxy-2-[(2Z)-4-hydroxy-3-(4-methylpent-3-	C19H26O5	333.1710935	No
5618	HMDB0134964	3-[3,4-dihydroxy-2-(8-hydroxy-3,7-dimethylocta-2,6-di	C19H26O5	333.1710935	No
5618	HMDB0134965	3-{3,4-dihydroxy-2-[(6E)-8-hydroxy-3,7-dimethylocta-2	C19H26O5	333.1710935	No
5618	HMDB0134966	3-[2-(3,7-dimethylocta-2,6-dien-1-yl)-3,4-dihydroxyphe	C19H26O5	333.1710935	No
5618	HMDB0134967	3-[3,4-dihydroxy-2-(5-hydroxy-3,7-dimethylocta-2,6-di	C19H26O5	333.1710935	No

5618	HMDB0134968	3-(3,4-dihydroxy-2-{[3-methyl-3-(4-methylpent-3-en-1-	C19H26O5	333.1710935	No
5618	HMDB0134969	3-{2-[5-(3,3-dimethyloxiran-2-yl)-3-methylpent-2-en-1-	C19H26O5	333.1710935	No
5618	HMDB0134970	3-[2-(3,7-dimethylocta-2,6-dien-1-yl)-3,4,6-trihydroxyp	C19H26O5	333.1710935	No
5618	HMDB0134971	3-[2-(3,7-dimethylocta-2,6-dien-1-yl)-3,4-dihydroxyphe	C19H26O5	333.1710935	No
5618	HMDB0134972	3-[2-(3,7-dimethylocta-2,6-dien-1-yl)-3,4,5-trihydroxyp	C19H26O5	333.1710935	No
5624	HMDB0000879; C13713; CHEBI:805752	Tetrahydrodeoxycorticosterone	C21H34O3	333.2426944	No
5624	HMDB0029583	Lycopersiconol	C21H34O3	333.2426944	No
5624	HMDB0035283	1-(3,5-Dihydroxyphenyl)-2-pentadecanone	C21H34O3	333.2426944	No
5624	HMDB0036302	ent-16beta-Methoxy-19-kauranoic acid	C21H34O3	333.2426944	No
5624	HMDB0038104	Isolinderenolide	C21H34O3	333.2426944	No
5624	HMDB0040642	[10]-Paradol	C21H34O3	333.2426944	No
5626	HMDB0002823; CHEBI:1038735	Docosatrienoic acid	C22H38O2	333.2808038	No
5626	HMDB0031099	(all-Z)-7,10,13-Docosatrienoic acid	C22H38O2	333.2808038	No
5647	HMDB0000489; C04540; CHEBI:17261	Aspartylglycosamine	C12H21N3O8	334.1284034	No
5648	HMDB0015054; CHEBI:520985	Almotriptan	C17H25N3O2S	334.1595459	No
5648	HMDB0015087; C07792; CHEBI:7478	Naratriptan	C17H25N3O2S	334.1595459	No
5678	HMDB0062424; CHEBI:58564	5-Phosphoribosyl-4-carboxy-5-aminoimidazole	C9H11N3O9P	335.0170779	Yes
5682	HMDB0029287	3-Caffeoyl-1,5-quinolactone	C16H16O8	335.0745781	No
5682	HMDB0029288	4-Caffeoyl-1,5-quinolactone	C16H16O8	335.0745781	No
5682	HMDB0030654	3-O-Caffeoylshikimic acid	C16H16O8	335.0745781	No
5682	HMDB0033563	Juglone glucoside	C16H16O8	335.0745781	No
5682	HMDB0033997	4-O-Caffeoylshikimic acid	C16H16O8	335.0745781	No
5682	HMDB0033999; C10434	Dattelic acid	C16H16O8	335.0745781	No
5682	HMDB0060562	Captopril-cysteine disulfide	C12H20N2O5S2	335.0745781	No
5682	HMDB0125035	5-(4,7-dihydroxy-6-methoxy-3,4-dihydro-2H-1-benzopy	C16H16O8	335.0745781	No
5682	HMDB0125036	1-(2,4-dihydroxy-5-methoxyphenyl)-2-(2,3,4,5-tetrahy	C16H16O8	335.0745781	No
5682	HMDB0125038	3-(2,3,5-trihydroxy-4-methoxyphenyl)-3,4-dihydro-2H-	C16H16O8	335.0745781	No
5682	HMDB0125039	2-(2,3,5-trihydroxy-4-methoxyphenyl)-1-(2,4,5-trihydr	C16H16O8	335.0745781	No
5682	HMDB0125050	4-(4,7-dihydroxy-6-methoxy-3,4-dihydro-2H-1-benzopy	C16H16O8	335.0745781	No
5682	HMDB0125051	1-(2,4-dihydroxy-5-methoxyphenyl)-2-(2,3,4,6-tetrahy	C16H16O8	335.0745781	No
5682	HMDB0125053	3-(2,3,6-trihydroxy-4-methoxyphenyl)-3,4-dihydro-2H-	C16H16O8	335.0745781	No
5682	HMDB0125054	2-(2,3,6-trihydroxy-4-methoxyphenyl)-1-(2,4,5-trihydr	C16H16O8	335.0745781	No
5682	HMDB0125065	6-methoxy-3-(2,4,5-trihydroxyphenyl)-3,4-dihydro-2H-	C16H16O8	335.0745781	No
5682	HMDB0125066	1-(2,3,4-trihydroxy-5-methoxyphenyl)-2-(2,4,5-trihydr	C16H16O8	335.0745781	No
5682	HMDB0125068	3-(2,5-dihydroxy-4-methoxyphenyl)-3,4-dihydro-2H-1-	C16H16O8	335.0745781	No
5682	HMDB0125069	2-(2,5-dihydroxy-4-methoxyphenyl)-1-(2,3,4,5-tetrahy	C16H16O8	335.0745781	No
5682	HMDB0125078	1-(2,4,6-trihydroxy-3-methoxyphenyl)-2-(2,4,5-trihydr	C16H16O8	335.0745781	No
5682	HMDB0125080	2-(2,5-dihydroxy-4-methoxyphenyl)-1-(2,3,4,6-tetrahy	C16H16O8	335.0745781	No
5682	HMDB0125083	7-hydroxy-6-methoxy-3-(1,2,5-trihydroxy-4-oxocyclohe	C16H16O8	335.0745781	No
5682	HMDB0126514	3,5,7-trihydroxy-2-(1-hydroxy-3-methoxy-4-oxocyclohe	C16H16O8	335.0745781	No
5685	HMDB0033107	Semilepidinoside A	C16H20N2O6	335.1256882	Yes
5691	HMDB0015324; C06803; CHEBI:2379	Acebutolol	C18H28N2O4	335.1977597	No
5693	HMDB0001085; C02165; CHEBI:15647	Leukotriene B4	C20H32O4	335.2229721	No
5693	HMDB0001193; C05356; CHEBI:15632	5(S)-Hydroperoxyeicosatetraenoic acid	C20H32O4	335.2229721	No
5693	HMDB0002236	8-iso-PGA1	C20H32O4	335.2229721	No
5693	HMDB0002656; C04685; CHEBI:15545	Prostaglandin A1	C20H32O4	335.2229721	No
5693	HMDB0002982; C00959; CHEBI:27624	Prostaglandin B1	C20H32O4	335.2229721	No
5693	HMDB0004243; C05965; CHEBI:15626	12(S)-HPETE	C20H32O4	335.2229721	No
5693	HMDB0004244; C05966; CHEBI:15628	15(S)-HPETE	C20H32O4	335.2229721	No
5693	HMDB0004688; C14808; CHEBI:15631	Hepoxilin A3	C20H32O4	335.2229721	No
5693	HMDB0004690; C14810; CHEBI:34784	Hepoxilin B3	C20H32O4	335.2229721	No
5693	HMDB0004692; C14812; CHEBI:34145	12(R)-HPETE	C20H32O4	335.2229721	No
5693	HMDB0004693; C14813	11H-14,15-EETA	C20H32O4	335.2229721	No
5693	HMDB0004696; C14820; CHEBI:34127	11(R)-HPETE	C20H32O4	335.2229721	No
5693	HMDB0004699; C14823; CHEBI:34487	8(S)-HPETE	C20H32O4	335.2229721	No
5693	HMDB0005050; C14781	15H-11,12-EETA	C20H32O4	335.2229721	No
5693	HMDB0005087; CHEBI:63981	6-trans-Leukotriene B4	C20H32O4	335.2229721	No
5693	HMDB0005088; CHEBI:63982	6-trans-12-epi-Leukotriene B4	C20H32O4	335.2229721	No
5693	HMDB0005089; C04853; CHEBI:1221367	12(S)-Leukotriene B4	C20H32O4	335.2229721	No
5693	HMDB0010204	14,15-DiHETE	C20H32O4	335.2229721	No
5693	HMDB0010211; CHEBI:1263764	17,18-DiHETE	C20H32O4	335.2229721	No

5693	HMDB0010216; CHEBI:91286	5,15-DiHETE	C20H32O4	335.2229721	No
5693	HMDB0010219	8,15-DiHETE	C20H32O4	335.2229721	No
5693	HMDB0011135; C05356	5-HPETE	C20H32O4	335.2229721	No
5693	HMDB0012498	10,11-dihydro-12-oxo-LTB4	C20H32O4	335.2229721	No
5693	HMDB0012839	6,7-dihydro-5-oxo-12-epi-LTB4	C20H32O4	335.2229721	No
5693	HMDB0034686	(14S)-14,15-Dihydroxy-8(17),13(16)-labdadien-19-oic a	C20H32O4	335.2229721	No
5693	HMDB0039171	9alpha-(3-Methylbutanoyloxy)-4S-hydroxy-10(14)-oplo	C20H32O4	335.2229721	No
5693	HMDB0039441	ent-1(10)-Halimene-15,19-dioic acid	C20H32O4	335.2229721	No
5693	HMDB0060103	9-Deoxy-delta12-PGD2	C20H32O4	335.2229721	No
5693	HMDB0060104; C04686; CHEBI:15546	Prostaglandin C1	C20H32O4	335.2229721	No
5693	HMDB0060105; CHEBI:90990	12,20-DiHETE	C20H32O4	335.2229721	No
5693	HMDB0062409	5,12-dihydroxy-6,8,10,14-eicosatetraenoic acid	C20H32O4	335.2229721	No
5693	HMDB0062479	10-hydroxy-11S,12S-epoxy-5Z,8Z,14Z-eicosatrienoic ac	C20H32O4	335.2229721	No
5693	HMDB0062614	5,20-DiHETE	C20H32O4	335.2229721	No
5693	HMDB0062615; CHEBI:91136	5(S),11(R)-DiHETE	C20H32O4	335.2229721	No
5693	HMDB0062619; C04849	(5Z,9E,14Z)-(8xi,11R,12S)-11,12-epoxy-8-hydroxyicosa	C20H32O4	335.2229721	No
5693	HMDB0062687; CHEBI:90982	8,20-DiHETE	C20H32O4	335.2229721	No
5693	HMDB0062688; CHEBI:91271	15-HPETE	C20H32O4	335.2229721	No
5693	HMDB0062749; CHEBI:91138	5(S),15(R)-DiHETE	C20H32O4	335.2229721	No
5713	HMDB0033374	Benzyl glycinate 4-methylbenzenesulfonate salt	C16H19NO5S	336.0919805	Yes
5715	HMDB0029332	5-Methoxynoracronycine	C20H19NO4	336.1221806	Yes
5715	HMDB0029827	Junosidine	C20H19NO4	336.1221806	Yes
5715	HMDB0033524	Lambertine	C20H19NO4	336.1221806	Yes
5734	HMDB0033444	15-Acetyl-4-deoxynivalenol	C17H22O7	337.129243	Yes
5734	HMDB0036137	Acetyldeoxynivalenol	C17H22O7	337.129243	Yes
5734	HMDB0039824	O-Acetylcyclocalopin A	C17H22O7	337.129243	Yes
5736	HMDB0014727; CHEBI:51164	Lisuride	C20H26N4O	337.2041706	No
5736	HMDB0038720	Dodecyl gallate	C19H30O5	337.2041706	No
5736	HMDB0040566	[6]-Gingerdiol 3-acetate	C19H30O5	337.2041706	No
5736	HMDB0040567	[6]-Gingerdiol 5-acetate	C19H30O5	337.2041706	No
5738	HMDB0002265; C14775; CHEBI:63966	14,15-DiHETrE	C20H34O4	337.2359332	No
5738	HMDB0002311; C14773; CHEBI:63970	8,9-DiHETrE	C20H34O4	337.2359332	No
5738	HMDB0002314; C14774; CHEBI:63969	11,12-DiHETrE	C20H34O4	337.2359332	No
5738	HMDB0002343; C14772; CHEBI:63974	5,6-DHET	C20H34O4	337.2359332	No
5738	HMDB0002995; C02165; CHEBI:15647	12-Keto-tetrahydro-leukotriene B4	C20H34O4	337.2359332	No
5738	HMDB0012504	10,11-dihydro-leukotriene B4	C20H34O4	337.2359332	No
5738	HMDB0012838	6,7-dihydro-12-epi-LTB4	C20H34O4	337.2359332	No
5738	HMDB0035379	Sterebin E	C20H34O4	337.2359332	No
5738	HMDB0060106	15-Hydroperoxyeicosa-8Z,11Z,13E-trienoate	C20H34O4	337.2359332	No
5741	HMDB0002068; C08316; CHEBI:28792	Erucic acid	C22H42O2	337.311112	No
5741	HMDB0002884; CHEBI:32428	Cetoleic acid	C22H42O2	337.311112	No
5741	HMDB0031013	Catelaidic acid	C22H42O2	337.311112	No
5741	HMDB0032470	Phytyl acetate	C22H42O2	337.311112	No
5741	HMDB0035566	7,9-Docosanedione	C22H42O2	337.311112	No
5741	HMDB0035567	6,8-Docosanedione	C22H42O2	337.311112	No
5741	HMDB0035568	5,7-Docosanedione	C22H42O2	337.311112	No
5741	HMDB0035569	4,6-Docosanedione	C22H42O2	337.311112	No
5741	HMDB0062659; CHEBI:82946	N-butyl Oleate	C22H42O2	337.311112	No
5762	HMDB0006273; C04751; CHEBI:28413	5-amino-1-(5-phospho-D-ribosyl)imidazole-4-carboxyla	C9H14N3O9P	338.0370551	No
5762	HMDB0012268	N5-Carboxyaminoimidazole ribonucleotide	C9H14N3O9P	338.0370551	No
5766	HMDB0005034; C07502; CHEBI:63631	Topiramate	C12H21NO8S	338.0914177	No
5766	HMDB0040829	N-(1-Deoxy-b-D-fructopyranosyl) (R)C(S)S-alliin	C12H21NO8S	338.0914177	No
5768	HMDB0014027	7-Hydroxygliclazide	C15H21N3O4S	338.1182477	No
5768	HMDB0014028	6-Hydroxygliclazide	C15H21N3O4S	338.1182477	No
5768	HMDB0014029	Methylhydroxygliclazide	C15H21N3O4S	338.1182477	No
5770	HMDB0015245; C06533; CHEBI:121716	Papaverine	C20H21NO4	338.1377553	No
5770	HMDB0036988	N-Methoxycarbonyl-N-nornuciferine	C20H21NO4	338.1377553	No
5770	HMDB0038629	Litebamine	C20H21NO4	338.1377553	No
5775	HMDB0000583	Docosanamide	C22H45NO	338.3447958	No
5784	HMDB0000968; C01220; CHEBI:17816	1D-Myo-inositol 1,4-bisphosphate	C6H14O12P2	338.9863173	No
5784	HMDB0001047; C00665; CHEBI:28602	D-Fructose 2,6-bisphosphate	C6H14O12P2	338.9863173	No

5784	HMDB0001058; C00354; CHEBI:16905	Fructose 1,6-bisphosphate	C6H14O12P2	338.9863173	No
5784	HMDB0003514; C01231; CHEBI:18148	Alpha-D-Glucose 1,6-bisphosphate	C6H14O12P2	338.9863173	No
5784	HMDB0006234; C04062; CHEBI:18225	1D-Myo-inositol 1,3-bisphosphate	C6H14O12P2	338.9863173	No
5784	HMDB0006235; C04063; CHEBI:28858	1D-Myo-inositol 3,4-bisphosphate	C6H14O12P2	338.9863173	No
5784	HMDB0006872; C03785; CHEBI:4250	D-Tagatose 1,6-bisphosphate	C6H14O12P2	338.9863173	No
5784	HMDB0060269	D-Mannose 1,6-bisphosphate	C6H14O12P2	338.9863173	No
5784	HMDB0060444; C05378; CHEBI:28013	beta-D-Fructose 1,6-bisphosphate	C6H14O12P2	338.9863173	No
5784	HMDB0062191; C04064; CHEBI:18156	1-phosphatidyl-1D-myo-inositol-3,4-bisphosphate	C6H14O12P2	338.9863173	No
5787	HMDB0001030; C05403	3''-Ketolactose	C12H20O11	339.0936076	Yes
5787	HMDB0029917	2-O-(4-O-Methyl-a-D-glucopyranuronosyl)-D-xylose	C12H20O11	339.0936076	Yes
5787	HMDB0039725	4-O-(4-O-Methyl-alpha-D-glucopyranuronosyl)-L-arabi	C12H20O11	339.0936076	Yes
5787	HMDB0039888	3-O-(4-O-Methyl-a-D-glucopyranuronosyl)-L-arabinose	C12H20O11	339.0936076	Yes
5787	HMDB0039889	4-O-beta-D-Glucopyranuronosyl-L-fucose	C12H20O11	339.0936076	Yes
5787	HMDB0040159	2-O-a-D-Galactopyranuronosyl-L-rhamnose	C12H20O11	339.0936076	Yes
5787	HMDB0124752	3,4,5-trihydroxy-6-[(2,3,5-trihydroxy-6-methyloxan-4-y	C12H20O11	339.0936076	Yes
5788	HMDB0030678	Linocinnamarin	C16H20O8	339.1089076	No
5788	HMDB0035441	Oenanthoside A	C16H20O8	339.1089076	No
5788	HMDB0060021; CHEBI:89553	trans-isoeugenol-O-glucuronide	C16H20O8	339.1089076	No
5788	HMDB0133636	6-(2-benzyl-3-oxopropoxy)-3,4,5-trihydroxyoxane-2-ca	C16H20O8	339.1089076	No
5788	HMDB0133639	3,4,5-trihydroxy-6-(2-methyl-3-oxo-1-phenylpropoxy)c	C16H20O8	339.1089076	No
5788	HMDB0133641	3,4,5-trihydroxy-6-[3-(2-methyl-3-oxopropyl)phenoxy]	C16H20O8	339.1089076	No
5788	HMDB0133643	3,4,5-trihydroxy-6-[4-(2-methyl-3-oxopropyl)phenoxy]	C16H20O8	339.1089076	No
5788	HMDB0133645	3,4,5-trihydroxy-6-[(2E)-3-hydroxy-2-(phenylmethylide	C16H20O8	339.1089076	No
5788	HMDB0133647	3,4,5-trihydroxy-6-{3-[(1E)-3-hydroxy-2-methylprop-1-	C16H20O8	339.1089076	No
5788	HMDB0133650	3,4,5-trihydroxy-6-{4-[(1E)-3-hydroxy-2-methylprop-1-	C16H20O8	339.1089076	No
5788	HMDB0133653	3,4,5-trihydroxy-6-[(2-methyl-3-phenyloxiran-2-yl)met	C16H20O8	339.1089076	No
5788	HMDB0133657	3,4,5-trihydroxy-6-{2-[(1E)-3-hydroxy-2-methylprop-1-	C16H20O8	339.1089076	No
5788	HMDB0133675	3,4,5-trihydroxy-6-[(2-methyl-3-phenylpropanoyl)oxy]	C16H20O8	339.1089076	No
5788	HMDB0133732	3,4,5-trihydroxy-6-{[(3E)-2-hydroxy-4-phenylbut-3-en-	C16H20O8	339.1089076	No
5788	HMDB0133733	3,4,5-trihydroxy-6-{[(3E)-1-hydroxy-4-phenylbut-3-en-	C16H20O8	339.1089076	No
5788	HMDB0133736	3,4,5-trihydroxy-6-{3-[(1E)-3-hydroxybut-1-en-1-yl]phe	C16H20O8	339.1089076	No
5788	HMDB0133739	3,4,5-trihydroxy-6-{4-[(1E)-3-hydroxybut-1-en-1-yl]phe	C16H20O8	339.1089076	No
5788	HMDB0133742	3,4,5-trihydroxy-6-[1-(3-phenyloxiran-2-yl)ethoxy]oxar	C16H20O8	339.1089076	No
5788	HMDB0133767	3,4,5-trihydroxy-6-(2-oxo-4-phenylbutoxy)oxane-2-car	C16H20O8	339.1089076	No
5788	HMDB0133769	3,4,5-trihydroxy-6-(3-oxo-1-phenylbutoxy)oxane-2-car	C16H20O8	339.1089076	No
5788	HMDB0133771	3,4,5-trihydroxy-6-[3-(3-oxobutyl)phenoxy]oxane-2-ca	C16H20O8	339.1089076	No
5788	HMDB0133773	3,4,5-trihydroxy-6-[4-(3-oxobutyl)phenoxy]oxane-2-ca	C16H20O8	339.1089076	No
5788	HMDB0135244	3,4,5-trihydroxy-6-[2-methoxy-4-(prop-2-en-1-yl)phen	C16H20O8	339.1089076	No
5788	HMDB0135652	3,4,5-trihydroxy-6-{[3-(4-methoxyphenyl)prop-2-en-1-	C16H20O8	339.1089076	No
5793	HMDB0000944; C08281; CHEBI:28941	Behenic acid	C22H44O2	339.3264081	No
5793	HMDB0035651	6-Hydroxy-8-docosanone	C22H44O2	339.3264081	No
5793	HMDB0035655	8-Hydroxy-6-docosanone	C22H44O2	339.3264081	No
5793	HMDB0035660	5-Hydroxy-7-docosanone	C22H44O2	339.3264081	No
5793	HMDB0035667	4-Hydroxy-6-docosanone	C22H44O2	339.3264081	No
5793	HMDB0040290; CHEBI:85983	Butyl octadecanoate	C22H44O2	339.3264081	No
5810	HMDB0014535; CHEBI:32168	Sulpiride	C15H23N3O4S	340.1338025	Yes
5810	HMDB0015135; C12766; CHEBI:31444	Cyclacillin	C15H23N3O4S	340.1338025	Yes
5831	HMDB0000048; C05402; CHEBI:28053	Melibiose	C12H22O11	341.1090999	Yes
5831	HMDB0000055; C06422; CHEBI:36217	Cellobiose	C12H22O11	341.1090999	Yes
5831	HMDB0000163; C00208; CHEBI:47937	D-Maltose	C12H22O11	341.1090999	Yes
5831	HMDB0000186; C00243; CHEBI:36219	Alpha-Lactose	C12H22O11	341.1090999	Yes
5831	HMDB0000258; C00089; CHEBI:17992	Sucrose	C12H22O11	341.1090999	Yes
5831	HMDB0000740; C07064; CHEBI:6359	Lactulose	C12H22O11	341.1090999	Yes
5831	HMDB0000975; C01083; CHEBI:16551	Trehalose	C12H22O11	341.1090999	Yes
5831	HMDB0002923; C00252; CHEBI:28189	Isomaltose	C12H22O11	341.1090999	Yes
5831	HMDB0005826; C01235; CHEBI:17505	Galactinol	C12H22O11	341.1090999	Yes
5831	HMDB0006603	3-b-Galactopyranosyl glucose	C12H22O11	341.1090999	Yes
5831	HMDB0006792; C05400; CHEBI:4808	Epimelibiose	C12H22O11	341.1090999	Yes
5831	HMDB0011740; C19636; CHEBI:32528	Turanose	C12H22O11	341.1090999	Yes
5831	HMDB0011742; C19632; CHEBI:33020	Kojibiose	C12H22O11	341.1090999	Yes
5831	HMDB0029880	Neotrehalose	C12H22O11	341.1090999	Yes

5831	HMDB0029882; C01518; CHEBI:7570	Sakebiose	C12H22O11	341.1090999	Yes
5831	HMDB0029898; C03323; CHEBI:16751	Inulobiose	C12H22O11	341.1090999	Yes
5831	HMDB0029902	Galabiose	C12H22O11	341.1090999	Yes
5831	HMDB0029919	Maltulose	C12H22O11	341.1090999	Yes
5831	HMDB0029933; CHEBI:28085	Mannobiose	C12H22O11	341.1090999	Yes
5831	HMDB0033368	DEAE-cellulose	C12H22O11	341.1090999	Yes
5831	HMDB0037138; C00897; CHEBI:18167	Maltodextrin	(C12H22O11)n	341.1090999	Yes
5831	HMDB0038489	Allolactose	C12H22O11	341.1090999	Yes
5831	HMDB0039237	Glucinol	C12H22O11	341.1090999	Yes
5831	HMDB0039727	Trehalulose	C12H22O11	341.1090999	Yes
5831	HMDB0039876	Fagopyritol A1	C12H22O11	341.1090999	Yes
5831	HMDB0041627; C01970; CHEBI:36218	beta-Lactose	C12H22O11	341.1090999	Yes
5831	HMDB0060068; C04147; CHEBI:27554	Glucose-1,3-mannose oligosaccharide	C12H22O11	341.1090999	Yes
5831	HMDB0125600	2-{[3,4-dihydroxy-2,5-bis(hydroxymethyl)oxolan-2-yl]o	C12H22O11	341.1090999	Yes
5831	HMDB0133305	6-({[3,4,5-trihydroxy-6-(hydroxymethyl)oxan-2-yl]oxy}r	C12H22O11	341.1090999	Yes
5832	HMDB0002277	2,3-Dinor-6-keto-prostaglandin F1 a	C18H30O6	341.1967265	Yes
5832	HMDB0002904	2,3-Dinor-TXB2	C18H30O6	341.1967265	Yes
5832	HMDB0038940	(-)-11-Hydroxy-9,15,16-trioxooctadecanoic acid	C18H30O6	341.1967265	Yes
5832	HMDB0061154	Monic acid	C18H30O6	341.1967265	Yes
5832	HMDB0062618; CHEBI:73944	2,3-dinor-6-oxoprostaglandin F1alpha	C18H30O6	341.1967265	Yes
5834	HMDB0002726	Guggulsterone	C22H30O3	341.2135325	No
5834	HMDB0031059; CHEBI:558148	2-Hydroxy-6-(8,11,14-pentadecatrienyl)benzoic acid	C22H30O3	341.2135325	No
5834	HMDB0060710	17-Hydroxymethylethisterone	C22H30O3	341.2135325	No
5882	HMDB0030477	Aflatoxin GM1	C17H12O8	343.0458547	Yes
5882	HMDB0037754	Wharangin	C17H12O8	343.0458547	Yes
5882	HMDB0142065	5-hydroxy-11-methoxy-6,8,19-trioxapentacyclo[10.7.0	C17H12O8	343.0458547	Yes
5883	HMDB0039287	Theogallin	C14H16O10	343.0694566	No
5883	HMDB0125148	6-[4-(carboxymethyl)-2-hydroxyphenoxy]-3,4,5-trihydr	C14H16O10	343.0694566	No
5883	HMDB0125149	6-[5-(carboxymethyl)-2-hydroxyphenoxy]-3,4,5-trihydr	C14H16O10	343.0694566	No
5883	HMDB0125150	6-{[2-(3,4-dihydroxyphenyl)acetyl]oxy}-3,4,5-trihydrox	C14H16O10	343.0694566	No
5883	HMDB0127989	6-(4-carboxy-2-methoxyphenoxy)-3,4,5-trihydroxyoxar	C14H16O10	343.0694566	No
5883	HMDB0127990	3,4,5-trihydroxy-6-(4-hydroxy-3-methoxybenzoyloxy)o	C14H16O10	343.0694566	No
5883	HMDB0128033	6-(3-carboxy-5-methoxyphenoxy)-3,4,5-trihydroxyoxar	C14H16O10	343.0694566	No
5883	HMDB0128034	3,4,5-trihydroxy-6-(3-hydroxy-5-methoxybenzoyloxy)o	C14H16O10	343.0694566	No
5883	HMDB0130459	6-[3-(carboxymethyl)-5-hydroxyphenoxy]-3,4,5-trihydr	C14H16O10	343.0694566	No
5883	HMDB0130460	6-{[2-(3,5-dihydroxyphenyl)acetyl]oxy}-3,4,5-trihydrox	C14H16O10	343.0694566	No
5883	HMDB0140927	6-(5-carboxy-2-methoxyphenoxy)-3,4,5-trihydroxyoxar	C14H16O10	343.0694566	No
5885	HMDB0029647	Domesticoside	C15H20O9	343.1035369	No
5885	HMDB0034862	4'',6''-Dihydroxy-2''-methoxyacetophenone 6''-glucosic	C15H20O9	343.1035369	No
5885	HMDB0128017	6-(5-ethyl-2-hydroxy-3-methoxyphenoxy)-3,4,5-trihydr	C15H20O9	343.1035369	No
5885	HMDB0128018	6-(4-ethyl-2-hydroxy-6-methoxyphenoxy)-3,4,5-trihydr	C15H20O9	343.1035369	No
5885	HMDB0128043	6-(2-ethyl-5-hydroxy-4-methoxyphenoxy)-3,4,5-trihydr	C15H20O9	343.1035369	No
5885	HMDB0128044	6-(4-ethyl-5-hydroxy-2-methoxyphenoxy)-3,4,5-trihydr	C15H20O9	343.1035369	No
5885	HMDB0128054	6-(6-ethyl-3-hydroxy-2-methoxyphenoxy)-3,4,5-trihydr	C15H20O9	343.1035369	No
5885	HMDB0128055	6-(4-ethyl-3-hydroxy-2-methoxyphenoxy)-3,4,5-trihydr	C15H20O9	343.1035369	No
5886	HMDB0002928; CHEBI:203534	Maltitol	C12H24O11	343.1249704	Yes
5886	HMDB0006791; C05399; CHEBI:27527	Melibiitol	C12H24O11	343.1249704	Yes
5886	HMDB0029911	1-O-alpha-D-Glucopyranosyl-D-mannitol	C12H24O11	343.1249704	Yes
5886	HMDB0040937	Lactitol	C12H24O11	343.1249704	Yes
5887	HMDB0033066	(-)-erythro-Anethole glycol 1-glucoside	C16H24O8	343.1400768	Yes
5887	HMDB0033067	(-)-erythro-Anethole glycol 2-glucoside	C16H24O8	343.1400768	Yes
5887	HMDB0041509	Dihydroconiferin	C16H24O8	343.1400768	Yes
5888	HMDB0015118; C07805; CHEBI:63082	Formoterol	C19H24N2O4	343.1663819	No
5888	HMDB0015399; CHEBI:408174	Arformoterol	C19H24N2O4	343.1663819	No
5889	HMDB0037146	5(6)-Pentyl-1,4-dioxan-2-one	C18H32O6	343.2130915	Yes
5890	HMDB0001939; C07119; CHEBI:6715	Medroxyprogesterone	C22H32O3	343.2297043	No
5890	HMDB0010213; CHEBI:72637	17-HDoHE	C22H32O3	343.2297043	No
5890	HMDB0013620; CHEBI:72653	19(20)-EpDPE	C22H32O3	343.2297043	No
5890	HMDB0013621; CHEBI:72855	16(17)-EpDPE	C22H32O3	343.2297043	No
5890	HMDB0014398; C14643; CHEBI:775124	Medrysone	C22H32O3	343.2297043	No
5890	HMDB0015489; C08158; CHEBI:290629	Testosterone Propionate	C22H32O3	343.2297043	No

5890	HMDB0033896	Anacardic acid	C22H32O3	343.2297043	No
5890	HMDB0039885	3-Hydroxyneogrifolin	C22H32O3	343.2297043	No
5890	HMDB0060037	10-HDoHE	C22H32O3	343.2297043	No
5890	HMDB0060040	11-HDoHE	C22H32O3	343.2297043	No
5890	HMDB0060047	16-HDoHE	C22H32O3	343.2297043	No
5890	HMDB0060048	20-HDoHE	C22H32O3	343.2297043	No
5890	HMDB0060049	4-HDoHE	C22H32O3	343.2297043	No
5890	HMDB0060050	7-HDoHE	C22H32O3	343.2297043	No
5890	HMDB0060051	8-HDoHE	C22H32O3	343.2297043	No
5890	HMDB0060112	4-Hydroxy-all-trans-retinyl acetate	C22H32O3	343.2297043	No
5907	HMDB0001913; C07324; CHEBI:7772	Omeprazole	C17H19N3O3S	344.1069792	No
5907	HMDB0005009; CHEBI:775271	(S)-Esomeprazole	C17H19N3O3S	344.1069792	No
5907	HMDB0014874; CHEBI:50275	Esomeprazole	C17H19N3O3S	344.1069792	No
5907	HMDB0031796; C11002; CHEBI:6009	Isofenphos	C15H24NO4PS	344.1069792	No
5913	HMDB0061637	3-hydroxyundecanoyl carnitine	C18H35NO5	344.2445847	No
5930	HMDB0015247; C07266; CHEBI:7565	Nifedipine	C17H18N2O6	345.1086415	Yes
5931	HMDB0032742	Di-O-methylcrenatin	C15H22O9	345.118446	Yes
5931	HMDB0035057	1-(3-Hydroxy-4-Methoxyphenyl)-1,2-ethanediol 3''-O-b	C15H22O9	345.118446	Yes
5931	HMDB0036562; C09771	Aucubin	C15H22O9	345.118446	Yes
5931	HMDB0040601	(1xi,2xi)-1-(4-Hydroxyphenyl)-1,2,3-propanetriol 2-O-b	C15H22O9	345.118446	Yes
5931	HMDB0040602	(1xi,2xi)-1-(4-Hydroxyphenyl)-1,2,3-propanetriol 3-O-b	C15H22O9	345.118446	Yes
5931	HMDB0041189	3,4,5-Trimethoxyphenyl glucoside	C15H22O9	345.118446	Yes
5933	HMDB0033222	(1R,3S,4S,6R)-6,9-Dihydroxyfenchone 6-O-b-D-glucosic	C16H26O8	345.1557693	Yes
5933	HMDB0033223	(1S,3R,4R)-8,10-Dihydroxyfenchone 10-O-b-D-glucosid	C16H26O8	345.1557693	Yes
5933	HMDB0035822	Glucosyl 6-hydroxy-2,6-dimethyl-2E,7-octadienoate	C16H26O8	345.1557693	Yes
5933	HMDB0038149	Nepetaside	C16H26O8	345.1557693	Yes
5935	HMDB0029885; CHEBI:1187364	Sorbitan laurate	C18H34O6	345.2287518	Yes
5964	HMDB0000045; C00020; CHEBI:16027	Adenosine monophosphate	C10H14N5O7P	346.0548736	No
5964	HMDB0001044; C00362; CHEBI:16192	2''-Deoxyguanosine 5''-monophosphate	C10H14N5O7P	346.0548736	No
5964	HMDB0003540; C01367; CHEBI:28931	3''-AMP	C10H14N5O7P	346.0548736	No
5964	HMDB0011617; C00946; CHEBI:28223	Adenosine 2''-phosphate	C10H14N5O7P	346.0548736	No
5964	HMDB0059594; C19970; CHEBI:63211	2-hydroxy-dAMP	C10H14N5O7P	346.0548736	No
5965	HMDB0014707; C06895; CHEBI:3534	Cephalexin	C16H17N3O4S	346.0874909	No
5993	HMDB0062442; C19592; CHEBI:82583	alpha-(1,2-Dihydroxyethyl)-1,2,3,4-tetrahydro-7-hydro	C17H16O8	347.076426	Yes
5993	HMDB0125025	7-hydroxy-6-methoxy-3-(2,3,5-trihydroxy-4-methoxypl	C17H16O8	347.076426	Yes
5993	HMDB0125040	7-hydroxy-6-methoxy-3-(2,3,6-trihydroxy-4-methoxypl	C17H16O8	347.076426	Yes
5993	HMDB0125055	3-(2,5-dihydroxy-4-methoxyphenyl)-7,8-dihydroxy-6-m	C17H16O8	347.076426	Yes
5993	HMDB0125070	3-(2,5-dihydroxy-4-methoxyphenyl)-5,7-dihydroxy-6-m	C17H16O8	347.076426	Yes
5993	HMDB0128201	5,6,7-trihydroxy-2-(4-hydroxy-3,5-dimethoxyphenyl)-3	C17H16O8	347.076426	Yes
5993	HMDB0128204	3-(4-hydroxy-3,5-dimethoxyphenyl)-1-(2,3,4,6-tetrahy	C17H16O8	347.076426	Yes
5993	HMDB0128218	5,7-dihydroxy-2-(1-hydroxy-3,5-dimethoxy-4-oxocyclo	C17H16O8	347.076426	Yes
5993	HMDB0128751	3,5,7-trihydroxy-2-(4-hydroxy-3-methoxyphenyl)-6-me	C17H16O8	347.076426	Yes
5993	HMDB0128753	3-(4-hydroxy-3-methoxyphenyl)-1-(2,4,6-trihydroxy-3-	C17H16O8	347.076426	Yes
5993	HMDB0128764	3,5,7-trihydroxy-2-(3-hydroxy-5-methoxyphenyl)-6-me	C17H16O8	347.076426	Yes
5993	HMDB0128765	3-(3-hydroxy-5-methoxyphenyl)-1-(2,4,6-trihydroxy-3-	C17H16O8	347.076426	Yes
5993	HMDB0129276	2-(3,4-dimethoxyphenyl)-3,5,7,8-tetrahydroxy-3,4-dih	C17H16O8	347.076426	Yes
5993	HMDB0129283	5,7,8-trihydroxy-2-(4-hydroxy-3-methoxyphenyl)-3-me	C17H16O8	347.076426	Yes
5993	HMDB0129290	5,7,8-trihydroxy-2-(3-hydroxy-4-methoxyphenyl)-3-me	C17H16O8	347.076426	Yes
5993	HMDB0129293	3-(3,4-dimethoxyphenyl)-1-(2,3,4,6-tetrahydroxypheny	C17H16O8	347.076426	Yes
5993	HMDB0130264	3,5,7-trihydroxy-2-(3-hydroxy-4-methoxyphenyl)-6-me	C17H16O8	347.076426	Yes
5993	HMDB0130265	3-(3-hydroxy-4-methoxyphenyl)-1-(2,4,6-trihydroxy-3-	C17H16O8	347.076426	Yes
5993	HMDB0130267	5,6,7-trihydroxy-2-(3-hydroxy-4-methoxyphenyl)-3-me	C17H16O8	347.076426	Yes
5993	HMDB0130272	2-(3,4-dihydroxyphenyl)-5,7-dihydroxy-3,6-dimethoxy-	C17H16O8	347.076426	Yes
5993	HMDB0136932	3,5,7-trihydroxy-2-(4-hydroxy-3,5-dimethoxyphenyl)-3	C17H16O8	347.076426	Yes
5995	HMDB0033009	Foeniculoside VIII	C16H28O8	347.1712012	Yes
5995	HMDB0033011	Foeniculoside IX	C16H28O8	347.1712012	Yes
5995	HMDB0033219	(1S,2S,4R,5S,7S)-2,5,7-Fenchanetriol 2-O-b-D-glucoside	C16H28O8	347.1712012	Yes
5995	HMDB0033236	cis-10-Hydroxylinalyl oxide 7-glucoside	C16H28O8	347.1712012	Yes
5995	HMDB0033645	(1S,2S,4S,5S)-2,4,7-Thujanetriol 4-glucoside	C16H28O8	347.1712012	Yes
5995	HMDB0034786	(1S,2S,4S)-1,8-Epoxy-p-menthane-2,7-diol 2-O-b-D-glu	C16H28O8	347.1712012	Yes
5995	HMDB0034874	Foeniculoside V	C16H28O8	347.1712012	Yes

5995	HMDB0039014	Nepetariaside	C16H28O8	347.1712012	Yes
5995	HMDB0039469	(1S,2S,4R)-p-Menth-8-ene-1,2,10-triol 2-glucoside	C16H28O8	347.1712012	Yes
5995	HMDB0041546	Foeniculoside VII	C16H28O8	347.1712012	Yes
5996	HMDB0015695; CHEBI:171339	Roxatidine acetate	C19H28N2O4	347.1984562	No
5998	HMDB0000353	3b,15b,17a-Trihydroxy-pregnenone	C21H32O4	347.222954	Yes
5998	HMDB0006755; C05478	3a,21-Dihydroxy-5b-pregnane-11,20-dione	C21H32O4	347.222954	Yes
5998	HMDB0006757; C05475	11b,21-Dihydroxy-5b-pregnane-3,20-dione	C21H32O4	347.222954	Yes
5998	HMDB0006762; HMDB0000382; C05487; CHI	17alpha,21-Dihydroxypregnenolone	C21H32O4	347.222954	Yes
5998	HMDB0012848	7''-Carboxy-alpha-chromanol	C21H32O4	347.222954	Yes
5998	HMDB0029475	[10]-Gingerdione	C21H32O4	347.222954	Yes
5998	HMDB0040641	[8]-Paradyl acetate	C21H32O4	347.222954	Yes
5998	HMDB0040954	9alpha-(3-Methyl-2E-pentenoyloxy)-4S-hydroxy-10(14)	C21H32O4	347.222954	Yes
5998	HMDB0137416	(4E)-6-hydroxy-1-(4-hydroxy-3-methoxyphenyl)tetrade	C21H32O4	347.222954	Yes
6031	HMDB0037851	Apigenin 7-sulfate	C15H10O8S	349.0025985	Yes
6031	HMDB0129972	[4-(5,7-dihydroxy-4-oxo-4H-chromen-3-yl)phenyl]oxida	C15H10O8S	349.0025985	Yes
6031	HMDB0133329	[2-hydroxy-5-(7-hydroxy-4-oxo-4H-chromen-2-yl)phen	C15H10O8S	349.0025985	Yes
6031	HMDB0133354	[3-(6,7-dihydroxy-4-oxo-4H-chromen-2-yl)phenyl]oxida	C15H10O8S	349.0025985	Yes
6031	HMDB0133378	[3-(5,7-dihydroxy-4-oxo-4H-chromen-2-yl)phenyl]oxida	C15H10O8S	349.0025985	Yes
6031	HMDB0137556	{2-hydroxy-5-[(6-hydroxy-3-oxo-2,3-dihydro-1-benzofu	C15H10O8S	349.0025985	Yes
6033	HMDB0129500	2-(2,5-dihydroxy-4-methoxyphenyl)-3,5,6,7-tetrahydro	C16H14O9	349.0546704	Yes
6033	HMDB0129508	3-(2,5-dihydroxy-4-methoxyphenyl)-1-(2,3,4,6-tetrahy	C16H14O9	349.0546704	Yes
6033	HMDB0131887	3,5,7-trihydroxy-6-methoxy-2-(3,4,5-trihydroxyphenyl)	C16H14O9	349.0546704	Yes
6033	HMDB0131892	3,5,7-trihydroxy-6-methoxy-2-(2,3,4-trihydroxyphenyl)	C16H14O9	349.0546704	Yes
6033	HMDB0131894	3,5,7-trihydroxy-6-methoxy-2-(2,4,5-trihydroxyphenyl)	C16H14O9	349.0546704	Yes
6035	HMDB0014561; C08126; CHEBI:27446	Penicillin V	C16H18N2O5S	349.0872605	No
6036	HMDB0001425; C02538; CHEBI:17474	Estrone sulfate	C18H22O5S	349.1111299	No
6038	HMDB0034784	trans-p-Menthane-1,7,8-triol 8-glucoside	C16H30O8	349.1861684	Yes
6038	HMDB0039894	(1R,2R,4S)-p-Menthane-1,2,8-triol 8-glucoside	C16H30O8	349.1861684	Yes
6038	HMDB0039977	(1S,2R,4R,8S)-p-Menthane-2,8,9-triol 2-glucoside	C16H30O8	349.1861684	Yes
6038	HMDB0039978	(1S,2R,4R,8S)-p-Menthane-2,8,9-triol 9-glucoside	C16H30O8	349.1861684	Yes
6039	HMDB0002341	8-iso-15-keto-PGE2	C20H30O5	349.2025793	Yes
6039	HMDB0002664; C06439; CHEBI:28031	Prostaglandin E3	C20H30O5	349.2025793	Yes
6039	HMDB0003034; C13802; CHEBI:34939	Prostaglandin D3	C20H30O5	349.2025793	Yes
6039	HMDB0003175; C04707; CHEBI:15547	15-Keto-prostaglandin E2	C20H30O5	349.2025793	Yes
6039	HMDB0010410	Resolvin E1	C20H30O5	349.2025793	Yes
6039	HMDB0012552	12-Oxo-20-hydroxy-leukotriene B4	C20H30O5	349.2025793	Yes
6039	HMDB0012588	15-Epi-lipoxin B5	C20H30O5	349.2025793	Yes
6039	HMDB0012590	15-Oxo-lipoxin A4	C20H30O5	349.2025793	Yes
6039	HMDB0012641; CHEBI:63979	20-oxo-leukotriene B4	C20H30O5	349.2025793	Yes
6039	HMDB0013040; C00427; CHEBI:15554	PGH3	C20H30O5	349.2025793	Yes
6039	HMDB0036701	(ent-6alpha,7alpha,12alpha)-6,7,12-Trihydroxy-16-kau	C20H30O5	349.2025793	Yes
6039	HMDB0037593	Oryzalic acid A	C20H30O5	349.2025793	Yes
6039	HMDB0038693	Oryzalic acid B	C20H30O5	349.2025793	Yes
6039	HMDB0060096	5,12,18R-TriHEPE	C20H30O5	349.2025793	Yes
6039	HMDB0062297; C04758; CHEBI:15557	9S-hydroxy-11,15-dioxo-5Z,13E-prostadienoic acid	C20H30O5	349.2025793	Yes
6039	HMDB0062300; CHEBI:63992	15-oxo-5S,6R-dihydroxy-7E,9E,11Z,13E-eicosatetraeno	C20H30O5	349.2025793	Yes
6039	HMDB0062616; CHEBI:91287	(5S)-hydroperoxy-18-hydroxy-EPE	C20H30O5	349.2025793	Yes
6040	HMDB0000268; C05476	Tetrahydrocorticosterone	C21H34O4	349.2382471	No
6040	HMDB0000449	5a-Tetrahydrocorticosterone	C21H34O4	349.2382471	No
6040	HMDB0005972; C14594	Tetrahydrodeoxycortisol	C21H34O4	349.2382471	No
6040	HMDB0011541	MG(0:0/18:4(6Z,9Z,12Z,15Z)/0:0)	C21H34O4	349.2382471	No
6040	HMDB0011571	MG(18:4(6Z,9Z,12Z,15Z)/0:0/0:0)	C21H34O4	349.2382471	No
6040	HMDB0033616; C17496	(S)-[10]-Gingerol	C21H34O4	349.2382471	No
6040	HMDB0041997; C15368; CHEBI:79870	Pregnanetriolone	C21H34O4	349.2382471	No
6052	HMDB0014952; C08169; CHEBI:120496	Meloxicam	C14H13N3O4S2	350.0277026	No
6057	HMDB0060001; CHEBI:89622	Indole-3-acetic-acid-O-glucuronide	C16H17NO8	350.0885483	Yes
6059	HMDB0030388	9-(beta-D-Ribofuranosyl)zeatin	C15H21N5O5	350.1462014	No
6080	HMDB0039720	a-L-threo-4-Hex-4-enopyranuronosyl-D-galacturonic ac	C12H16O12	351.0571519	Yes
6082	HMDB0126417	6-[(2,2-dimethyl-2H-chromen-5-yl)oxy]-3,4,5-trihydrox	C17H20O8	351.1079935	Yes
6082	HMDB0132988	3,4,5-trihydroxy-6-{4-[(1E)-3-oxopent-1-en-1-yl]pheno:	C17H20O8	351.1079935	Yes
6082	HMDB0133688	3,4,5-trihydroxy-6-[(3-methyl-2-oxo-4-phenylbut-3-en-	C17H20O8	351.1079935	Yes

6082	HMDB0133690	3,4,5-trihydroxy-6-[3-oxo-2-(phenylmethylidene)butox	C17H20O8	351.1079935	Yes
6082	HMDB0133692	3,4,5-trihydroxy-6-[3-(2-methyl-3-oxobut-1-en-1-yl)phe	C17H20O8	351.1079935	Yes
6082	HMDB0133694	3,4,5-trihydroxy-6-[4-(2-methyl-3-oxobut-1-en-1-yl)phe	C17H20O8	351.1079935	Yes
6083	HMDB0004448; C08357; CHEBI:4866	Estradiol-17beta 3-sulfate	C18H24O5S	351.129645	No
6086	HMDB0014909; C07853; CHEBI:3743	Clidinium	C22H26NO3	351.1827905	Yes
6086	HMDB0039132	[4]-Gingerdiol 3,5-diacetate	C19H28O6	351.1827905	Yes
6087	HMDB0001220; C00584; CHEBI:15551	Prostaglandin E2	C20H32O5	351.2178733	Yes
6087	HMDB0001320; C04654; CHEBI:15548	(13E)-11a-Hydroxy-9,15-dioxoprost-13-enoic acid	C20H32O5	351.2178733	Yes
6087	HMDB0001335; C01312; CHEBI:15552	Prostaglandin I2	C20H32O5	351.2178733	Yes
6087	HMDB0001381; C00427; CHEBI:15554	Prostaglandin H2	C20H32O5	351.2178733	Yes
6087	HMDB0001403; C00696; CHEBI:15555	Prostaglandin D2	C20H32O5	351.2178733	Yes
6087	HMDB0001452; C02198; CHEBI:15627	Thromboxane A2	C20H32O5	351.2178733	Yes
6087	HMDB0001509; C04853; CHEBI:15646	20-Hydroxy-leukotriene B4	C20H32O5	351.2178733	Yes
6087	HMDB0002122; C06476; CHEBI:36075	Prostaglandin F3a	C20H32O5	351.2178733	Yes
6087	HMDB0002132	8-iso-PGF3a	C20H32O5	351.2178733	Yes
6087	HMDB0002363; C13807; CHEBI:34821	Levuglandin E2	C20H32O5	351.2178733	Yes
6087	HMDB0002400; C13808; CHEBI:34820	Levuglandin D2	C20H32O5	351.2178733	Yes
6087	HMDB0002776; C04671; CHEBI:15550	13,14-Dihydro-15-keto-PGE2	C20H32O5	351.2178733	Yes
6087	HMDB0004240; C05960; CHEBI:28442	15-Keto-prostaglandin F2a	C20H32O5	351.2178733	Yes
6087	HMDB0004385; C06314; CHEBI:6498	Lipoxin A4	C20H32O5	351.2178733	Yes
6087	HMDB0005077	8-iso-15-keto-PGF2a	C20H32O5	351.2178733	Yes
6087	HMDB0005082; C06315; CHEBI:6499	Lipoxin B4	C20H32O5	351.2178733	Yes
6087	HMDB0005844; CHEBI:131888	8-isoprostaglandin E2	C20H32O5	351.2178733	Yes
6087	HMDB0012481; C04671; CHEBI:15550	(5Z)-(15S)-11alpha-Hydroxy-9,15-dioxoprostanoate	C20H32O5	351.2178733	Yes
6087	HMDB0012564	13,14-Dihydro-15-oxo-lipoxin A4	C20H32O5	351.2178733	Yes
6087	HMDB0012587	15-Epi-lipoxin A4	C20H32O5	351.2178733	Yes
6087	HMDB0034676	Cinncassiol D1	C20H32O5	351.2178733	Yes
6087	HMDB0035064	Cinncassiol D4	C20H32O5	351.2178733	Yes
6087	HMDB0035338	Sterebin B	C20H32O5	351.2178733	Yes
6087	HMDB0035339	Sterebin C	C20H32O5	351.2178733	Yes
6087	HMDB0036756	(ent-6alpha,7alpha,16alphaH)-6,7,17-Trihydroxy-19-ka	C20H32O5	351.2178733	Yes
6087	HMDB0060041	11b-PGE2	C20H32O5	351.2178733	Yes
6087	HMDB0060042	13,14-Dihydro-15-keto-PGD2	C20H32O5	351.2178733	Yes
6087	HMDB0062291; CHEBI:63993	15-oxo-5S,6R-dihydroxy-7E,9E,11Z-eicosatrienoic acid	C20H32O5	351.2178733	Yes
6087	HMDB0062798; CHEBI:90833	5-hydroperoxy-15-HETE	C20H32O5	351.2178733	Yes
6088	HMDB0011539	MG(0:0/18:3(6Z,9Z,12Z)/0:0)	C21H36O4	351.2535494	No
6088	HMDB0011540	MG(0:0/18:3(9Z,12Z,15Z)/0:0)	C21H36O4	351.2535494	No
6088	HMDB0011569	MG(18:3(6Z,9Z,12Z)/0:0/0:0)	C21H36O4	351.2535494	No
6088	HMDB0011570	MG(18:3(9Z,12Z,15Z)/0:0/0:0)	C21H36O4	351.2535494	No
6099	HMDB0032802	4-Hydroxybenzyl isothiocyanate 4''''-acetylrhamnoside	C16H19NO6S	352.0863415	No
6103	HMDB0060595	Olopatadine n-oxide	C21H23NO4	352.1533873	No
6143	HMDB0031758	Allithiamine	C15H22N4O2S2	353.1097443	Yes
6144	HMDB0040797	Methyl helianthenoate F glucoside	C17H22O8	353.1256019	Yes
6144	HMDB0040898	Methyl (R)-8-Hydroxy-9-decene-4,6-diynoate glucoside	C17H22O8	353.1256019	Yes
6144	HMDB0126175	3,4,5-trihydroxy-6-[3-hydroxy-2-(3-methylbut-2-en-1-y	C17H22O8	353.1256019	Yes
6144	HMDB0133001	3,4,5-trihydroxy-6-[4-(3-oxopentyl)phenoxy]oxane-2-c	C17H22O8	353.1256019	Yes
6144	HMDB0133204	3,4,5-trihydroxy-6-[5-hydroxy-2-(3-methylbut-2-en-1-y	C17H22O8	353.1256019	Yes
6144	HMDB0133205	3,4,5-trihydroxy-6-[3-hydroxy-4-(3-methylbut-2-en-1-y	C17H22O8	353.1256019	Yes
6144	HMDB0133698	3,4,5-trihydroxy-6-[(2-methyl-3-oxo-1-phenylbutan-2-y	C17H22O8	353.1256019	Yes
6144	HMDB0133699	3,4,5-trihydroxy-6-(2-methyl-3-oxo-1-phenylbutoxy)ox	C17H22O8	353.1256019	Yes
6144	HMDB0133701	3,4,5-trihydroxy-6-[3-(2-methyl-3-oxobutyl)phenoxy]o:	C17H22O8	353.1256019	Yes
6144	HMDB0133703	3,4,5-trihydroxy-6-[4-(2-methyl-3-oxobutyl)phenoxy]o:	C17H22O8	353.1256019	Yes
6144	HMDB0133705	6-(2-benzyl-3-oxobutoxy)-3,4,5-trihydroxyoxane-2-carl	C17H22O8	353.1256019	Yes
6144	HMDB0133707	3,4,5-trihydroxy-6-(3-methyl-2-oxo-4-phenylbutoxy)ox	C17H22O8	353.1256019	Yes
6146	HMDB0041513	Isopropyl apiosylglucoside	C14H26O10	353.1456068	Yes
6148	HMDB0060933	Propofol glucuronide	C18H26O7	353.1617693	Yes
6149	HMDB0031877	Acetylsalvipisone	C22H26O4	353.1759995	No
6149	HMDB0062319; CHEBI:62840	1-dodecanoyl-glycero-3-phosphate	C15H31O7P	353.1759995	No
6149	HMDB0114763	LPA(i-12:0/0:0)	C15H31O7P	353.1759995	No
6153	HMDB0001139; C00639; CHEBI:15553	Prostaglandin F2a	C20H34O5	353.2339697	Yes
6153	HMDB0001442; C04741; CHEBI:15544	Prostaglandin E1	C20H34O5	353.2339697	Yes

6153	HMDB0001483; C02314; CHEBI:28922	Prostaglandin F2b	C20H34O5	353.2339697	Yes
6153	HMDB0001965; C14811; CHEBI:35032	Troxilin B3	C20H34O5	353.2339697	Yes
6153	HMDB0001977; C14809; CHEBI:36203	Trioxilin A3	C20H34O5	353.2339697	Yes
6153	HMDB0002115	8-isoprostaglandin PGF2b	C20H34O5	353.2339697	Yes
6153	HMDB0004684; C14782	11,12,15-THETA	C20H34O5	353.2339697	Yes
6153	HMDB0004685; CHEBI:63976	13,14-Dihydro-15-keto PGF2a	C20H34O5	353.2339697	Yes
6153	HMDB0004686	8-Isoprostaglandin E1	C20H34O5	353.2339697	Yes
6153	HMDB0004694; C14814	11,14,15-THETA	C20H34O5	353.2339697	Yes
6153	HMDB0005083; C13809; CHEBI:34505	8-Isoprostaglandin F2a	C20H34O5	353.2339697	Yes
6153	HMDB0005102; C06438; CHEBI:27696	Prostaglandin D1	C20H34O5	353.2339697	Yes
6153	HMDB0006562	8-iso-13,14-dihydro-15-keto-PGF2a	C20H34O5	353.2339697	Yes
6153	HMDB0010170	11,12,15-TriHETRE	C20H34O5	353.2339697	Yes
6153	HMDB0010199; C05959; CHEBI:27595	11b-PGF2a	C20H34O5	353.2339697	Yes
6153	HMDB0011137; C05959; CHEBI:27595	11-Epi-PGF2a	C20H34O5	353.2339697	Yes
6153	HMDB0012563	13,14-Dihydro- lipoxin A4	C20H34O5	353.2339697	Yes
6153	HMDB0013041	Prostaglandin H1	C20H34O5	353.2339697	Yes
6153	HMDB0035380	Sterebin G	C20H34O5	353.2339697	Yes
6153	HMDB0060045; CHEBI:72593	15-keto-PGF1alpha	C20H34O5	353.2339697	Yes
6153	HMDB0060107; CHEBI:15630	(5Z,9E,12S,14Z)-8,11,12-Trihydroxyicosa-5,9,14-trieno	C20H34O5	353.2339697	Yes
6153	HMDB0062278	11,12,15-trihydroxyeicosatrienoic acid	C20H34O5	353.2339697	Yes
6155	HMDB0001160; CHEBI:42394	Tricosanoic acid	C23H46O2	353.3431536	No
6155	HMDB0035638	6-Hydroxy-8-tricosanone	C23H46O2	353.3431536	No
6155	HMDB0035642	8-Hydroxy-6-tricosanone	C23H46O2	353.3431536	No
6155	HMDB0035649	4-Hydroxy-6-tricosanone	C23H46O2	353.3431536	No
6155	HMDB0035650	6-Hydroxy-4-tricosanone	C23H46O2	353.3431536	No
6174	HMDB0000988; C01137; CHEBI:15625	S-Adenosylmethioninamine	C14H23N6O3S	354.1489139	Yes
6178	HMDB0033449	Pipercide	C22H29NO3	354.207179	No
6178	HMDB0144279	4-(dimethylamino)-4-hydroxy-3-methyl-1,2-diphenylbu	C22H29NO3	354.207179	No
6178	HMDB0144280	4-(dimethylamino)-3-hydroxy-3-methyl-1,2-diphenylbu	C22H29NO3	354.207179	No
6178	HMDB0144282	4-(dimethylamino)-1-hydroxy-3-methyl-1,2-diphenylbu	C22H29NO3	354.207179	No
6178	HMDB0144283	4-(dimethylamino)-2-(3-hydroxyphenyl)-3-methyl-1-ph	C22H29NO3	354.207179	No
6178	HMDB0144284	4-(dimethylamino)-1-(3-hydroxyphenyl)-3-methyl-2-ph	C22H29NO3	354.207179	No
6178	HMDB0144285	4-(dimethylamino)-2-(4-hydroxyphenyl)-3-methyl-1-ph	C22H29NO3	354.207179	No
6178	HMDB0144286	4-(dimethylamino)-1-(4-hydroxyphenyl)-3-methyl-2-ph	C22H29NO3	354.207179	No
6178	HMDB0144287	4-(dimethylamino)-3-methyl-1,2-diphenylbutan-2-yl 2-	C22H29NO3	354.207179	No
6178	HMDB0144288	3-[(dimethylamino)methyl]-4-hydroxy-1,2-diphenylbut	C22H29NO3	354.207179	No
6178	HMDB0144289	4-(dimethylamino)-3-methyl-1,2-diphenylbutan-2-yl 3-	C22H29NO3	354.207179	No
6178	HMDB0144290	4-(dimethylamino)-2-(2-hydroxyphenyl)-3-methyl-1-ph	C22H29NO3	354.207179	No
6178	HMDB0144291	4-(dimethylamino)-1-(2-hydroxyphenyl)-3-methyl-2-ph	C22H29NO3	354.207179	No
6178	HMDB0144292	4-(dimethyl-oxo-??-azanyl)-3-methyl-1,2-diphenylbuta	C22H29NO3	354.207179	No
6196	HMDB0036617	(+)-Chebulic acid	C14H12O11	355.0294836	Yes
6198	HMDB0127752	{3-[2-hydroxy-3-(2,4,6-trihydroxyphenyl)propyl]phenyl	C15H16O8S	355.0501836	No
6198	HMDB0127753	{[1-(3-hydroxyphenyl)-3-(2,4,6-trihydroxyphenyl)propa	C15H16O8S	355.0501836	No
6198	HMDB0128653	{[4-(7-hydroxy-8-methoxy-2-oxo-2H-chromen-6-yl)-2-n	C15H16O8S	355.0501836	No
6198	HMDB0132699	({3-[(7-methoxy-2-oxo-2H-chromen-8-yl)methyl]-2-me	C15H16O8S	355.0501836	No
6198	HMDB0132708	{[1-(6-hydroxy-7-methoxy-2-oxo-2H-chromen-8-yl)-3-n	C15H16O8S	355.0501836	No
6198	HMDB0132713	[3-hydroxy-4-(7-methoxy-2-oxo-2H-chromen-8-yl)-2-m	C15H16O8S	355.0501836	No
6198	HMDB0132714	{[4-hydroxy-1-(7-methoxy-2-oxo-2H-chromen-8-yl)-3-n	C15H16O8S	355.0501836	No
6198	HMDB0132717	{[2-hydroxy-1-(7-methoxy-2-oxo-2H-chromen-8-yl)-3-n	C15H16O8S	355.0501836	No
6198	HMDB0132718	{[1-hydroxy-1-(7-methoxy-2-oxo-2H-chromen-8-yl)-3-n	C15H16O8S	355.0501836	No
6198	HMDB0132720	[2-(7-methoxy-2-oxo-2H-chromen-8-yl)-1-(2-methyloxi	C15H16O8S	355.0501836	No
6200	HMDB0029938	Acaciabiuronic acid	C12H20O12	355.0890137	No
6200	HMDB0039722	2-O-beta-D-Glucopyranuronosyl-D-mannose	C12H20O12	355.0890137	No
6200	HMDB0039724	4-O-alpha-D-Glucopyranuronosyl-D-galactose	C12H20O12	355.0890137	No
6200	HMDB0039726	3-O-beta-D-Galactopyranuronosyl-D-galactose	C12H20O12	355.0890137	No
6200	HMDB0125609	6-{[4,5-dihydroxy-2,5-bis(hydroxymethyl)oxolan-3-yl]o	C12H20O12	355.0890137	No
6200	HMDB0125610	6-{[2,4-dihydroxy-2,5-bis(hydroxymethyl)oxolan-3-yl]o	C12H20O12	355.0890137	No
6200	HMDB0125611	3,4,5-trihydroxy-6-{[2,3,4-trihydroxy-5-(hydroxymethy	C12H20O12	355.0890137	No
6200	HMDB0125612	3,4,5-trihydroxy-6-{[3,4,5-trihydroxy-5-(hydroxymethy	C12H20O12	355.0890137	No
6201	HMDB0031695	xi-3-Hydroxy-5-phenylpentanoic acid O-beta-D-Glucop	C17H24O8	355.1398382	No
6201	HMDB0040558	Zingerone glucoside	C17H24O8	355.1398382	No

6203	HMDB0035099	gamma-Crocetin	C22H28O4	355.1919214	No
6203	HMDB0040634	Piperochromanoic acid	C22H28O4	355.1919214	No
6204	HMDB0041044	5,7-Megastigmadien-9-ol glucoside	C19H32O6	355.2124598	No
6205	HMDB0041882	Dopexamine	C22H32N2O2	355.241694	No
6206	HMDB0002685; C06475; CHEBI:28852	Prostaglandin F1a	C20H36O5	355.2493986	No
6206	HMDB0002689; CHEBI:1000694	13,14-Dihydro PGE1	C20H36O5	355.2493986	No
6206	HMDB0004239	13,14-Dihydro PGF2a	C20H36O5	355.2493986	No
6224	HMDB0029833; CHEBI:134458	Cyclodopa glucoside	C15H19NO9	356.0986808	No
6224	HMDB0037263	HMBOA-Glc	C15H19NO9	356.0986808	No
6224	HMDB0240215; CHEBI:133005	2-Methoxyacetaminophen glucuronide	C15H19NO9	356.0986808	No
6225	HMDB0031944	Niazimicin A	C16H23NO6S	356.1173563	Yes
6233	HMDB0094648	Leu-Leu-Leu	C18H35N3O4	356.2549344	No
6250	HMDB0132923	(2-hydroxy-2-{9-hydroxy-7-oxo-2H,3H,7H-furo[3,2-g]ch	C14H14O9S	357.0308579	No
6250	HMDB0132925	[9-hydroxy-2-(2-hydroxypropan-2-yl)-7-oxo-2H,3H,7H-	C14H14O9S	357.0308579	No
6252	HMDB0041720	Dihydrocaffeic acid 3-O-glucuronide	C15H18O10	357.0831058	Yes
6252	HMDB0124979	6-[4-(2-carboxyethyl)-2-hydroxyphenoxy]-3,4,5-trihydr	C15H18O10	357.0831058	Yes
6252	HMDB0124980	6-[5-(2-carboxyethyl)-2-hydroxyphenoxy]-3,4,5-trihydr	C15H18O10	357.0831058	Yes
6252	HMDB0124981	6-{[3-(3,4-dihydroxyphenyl)propanoyl]oxy}-3,4,5-trihy	C15H18O10	357.0831058	Yes
6252	HMDB0126431	6-[4-(2-carboxyethyl)-3-hydroxyphenoxy]-3,4,5-trihydr	C15H18O10	357.0831058	Yes
6252	HMDB0126432	6-[2-(2-carboxyethyl)-5-hydroxyphenoxy]-3,4,5-trihydr	C15H18O10	357.0831058	Yes
6252	HMDB0126433	6-{[3-(2,4-dihydroxyphenyl)propanoyl]oxy}-3,4,5-trihy	C15H18O10	357.0831058	Yes
6252	HMDB0128026	6-[3-(2-carboxyethyl)-5-hydroxyphenoxy]-3,4,5-trihydr	C15H18O10	357.0831058	Yes
6252	HMDB0128027	6-{[3-(3,5-dihydroxyphenyl)propanoyl]oxy}-3,4,5-trihy	C15H18O10	357.0831058	Yes
6252	HMDB0133881	6-[2-(1-carboxyethyl)-5-hydroxyphenoxy]-3,4,5-trihydr	C15H18O10	357.0831058	Yes
6252	HMDB0133882	6-[4-(1-carboxyethyl)-3-hydroxyphenoxy]-3,4,5-trihydr	C15H18O10	357.0831058	Yes
6252	HMDB0133883	6-{[2-(2,4-dihydroxyphenyl)propanoyl]oxy}-3,4,5-trihy	C15H18O10	357.0831058	Yes
6252	HMDB0134096	6-[3-(2-carboxyethyl)-2-hydroxyphenoxy]-3,4,5-trihydr	C15H18O10	357.0831058	Yes
6252	HMDB0134097	6-[2-(2-carboxyethyl)-6-hydroxyphenoxy]-3,4,5-trihydr	C15H18O10	357.0831058	Yes
6252	HMDB0134098	6-{[3-(2,3-dihydroxyphenyl)propanoyl]oxy}-3,4,5-trihy	C15H18O10	357.0831058	Yes
6252	HMDB0134106	6-[3-(2-carboxyethyl)-4-hydroxyphenoxy]-3,4,5-trihydr	C15H18O10	357.0831058	Yes
6252	HMDB0134107	6-[2-(2-carboxyethyl)-4-hydroxyphenoxy]-3,4,5-trihydr	C15H18O10	357.0831058	Yes
6252	HMDB0134108	6-{[3-(2,5-dihydroxyphenyl)propanoyl]oxy}-3,4,5-trihy	C15H18O10	357.0831058	Yes
6254	HMDB0029651	Phlorisobutyrophenone 2-glucoside	C16H22O9	357.1189145	Yes
6260	HMDB0033443	Sorgoleone 358	C22H30O4	357.2078923	No
6260	HMDB0035856	11,12-Dimethoxy-8,11,13-abietatrien-20,7-olide	C22H30O4	357.2078923	No
6260	HMDB0040619	Piperoic acid	C22H30O4	357.2078923	No
6263	HMDB0005076; CHEBI:155467	13,14-Dihydro PGF-1a	C20H38O5	357.265047	No
6278	HMDB0034070; C08427; CHEBI:9162	Sinigrin	C10H17NO9S2	358.02595	No
6281	HMDB0030934; C08342	Triglochinin	C14H17NO10	358.0772101	Yes
6282	HMDB0010336	Epinephrine glucuronide	C15H21NO9	358.1148432	Yes
6282	HMDB0029452	L-DOPA 3''-glucoside	C15H21NO9	358.1148432	Yes
6284	HMDB0135480	N-[2-(3,4-dimethoxyphenyl)ethyl]-3-(3-hydroxy-4-metl	C20H25NO5	358.1657103	No
6284	HMDB0135481	3-(3,4-dimethoxyphenyl)-N-[2-(4-hydroxy-3-methoxyp	C20H25NO5	358.1657103	No
6284	HMDB0135482	3-(3,4-dimethoxyphenyl)-N-[2-(3-hydroxy-4-methoxyp	C20H25NO5	358.1657103	No
6284	HMDB0135483	N-[2-(3,4-dimethoxyphenyl)ethyl]-3-(4-hydroxy-3-metl	C20H25NO5	358.1657103	No
6299	HMDB0000496; C03033	3-Methoxy-4-hydroxyphenylglycol glucuronide	C15H20O10	359.0991171	Yes
6299	HMDB0035476	6''-Methoxypolygoacetophenoside	C15H20O10	359.0991171	Yes
6300	HMDB0034626	1-(3,4-Dimethoxyphenyl)-1,2-ethanediol 1-O-b-D-gluco	C16H24O9	359.1351358	Yes
6300	HMDB0034627	1-(3,4-Dimethoxyphenyl)-1,2-ethanediol 2-O-b-D-gluco	C16H24O9	359.1351358	Yes
6300	HMDB0037028; C11647	Deoxyloganic acid	C16H24O9	359.1351358	Yes
6300	HMDB0038381	2-(4-Hydroxy-3,5-dimethoxyphenyl)ethanol 4''-glucosi	C16H24O9	359.1351358	Yes
6300	HMDB0039473	2''-Methoxy-3-(2,4-dihydroxyphenyl)-1,2-propanediol 4	C16H24O9	359.1351358	Yes
6301	HMDB0032717	Lepidine C	C21H20N4O2	359.1536324	No
6301	HMDB0033437; CHEBI:48983	Lepidine	C21H20N4O2	359.1536324	No
6314	HMDB0014010; CHEBI:63840	5-Hydroxyomeprazole	C17H19N3O4S	360.1022645	No
6314	HMDB0014012; CHEBI:388428	Omeprazole sulfone	C17H19N3O4S	360.1022645	No
6314	HMDB0014013	3-Hydroxyomeprazole	C17H19N3O4S	360.1022645	No
6314	HMDB0061091	5-hydroxyesomeprazole	C17H19N3O4S	360.1022645	No
6333	HMDB0134656	[(5-oxo-1,7-diphenylheptan-2-yl)oxy]sulfonic acid	C19H22O5S	361.1141566	Yes
6333	HMDB0134658	[(5-oxo-1,7-diphenylheptan-3-yl)oxy]sulfonic acid	C19H22O5S	361.1141566	Yes
6333	HMDB0134660	[(3-oxo-1,7-diphenylheptan-4-yl)oxy]sulfonic acid	C19H22O5S	361.1141566	Yes

6333	HMDB0134662	[(3-oxo-1,7-diphenylheptan-2-yl)oxy]sulfonic acid	C19H22O5S	361.1141566	Yes
6333	HMDB0134664	[(5-oxo-1,7-diphenylheptyl)oxy]sulfonic acid	C19H22O5S	361.1141566	Yes
6333	HMDB0134666	[(3-oxo-1,7-diphenylheptyl)oxy]sulfonic acid	C19H22O5S	361.1141566	Yes
6333	HMDB0134668	[3-(5-oxo-7-phenylheptyl)phenyl]oxidanesulfonic acid	C19H22O5S	361.1141566	Yes
6333	HMDB0134670	[3-(3-oxo-7-phenylheptyl)phenyl]oxidanesulfonic acid	C19H22O5S	361.1141566	Yes
6333	HMDB0134672	[4-(5-oxo-7-phenylheptyl)phenyl]oxidanesulfonic acid	C19H22O5S	361.1141566	Yes
6333	HMDB0134674	[4-(3-oxo-7-phenylheptyl)phenyl]oxidanesulfonic acid	C19H22O5S	361.1141566	Yes
6337	HMDB0000063; C00735	Cortisol	C21H30O5	361.2044078	No
6337	HMDB0000319; C01124; CHEBI:16485	18-Hydroxycorticosterone	C21H30O5	361.2044078	No
6337	HMDB0006758; C05469; CHEBI:18093	17a,21-Dihydroxy-5b-pregnane-3,11,20-trione	C21H30O5	361.2044078	No
6337	HMDB0014879; C00735; CHEBI:17650	Hydrocortisone	C21H30O5	361.2044078	No
6337	HMDB0030026	Isohumulone A	C21H30O5	361.2044078	No
6337	HMDB0030138	Adhumulone	C21H30O5	361.2044078	No
6337	HMDB0032095	5-Hydroxy-4-methoxy-5-(1-oxo-9,12,15-hexadecatrien	C21H30O5	361.2044078	No
6337	HMDB0034090	(R)-Humulone	C21H30O5	361.2044078	No
6342	HMDB0038380	Triton X 100	C24H42O2	361.3121263	No
6357	HMDB0001397; C00144; CHEBI:17345	Guanosine monophosphate	C10H14N5O8P	362.0523786	No
6357	HMDB0011670	8-Oxo-dGMP	C10H14N5O8P	362.0523786	No
6357	HMDB0059639; C18239; CHEBI:60210	Cyclic pyranopterin monophosphate	C10H14N5O8P	362.0523786	No
6358	HMDB0015271; C06878; CHEBI:3479	Cefadroxil	C16H17N3O5S	362.0840454	No
6360	HMDB0041143	8-Methyldihydrochelerythrine	C22H21NO4	362.1425288	No
6383	HMDB0060982	hydroxytorsemide	C16H20N4O4S	363.1122508	No
6387	HMDB0012550	12-Oxo-20-carboxy-leukotriene B4	C20H28O6	363.1812409	Yes
6387	HMDB0030899	Eremopetasitenin A1	C20H28O6	363.1812409	Yes
6387	HMDB0035168; C17642	Cinncassiol C2	C20H28O6	363.1812409	Yes
6387	HMDB0036665	Blumealactone A	C20H28O6	363.1812409	Yes
6387	HMDB0036666	Blumealactone B	C20H28O6	363.1812409	Yes
6387	HMDB0036944	Gibberellin A123	C20H28O6	363.1812409	Yes
6387	HMDB0038746	Gibberellin A74	C20H28O6	363.1812409	Yes
6387	HMDB0040757	3b,8a-Dihydroxy-6b-angeloyloxy-7(11)-eremophilen-1:	C20H28O6	363.1812409	Yes
6387	HMDB0040758	3b,8b-Dihydroxy-6b-angeloyloxy-7(11)-eremophilen-1:	C20H28O6	363.1812409	Yes
6387	HMDB0041226	3b,9b-Dihydroxy-6b-angeloyloxy-7(11)-eremophilen-1:	C20H28O6	363.1812409	Yes
6387	HMDB0041279	3b-Hydroxy-6b-(2,3-epoxy-2-methylbutanoyloxy)-7(11	C20H28O6	363.1812409	Yes
6387	HMDB0041348	Gibberellin A100	C20H28O6	363.1812409	Yes
6387	HMDB0041496	Gibberellin A97	C20H28O6	363.1812409	Yes
6387	HMDB0059992; CHEBI:88747	5-(4''-Hydroxyphenyl)-gamma-valerolactone-4''-O-gluc	C20H28O6	363.1812409	Yes
6390	HMDB0000903	Tetrahydrocortisone	C21H32O5	363.219541	No
6390	HMDB0003259; C05471	Dihydrocortisol	C21H32O5	363.219541	No
6390	HMDB0006753; C05474	3a,11b,21-Trihydroxy-20-oxo-5b-pregnan-18-al	C21H32O5	363.219541	No
6390	HMDB0006754; C05473	11b,21-Dihydroxy-3,20-oxo-5b-pregnan-18-al	C21H32O5	363.219541	No
6390	HMDB0006760; C05489	11b,17a,21-Trihydroxypreg-nenolone	C21H32O5	363.219541	No
6390	HMDB0013157	18-Hydroxy-11-dehydrotetrahydrocorticosterone	C21H32O5	363.219541	No
6390	HMDB0032553	Vanillin 3-(L-menthoxy)propane-1,2-diol acetal	C21H32O5	363.219541	No
6393	HMDB0036803	6-O-Acetylaustroinulin	C22H36O4	363.2521799	No
6393	HMDB0036804	7-O-Acetylaustroinulin	C22H36O4	363.2521799	No
6405	HMDB0012622	2-Hydroxyestrone sulfate	C18H21O6S	364.0979907	No
6405	HMDB0015193; C06827; CHEBI:2676	Amoxicillin	C16H19N3O5S	364.0979907	No
6405	HMDB0030500; C06827; CHEBI:323183	Amoxycillin	C16H19N3O5S	364.0979907	No
6407	HMDB0013958	5-O-Desmethyldonepezil	C23H27NO3	364.1925371	No
6407	HMDB0013959	6-O-Desmethyldonepezil	C23H27NO3	364.1925371	No
6407	HMDB0040775	Aegle marmelos Alkaloid C	C23H27NO3	364.1925371	No
6434	HMDB0029992	Tetrahydropentoxyline	C17H22N2O7	365.135785	No
6434	HMDB0033108	Semilepidinoside B	C17H22N2O7	365.135785	No
6435	HMDB0035104; C09662	Diacetoxyscirpenol	C19H26O7	365.160185	Yes
6435	HMDB0038282	4-Acetyl-2-prenylphenol glucoside	C19H26O7	365.160185	Yes
6435	HMDB0041364	Secoeremopetasitolide A	C19H26O7	365.160185	Yes
6436	HMDB0033216	(2R,6x)-7-Methyl-3-methylene-1,2,6,7-octanetetrol 2-g	C16H30O9	365.1826491	Yes
6436	HMDB0039975	(1S,2S,4R,8S)-p-Menthane-1,2,8,9-tetrol 2-glucoside	C16H30O9	365.1826491	Yes
6436	HMDB0039976	(1R*,2R*,4R*,8S*)-p-Menthane-1,2,8,9-tetrol 9-glucos	C16H30O9	365.1826491	Yes
6437	HMDB0006059; C05950; CHEBI:27562	20-Carboxy-leukotriene B4	C20H30O6	365.1961178	Yes
6437	HMDB0012549	12-oxo-10,11-dihydro-20-COOH-LTB4	C20H30O6	365.1961178	Yes

6437	HMDB0012551	12-oxo-20-dihydroxy-leukotriene B4	C20H30O6	365.1961178	Yes
6437	HMDB0036863	Anhydrocinnzeylanol	C20H30O6	365.1961178	Yes
6437	HMDB0037062	Sporotrichiol	C20H30O6	365.1961178	Yes
6440	HMDB0000314	3b-Allotetrahydrocortisol	C21H34O5	365.2333752	No
6440	HMDB0000526	5a-Tetrahydrocortisol	C21H34O5	365.2333752	No
6440	HMDB0000949; C05472	Tetrahydrocortisol	C21H34O5	365.2333752	No
6440	HMDB0003128; C05481; CHEBI:3899	Cortolone	C21H34O5	365.2333752	No
6440	HMDB0013221; C05481	Beta-Cortolone	C21H34O5	365.2333752	No
6441	HMDB0000314	3b-Allotetrahydrocortisol	C21H34O5	365.2338599	No
6441	HMDB0000526	5a-Tetrahydrocortisol	C21H34O5	365.2338599	No
6441	HMDB0000949; C05472	Tetrahydrocortisol	C21H34O5	365.2338599	No
6441	HMDB0003128; C05481; CHEBI:3899	Cortolone	C21H34O5	365.2338599	No
6441	HMDB0013221; C05481	Beta-Cortolone	C21H34O5	365.2338599	No
6444	HMDB0002368; C08323; CHEBI:44247	Nervonic acid	C24H46O2	365.3424306	No
6444	HMDB0029799	(E)-2-Tetracosenoic acid	C24H46O2	365.3424306	No
6444	HMDB0035559	10,12-Tetracosanedione	C24H46O2	365.3424306	No
6444	HMDB0035560	7,9-Tetracosanedione	C24H46O2	365.3424306	No
6444	HMDB0035561	6,8-Tetracosanedione	C24H46O2	365.3424306	No
6444	HMDB0035562	5,7-Tetracosanedione	C24H46O2	365.3424306	No
6444	HMDB0035563	4,6-Tetracosanedione	C24H46O2	365.3424306	No
6478	HMDB0128633	3,4,5-trihydroxy-6-{[3-(hydroxymethyl)-1-oxo-1H-isoch	C16H16O10	367.0682889	Yes
6478	HMDB0128635	3,4,5-trihydroxy-6-{[3-(hydroxymethyl)-1-oxo-1H-isoch	C16H16O10	367.0682889	Yes
6478	HMDB0128637	3,4,5-trihydroxy-6-{[3-(hydroxymethyl)-1-oxo-1H-isoch	C16H16O10	367.0682889	Yes
6478	HMDB0129409	3,4,5-trihydroxy-6-(4-methoxy-1-benzofuran-5-carbon	C16H16O10	367.0682889	Yes
6478	HMDB0132250	3,4,5-trihydroxy-6-[(6-methoxy-2-oxo-2H-chromen-7-y	C16H16O10	367.0682889	Yes
6478	HMDB0136768	3,4,5-trihydroxy-6-[(6-hydroxy-4-methyl-2-oxo-2H-chr	C16H16O10	367.0682889	Yes
6478	HMDB0136769	3,4,5-trihydroxy-6-{[4-(hydroxymethyl)-2-oxo-2H-chro	C16H16O10	367.0682889	Yes
6478	HMDB0136772	3,4,5-trihydroxy-6-[(8-hydroxy-4-methyl-2-oxo-2H-chr	C16H16O10	367.0682889	Yes
6478	HMDB0136774	3,4,5-trihydroxy-6-[(5-hydroxy-4-methyl-2-oxo-2H-chr	C16H16O10	367.0682889	Yes
6479	HMDB0030669	3-O-Feruloylquinic acid	C17H20O9	367.104789	No
6479	HMDB0039959	3-O-Caffeoyl-1-O-methylquinic acid	C17H20O9	367.104789	No
6479	HMDB0039960	3-O-Caffeoyl-4-O-methylquinic acid	C17H20O9	367.104789	No
6479	HMDB0126418	3,4,5-trihydroxy-6-[(5-hydroxy-2,2-dimethyl-2H-chrom	C17H20O9	367.104789	No
6479	HMDB0126419	3,4,5-trihydroxy-6-[(7-hydroxy-2,2-dimethyl-2H-chrom	C17H20O9	367.104789	No
6479	HMDB0126420	3,4,5-trihydroxy-6-[(5-hydroxy-2,2-dimethyl-2H-chrom	C17H20O9	367.104789	No
6479	HMDB0126421	3,4,5-trihydroxy-6-[(6-hydroxy-2,2-dimethyl-2H-chrom	C17H20O9	367.104789	No
6479	HMDB0126422	6-({5,5-dimethyl-3,6-dioxatricyclo[5.4.0.0²,?]undeca-1(	C17H20O9	367.104789	No
6479	HMDB0126423	3,4,5-trihydroxy-6-{[2-(hydroxymethyl)-2-methyl-2H-cl	C17H20O9	367.104789	No
6479	HMDB0127742	3,4,5-trihydroxy-6-{3-[(5-oxooxolan-2-yl)methyl]pheno	C17H20O9	367.104789	No
6479	HMDB0129399	3,4,5-trihydroxy-6-[1-(4-methoxy-1-benzofuran-5-yl)et	C17H20O9	367.104789	No
6479	HMDB0135673	3,4,5-trihydroxy-6-[2-methoxy-4-(3-oxobut-1-en-1-yl)p	C17H20O9	367.104789	No
6481	HMDB0013919	N-Desmethyleletriptan	C21H24N2O2S	367.1477591	No
6482	HMDB0038815	2'',4-Dihydroxy-4'',6''-dimethoxy-3''-prenylchalcone	C22H24O5	367.1543591	No
6482	HMDB0039057	9-Hydroxy-4-(3,7-dimethyl-2,6-octadienyloxy)-psoraler	C22H24O5	367.1543591	No
6482	HMDB0041193	Kanzonol P	C22H24O5	367.1543591	No
6482	HMDB0125890	1-(4-hydroxy-3-methoxyphenyl)-3-(5-methoxy-2,2-dim	C22H24O5	367.1543591	No
6483	HMDB0001032; C04555; CHEBI:16814	Dehydroepiandrosterone sulfate	C19H28O5S	367.1598246	No
6483	HMDB0002833; CHEBI:84094	Testosterone sulfate	C19H28O5S	367.1598246	No
6483	HMDB0013230	Epitestosterone sulfate	C19H28O5S	367.1598246	No
6483	HMDB0041255	(2S)-2-Butanol O-[b-D-Apiofuranosyl-(1->6)-b-D-glucop	C15H28O10	367.1598246	No
6485	HMDB0001908	19-Hydroxy-PGE2	C20H32O6	367.2131844	Yes
6485	HMDB0001979; CHEBI:72595	6,15-Diketo,13,14-dihydro-PGF1a	C20H32O6	367.2131844	Yes
6485	HMDB0003235; C05956; CHEBI:27647	Prostaglandin G2	C20H32O6	367.2131844	Yes
6485	HMDB0003247; CHEBI:137370	20-Hydroxy-PGE2	C20H32O6	367.2131844	Yes
6485	HMDB0004241; C05962; CHEBI:28269	6-Ketoprostaglandin E1	C20H32O6	367.2131844	Yes
6485	HMDB0004242; C05964; CHEBI:28667	11-Dehydro-thromboxane B2	C20H32O6	367.2131844	Yes
6485	HMDB0005099	Thromboxane B3	C20H32O6	367.2131844	Yes
6485	HMDB0012110; C04741	5(6)-Epoxy Prostaglandin E1	C20H32O6	367.2131844	Yes
6485	HMDB0012633	20-COOH-10,11-dihydro-LTB4	C20H32O6	367.2131844	Yes
6485	HMDB0012635	20-dihydroxyleukotriene B4	C20H32O6	367.2131844	Yes
6485	HMDB0034678	Cinncassiol D2	C20H32O6	367.2131844	Yes

6485	HMDB0036854	Cinncassiol D3	C20H32O6	367.2131844	Yes
6485	HMDB0062748; CHEBI:90976	20-hydroxylipoxin A4	C20H32O6	367.2131844	Yes
6489	HMDB0003180; C05482; CHEBI:3898	Cortol	C21H36O5	367.2468293	No
6489	HMDB0005821	Beta-Cortol	C21H36O5	367.2468293	No
6489	HMDB0037961	gamma-Eudesmol rhamnoside	C21H36O5	367.2468293	No
6491	HMDB0036013	(R)-3,4-Dihydro-2-(4,8,12-trimethyl-3,7,11-tridecatrien	C25H36O2	367.2640165	No
6491	HMDB0036054	12''-Apo-b-carotene-3,12''-diol	C25H36O2	367.2640165	No
6491	HMDB0039761	1,1''-[1,13-Tridecanediylbis(oxy)]bisbenzene	C25H36O2	367.2640165	No
6492	HMDB0038073	Octadecyl fumarate	C22H40O4	367.2859783	No
6493	HMDB0002003; C08320; CHEBI:28866	Tetracosanoic acid	C24H48O2	367.3581025	No
6493	HMDB0035633	5-Hydroxy-7-tetracosanone	C24H48O2	367.3581025	No
6493	HMDB0035636	4-Hydroxy-6-tetracosanone	C24H48O2	367.3581025	No
6493	HMDB0040887	20-Tetracosene-1,18-diol	C24H48O2	367.3581025	No
6512	HMDB0015633; CHEBI:64045	Amisulpride	C17H27N3O4S	368.162872	Yes
6514	HMDB0015092; CHEBI:50815	Norgestimate	C23H31NO3	368.2224113	Yes
6545	HMDB0127729	[2-hydroxy-5-(3,5,7-trihydroxy-3,4-dihydro-2H-1-benzo	C15H14O9S	369.0296929	Yes
6545	HMDB0130010	{4-[1-oxo-1-(2,3,4,6-tetrahydroxyphenyl)propan-2-yl]p	C15H14O9S	369.0296929	Yes
6545	HMDB0130254	{4-[3-oxo-3-(2,3,4,6-tetrahydroxyphenyl)propyl]phenyl	C15H14O9S	369.0296929	Yes
6545	HMDB0133576	{4-[(2,3,4,6-tetrahydroxy-2,3-dihydro-1-benzofuran-2-	C15H14O9S	369.0296929	Yes
6545	HMDB0133577	{2,4,6-trihydroxy-2-[(4-hydroxyphenyl)methyl]-2,3-dih	C15H14O9S	369.0296929	Yes
6545	HMDB0134831	{4-[(1E)-3-hydroxy-3-(2,3,4,6-tetrahydroxyphenyl)prop	C15H14O9S	369.0296929	Yes
6545	HMDB0134832	{[(2E)-3-(4-hydroxyphenyl)-1-(2,3,4,6-tetrahydroxyphe	C15H14O9S	369.0296929	Yes
6545	HMDB0135523	[4-(3,4,5,7-tetrahydroxy-3,4-dihydro-2H-1-benzopyran	C15H14O9S	369.0296929	Yes
6545	HMDB0135524	[3,5,7-trihydroxy-2-(4-hydroxyphenyl)-3,4-dihydro-2H-	C15H14O9S	369.0296929	Yes
6545	HMDB0135525	[4,5,7-trihydroxy-2-(4-hydroxyphenyl)-3,4-dihydro-2H-	C15H14O9S	369.0296929	Yes
6546	HMDB0037371	Suillusin	C19H14O8	369.062993	Yes
6546	HMDB0127782	{[1-(3-methoxyphenyl)-3-(2,4,6-trihydroxyphenyl)prop	C16H18O8S	369.062993	Yes
6546	HMDB0128900	15,17-dihydroxy-11,18-dimethoxy-6,8,20-trioxapentac	C19H14O8	369.062993	Yes
6546	HMDB0128901	15,18-dihydroxy-11,17-dimethoxy-6,8,20-trioxapentac	C19H14O8	369.062993	Yes
6546	HMDB0128902	11,15-dihydroxy-17,18-dimethoxy-6,8,20-trioxapentac	C19H14O8	369.062993	Yes
6547	HMDB0039774	5-Hydroxy-6-methoxycoumarin 7-glucoside	C16H18O10	369.0832614	No
6547	HMDB0041733	Ferulic acid 4-O-glucuronide	C16H18O10	369.0832614	No
6547	HMDB0041734	Feruloyl C1-glucuronide	C16H18O10	369.0832614	No
6547	HMDB0041747	Isoferulic acid 3-O-glucuronide	C16H18O10	369.0832614	No
6547	HMDB0041749	Isoferuloyl C1-glucuronide	C16H18O10	369.0832614	No
6547	HMDB0127515	3,4,5-trihydroxy-6-{[3-(4-methoxyphenyl)-2-oxopropar	C16H18O10	369.0832614	No
6547	HMDB0127953	6-[4-(2-carboxyeth-1-en-1-yl)-2-methoxyphenoxy]-3,4,	C16H18O10	369.0832614	No
6547	HMDB0127954	3,4,5-trihydroxy-6-{[3-(4-hydroxy-3-methoxyphenyl)pr	C16H18O10	369.0832614	No
6547	HMDB0127997	6-[3-(2-carboxyeth-1-en-1-yl)-5-methoxyphenoxy]-3,4,	C16H18O10	369.0832614	No
6547	HMDB0127998	3,4,5-trihydroxy-6-{[3-(3-hydroxy-5-methoxyphenyl)pr	C16H18O10	369.0832614	No
6547	HMDB0133307	6-{4-[(1E)-2-carboxyeth-1-en-1-yl]-2-methoxyphenoxy]	C16H18O10	369.0832614	No
6547	HMDB0133308	3,4,5-trihydroxy-6-{[(2E)-3-(4-hydroxy-3-methoxyphen	C16H18O10	369.0832614	No
6547	HMDB0135747	6-[2-(2-carboxyeth-1-en-1-yl)-5-methoxyphenoxy]-3,4,	C16H18O10	369.0832614	No
6547	HMDB0135748	3,4,5-trihydroxy-6-{[3-(2-hydroxy-4-methoxyphenyl)pr	C16H18O10	369.0832614	No
6548	HMDB0040865	Linusitamarin	C17H22O9	369.1190431	Yes
6548	HMDB0041115	Perilloside E	C17H22O9	369.1190431	Yes
6548	HMDB0059980; CHEBI:88704	4-Hydroxy-5-(phenyl)-valeric acid-O-glucuronide	C17H22O9	369.1190431	Yes
6548	HMDB0126241	3,4,5-trihydroxy-6-[3-hydroxy-2-(4-hydroxy-3-methylb	C17H22O9	369.1190431	Yes
6548	HMDB0126243	6-[2,4-dihydroxy-3-(3-methylbut-2-en-1-yl)phenoxy]-3,	C17H22O9	369.1190431	Yes
6548	HMDB0126244	6-[3,6-dihydroxy-2-(3-methylbut-2-en-1-yl)phenoxy]-3,	C17H22O9	369.1190431	Yes
6548	HMDB0126245	6-[3,4-dihydroxy-2-(3-methylbut-2-en-1-yl)phenoxy]-3,	C17H22O9	369.1190431	Yes
6548	HMDB0126247	6-{2-[(3,3-dimethyloxiran-2-yl)methyl]-3-hydroxyphen	C17H22O9	369.1190431	Yes
6548	HMDB0126372	3,4,5-trihydroxy-6-{3-hydroxy-2-[(2E)-4-hydroxy-3-met	C17H22O9	369.1190431	Yes
6548	HMDB0133251	3,4,5-trihydroxy-6-[5-hydroxy-2-(4-hydroxy-3-methylb	C17H22O9	369.1190431	Yes
6548	HMDB0133252	3,4,5-trihydroxy-6-{3-hydroxy-4-[(2E)-4-hydroxy-3-met	C17H22O9	369.1190431	Yes
6548	HMDB0133254	3,4,5-trihydroxy-6-[3-hydroxy-4-(4-hydroxy-3-methylb	C17H22O9	369.1190431	Yes
6548	HMDB0133256	6-[2,6-dihydroxy-3-(3-methylbut-2-en-1-yl)phenoxy]-3,	C17H22O9	369.1190431	Yes
6548	HMDB0133257	6-[2,3-dihydroxy-6-(3-methylbut-2-en-1-yl)phenoxy]-3,	C17H22O9	369.1190431	Yes
6548	HMDB0133258	6-[2,3-dihydroxy-4-(3-methylbut-2-en-1-yl)phenoxy]-3,	C17H22O9	369.1190431	Yes
6548	HMDB0133260	6-[2,4-dihydroxy-5-(3-methylbut-2-en-1-yl)phenoxy]-3,	C17H22O9	369.1190431	Yes
6548	HMDB0133261	6-[4,5-dihydroxy-2-(3-methylbut-2-en-1-yl)phenoxy]-3,	C17H22O9	369.1190431	Yes

6548	HMDB0133264	6-{2-[(3,3-dimethyloxiran-2-yl)methyl]-5-hydroxypheno	C17H22O9	369.1190431	Yes
6548	HMDB0133265	6-{4-[(3,3-dimethyloxiran-2-yl)methyl]-3-hydroxypheno	C17H22O9	369.1190431	Yes
6548	HMDB0135675	3,4,5-trihydroxy-6-[2-methoxy-4-(3-oxobutyl)phenoxy]	C17H22O9	369.1190431	Yes
6548	HMDB0135697	3,4,5-trihydroxy-6-[4-(3-hydroxybut-1-en-1-yl)-2-meth	C17H22O9	369.1190431	Yes
6549	HMDB0133009	3,4,5-trihydroxy-6-{[1-(4-methoxyphenyl)pentan-3-yl]c	C18H26O8	369.1557729	Yes
6551	HMDB0002759; CHEBI:83037	Androsterone sulfate	C19H30O5S	369.1742832	No
6551	HMDB0006278; CHEBI:138026	5a-Dihydrotestosterone sulfate	C19H30O5S	369.1742832	No
6551	HMDB0013232	Etiocholanolone sulfate	C19H30O5S	369.1742832	No
6551	HMDB0062657; CHEBI:83040	Epiandrosterone Sulfate	C19H30O5S	369.1742832	No
6553	HMDB0032841	5-Megastigmen-7-yne-3,9-diol 9-glucoside	C19H30O7	369.1922831	No
6553	HMDB0032842	5-Megastigmen-7-yne-3,9-diol 3-glucoside	C19H30O7	369.1922831	No
6553	HMDB0036822	(3S,7E,9S)-9-Hydroxy-4,7-megastigmadien-3-one 9-glu	C19H30O7	369.1922831	No
6555	HMDB0094680; CHEBI:44794	Octaethylene glycol	C16H34O9	369.2132866	Yes
6556	HMDB0002886; C05961; CHEBI:28158	6-Keto-prostaglandin F1a	C20H34O6	369.2288536	Yes
6556	HMDB0003252; C05963; CHEBI:28728	Thromboxane B2	C20H34O6	369.2288536	Yes
6556	HMDB0004049	20-Hydroxy-PGF2a	C20H34O6	369.2288536	Yes
6556	HMDB0012502	10,11-dihydro-20-dihydroxy-LTB4	C20H34O6	369.2288536	Yes
6556	HMDB0013039	Prostaglandin G1	C20H34O6	369.2288536	Yes
6558	HMDB0040270; C14240; CHEBI:34675	Diethylhexyl adipate	C22H42O4	369.3012052	No
6558	HMDB0041619	Dioctyl hexanedioate	C22H42O4	369.3012052	No
6571	HMDB0240252; CHEBI:133026	Salicyluric beta-D-glucuronide	C15H17NO10	370.0771538	No
6574	HMDB0014813; C07108; CHEBI:41774	Tamoxifen	C26H29NO	370.2168378	No
6589	HMDB0031592	Brevifolincarboxylic acid 9-sulfate	C13H8O11S	370.9698768	No
6595	HMDB0031756	Veranisatin C	C16H20O10	371.0985254	No
6595	HMDB0041723	Dihydroferulic acid 4-O-glucuronide	C16H20O10	371.0985254	No
6595	HMDB0127981	6-[4-(2-carboxyethyl)-2-methoxyphenoxy]-3,4,5-trihyd	C16H20O10	371.0985254	No
6595	HMDB0127982	3,4,5-trihydroxy-6-{[3-(4-hydroxy-3-methoxyphenyl)pr	C16H20O10	371.0985254	No
6595	HMDB0128015	6-[3-(2-carboxyethyl)-5-methoxyphenoxy]-3,4,5-trihyd	C16H20O10	371.0985254	No
6595	HMDB0128016	3,4,5-trihydroxy-6-{[3-(3-hydroxy-5-methoxyphenyl)pr	C16H20O10	371.0985254	No
6595	HMDB0133870	6-[2-(1-carboxyethyl)-5-methoxyphenoxy]-3,4,5-trihyd	C16H20O10	371.0985254	No
6595	HMDB0133871	3,4,5-trihydroxy-6-{[2-(2-hydroxy-4-methoxyphenyl)pr	C16H20O10	371.0985254	No
6599	HMDB0039234	Citrusin E	C17H24O9	371.1354072	Yes
6599	HMDB0135716	3,4,5-trihydroxy-6-[4-(3-hydroxybutyl)-2-methoxypher	C17H24O9	371.1354072	Yes
6604	HMDB0040668	Blumenol C glucoside	C19H32O7	371.2076173	Yes
6604	HMDB0040701	9-Hydroxy-7-megastigmen-3-one glucoside	C19H32O7	371.2076173	Yes
6606	HMDB0014550; CHEBI:604234	Gentian Violet	C25H30N3	371.2357174	No
6652	HMDB0029538; CHEBI:506254	Neodiospyrin	C22H14O6	373.072791	Yes
6652	HMDB0129030	{4-[(E)-2-{3,5-dihydroxy-4-[(1E)-3-methylbuta-1,3-dien	C19H18O6S	373.072791	Yes
6653	HMDB0034942; C11673	Geniposidic acid	C16H22O10	373.1144482	Yes
6655	HMDB0030164	Cyclonormammein	C21H26O6	373.1646974	No
6655	HMDB0030649	Mammea B/BC cyclo E	C21H26O6	373.1646974	No
6655	HMDB0030650; CHEBI:554198	8,9-Dihydro-5-hydroxy-8-(1-hydroxy-1-methylethyl)-6-	C21H26O6	373.1646974	No
6655	HMDB0033534	Fragransin C1	C21H26O6	373.1646974	No
6655	HMDB0033537	Fragransol A	C21H26O6	373.1646974	No
6655	HMDB0036463	1-(4-Hydroxy-3,5-dimethoxyphenyl)-2-[2-methoxy-4-(1	C21H26O6	373.1646974	No
6655	HMDB0039248	2-(4-Allyl-2,6-dimethoxyphenoxy)-1-(4-hydroxy-3-metl	C21H26O6	373.1646974	No
6655	HMDB0041139	Sonchifolin	C21H26O6	373.1646974	No
6657	HMDB0029782	6-Epi-7-isocucurbic acid glucoside	C18H30O8	373.1873669	Yes
6659	HMDB0040824	Tsangane L 3-glucoside	C19H34O7	373.2233398	No
6661	HMDB0061030	omega-hydroxyfinasteride	C22H34N2O3	373.2506114	No
6663	HMDB0000308; CHEBI:384794	3b-Hydroxy-5-cholenoic acid	C24H38O3	373.2748574	No
6663	HMDB0038522	2-(10-Heptadecenyl)-6-hydroxybenzoic acid	C24H38O3	373.2748574	No
6663	HMDB0041453	D8''-Merulinic acid C	C24H38O3	373.2748574	No
6692	HMDB0033300	(1RS,2RS)-Guaiacylglycerol 1-glucoside	C16H24O10	375.1281801	Yes
6692	HMDB0033301	(1x,2x)-Guaiacylglycerol 2-glucoside	C16H24O10	375.1281801	Yes
6692	HMDB0040600	(1x,2x)-Guaiacylglycerol 3-glucoside	C16H24O10	375.1281801	Yes
6693	HMDB0038971	Diacetylfusarochromanone	C19H24N2O6	375.1548151	Yes
6695	HMDB0000332	18-Oxocortisol	C21H28O6	375.1811852	Yes
6695	HMDB0031932	11,12,14-Trihydroxy-7-methoxy-8,11,13-abietatrien-2(	C21H28O6	375.1811852	Yes
6695	HMDB0039557	12alpha-Hydroxy-13,18-dehydroparain	C21H28O6	375.1811852	Yes
6695	HMDB0061016	16α-hydroxyprednisolone	C21H28O6	375.1811852	Yes

6696	HMDB0014722; C06977; CHEBI:4784	Enalapril	C20H28N2O5	375.1925455	No
6696	HMDB0015036; C08021; CHEBI:8802	Remifentanil	C20H28N2O5	375.1925455	No
6701	HMDB0000381	Allolithocholic acid	C24H40O3	375.290574	No
6701	HMDB0000713	Isoallolithocholic acid	C24H40O3	375.290574	No
6701	HMDB0000717; C17658	Isolithocholic acid	C24H40O3	375.290574	No
6701	HMDB0000761; C03990; CHEBI:16325	Lithocholic acid	C24H40O3	375.290574	No
6701	HMDB0002431	12b-Hydroxy-5b-cholanoic acid	C24H40O3	375.290574	No
6701	HMDB0002492; CHEBI:542339	7a-Hydroxy-5b-cholanic acid	C24H40O3	375.290574	No
6746	HMDB0129424	3,4,5-trihydroxy-6-({7-oxo-7H-furo[3,2-g]chromen-4-yl	C17H14O10	377.0511988	No
6749	HMDB0029304; CHEBI:89421	3,4-DHPEA-EA	C19H22O8	377.1248589	No
6749	HMDB0035037	Gibberellin A32	C19H22O8	377.1248589	No
6749	HMDB0039137	2-(3,4-Dihydroxyphenylethyl)-6-epi-elenaiate	C19H22O8	377.1248589	No
6750	HMDB0011739	Galactinol dihydrate	C12H26O13	377.132559	No
6751	HMDB0030656; CHEBI:657230	Demethylcalabaxanthone	C23H22O5	377.136959	No
6751	HMDB0040350	6,11-Dihydroxy-3-methyl-3-(4-methyl-3-pentenyl)-3H,7	C23H22O5	377.136959	No
6753	HMDB0030657	Carinol	C20H26O7	377.160949	Yes
6753	HMDB0034474; C09517	Niveusin C	C20H26O7	377.160949	Yes
6753	HMDB0035053	Gibberellin A52	C20H26O7	377.160949	Yes
6753	HMDB0036694	15-Hydroxyleptocarpin	C20H26O7	377.160949	Yes
6753	HMDB0036897; CHEBI:24236	Gibberellin A17	C20H26O7	377.160949	Yes
6753	HMDB0036900	Gibberellin A66	C20H26O7	377.160949	Yes
6753	HMDB0036945	Gibberellin A125	C20H26O7	377.160949	Yes
6753	HMDB0041002	(1E,4Z,6a,8b,10a)-8-(2-Methylbutanoyloxy)-10,15-dihy	C20H26O7	377.160949	Yes
6753	HMDB0041231	Gibberellin A99	C20H26O7	377.160949	Yes
6753	HMDB0041349	Gibberellin A102	C20H26O7	377.160949	Yes
6757	HMDB0000418	18-Hydroxycortisol	C21H30O6	377.1971195	Yes
6757	HMDB0030045	Isohumulinone A	C21H30O6	377.1971195	Yes
6757	HMDB0030105	Humulinone	C21H30O6	377.1971195	Yes
6757	HMDB0032770	Eremopetasitenin C1	C21H30O6	377.1971195	Yes
6757	HMDB0032773	Eremopetasitenin D1	C21H30O6	377.1971195	Yes
6757	HMDB0036889	11-Dihydro-12-norneoquassin	C21H30O6	377.1971195	Yes
6757	HMDB0040702	Sugetriol triacetate	C21H30O6	377.1971195	Yes
6757	HMDB0061033	6-beta-hydrocortisol	C21H30O6	377.1971195	Yes
6759	HMDB0035508	Annoglabasin F	C22H34O5	377.2334691	No
6777	HMDB0060415; C16619; CHEBI:1267082	6-Thioguanosine monophosphate	C10H14N5O7PS	378.0265507	No
6777	HMDB0060789	6-Thioguanylic acid	C10H14N5O7PS	378.0265507	No
6779	HMDB0033513	Gonyautoxin V	C10H17N7O7S	378.0815508	Yes
6783	HMDB0039505	2-(Arabinosylamino)-3-(glucosylamino)propanenitrile	C14H25N3O9	378.1494309	Yes
6788	HMDB0032529	Thaumatin b, recombinant	C19H29N3O3S	378.185731	No
6814	HMDB0036893	Gibberellin A75	C19H24O8	379.1403938	No
6814	HMDB0037056	8-Oxodiacetoxyscirpenol	C19H24O8	379.1403938	No
6814	HMDB0038328	(S)-Bitalin A 12-glucoside	C19H24O8	379.1403938	No
6814	HMDB0038450	Gibberellin A86	C19H24O8	379.1403938	No
6814	HMDB0040899	Methyl helianthenoate A glucoside	C19H24O8	379.1403938	No
6815	HMDB0031954	3-Methyl-3-butenyl apiosyl-(1->6)-glucoside	C16H28O10	379.1614598	Yes
6815	HMDB0031956	Prenyl apiosyl-(1->6)-glucoside	C16H28O10	379.1614598	Yes
6815	HMDB0041360	Prenyl arabinosyl-(1->6)-glucoside	C16H28O10	379.1614598	Yes
6816	HMDB0030760	Pteroside B	C20H28O7	379.1763729	No
6816	HMDB0035166	Cinncassiol C	C20H28O7	379.1763729	No
6816	HMDB0035840	Deacetylisovaltrate	C20H28O7	379.1763729	No
6816	HMDB0041228	6b-Angeloyl-3b,8b,9b-trihydroxy-7(11)-eremophilen-1	C20H28O7	379.1763729	No
6816	HMDB0126521	3,4,5-trihydroxy-6-{[(2Z)-2-(phenylmethylidene)heptyl	C20H28O7	379.1763729	No
6817	HMDB0030760	Pteroside B	C20H28O7	379.1783907	Yes
6817	HMDB0035166	Cinncassiol C	C20H28O7	379.1783907	Yes
6817	HMDB0035840	Deacetylisovaltrate	C20H28O7	379.1783907	Yes
6817	HMDB0041228	6b-Angeloyl-3b,8b,9b-trihydroxy-7(11)-eremophilen-1	C20H28O7	379.1783907	Yes
6817	HMDB0126521	3,4,5-trihydroxy-6-{[(2Z)-2-(phenylmethylidene)heptyl	C20H28O7	379.1783907	Yes
6820	HMDB0040568	[6]-Gingerdiol 3,5-diacetate	C21H32O6	379.2125663	Yes
6823	HMDB0002082	Bisnorcholic acid	C22H36O5	379.250223	No
6836	HMDB0037269	Desacetycefapirin	C15H15N3O5S2	380.0377145	No
6855	HMDB0128640	7-[(6-carboxy-3,4,5-trihydroxyoxan-2-yl)oxy]-1-oxo-1H	C16H14O11	381.0488364	Yes

6855	HMDB0128641	3,4,5-trihydroxy-6-(7-hydroxy-1-oxo-1H-isochromene-:	C16H14O11	381.0488364	Yes
6855	HMDB0128643	6-[(6-carboxy-3,4,5-trihydroxyoxan-2-yl)oxy]-1-oxo-1H	C16H14O11	381.0488364	Yes
6855	HMDB0128644	3,4,5-trihydroxy-6-(6-hydroxy-1-oxo-1H-isochromene-:	C16H14O11	381.0488364	Yes
6855	HMDB0128646	5-[(6-carboxy-3,4,5-trihydroxyoxan-2-yl)oxy]-1-oxo-1H	C16H14O11	381.0488364	Yes
6855	HMDB0128647	3,4,5-trihydroxy-6-(5-hydroxy-1-oxo-1H-isochromene-:	C16H14O11	381.0488364	Yes
6857	HMDB0000961; C00448; CHEBI:17407	Farnesyl pyrophosphate	C15H28O7P2	381.1221366	Yes
6858	HMDB0010343; C03033	1-(alpha-Methyl-4-(2-methylpropyl)benzeneacetate)-b	C19H26O8	381.1561546	No
6858	HMDB0035201	4,8-Diacetyl-T2-tetrol	C19H26O8	381.1561546	No
6858	HMDB0038916	7alpha,8alpha-Dihydroxycalonectrin	C19H26O8	381.1561546	No
6858	HMDB0039811	Cyclocalopin B	C19H26O8	381.1561546	No
6858	HMDB0060919	Ibuprofen glucuronide	C19H26O8	381.1561546	No
6858	HMDB0132958	3,4,5-trihydroxy-6-{[1-(4-methoxyphenyl)-4-methylper	C19H26O8	381.1561546	No
6858	HMDB0140970	3,4,5-trihydroxy-6-({2-[4-(2-methylpropyl)phenyl]prop	C19H26O8	381.1561546	No
6859	HMDB0012553	12-Oxo-20-trihydroxy-leukotriene B4	C20H30O7	381.1926179	No
6859	HMDB0035164	Cinncassiol A	C20H30O7	381.1926179	No
6859	HMDB0035396	T2 Triol	C20H30O7	381.1926179	No
6859	HMDB0036859	Cinncassiol C3	C20H30O7	381.1926179	No
6862	HMDB0034953	Apo-12''-violaxanthal	C25H34O3	381.2460672	No
6867	HMDB0002361; CHEBI:39420	Pentacosanoic acid	C25H50O2	381.3731891	No
6867	HMDB0035629	6-Hydroxy-8-pentacosanone	C25H50O2	381.3731891	No
6867	HMDB0035630	4-Hydroxy-6-pentacosanone	C25H50O2	381.3731891	No
6867	HMDB0035632	6-Hydroxy-4-pentacosanone	C25H50O2	381.3731891	No
6867	HMDB0036843	Lepidiumsesterterpenol	C25H50O2	381.3731891	No
6876	HMDB0015427; C06890; CHEBI:553473	Ceftizoxime	C13H13N5O5S2	382.0301483	Yes
6877	HMDB0015325; C07760; CHEBI:3176	Brinzolamide	C12H21N3O5S3	382.0581483	Yes
6879	HMDB0000912; CHEBI:71169	Succinyladenosine	C14H17N5O8	382.0997484	No
6881	HMDB0001542; C00611; CHEBI:16153	N-Acetyllactosamine	C14H25NO11	382.1362485	No
6881	HMDB0006535	Beta-1,4-mannose-N-acetylglucosamine	C14H25NO11	382.1362485	No
6881	HMDB0006575; C06372; CHEBI:27707	Lacto-N-biose I	C14H25NO11	382.1362485	No
6881	HMDB0006583	Poly-N-acetyllactosamine	C14H25NO11	382.1362485	No
6882	HMDB0005021; C07397; CHEBI:8707	Quetiapine	C21H25N3O2S	382.1594149	No
6897	HMDB0000386	3b,16a-Dihydroxyandrostenone sulfate	C19H28O6S	383.1537804	Yes
6897	HMDB0033063	2,3-Butanediol apiosylglucoside	C15H28O11	383.1537804	Yes
6897	HMDB0062611; CHEBI:87774	16alpha-hydroxydehydroepiandrosterone 3-sulfate	C19H28O6S	383.1537804	Yes
6901	HMDB0011543	MG(0:0/20:1(11Z)/0:0)	C23H44O4	383.3168856	No
6901	HMDB0011573	MG(20:1(11Z)/0:0/0:0)	C23H44O4	383.3168856	No
6901	HMDB0036344	Tetrahydropersin	C23H44O4	383.3168856	No
6902	HMDB0039540; C17873; CHEBI:61302	Cerebronic acid	C24H48O3	383.3529976	No
6912	HMDB0014291; CHEBI:18303	Phosphatidylserine	C13H24NO10P	384.1088122	Yes
6928	HMDB0012503	10,11-dihydro-20-trihydroxy-leukotriene B4	C20H34O7	385.2237129	Yes
6943	HMDB0001245; C00705; CHEBI:28846	dCDP	C9H15N3O10P2	386.0134057	No
6946	HMDB0031401	Dihydroxycitracridone I	C20H21NO7	386.124287	No
6969	HMDB0039964	beta-D-Glucopyranosyl-11-hydroxyjasmonic acid	C18H28O9	387.1669432	Yes
6969	HMDB0040706	7-Epi-12-hydroxyjasmonic acid glucoside	C18H28O9	387.1669432	Yes
6969	HMDB0041552	2-[4-(3-Hydroxypropyl)-2-methoxyphenoxy]-1,3-propa	C18H28O9	387.1669432	Yes
6973	HMDB0031676	5a,6a-Epoxy-7E-megastigmene-3a,9e-diol 3-glucoside	C19H32O8	387.2027208	Yes
6973	HMDB0036318	9,13-Dihydroxy-4-megastigmen-3-one 9-glucoside	C19H32O8	387.2027208	Yes
6973	HMDB0036846	Icariside B8	C19H32O8	387.2027208	Yes
6973	HMDB0038306	5a,6a-Epoxy-7E-megastigmene-3b,9e-diol 9-glucoside	C19H32O8	387.2027208	Yes
6973	HMDB0040614	Dihydroroseoside	C19H32O8	387.2027208	Yes
6975	HMDB0012109; C06475	5,6-Dihydroxyprostaglandin F1a	C20H36O7	387.2390476	Yes
6993	HMDB0034071; C08425; CHEBI:8454	Progoitrin	C11H19NO10S2	388.0352494	No
7000	HMDB0030381	Roquefortine	C22H23N5O2	388.1791497	Yes
7000	HMDB0135477	3-(3,4-dimethoxyphenyl)-N-[2-(3,4-dimethoxyphenyl)e	C21H27NO6	388.1791497	Yes
7000	HMDB0135478	3-(3,4-dimethoxyphenyl)-N-[2-(3,4-dimethoxyphenyl)-:	C21H27NO6	388.1791497	Yes
7000	HMDB0135479	3-(3,4-dimethoxyphenyl)-N-[2-(3,4-dimethoxyphenyl)e	C21H27NO6	388.1791497	Yes
7023	HMDB0034223	2,3,4,5,6-Penta-O-acetyl-D-glucose	C16H22O11	389.1091495	Yes
7023	HMDB0035608; C09788	Monotropein	C16H22O11	389.1091495	Yes
7025	HMDB0029094	Tryptophyl-Tryptophan	C22H22N4O3	389.1644214	Yes
7030	HMDB0041176	(3S,5R,6S,7E,9x)-7-Megastigmene-3,6,9-triol 9-glucosic	C19H34O8	389.2184109	Yes
7030	HMDB0041578	7,8-Dihydro-3b,6a-dihydroxy-alpha-ionol 9-glucoside	C19H34O8	389.2184109	Yes

7033	HMDB0000328; CHEBI:803536	12-Ketodeoxycholic acid	C24H38O4	389.2685023	No
7033	HMDB0000460	7-Hydroxy-3-oxocholanoic acid	C24H38O4	389.2685023	No
7033	HMDB0000467	Nutriacholic acid	C24H38O4	389.2685023	No
7033	HMDB0000503	7a-Hydroxy-3-oxo-5b-cholanoic acid	C24H38O4	389.2685023	No
7033	HMDB0012866	9''-Carboxy-alpha-chromanol	C24H38O4	389.2685023	No
7033	HMDB0041454	D8''-Merulinic acid A	C24H38O4	389.2685023	No
7033	HMDB0062742; CHEBI:88104	12alpha-hydroxy-3-oxo-5beta-cholan-24-oic Acid	C24H38O4	389.2685023	No
7088	HMDB0038066	Garcimangosone D	C19H20O9	391.1040063	No
7088	HMDB0130035	3,4,5-trihydroxy-6-{[(3E)-4-(1-oxo-1H-isochromen-3-yl]	C19H20O9	391.1040063	No
7088	HMDB0130037	6-({3-[(1E)-but-1-en-1-yl]-1-oxo-1H-isochromen-7-yl}o›	C19H20O9	391.1040063	No
7088	HMDB0130038	6-({3-[(1E)-but-1-en-1-yl]-1-oxo-1H-isochromen-6-yl}o›	C19H20O9	391.1040063	No
7088	HMDB0130041	6-({3-[(1E)-but-1-en-1-yl]-1-oxo-1H-isochromen-5-yl}o›	C19H20O9	391.1040063	No
7088	HMDB0130042	3,4,5-trihydroxy-6-{[(3E)-4-(1-oxo-1H-isochromen-3-yl]	C19H20O9	391.1040063	No
7088	HMDB0137957	2-(4-benzoyl-3,5-dihydroxyphenoxy)-6-(hydroxymethy	C19H20O9	391.1040063	No
7089	HMDB0039564	Marmesin rhamnoside	C20H24O8	391.139806	No
7092	HMDB0015608; CHEBI:725589	Indacaterol	C24H28N2O3	391.2032851	No
7092	HMDB0015705; CHEBI:66901	Ivacaftor	C24H28N2O3	391.2032851	No
7095	HMDB0060730	20, 22-Dihydrodigoxigenin	C23H36O5	391.2498752	Yes
7099	HMDB0000348	3b,12a-Dihydroxy-5a-cholanoic acid	C24H40O4	391.2839807	No
7099	HMDB0000361; C17660	3b,7a-Dihydroxy-5b-cholanoic acid	C24H40O4	391.2839807	No
7099	HMDB0000384	3a,7a-Dihydroxycholanoic acid	C24H40O4	391.2839807	No
7099	HMDB0000411; C17646; CHEBI:542045	3a,12b-Dihydroxy-5b-cholanoic acid	C24H40O4	391.2839807	No
7099	HMDB0000438; CHEBI:473574	3b,12a-Dihydroxy-5b-cholanoic acid	C24H40O4	391.2839807	No
7099	HMDB0000478	Allodeoxycholic acid	C24H40O4	391.2839807	No
7099	HMDB0000514	Allochenodeoxycholic acid	C24H40O4	391.2839807	No
7099	HMDB0000518; C02528; CHEBI:16755	Chenodeoxycholic acid	C24H40O4	391.2839807	No
7099	HMDB0000626; C04483; CHEBI:28834	Deoxycholic acid	C24H40O4	391.2839807	No
7099	HMDB0000664	Isohyodeoxycholic acid	C24H40O4	391.2839807	No
7099	HMDB0000686; C17662; CHEBI:43419	Isoursodeoxycholic acid	C24H40O4	391.2839807	No
7099	HMDB0000733	Hyodeoxycholic acid	C24H40O4	391.2839807	No
7099	HMDB0000811; C15515; CHEBI:52030	Murocholic acid	C24H40O4	391.2839807	No
7099	HMDB0000946; C07880; CHEBI:9907	Ursodeoxycholic acid	C24H40O4	391.2839807	No
7099	HMDB0002451	7b,12a-Dihydroxycholanoic acid	C24H40O4	391.2839807	No
7099	HMDB0002488	7a,12b-dihydroxy-5b-Cholan-24-oic acid	C24H40O4	391.2839807	No
7099	HMDB0002536; C17661	Isodeoxycholic acid	C24H40O4	391.2839807	No
7099	HMDB0002585	3b,12b-Dihydroxy-5b-cholanoic acid	C24H40O4	391.2839807	No
7106	HMDB0014421; C01553; CHEBI:2687	Amsacrine	C21H19N3O3S	392.1072028	Yes
7131	HMDB0030817	6-Hydroxymusizin 8-O-b-D-glucopyranoside	C19H22O9	393.1194171	Yes
7131	HMDB0035734	2''-Oxoaloesol 7-glucoside	C19H22O9	393.1194171	Yes
7133	HMDB0012530	11-Hydroxyeicosatetraenoate glyceryl ester	C23H38O5	393.2647908	Yes
7133	HMDB0013651; CHEBI:132121	2-(14,15-Epoxyeicosatrienoyl) Glycerol	C23H38O5	393.2647908	Yes
7136	HMDB0035215	Ximenic acid	C26H50O2	393.3735843	No
7136	HMDB0035549	11,13-Hexacosanedione	C26H50O2	393.3735843	No
7136	HMDB0035550	10,12-Hexacosanedione	C26H50O2	393.3735843	No
7136	HMDB0035551	7,9-Hexacosanedione	C26H50O2	393.3735843	No
7136	HMDB0035552	6,8-Hexacosanedione	C26H50O2	393.3735843	No
7136	HMDB0035553	5,7-Hexacosanedione	C26H50O2	393.3735843	No
7136	HMDB0035554	4,6-Hexacosanedione	C26H50O2	393.3735843	No
7136	HMDB0062452; CHEBI:77525	17Z-hexacosenoic acid	C26H50O2	393.3735843	No
7155	HMDB0034873	Ethyl 2-hydroxy-3-(3-indolyl)propanoate glucoside	C19H25NO8	394.1531489	No
7176	HMDB0039953	1-(3-Methylbutanoyl)-6-apiosylglucose	C16H28O11	395.1561806	No
7178	HMDB0000774	Pregnenolone sulfate	C21H32O5S	395.1909121	Yes
7178	HMDB0031689	1-Hexanol arabinosylglucoside	C17H32O10	395.1909121	Yes
7178	HMDB0060382; CHEBI:35420	3beta-Hydroxypregn-5-en-20-one sulfate	C21H32O5S	395.1909121	Yes
7186	HMDB0002356; CHEBI:31009	Hexacosanoic acid	C26H52O2	395.3890851	No
7186	HMDB0035628	6-Hydroxy-8-hexacosanone	C26H52O2	395.3890851	No
7218	HMDB0029190; CHEBI:89287	5-(3'',4''-dihydroxyphenyl)-gamma-valerolactone-3''-O	C18H22O10	397.1144154	Yes
7218	HMDB0030055	Taraxacoside	C18H22O10	397.1144154	Yes
7218	HMDB0041285	Picraquassioside A	C18H22O10	397.1144154	Yes
7218	HMDB0059988; CHEBI:88694	5-(3'',4''-Dihydroxyphenyl)-gamma-valerolactone-3''-O	C18H22O10	397.1144154	Yes
7218	HMDB0059990; CHEBI:88748	5-(3'',4''-Dihydroxyphenyl)-gamma-valerolactone-4''-O	C18H22O10	397.1144154	Yes

7218	HMDB0060030; CHEBI:89537	5-(3'',5''-Dihydroxyphenyl)-gamma-valerolactone-O-glı	C18H22O10	397.1144154	Yes
7218	HMDB0127768	3,4,5-trihydroxy-6-{2-methoxy-4-[(5-oxooxolan-2-yl)me	C18H22O10	397.1144154	Yes
7218	HMDB0127830	3,4,5-trihydroxy-6-{2-methoxy-5-[(5-oxooxolan-2-yl)me	C18H22O10	397.1144154	Yes
7219	HMDB0040121	Methyl 3,4-dihydroxy-5-prenylbenzoate 3-glucoside	C19H26O9	397.151258	No
7219	HMDB0040896	Methyl (3x,10R)-dihydroxy-11-dodecene-6,8-diynoate	C19H26O9	397.151258	No
7219	HMDB0132960	3,4,5-trihydroxy-6-{[1-(4-methoxyphenyl)-4-methyl-3-(	C19H26O9	397.151258	No
7219	HMDB0132964	3,4,5-trihydroxy-6-{[5-(4-methoxyphenyl)-2-methyl-3-(	C19H26O9	397.151258	No
7219	HMDB0132966	3,4,5-trihydroxy-6-{[5-(4-methoxyphenyl)-2-methyl-3-(	C19H26O9	397.151258	No
7219	HMDB0132967	3,4,5-trihydroxy-6-{[1-(4-methoxyphenyl)-4-methyl-3-(	C19H26O9	397.151258	No
7243	HMDB0031945	Niaziminin A	C18H25NO7S	398.1278872	Yes
7243	HMDB0041970; C11803; CHEBI:7847	Oxmetidine	C19H21N5O3S	398.1278872	Yes
7244	HMDB0015686	Pipazethate	C21H25N3O3S	398.1544559	Yes
7244	HMDB0032156	alpha-Acetolactate decarboxylase (enzyme preparatior	C21H25N3O3S	398.1544559	Yes
7262	HMDB0125816	3,4,5-trihydroxy-6-[(4-hydroxy-7-methoxy-2,2-dimethy	C18H24O10	399.1300722	Yes
7262	HMDB0127783	6-{[4-carboxy-1-(3-methoxyphenyl)butan-2-yl]oxy}-3,4	C18H24O10	399.1300722	Yes
7262	HMDB0127784	3,4,5-trihydroxy-6-{[4-hydroxy-5-(3-methoxyphenyl)pe	C18H24O10	399.1300722	Yes
7262	HMDB0127790	6-[4-(4-carboxybutyl)-2-methoxyphenoxy]-3,4,5-trihyd	C18H24O10	399.1300722	Yes
7262	HMDB0127791	3,4,5-trihydroxy-6-{[5-(4-hydroxy-3-methoxyphenyl)pe	C18H24O10	399.1300722	Yes
7263	HMDB0030975	Corchoionoside B	C19H28O9	399.1661335	No
7264	HMDB0036855	Cincassiol B	C20H32O8	399.2045183	No
7270	HMDB0000501	7-Ketocholesterol	C27H44O2	399.3245535	No
7270	HMDB0001993; C05455; CHEBI:17899	7a-Hydroxy-cholestene-3-one	C27H44O2	399.3245535	No
7270	HMDB0003550; C01561; CHEBI:17933	Calcidiol	C27H44O2	399.3245535	No
7270	HMDB0006721	5,6-trans-25-Hydroxyvitamin D3	C27H44O2	399.3245535	No
7270	HMDB0006722	25-Hydroxytachysterol3	C27H44O2	399.3245535	No
7270	HMDB0015504; CHEBI:1125553	Alfacalcidol	C27H44O2	399.3245535	No
7270	HMDB0034328	(3beta,5alpha,6a)-Cholesta-8,14-diene-3,6-diol	C27H44O2	399.3245535	No
7270	HMDB0035585	1-Phenyl-1,3-heneicosanedione	C27H44O2	399.3245535	No
7270	HMDB0036012	5-Methyl-2,4-bis(3-methyl-2-butenyl)-6-(2-methyl-1-ox	C27H44O2	399.3245535	No
7270	HMDB0039871	5-(12,15-Heneicosadienyl)-1,3-benzenediol	C27H44O2	399.3245535	No
7270	HMDB0060131	3beta-Hydroxy-5-cholestenal	C27H44O2	399.3245535	No
7270	HMDB0060132	27alpha-Hydroxy-8-dehydrocholesterol	C27H44O2	399.3245535	No
7283	HMDB0061107	Buspirone N-oxide	C21H31N5O3	400.2357187	No
7283	HMDB0061108	3''-Hydroxybuspirone	C21H31N5O3	400.2357187	No
7283	HMDB0061109	5-Hydroxybuspirone	C21H31N5O3	400.2357187	No
7283	HMDB0061110	6''-Hydroxybuspirone	C21H31N5O3	400.2357187	No
7297	HMDB0001274; C00363; CHEBI:18075	dTDP	C10H16N2O11P2	401.016152	Yes
7297	HMDB0136103	[2,6-dihydroxy-4-(3,4,5,7-tetrahydroxy-3,4-dihydro-2H	C15H14O11S	401.016152	Yes
7297	HMDB0136104	[4,5,7-trihydroxy-2-(3,4,5-trihydroxyphenyl)-3,4-dihydı	C15H14O11S	401.016152	Yes
7297	HMDB0136105	[3,5,7-trihydroxy-2-(3,4,5-trihydroxyphenyl)-3,4-dihydı	C15H14O11S	401.016152	Yes
7298	HMDB0001117; C04352; CHEBI:15769	4-Phosphopantothenoylcysteine	C12H23N2O9PS	401.0760521	Yes
7299	HMDB0059971; CHEBI:88690	4-Hydroxy-5-(3'',4''-dihydroxyphenyl)-valeric acid-O-glı	C17H22O11	401.1093723	Yes
7299	HMDB0059973; CHEBI:88688	4-Hydroxy-5-(3'',5''-dihydroxyphenyl)-valeric acid-O-glı	C17H22O11	401.1093723	Yes
7299	HMDB0127743	6-[4-(4-carboxy-2-hydroxybutyl)-2-hydroxyphenoxy]-3,	C17H22O11	401.1093723	Yes
7299	HMDB0127744	6-[5-(4-carboxy-2-hydroxybutyl)-2-hydroxyphenoxy]-3,	C17H22O11	401.1093723	Yes
7299	HMDB0127745	6-{[5-(3,4-dihydroxyphenyl)-4-hydroxypentanoyl]oxy}-	C17H22O11	401.1093723	Yes
7299	HMDB0128703	3,4,5-trihydroxy-6-[2-hydroxy-3,4-dimethoxy-5-(3-oxop	C17H22O11	401.1093723	Yes
7299	HMDB0128704	3,4,5-trihydroxy-6-[6-hydroxy-2,3-dimethoxy-4-(3-oxop	C17H22O11	401.1093723	Yes
7299	HMDB0128718	3,4,5-trihydroxy-6-[2-hydroxy-5-(3-hydroxyprop-1-en-1	C17H22O11	401.1093723	Yes
7299	HMDB0128719	3,4,5-trihydroxy-6-[6-hydroxy-4-(3-hydroxyprop-1-en-1	C17H22O11	401.1093723	Yes
7300	HMDB0041190	Benzyl beta-primeveroside	C18H26O10	401.1454923	Yes
7300	HMDB0041514	Benzyl O-[arabinofuranosyl-(1->6)-glucoside]	C18H26O10	401.1454923	Yes
7301	HMDB0031763	Methyl 7-epi-12-hydroxyjasmonate glucoside	C19H30O9	401.1820189	No
7301	HMDB0040729	D-Linalool 3-(6''''-malonylglucoside)	C19H30O9	401.1820189	No
7302	HMDB0015698	Cinitapride	C21H30N4O4	401.2166585	No
7302	HMDB0034159	Acetyl tributyl citrate	C20H34O8	401.2166585	No
7303	HMDB0035844	Lucidone A	C24H34O5	401.2308724	No
7307	HMDB0000541	7b-Hydroxy-3-oxo-5b-cholanoic acid	C25H38O4	401.2700871	No
7307	HMDB0011557	MG(0:0/22:6(4Z,7Z,10Z,13Z,16Z,19Z)/0:0)	C25H38O4	401.2700871	No
7307	HMDB0011587	MG(22:6(4Z,7Z,10Z,13Z,16Z,19Z)/0:0/0:0)	C25H38O4	401.2700871	No
7320	HMDB0037550	2,4-Dihydroxy-7,8-dimethoxy-2H-1,4-benzoxazin-3(4H)	C16H21NO11	402.1042902	No

7341	HMDB0029222; CHEBI:89099	Urolithin A-3-O-glucuronide	C19H16O10	403.0672935	No
7341	HMDB0060022; CHEBI:89554	Urolithin A-8-O-glucuronide	C19H16O10	403.0672935	No
7344	HMDB0039947	Pisumionoside	C19H32O9	403.1972442	No
7346	HMDB0000447; C15568; CHEBI:49269	7a,12a-Dihydroxy-3-oxo-4-cholenoic acid	C24H36O5	403.2495758	No
7346	HMDB0014372; C07074; CHEBI:40303	Lovastatin	C24H36O5	403.2495758	No
7346	HMDB0038152	Lucidone C	C24H36O5	403.2495758	No
7363	HMDB0001938; D00362; CHEBI:43755	Lisinopril	C21H31N3O5	404.219193	No
7382	HMDB0127951	2-hydroxy-3-({3-[3-methoxy-4-(sulfooxy)phenyl]prop-2	C14H14O12S	405.0159587	No
7382	HMDB0127952	2-{[3-(4-hydroxy-3-methoxyphenyl)prop-2-enoyl]oxy}-:	C14H14O12S	405.0159587	No
7386	HMDB0041174	1-O-p-Coumaroyl-(b-D-glucose 6-O-sulfate)	C15H18O11S	405.0500935	No
7386	HMDB0125132	{4-[(1E)-3-oxo-3-[(3,4,5,6-tetrahydroxyoxan-2-yl)meth	C15H18O11S	405.0500935	No
7387	HMDB0030824	Afzelechin 7-apioside	C20H22O9	405.1193651	No
7387	HMDB0031423	omega-Salicoyisalicin	C20H22O9	405.1193651	No
7387	HMDB0038330	Benzyl 2,6-dihydroxybenzoate 2-glucoside	C20H22O9	405.1193651	No
7387	HMDB0039039	(R)-Apiumetin glucoside	C20H22O9	405.1193651	No
7387	HMDB0039172	Flacourtin	C20H22O9	405.1193651	No
7387	HMDB0040555	(E)-Oxyresveratrol 3''-O-b-D-glucoside	C20H22O9	405.1193651	No
7387	HMDB0040863	Piceatannol 4''-glucoside	C20H22O9	405.1193651	No
7387	HMDB0041265	Edulisin VI	C20H22O9	405.1193651	No
7387	HMDB0060923	Naproxen O-glucuronide	C20H22O9	405.1193651	No
7387	HMDB0129949	3,4,5-trihydroxy-6-{3-hydroxy-5-[2-(3-hydroxyphenyl)e	C20H22O9	405.1193651	No
7387	HMDB0129950	6-{3-[2-(3,5-dihydroxyphenyl)ethyl]phenoxy}-3,4,5-trih	C20H22O9	405.1193651	No
7387	HMDB0134112	2-{4-[(E)-2-(3,5-dihydroxyphenyl)ethenyl]-3-hydroxyph	C20H22O9	405.1193651	No
7387	HMDB0134113	2-{3-[(E)-2-(2,4-dihydroxyphenyl)ethenyl]-5-hydroxyph	C20H22O9	405.1193651	No
7387	HMDB0135774	3,4,5-trihydroxy-6-{3-hydroxy-5-[2-(2-hydroxyphenyl)e	C20H22O9	405.1193651	No
7387	HMDB0135775	6-{2-[2-(3,5-dihydroxyphenyl)ethyl]phenoxy}-3,4,5-trih	C20H22O9	405.1193651	No
7393	HMDB0000391; CHEBI:16390	7-Ketodeoxycholic acid	C24H38O5	405.264836	Yes
7393	HMDB0000400; CHEBI:16312	3,7-Dihydroxy-12-oxocholanoic acid	C24H38O5	405.264836	Yes
7393	HMDB0000502	3-Oxocholic acid	C24H38O5	405.264836	Yes
7393	HMDB0033399	Annocherin A	C24H38O5	405.264836	Yes
7393	HMDB0035288	(6b,7b,13R)-6,7-Diacetoxy-8,14-labdadiene-13-ol	C24H38O5	405.264836	Yes
7393	HMDB0062680; CHEBI:88108	7alpha,12alpha-dihydroxy-3-oxo-5beta-cholan-24-oic /	C24H38O5	405.264836	Yes
7403	HMDB0039895	4-Mercaptobutyl glucosinolate	C11H21NO9S3	406.0317303	Yes
7409	HMDB0013024	Neurotensin 11-13	C21H33N3O5	406.2345321	No
7410	HMDB0011678	Geranylgeranylcysteine	C23H37NO3S	406.2418207	No
7424	HMDB0129590	[4-(5-hydroxy-7-methoxy-8-methyl-4-oxo-4H-chromen	C18H16O9S	407.0416919	Yes
7425	HMDB0036862; C07601	Ginkgolide A	C20H24O9	407.1338482	No
7425	HMDB0038600	Torachrysone 8-glucoside	C20H24O9	407.1338482	No
7425	HMDB0039126	Wybutoxine	C16H20N6O7	407.1338482	No
7425	HMDB0041508	Marmesin galactoside	C20H24O9	407.1338482	No
7427	HMDB0036836; C09071	Cascarillin	C22H32O7	407.2050522	No
7427	HMDB0036864	Anhydrocinnzeylanine	C22H32O7	407.2050522	No
7429	HMDB0062190; C01192; CHEBI:17868	1-palmitoyl-dihydroxyacetone-phosphate	C19H37O7P	407.2213222	No
7429	HMDB0062323; CHEBI:75070	1-(9Z-hexadecenoyl)-glycero-3-phosphate	C19H37O7P	407.2213222	No
7430	HMDB0032021	cis-Methylbixin	C26H32O4	407.224602	No
7430	HMDB0039078	trans-Methylbixin	C26H32O4	407.224602	No
7430	HMDB0041126	Heterophylol	C26H32O4	407.224602	No
7434	HMDB0000312; CHEBI:36240	3a,7a,12b-Trihydroxy-5b-cholanoic acid	C24H40O5	407.2805955	Yes
7434	HMDB0000320	3a,4b,7a-Trihydroxy-5b-cholanoic acid	C24H40O5	407.2805955	Yes
7434	HMDB0000326; CHEBI:89219	1b,3a,12a-Trihydroxy-5b-cholanoic acid	C24H40O5	407.2805955	Yes
7434	HMDB0000330	3a,4b,12a-Trihydroxy-5b-cholanoic acid	C24H40O5	407.2805955	Yes
7434	HMDB0000364; C17727	3a,6a,7b-Trihydroxy-5b-cholanoic acid	C24H40O5	407.2805955	Yes
7434	HMDB0000371	1,3,12-Trihydroxycholan-24-oic acid	C24H40O5	407.2805955	Yes
7434	HMDB0000376	3b,7a,12a-Trihydroxy-5a-Cholanoic acid	C24H40O5	407.2805955	Yes
7434	HMDB0000390	3a,7b,12b-Trihydroxy-5b-cholanoic acid	C24H40O5	407.2805955	Yes
7434	HMDB0000395	3b,7b,12a-Trihydroxy-5b-cholanoic acid	C24H40O5	407.2805955	Yes
7434	HMDB0000404	2b,3a,7a-Trihydroxy-5b-cholanoic acid	C24H40O5	407.2805955	Yes
7434	HMDB0000414	1b,3a,7a-Trihydroxy-5b-cholanoic acid	C24H40O5	407.2805955	Yes
7434	HMDB0000415; C17726	3a,6b,7b-Trihydroxy-5b-cholanoic acid	C24H40O5	407.2805955	Yes
7434	HMDB0000419; CHEBI:88105	3b,7a,12a-Trihydroxy-5b-cholanoic acid	C24H40O5	407.2805955	Yes
7434	HMDB0000427	3b,7b,12a-Trihydroxy-5a-Cholanoic acid	C24H40O5	407.2805955	Yes

7434	HMDB0000432	3a,7b,12a-Trihydroxy-5a-Cholanoic acid	C24H40O5	407.2805955	Yes
7434	HMDB0000505	Allocholic acid	C24H40O5	407.2805955	Yes
7434	HMDB0000506	Alpha-Muricholic acid	C24H40O5	407.2805955	Yes
7434	HMDB0000527	6a,12a-Dihydroxylithocholic acid	C24H40O5	407.2805955	Yes
7434	HMDB0000619; C00695; CHEBI:16359	Cholic acid	C24H40O5	407.2805955	Yes
7434	HMDB0000760	Hyocholic acid	C24H40O5	407.2805955	Yes
7434	HMDB0000865; CHEBI:134216	Muricholic acid	C24H40O5	407.2805955	Yes
7434	HMDB0000917; C17644; CHEBI:803879	Ursocholic acid	C24H40O5	407.2805955	Yes
7447	HMDB0040793	Acidissiminol epoxide	C25H31NO4	408.2201414	No
7463	HMDB0030560	7,11,12-Triacetoxycoumestan	C21H14O9	409.0584652	Yes
7464	HMDB0130066	3,4,5-trihydroxy-6-[2-hydroxy-1-(1-oxo-1H-isochromer	C19H22O10	409.1147653	Yes
7464	HMDB0130067	3,4,5-trihydroxy-6-{[1-hydroxy-1-(1-oxo-1H-isochrome	C19H22O10	409.1147653	Yes
7465	HMDB0042005	Quinaprilat	C23H26N2O5	409.177453	Yes
7475	HMDB0001023; C11455; CHEBI:17813	4,4-Dimethylcholesta-8,14,24-trienol	C29H46O	409.3468744	No
7475	HMDB0006852; C15783	5-Dehydroavenasterol	C29H46O	409.3468744	No
7475	HMDB0006928; C11508; CHEBI:30109	Delta 8,14 -Sterol	C29H46O	409.3468744	No
7475	HMDB0032882	28-Norcyclomusalenone	C29H46O	409.3468744	No
7475	HMDB0033810	(4alpha,5alpha)-4,14-Dimethyl-9,19-cyclocholest-20-er	C29H46O	409.3468744	No
7475	HMDB0033825	Corbisterol	C29H46O	409.3468744	No
7475	HMDB0034078	22-Dehydroclerosterol	C29H46O	409.3468744	No
7475	HMDB0034196	(3beta,5alpha,22E,24S)-Stigmasta-7,22,25-trien-3-ol	C29H46O	409.3468744	No
7475	HMDB0036312	(3beta,22E,24R)-23-Methylergosta-5,7,22-trien-3-ol	C29H46O	409.3468744	No
7475	HMDB0039196	Stigmasta-4,6-dien-3-one	C29H46O	409.3468744	No
7484	HMDB0061172	O-Desmethylvenlafaxine glucuronide	C20H29NO8	410.1809167	Yes
7488	HMDB0015648; CHEBI:775715	Fesoterodine	C26H37NO3	410.2702396	No
7510	HMDB0060914	Zileuton O-glucuronide	C17H20N2O8S	411.0844284	No
7511	HMDB0038347	Methylpicraquassioside A	C19H24O10	411.1305767	Yes
7513	HMDB0030082	5-(2,3-Dihydroxy-3-methylbutyl)-4-(3,4-epoxy-4-methy	C21H32O8	411.2027487	Yes
7513	HMDB0039636	Abscisic alcohol 11-glucoside	C21H32O8	411.2027487	Yes
7527	HMDB0041868	Desacetylcefotaxime	C14H15N5O6S2	412.0394399	No
7550	HMDB0128795	[2-methoxy-4-(3,5,6,7-tetrahydroxy-4-oxo-3,4-dihydro	C16H14O11S	413.0176414	No
7550	HMDB0128796	[5,6,7-trihydroxy-2-(4-hydroxy-3-methoxyphenyl)-4-ox	C16H14O11S	413.0176414	No
7550	HMDB0128806	{4-[2,3-dioxo-3-(2,3,4,6-tetrahydroxyphenyl)propyl]-2-	C16H14O11S	413.0176414	No
7550	HMDB0128819	[2-hydroxy-5-(3,5,7-trihydroxy-6-methoxy-4-oxo-3,4-d	C16H14O11S	413.0176414	No
7550	HMDB0128820	[2-(3,4-dihydroxyphenyl)-5,7-dihydroxy-6-methoxy-4-c	C16H14O11S	413.0176414	No
7550	HMDB0128831	{5-[2,3-dioxo-3-(2,4,6-trihydroxy-3-methoxyphenyl)prc	C16H14O11S	413.0176414	No
7550	HMDB0134872	[3,5-dihydroxy-2-({7-oxo-7H-furo[3,2-g]chromen-9-yl}c	C16H14O11S	413.0176414	No
7550	HMDB0134873	[4,5-dihydroxy-2-({7-oxo-7H-furo[3,2-g]chromen-9-yl}c	C16H14O11S	413.0176414	No
7552	HMDB0127309	6-(3,4-dihydroxy-6-methyl-5-oxooxan-2-yl)-5,7-dihydrc	C21H18O9	413.0862415	No
7552	HMDB0127340	6-(3,4-dihydroxy-6-methyl-5-oxooxan-2-yl)-5,7-dihydrc	C21H18O9	413.0862415	No
7552	HMDB0134551	3,4,5-trihydroxy-6-[(4-oxo-2-phenyl-4H-chromen-3-yl)c	C21H18O9	413.0862415	No
7553	HMDB0032600	Ptelatoside A	C19H26O10	413.1456389	No
7555	HMDB0030895	(4R,5S,7R,11x)-11,12-Dihydroxy-1(10)-spirovetiven-2-c	C21H34O8	413.2178607	No
7555	HMDB0033150	(4R,5S,7R,11S)-11,12-Dihydroxy-1(10)-spirovetiven-2-c	C21H34O8	413.2178607	No
7558	HMDB0039883	Ascorbyl palmitate	C22H38O7	413.2545433	No
7571	HMDB0038425	6-Heptenyl glucosinolate	C14H25NO9S2	414.089193	No
7594	HMDB0130154	3,4-dihydroxy-5-{[(2E)-3-[4-hydroxy-3-(sulfooxy)pheny	C16H16O11S	415.0345045	Yes
7594	HMDB0130155	5-{[(2E)-3-(3,4-dihydroxyphenyl)prop-2-enoyl]oxy}-3-h	C16H16O11S	415.0345045	Yes
7594	HMDB0130156	5-{[(2E)-3-(3,4-dihydroxyphenyl)prop-2-enoyl]oxy}-4-h	C16H16O11S	415.0345045	Yes
7595	HMDB0125858	{4-[3-(5-methoxy-2,2-dimethyl-2H-chromen-6-yl)prop-	C21H20O7S	415.0883045	Yes
7595	HMDB0128730	6-{[6-(2-carboxyethyl)-4-methoxy-2H-1,3-benzodioxol-	C17H20O12	415.0883045	Yes
7595	HMDB0128731	3,4,5-trihydroxy-6-{[3-(6-hydroxy-7-methoxy-2H-1,3-b	C17H20O12	415.0883045	Yes
7595	HMDB0128735	6-{[6-(2-carboxyethyl)-5-methoxy-2H-1,3-benzodioxol-	C17H20O12	415.0883045	Yes
7595	HMDB0128736	3,4,5-trihydroxy-6-{[3-(7-hydroxy-6-methoxy-2H-1,3-b	C17H20O12	415.0883045	Yes
7596	HMDB0034157	2-Hydroxybenzaldehyde O-[xylosyl-(1->6)-glucoside]	C18H24O11	415.1252046	No
7596	HMDB0125818	6-[(4,5-dihydroxy-7-methoxy-2,2-dimethyl-3,4-dihydro	C18H24O11	415.1252046	No
7596	HMDB0125819	6-[(4,6-dihydroxy-7-methoxy-2,2-dimethyl-3,4-dihydro	C18H24O11	415.1252046	No
7596	HMDB0125824	3,4,5-trihydroxy-6-{[4-hydroxy-2-(hydroxymethyl)-7-m	C18H24O11	415.1252046	No
7596	HMDB0127776	6-[4-(4-carboxy-2-hydroxybutyl)-2-methoxyphenoxy]-3	C18H24O11	415.1252046	No
7596	HMDB0127777	3,4,5-trihydroxy-6-{[4-hydroxy-5-(4-hydroxy-3-methox	C18H24O11	415.1252046	No
7596	HMDB0127837	6-[5-(4-carboxy-2-hydroxybutyl)-2-methoxyphenoxy]-3	C18H24O11	415.1252046	No

7596	HMDB0127838	3,4,5-trihydroxy-6-{[4-hydroxy-5-(3-hydroxy-4-methox	C18H24O11	415.1252046	No
7596	HMDB0128708	6-[3-(6,7-dimethoxy-2H-1,3-benzodioxol-5-yl)propoxy]	C18H24O11	415.1252046	No
7597	HMDB0041274	Phenylethyl primeveroside	C19H28O10	415.1608697	Yes
7598	HMDB0036340	Ethyl 7-epi-12-hydroxyjasmonate glucoside	C20H32O9	415.1975077	Yes
7599	HMDB0015389; CHEBI:204734	Desonide	C24H32O6	415.2150947	Yes
7599	HMDB0030522	Citreoviridin D	C24H32O6	415.2150947	Yes
7599	HMDB0030749	Heterotropan	C24H32O6	415.2150947	Yes
7599	HMDB0035849	Armillyl everninate	C24H32O6	415.2150947	Yes
7599	HMDB0039145	Diasarone 2	C24H32O6	415.2150947	Yes
7599	HMDB0134777	7-{[2,6-dihydroxy-5-(hydroxymethyl)-2,5,8a-trimethyl-	C24H32O6	415.2150947	Yes
7599	HMDB0134778	7-{[2,6-dihydroxy-2-(hydroxymethyl)-5,5,8a-trimethyl-	C24H32O6	415.2150947	Yes
7599	HMDB0134779	7-{[2,6-dihydroxy-8a-(hydroxymethyl)-2,5,5-trimethyl-	C24H32O6	415.2150947	Yes
7599	HMDB0134780	7-[(2,6-dihydroxy-2,5,5,8a-tetramethyl-decahydronapl	C24H32O6	415.2150947	Yes
7599	HMDB0134781	7-[(2,6-dihydroxy-2,5,5,8a-tetramethyl-decahydronapl	C24H32O6	415.2150947	Yes
7599	HMDB0134782	7-[(2,6-dihydroxy-2,5,5,8a-tetramethyl-decahydronapl	C24H32O6	415.2150947	Yes
7599	HMDB0134783	7-[(2,4a,6-trihydroxy-2,5,5,8a-tetramethyl-decahydror	C24H32O6	415.2150947	Yes
7599	HMDB0134784	7-[(2,4,6-trihydroxy-2,5,5,8a-tetramethyl-decahydrona	C24H32O6	415.2150947	Yes
7599	HMDB0134785	7-[(2,3,6-trihydroxy-2,5,5,8a-tetramethyl-decahydrona	C24H32O6	415.2150947	Yes
7599	HMDB0134786	7-[(1,2,6-trihydroxy-2,5,5,8a-tetramethyl-decahydrona	C24H32O6	415.2150947	Yes
7599	HMDB0134787	7-[(2,6,8-trihydroxy-2,5,5,8a-tetramethyl-decahydrona	C24H32O6	415.2150947	Yes
7599	HMDB0134788	7-[(2,6,7-trihydroxy-2,5,5,8a-tetramethyl-decahydrona	C24H32O6	415.2150947	Yes
7607	HMDB0000430; CHEBI:89324	24,25-Dihydroxyvitamin D	C27H44O3	415.3211891	No
7607	HMDB0001420; CHEBI:89079	25,26-dihydroxyvitamin D	C27H44O3	415.3211891	No
7607	HMDB0001903; C01673; CHEBI:17823	Calcitriol	C27H44O3	415.3211891	No
7607	HMDB0002197; C05457; CHEBI:28477	7a,12a-Dihydroxy-cholestene-3-one	C27H44O3	415.3211891	No
7607	HMDB0006226	24R,25-Dihydroxyvitamin D3	C27H44O3	415.3211891	No
7607	HMDB0006720	23S,25-dihydroxyvitamin D3	C27H44O3	415.3211891	No
7607	HMDB0012453; C17333	3 beta-Hydroxy-5-cholestenoate	C27H44O3	415.3211891	No
7607	HMDB0012457; C17331	7 alpha,24-Dihydroxy-4-cholesten-3-one	C27H44O3	415.3211891	No
7607	HMDB0012459; C17336	7 alpha,26-Dihydroxy-4-cholesten-3-one	C27H44O3	415.3211891	No
7607	HMDB0015046; C08127; CHEBI:7931	Paricalcitol	C27H44O3	415.3211891	No
7607	HMDB0030024; C03963; CHEBI:15578	Sarsasapogenin	C27H44O3	415.3211891	No
7607	HMDB0030047; C16819	Neotigogenin	C27H44O3	415.3211891	No
7607	HMDB0030048; C08913; CHEBI:28933	Smilagenin	C27H44O3	415.3211891	No
7607	HMDB0035300	Octadecyl cis-p-coumarate	C27H44O3	415.3211891	No
7607	HMDB0060133	(24R)-24,25-Dihydroxycalciol	C27H44O3	415.3211891	No
7607	HMDB0060425; C17332	7alpha,25-Dihydroxy-4-cholesten-3-one	C27H44O3	415.3211891	No
7607	HMDB0060508; C07712	Secalciferol	C27H44O3	415.3211891	No
7607	HMDB0062401; C17331; CHEBI:48701	7alpha,24-dihydroxycholest-4-en-3-one	C27H44O3	415.3211891	No
7607	HMDB0062610; CHEBI:87476	(25R)-7alpha,26-dihydroxycholest-4-en-3-one	C27H44O3	415.3211891	No
7607	HMDB0062736; CHEBI:86115	(25R)-3beta,4beta-dihydroxycholest-5-en-26-al	C27H44O3	415.3211891	No
7632	HMDB0128381	3-[3,5-dihydroxy-4-(sulfooxy)benzoyloxy]-2,4,5-trihydr	C14H10O13S	416.9784374	No
7632	HMDB0128391	3-hydroxy-4-(sulfooxy)-5-(2,3,4,5-tetrahydroxybenzoyl	C14H10O13S	416.9784374	No
7632	HMDB0128400	5-[3,5-dihydroxy-4-(sulfooxy)benzoyloxy]-2,3,4-trihydr	C14H10O13S	416.9784374	No
7635	HMDB0034590	Shoyuflavone C	C19H14O11	417.0468075	No
7638	HMDB0040353	2-[4-(3-Hydroxypropyl)-2-methoxyphenoxy]-1,3-propa	C19H30O10	417.1766077	Yes
7669	HMDB0037483	2'',4'',6''-Trihydroxydihydrochalcone 2''-glucoside	C21H24O9	419.1337806	No
7669	HMDB0037484	2'',4'',6''-Trihydroxydihydrochalcone 4''-glucoside	C21H24O9	419.1337806	No
7669	HMDB0129923	3,4,5-trihydroxy-6-{3-[2-(3-hydroxyphenyl)ethyl]-5-me	C21H24O9	419.1337806	No
7669	HMDB0129924	3,4,5-trihydroxy-6-{3-[2-(3-hydroxy-5-methoxyphenyl)	C21H24O9	419.1337806	No
7669	HMDB0135394	3,4,5-trihydroxy-6-[4-hydroxy-2-(1-hydroxy-3-phenylpı	C21H24O9	419.1337806	No
7669	HMDB0135395	3,4,5-trihydroxy-6-[4-hydroxy-3-(1-hydroxy-3-phenylpı	C21H24O9	419.1337806	No
7669	HMDB0135762	3,4,5-trihydroxy-6-{3-[2-(2-hydroxyphenyl)ethyl]-5-me	C21H24O9	419.1337806	No
7669	HMDB0135763	3,4,5-trihydroxy-6-{2-[2-(3-hydroxy-5-methoxyphenyl)	C21H24O9	419.1337806	No
7671	HMDB0015354; CHEBI:894445	Aminophylline	C16H24N10O4	419.191835	Yes
7712	HMDB0032853	3-(1,1-Dimethylallyl)scopoletin 7-glucoside	C21H26O9	421.1526335	No
7714	HMDB0032386; C07874	2-Methylacetophenone	C20H38O7S	421.2250795	No
7732	HMDB0006461; CHEBI:72715	Linoelaidyl carnitine	C25H45NO4	422.3261575	No
7732	HMDB0006469; CHEBI:84098	Linoleyl carnitine	C25H45NO4	422.3261575	No
7747	HMDB0005022; C01844; CHEBI:63618	Pravastatin	C23H36O7	423.2392109	No
7750	HMDB0000307	1b-Hydroxycholic acid	C24H40O6	423.2752504	No

7750	HMDB0000311	3b,4b,7a,12a-Tetrahydroxy-5b-cholanoic acid	C24H40O6	423.2752504	No
7750	HMDB0000316	2b,3a,7a,12a-Tetrahydroxy-5b-cholanoic acid	C24H40O6	423.2752504	No
7750	HMDB0000340	3a,7a,12a,19-Tetrahydroxy-5b-cholanoic acid	C24H40O6	423.2752504	No
7750	HMDB0000367	3a,6b,7b,12a-Tetrahydroxy-5b-cholanoic acid	C24H40O6	423.2752504	No
7750	HMDB0000377	3a,6b,7b,12b-Tetrahydroxy-5b-cholanoic acid	C24H40O6	423.2752504	No
7750	HMDB0000399	3a,6b,7a,12a-Tetrahydroxy-5b-cholanoic acid	C24H40O6	423.2752504	No
7750	HMDB0000433	1,3,7,12-Tetrahydroxycholan-24-oic acid	C24H40O6	423.2752504	No
7750	HMDB0000437	3a,4b,7a,12a-Tetrahydroxy-5b-cholanoic acid	C24H40O6	423.2752504	No
7750	HMDB0013158	1b,3a,7b-Trihydroxy-5b-cholanoic acid	C24H40O6	423.2752504	No
7750	HMDB0013192	3a,7b,21-Trihydroxy-5b-cholanoic acid	C24H40O6	423.2752504	No
7752	HMDB0006327; C14153; CHEBI:33270	Alpha-Tocotrienol	C29H44O2	423.3270167	No
7752	HMDB0038656	(22E,24R)-Stigmasta-4,22-diene-3,6-dione	C29H44O2	423.3270167	No
7755	HMDB0002348; CHEBI:31001	Octacosanoic acid	C28H56O2	423.4206797	No
7755	HMDB0033784	28-Hydroxy-7-octacosanone	C28H56O2	423.4206797	No
7755	HMDB0040885	10-Octacosene-1,12-diol	C28H56O2	423.4206797	No
7768	HMDB0060856	O-Desmethyl-tramado glucuronide	C21H31NO8	424.197249	No
7768	HMDB0061167	N,O-Didesmethylvenlafaxine glucuronide	C21H31NO8	424.197249	No
7830	HMDB0000851; CHEBI:88539	Pyridinoline	C18H28N4O8	427.1807822	Yes
7832	HMDB0035610	Taraxacolide 1-O-b-D-glucopyranoside	C21H32O9	427.197145	Yes
7834	HMDB0015163; C07469; CHEBI:5959	Irbesartan	C25H28N6O	427.2244922	No
7836	HMDB0003822; C02530	Cholesteryl acetate	C29H48O2	427.3577498	No
7836	HMDB0012168	4alpha-Formyl-4beta-methyl-5alpha-cholesta-8-en-3be	C29H48O2	427.3577498	No
7836	HMDB0012170	4alpha-Hydroxymethyl-4beta-methyl-5alpha-cholesta-	C29H48O2	427.3577498	No
7836	HMDB0034422	3-Hydroxystigmast-5-en-7-one	C29H48O2	427.3577498	No
7836	HMDB0036018	Mangiferdesmethylursanone	C29H48O2	427.3577498	No
7836	HMDB0039498	(6beta,24R)-6-Hydroxystigmast-4-en-3-one	C29H48O2	427.3577498	No
7836	HMDB0039822	Stigmastane-3,6-dione	C29H48O2	427.3577498	No
7836	HMDB0041112	(3alpha,5alpha,7alpha)-14-Methylergosta-9(11),24(28)	C29H48O2	427.3577498	No
7836	HMDB0059643	3-beta-Hydroxy-4-beta-methyl-5-alpha-cholest-7-ene-4	C29H48O2	427.3577498	No
7836	HMDB0062388; CHEBI:87046	4?-formyl-4?-methyl-5?-cholesta-8-en-3?-ol	C29H48O2	427.3577498	No
7836	HMDB0062390; CHEBI:87289	4?-hydroxymethyl-4?-methyl-5?-cholesta-8,24-dien-3?	C29H48O2	427.3577498	No
7849	HMDB0013855	Sulfamethoxazole N1-glucuronide	C16H19N3O9S	428.0774393	No
7854	HMDB0062336; CHEBI:134021	N-Oleoyl phenylalanine	C27H43NO3	428.3148087	No
7870	HMDB0033587	Coumestrin	C21H18O10	429.0807648	Yes
7870	HMDB0041717	Daidzein 4''-O-glucuronide	C21H18O10	429.0807648	Yes
7870	HMDB0041718	Daidzein 7-O-glucuronide	C21H18O10	429.0807648	Yes
7870	HMDB0127308	6-(3,4-dihydroxy-6-methyl-5-oxooxan-2-yl)-2-(3,4-dihy	C21H18O10	429.0807648	Yes
7870	HMDB0129981	3,4,5-trihydroxy-6-[(5-hydroxy-4-oxo-3-phenyl-4H-chro	C21H18O10	429.0807648	Yes
7870	HMDB0132466	3,4,5-trihydroxy-6-[(5-hydroxy-4-oxo-2-phenyl-4H-chro	C21H18O10	429.0807648	Yes
7870	HMDB0132467	3,4,5-trihydroxy-6-[(7-hydroxy-4-oxo-2-phenyl-4H-chro	C21H18O10	429.0807648	Yes
7870	HMDB0133342	3,4,5-trihydroxy-6-{[2-(3-hydroxyphenyl)-4-oxo-4H-chr	C21H18O10	429.0807648	Yes
7870	HMDB0133343	3,4,5-trihydroxy-6-[3-(7-hydroxy-4-oxo-4H-chromen-2-	C21H18O10	429.0807648	Yes
7870	HMDB0134552	3,4,5-trihydroxy-6-[2-(3-hydroxy-4-oxo-4H-chromen-2-	C21H18O10	429.0807648	Yes
7870	HMDB0134553	3,4,5-trihydroxy-6-{[2-(2-hydroxyphenyl)-4-oxo-4H-chr	C21H18O10	429.0807648	Yes
7870	HMDB0134554	3,4,5-trihydroxy-6-[(3-hydroxy-4-oxo-2-phenyl-4H-chro	C21H18O10	429.0807648	Yes
7870	HMDB0134555	3,4,5-trihydroxy-6-[(6-hydroxy-4-oxo-2-phenyl-4H-chro	C21H18O10	429.0807648	Yes
7870	HMDB0134556	3,4,5-trihydroxy-6-[(3-hydroxy-4-oxo-2-phenyl-4H-chro	C21H18O10	429.0807648	Yes
7870	HMDB0134557	3,4,5-trihydroxy-6-[(7-hydroxy-4-oxo-2-phenyl-4H-chro	C21H18O10	429.0807648	Yes
7870	HMDB0134558	3,4,5-trihydroxy-6-[3-(3-hydroxy-4-oxo-4H-chromen-2-	C21H18O10	429.0807648	Yes
7870	HMDB0134559	3,4,5-trihydroxy-6-{[2-(3-hydroxyphenyl)-4-oxo-4H-chr	C21H18O10	429.0807648	Yes
7870	HMDB0134561	3,4,5-trihydroxy-6-[4-(3-hydroxy-4-oxo-4H-chromen-2-	C21H18O10	429.0807648	Yes
7870	HMDB0134562	3,4,5-trihydroxy-6-{[2-(4-hydroxyphenyl)-4-oxo-4H-chr	C21H18O10	429.0807648	Yes
7870	HMDB0134563	3,4,5-trihydroxy-6-[(3-hydroxy-4-oxo-2-phenyl-4H-chro	C21H18O10	429.0807648	Yes
7870	HMDB0134564	3,4,5-trihydroxy-6-[(5-hydroxy-4-oxo-2-phenyl-4H-chro	C21H18O10	429.0807648	Yes
7872	HMDB0032622	Phenethyl rutinoside	C20H30O10	429.1767649	No
7874	HMDB0015353; CHEBI:3207	Budesonide	C25H34O6	429.230675	Yes
7874	HMDB0033080	Antibiotic CJ 15544	C25H34O6	429.230675	Yes
7874	HMDB0033081	Antibiotic CP 412065	C25H34O6	429.230675	Yes
7874	HMDB0034659	Homofukinolide	C25H34O6	429.230675	Yes
7874	HMDB0040990	3b,6b-Diangeloyloxy-7(11)-eremophilen-12,8b-olide	C25H34O6	429.230675	Yes
7880	HMDB0035640	Sintaxanthin	C31H42O	429.3168163	No

7881	HMDB0001893; C02477; CHEBI:18145	Alpha-Tocopherol	C29H50O2	429.3735658	No
7881	HMDB0012171	4alpha-Hydroxymethyl-4beta-methyl-5alpha-cholesta-	C29H50O2	429.3735658	No
7881	HMDB0030022	Ikshusterol	C29H50O2	429.3735658	No
7881	HMDB0059642	4-beta-Hydroxymethyl-4-alpha-methyl-5-alpha-cholest	C29H50O2	429.3735658	No
7881	HMDB0062391; CHEBI:87045	4?-hydroxymethyl-4?-methyl-5?-cholesta-8-en-3?-ol	C29H50O2	429.3735658	No
7907	HMDB0029501	alpha-Rhamnorobin	C21H20O10	431.1005975	No
7907	HMDB0029577	6-Hydroxydaidzein 4''-glucoside	C21H20O10	431.1005975	No
7907	HMDB0029679	1,8-Dihydroxy-3-hydroxymethylanthraquinone 8-O-b-D	C21H20O10	431.1005975	No
7907	HMDB0033982	Isogenistein 7-glucoside	C21H20O10	431.1005975	No
7907	HMDB0033988; C09126; CHEBI:27514	Genistin	C21H20O10	431.1005975	No
7907	HMDB0034405	w-O-beta-D-Glucopyranosylaloeemodin	C21H20O10	431.1005975	No
7907	HMDB0037340; C04608; CHEBI:16778	Cosmosiin	C21H20O10	431.1005975	No
7907	HMDB0038293	1-O-beta-D-Glucopyranosylaloeemodin	C21H20O10	431.1005975	No
7907	HMDB0038740	Genistein 5-glucoside	C21H20O10	431.1005975	No
7907	HMDB0038887	Glucoemodin	C21H20O10	431.1005975	No
7907	HMDB0038898	6-C-Fucosylluteolin	C21H20O10	431.1005975	No
7907	HMDB0040542	Kaempferol 3-O-alpha-L-rhamnofuranoside	C21H20O10	431.1005975	No
7907	HMDB0041591	Apigenin 4''-O-glucoside	C21H20O10	431.1005975	No
7907	HMDB0041722	Dihydrodaidzein 7-O-glucuronide	C21H20O10	431.1005975	No
7907	HMDB0126551	3,5-dihydroxy-2-(4-hydroxyphenyl)-7-[(3,4,5-trihydroxy	C21H20O10	431.1005975	No
7907	HMDB0127106	5,7-dihydroxy-2-(4-hydroxyphenyl)-6-[3,4,5-trihydroxy	C21H20O10	431.1005975	No
7907	HMDB0127129	5,7-dihydroxy-2-(4-hydroxyphenyl)-8-[3,4,5-trihydroxy	C21H20O10	431.1005975	No
7907	HMDB0127134	5,6,7-trihydroxy-2-phenyl-8-[3,4,5-trihydroxy-6-(hydro	C21H20O10	431.1005975	No
7907	HMDB0127136	5,7-dihydroxy-2-(3-hydroxyphenyl)-8-[3,4,5-trihydroxy	C21H20O10	431.1005975	No
7907	HMDB0127223	5,6-dihydroxy-2-(4-hydroxyphenyl)-7-methoxy-8-(3,4,5	C21H20O10	431.1005975	No
7907	HMDB0127224	2-(3,4-dihydroxyphenyl)-5-hydroxy-7-methoxy-8-(3,4,5	C21H20O10	431.1005975	No
7907	HMDB0127294	5,7-dihydroxy-2-(4-hydroxy-3-methoxyphenyl)-8-(3,4,5	C21H20O10	431.1005975	No
7907	HMDB0127358	5,7-dihydroxy-2-(3-hydroxyphenyl)-6-[3,4,5-trihydroxy	C21H20O10	431.1005975	No
7907	HMDB0127485	5,7-dihydroxy-2-(4-hydroxyphenyl)-3-[(3,4,5-trihydroxy	C21H20O10	431.1005975	No
7907	HMDB0128089	3,4,5-trihydroxy-6-[(5-hydroxy-4-oxo-2-phenyl-3,4-dihy	C21H20O10	431.1005975	No
7907	HMDB0128090	3,4,5-trihydroxy-6-[(7-hydroxy-4-oxo-2-phenyl-3,4-dihy	C21H20O10	431.1005975	No
7907	HMDB0128094	6-[3,5-dihydroxy-2-(3-phenylprop-2-enoyl)phenoxy]-3,	C21H20O10	431.1005975	No
7907	HMDB0128095	6-[3,5-dihydroxy-4-(3-phenylprop-2-enoyl)phenoxy]-3,	C21H20O10	431.1005975	No
7907	HMDB0128107	6-{3,5-dihydroxy-2-[(2E)-3-phenylprop-2-enoyl]phenox	C21H20O10	431.1005975	No
7907	HMDB0129987	3,4,5-trihydroxy-6-[(7-hydroxy-4-oxo-3-phenyl-3,4-dihy	C21H20O10	431.1005975	No
7907	HMDB0129988	3,4,5-trihydroxy-6-[(5-hydroxy-4-oxo-3-phenyl-3,4-dihy	C21H20O10	431.1005975	No
7907	HMDB0134579	3,4,5-trihydroxy-6-[(8-hydroxy-4-oxo-2-phenyl-3,4-dihy	C21H20O10	431.1005975	No
7907	HMDB0141940	2-(3,4-dihydroxyphenyl)-7-{[3,4,5-trihydroxy-6-(hydrox	C21H20O10	431.1005975	No
7908	HMDB0031723	Trichocarposide	C22H24O9	431.1333976	Yes
7908	HMDB0033855; C16223	Medicocarpin	C22H24O9	431.1333976	Yes
7908	HMDB0037689	3-Methoxynobiletin	C22H24O9	431.1333976	Yes
7908	HMDB0039927	4''-O-Methylglucoliquiritigenin	C22H24O9	431.1333976	Yes
7908	HMDB0040503	Pinostrobin 5-glucoside	C22H24O9	431.1333976	Yes
7908	HMDB0130340	3,4,5-trihydroxy-6-{2-[(1E)-3-(4-hydroxy-2-methoxyphe	C22H24O9	431.1333976	Yes
7908	HMDB0130341	3,4,5-trihydroxy-6-{4-[3-(2-hydroxyphenyl)prop-2-en-1	C22H24O9	431.1333976	Yes
7908	HMDB0130344	3,4,5-trihydroxy-6-{6-hydroxy-2-methoxy-3-[(2E)-3-phe	C22H24O9	431.1333976	Yes
7908	HMDB0130345	3,4,5-trihydroxy-6-{2-hydroxy-3-methoxy-4-[(2E)-3-phe	C22H24O9	431.1333976	Yes
7908	HMDB0130348	3,4,5-trihydroxy-6-[2-hydroxy-4-methoxy-5-(3-phenylp	C22H24O9	431.1333976	Yes
7908	HMDB0130349	3,4,5-trihydroxy-6-[2-hydroxy-5-methoxy-4-(3-phenylp	C22H24O9	431.1333976	Yes
7908	HMDB0130351	3,4,5-trihydroxy-6-{4-[(1E)-3-(4-hydroxy-2-methoxyphe	C22H24O9	431.1333976	Yes
7908	HMDB0130352	3,4,5-trihydroxy-6-{4-[3-(4-hydroxyphenyl)prop-2-en-1	C22H24O9	431.1333976	Yes
7908	HMDB0130354	3,4,5-trihydroxy-6-{3-[(1E)-3-(4-hydroxy-2-methoxyphe	C22H24O9	431.1333976	Yes
7908	HMDB0130355	3,4,5-trihydroxy-6-{4-[3-(3-hydroxyphenyl)prop-2-en-1	C22H24O9	431.1333976	Yes
7908	HMDB0130357	3,4,5-trihydroxy-6-{3-methoxy-4-[(3-phenyloxiran-2-yl)	C22H24O9	431.1333976	Yes
7908	HMDB0130995	6-{4-[(E)-2-(3,5-dimethoxyphenyl)ethenyl]phenoxy}-3,4	C22H24O9	431.1333976	Yes
7908	HMDB0135632	3,4,5-trihydroxy-6-{2-[3-(4-methoxyphenyl)-3-oxoprop	C22H24O9	431.1333976	Yes
7908	HMDB0135633	3,4,5-trihydroxy-6-[3-(4-methoxyphenyl)-3-oxo-1-phen	C22H24O9	431.1333976	Yes
7908	HMDB0135635	3,4,5-trihydroxy-6-[5-methoxy-2-(3-phenylpropanoyl)p	C22H24O9	431.1333976	Yes
7908	HMDB0135636	3,4,5-trihydroxy-6-{3-[3-(4-methoxyphenyl)-3-oxoprop	C22H24O9	431.1333976	Yes
7908	HMDB0135638	3,4,5-trihydroxy-6-{4-[3-(4-methoxyphenyl)-3-oxoprop	C22H24O9	431.1333976	Yes
7910	HMDB0038787	Melleolide B	C24H32O7	431.2075177	Yes

7910	HMDB0038793	Melleolide G	C24H32O7	431.2075177	Yes
7911	HMDB0062316; CHEBI:74933	1-(6Z,9Z,12Z-octadecatrienoyl)-glycero-3-phosphate	C21H37O7P	431.2206177	Yes
7911	HMDB0062320; CHEBI:74831	1-(9Z,12Z,15Z-octadecatrienoyl)-glycero-3-phosphate	C21H37O7P	431.2206177	Yes
7911	HMDB0114743	LPA(18:3(6Z,9Z,12Z)/0:0)	C21H37O7P	431.2206177	Yes
7911	HMDB0114744	LPA(18:3(9Z,12Z,15Z)/0:0)	C21H37O7P	431.2206177	Yes
7912	HMDB0037823	Glucosyl (2E,6E,10x)-10,11-dihydroxy-2,6-farnesadienc	C21H36O9	431.22861	Yes
7914	HMDB0006228; CHEBI:47799	24-Hydroxycalcitriol	C27H44O4	431.3185578	No
7914	HMDB0012454; HMDB0062211; C17335; CHI	3beta,7alpha-Dihydroxy-5-cholestenoate	C27H44O4	431.3185578	No
7914	HMDB0030702	Neochlorogenin	C27H44O4	431.3185578	No
7914	HMDB0033769	Rockogenin	C27H44O4	431.3185578	No
7914	HMDB0060134; CHEBI:47803	23S,25,26-Trihydroxyvitamin D3	C27H44O4	431.3185578	No
7914	HMDB0062399; CHEBI:48714	4-cholesten-7?,12?,24-triol-3-one	C27H44O4	431.3185578	No
7914	HMDB0062400; CHEBI:48833	7alpha,12alpha,26-trihydroxycholest-4-en-3-one	C27H44O4	431.3185578	No
7914	HMDB0062686; CHEBI:90970	1alpha,23(S),25-trihydroxyvitamin D3	C27H44O4	431.3185578	No
7914	HMDB0062712; CHEBI:72727	3alpha,7beta-dihydroxy-5beta-cholest-24-en-26-oic Ac	C27H44O4	431.3185578	No
7924	HMDB0010341; CHEBI:32645	Dextrorphan O-glucuronide	C23H31NO7	432.2032881	No
7924	HMDB0014826; C07908; CHEBI:8764	Mycophenolate mofetil	C23H31NO7	432.2032881	No
7940	HMDB0031347	2-O-Caffeoylarbutin	C21H22O10	433.1133144	Yes
7940	HMDB0033739	Floribundoside	C21H22O10	433.1133144	Yes
7940	HMDB0037480	Eriodictin	C21H22O10	433.1133144	Yes
7940	HMDB0038775	5,7,8-Trihydroxyflavanone 7-glucoside	C21H22O10	433.1133144	Yes
7940	HMDB0039935	Dihydrogenistin	C21H22O10	433.1133144	Yes
7940	HMDB0040536	(2R,3R)-3,3'',4'',7-Tetrahydroxyflavanone 7-O-alpha-L-I	C21H22O10	433.1133144	Yes
7940	HMDB0040832	5-Hydroxyaloin A	C21H22O10	433.1133144	Yes
7940	HMDB0041150	7-Hydroxyaloin B	C21H22O10	433.1133144	Yes
7940	HMDB0060772	4''-Hydroxyfenoprofen glucuronide	C21H22O10	433.1133144	Yes
7940	HMDB0124767	5,7-dihydroxy-2-(4-hydroxyphenyl)-8-[3,4,5-trihydroxy	C21H22O10	433.1133144	Yes
7940	HMDB0128100	6-[3,5-dihydroxy-2-(3-phenylpropanoyl)phenoxy]-3,4,5	C21H22O10	433.1133144	Yes
7940	HMDB0128101	6-[3,5-dihydroxy-4-(3-phenylpropanoyl)phenoxy]-3,4,5	C21H22O10	433.1133144	Yes
7940	HMDB0130017	6-[3,5-dihydroxy-2-(2-phenylpropanoyl)phenoxy]-3,4,5	C21H22O10	433.1133144	Yes
7940	HMDB0130018	6-[3,5-dihydroxy-4-(2-phenylpropanoyl)phenoxy]-3,4,5	C21H22O10	433.1133144	Yes
7940	HMDB0134585	3,4,5-trihydroxy-6-{3-hydroxy-5-[(E)-2-(3-hydroxy-4-m	C21H22O10	433.1133144	Yes
7940	HMDB0134586	6-{5-[(E)-2-(3,5-dihydroxyphenyl)ethenyl]-2-methoxypl	C21H22O10	433.1133144	Yes
7940	HMDB0135129	6-[3-(3,7-dihydroxy-3,4-dihydro-2H-1-benzopyran-2-yl)	C21H22O10	433.1133144	Yes
7940	HMDB0135130	3,4,5-trihydroxy-6-{[3-hydroxy-2-(3-hydroxyphenyl)-3,4	C21H22O10	433.1133144	Yes
7940	HMDB0135368	3,4,5-trihydroxy-6-[4-hydroxy-2-(3-hydroxy-3-phenylpr	C21H22O10	433.1133144	Yes
7940	HMDB0135369	3,4,5-trihydroxy-6-[4-hydroxy-3-(3-hydroxy-3-phenylpr	C21H22O10	433.1133144	Yes
7940	HMDB0135371	6-{3-[3-(2,5-dihydroxyphenyl)-3-oxopropyl]phenoxy}-3	C21H22O10	433.1133144	Yes
7940	HMDB0135372	3,4,5-trihydroxy-6-{4-hydroxy-2-[3-(3-hydroxyphenyl)p	C21H22O10	433.1133144	Yes
7940	HMDB0135373	3,4,5-trihydroxy-6-{4-hydroxy-3-[3-(3-hydroxyphenyl)p	C21H22O10	433.1133144	Yes
7940	HMDB0135375	6-{4-[3-(2,5-dihydroxyphenyl)-3-oxopropyl]phenoxy}-3	C21H22O10	433.1133144	Yes
7940	HMDB0135376	3,4,5-trihydroxy-6-{4-hydroxy-2-[3-(4-hydroxyphenyl)p	C21H22O10	433.1133144	Yes
7940	HMDB0135377	3,4,5-trihydroxy-6-{4-hydroxy-3-[3-(4-hydroxyphenyl)p	C21H22O10	433.1133144	Yes
7940	HMDB0135379	6-{2-[3-(2,5-dihydroxyphenyl)-3-oxopropyl]phenoxy}-3	C21H22O10	433.1133144	Yes
7940	HMDB0135380	3,4,5-trihydroxy-6-{4-hydroxy-2-[3-(2-hydroxyphenyl)p	C21H22O10	433.1133144	Yes
7940	HMDB0135381	3,4,5-trihydroxy-6-{4-hydroxy-3-[3-(2-hydroxyphenyl)p	C21H22O10	433.1133144	Yes
7940	HMDB0135382	6-[2,5-dihydroxy-4-(3-phenylpropanoyl)phenoxy]-3,4,5	C21H22O10	433.1133144	Yes
7940	HMDB0135383	6-[4,5-dihydroxy-2-(3-phenylpropanoyl)phenoxy]-3,4,5	C21H22O10	433.1133144	Yes
7940	HMDB0135384	6-[2,4-dihydroxy-5-(3-phenylpropanoyl)phenoxy]-3,4,5	C21H22O10	433.1133144	Yes
7940	HMDB0135386	6-[2,5-dihydroxy-3-(3-phenylpropanoyl)phenoxy]-3,4,5	C21H22O10	433.1133144	Yes
7940	HMDB0135387	6-[2,4-dihydroxy-6-(3-phenylpropanoyl)phenoxy]-3,4,5	C21H22O10	433.1133144	Yes
7940	HMDB0135388	6-[3,4-dihydroxy-5-(3-phenylpropanoyl)phenoxy]-3,4,5	C21H22O10	433.1133144	Yes
7940	HMDB0135390	6-[3,6-dihydroxy-2-(3-phenylpropanoyl)phenoxy]-3,4,5	C21H22O10	433.1133144	Yes
7940	HMDB0135391	6-[3,4-dihydroxy-2-(3-phenylpropanoyl)phenoxy]-3,4,5	C21H22O10	433.1133144	Yes
7940	HMDB0135392	6-[2,4-dihydroxy-3-(3-phenylpropanoyl)phenoxy]-3,4,5	C21H22O10	433.1133144	Yes
7940	HMDB0135418	6-{2-[3-(3,4-dihydroxyphenyl)-3-oxopropyl]phenoxy}-3	C21H22O10	433.1133144	Yes
7940	HMDB0135419	3,4,5-trihydroxy-6-{2-hydroxy-5-[3-(2-hydroxyphenyl)p	C21H22O10	433.1133144	Yes
7940	HMDB0135420	3,4,5-trihydroxy-6-{2-hydroxy-4-[3-(2-hydroxyphenyl)p	C21H22O10	433.1133144	Yes
7940	HMDB0135423	6-[2,3-dihydroxy-5-(3-phenylpropanoyl)phenoxy]-3,4,5	C21H22O10	433.1133144	Yes
7940	HMDB0135424	6-[2,6-dihydroxy-4-(3-phenylpropanoyl)phenoxy]-3,4,5	C21H22O10	433.1133144	Yes
7940	HMDB0135428	6-[2,6-dihydroxy-3-(3-phenylpropanoyl)phenoxy]-3,4,5	C21H22O10	433.1133144	Yes

7940	HMDB0135429	6-[2,3-dihydroxy-4-(3-phenylpropanoyl)phenoxy]-3,4,5	C21H22O10	433.1133144	Yes
7940	HMDB0135431	3,4,5-trihydroxy-6-[2-hydroxy-5-(3-hydroxy-3-phenylpı	C21H22O10	433.1133144	Yes
7940	HMDB0135432	3,4,5-trihydroxy-6-[2-hydroxy-4-(3-hydroxy-3-phenylpı	C21H22O10	433.1133144	Yes
7940	HMDB0135436	6-{3-[3-(3,4-dihydroxyphenyl)-3-oxopropyl]phenoxy}-3	C21H22O10	433.1133144	Yes
7940	HMDB0135437	3,4,5-trihydroxy-6-{2-hydroxy-5-[3-(3-hydroxyphenyl)p	C21H22O10	433.1133144	Yes
7940	HMDB0135442	6-{4-[3-(3,4-dihydroxyphenyl)-3-oxopropyl]phenoxy}-3	C21H22O10	433.1133144	Yes
7940	HMDB0135443	3,4,5-trihydroxy-6-{2-hydroxy-5-[3-(4-hydroxyphenyl)p	C21H22O10	433.1133144	Yes
7940	HMDB0135444	3,4,5-trihydroxy-6-{2-hydroxy-4-[3-(4-hydroxyphenyl)p	C21H22O10	433.1133144	Yes
7940	HMDB0140590	5-hydroxy-2-(4-hydroxyphenyl)-7-{[3,4,5-trihydroxy-6-	C21H22O10	433.1133144	Yes
7940	HMDB0141964	2-[3,4-bis(2-hydroxyethoxy)phenyl]-3,5-dihydroxy-7-(2	C21H22O10	433.1133144	Yes
7942	HMDB0007852; C00416	LPA(0:0/18:2(9Z,12Z))	C21H39O7P	433.2359405	Yes
7942	HMDB0007856; C00416; CHEBI:62834	LPA(18:2(9Z,12Z)/0:0)	C21H39O7P	433.2359405	Yes
7944	HMDB0037183; CHEBI:53425	Polysorbate 60	C22H42O8	433.2804715	No
7945	HMDB0000359	3a,7a-Dihydroxycoprostanic acid	C27H46O4	433.3326276	No
7945	HMDB0003533; C01301; CHEBI:16466	3a,7a,12a-Trihydroxy-5b-cholestan-26-al	C27H46O4	433.3326276	No
7945	HMDB0062208; CHEBI:63848	3alpha,7alpha,24(S)-trihydroxy-5beta-cholestan-27-al	C27H46O4	433.3326276	No
7950	HMDB0014323; CHEBI:9927	Valsartan	C24H29N5O3	434.2194424	Yes
7970	HMDB0002195	Varanic acid	C26H44O5	435.3106304	No
7987	HMDB0033469; CHEBI:85530	N1,N10-Dicoumaroylspermidine	C25H31N3O4	436.2259158	No
7989	HMDB0011152	PE(P-16:0e/0:0)	C21H44NO6P	436.2829895	No
8007	HMDB0007850; C00416	LPA(0:0/18:0)	C21H43O7P	437.2675157	No
8007	HMDB0007854; C00416	LPA(18:0/0:0)	C21H43O7P	437.2675157	No
8010	HMDB0002126	27-Nor-5b-cholestane-3a,7a,12a,24,25-pentol	C26H46O5	437.3259823	No
8036	HMDB0039914	1,6-Dihydroxy-3,7-dimethoxy-2-(3-methyl-2-butenyl)-8	C25H28O7	439.1781181	No
8036	HMDB0039915	1,6-Dihydroxy-3,7-dimethoxy-2-(2-hydroxy-3-methyl-3	C25H28O7	439.1781181	No
8036	HMDB0039916	1,6-Dihydroxy-3,7-dimethoxy-2-(3-methyl-2-butenyl)-8	C25H28O7	439.1781181	No
8036	HMDB0039917	1,3-Dihydroxy-6,7-dimethoxy-2-(2-hydroxy-3-methyl-3	C25H28O7	439.1781181	No
8042	HMDB0013961	O-Desmethylverapamil (D-702)	C26H36N2O4	439.2599682	No
8042	HMDB0014246	O-Desmethylverapamil (D-703)	C26H36N2O4	439.2599682	No
8042	HMDB0060540; CHEBI:134080	Norverapamil	C26H36N2O4	439.2599682	No
8046	HMDB0029804	Momordol	C26H48O5	439.3427167	No
8047	HMDB0011559	MG(0:0/24:1(15Z)/0:0)	C27H52O4	439.3788601	No
8047	HMDB0011589	MG(24:1(15Z)/0:0/0:0)	C27H52O4	439.3788601	No
8076	HMDB0034120; C10474	Lusitanicoside	C21H30O10	441.1770658	Yes
8100	HMDB0060769	4-Hydroxyphenytoin glucuronide	C21H20N2O9	443.1098449	No
8102	HMDB0011662; C04840; CHEBI:18134	3-beta-Hydroxy-4-beta-methyl-5-alpha-cholest-7-ene-4	C29H48O3	443.3529933	No
8102	HMDB0012165	4alpha-Carboxy-4beta-methyl-5alpha-cholesta-8-en-3b	C29H48O3	443.3529933	No
8102	HMDB0033633	(3beta,5alpha,6beta,22E,24R)-23-Methylergosta-7,22-	C29H48O3	443.3529933	No
8102	HMDB0035804	Schleicherastatin 5	C29H48O3	443.3529933	No
8102	HMDB0062384; CHEBI:87277	4?-carboxy-4?-methyl-5?-cholesta-8-en-3?-ol	C29H48O3	443.3529933	No
8145	HMDB0030913	Cepagenin	C27H42O5	445.2964406	No
8145	HMDB0031079	12-Ketoporrigenin	C27H42O5	445.2964406	No
8145	HMDB0032680	Neoporrigenin B	C27H42O5	445.2964406	No
8145	HMDB0033583	Leontogenin	C27H42O5	445.2964406	No
8145	HMDB0034189	Taccagenin	C27H42O5	445.2964406	No
8145	HMDB0034424	Spirotaccagenin	C27H42O5	445.2964406	No
8145	HMDB0060129; CHEBI:47813	24-Oxo-1alpha,23,25-trihydroxyvitamin D3	C27H42O5	445.2964406	No
8147	HMDB0012557	13''-Carboxy-gamma-tocopherol	C28H46O4	445.3316134	No
8147	HMDB0032107	(3beta,5alpha,6beta,7alpha,22E,24R)-Ergosta-8,22-die	C28H46O4	445.3316134	No
8147	HMDB0032123	(3beta,5alpha,6alpha,9alpha,22E,24R)-Ergosta-7,22-di	C28H46O4	445.3316134	No
8147	HMDB0032875	3,5,9-Trihydroxyergost-7-en-6-one	C28H46O4	445.3316134	No
8147	HMDB0040999	Secasterone	C28H46O4	445.3316134	No
8147	HMDB0041527	3-Dehydroteasterone	C28H46O4	445.3316134	No
8148	HMDB0012559; CHEBI:84962	13''-Hydroxy-alpha-tocopherol	C29H50O3	445.3682287	No
8148	HMDB0029607	Stigmastentriol	C29H50O3	445.3682287	No
8148	HMDB0034408	alpha-Tocopherolquinone	C29H50O3	445.3682287	No
8148	HMDB0035512	Schleicherastatin 3	C29H50O3	445.3682287	No
8187	HMDB0029209	naringenin-7-O-glucuronide	C21H20O11	447.0935747	No
8187	HMDB0030614; C10114; CHEBI:7781	Orientin	C21H20O11	447.0935747	No
8187	HMDB0030864	Trifolin	C21H20O11	447.0935747	No
8187	HMDB0032903	1,2,6,8-Tetrahydroxy-3-methylanthraquinone 2-O-b-D-	C21H20O11	447.0935747	No

8187	HMDB0033751; C01750; CHEBI:17558	Quercitrin	C21H20O11	447.0935747	No
8187	HMDB0035588; C03951; CHEBI:27994	Luteolin 7-glucoside	C21H20O11	447.0935747	No
8187	HMDB0037359	Azaleatin 3-arabinoside	C21H20O11	447.0935747	No
8187	HMDB0037377; C08598	Carthamone	C21H20O11	447.0935747	No
8187	HMDB0037429; C12249; CHEBI:30200	Astragalin	C21H20O11	447.0935747	No
8187	HMDB0037572	Kaempferol 5-glucoside	C21H20O11	447.0935747	No
8187	HMDB0038467	Luteolin 7-galactoside	C21H20O11	447.0935747	No
8187	HMDB0038468	Luteolin 4''-glucoside	C21H20O11	447.0935747	No
8187	HMDB0041759	Naringenin 4''-O-glucuronide	C21H20O11	447.0935747	No
8187	HMDB0041760	Naringenin 5-O-glucuronide	C21H20O11	447.0935747	No
8187	HMDB0041761; CHEBI:132835	Naringenin 7-O-glucuronide	C21H20O11	447.0935747	No
8187	HMDB0125404	6-[(3,7-dihydroxy-4-oxo-2-phenyl-3,4-dihydro-2H-1-be	C21H20O11	447.0935747	No
8187	HMDB0125405	6-[(3,5-dihydroxy-4-oxo-2-phenyl-3,4-dihydro-2H-1-be	C21H20O11	447.0935747	No
8187	HMDB0125410	6-[3,5-dihydroxy-2-(2-oxo-3-phenylpropanoyl)phenoxy	C21H20O11	447.0935747	No
8187	HMDB0125411	6-[3,5-dihydroxy-4-(2-oxo-3-phenylpropanoyl)phenoxy	C21H20O11	447.0935747	No
8187	HMDB0127132	5,6,7-trihydroxy-2-(4-hydroxyphenyl)-8-[3,4,5-trihydro	C21H20O11	447.0935747	No
8187	HMDB0127356	2-(3,4-dihydroxyphenyl)-5,7-dihydroxy-6-[3,4,5-trihydr	C21H20O11	447.0935747	No
8187	HMDB0127455	3,5,7-trihydroxy-2-(4-{[3,4,5-trihydroxy-6-(hydroxymet	C21H20O11	447.0935747	No
8187	HMDB0127468	3,5-dihydroxy-2-(4-hydroxyphenyl)-7-{[3,4,5-trihydroxy	C21H20O11	447.0935747	No
8187	HMDB0128082	3,4,5-trihydroxy-6-{[7-hydroxy-2-(4-hydroxyphenyl)-4-	C21H20O11	447.0935747	No
8187	HMDB0128083	6-[4-(5,7-dihydroxy-4-oxo-3,4-dihydro-2H-1-benzopyra	C21H20O11	447.0935747	No
8187	HMDB0128085	6-{3,5-dihydroxy-2-[3-(4-hydroxyphenyl)prop-2-enoyl]	C21H20O11	447.0935747	No
8187	HMDB0128086	6-{3,5-dihydroxy-4-[3-(4-hydroxyphenyl)prop-2-enoyl]	C21H20O11	447.0935747	No
8187	HMDB0128087	3,4,5-trihydroxy-6-{4-[3-oxo-3-(2,4,6-trihydroxyphenyl	C21H20O11	447.0935747	No
8187	HMDB0128104	3,4,5-trihydroxy-6-{4-[(1E)-3-oxo-3-(2,4,6-trihydroxyph	C21H20O11	447.0935747	No
8187	HMDB0128105	6-{3,5-dihydroxy-2-[(2E)-3-(4-hydroxyphenyl)prop-2-er	C21H20O11	447.0935747	No
8187	HMDB0129299	5,6,7-trihydroxy-2-(3-hydroxyphenyl)-8-[3,4,5-trihydro	C21H20O11	447.0935747	No
8187	HMDB0129977	3,4,5-trihydroxy-6-{[7-hydroxy-3-(4-hydroxyphenyl)-4-	C21H20O11	447.0935747	No
8187	HMDB0129978	3,4,5-trihydroxy-6-{[5-hydroxy-3-(4-hydroxyphenyl)-4-	C21H20O11	447.0935747	No
8187	HMDB0129979	6-[4-(5,7-dihydroxy-4-oxo-3,4-dihydro-2H-1-benzopyra	C21H20O11	447.0935747	No
8187	HMDB0130011	6-[(5,7-dihydroxy-4-oxo-3-phenyl-3,4-dihydro-2H-1-be	C21H20O11	447.0935747	No
8187	HMDB0130012	6-[(6,7-dihydroxy-4-oxo-3-phenyl-3,4-dihydro-2H-1-be	C21H20O11	447.0935747	No
8187	HMDB0130013	6-[(5,6-dihydroxy-4-oxo-3-phenyl-3,4-dihydro-2H-1-be	C21H20O11	447.0935747	No
8187	HMDB0130214	6-[(7,8-dihydroxy-4-oxo-2-phenyl-3,4-dihydro-2H-1-be	C21H20O11	447.0935747	No
8187	HMDB0130215	6-[(5,8-dihydroxy-4-oxo-2-phenyl-3,4-dihydro-2H-1-be	C21H20O11	447.0935747	No
8187	HMDB0130245	3,4,5-trihydroxy-6-[3,4,5-trihydroxy-2-(3-phenylprop-2	C21H20O11	447.0935747	No
8187	HMDB0130246	3,4,5-trihydroxy-6-[2,3,5-trihydroxy-4-(3-phenylprop-2	C21H20O11	447.0935747	No
8187	HMDB0130247	3,4,5-trihydroxy-6-[2,4,6-trihydroxy-3-(3-phenylprop-2	C21H20O11	447.0935747	No
8187	HMDB0130248	3,4,5-trihydroxy-6-[2,3,5-trihydroxy-6-(3-phenylprop-2	C21H20O11	447.0935747	No
8187	HMDB0132732	3-{[3,4-dihydroxy-5-(hydroxymethyl)oxolan-2-yl]oxy}-5	C21H20O11	447.0935747	No
8187	HMDB0133384	3,4,5-trihydroxy-6-{[7-hydroxy-2-(3-hydroxyphenyl)-4-	C21H20O11	447.0935747	No
8187	HMDB0133385	3,4,5-trihydroxy-6-{[5-hydroxy-2-(3-hydroxyphenyl)-4-	C21H20O11	447.0935747	No
8187	HMDB0133386	6-[3-(5,7-dihydroxy-4-oxo-3,4-dihydro-2H-1-benzopyra	C21H20O11	447.0935747	No
8187	HMDB0133578	6-[(2-benzyl-2,6-dihydroxy-3-oxo-2,3-dihydro-1-benzof	C21H20O11	447.0935747	No
8187	HMDB0133579	6-[(2-benzyl-2,4-dihydroxy-3-oxo-2,3-dihydro-1-benzof	C21H20O11	447.0935747	No
8187	HMDB0134576	3,4,5-trihydroxy-6-{[8-hydroxy-2-(4-hydroxyphenyl)-4-	C21H20O11	447.0935747	No
8187	HMDB0134577	6-[4-(5,8-dihydroxy-4-oxo-3,4-dihydro-2H-1-benzopyra	C21H20O11	447.0935747	No
8187	HMDB0134898	6-[(6,7-dihydroxy-4-oxo-2-phenyl-3,4-dihydro-2H-1-be	C21H20O11	447.0935747	No
8187	HMDB0134899	6-[(5,7-dihydroxy-4-oxo-2-phenyl-3,4-dihydro-2H-1-be	C21H20O11	447.0935747	No
8187	HMDB0134900	6-[(5,6-dihydroxy-4-oxo-2-phenyl-3,4-dihydro-2H-1-be	C21H20O11	447.0935747	No
8187	HMDB0134901	3,4,5-trihydroxy-6-{2,3,5-trihydroxy-4-[(2E)-3-phenylpr	C21H20O11	447.0935747	No
8187	HMDB0135149	2-(3,4-dihydroxyphenyl)-5,7-dihydroxy-3-[(3,4,5-trihyd	C21H20O11	447.0935747	No
8188	HMDB0029481	Isosakuranin	C22H24O10	447.1287678	Yes
8188	HMDB0031586	7-Hydroxy-8-O-methylaloin B	C22H24O10	447.1287678	Yes
8188	HMDB0033741	Puddumin A	C22H24O10	447.1287678	Yes
8188	HMDB0037509	Chalconosakuranetin	C22H24O10	447.1287678	Yes
8188	HMDB0040561	Aromadendrin 4''-methyl ether 7-rhamnoside	C22H24O10	447.1287678	Yes
8188	HMDB0041536	Piperenol C	C22H24O10	447.1287678	Yes
8192	HMDB0029347	Acuminoside	C21H36O10	447.2232686	Yes
8192	HMDB0029348	Neryl arabinofuranosyl-glucoside	C21H36O10	447.2232686	Yes
8192	HMDB0030421	L-Linalool 3-[xylosyl-(1->6)-glucoside]	C21H36O10	447.2232686	Yes

8192	HMDB0039573	Geranyl arabinopyranosyl-glucoside	C21H36O10	447.2232686	Yes
8192	HMDB0039749	Kenposide B	C21H36O10	447.2232686	Yes
8200	HMDB0032783	Porrigenin A	C27H44O5	447.3105116	No
8200	HMDB0034293	Asperagenin	C27H44O5	447.3105116	No
8201	HMDB0034332	6-Deoxodolichosterone	C28H48O4	447.3470666	No
8201	HMDB0034423; C15793	2-Deoxycastasterone	C28H48O4	447.3470666	No
8203	HMDB0030014	(3beta,5alpha,6beta,24R)-Stigmastane-3,5,6-triol	C29H52O3	447.3838318	No
8218	HMDB0039900	Thesinine 4''-O-glucoside	C23H31NO8	448.1961224	Yes
8219	HMDB0039857	Cadabicine methyl ether	C26H31N3O4	448.2247783	Yes
8227	HMDB0000631; C05464; CHEBI:27471	Deoxycholic acid glycine conjugate	C26H43NO5	448.3071816	No
8227	HMDB0000637; C05466	Chenodeoxycholic acid glycine conjugate	C26H43NO5	448.3071816	No
8227	HMDB0000708; CHEBI:542261	Glycoursodeoxycholic acid	C26H43NO5	448.3071816	No
8227	HMDB0006898; C05462	Chenodeoxyglycocholic acid	C26H43NO5	448.3071816	No
8257	HMDB0002385; CHEBI:63959	Celastrol	C29H38O4	449.268729	No
8263	HMDB0033984; C15802; CHEBI:20712	6-Deoxocastasterone	C28H50O4	449.3624381	No
8271	HMDB0011474	LysoPE(0:0/16:1(9Z))	C21H42NO7P	450.2623314	No
8271	HMDB0011504	LysoPE(16:1(9Z)/0:0)	C21H42NO7P	450.2623314	No
8271	HMDB0035366	Cytochalasin Opho	C28H37NO4	450.2623314	No
8293	HMDB0094682; CHEBI:133101	5alpha-Androstan-3alpha,17beta-diol disulfate	C19H32O8S2	451.1469024	Yes
8297	HMDB0015048; C07670; CHEBI:348669	Repaglinide	C27H36N2O4	451.2595026	No
8301	HMDB0000483	5b-Cholestane-3a,7a,12a,23S,25-pentol	C27H48O5	451.342471	No
8301	HMDB0000513	5b-Cholestane-3a,7a,12a,23R,25-pentol	C27H48O5	451.342471	No
8301	HMDB0000556	5b-Cholestane-3a,7a,12a,24,25-pentol	C27H48O5	451.342471	No
8301	HMDB0000558	5a-Cholestane-3a,7a,12a,23,25-pentol	C27H48O5	451.342471	No
8301	HMDB0002180	5b-Cholestane-3a,7a,12a,25,26-pentol	C27H48O5	451.342471	No
8301	HMDB0002208	Cholestane-3,7,12,24,25-pentol	C27H48O5	451.342471	No
8301	HMDB0062416; CHEBI:63835	5beta-cholestan-3alpha,7alpha,12alpha,24(S),27-pent	C27H48O5	451.342471	No
8301	HMDB0062421	5beta-cholestane-3alpha,7alpha,12alpha,23,25-pentol	C27H48O5	451.342471	No
8307	HMDB0060858	o-O-sulfate rosiglitazone	C18H19N3O7S2	452.0613935	No
8309	HMDB0031948	1-Isobutanol	C21H27NO10	452.1557936	No
8309	HMDB0061140	Endoxifen sulfate	C25H27NO5S	452.1557936	No
8313	HMDB0011473; CHEBI:131743	LysoPE(0:0/16:0)	C21H44NO7P	452.2755518	No
8313	HMDB0011503; CHEBI:73004	LysoPE(16:0/0:0)	C21H44NO7P	452.2755518	No
8344	HMDB0002157	27-Norcholestanehexol	C26H46O6	453.3227965	No
8380	HMDB0036883	Citranaxanthin	C33H44O	455.3310842	No
8381	HMDB0002364; C17148; CHEBI:37659	Oleanolic acid	C30H48O3	455.3504322	No
8381	HMDB0002388; CHEBI:499685	Boswellic acid	C30H48O3	455.3504322	No
8381	HMDB0002395; C08988; CHEBI:9908	Ursolic acid	C30H48O3	455.3504322	No
8381	HMDB0030094; C08619	Betulinic acid	C30H48O3	455.3504322	No
8381	HMDB0031882	Sandosapogenol	C30H48O3	455.3504322	No
8381	HMDB0032256	(+-)-Ethyl 3-hydroxy-2-methylbutyrate	C30H48O3	455.3504322	No
8381	HMDB0033020	Ganodermanondiol	C30H48O3	455.3504322	No
8381	HMDB0034652; C17420	Soyasapogenol E	C30H48O3	455.3504322	No
8381	HMDB0034654	Katonic acid	C30H48O3	455.3504322	No
8381	HMDB0034655	3-Epikatonic acid	C30H48O3	455.3504322	No
8381	HMDB0034961	beta-Elemolic acid	C30H48O3	455.3504322	No
8381	HMDB0034962	3alpha-3-Hydroxytirucalla-7,24-dien-21-oic acid	C30H48O3	455.3504322	No
8381	HMDB0034964; C08623	alpha-Elemolic acid	C30H48O3	455.3504322	No
8381	HMDB0035119	Isomangiferolic acid	C30H48O3	455.3504322	No
8381	HMDB0035263	Oxyallobetulin	C30H48O3	455.3504322	No
8381	HMDB0035315	Ganodermatriol	C30H48O3	455.3504322	No
8381	HMDB0035353	Epoxyganoderiol C	C30H48O3	455.3504322	No
8381	HMDB0035511	Trametenolic acid B	C30H48O3	455.3504322	No
8381	HMDB0035775	11-Deoxoglycyrrhetinic acid	C30H48O3	455.3504322	No
8381	HMDB0036675	3beta-Hydroxy-28,13-ursanolide	C30H48O3	455.3504322	No
8381	HMDB0036757	Bryonolic acid	C30H48O3	455.3504322	No
8381	HMDB0036962	3-Epioleanolic acid	C30H48O3	455.3504322	No
8381	HMDB0038177	3-Hydroxycycloart-24-en-21-oic acid	C30H48O3	455.3504322	No
8381	HMDB0038703; CHEBI:95136	Carissic acid	C30H48O3	455.3504322	No
8381	HMDB0039690	Glycyrrhetol	C30H48O3	455.3504322	No
8381	HMDB0040455	Lucidadiol	C30H48O3	455.3504322	No

8381	HMDB0040456	Ganoderic acid Z	C30H48O3	455.3504322	No
8381	HMDB0040652	3-Hydroxy-28,13-lupanolide	C30H48O3	455.3504322	No
8381	HMDB0040976	3-Epimasticadienolic acid	C30H48O3	455.3504322	No
8410	HMDB0033728	Pubesenolide	C28H42O5	457.2950862	No
8442	HMDB0033138	Methyl salicylate O-[rhamnosyl-(1->6)-glucoside]	C20H28O12	459.1510312	No
8445	HMDB0030909	2,3-Secoporrigenin	C27H40O6	459.2731869	No
8445	HMDB0038352	Lucidenic acid N	C27H40O6	459.2731869	No
8449	HMDB0032879	all-trans-Carophyll yellow	C32H44O2	459.3265787	No
8465	HMDB0015194; C06839; CHEBI:2956	Azlocillin	C20H23N5O6S	460.1287946	No
8467	HMDB0060934	3-cis-Hydroxyglipizide	C21H27N5O5S	460.1662402	No
8467	HMDB0060935	4-trans-Hydroxyglipizide	C21H27N5O5S	460.1662402	No
8501	HMDB0034430	6-Deoxohomodolichosterone	C29H50O4	461.3620339	No
8501	HMDB0039713	(3beta,22R,23R,24S)-3,22,23-Trihydroxystigmastan-6-c	C29H50O4	461.3620339	No
8512	HMDB0060820	Dihydroisomorphine-3-glucuronide	C23H29NO9	462.1768537	No
8512	HMDB0060821	Dihydromorphine-3-glucuronide	C23H29NO9	462.1768537	No
8512	HMDB0060916	Etodolac acyl glucuronide	C23H29NO9	462.1768537	No
8512	HMDB0061137	Dihydroisomorphine-6-glucuronide	C23H29NO9	462.1768537	No
8530	HMDB0030901	Eremopetasitenin B2	C24H32O7S	463.1800416	Yes
8530	HMDB0041532	Eremosulphoxinolide A	C24H32O7S	463.1800416	Yes
8531	HMDB0006766; C05504	Estriol-16-Glucuronide	C24H32O9	463.1988035	Yes
8531	HMDB0010333	Estriol-17-glucuronide	C24H32O9	463.1988035	Yes
8531	HMDB0010335	Estriol-3-glucuronide	C24H32O9	463.1988035	Yes
8531	HMDB0010353; C03033	15-Hydroxynorandrostene-3,17-dione glucuronide	C24H32O9	463.1988035	Yes
8531	HMDB0010354; C11346	16-alpha,17-beta-estriol 17-beta-D-glucuronide	C24H32O9	463.1988035	Yes
8532	HMDB0031367	Linalool oxide D 3-[apiosyl-(1->6)-glucoside]	C21H36O11	463.2178953	No
8532	HMDB0035489	Linalool 3,6-oxide primeveroside	C21H36O11	463.2178953	No
8532	HMDB0036571	Linalool 3,7-oxide beta-primeveroside	C21H36O11	463.2178953	No
8535	HMDB0003193; C11134; CHEBI:28835	Testosterone glucuronide	C25H36O8	463.2340072	Yes
8535	HMDB0010327; C03033	Dehydroisoandrosterone 3-glucuronide	C25H36O8	463.2340072	Yes
8535	HMDB0010348; CHEBI:68834	Dehydroepiandrosterone 3-glucuronide	C25H36O8	463.2340072	Yes
8535	HMDB0029655	Erinacine G	C25H36O8	463.2340072	Yes
8546	HMDB0002163; CHEBI:89753	Trihydroxycoprostanoic acid	C28H48O5	463.3431772	No
8546	HMDB0041135	2-Deoxybrassinolide	C28H48O5	463.3431772	No
8557	HMDB0003323	Biotripyrrin-a	C25H27N3O6	464.1830771	Yes
8557	HMDB0003324	Biotripyrrin-b	C25H27N3O6	464.1830771	Yes
8566	HMDB0010380; C04230	LysoPC(14:1(9Z))	C22H44NO7P	464.2776864	No
8568	HMDB0000138; C01921; CHEBI:29746	Glycocholic acid	C26H43NO6	464.3020993	Yes
8568	HMDB0000331	3a,7b,12a-Trihydroxyoxocholanyl-Glycine	C26H43NO6	464.3020993	Yes
8568	HMDB0032596; CHEBI:36273	Sodium glycocholate	C26H43NO6	464.3020993	Yes
8588	HMDB0036600; C09738	T2 Toxin	C24H34O9	465.2120973	Yes
8588	HMDB0038558	8-Pentanoylneosolaniol	C24H34O9	465.2120973	Yes
8590	HMDB0001054; C01246; CHEBI:15812	Dolichyl b-D-glucosyl phosphate	C21H39O9P	465.2238973	Yes
8593	HMDB0002829; C11135; CHEBI:28832	Androsterone glucuronide	C25H38O8	465.2500602	No
8593	HMDB0004484; C11136; CHEBI:37451	Etiocholanolone glucuronide	C25H38O8	465.2500602	No
8593	HMDB0006203; CHEBI:89417	5-alpha-Dihydrotestosterone glucuronide	C25H38O8	465.2500602	No
8593	HMDB0010365	3-alpha-hydroxy-5-alpha-androstane-17-one 3-D-glucu	C25H38O8	465.2500602	No
8608	HMDB0031949	Bis-N-butyl phthalate	C22H29NO10	466.1718498	No
8613	HMDB0042042	Tiropramide	C28H41N3O3	466.3077374	No
8635	HMDB0010321	3,17-Androstanediol glucuronide	C25H40O8	467.2639628	Yes
8635	HMDB0010339	3-alpha-Androstanediol glucuronide	C25H40O8	467.2639628	Yes
8635	HMDB0010359	17-Hydroxyandrostane-3-glucuronide	C25H40O8	467.2639628	Yes
8639	HMDB0035806	psi-Taraxasteryl acetate	C32H52O2	467.3901914	No
8639	HMDB0036658	alpha-Amyrin acetate	C32H52O2	467.3901914	No
8639	HMDB0036960	Urs-13(18)-en-3beta-yl acetate	C32H52O2	467.3901914	No
8639	HMDB0038062	Isomultiflorenyl acetate	C32H52O2	467.3901914	No
8639	HMDB0040516	Bauerenyl acetate	C32H52O2	467.3901914	No
8675	HMDB0003850; C04549; CHEBI:17283	1-Phosphatidyl-1D-myo-inositol 3-phosphate	C11H20O16P2	469.0135709	Yes
8676	HMDB0037073	(E)-Resveratrol 3-glucoside 4''-sulfate	C20H22O11S	469.078571	No
8676	HMDB0037074	(Z)-Resveratrol 3-glucoside 5-sulfate	C20H22O11S	469.078571	No
8676	HMDB0037075	(Z)-Resveratrol 3-(2''''-sulfoglucoside)	C20H22O11S	469.078571	No
8676	HMDB0037076	(Z)-Resveratrol 3-(4''''-sulfoglucoside)	C20H22O11S	469.078571	No

8676	HMDB0037077	(Z)-Resveratrol 3-(6''''-sulfoglucopyranoside)	C20H22O11S	469.078571	No
8676	HMDB0037078	(Z)-Resveratrol 3-(3''''-sulfoglucoside)	C20H22O11S	469.078571	No
8676	HMDB0128509	{4-[(E)-2-(3-hydroxy-5-{[3,4,5-trihydroxy-6-(hydroxyme	C20H22O11S	469.078571	No
8680	HMDB0029340	Ceanothine C	C26H38N4O4	469.2808253	No
8725	HMDB0001534; C00664; CHEBI:15639	5-Formiminotetrahydrofolic acid	C20H24N8O6	471.1734629	Yes
8725	HMDB0039741	alpha-L-Rhamnopyranosyl-(1->3)-alpha-D-galactopyrar	C18H32O14	471.1734629	Yes
8725	HMDB0127459	6-[({4,5-dihydroxy-6-methyl-3-[(3,4,5-trihydroxy-6-met	C18H32O14	471.1734629	Yes
8752	HMDB0030004	Austalide B	C26H34O8	473.2172048	No
8764	HMDB0034505; C17419	Soyasapogenol A	C30H50O4	473.3628355	No
8764	HMDB0034528	Camelliagenin A	C30H50O4	473.3628355	No
8764	HMDB0034644	Priverogenin B	C30H50O4	473.3628355	No
8764	HMDB0034683	(3alphaOH,20S,24S)-3,19:20,24-Diepoxydammarane-3	C30H50O4	473.3628355	No
8764	HMDB0035326	Ganoderiol A	C30H50O4	473.3628355	No
8764	HMDB0039692	20,24-Epoxy-25,26-dihydroxydammaran-3-one	C30H50O4	473.3628355	No
8772	HMDB0033511	Protogonyautoxin I	C10H17N7O11S2	474.0349255	No
8776	HMDB0060464; C16577	Codeine-6-glucuronide	C24H29NO9	474.1774241	Yes
8779	HMDB0011478	LysoPE(0:0/18:3(6Z,9Z,12Z))	C23H42NO7P	474.2609982	No
8779	HMDB0011479	LysoPE(0:0/18:3(9Z,12Z,15Z))	C23H42NO7P	474.2609982	No
8779	HMDB0011508	LysoPE(18:3(6Z,9Z,12Z)/0:0)	C23H42NO7P	474.2609982	No
8779	HMDB0011509	LysoPE(18:3(9Z,12Z,15Z)/0:0)	C23H42NO7P	474.2609982	No
8782	HMDB0014907; CHEBI:50851	Hydrocortamate	C27H41NO6	474.2864357	No
8796	HMDB0037452	Diosmetin 7-O-beta-D-glucuronopyranoside	C22H20O12	475.0870165	Yes
8796	HMDB0124901	3,4,5-trihydroxy-6-{[5-hydroxy-2-(3-hydroxy-5-methox	C22H20O12	475.0870165	Yes
8796	HMDB0124902	3,4,5-trihydroxy-6-{[7-hydroxy-2-(3-hydroxy-5-methox	C22H20O12	475.0870165	Yes
8796	HMDB0124903	6-[3-(5,7-dihydroxy-4-oxo-4H-chromen-2-yl)-5-methox	C22H20O12	475.0870165	Yes
8796	HMDB0126583	6-{[3,5-dihydroxy-2-(3-methoxyphenyl)-4-oxo-4H-chro	C22H20O12	475.0870165	Yes
8796	HMDB0126584	6-{[3,7-dihydroxy-2-(3-methoxyphenyl)-4-oxo-4H-chro	C22H20O12	475.0870165	Yes
8796	HMDB0126585	6-{[5,7-dihydroxy-2-(3-methoxyphenyl)-4-oxo-4H-chro	C22H20O12	475.0870165	Yes
8796	HMDB0127503	6-{[3,5-dihydroxy-2-(4-methoxyphenyl)-4-oxo-4H-chro	C22H20O12	475.0870165	Yes
8796	HMDB0127504	6-{[3,7-dihydroxy-2-(4-methoxyphenyl)-4-oxo-4H-chro	C22H20O12	475.0870165	Yes
8796	HMDB0127505	6-{[5,7-dihydroxy-2-(4-methoxyphenyl)-4-oxo-4H-chro	C22H20O12	475.0870165	Yes
8796	HMDB0128594	3,4,5-trihydroxy-6-{[5-hydroxy-2-(4-hydroxyphenyl)-6-	C22H20O12	475.0870165	Yes
8796	HMDB0128595	3,4,5-trihydroxy-6-{[7-hydroxy-2-(4-hydroxyphenyl)-6-	C22H20O12	475.0870165	Yes
8796	HMDB0128596	6-[4-(5,7-dihydroxy-6-methoxy-4-oxo-4H-chromen-2-y	C22H20O12	475.0870165	Yes
8796	HMDB0128603	3,4,5-trihydroxy-6-{[6-hydroxy-2-(4-hydroxyphenyl)-5-	C22H20O12	475.0870165	Yes
8796	HMDB0128604	3,4,5-trihydroxy-6-{[7-hydroxy-2-(4-hydroxyphenyl)-5-	C22H20O12	475.0870165	Yes
8796	HMDB0128605	6-[4-(6,7-dihydroxy-5-methoxy-4-oxo-4H-chromen-2-y	C22H20O12	475.0870165	Yes
8796	HMDB0129969	6-{[5,6-dihydroxy-3-(4-methoxyphenyl)-4-oxo-4H-chro	C22H20O12	475.0870165	Yes
8796	HMDB0130184	6-{[7,8-dihydroxy-2-(4-methoxyphenyl)-4-oxo-4H-chro	C22H20O12	475.0870165	Yes
8796	HMDB0130185	6-{[5,8-dihydroxy-2-(4-methoxyphenyl)-4-oxo-4H-chro	C22H20O12	475.0870165	Yes
8796	HMDB0133322	3,4,5-trihydroxy-6-{[7-hydroxy-2-(3-hydroxy-4-methox	C22H20O12	475.0870165	Yes
8796	HMDB0133323	3,4,5-trihydroxy-6-{[6-hydroxy-2-(3-hydroxy-4-methox	C22H20O12	475.0870165	Yes
8796	HMDB0133324	6-[5-(6,7-dihydroxy-4-oxo-4H-chromen-2-yl)-2-methox	C22H20O12	475.0870165	Yes
8796	HMDB0133331	3,4,5-trihydroxy-6-[2-hydroxy-6-(7-hydroxy-4-oxo-4H-(	C22H20O12	475.0870165	Yes
8796	HMDB0133332	6-{[2-(2,3-dihydroxy-4-methoxyphenyl)-4-oxo-4H-chro	C22H20O12	475.0870165	Yes
8796	HMDB0133333	3,4,5-trihydroxy-6-[2-hydroxy-3-(7-hydroxy-4-oxo-4H-(	C22H20O12	475.0870165	Yes
8796	HMDB0133336	3,4,5-trihydroxy-6-[4-hydroxy-2-(7-hydroxy-4-oxo-4H-(	C22H20O12	475.0870165	Yes
8796	HMDB0133337	6-{[2-(2,5-dihydroxy-4-methoxyphenyl)-4-oxo-4H-chro	C22H20O12	475.0870165	Yes
8796	HMDB0133338	3,4,5-trihydroxy-6-[4-hydroxy-5-(7-hydroxy-4-oxo-4H-(	C22H20O12	475.0870165	Yes
8796	HMDB0133339	3,4,5-trihydroxy-6-{[7-hydroxy-2-(3-hydroxy-4-methox	C22H20O12	475.0870165	Yes
8796	HMDB0133340	6-[5-(5,7-dihydroxy-4-oxo-4H-chromen-2-yl)-2-methox	C22H20O12	475.0870165	Yes
8796	HMDB0133831	6-[3-(3,5-dihydroxy-7-methoxy-4-oxo-4H-chromen-2-y	C22H20O12	475.0870165	Yes
8796	HMDB0133832	3,4,5-trihydroxy-6-{[5-hydroxy-2-(3-hydroxyphenyl)-7-	C22H20O12	475.0870165	Yes
8796	HMDB0133833	3,4,5-trihydroxy-6-{[3-hydroxy-2-(3-hydroxyphenyl)-7-	C22H20O12	475.0870165	Yes
8796	HMDB0137318	3,4,5-trihydroxy-6-{[5-hydroxy-2-(2-hydroxyphenyl)-6-	C22H20O12	475.0870165	Yes
8799	HMDB0003141; C11061; CHEBI:28870	Retinoyl b-glucuronide	C26H36O8	475.2331566	No
8799	HMDB0030153	Austalide H	C26H36O8	475.2331566	No
8799	HMDB0062632	1-O-all-trans-retinoyl-beta-glucuronic Acid	C26H36O8	475.2331566	No
8803	HMDB0038496	Polyporusterone B	C28H44O6	475.3046666	No
8803	HMDB0038497	Polyporusterone C	C28H44O6	475.3046666	No
8807	HMDB0038244	Panaxatriol	C30H52O4	475.3785157	No

8881	HMDB0010381; C04230; CHEBI:580912	LysoPC(15:0)	C23H48NO7P	480.3086309	No
8881	HMDB0011129; CHEBI:133144	LysoPE(0:0/18:0)	C23H48NO7P	480.3086309	No
8881	HMDB0011130; CHEBI:75036	LysoPE(18:0/0:0)	C23H48NO7P	480.3086309	No
8904	HMDB0010351	11-beta-Hydroxyandrosterone-3-glucuronide	C25H38O9	481.2442329	Yes
8940	HMDB0038539	Goshonoside F1	C26H44O8	483.2940052	No
8940	HMDB0038540	Goshonoside F2	C26H44O8	483.2940052	No
8970	HMDB0000585; CHEBI:89573	Glucosylgalactosyl hydroxylysine	C18H34N2O13	485.1989447	Yes
8976	HMDB0011156	PA(20:4(5Z,8Z,11Z,14Z)e/2:0)	C25H43O7P	485.2678343	No
8976	HMDB0062310	1-(7Z,10Z,13Z,16Z-docosatetraenoyl)-glycero-3-phospl	C25H43O7P	485.2678343	No
8976	HMDB0114752	LPA(22:4(7Z,10Z,13Z,16Z)/0:0)	C25H43O7P	485.2678343	No
9005	HMDB0034036	Pitheduloside I	C30H48O5	487.3434494	No
9005	HMDB0034502	Arjunolic acid	C30H48O5	487.3434494	No
9005	HMDB0034531	Camelliagenin B	C30H48O5	487.3434494	No
9005	HMDB0035118	Madasiatic acid	C30H48O5	487.3434494	No
9005	HMDB0035782	Esculentic acid (Diplazium)	C30H48O5	487.3434494	No
9005	HMDB0035957	Hovenolactone	C30H48O5	487.3434494	No
9005	HMDB0036311	Centellasapogenol A	C30H48O5	487.3434494	No
9005	HMDB0036639	Glyyunnansapogenin B	C30H48O5	487.3434494	No
9005	HMDB0036651; CHEBI:67914	Euscaphic acid	C30H48O5	487.3434494	No
9005	HMDB0036961	(3beta,6alpha,19alpha)-3,6,19-Trihydroxy-12-ursen-28	C30H48O5	487.3434494	No
9005	HMDB0037782	Ganoderiol D	C30H48O5	487.3434494	No
9005	HMDB0038116; C12599	Ananasic acid	C30H48O5	487.3434494	No
9005	HMDB0040391	16beta-Hydroxystellatogenin	C30H48O5	487.3434494	No
9005	HMDB0041039	21beta-Hydroxyhederagenin	C30H48O5	487.3434494	No
9011	HMDB0060763	4-Hydroxy duloxetine glucuronide	C24H27NO8S	488.1383843	No
9034	HMDB0128306	3-[3,4-dihydroxy-5-(3,4,5-trihydroxybenzoyloxy)benzo	C21H14O14	489.0301876	No
9034	HMDB0128307	3,4-dihydroxy-5-[2,4,5-trihydroxy-3-(3,4,5-trihydroxyb	C21H14O14	489.0301876	No
9034	HMDB0128308	3-[3,4-dihydroxy-5-(2,3,4,5-tetrahydroxybenzoyloxy)be	C21H14O14	489.0301876	No
9036	HMDB0040512	4'',5,6-Trimethylscutellarein 7-glucoside	C24H26O11	489.1402969	No
9037	HMDB0034434	Austalide F	C26H34O9	489.2126348	Yes
9037	HMDB0035073	Deacetylnomilinic acid	C26H34O9	489.2126348	Yes
9037	HMDB0039120	alpha-Crocetin glucosyl ester	C26H34O9	489.2126348	Yes
9037	HMDB0060122	4-Oxo-9-cis-retinoyl-beta-glucuronide	C26H34O9	489.2126348	Yes
9037	HMDB0061687	Estradiol acetate glucuronide	C26H34O9	489.2126348	Yes
9037	HMDB0061689	4-Ketoretinoic acid glucuronide	C26H34O9	489.2126348	Yes
9039	HMDB0060118	12-Hydroxy-13-O-D-glucuronoside-octadec-9Z-enoate	C24H42O10	489.2708149	No
9039	HMDB0060119	12-O-D-Glucuronoside-13-hydroxyoctadec-9Z-enoate	C24H42O10	489.2708149	No
9039	HMDB0060120	10-Hydroxy-octadec-12Z-enoate-9-beta-D-glucuronide	C24H42O10	489.2708149	No
9039	HMDB0060121	9-Hydroxy-10-O-D-glucuronoside-12Z-octadecenoate	C24H42O10	489.2708149	No
9041	HMDB0062306	1-(13Z,16Z-docosadienoyl)-glycero-3-phosphate	C25H47O7P	489.2981378	No
9041	HMDB0114751	LPA(22:2(13Z,16Z)/0:0)	C25H47O7P	489.2981378	No
9044	HMDB0034525; C08931	Barringtogenol C	C30H50O5	489.3579199	No
9044	HMDB0034532	Camelliagenin C	C30H50O5	489.3579199	No
9044	HMDB0037783	Ganoderiol H	C30H50O5	489.3579199	No
9079	HMDB0036313	Erinacine P	C27H40O8	491.2647578	No
9082	HMDB0062304	1-(11Z-docosenoyl)-glycero-3-phosphate	C25H49O7P	491.3150739	No
9082	HMDB0114750	LPA(22:1(13Z)/0:0)	C25H49O7P	491.3150739	No
9114	HMDB0033655	7,11-Bisdeacetylvaltrate 7-(3-methylpentanoate) 11-(3	C26H38O9	493.2446853	No
9144	HMDB0038680	Musabalbisiane A	C23H28O12	495.1512171	No
9144	HMDB0039026	(R)-Isobyakangelicin 3''-glucoside	C23H28O12	495.1512171	No
9144	HMDB0039069	(R)-Byakangelicin 3''-glucoside	C23H28O12	495.1512171	No
9144	HMDB0039070	(R)-Byakangelicin 2''-glucoside	C23H28O12	495.1512171	No
9144	HMDB0060491; CHEBI:64689	Mycophenolic acid O-acyl-glucuronide	C23H28O12	495.1512171	No
9144	HMDB0060634	Mycophenolic acid glucuronide	C23H28O12	495.1512171	No
9144	HMDB0130138	3,4,5-trihydroxy-6-{[8-(2-hydroxypropan-2-yl)-4-(1-hyd	C23H28O12	495.1512171	No
9144	HMDB0130140	3,4,5-trihydroxy-6-{[8-(2-hydroxypropan-2-yl)-4-(2-hyd	C23H28O12	495.1512171	No
9144	HMDB0130142	6-{[8-(1,2-dihydroxypropan-2-yl)-2-oxo-4-propyl-2H,8H	C23H28O12	495.1512171	No
9144	HMDB0130144	3,4,5-trihydroxy-6-{[8-(2-hydroxypropan-2-yl)-4-(3-hyd	C23H28O12	495.1512171	No
9145	HMDB0010318; C03033	Pregnanediol-3-glucuronide	C27H44O8	495.2962419	No
9145	HMDB0010352	3-alpha,20-alpha-Dihydroxy-5-beta-pregnane 3-glucur	C27H44O8	495.2962419	No
9145	HMDB0041331	(3b,16b,20R)-Pregn-5-ene-3,16,20-triol 3-glucoside	C27H44O8	495.2962419	No

9178	HMDB0029291	3,4-Dicaffeoyl-1,5-quinolactone	C25H22O11	497.1093131	Yes
9195	HMDB0011489	LysoPE(0:0/20:5(5Z,8Z,11Z,14Z,17Z))	C25H42NO7P	498.2627138	No
9195	HMDB0011519	LysoPE(20:5(5Z,8Z,11Z,14Z,17Z)/0:0)	C25H42NO7P	498.2627138	No
9214	HMDB0030127	Withaperuvin E	C28H36O8	499.2338544	No
9214	HMDB0041779	Tigloylgomicin H	C28H36O8	499.2338544	No
9222	HMDB0014554; C11758; CHEBI:135271	Mupirocin	C26H44O9	499.2908018	No
9223	HMDB0005770; CHEBI:88947	Tuftsin	C21H40N8O6	499.2989644	No
9223	HMDB0005776	Kentsin	C21H40N8O6	499.2989644	No
9224	HMDB0033234	Ganoderic acid beta	C30H44O6	499.3061743	No
9224	HMDB0034200	Physalolactone B	C30H44O6	499.3061743	No
9224	HMDB0035299	Ganolucidic acid D	C30H44O6	499.3061743	No
9224	HMDB0035302	Ganolucidic acid A	C30H44O6	499.3061743	No
9234	HMDB0005030; C06999; CHEBI:5050	Fexofenadine	C32H39NO4	500.2802949	No
9251	HMDB0125131	3,4,5-trihydroxy-6-{4-[(1E)-3-oxo-3-[(3,4,5,6-tetrahydr	C21H26O14	501.1243155	Yes
9251	HMDB0129614	({1-[2-(2,4-dihydroxyphenyl)-7-hydroxy-4-oxo-4H-chro	C25H26O9S	501.1243155	Yes
9251	HMDB0129618	{[(2Z)-2-{2-[2-(2,4-dihydroxyphenyl)-7-hydroxy-4-oxo-4	C25H26O9S	501.1243155	Yes
9251	HMDB0129622	({8-[2-(2,4-dihydroxyphenyl)-7-hydroxy-4-oxo-4H-chro	C25H26O9S	501.1243155	Yes
9251	HMDB0129626	{[(2E)-8-[2-(2,4-dihydroxyphenyl)-7-hydroxy-4-oxo-4H-	C25H26O9S	501.1243155	Yes
9251	HMDB0129630	({8-[2-(2,4-dihydroxyphenyl)-7-hydroxy-4-oxo-4H-chro	C25H26O9S	501.1243155	Yes
9251	HMDB0129650	{3-[3-(3,7-dimethylocta-2,6-dien-1-yl)-7-hydroxy-4-oxo	C25H26O9S	501.1243155	Yes
9251	HMDB0134742	{4-[5,7-dihydroxy-8-(3-methylbut-2-en-1-yl)-4-oxo-4H-	C25H26O9S	501.1243155	Yes
9253	HMDB0034551	Medicagenic acid	C30H46O6	501.3218149	No
9253	HMDB0034639	Esculentic acid (Phytolacca)	C30H46O6	501.3218149	No
9253	HMDB0034704	22-Deoxoisocucurbitacin D	C30H46O6	501.3218149	No
9253	HMDB0034705	22-Deoxocucurbitacin D	C30H46O6	501.3218149	No
9253	HMDB0035751	Ganolucidic acid B	C30H46O6	501.3218149	No
9264	HMDB0011484	LysoPE(0:0/20:3(11Z,14Z,17Z))	C25H46NO7P	502.2933162	No
9264	HMDB0011485; CHEBI:90579	LysoPE(0:0/20:3(5Z,8Z,11Z))	C25H46NO7P	502.2933162	No
9264	HMDB0011486	LysoPE(0:0/20:3(8Z,11Z,14Z))	C25H46NO7P	502.2933162	No
9264	HMDB0011514	LysoPE(20:3(11Z,14Z,17Z)/0:0)	C25H46NO7P	502.2933162	No
9264	HMDB0011515	LysoPE(20:3(5Z,8Z,11Z)/0:0)	C25H46NO7P	502.2933162	No
9264	HMDB0011516	LysoPE(20:3(8Z,11Z,14Z)/0:0)	C25H46NO7P	502.2933162	No
9296	HMDB0001512; C03028; CHEBI:9534	Thiamine triphosphate	C12H20N4O10P3S	504.0049872	No
9329	HMDB0001134; C00053; CHEBI:17980	Phosphoadenosine phosphosulfate	C10H15N5O13P2S	505.9806883	Yes
9351	HMDB0034700	1,2,4,8-Tetraacetoxy-7-hydroxy-3-(4-hydroxyphenyl)di	C26H20O11	507.095558	No
9351	HMDB0034701	1,2,4,7-Tetraacetoxy-8-hydroxy-3-(4-hydroxyphenyl)di	C26H20O11	507.095558	No
9351	HMDB0039566	BL III	C26H20O11	507.095558	No
9354	HMDB0033164	Acetyl-T2 Toxin	C26H36O10	507.2228851	No
9354	HMDB0037002	Mozambioside	C26H36O10	507.2228851	No
9354	HMDB0037003	Cafamarine	C26H36O10	507.2228851	No
9354	HMDB0038611	Gibberellin A37 glucosyl ester	C26H36O10	507.2228851	No
9355	HMDB0014423; C04931	Argatroban	C23H36N6O5S	507.2377032	Yes
9355	HMDB0129722	2-(2,4-dihydroxyphenyl)-3-(3,7-dimethylocta-2,6-dien-	C30H36O7	507.2377032	Yes
9355	HMDB0129723	2-(2,4-dihydroxyphenyl)-5,7-dihydroxy-3-(4-hydroxy-3,	C30H36O7	507.2377032	Yes
9355	HMDB0129724	2-(2,4-dihydroxyphenyl)-5,7-dihydroxy-3-[4-hydroxy-3-	C30H36O7	507.2377032	Yes
9355	HMDB0129725	2-(2,4-dihydroxyphenyl)-5,7-dihydroxy-3-(8-hydroxy-3,	C30H36O7	507.2377032	Yes
9355	HMDB0129726	2-(2,4-dihydroxyphenyl)-5,7-dihydroxy-3-[(6E)-8-hydro	C30H36O7	507.2377032	Yes
9355	HMDB0129727	2-(2,4-dihydroxyphenyl)-5,7-dihydroxy-3-(5-hydroxy-3,	C30H36O7	507.2377032	Yes
9355	HMDB0129728	2-(2,4-dihydroxyphenyl)-3-(3,7-dimethylocta-2,6-dien-	C30H36O7	507.2377032	Yes
9355	HMDB0129729	2-(2,4-dihydroxyphenyl)-5,7-dihydroxy-3-{[3-methyl-3-	C30H36O7	507.2377032	Yes
9355	HMDB0129730	2-(2,4-dihydroxyphenyl)-3-[5-(3,3-dimethyloxiran-2-yl)	C30H36O7	507.2377032	Yes
9355	HMDB0129731	3-(3,7-dimethylocta-2,6-dien-1-yl)-5,7-dihydroxy-6-(3-r	C30H36O7	507.2377032	Yes
9358	HMDB0115485	PA(8:0/14:0)	C25H49O8P	507.3088697	No
9358	HMDB0115611	PA(10:0/i-12:0)	C25H49O8P	507.3088697	No
9358	HMDB0115687	PA(8:0/i-14:0)	C25H49O8P	507.3088697	No
9361	HMDB0035853	Fasciculol C	C30H52O6	507.3682039	No
9386	HMDB0127320	{5-[6-(3,4-dihydroxy-6-methyl-5-oxooxan-2-yl)-5,7-dihy	C21H18O13S	509.0379599	No
9386	HMDB0127321	{2-[2-(3,4-dihydroxyphenyl)-5,7-dihydroxy-4-oxo-4H-cl	C21H18O13S	509.0379599	No
9386	HMDB0127322	{2-[2-(3,4-dihydroxyphenyl)-5,7-dihydroxy-4-oxo-4H-cl	C21H18O13S	509.0379599	No
9388	HMDB0041535	Piperenol A triacetate	C27H26O10	509.1430499	No
9402	HMDB0013863	AFN911	C29H33N7O2	510.2599005	No

9419	HMDB0035980	3,7,11,15,23-Pentaoxolanost-8-en-26-oic acid	C30H40O7	511.270531	Yes
9419	HMDB0036059	Ganoderenic acid D	C30H40O7	511.270531	Yes
9419	HMDB0036406	Ganosporelactone A	C30H40O7	511.270531	Yes
9419	HMDB0036414	Anabsin	C30H40O7	511.270531	Yes
9429	HMDB0127805	4-{7-[(6-carboxy-3,4,5-trihydroxyoxan-2-yl)oxy]-3-hydr	C21H21O13S	512.0631414	No
9429	HMDB0127806	2-{3-[(6-carboxy-3,4,5-trihydroxyoxan-2-yl)oxy]-4-hydr	C21H21O13S	512.0631414	No
9429	HMDB0127902	2-[(6-carboxy-3,4,5-trihydroxyoxan-2-yl)oxy]-4-(3,5-dih	C21H21O13S	512.0631414	No
9433	HMDB0032880	trans-Zeatin-O-glucoside riboside	C21H31N5O10	512.1987996	No
9433	HMDB0036593	Validamycin B	C20H35NO14	512.1987996	No
9457	HMDB0035079	(7alpha,10beta)-1(10->19)-Abeo-7-acetoxyisoobacun-3	C28H34O9	513.2123219	No
9457	HMDB0035772; C08773	Nomilin	C28H34O9	513.2123219	No
9464	HMDB0030737	Capsianoside V	C26H42O10	513.2706698	Yes
9464	HMDB0034677	Cinncassiol D1 glucoside	C26H42O10	513.2706698	Yes
9464	HMDB0035065	Cinncassiol D4 2-glucoside	C26H42O10	513.2706698	Yes
9464	HMDB0035096	Cofaryloside	C26H42O10	513.2706698	Yes
9483	HMDB0000036; C05122; CHEBI:28865	Taurocholic acid	C26H45NO7S	514.2841366	Yes
9483	HMDB0000889	Tauroursocholic acid	C26H45NO7S	514.2841366	Yes
9483	HMDB0000922	Taurallocholic acid	C26H45NO7S	514.2841366	Yes
9483	HMDB0000932	Tauro-b-muricholic acid	C26H45NO7S	514.2841366	Yes
9483	HMDB0011637; C15516	Taurohyocholate	C26H45NO7S	514.2841366	Yes
9504	HMDB0060863	Perindoprilat glucuronide	C23H36N2O11	515.2253028	No
9544	HMDB0034392	Perulactone	C30H46O7	517.3164303	No
9544	HMDB0035304	Ganoderic acid C2	C30H46O7	517.3164303	No
9544	HMDB0035699	Corosin	C30H46O7	517.3164303	No
9544	HMDB0039691	Ganolucidic acid C	C30H46O7	517.3164303	No
9545	HMDB0037781	Ganoderiol C	C32H54O5	517.3892479	No
9574	HMDB0030116	Withaperuvin D	C28H40O9	519.2608488	No
9574	HMDB0034191	Withaperuvin B	C28H40O9	519.2608488	No
9574	HMDB0060141	rac-4-Hydroxy-4-O-(beta-D-glucuronide)-all-trans-retin	C28H40O9	519.2608488	No
9613	HMDB0037182; C11624	Polysorbate 20	C26H50O10	521.3335502	No
9657	HMDB0127110	({6-[5,7-dihydroxy-2-(4-methoxyphenyl)-4-oxo-4H-chro	C22H22O13S	525.0721066	No
9657	HMDB0127111	{2-[5,7-dihydroxy-2-(4-methoxyphenyl)-4-oxo-4H-chro	C22H22O13S	525.0721066	No
9657	HMDB0127112	{2-[5,7-dihydroxy-2-(4-methoxyphenyl)-4-oxo-4H-chro	C22H22O13S	525.0721066	No
9657	HMDB0127113	{6-[5,7-dihydroxy-2-(4-methoxyphenyl)-4-oxo-4H-chro	C22H22O13S	525.0721066	No
9657	HMDB0127139	({3,4,5-trihydroxy-6-[5-hydroxy-2-(4-hydroxyphenyl)-7-	C22H22O13S	525.0721066	No
9657	HMDB0127140	{4,5-dihydroxy-2-[5-hydroxy-2-(4-hydroxyphenyl)-7-me	C22H22O13S	525.0721066	No
9657	HMDB0127141	{3,5-dihydroxy-2-[5-hydroxy-2-(4-hydroxyphenyl)-7-me	C22H22O13S	525.0721066	No
9657	HMDB0127142	{4,5-dihydroxy-6-[5-hydroxy-2-(4-hydroxyphenyl)-7-me	C22H22O13S	525.0721066	No
9657	HMDB0127186	{[6-(5,6-dihydroxy-7-methoxy-4-oxo-2-phenyl-4H-chro	C22H22O13S	525.0721066	No
9657	HMDB0127187	[2-(5,6-dihydroxy-7-methoxy-4-oxo-2-phenyl-4H-chron	C22H22O13S	525.0721066	No
9657	HMDB0127188	[2-(5,6-dihydroxy-7-methoxy-4-oxo-2-phenyl-4H-chron	C22H22O13S	525.0721066	No
9657	HMDB0127189	[6-(5,6-dihydroxy-7-methoxy-4-oxo-2-phenyl-4H-chron	C22H22O13S	525.0721066	No
9657	HMDB0127209	(3-{5-hydroxy-7-methoxy-4-oxo-8-[3,4,5-trihydroxy-6-(	C22H22O13S	525.0721066	No
9657	HMDB0127210	({3,4,5-trihydroxy-6-[5-hydroxy-2-(3-hydroxyphenyl)-7-	C22H22O13S	525.0721066	No
9657	HMDB0127211	{4,5-dihydroxy-2-[5-hydroxy-2-(3-hydroxyphenyl)-7-me	C22H22O13S	525.0721066	No
9657	HMDB0127212	{3,5-dihydroxy-2-[5-hydroxy-2-(3-hydroxyphenyl)-7-me	C22H22O13S	525.0721066	No
9657	HMDB0127213	{4,5-dihydroxy-6-[5-hydroxy-2-(3-hydroxyphenyl)-7-me	C22H22O13S	525.0721066	No
9657	HMDB0127377	({6-[5,7-dihydroxy-2-(3-methoxyphenyl)-4-oxo-4H-chro	C22H22O13S	525.0721066	No
9657	HMDB0127378	{2-[5,7-dihydroxy-2-(3-methoxyphenyl)-4-oxo-4H-chro	C22H22O13S	525.0721066	No
9657	HMDB0127379	{2-[5,7-dihydroxy-2-(3-methoxyphenyl)-4-oxo-4H-chro	C22H22O13S	525.0721066	No
9657	HMDB0127380	{6-[5,7-dihydroxy-2-(3-methoxyphenyl)-4-oxo-4H-chro	C22H22O13S	525.0721066	No
9657	HMDB0128570	(3-{5-hydroxy-7-methoxy-4-oxo-6-[3,4,5-trihydroxy-6-(	C22H22O13S	525.0721066	No
9657	HMDB0128571	({3,4,5-trihydroxy-6-[5-hydroxy-2-(3-hydroxyphenyl)-7-	C22H22O13S	525.0721066	No
9657	HMDB0128572	{4,5-dihydroxy-2-[5-hydroxy-2-(3-hydroxyphenyl)-7-me	C22H22O13S	525.0721066	No
9657	HMDB0128573	{3,5-dihydroxy-2-[5-hydroxy-2-(3-hydroxyphenyl)-7-me	C22H22O13S	525.0721066	No
9657	HMDB0128574	{4,5-dihydroxy-6-[5-hydroxy-2-(3-hydroxyphenyl)-7-me	C22H22O13S	525.0721066	No
9665	HMDB0006744; C05381; CHEBI:1463	3-Carboxy-1-hydroxypropylthiamine diphosphate	C16H25N4O10P2S	526.068897	No
9689	HMDB0040647	16,17-Dihydro-16a,17-dihydroxygibberellin A4 17-gluc	C25H36O12	527.2137298	No
9693	HMDB0038546	ent-6R,16bOH,17-Trihydroxy-7-oxo-6,7-seco-19,6-kaur	C26H40O11	527.2496572	Yes
9694	HMDB0033976	Physapubenolide	C30H40O8	527.2637573	No
9694	HMDB0035797	Ganoderenic acid E	C30H40O8	527.2637573	No

9694	HMDB0035895	20-Hydroxy-3,7,11,15,23-pentaoxolanost-8-en-26-oic a	C30H40O8	527.2637573	No
9694	HMDB0037565	Artenolide	C30H40O8	527.2637573	No
9706	HMDB0002409	N-[(3a,5b,7b)-7-hydroxy-24-oxo-3-(sulfooxy)cholan-24	C26H43NO8S	528.2624934	Yes
9706	HMDB0002496; C15559; CHEBI:52031	N-[(3a,5b,7a)-3-hydroxy-24-oxo-7-(sulfooxy)cholan-24	C26H43NO8S	528.2624934	Yes
9706	HMDB0002497; CHEBI:72733	Glycochenodeoxycholate-3-sulfate	C26H43NO8S	528.2624934	Yes
9721	HMDB0034679	Cinncassiol D2 glucoside	C26H42O11	529.2657579	No
9724	HMDB0030392; C17615; CHEBI:227037	Kukoamine A	C28H42N4O6	529.3044579	No
9724	HMDB0040844	(S)-Nerolidol 3-O-[a-L-rhamnopyranosyl-(1->2)-b-D-glu	C27H46O10	529.3044579	No
9724	HMDB0060525; C17616	Kukoamine B	C28H42N4O6	529.3044579	No
9724	HMDB0060526; C17617	Kukoamine C	C28H42N4O6	529.3044579	No
9724	HMDB0060527; C17618	Kukoamine D	C28H42N4O6	529.3044579	No
9728	HMDB0034033	Sabadelin	C35H62O3	529.4619976	Yes
9728	HMDB0040920; C08484	Epoxymurin A	C35H62O3	529.4619976	Yes
9728	HMDB0040922	Epoxymurin B	C35H62O3	529.4619976	Yes
9728	HMDB0040923	Epomuricenin B	C35H62O3	529.4619976	Yes
9780	HMDB0029770	7,8-Dihydrovomifoliol 9-[rhamnosyl-(1->6)-glucoside]	C25H42O12	533.261389	No
9780	HMDB0037527	3-Hydroxy-beta-ionol 3-[glucosyl-(1->6)-glucoside]	C25H42O12	533.261389	No
9781	HMDB0033846	Corchoroside A	C29H42O9	533.274189	No
9784	HMDB0035885	Ganoderic acid L	C30H46O8	533.3146336	No
9785	HMDB0061692	1-Linoleoylglycerophosphocholine	C27H53NO7P	533.3486875	No
9785	HMDB0061700	2-Linoleoylglycerophosphocholine	C27H53NO7P	533.3486875	No
9797	HMDB0037974	Cyanidin 3-(6''''-malonylglucoside)	C24H23O14	534.0990992	No
9797	HMDB0041409	Cyanidin 3-(3''''-malonyl-glucoside)	C24H23O14	534.0990992	No
9807	HMDB0135509	6-[5-(2-{[2-(3,4-dimethoxyphenyl)ethyl]-C-hydroxycarb	C26H33NO11	534.1980992	No
9807	HMDB0135511	6-[4-(2-{[3-(3,4-dimethoxyphenyl)-1-hydroxypropylide	C26H33NO11	534.1980992	No
9807	HMDB0135513	6-[5-(2-{[3-(3,4-dimethoxyphenyl)-1-hydroxypropylide	C26H33NO11	534.1980992	No
9807	HMDB0135515	6-[4-(2-{[2-(3,4-dimethoxyphenyl)ethyl]-C-hydroxycarb	C26H33NO11	534.1980992	No
9841	HMDB0041137	Corchorosol A	C29H44O9	535.2897895	No
9842	HMDB0041137	Corchorosol A	C29H44O9	535.2911185	No
9859	HMDB0000790	N-Palmitoylsphingosine	C34H67NO3	536.5040214	No
9859	HMDB0004949; C00195; CHEBI:72959	Ceramide (d18:1/16:0)	C34H67NO3	536.5040214	No
9875	HMDB0037690	Limocitrol 3-glucoside	C24H26O14	537.1241082	No
9875	HMDB0038757	Isolimocitrol 3-glucoside	C24H26O14	537.1241082	No
9908	HMDB0010357	Tetrahydroaldosterone-3-glucuronide	C27H40O11	539.2502196	Yes
9913	HMDB0071246	TG(8:0/8:0/a-13:0)[rac]	C32H60O6	539.4303956	No
9913	HMDB0071529	TG(8:0/8:0/i-13:0)	C32H60O6	539.4303956	No
9913	HMDB0072647	TG(8:0/8:0/13:0)	C32H60O6	539.4303956	No
9913	HMDB0096559	TG(a-13:0/8:0/8:0)[rac]	C32H60O6	539.4303956	No
9913	HMDB0096560	TG(i-13:0/8:0/8:0)	C32H60O6	539.4303956	No
9913	HMDB0096561	TG(13:0/8:0/8:0)	C32H60O6	539.4303956	No
9936	HMDB0010320	Cortolone-3-glucuronide	C27H42O11	541.2654781	Yes
9946	HMDB0134859	6,11-dihydroxy-7-(4-hydroxy-3,5-dimethoxyphenyl)-3-|	C25H19O12S	542.0498207	No
9946	HMDB0134867	3,8-dihydroxy-7-(4-hydroxy-3,5-dimethoxyphenyl)-12-|	C25H19O12S	542.0498207	No
9946	HMDB0134868	7-[3,5-dimethoxy-4-(sulfooxy)phenyl]-3,8-dihydroxy-12	C25H19O12S	542.0498207	No
9949	HMDB0128686	2-amino-4-({1-[(carboxymethyl)-C-hydroxycarbonimido	C22H29N3O11S	542.1422207	No
9994	HMDB0002640	N-[(3a,5b,7a,12a)-3,12-dihydroxy-24-oxo-7-(sulfooxy)c	C26H43NO9S	544.257491	No
9994	HMDB0002644	N-[(3a,5b,7a,12a)-3,7-dihydroxy-24-oxo-12-(sulfooxy)c	C26H43NO9S	544.257491	No
10031	HMDB0040921	Diepomuricanin A	C35H62O4	545.4564458	No
10120	HMDB0030870	1-Hydroxy-3-methoxy-7-primeverosyloxyxanthone	C25H28O14	551.1379916	No
10120	HMDB0030872	Gentioside	C25H28O14	551.1379916	No
10120	HMDB0033770	7-Hydroxy-3-methoxy-1-primeverosyloxyxanthone	C25H28O14	551.1379916	No
10120	HMDB0040506	3'',5,6-Trihydroxy-3,4'',7,8-tetramethoxyflavone 3-gluc	C25H28O14	551.1379916	No
10124	HMDB0004309; C06085; CHEBI:27151	Triterpenoid	C30H48O7S	551.3056004	No
10124	HMDB0015642; CHEBI:37922	Arbekacin	C22H44N6O10	551.3056004	No
10125	HMDB0002513; C03033	Lithocholate 3-O-glucuronide	C30H48O9	551.322608	No
10142	HMDB0128320	3-{3-[3,5-dihydroxy-4-(sulfooxy)benzoyloxy]-4,5-dihyd	C21H14O16S	552.9924517	No
10142	HMDB0128321	3,4-dihydroxy-5-[3-hydroxy-4-(sulfooxy)-5-(3,4,5-trihy	C21H14O16S	552.9924517	No
10142	HMDB0128322	3-[3,4-dihydroxy-5-(3,4,5-trihydroxybenzoyloxy)benzo	C21H14O16S	552.9924517	No
10146	HMDB0039051	Notoginsenoside R10	C30H50O9	553.3366672	No
10299	HMDB0036856	19-Hydroxycinnzeylanol 19-glucoside	C26H42O13	561.2561987	No
10304	HMDB0007017	DG(14:0/18:3(6Z,9Z,12Z)/0:0)	C35H62O5	561.4517409	No

10304	HMDB0007018	DG(14:0/18:3(9Z,12Z,15Z)/0:0)	C35H62O5	561.4517409	No
10304	HMDB0007045	DG(14:1(9Z)/18:2(9Z,12Z)/0:0)	C35H62O5	561.4517409	No
10304	HMDB0007241	DG(18:2(9Z,12Z)/14:1(9Z)/0:0)	C35H62O5	561.4517409	No
10304	HMDB0007269	DG(18:3(6Z,9Z,12Z)/14:0/0:0)	C35H62O5	561.4517409	No
10304	HMDB0007298	DG(18:3(9Z,12Z,15Z)/14:0/0:0)	C35H62O5	561.4517409	No
10304	HMDB0035898	Robustocin	C35H62O5	561.4517409	No
10304	HMDB0055968	DG(14:0/0:0/18:3n6)	C35H62O5	561.4517409	No
10304	HMDB0055975	DG(14:0/0:0/18:3n3)	C35H62O5	561.4517409	No
10327	HMDB0029260	Apigenin 6-C-glucoside 8-C-arabinoside	C26H28O14	563.1427315	Yes
10327	HMDB0030803	Corymboside	C26H28O14	563.1427315	Yes
10327	HMDB0030843; C04858; CHEBI:15932	Apiin	C26H28O14	563.1427315	Yes
10327	HMDB0033571	6-beta-D-Glucopyranosyl-8-beta-D-ribopyranosylapige	C26H28O14	563.1427315	Yes
10327	HMDB0037569	Isovitexin 2''''-O-arabinoside	C26H28O14	563.1427315	Yes
10327	HMDB0037575	Kaempferol 3-arabinofuranoside 7-rhamnofuranoside	C26H28O14	563.1427315	Yes
10327	HMDB0039319	Kaempferol 3-rhamnoside 7-xyloside	C26H28O14	563.1427315	Yes
10327	HMDB0039321	Kaempferol 3-rhamnoside 4''-xyloside	C26H28O14	563.1427315	Yes
10327	HMDB0126884	5,7-dihydroxy-2-(3-hydroxyphenyl)-6-[3,4,5-trihydroxy	C26H28O14	563.1427315	Yes
10339	HMDB0000829	N-Stearoylsphingosine	C36H71NO3	564.5381843	No
10339	HMDB0004950; C00195	Ceramide (d18:1/18:0)	C36H71NO3	564.5381843	No
10339	HMDB0011762; CHEBI:88431	Cer(d18:0/18:1(11Z))	C36H71NO3	564.5381843	No
10339	HMDB0011763; CHEBI:74100	Cer(d18:0/18:1(9Z))	C36H71NO3	564.5381843	No
10370	HMDB0014365; C07257; CHEBI:117224	Nelfinavir	C32H45N3O4S	566.3048651	No
10389	HMDB0029252	Phloretin xylosyl-galactoside	C26H32O14	567.1713428	No
10389	HMDB0031726	cis-Mulberroside A	C26H32O14	567.1713428	No
10389	HMDB0039384	Piceatannol 3,4''-diglucoside	C26H32O14	567.1713428	No
10390	HMDB0006465; C06462	Leukotriene F4	C28H44N2O8S	567.2723911	No
10390	HMDB0062488	leukotriene-f4	C28H44N2O8S	567.2723911	No
10391	HMDB0034084	Canesceol	C29H44O11	567.2812494	No
10391	HMDB0039773	11-alpha-O-beta-D-Glucopyranosyl-16alpha-O-methylr	C29H44O11	567.2812494	No
10392	HMDB0001097; C01079; CHEBI:15435	Protoporphyrinogen IX	C34H40N4O4	567.2971011	No
10392	HMDB0029342	Ceanothine E	C34H40N4O4	567.2971011	No
10392	HMDB0034101; C09994	Adouetine Y	C34H40N4O4	567.2971011	No
10395	HMDB0002430	(3a,5b,7a)-23-Carboxy-7-hydroxy-24-norcholan-3-yl-b-	C30H48O10	567.3172343	No
10395	HMDB0002596; C03033	Deoxycholic acid 3-glucuronide	C30H48O10	567.3172343	No
10398	HMDB0071165	TG(10:0/13:0/8:0)	C34H64O6	567.4619086	No
10398	HMDB0071222	TG(8:0/8:0/15:0)	C34H64O6	567.4619086	No
10398	HMDB0071443	TG(10:0/a-13:0/8:0)[rac]	C34H64O6	567.4619086	No
10398	HMDB0071663	TG(8:0/10:0/i-13:0)	C34H64O6	567.4619086	No
10398	HMDB0071676	TG(8:0/8:0/a-15:0)[rac]	C34H64O6	567.4619086	No
10398	HMDB0071685	TG(10:0/i-13:0/8:0)	C34H64O6	567.4619086	No
10398	HMDB0071738	TG(8:0/8:0/i-15:0)	C34H64O6	567.4619086	No
10398	HMDB0071968	TG(8:0/a-13:0/10:0)[rac]	C34H64O6	567.4619086	No
10398	HMDB0072206	TG(10:0/8:0/a-13:0)[rac]	C34H64O6	567.4619086	No
10398	HMDB0072245	TG(8:0/10:0/a-13:0)[rac]	C34H64O6	567.4619086	No
10398	HMDB0072333	TG(8:0/10:0/13:0)	C34H64O6	567.4619086	No
10398	HMDB0072338	TG(10:0/8:0/i-13:0)	C34H64O6	567.4619086	No
10398	HMDB0072529	TG(8:0/13:0/10:0)	C34H64O6	567.4619086	No
10398	HMDB0072531	TG(10:0/8:0/13:0)	C34H64O6	567.4619086	No
10398	HMDB0072546	TG(8:0/i-13:0/10:0)	C34H64O6	567.4619086	No
10398	HMDB0096562	TG(a-13:0/8:0/10:0)[rac]	C34H64O6	567.4619086	No
10398	HMDB0096563	TG(i-13:0/8:0/10:0)	C34H64O6	567.4619086	No
10398	HMDB0096564	TG(13:0/8:0/10:0)	C34H64O6	567.4619086	No
10398	HMDB0096649	TG(a-13:0/10:0/8:0)[rac]	C34H64O6	567.4619086	No
10398	HMDB0096650	TG(i-13:0/10:0/8:0)	C34H64O6	567.4619086	No
10398	HMDB0096651	TG(13:0/10:0/8:0)	C34H64O6	567.4619086	No
10398	HMDB0100884	TG(a-15:0/8:0/8:0)[rac]	C34H64O6	567.4619086	No
10398	HMDB0100885	TG(i-15:0/8:0/8:0)	C34H64O6	567.4619086	No
10398	HMDB0100886	TG(15:0/8:0/8:0)	C34H64O6	567.4619086	No
10406	HMDB0041911	Isepamicin	C22H43N5O12	568.2843109	No
10429	HMDB0035696	Pyrrhoxanthinol	C37H46O5	569.3256224	No
10430	HMDB0035292	Ganodermic acid P2	C34H50O7	569.3507708	No

10430	HMDB0035294	Ganoderic acid Mk	C34H50O7	569.3507708	No
10430	HMDB0035605	3alpha,15alpha-Diacetoxy-(22R)-hydroxylanosta-7,9(1	C34H50O7	569.3507708	No
10441	HMDB0035519	1,1''-(1,4-Dihydro-4-nonyl-3,5-pyridinediyl)bis[1-dodec	C38H69NO2	570.5246506	No
10497	HMDB0007077	DG(15:0/18:4(6Z,9Z,12Z,15Z)/0:0)	C36H62O5	573.4511664	No
10497	HMDB0007329	DG(18:4(6Z,9Z,12Z,15Z)/15:0/0:0)	C36H62O5	573.4511664	No
10497	HMDB0056004	DG(15:0/0:0/18:4n3)	C36H62O5	573.4511664	No
10549	HMDB0030046	Asparagoside A	C33H54O8	577.3746729	No
10556	HMDB0125088	[(6-{[5,7-dihydroxy-2-(4-oxocyclohexa-2,5-dien-1-ylide	C30H27O12	578.1434591	No
10601	HMDB0032522	Stearyl monoglyceridyl citrate	C28H54O12	581.3537869	No
10635	HMDB0002577	Cholic acid glucuronide	C30H48O11	583.3118804	Yes
10666	HMDB0001278; C03428; CHEBI:15442	Presqualene diphosphate	C30H52O7P2	585.3121916	No
10666	HMDB0012188; C01230; CHEBI:17528	All-trans-hexaprenyl diphosphate	C30H52O7P2	585.3121916	No
10705	HMDB0033261	Mulberrofuran M	C34H22O10	589.1153358	No
10708	HMDB0037956	(24R)-5b,8b-Epidioxyergosta-6,22E-dien-3b-ol 3-glucos	C34H54O8	589.3751831	No
10728	HMDB0001898; C05790; CHEBI:29026	Mesobilirubinogen	C33H44N4O6	591.3183723	No
10729	HMDB0034391	Agavoside A	C33H52O9	591.3523595	No
10744	HMDB0039077	Lactariamide B	C37H71NO4	592.5284645	No
10745	HMDB0039077	Lactariamide B	C37H71NO4	592.5326645	No
10749	HMDB0004951; C00195	Ceramide (d18:1/20:0)	C38H75NO3	592.5679545	No
10756	HMDB0034955; C17565	Zizybeoside II	C25H38O16	593.2064642	No
10757	HMDB0004159; C05793	L-Urobilin	C33H46N4O6	593.3337831	No
10764	HMDB0127912	6-(4-{5,7-dihydroxy-3-[(3,4,5-trihydroxyoxan-2-yl)oxy]-	C26H27O16	594.1224251	No
10764	HMDB0127913	6-(5-{5,7-dihydroxy-3-[(3,4,5-trihydroxyoxan-2-yl)oxy]-	C26H27O16	594.1224251	No
10764	HMDB0127914	6-{[2-(3,4-dihydroxyphenyl)-5-hydroxy-3-[(3,4,5-trihyd	C26H27O16	594.1224251	No
10764	HMDB0127915	6-{[2-(3,4-dihydroxyphenyl)-7-hydroxy-3-[(3,4,5-trihyd	C26H27O16	594.1224251	No
10788	HMDB0005811; C09732	Eriocitrin	C27H32O15	595.1664834	No
10788	HMDB0034609	Isorubrofusarin 10-gentiobioside	C27H32O15	595.1664834	No
10788	HMDB0037407	(2R)-6,8-Diglucopyranosyl-4'',5,7-trihydroxyflavanone	C27H32O15	595.1664834	No
10788	HMDB0037506; C09826	Pinobanksin 5-[galactosyl-(1->4)-glucoside]	C27H32O15	595.1664834	No
10788	HMDB0038478	Rubrofusarin 6-gentiobioside	C27H32O15	595.1664834	No
10788	HMDB0038550	Cassiaside C	C27H32O15	595.1664834	No
10788	HMDB0141965	2-[3,4-bis(2-hydroxyethoxy)phenyl]-5-hydroxy-7-(2-hy	C27H32O15	595.1664834	No
10794	HMDB0004157; C05789; CHEBI:36479	L-Urobilinogen	C33H48N4O6	595.3492986	No
10796	HMDB0002204; C08580; CHEBI:40968	Astaxanthin	C40H52O4	595.3789633	No
10796	HMDB0039091	Capsorubinidione	C40H52O4	595.3789633	No
10796	HMDB0039128	(3''RS,3''SR)-Astaxanthin	C40H52O4	595.3789633	No
10797	HMDB0029315	Asparagoside B	C33H56O9	595.3852633	No
10826	HMDB0039579	Sucrose monopalmitate	C28H54O13	597.3475325	No
10880	HMDB0014824	Pentosan Polysulfate	C10H18O21S4	600.9160808	No
10892	HMDB0015006; CHEBI:3032	Benzonatate	C30H53NO11	602.3539393	No
10892	HMDB0033397	Fumonisin AK1	C30H53NO11	602.3539393	No
10905	HMDB0036060	Hebevinoside IV	C36H60O7	603.4282382	No
10905	HMDB0039645	Ginsenoside Rh3	C36H60O7	603.4282382	No
10906	HMDB0036295	Cycloartanyl ferulate	C40H60O4	603.4421657	No
10947	HMDB0041368	24-Acetyl- 25-cinnamoylvulgaroside	C36H48O8	607.3274775	No
10975	HMDB0132725	6-[5-(3-{[3,4-dihydroxy-5-(hydroxymethyl)oxolan-2-yl](	C26H26O17	609.1086064	No
10975	HMDB0132726	6-[(3-{[3,4-dihydroxy-5-(hydroxymethyl)oxolan-2-yl]ox	C26H26O17	609.1086064	No
10975	HMDB0132727	6-[(3-{[3,4-dihydroxy-5-(hydroxymethyl)oxolan-2-yl]ox	C26H26O17	609.1086064	No
10975	HMDB0135136	(3-{3,7-dihydroxy-4-[3,5,7-trihydroxy-2-(3-hydroxyphe	C30H26O12S	609.1086064	No
10975	HMDB0135137	(3-{8-[3,7-dihydroxy-2-(3-hydroxyphenyl)-3,4-dihydro-	C30H26O12S	609.1086064	No
10975	HMDB0135138	[7-hydroxy-2-(3-hydroxyphenyl)-4-[3,5,7-trihydroxy-2-	C30H26O12S	609.1086064	No
11013	HMDB0035606	(24E)-3beta,15alpha,22S-Triacetoxylanosta-7,9(11),24-	C36H52O8	611.3572851	No
11017	HMDB0010355; C03033	Cholestane-3,7,12,25-tetrol-3-glucuronide	C33H56O10	611.3784662	No
11094	HMDB0013022	Neuromedin N	C32H51N5O7	616.3693637	No
11115	HMDB0129016	2-amino-4-({1-[(carboxymethyl)-C-hydroxycarbonimid(	C29H37N3O10S	618.2118708	No
11115	HMDB0129017	2-amino-4-({1-[(carboxymethyl)-C-hydroxycarbonimid(	C29H37N3O10S	618.2118708	No
11115	HMDB0129021	2-amino-4-({1-[(carboxymethyl)-C-hydroxycarbonimid(	C29H37N3O10S	618.2118708	No
11115	HMDB0129022	2-amino-4-({1-[(carboxymethyl)-C-hydroxycarbonimid(	C29H37N3O10S	618.2118708	No
11188	HMDB0116642	PG(a-13:0/i-12:0)	C31H61O10P	623.3922501	No
11188	HMDB0116655	PG(i-12:0/a-13:0)	C31H61O10P	623.3922501	No
11188	HMDB0116661	PG(i-12:0/i-13:0)	C31H61O10P	623.3922501	No

11188	HMDB0116678	PG(i-13:0/i-12:0)	C31H61O10P	623.3922501	No
11214	HMDB0039580	Sucrose monostearate	C30H58O13	625.3816532	No
11222	HMDB0015641; CHEBI:661520	Dabigatran etexilate	C34H41N7O5	626.3108349	No
11260	HMDB0038377	Goshonoside F4	C32H54O12	629.3536374	No
11260	HMDB0038378	Goshonoside F7	C32H54O12	629.3536374	No
11260	HMDB0039577	Lyciumoside I	C32H54O12	629.3536374	No
11289	HMDB0000825	3''-Sialyllactose	C23H39NO19	632.2034359	Yes
11289	HMDB0006569	6''-Sialyllactose	C23H39NO19	632.2034359	Yes
11364	HMDB0037462	Tricin 7-neohesperidoside	C29H34O16	637.177456	No
11364	HMDB0037467; C17441	Rhamnazin 3-rutinoside	C29H34O16	637.177456	No
11441	HMDB0125359	({5-[(4,5-dihydroxy-2-{[5-hydroxy-2-(4-hydroxyphenyl)-	C26H28O17S	643.0958925	No
11441	HMDB0125360	{2-[(4,5-dihydroxy-2-{[5-hydroxy-2-(4-hydroxyphenyl)-4	C26H28O17S	643.0958925	No
11441	HMDB0126914	(3-{5,7-dihydroxy-4-oxo-6-[3,4,5-trihydroxy-6-(hydroxy	C26H28O17S	643.0958925	No
11441	HMDB0126915	({6-[5,7-dihydroxy-2-(3-hydroxyphenyl)-4-oxo-8-(3,4,5-	C26H28O17S	643.0958925	No
11441	HMDB0126916	{2-[5,7-dihydroxy-2-(3-hydroxyphenyl)-4-oxo-6-[3,4,5-t	C26H28O17S	643.0958925	No
11441	HMDB0126917	{2-[5,7-dihydroxy-2-(3-hydroxyphenyl)-4-oxo-6-[3,4,5-t	C26H28O17S	643.0958925	No
11441	HMDB0126918	{6-[5,7-dihydroxy-2-(3-hydroxyphenyl)-4-oxo-6-[3,4,5-t	C26H28O17S	643.0958925	No
11441	HMDB0126919	{6-[5,7-dihydroxy-2-(3-hydroxyphenyl)-4-oxo-8-(3,4,5-t	C26H28O17S	643.0958925	No
11441	HMDB0126920	{2-[5,7-dihydroxy-2-(3-hydroxyphenyl)-4-oxo-8-(3,4,5-t	C26H28O17S	643.0958925	No
11441	HMDB0126921	{2-[5,7-dihydroxy-2-(3-hydroxyphenyl)-4-oxo-8-(3,4,5-t	C26H28O17S	643.0958925	No
11441	HMDB0140261	{2-[5,7-dihydroxy-2-(4-hydroxyphenyl)-4-oxo-6-[3,4,5-t	C26H28O17S	643.0958925	No
11441	HMDB0140262	{2-[5,7-dihydroxy-2-(4-hydroxyphenyl)-4-oxo-6-[3,4,5-t	C26H28O17S	643.0958925	No
11441	HMDB0140263	{6-[5,7-dihydroxy-2-(4-hydroxyphenyl)-4-oxo-6-[3,4,5-t	C26H28O17S	643.0958925	No
11441	HMDB0140264	{6-[5,7-dihydroxy-2-(4-hydroxyphenyl)-4-oxo-8-(3,4,5-t	C26H28O17S	643.0958925	No
11441	HMDB0140265	{2-[5,7-dihydroxy-2-(4-hydroxyphenyl)-4-oxo-8-(3,4,5-t	C26H28O17S	643.0958925	No
11441	HMDB0140266	{2-[5,7-dihydroxy-2-(4-hydroxyphenyl)-4-oxo-8-(3,4,5-t	C26H28O17S	643.0958925	No
11451	HMDB0030530	Janthitrem F	C39H51NO7	644.3604294	No
11513	HMDB0011768; CHEBI:52961	Cer(d18:0/24:0)	C42H85NO3	650.6470332	No
11541	HMDB0012187; C04216; CHEBI:17613	All-trans-heptaprenyl diphosphate	C35H60O7P2	653.3756135	No
11617	HMDB0002318	Capsianoside I	C32H52O14	659.3289239	No
11628	HMDB0008825; C00350	PE(14:0/16:1(9Z))	C35H68NO8P	660.4604023	No
11628	HMDB0008857; C00350	PE(14:1(9Z)/16:0)	C35H68NO8P	660.4604023	No
11628	HMDB0008921; C00350	PE(16:0/14:1(9Z))	C35H68NO8P	660.4604023	No
11628	HMDB0008953; C00350	PE(16:1(9Z)/14:0)	C35H68NO8P	660.4604023	No
11628	HMDB0112938	PE-NMe(14:1(9Z)/15:0)	C35H68NO8P	660.4604023	No
11628	HMDB0113018	PE-NMe(15:0/14:1(9Z))	C35H68NO8P	660.4604023	No
11628	HMDB0113839	PE-NMe2(14:0/14:1(9Z))	C35H68NO8P	660.4604023	No
11628	HMDB0113899	PE-NMe2(14:1(9Z)/14:0)	C35H68NO8P	660.4604023	No
11640	HMDB0008824; C00350	PE(14:0/16:0)	C35H70NO8P	662.4755849	No
11640	HMDB0008889; C00350	PE(15:0/15:0)	C35H70NO8P	662.4755849	No
11640	HMDB0008920; C00350	PE(16:0/14:0)	C35H70NO8P	662.4755849	No
11640	HMDB0112936	PE-NMe(14:0/15:0)	C35H70NO8P	662.4755849	No
11640	HMDB0113017	PE-NMe(15:0/14:0)	C35H70NO8P	662.4755849	No
11640	HMDB0113837	PE-NMe2(14:0/14:0)	C35H70NO8P	662.4755849	No
11652	HMDB0114803	PA(14:1(9Z)/20:5(5Z,8Z,11Z,14Z,17Z))	C37H61O8P	663.4046323	No
11652	HMDB0115199	PA(20:5(5Z,8Z,11Z,14Z,17Z)/14:1(9Z))	C37H61O8P	663.4046323	No
11674	HMDB0000757; C00182; CHEBI:28087	Glycogen	C24H42O21	665.2139976	Yes
11674	HMDB0001296; C02052; CHEBI:61988	Maltotetraose	C24H42O21	665.2139976	Yes
11674	HMDB0003553; C01613; CHEBI:17164	Stachyose	C24H42O21	665.2139976	Yes
11674	HMDB0029931; C00464; CHEBI:28808	Mannan	C24H42O21	665.2139976	Yes
11674	HMDB0035323	Fagopyritol B3	C24H42O21	665.2139976	Yes
11674	HMDB0038490	Bifurcose	C24H42O21	665.2139976	Yes
11674	HMDB0038870	Neobifurcose	C24H42O21	665.2139976	Yes
11674	HMDB0038871; C08248	Sesamose	C24H42O21	665.2139976	Yes
11674	HMDB0039173	3-beta-Glucosylcellotriose	C24H42O21	665.2139976	Yes
11674	HMDB0039174	3-beta-Cellobiosylcellobiose	C24H42O21	665.2139976	Yes
11674	HMDB0039176	Nystose	C24H42O21	665.2139976	Yes
11674	HMDB0040182	Fagopyritol A3	C24H42O21	665.2139976	Yes
11675	HMDB0126250	2-{6-[2,4-dihydroxy-3-(3-methylbut-2-en-1-yl)benzoyl]-	C39H38O10	665.2391571	No
11675	HMDB0126251	2-{6-[2,4-dihydroxy-3-(3-methylbut-2-en-1-yl)benzoyl]-	C39H38O10	665.2391571	No
11675	HMDB0126252	2-{6-[2,4-dihydroxy-3-(4-hydroxy-3-methylbut-2-en-1-y	C39H38O10	665.2391571	No

11675	HMDB0126253	2-(6-{2,4-dihydroxy-3-[(2E)-4-hydroxy-3-methylbut-2-e	C39H38O10	665.2391571	No
11675	HMDB0126254	2-{6-[2,4-dihydroxy-3-(3-methylbut-2-en-1-yl)benzoyl]·	C39H38O10	665.2391571	No
11675	HMDB0126255	2-{6-[2,4-dihydroxy-3-(3-methylbut-2-en-1-yl)benzoyl]·	C39H38O10	665.2391571	No
11675	HMDB0126256	2-[5-(2,4-dihydroxyphenyl)-6-{3-[(3,3-dimethyloxiran-2	C39H38O10	665.2391571	No
11675	HMDB0126257	2-{3-[2,4-dihydroxy-3-(3-methylbut-2-en-1-yl)benzoyl]·	C39H38O10	665.2391571	No
11675	HMDB0126258	5-{6-[2,4-dihydroxy-3-(3-methylbut-2-en-1-yl)benzoyl]·	C39H38O10	665.2391571	No
11675	HMDB0126259	3-{6-[2,4-dihydroxy-3-(3-methylbut-2-en-1-yl)benzoyl]·	C39H38O10	665.2391571	No
11675	HMDB0126260	5-[(E)-2-(4-{6-[2,4-dihydroxy-3-(3-methylbut-2-en-1-yl)	C39H38O10	665.2391571	No
11675	HMDB0126261	5-[6-(2,4-dihydroxyphenyl)-2-{4-[(E)-2-(2,4-dihydroxyp	C39H38O10	665.2391571	No
11681	HMDB0114787	PA(14:0/20:5(5Z,8Z,11Z,14Z,17Z))	C37H63O8P	665.4170269	No
11681	HMDB0114801	PA(14:1(9Z)/20:4(5Z,8Z,11Z,14Z))	C37H63O8P	665.4170269	No
11681	HMDB0114802	PA(14:1(9Z)/20:4(8Z,11Z,14Z,17Z))	C37H63O8P	665.4170269	No
11681	HMDB0114859	PA(16:1(9Z)/18:4(6Z,9Z,12Z,15Z))	C37H63O8P	665.4170269	No
11681	HMDB0115031	PA(18:4(6Z,9Z,12Z,15Z)/16:1(9Z))	C37H63O8P	665.4170269	No
11681	HMDB0115145	PA(20:4(5Z,8Z,11Z,14Z)/14:1(9Z))	C37H63O8P	665.4170269	No
11681	HMDB0115172	PA(20:4(8Z,11Z,14Z,17Z)/14:1(9Z))	C37H63O8P	665.4170269	No
11681	HMDB0115198	PA(20:5(5Z,8Z,11Z,14Z,17Z)/14:0)	C37H63O8P	665.4170269	No
11682	HMDB0039050	Notoginsenoside T2	C37H62O10	665.426055	No
11683	HMDB0007093	DG(15:0/24:0/0:0)	C42H82O5	665.6091873	No
11683	HMDB0007793	DG(24:0/15:0/0:0)	C42H82O5	665.6091873	No
11683	HMDB0055986	DG(15:0/0:0/24:0)	C42H82O5	665.6091873	No
11683	HMDB0093423	DG(i-14:0/a-25:0/0:0)	C42H82O5	665.6091873	No
11683	HMDB0093424	DG(i-14:0/0:0/a-25:0)	C42H82O5	665.6091873	No
11683	HMDB0093425	DG(14:0/a-25:0/0:0)	C42H82O5	665.6091873	No
11683	HMDB0093426	DG(14:0/0:0/a-25:0)	C42H82O5	665.6091873	No
11683	HMDB0093584	DG(a-15:0/i-24:0/0:0)	C42H82O5	665.6091873	No
11683	HMDB0093585	DG(a-15:0/0:0/i-24:0)	C42H82O5	665.6091873	No
11683	HMDB0093586	DG(i-15:0/i-24:0/0:0)	C42H82O5	665.6091873	No
11683	HMDB0093587	DG(i-15:0/0:0/i-24:0)	C42H82O5	665.6091873	No
11683	HMDB0093588	DG(15:0/i-24:0/0:0)	C42H82O5	665.6091873	No
11683	HMDB0093589	DG(15:0/0:0/i-24:0)	C42H82O5	665.6091873	No
11683	HMDB0093862	DG(17:0/22:0/0:0)	C42H82O5	665.6091873	No
11683	HMDB0093863	DG(17:0/0:0/22:0)	C42H82O5	665.6091873	No
11683	HMDB0093864	DG(i-17:0/22:0/0:0)	C42H82O5	665.6091873	No
11683	HMDB0093865	DG(i-17:0/0:0/22:0)	C42H82O5	665.6091873	No
11683	HMDB0093866	DG(a-17:0/i-22:0/0:0)	C42H82O5	665.6091873	No
11683	HMDB0093867	DG(a-17:0/0:0/i-22:0)	C42H82O5	665.6091873	No
11683	HMDB0093868	DG(a-17:0/22:0/0:0)	C42H82O5	665.6091873	No
11683	HMDB0093869	DG(a-17:0/0:0/22:0)	C42H82O5	665.6091873	No
11683	HMDB0093870	DG(i-17:0/i-22:0/0:0)	C42H82O5	665.6091873	No
11683	HMDB0093871	DG(i-17:0/0:0/i-22:0)	C42H82O5	665.6091873	No
11683	HMDB0093872	DG(17:0/i-22:0/0:0)	C42H82O5	665.6091873	No
11683	HMDB0093873	DG(17:0/0:0/i-22:0)	C42H82O5	665.6091873	No
11683	HMDB0093971	DG(18:0/21:0/0:0)	C42H82O5	665.6091873	No
11683	HMDB0093972	DG(18:0/0:0/21:0)	C42H82O5	665.6091873	No
11683	HMDB0093973	DG(i-18:0/21:0/0:0)	C42H82O5	665.6091873	No
11683	HMDB0093974	DG(i-18:0/0:0/21:0)	C42H82O5	665.6091873	No
11683	HMDB0093975	DG(i-18:0/a-21:0/0:0)	C42H82O5	665.6091873	No
11683	HMDB0093976	DG(i-18:0/0:0/a-21:0)	C42H82O5	665.6091873	No
11683	HMDB0093977	DG(i-18:0/i-21:0/0:0)	C42H82O5	665.6091873	No
11683	HMDB0093978	DG(i-18:0/0:0/i-21:0)	C42H82O5	665.6091873	No
11683	HMDB0093979	DG(18:0/i-21:0/0:0)	C42H82O5	665.6091873	No
11683	HMDB0093980	DG(18:0/0:0/i-21:0)	C42H82O5	665.6091873	No
11683	HMDB0093981	DG(18:0/a-21:0/0:0)	C42H82O5	665.6091873	No
11683	HMDB0093982	DG(18:0/0:0/a-21:0)	C42H82O5	665.6091873	No
11683	HMDB0094081	DG(19:0/20:0/0:0)	C42H82O5	665.6091873	No
11683	HMDB0094082	DG(19:0/0:0/20:0)	C42H82O5	665.6091873	No
11683	HMDB0094083	DG(i-19:0/20:0/0:0)	C42H82O5	665.6091873	No
11683	HMDB0094084	DG(i-19:0/0:0/20:0)	C42H82O5	665.6091873	No
11683	HMDB0094085	DG(i-19:0/i-20:0/0:0)	C42H82O5	665.6091873	No
11683	HMDB0094086	DG(i-19:0/0:0/i-20:0)	C42H82O5	665.6091873	No

11683	HMDB0094087	DG(19:0/i-20:0/0:0)	C42H82O5	665.6091873	No
11683	HMDB0094088	DG(19:0/0:0/i-20:0)	C42H82O5	665.6091873	No
11683	HMDB0094189	DG(20:0/19:0/0:0)	C42H82O5	665.6091873	No
11683	HMDB0094190	DG(20:0/0:0/19:0)	C42H82O5	665.6091873	No
11683	HMDB0094191	DG(i-20:0/19:0/0:0)	C42H82O5	665.6091873	No
11683	HMDB0094192	DG(i-20:0/0:0/19:0)	C42H82O5	665.6091873	No
11683	HMDB0094193	DG(i-20:0/i-19:0/0:0)	C42H82O5	665.6091873	No
11683	HMDB0094194	DG(i-20:0/0:0/i-19:0)	C42H82O5	665.6091873	No
11683	HMDB0094195	DG(20:0/i-19:0/0:0)	C42H82O5	665.6091873	No
11683	HMDB0094196	DG(20:0/0:0/i-19:0)	C42H82O5	665.6091873	No
11683	HMDB0094331	DG(21:0/18:0/0:0)	C42H82O5	665.6091873	No
11683	HMDB0094332	DG(21:0/0:0/18:0)	C42H82O5	665.6091873	No
11683	HMDB0094333	DG(i-21:0/18:0/0:0)	C42H82O5	665.6091873	No
11683	HMDB0094334	DG(i-21:0/0:0/18:0)	C42H82O5	665.6091873	No
11683	HMDB0094335	DG(a-21:0/i-18:0/0:0)	C42H82O5	665.6091873	No
11683	HMDB0094336	DG(a-21:0/0:0/i-18:0)	C42H82O5	665.6091873	No
11683	HMDB0094337	DG(a-21:0/18:0/0:0)	C42H82O5	665.6091873	No
11683	HMDB0094338	DG(a-21:0/0:0/18:0)	C42H82O5	665.6091873	No
11683	HMDB0094339	DG(i-21:0/i-18:0/0:0)	C42H82O5	665.6091873	No
11683	HMDB0094340	DG(i-21:0/0:0/i-18:0)	C42H82O5	665.6091873	No
11683	HMDB0094341	DG(21:0/i-18:0/0:0)	C42H82O5	665.6091873	No
11683	HMDB0094342	DG(21:0/0:0/i-18:0)	C42H82O5	665.6091873	No
11683	HMDB0094462	DG(22:0/17:0/0:0)	C42H82O5	665.6091873	No
11683	HMDB0094463	DG(22:0/0:0/17:0)	C42H82O5	665.6091873	No
11683	HMDB0094464	DG(i-22:0/17:0/0:0)	C42H82O5	665.6091873	No
11683	HMDB0094465	DG(i-22:0/0:0/17:0)	C42H82O5	665.6091873	No
11683	HMDB0094466	DG(i-22:0/a-17:0/0:0)	C42H82O5	665.6091873	No
11683	HMDB0094467	DG(i-22:0/0:0/a-17:0)	C42H82O5	665.6091873	No
11683	HMDB0094468	DG(i-22:0/i-17:0/0:0)	C42H82O5	665.6091873	No
11683	HMDB0094469	DG(i-22:0/0:0/i-17:0)	C42H82O5	665.6091873	No
11683	HMDB0094470	DG(22:0/i-17:0/0:0)	C42H82O5	665.6091873	No
11683	HMDB0094471	DG(22:0/0:0/i-17:0)	C42H82O5	665.6091873	No
11683	HMDB0094472	DG(22:0/a-17:0/0:0)	C42H82O5	665.6091873	No
11683	HMDB0094473	DG(22:0/0:0/a-17:0)	C42H82O5	665.6091873	No
11683	HMDB0094540	DG(i-24:0/15:0/0:0)	C42H82O5	665.6091873	No
11683	HMDB0094541	DG(i-24:0/0:0/15:0)	C42H82O5	665.6091873	No
11683	HMDB0094542	DG(i-24:0/a-15:0/0:0)	C42H82O5	665.6091873	No
11683	HMDB0094543	DG(i-24:0/0:0/a-15:0)	C42H82O5	665.6091873	No
11683	HMDB0094544	DG(i-24:0/i-15:0/0:0)	C42H82O5	665.6091873	No
11683	HMDB0094545	DG(i-24:0/0:0/i-15:0)	C42H82O5	665.6091873	No
11683	HMDB0094596	DG(a-25:0/i-14:0/0:0)	C42H82O5	665.6091873	No
11683	HMDB0094597	DG(a-25:0/0:0/i-14:0)	C42H82O5	665.6091873	No
11683	HMDB0094598	DG(a-25:0/14:0/0:0)	C42H82O5	665.6091873	No
11683	HMDB0094599	DG(a-25:0/0:0/14:0)	C42H82O5	665.6091873	No
11694	HMDB0059796	Gluten exorphin C	C29H57N5O12	666.3932157	No
11719	HMDB0032199; C05821; CHEBI:28855	Chymosin preparation, escherichia coli k-12	C30H27N3O15	668.1366657	No
11735	HMDB0039436	Ginsenoside Rh6	C36H62O11	669.4212869	No
11752	HMDB0008885; C00350	PE(14:1(9Z)/P-18:1(11Z))	C37H70NO7P	670.4790079	No
11752	HMDB0008886; C00350	PE(14:1(9Z)/P-18:1(9Z))	C37H70NO7P	670.4790079	No
11752	HMDB0011402	PE(P-18:1(11Z)/14:1(9Z))	C37H70NO7P	670.4790079	No
11752	HMDB0011435	PE(P-18:1(9Z)/14:1(9Z))	C37H70NO7P	670.4790079	No
11775	HMDB0008852; C00350	PE(14:0/P-18:1(11Z))	C37H72NO7P	672.4953768	No
11775	HMDB0008853; C00350	PE(14:0/P-18:1(9Z))	C37H72NO7P	672.4953768	No
11775	HMDB0008884; C00350	PE(14:1(9Z)/P-18:0)	C37H72NO7P	672.4953768	No
11775	HMDB0008982; C00350	PE(16:1(9Z)/P-16:0)	C37H72NO7P	672.4953768	No
11775	HMDB0011339	PE(P-16:0/16:1(9Z))	C37H72NO7P	672.4953768	No
11775	HMDB0011369	PE(P-18:0/14:1(9Z))	C37H72NO7P	672.4953768	No
11775	HMDB0011401	PE(P-18:1(11Z)/14:0)	C37H72NO7P	672.4953768	No
11775	HMDB0011434	PE(P-18:1(9Z)/14:0)	C37H72NO7P	672.4953768	No
11780	HMDB0001081; C04886; CHEBI:17725	(N-acetylneuraminosyl(a2-6)lactosamine)	C25H42N2O19	673.2306473	Yes
11780	HMDB0006581	3-Sialyl-N-acetyllactosamine	C25H42N2O19	673.2306473	Yes

11780	HMDB0006584	6-Sialyl-N-acetyllactosamine	C25H42N2O19	673.2306473	Yes
11809	HMDB0114783	PA(14:0/20:0)	C37H73O8P	675.4966566	No
11809	HMDB0114876	PA(18:0/16:0)	C37H73O8P	675.4966566	No
11809	HMDB0115061	PA(20:0/14:0)	C37H73O8P	675.4966566	No
11809	HMDB0115226	PA(21:0/13:0)	C37H73O8P	675.4966566	No
11809	HMDB0115238	PA(22:0/12:0)	C37H73O8P	675.4966566	No
11809	HMDB0115622	PA(10:0/i-24:0)	C37H73O8P	675.4966566	No
11809	HMDB0115636	PA(20:0/i-14:0)	C37H73O8P	675.4966566	No
11809	HMDB0115646	PA(21:0/a-13:0)	C37H73O8P	675.4966566	No
11809	HMDB0115652	PA(21:0/i-13:0)	C37H73O8P	675.4966566	No
11809	HMDB0115668	PA(22:0/i-12:0)	C37H73O8P	675.4966566	No
11809	HMDB0115701	PA(a-13:0/a-21:0)	C37H73O8P	675.4966566	No
11809	HMDB0115712	PA(a-13:0/i-21:0)	C37H73O8P	675.4966566	No
11809	HMDB0115717	PA(a-21:0/13:0)	C37H73O8P	675.4966566	No
11809	HMDB0115728	PA(a-21:0/a-13:0)	C37H73O8P	675.4966566	No
11809	HMDB0115734	PA(a-21:0/i-13:0)	C37H73O8P	675.4966566	No
11809	HMDB0115791	PA(i-12:0/i-22:0)	C37H73O8P	675.4966566	No
11809	HMDB0115797	PA(i-13:0/a-21:0)	C37H73O8P	675.4966566	No
11809	HMDB0115808	PA(i-13:0/i-21:0)	C37H73O8P	675.4966566	No
11809	HMDB0115833	PA(i-20:0/14:0)	C37H73O8P	675.4966566	No
11809	HMDB0115850	PA(i-20:0/i-14:0)	C37H73O8P	675.4966566	No
11809	HMDB0115862	PA(i-21:0/13:0)	C37H73O8P	675.4966566	No
11809	HMDB0115873	PA(i-21:0/a-13:0)	C37H73O8P	675.4966566	No
11809	HMDB0115879	PA(i-21:0/i-13:0)	C37H73O8P	675.4966566	No
11809	HMDB0115891	PA(i-22:0/12:0)	C37H73O8P	675.4966566	No
11809	HMDB0115908	PA(i-22:0/i-12:0)	C37H73O8P	675.4966566	No
11809	HMDB0115920	PA(i-24:0/10:0)	C37H73O8P	675.4966566	No
11831	HMDB0060814	Descladinose roxithromycin	C33H62N2O12	677.420571	No
11842	HMDB0015616; CHEBI:494996	Telaprevir	C36H53N7O6	678.4002131	No
11845	HMDB0012330	PS(14:0/14:0)	C34H66NO10P	678.4339231	No
11954	HMDB0008829; C00350	PE(14:0/18:2(9Z,12Z))	C37H70NO8P	686.4748595	No
11954	HMDB0008860; C00350	PE(14:1(9Z)/18:1(11Z))	C37H70NO8P	686.4748595	No
11954	HMDB0008861; C00350	PE(14:1(9Z)/18:1(9Z))	C37H70NO8P	686.4748595	No
11954	HMDB0008957; C00350	PE(16:1(9Z)/16:1(9Z))	C37H70NO8P	686.4748595	No
11954	HMDB0009020; C00350	PE(18:1(11Z)/14:1(9Z))	C37H70NO8P	686.4748595	No
11954	HMDB0009053; C00350	PE(18:1(9Z)/14:1(9Z))	C37H70NO8P	686.4748595	No
11954	HMDB0009085; C00350	PE(18:2(9Z,12Z)/14:0)	C37H70NO8P	686.4748595	No
11954	HMDB0113844	PE-NMe2(14:1(9Z)/16:1(9Z))	C37H70NO8P	686.4748595	No
11954	HMDB0113963	PE-NMe2(16:1(9Z)/14:1(9Z))	C37H70NO8P	686.4748595	No
11982	HMDB0007901; C00157	PC(14:1(9Z)/15:0)	C37H72NO8P	688.4914723	No
11982	HMDB0007933; C00157	PC(15:0/14:1(9Z))	C37H72NO8P	688.4914723	No
11982	HMDB0008827; C00350	PE(14:0/18:1(11Z))	C37H72NO8P	688.4914723	No
11982	HMDB0008828; C00350	PE(14:0/18:1(9Z))	C37H72NO8P	688.4914723	No
11982	HMDB0008859; C00350	PE(14:1(9Z)/18:0)	C37H72NO8P	688.4914723	No
11982	HMDB0008924; C00350	PE(16:0/16:1(9Z))	C37H72NO8P	688.4914723	No
11982	HMDB0008956; C00350	PE(16:1(9Z)/16:0)	C37H72NO8P	688.4914723	No
11982	HMDB0008987; C00350	PE(18:0/14:1(9Z))	C37H72NO8P	688.4914723	No
11982	HMDB0009019; C00350	PE(18:1(11Z)/14:0)	C37H72NO8P	688.4914723	No
11982	HMDB0009052; C00350	PE(18:1(9Z)/14:0)	C37H72NO8P	688.4914723	No
11982	HMDB0112945	PE-NMe(15:0/16:1(9Z))	C37H72NO8P	688.4914723	No
11982	HMDB0113063	PE-NMe(16:1(9Z)/15:0)	C37H72NO8P	688.4914723	No
11982	HMDB0113842	PE-NMe2(14:0/16:1(9Z))	C37H72NO8P	688.4914723	No
11982	HMDB0113843	PE-NMe2(14:1(9Z)/16:0)	C37H72NO8P	688.4914723	No
11982	HMDB0113941	PE-NMe2(16:0/14:1(9Z))	C37H72NO8P	688.4914723	No
11982	HMDB0113962	PE-NMe2(16:1(9Z)/14:0)	C37H72NO8P	688.4914723	No
12019	HMDB0129099	4-[(2-{[4-({8-[2-(acetyloxy)propan-2-yl]-2-oxo-2H,8H,9H	C31H39N3O13S	692.2148113	No
12026	HMDB0030492	Spirolide B	C42H63NO7	692.4514382	No
12026	HMDB0112273	PS(14:0/15:0)	C35H68NO10P	692.4514382	No
12026	HMDB0112320	PS(15:0/14:0)	C35H68NO10P	692.4514382	No
12039	HMDB0114791	PA(14:0/22:5(4Z,7Z,10Z,13Z,16Z))	C39H67O8P	693.4510422	No
12039	HMDB0114806	PA(14:1(9Z)/22:4(7Z,10Z,13Z,16Z))	C39H67O8P	693.4510422	No

12039	HMDB0114842	PA(16:0/20:5(5Z,8Z,11Z,14Z,17Z))	C39H67O8P	693.4510422	No
12039	HMDB0114861	PA(16:1(9Z)/20:4(5Z,8Z,11Z,14Z))	C39H67O8P	693.4510422	No
12039	HMDB0114862	PA(16:1(9Z)/20:4(8Z,11Z,14Z,17Z))	C39H67O8P	693.4510422	No
12039	HMDB0114905	PA(18:1(11Z)/18:4(6Z,9Z,12Z,15Z))	C39H67O8P	693.4510422	No
12039	HMDB0114930	PA(18:1(9Z)/18:4(6Z,9Z,12Z,15Z))	C39H67O8P	693.4510422	No
12039	HMDB0114955	PA(18:2(9Z,12Z)/18:3(6Z,9Z,12Z))	C39H67O8P	693.4510422	No
12039	HMDB0114956	PA(18:2(9Z,12Z)/18:3(9Z,12Z,15Z))	C39H67O8P	693.4510422	No
12039	HMDB0114981	PA(18:3(6Z,9Z,12Z)/18:2(9Z,12Z))	C39H67O8P	693.4510422	No
12039	HMDB0115008	PA(18:3(9Z,12Z,15Z)/18:2(9Z,12Z))	C39H67O8P	693.4510422	No
12039	HMDB0115033	PA(18:4(6Z,9Z,12Z,15Z)/18:1(11Z))	C39H67O8P	693.4510422	No
12039	HMDB0115034	PA(18:4(6Z,9Z,12Z,15Z)/18:1(9Z))	C39H67O8P	693.4510422	No
12039	HMDB0115148	PA(20:4(5Z,8Z,11Z,14Z)/16:1(9Z))	C39H67O8P	693.4510422	No
12039	HMDB0115175	PA(20:4(8Z,11Z,14Z,17Z)/16:1(9Z))	C39H67O8P	693.4510422	No
12039	HMDB0115201	PA(20:5(5Z,8Z,11Z,14Z,17Z)/16:0)	C39H67O8P	693.4510422	No
12039	HMDB0115324	PA(22:4(7Z,10Z,13Z,16Z)/14:1(9Z))	C39H67O8P	693.4510422	No
12039	HMDB0115349	PA(22:5(4Z,7Z,10Z,13Z,16Z)/14:0)	C39H67O8P	693.4510422	No
12039	HMDB0116700	PA(14:0/22:5(7Z,10Z,13Z,16Z,19Z))	C39H67O8P	693.4510422	No
12039	HMDB0116708	PA(22:5(7Z,10Z,13Z,16Z,19Z)/14:0)	C39H67O8P	693.4510422	No
12049	HMDB0009213; C00350	PE(18:4(6Z,9Z,12Z,15Z)/P-16:0)	C39H70NO7P	694.4811321	No
12049	HMDB0011346	PE(P-16:0/18:4(6Z,9Z,12Z,15Z))	C39H70NO7P	694.4811321	No
12086	HMDB0114805	PA(14:1(9Z)/22:2(13Z,16Z))	C39H71O8P	697.4796911	No
12086	HMDB0114840	PA(16:0/20:3(5Z,8Z,11Z))	C39H71O8P	697.4796911	No
12086	HMDB0114878	PA(18:0/18:3(6Z,9Z,12Z))	C39H71O8P	697.4796911	No
12086	HMDB0114879	PA(18:0/18:3(9Z,12Z,15Z))	C39H71O8P	697.4796911	No
12086	HMDB0114902	PA(18:1(11Z)/18:2(9Z,12Z))	C39H71O8P	697.4796911	No
12086	HMDB0114927	PA(18:1(9Z)/18:2(9Z,12Z))	C39H71O8P	697.4796911	No
12086	HMDB0114952	PA(18:2(9Z,12Z)/18:1(11Z))	C39H71O8P	697.4796911	No
12086	HMDB0114953	PA(18:2(9Z,12Z)/18:1(9Z))	C39H71O8P	697.4796911	No
12086	HMDB0114978	PA(18:3(6Z,9Z,12Z)/18:0)	C39H71O8P	697.4796911	No
12086	HMDB0115005	PA(18:3(9Z,12Z,15Z)/18:0)	C39H71O8P	697.4796911	No
12086	HMDB0115121	PA(20:3(5Z,8Z,11Z)/16:0)	C39H71O8P	697.4796911	No
12086	HMDB0115297	PA(22:2(13Z,16Z)/14:1(9Z))	C39H71O8P	697.4796911	No
12086	HMDB0115511	PA(16:0/20:3(8Z,11Z,14Z))	C39H71O8P	697.4796911	No
12086	HMDB0115512	PA(16:1(9Z)/20:2(11Z,14Z))	C39H71O8P	697.4796911	No
12086	HMDB0115536	PA(20:2(11Z,14Z)/16:1(9Z))	C39H71O8P	697.4796911	No
12086	HMDB0115564	PA(20:3(8Z,11Z,14Z)/16:0)	C39H71O8P	697.4796911	No
12124	HMDB0007903; C00157	PC(14:1(9Z)/16:1(9Z))	C38H72NO8P	700.4902987	No
12124	HMDB0007999; C00157	PC(16:1(9Z)/14:1(9Z))	C38H72NO8P	700.4902987	No
12124	HMDB0008895; C00350	PE(15:0/18:2(9Z,12Z))	C38H72NO8P	700.4902987	No
12124	HMDB0009087; C00350	PE(18:2(9Z,12Z)/15:0)	C38H72NO8P	700.4902987	No
12124	HMDB0112946	PE-NMe(14:1(9Z)/18:1(9Z))	C38H72NO8P	700.4902987	No
12124	HMDB0112947	PE-NMe(14:1(9Z)/18:1(11Z))	C38H72NO8P	700.4902987	No
12124	HMDB0112948	PE-NMe(16:1(9Z)/16:1(9Z))	C38H72NO8P	700.4902987	No
12124	HMDB0112979	PE-NMe(14:0/18:2(9Z,12Z))	C38H72NO8P	700.4902987	No
12124	HMDB0113111	PE-NMe(18:1(11Z)/14:1(9Z))	C38H72NO8P	700.4902987	No
12124	HMDB0113138	PE-NMe(18:1(9Z)/14:1(9Z))	C38H72NO8P	700.4902987	No
12124	HMDB0113164	PE-NMe(18:2(9Z,12Z)/14:0)	C38H72NO8P	700.4902987	No
12152	HMDB0007870; C00157	PC(14:0/16:1(9Z))	C38H74NO8P	702.5066838	No
12152	HMDB0007902; C00157	PC(14:1(9Z)/16:0)	C38H74NO8P	702.5066838	No
12152	HMDB0007966; C00157	PC(16:0/14:1(9Z))	C38H74NO8P	702.5066838	No
12152	HMDB0007998; C00157	PC(16:1(9Z)/14:0)	C38H74NO8P	702.5066838	No
12152	HMDB0008893; C00350	PE(15:0/18:1(11Z))	C38H74NO8P	702.5066838	No
12152	HMDB0008894; C00350	PE(15:0/18:1(9Z))	C38H74NO8P	702.5066838	No
12152	HMDB0009021; C00350	PE(18:1(11Z)/15:0)	C38H74NO8P	702.5066838	No
12152	HMDB0009054; C00350	PE(18:1(9Z)/15:0)	C38H74NO8P	702.5066838	No
12152	HMDB0112949	PE-NMe(16:0/16:1(9Z))	C38H74NO8P	702.5066838	No
12152	HMDB0112950	PE-NMe(14:0/18:1(9Z))	C38H74NO8P	702.5066838	No
12152	HMDB0112951	PE-NMe(14:0/18:1(11Z))	C38H74NO8P	702.5066838	No
12152	HMDB0112952	PE-NMe(14:1(9Z)/18:0)	C38H74NO8P	702.5066838	No
12152	HMDB0113064	PE-NMe(16:1(9Z)/16:0)	C38H74NO8P	702.5066838	No
12152	HMDB0113085	PE-NMe(18:0/14:1(9Z))	C38H74NO8P	702.5066838	No

12152	HMDB0113110	PE-NMe(18:1(11Z)/14:0)	C38H74NO8P	702.5066838	No
12152	HMDB0113137	PE-NMe(18:1(9Z)/14:0)	C38H74NO8P	702.5066838	No
12152	HMDB0113846	PE-NMe2(15:0/16:1(9Z))	C38H74NO8P	702.5066838	No
12152	HMDB0113964	PE-NMe2(16:1(9Z)/15:0)	C38H74NO8P	702.5066838	No
12190	HMDB0114828	PA(15:0/22:5(4Z,7Z,10Z,13Z,16Z))	C40H69O8P	707.4664437	No
12190	HMDB0114829	PA(15:0/22:5(7Z,10Z,13Z,16Z,19Z))	C40H69O8P	707.4664437	No
12190	HMDB0115351	PA(22:5(4Z,7Z,10Z,13Z,16Z)/15:0)	C40H69O8P	707.4664437	No
12190	HMDB0115377	PA(22:5(7Z,10Z,13Z,16Z,19Z)/15:0)	C40H69O8P	707.4664437	No
12260	HMDB0032677	Soyacerebroside I	C40H75NO9	712.5375216	No
12286	HMDB0007897; C00157	PC(14:0/P-18:1(11Z))	C40H78NO7P	714.5455341	No
12286	HMDB0007898; C00157	PC(14:0/P-18:1(9Z))	C40H78NO7P	714.5455341	No
12286	HMDB0007929; C00157	PC(14:1(9Z)/P-18:0)	C40H78NO7P	714.5455341	No
12286	HMDB0008027; C00157	PC(16:1(9Z)/P-16:0)	C40H78NO7P	714.5455341	No
12286	HMDB0011207	PC(P-16:0/16:1(9Z))	C40H78NO7P	714.5455341	No
12286	HMDB0011237	PC(P-18:0/14:1(9Z))	C40H78NO7P	714.5455341	No
12286	HMDB0011269	PC(P-18:1(11Z)/14:0)	C40H78NO7P	714.5455341	No
12286	HMDB0011302	PC(P-18:1(9Z)/14:0)	C40H78NO7P	714.5455341	No
12286	HMDB0013411	PC(o-16:1(9Z)/16:1(9Z))	C40H78NO7P	714.5455341	No
12320	HMDB0010586	PG(16:1(9Z)/16:1(9Z))	C38H71O10P	717.4708783	No
12345	HMDB0014646; C07240	Ritonavir	C37H48N6O5S2	719.302946	No
12350	HMDB0010571	PG(16:0/16:1(9Z))	C38H73O10P	719.4862273	No
12350	HMDB0010585	PG(16:1(9Z)/16:0)	C38H73O10P	719.4862273	No
12361	HMDB0029371	gamma2-Solamarine	C39H63NO11	720.4338649	No
12361	HMDB0033696	beta-Solanine	C39H63NO11	720.4338649	No
12371	HMDB0001094; C04146; CHEBI:16275	Octaprenyl diphosphate	C40H68O7P2	721.4356235	No
12371	HMDB0003023; C03427; CHEBI:17090	Prephytoene diphosphate	C40H68O7P2	721.4356235	No
12395	HMDB0007587	DG(20:5(5Z,8Z,11Z,14Z,17Z)/24:1(15Z)/0:0)	C47H80O5	723.5959865	No
12395	HMDB0007614	DG(22:0/22:6(4Z,7Z,10Z,13Z,16Z,19Z)/0:0)	C47H80O5	723.5959865	No
12395	HMDB0007641	DG(22:1(13Z)/22:5(4Z,7Z,10Z,13Z,16Z)/0:0)	C47H80O5	723.5959865	No
12395	HMDB0007642	DG(22:1(13Z)/22:5(7Z,10Z,13Z,16Z,19Z)/0:0)	C47H80O5	723.5959865	No
12395	HMDB0007669	DG(22:2(13Z,16Z)/22:4(7Z,10Z,13Z,16Z)/0:0)	C47H80O5	723.5959865	No
12395	HMDB0007697	DG(22:4(7Z,10Z,13Z,16Z)/22:2(13Z,16Z)/0:0)	C47H80O5	723.5959865	No
12395	HMDB0007725	DG(22:5(4Z,7Z,10Z,13Z,16Z)/22:1(13Z)/0:0)	C47H80O5	723.5959865	No
12395	HMDB0007754	DG(22:5(7Z,10Z,13Z,16Z,19Z)/22:1(13Z)/0:0)	C47H80O5	723.5959865	No
12395	HMDB0007782	DG(22:6(4Z,7Z,10Z,13Z,16Z,19Z)/22:0/0:0)	C47H80O5	723.5959865	No
12395	HMDB0007839	DG(24:1(15Z)/20:5(5Z,8Z,11Z,14Z,17Z)/0:0)	C47H80O5	723.5959865	No
12395	HMDB0056110	DG(22:0/0:0/22:6n3)	C47H80O5	723.5959865	No
12395	HMDB0056260	DG(22:1n9/0:0/22:5n6)	C47H80O5	723.5959865	No
12395	HMDB0056265	DG(22:1n9/0:0/22:5n3)	C47H80O5	723.5959865	No
12395	HMDB0056279	DG(24:1n9/0:0/20:5n3)	C47H80O5	723.5959865	No
12395	HMDB0056343	DG(22:2n6/0:0/22:4n6)	C47H80O5	723.5959865	No
12405	HMDB0007877; C00157	PC(14:0/18:4(6Z,9Z,12Z,15Z))	C40H72NO8P	724.490768	No
12405	HMDB0007908; C00157	PC(14:1(9Z)/18:3(6Z,9Z,12Z))	C40H72NO8P	724.490768	No
12405	HMDB0007909; C00157	PC(14:1(9Z)/18:3(9Z,12Z,15Z))	C40H72NO8P	724.490768	No
12405	HMDB0008164; C00157	PC(18:3(6Z,9Z,12Z)/14:1(9Z))	C40H72NO8P	724.490768	No
12405	HMDB0008197; C00157	PC(18:3(9Z,12Z,15Z)/14:1(9Z))	C40H72NO8P	724.490768	No
12405	HMDB0008229; C00157	PC(18:4(6Z,9Z,12Z,15Z)/14:0)	C40H72NO8P	724.490768	No
12405	HMDB0008904; C00350	PE(15:0/20:4(5Z,8Z,11Z,14Z))	C40H72NO8P	724.490768	No
12405	HMDB0008905; C00350	PE(15:0/20:4(8Z,11Z,14Z,17Z))	C40H72NO8P	724.490768	No
12405	HMDB0009384; C00350	PE(20:4(5Z,8Z,11Z,14Z)/15:0)	C40H72NO8P	724.490768	No
12405	HMDB0009417; C00350	PE(20:4(8Z,11Z,14Z,17Z)/15:0)	C40H72NO8P	724.490768	No
12405	HMDB0112987	PE-NMe(14:0/20:4(5Z,8Z,11Z,14Z))	C40H72NO8P	724.490768	No
12405	HMDB0112988	PE-NMe(14:0/20:4(8Z,11Z,14Z,17Z))	C40H72NO8P	724.490768	No
12405	HMDB0113005	PE-NMe(14:1(9Z)/20:3(5Z,8Z,11Z))	C40H72NO8P	724.490768	No
12405	HMDB0113006	PE-NMe(14:1(9Z)/20:3(8Z,11Z,14Z))	C40H72NO8P	724.490768	No
12405	HMDB0113045	PE-NMe(16:0/18:4(6Z,9Z,12Z,15Z))	C40H72NO8P	724.490768	No
12405	HMDB0113066	PE-NMe(16:1(9Z)/18:3(6Z,9Z,12Z))	C40H72NO8P	724.490768	No
12405	HMDB0113067	PE-NMe(16:1(9Z)/18:3(9Z,12Z,15Z))	C40H72NO8P	724.490768	No
12405	HMDB0113197	PE-NMe(18:3(6Z,9Z,12Z)/16:1(9Z))	C40H72NO8P	724.490768	No
12405	HMDB0113225	PE-NMe(18:3(9Z,12Z,15Z)/16:1(9Z))	C40H72NO8P	724.490768	No
12405	HMDB0113253	PE-NMe(18:4(6Z,9Z,12Z,15Z)/16:0)	C40H72NO8P	724.490768	No

12405	HMDB0113367	PE-NMe(20:3(5Z,8Z,11Z)/14:1(9Z))	C40H72NO8P	724.490768	No
12405	HMDB0113395	PE-NMe(20:3(8Z,11Z,14Z)/14:1(9Z))	C40H72NO8P	724.490768	No
12405	HMDB0113422	PE-NMe(20:4(5Z,8Z,11Z,14Z)/14:0)	C40H72NO8P	724.490768	No
12405	HMDB0113450	PE-NMe(20:4(8Z,11Z,14Z,17Z)/14:0)	C40H72NO8P	724.490768	No
12405	HMDB0113923	PE-NMe2(15:0/18:4(6Z,9Z,12Z,15Z))	C40H72NO8P	724.490768	No
12405	HMDB0114154	PE-NMe2(18:4(6Z,9Z,12Z,15Z)/15:0)	C40H72NO8P	724.490768	No
12463	HMDB0125179	({6-[(2-{[2-(3,4-dihydroxyphenyl)-7-hydroxy-5-oxo-5H-(	C34H35O18	730.1753475	No
12488	HMDB0008880; C00350	PE(14:1(9Z)/22:6(4Z,7Z,10Z,13Z,16Z,19Z))	C41H68NO8P	732.4611632	No
12488	HMDB0009129; C00350	PE(18:3(6Z,9Z,12Z)/18:4(6Z,9Z,12Z,15Z))	C41H68NO8P	732.4611632	No
12488	HMDB0009162; C00350	PE(18:3(9Z,12Z,15Z)/18:4(6Z,9Z,12Z,15Z))	C41H68NO8P	732.4611632	No
12488	HMDB0009193; C00350	PE(18:4(6Z,9Z,12Z,15Z)/18:3(6Z,9Z,12Z))	C41H68NO8P	732.4611632	No
12488	HMDB0009194; C00350	PE(18:4(6Z,9Z,12Z,15Z)/18:3(9Z,12Z,15Z))	C41H68NO8P	732.4611632	No
12488	HMDB0009680; C00350	PE(22:6(4Z,7Z,10Z,13Z,16Z,19Z)/14:1(9Z))	C41H68NO8P	732.4611632	No
12489	HMDB0012335	PS(14:0/18:1(9Z))	C38H72NO10P	732.4827831	No
12489	HMDB0012345	PS(14:1(9Z)/18:0)	C38H72NO10P	732.4827831	No
12489	HMDB0012355	PS(16:0/16:1(9Z))	C38H72NO10P	732.4827831	No
12489	HMDB0012365	PS(16:1(9Z)/16:0)	C38H72NO10P	732.4827831	No
12489	HMDB0012375	PS(18:0/14:1(9Z))	C38H72NO10P	732.4827831	No
12489	HMDB0012385	PS(18:1(9Z)/14:0)	C38H72NO10P	732.4827831	No
12489	HMDB0112266	PS(14:0/18:1(11Z))	C38H72NO10P	732.4827831	No
12489	HMDB0112269	PS(18:1(11Z)/14:0)	C38H72NO10P	732.4827831	No
12510	HMDB0008847; C00350	PE(14:0/22:6(4Z,7Z,10Z,13Z,16Z,19Z))	C41H70NO8P	734.4763653	No
12510	HMDB0008878; C00350	PE(14:1(9Z)/22:5(4Z,7Z,10Z,13Z,16Z))	C41H70NO8P	734.4763653	No
12510	HMDB0008879; C00350	PE(14:1(9Z)/22:5(7Z,10Z,13Z,16Z,19Z))	C41H70NO8P	734.4763653	No
12510	HMDB0008972; C00350	PE(16:1(9Z)/20:5(5Z,8Z,11Z,14Z,17Z))	C41H70NO8P	734.4763653	No
12510	HMDB0009096; C00350	PE(18:2(9Z,12Z)/18:4(6Z,9Z,12Z,15Z))	C41H70NO8P	734.4763653	No
12510	HMDB0009127; C00350	PE(18:3(6Z,9Z,12Z)/18:3(6Z,9Z,12Z))	C41H70NO8P	734.4763653	No
12510	HMDB0009128; C00350	PE(18:3(6Z,9Z,12Z)/18:3(9Z,12Z,15Z))	C41H70NO8P	734.4763653	No
12510	HMDB0009160; C00350	PE(18:3(9Z,12Z,15Z)/18:3(6Z,9Z,12Z))	C41H70NO8P	734.4763653	No
12510	HMDB0009161; C00350	PE(18:3(9Z,12Z,15Z)/18:3(9Z,12Z,15Z))	C41H70NO8P	734.4763653	No
12510	HMDB0009192; C00350	PE(18:4(6Z,9Z,12Z,15Z)/18:2(9Z,12Z))	C41H70NO8P	734.4763653	No
12510	HMDB0009452; C00350	PE(20:5(5Z,8Z,11Z,14Z,17Z)/16:1(9Z))	C41H70NO8P	734.4763653	No
12510	HMDB0009614; C00350	PE(22:5(4Z,7Z,10Z,13Z,16Z)/14:1(9Z))	C41H70NO8P	734.4763653	No
12510	HMDB0009647; C00350	PE(22:5(7Z,10Z,13Z,16Z,19Z)/14:1(9Z))	C41H70NO8P	734.4763653	No
12510	HMDB0009679; C00350	PE(22:6(4Z,7Z,10Z,13Z,16Z,19Z)/14:0)	C41H70NO8P	734.4763653	No
12510	HMDB0113909	PE-NMe2(14:1(9Z)/20:5(5Z,8Z,11Z,14Z,17Z))	C41H70NO8P	734.4763653	No
12510	HMDB0114382	PE-NMe2(20:5(5Z,8Z,11Z,14Z,17Z)/14:1(9Z))	C41H70NO8P	734.4763653	No
12512	HMDB0000614; C02737	PS(16:0/16:0)	C38H74NO10P	734.4959492	No
12512	HMDB0012334	PS(14:0/18:0)	C38H74NO10P	734.4959492	No
12512	HMDB0012374	PS(18:0/14:0)	C38H74NO10P	734.4959492	No
12537	HMDB0040991; C16302	Ternatin	C37H67N7O8	736.4968654	No
12571	HMDB0115051	PA(18:4(6Z,9Z,12Z,15Z)/22:6(4Z,7Z,10Z,13Z,16Z,19Z))	C43H65O8P	739.4368173	No
12571	HMDB0115214	PA(20:5(5Z,8Z,11Z,14Z,17Z)/20:5(5Z,8Z,11Z,14Z,17Z))	C43H65O8P	739.4368173	No
12571	HMDB0115412	PA(22:6(4Z,7Z,10Z,13Z,16Z,19Z)/18:4(6Z,9Z,12Z,15Z))	C43H65O8P	739.4368173	No
12580	HMDB0038822	Malvidin 3-(6-malonylglucoside) 5-glucoside	C32H37O20	740.178579	No
12586	HMDB0007941; C00157	PC(15:0/18:3(6Z,9Z,12Z))	C41H76NO8P	740.5231574	No
12586	HMDB0007942; C00157	PC(15:0/18:3(9Z,12Z,15Z))	C41H76NO8P	740.5231574	No
12586	HMDB0008165; C00157	PC(18:3(6Z,9Z,12Z)/15:0)	C41H76NO8P	740.5231574	No
12586	HMDB0008198; C00157	PC(18:3(9Z,12Z,15Z)/15:0)	C41H76NO8P	740.5231574	No
12586	HMDB0008876; C00350	PE(14:1(9Z)/22:2(13Z,16Z))	C41H76NO8P	740.5231574	No
12586	HMDB0008935; C00350	PE(16:0/20:3(5Z,8Z,11Z))	C41H76NO8P	740.5231574	No
12586	HMDB0008936; C00350	PE(16:0/20:3(8Z,11Z,14Z))	C41H76NO8P	740.5231574	No
12586	HMDB0008967; C00350	PE(16:1(9Z)/20:2(11Z,14Z))	C41H76NO8P	740.5231574	No
12586	HMDB0008995; C00350	PE(18:0/18:3(6Z,9Z,12Z))	C41H76NO8P	740.5231574	No
12586	HMDB0008996; C00350	PE(18:0/18:3(9Z,12Z,15Z))	C41H76NO8P	740.5231574	No
12586	HMDB0009027; C00350	PE(18:1(11Z)/18:2(9Z,12Z))	C41H76NO8P	740.5231574	No
12586	HMDB0009060; C00350	PE(18:1(9Z)/18:2(9Z,12Z))	C41H76NO8P	740.5231574	No
12586	HMDB0009091; C00350	PE(18:2(9Z,12Z)/18:1(11Z))	C41H76NO8P	740.5231574	No
12586	HMDB0009092; C00350	PE(18:2(9Z,12Z)/18:1(9Z))	C41H76NO8P	740.5231574	No
12586	HMDB0009123; C00350	PE(18:3(6Z,9Z,12Z)/18:0)	C41H76NO8P	740.5231574	No
12586	HMDB0009156; C00350	PE(18:3(9Z,12Z,15Z)/18:0)	C41H76NO8P	740.5231574	No

12586	HMDB0009287; C00350	PE(20:2(11Z,14Z)/16:1(9Z))	C41H76NO8P	740.5231574	No
12586	HMDB0009319; C00350	PE(20:3(5Z,8Z,11Z)/16:0)	C41H76NO8P	740.5231574	No
12586	HMDB0009352; C00350	PE(20:3(8Z,11Z,14Z)/16:0)	C41H76NO8P	740.5231574	No
12586	HMDB0009548; C00350	PE(22:2(13Z,16Z)/14:1(9Z))	C41H76NO8P	740.5231574	No
12586	HMDB0113026	PE-NMe(15:0/20:3(5Z,8Z,11Z))	C41H76NO8P	740.5231574	No
12586	HMDB0113027	PE-NMe(15:0/20:3(8Z,11Z,14Z))	C41H76NO8P	740.5231574	No
12586	HMDB0113368	PE-NMe(20:3(5Z,8Z,11Z)/15:0)	C41H76NO8P	740.5231574	No
12586	HMDB0113396	PE-NMe(20:3(8Z,11Z,14Z)/15:0)	C41H76NO8P	740.5231574	No
12586	HMDB0113885	PE-NMe2(14:0/20:3(5Z,8Z,11Z))	C41H76NO8P	740.5231574	No
12586	HMDB0113886	PE-NMe2(14:0/20:3(8Z,11Z,14Z))	C41H76NO8P	740.5231574	No
12586	HMDB0113904	PE-NMe2(14:1(9Z)/20:2(11Z,14Z))	C41H76NO8P	740.5231574	No
12586	HMDB0113944	PE-NMe2(16:0/18:3(6Z,9Z,12Z))	C41H76NO8P	740.5231574	No
12586	HMDB0113945	PE-NMe2(16:0/18:3(9Z,12Z,15Z))	C41H76NO8P	740.5231574	No
12586	HMDB0113966	PE-NMe2(16:1(9Z)/18:2(9Z,12Z))	C41H76NO8P	740.5231574	No
12586	HMDB0114069	PE-NMe2(18:2(9Z,12Z)/16:1(9Z))	C41H76NO8P	740.5231574	No
12586	HMDB0114097	PE-NMe2(18:3(6Z,9Z,12Z)/16:0)	C41H76NO8P	740.5231574	No
12586	HMDB0114126	PE-NMe2(18:3(9Z,12Z,15Z)/16:0)	C41H76NO8P	740.5231574	No
12586	HMDB0114240	PE-NMe2(20:2(11Z,14Z)/14:1(9Z))	C41H76NO8P	740.5231574	No
12586	HMDB0114268	PE-NMe2(20:3(5Z,8Z,11Z)/14:0)	C41H76NO8P	740.5231574	No
12586	HMDB0114296	PE-NMe2(20:3(8Z,11Z,14Z)/14:0)	C41H76NO8P	740.5231574	No
12596	HMDB0038345	Alliosterol 1-rhamnoside 16-galactoside	C39H66O13	741.4409456	No
12597	HMDB0114997	PA(18:3(6Z,9Z,12Z)/22:6(4Z,7Z,10Z,13Z,16Z,19Z))	C43H67O8P	741.4509757	No
12597	HMDB0115024	PA(18:3(9Z,12Z,15Z)/22:6(4Z,7Z,10Z,13Z,16Z,19Z))	C43H67O8P	741.4509757	No
12597	HMDB0115049	PA(18:4(6Z,9Z,12Z,15Z)/22:5(4Z,7Z,10Z,13Z,16Z))	C43H67O8P	741.4509757	No
12597	HMDB0115050	PA(18:4(6Z,9Z,12Z,15Z)/22:5(7Z,10Z,13Z,16Z,19Z))	C43H67O8P	741.4509757	No
12597	HMDB0115161	PA(20:4(5Z,8Z,11Z,14Z)/20:5(5Z,8Z,11Z,14Z,17Z))	C43H67O8P	741.4509757	No
12597	HMDB0115188	PA(20:4(8Z,11Z,14Z,17Z)/20:5(5Z,8Z,11Z,14Z,17Z))	C43H67O8P	741.4509757	No
12597	HMDB0115212	PA(20:5(5Z,8Z,11Z,14Z,17Z)/20:4(5Z,8Z,11Z,14Z))	C43H67O8P	741.4509757	No
12597	HMDB0115213	PA(20:5(5Z,8Z,11Z,14Z,17Z)/20:4(8Z,11Z,14Z,17Z))	C43H67O8P	741.4509757	No
12597	HMDB0115360	PA(22:5(4Z,7Z,10Z,13Z,16Z)/18:4(6Z,9Z,12Z,15Z))	C43H67O8P	741.4509757	No
12597	HMDB0115386	PA(22:5(7Z,10Z,13Z,16Z,19Z)/18:4(6Z,9Z,12Z,15Z))	C43H67O8P	741.4509757	No
12597	HMDB0115410	PA(22:6(4Z,7Z,10Z,13Z,16Z,19Z)/18:3(6Z,9Z,12Z))	C43H67O8P	741.4509757	No
12597	HMDB0115411	PA(22:6(4Z,7Z,10Z,13Z,16Z,19Z)/18:3(9Z,12Z,15Z))	C43H67O8P	741.4509757	No
12599	HMDB0116374	DG(11D3/13M5/0:0)	C46H78O7	741.5697973	No
12599	HMDB0116381	DG(11D5/11M5/0:0)	C46H78O7	741.5697973	No
12599	HMDB0116391	DG(11M3/13D5/0:0)	C46H78O7	741.5697973	No
12599	HMDB0116397	DG(11M5/11D5/0:0)	C46H78O7	741.5697973	No
12599	HMDB0116407	DG(13D5/11M3/0:0)	C46H78O7	741.5697973	No
12599	HMDB0116413	DG(13D5/9M5/0:0)	C46H78O7	741.5697973	No
12599	HMDB0116414	DG(13M5/11D3/0:0)	C46H78O7	741.5697973	No
12599	HMDB0116436	DG(9D5/13M5/0:0)	C46H78O7	741.5697973	No
12599	HMDB0116444	DG(9M5/13D5/0:0)	C46H78O7	741.5697973	No
12632	HMDB0010576	PG(16:0/18:3(6Z,9Z,12Z))	C40H73O10P	743.4869512	No
12632	HMDB0010577	PG(16:0/18:3(9Z,12Z,15Z))	C40H73O10P	743.4869512	No
12632	HMDB0010590	PG(16:1(9Z)/18:2(9Z,12Z))	C40H73O10P	743.4869512	No
12632	HMDB0010646	PG(18:2(9Z,12Z)/16:1(9Z))	C40H73O10P	743.4869512	No
12632	HMDB0010660	PG(18:3(6Z,9Z,12Z)/16:0)	C40H73O10P	743.4869512	No
12632	HMDB0010675	PG(18:3(9Z,12Z,15Z)/16:0)	C40H73O10P	743.4869512	No
12657	HMDB0030642	Bisisodiospyrin	C44H26O12	745.1351181	No
12677	HMDB0034699	Fumonisin A2	C36H61NO15	746.3940239	No
12681	HMDB0015342; C06912; CHEBI:3732	Clarithromycin	C38H69NO13	746.4690965	No
12684	HMDB0126176	6-{2-[6-carboxy-5-(2,4-dihydroxyphenyl)-3-(hydroxyme	C39H40O15	747.2297024	No
12684	HMDB0126177	6-[4-(6-carboxy-5-{2,6-dihydroxy-4-[6-hydroxy-7-(3-me	C39H40O15	747.2297024	No
12684	HMDB0126178	6-[2-(6-carboxy-5-{2,6-dihydroxy-4-[6-hydroxy-7-(3-me	C39H40O15	747.2297024	No
12684	HMDB0126179	6-[(2-{4-[6-carboxy-5-(2,4-dihydroxyphenyl)-3-(hydroxy	C39H40O15	747.2297024	No
12684	HMDB0126180	6-(2-{2,6-dihydroxy-4-[6-hydroxy-7-(3-methylbut-2-en-	C39H40O15	747.2297024	No
12684	HMDB0126182	6-[(2-{4-[6-carboxy-5-(2,4-dihydroxyphenyl)-3-methylc	C39H40O15	747.2297024	No
12684	HMDB0126183	6-{2-[6-carboxy-5-(2,4-dihydroxyphenyl)-3-methylcyclo	C39H40O15	747.2297024	No
12684	HMDB0126184	6-[4-(6-carboxy-5-{4-[5,6-dihydroxy-7-(3-methylbut-2-	C39H40O15	747.2297024	No
12684	HMDB0126185	6-[2-(6-carboxy-5-{4-[5,6-dihydroxy-7-(3-methylbut-2-	C39H40O15	747.2297024	No
12684	HMDB0126186	6-[(2-{4-[6-carboxy-5-(2,4-dihydroxyphenyl)-3-methylc	C39H40O15	747.2297024	No

12684	HMDB0126187	6-(2-{4-[5,6-dihydroxy-7-(3-methylbut-2-en-1-yl)-1-ber	C39H40O15	747.2297024	No
12684	HMDB0126188	6-{2-[6-carboxy-5-(2,4-dihydroxyphenyl)-4-hydroxy-3-r	C39H40O15	747.2297024	No
12684	HMDB0126189	6-[4-(6-carboxy-5-{2,6-dihydroxy-4-[6-hydroxy-7-(3-me	C39H40O15	747.2297024	No
12684	HMDB0126190	6-[2-(6-carboxy-5-{2,6-dihydroxy-4-[6-hydroxy-7-(3-me	C39H40O15	747.2297024	No
12684	HMDB0126191	6-[(2-{4-[6-carboxy-5-(2,4-dihydroxyphenyl)-4-hydroxy	C39H40O15	747.2297024	No
12684	HMDB0126192	6-(2-{2,6-dihydroxy-4-[6-hydroxy-7-(3-methylbut-2-en-	C39H40O15	747.2297024	No
12684	HMDB0126194	6-{2-[6-carboxy-5-(2,4-dihydroxyphenyl)-3-methylcyclo	C39H40O15	747.2297024	No
12684	HMDB0126195	6-[4-(6-carboxy-5-{2,6-dihydroxy-4-[6-hydroxy-7-(4-hy	C39H40O15	747.2297024	No
12684	HMDB0126196	6-[2-(6-carboxy-5-{2,6-dihydroxy-4-[6-hydroxy-7-(4-hy	C39H40O15	747.2297024	No
12684	HMDB0126197	6-[(2-{4-[6-carboxy-5-(2,4-dihydroxyphenyl)-3-methylc	C39H40O15	747.2297024	No
12684	HMDB0126198	6-(2-{2,6-dihydroxy-4-[6-hydroxy-7-(4-hydroxy-3-meth	C39H40O15	747.2297024	No
12684	HMDB0126200	6-{2-[6-carboxy-5-(2,4-dihydroxyphenyl)-3-methylcyclo	C39H40O15	747.2297024	No
12684	HMDB0126201	6-{4-[6-carboxy-5-(2,6-dihydroxy-4-{6-hydroxy-7-[(2E)-	C39H40O15	747.2297024	No
12684	HMDB0126202	6-{2-[6-carboxy-5-(2,6-dihydroxy-4-{6-hydroxy-7-[(2E)-	C39H40O15	747.2297024	No
12684	HMDB0126203	6-[(2-{4-[6-carboxy-5-(2,4-dihydroxyphenyl)-3-methylc	C39H40O15	747.2297024	No
12684	HMDB0126204	6-[2-(2,6-dihydroxy-4-{6-hydroxy-7-[(2E)-4-hydroxy-3-r	C39H40O15	747.2297024	No
12684	HMDB0126206	6-{2-[6-carboxy-5-(2,4-dihydroxyphenyl)-5-hydroxy-3-r	C39H40O15	747.2297024	No
12684	HMDB0126207	6-[4-(6-carboxy-5-{2,6-dihydroxy-4-[6-hydroxy-7-(3-me	C39H40O15	747.2297024	No
12684	HMDB0126208	6-[2-(6-carboxy-5-{2,6-dihydroxy-4-[6-hydroxy-7-(3-me	C39H40O15	747.2297024	No
12684	HMDB0126209	6-[(2-{4-[6-carboxy-5-(2,4-dihydroxyphenyl)-5-hydroxy	C39H40O15	747.2297024	No
12684	HMDB0126210	6-(2-{2,6-dihydroxy-4-[6-hydroxy-7-(3-methylbut-2-en-	C39H40O15	747.2297024	No
12684	HMDB0126211	6-{2-[6-carboxy-5-(2,4-dihydroxyphenyl)-3-methylcyclo	C39H40O15	747.2297024	No
12684	HMDB0126212	6-{4-[6-carboxy-5-(4-{7-[(3,3-dimethyloxiran-2-yl)meth	C39H40O15	747.2297024	No
12684	HMDB0126213	6-{2-[6-carboxy-5-(4-{7-[(3,3-dimethyloxiran-2-yl)meth	C39H40O15	747.2297024	No
12684	HMDB0126214	6-[(2-{4-[6-carboxy-5-(2,4-dihydroxyphenyl)-3-methylc	C39H40O15	747.2297024	No
12684	HMDB0126215	6-[6-(2,4-dihydroxyphenyl)-2-(4-{7-[(3,3-dimethyloxira	C39H40O15	747.2297024	No
12684	HMDB0126216	2-{2-[(6-carboxy-3,4,5-trihydroxyoxan-2-yl)oxy]-6-hydr	C39H40O15	747.2297024	No
12684	HMDB0126217	4-{4-[(6-carboxy-3,4,5-trihydroxyoxan-2-yl)oxy]-2-hydr	C39H40O15	747.2297024	No
12684	HMDB0126218	4-{2-[(6-carboxy-3,4,5-trihydroxyoxan-2-yl)oxy]-4-hydr	C39H40O15	747.2297024	No
12684	HMDB0126219	2-(4-{6-[(6-carboxy-3,4,5-trihydroxyoxan-2-yl)oxy]-7-(3	C39H40O15	747.2297024	No
12684	HMDB0126220	6-(2-{2,6-dihydroxy-4-[6-hydroxy-7-(3-methylbut-2-en-	C39H40O15	747.2297024	No
12684	HMDB0126221	6-{3-[6-carboxy-5-(2,4-dihydroxyphenyl)-3-methylcyclo	C39H40O15	747.2297024	No
12684	HMDB0126222	6-{2-[6-carboxy-5-(2,4-dihydroxyphenyl)-3-methylcyclo	C39H40O15	747.2297024	No
12684	HMDB0126223	6-[4-(6-carboxy-3-methyl-5-{2,3,6-trihydroxy-4-[6-hydr	C39H40O15	747.2297024	No
12684	HMDB0126224	6-[2-(6-carboxy-3-methyl-5-{2,3,6-trihydroxy-4-[6-hydr	C39H40O15	747.2297024	No
12684	HMDB0126225	6-{2-[6-carboxy-5-(2,4-dihydroxyphenyl)-3-methylcyclo	C39H40O15	747.2297024	No
12684	HMDB0126226	6-[(2-{4-[6-carboxy-5-(2,4-dihydroxyphenyl)-3-methylc	C39H40O15	747.2297024	No
12684	HMDB0126227	6-[6-(2,4-dihydroxyphenyl)-4-methyl-2-{2,3,6-trihydrox	C39H40O15	747.2297024	No
12684	HMDB0126229	6-[5-(6-carboxy-5-{2,6-dihydroxy-4-[6-hydroxy-7-(3-me	C39H40O15	747.2297024	No
12684	HMDB0126230	6-{2-[6-carboxy-3-methyl-5-(2,4,5-trihydroxyphenyl)cy	C39H40O15	747.2297024	No
12684	HMDB0126231	6-[4-(6-carboxy-5-{2,6-dihydroxy-4-[6-hydroxy-7-(3-me	C39H40O15	747.2297024	No
12684	HMDB0126232	6-[2-(6-carboxy-5-{2,6-dihydroxy-4-[6-hydroxy-7-(3-me	C39H40O15	747.2297024	No
12684	HMDB0126233	6-[(2-{4-[6-carboxy-3-methyl-5-(2,4,5-trihydroxypheny	C39H40O15	747.2297024	No
12684	HMDB0126234	6-(2-{2,6-dihydroxy-4-[6-hydroxy-7-(3-methylbut-2-en-	C39H40O15	747.2297024	No
12684	HMDB0126236	6-{2-[6-carboxy-5-(2,4-dihydroxyphenyl)-6-hydroxy-3-r	C39H40O15	747.2297024	No
12684	HMDB0126237	6-[4-(6-carboxy-5-{2,6-dihydroxy-4-[6-hydroxy-7-(3-me	C39H40O15	747.2297024	No
12684	HMDB0126238	6-[2-(6-carboxy-5-{2,6-dihydroxy-4-[6-hydroxy-7-(3-me	C39H40O15	747.2297024	No
12684	HMDB0126239	6-[(2-{4-[6-carboxy-5-(2,4-dihydroxyphenyl)-6-hydroxy	C39H40O15	747.2297024	No
12684	HMDB0126240	6-(2-{2,6-dihydroxy-4-[6-hydroxy-7-(3-methylbut-2-en-	C39H40O15	747.2297024	No
12687	HMDB0001916; C06838	Azythromycin	C38H72N2O12	747.4989032	No
12687	HMDB0014352; C06838; CHEBI:2955	Azithromycin	C38H72N2O12	747.4989032	No
12687	HMDB0114893	PA(18:0/22:6(4Z,7Z,10Z,13Z,16Z,19Z))	C43H73O8P	747.4989032	No
12687	HMDB0114916	PA(18:1(11Z)/22:5(4Z,7Z,10Z,13Z,16Z))	C43H73O8P	747.4989032	No
12687	HMDB0114917	PA(18:1(11Z)/22:5(7Z,10Z,13Z,16Z,19Z))	C43H73O8P	747.4989032	No
12687	HMDB0114941	PA(18:1(9Z)/22:5(4Z,7Z,10Z,13Z,16Z))	C43H73O8P	747.4989032	No
12687	HMDB0114942	PA(18:1(9Z)/22:5(7Z,10Z,13Z,16Z,19Z))	C43H73O8P	747.4989032	No
12687	HMDB0114967	PA(18:2(9Z,12Z)/22:4(7Z,10Z,13Z,16Z))	C43H73O8P	747.4989032	No
12687	HMDB0115047	PA(18:4(6Z,9Z,12Z,15Z)/22:2(13Z,16Z))	C43H73O8P	747.4989032	No
12687	HMDB0115108	PA(20:1(11Z)/20:5(5Z,8Z,11Z,14Z,17Z))	C43H73O8P	747.4989032	No
12687	HMDB0115132	PA(20:3(5Z,8Z,11Z)/20:3(5Z,8Z,11Z))	C43H73O8P	747.4989032	No
12687	HMDB0115210	PA(20:5(5Z,8Z,11Z,14Z,17Z)/20:1(11Z))	C43H73O8P	747.4989032	No

12687	HMDB0115307	PA(22:2(13Z,16Z)/18:4(6Z,9Z,12Z,15Z))	C43H73O8P	747.4989032	No
12687	HMDB0115331	PA(22:4(7Z,10Z,13Z,16Z)/18:2(9Z,12Z))	C43H73O8P	747.4989032	No
12687	HMDB0115355	PA(22:5(4Z,7Z,10Z,13Z,16Z)/18:1(11Z))	C43H73O8P	747.4989032	No
12687	HMDB0115356	PA(22:5(4Z,7Z,10Z,13Z,16Z)/18:1(9Z))	C43H73O8P	747.4989032	No
12687	HMDB0115381	PA(22:5(7Z,10Z,13Z,16Z,19Z)/18:1(11Z))	C43H73O8P	747.4989032	No
12687	HMDB0115382	PA(22:5(7Z,10Z,13Z,16Z,19Z)/18:1(9Z))	C43H73O8P	747.4989032	No
12687	HMDB0115406	PA(22:6(4Z,7Z,10Z,13Z,16Z,19Z)/18:0)	C43H73O8P	747.4989032	No
12687	HMDB0115494	PA(20:3(5Z,8Z,11Z)/20:3(8Z,11Z,14Z))	C43H73O8P	747.4989032	No
12687	HMDB0115496	PA(20:3(8Z,11Z,14Z)/20:3(5Z,8Z,11Z))	C43H73O8P	747.4989032	No
12687	HMDB0115497	PA(20:3(8Z,11Z,14Z)/20:3(8Z,11Z,14Z))	C43H73O8P	747.4989032	No
12687	HMDB0115498	PA(20:4(5Z,8Z,11Z,14Z)/20:2(11Z,14Z))	C43H73O8P	747.4989032	No
12687	HMDB0115500	PA(20:4(8Z,11Z,14Z,17Z)/20:2(11Z,14Z))	C43H73O8P	747.4989032	No
12687	HMDB0115548	PA(20:2(11Z,14Z)/20:4(5Z,8Z,11Z,14Z))	C43H73O8P	747.4989032	No
12687	HMDB0115549	PA(20:2(11Z,14Z)/20:4(8Z,11Z,14Z,17Z))	C43H73O8P	747.4989032	No
12688	HMDB0030449	20-Deoxynarasin	C43H72O10	747.5064032	No
12689	HMDB0010573	PG(16:0/18:1(11Z))	C40H77O10P	747.5158519	No
12689	HMDB0010574	PG(16:0/18:1(9Z))	C40H77O10P	747.5158519	No
12689	HMDB0010587	PG(16:1(9Z)/18:0)	C40H77O10P	747.5158519	No
12689	HMDB0010601	PG(18:0/16:1(9Z))	C40H77O10P	747.5158519	No
12689	HMDB0010615	PG(18:1(11Z)/16:0)	C40H77O10P	747.5158519	No
12689	HMDB0010630	PG(18:1(9Z)/16:0)	C40H77O10P	747.5158519	No
12701	HMDB0009413; C00350	PE(20:4(5Z,8Z,11Z,14Z)/P-18:1(11Z))	C43H76NO7P	748.5278911	No
12701	HMDB0009414; C00350	PE(20:4(5Z,8Z,11Z,14Z)/P-18:1(9Z))	C43H76NO7P	748.5278911	No
12701	HMDB0009446; C00350	PE(20:4(8Z,11Z,14Z,17Z)/P-18:1(11Z))	C43H76NO7P	748.5278911	No
12701	HMDB0009447; C00350	PE(20:4(8Z,11Z,14Z,17Z)/P-18:1(9Z))	C43H76NO7P	748.5278911	No
12701	HMDB0009478; C00350	PE(20:5(5Z,8Z,11Z,14Z,17Z)/P-18:0)	C43H76NO7P	748.5278911	No
12701	HMDB0009642; C00350	PE(22:5(4Z,7Z,10Z,13Z,16Z)/P-16:0)	C43H76NO7P	748.5278911	No
12701	HMDB0009675; C00350	PE(22:5(7Z,10Z,13Z,16Z,19Z)/P-16:0)	C43H76NO7P	748.5278911	No
12701	HMDB0011359	PE(P-16:0/22:5(4Z,7Z,10Z,13Z,16Z))	C43H76NO7P	748.5278911	No
12701	HMDB0011360	PE(P-16:0/22:5(7Z,10Z,13Z,16Z,19Z))	C43H76NO7P	748.5278911	No
12701	HMDB0011387	PE(P-18:0/20:5(5Z,8Z,11Z,14Z,17Z))	C43H76NO7P	748.5278911	No
12701	HMDB0011418	PE(P-18:1(11Z)/20:4(5Z,8Z,11Z,14Z))	C43H76NO7P	748.5278911	No
12701	HMDB0011419	PE(P-18:1(11Z)/20:4(8Z,11Z,14Z,17Z))	C43H76NO7P	748.5278911	No
12701	HMDB0011451	PE(P-18:1(9Z)/20:4(5Z,8Z,11Z,14Z))	C43H76NO7P	748.5278911	No
12701	HMDB0011452	PE(P-18:1(9Z)/20:4(8Z,11Z,14Z,17Z))	C43H76NO7P	748.5278911	No
12712	HMDB0114891	PA(18:0/22:5(4Z,7Z,10Z,13Z,16Z))	C43H75O8P	749.514689	No
12712	HMDB0114892	PA(18:0/22:5(7Z,10Z,13Z,16Z,19Z))	C43H75O8P	749.514689	No
12712	HMDB0114915	PA(18:1(11Z)/22:4(7Z,10Z,13Z,16Z))	C43H75O8P	749.514689	No
12712	HMDB0114940	PA(18:1(9Z)/22:4(7Z,10Z,13Z,16Z))	C43H75O8P	749.514689	No
12712	HMDB0114993	PA(18:3(6Z,9Z,12Z)/22:2(13Z,16Z))	C43H75O8P	749.514689	No
12712	HMDB0115020	PA(18:3(9Z,12Z,15Z)/22:2(13Z,16Z))	C43H75O8P	749.514689	No
12712	HMDB0115046	PA(18:4(6Z,9Z,12Z,15Z)/22:1(13Z))	C43H75O8P	749.514689	No
12712	HMDB0115080	PA(20:0/20:5(5Z,8Z,11Z,14Z,17Z))	C43H75O8P	749.514689	No
12712	HMDB0115106	PA(20:1(11Z)/20:4(5Z,8Z,11Z,14Z))	C43H75O8P	749.514689	No
12712	HMDB0115107	PA(20:1(11Z)/20:4(8Z,11Z,14Z,17Z))	C43H75O8P	749.514689	No
12712	HMDB0115157	PA(20:4(5Z,8Z,11Z,14Z)/20:1(11Z))	C43H75O8P	749.514689	No
12712	HMDB0115184	PA(20:4(8Z,11Z,14Z,17Z)/20:1(11Z))	C43H75O8P	749.514689	No
12712	HMDB0115209	PA(20:5(5Z,8Z,11Z,14Z,17Z)/20:0)	C43H75O8P	749.514689	No
12712	HMDB0115281	PA(22:1(13Z)/18:4(6Z,9Z,12Z,15Z))	C43H75O8P	749.514689	No
12712	HMDB0115305	PA(22:2(13Z,16Z)/18:3(6Z,9Z,12Z))	C43H75O8P	749.514689	No
12712	HMDB0115306	PA(22:2(13Z,16Z)/18:3(9Z,12Z,15Z))	C43H75O8P	749.514689	No
12712	HMDB0115329	PA(22:4(7Z,10Z,13Z,16Z)/18:1(11Z))	C43H75O8P	749.514689	No
12712	HMDB0115330	PA(22:4(7Z,10Z,13Z,16Z)/18:1(9Z))	C43H75O8P	749.514689	No
12712	HMDB0115354	PA(22:5(4Z,7Z,10Z,13Z,16Z)/18:0)	C43H75O8P	749.514689	No
12712	HMDB0115380	PA(22:5(7Z,10Z,13Z,16Z,19Z)/18:0)	C43H75O8P	749.514689	No
12712	HMDB0115546	PA(20:2(11Z,14Z)/20:3(5Z,8Z,11Z))	C43H75O8P	749.514689	No
12712	HMDB0115547	PA(20:2(11Z,14Z)/20:3(8Z,11Z,14Z))	C43H75O8P	749.514689	No
12712	HMDB0115560	PA(20:3(5Z,8Z,11Z)/20:2(11Z,14Z))	C43H75O8P	749.514689	No
12712	HMDB0115574	PA(20:3(8Z,11Z,14Z)/20:2(11Z,14Z))	C43H75O8P	749.514689	No
12743	HMDB0007883; C00157	PC(14:0/20:4(5Z,8Z,11Z,14Z))	C42H76NO8P	752.5243301	No
12743	HMDB0007884; C00157	PC(14:0/20:4(8Z,11Z,14Z,17Z))	C42H76NO8P	752.5243301	No

12743	HMDB0007914; C00157	PC(14:1(9Z)/20:3(5Z,8Z,11Z))	C42H76NO8P	752.5243301	No
12743	HMDB0007915; C00157	PC(14:1(9Z)/20:3(8Z,11Z,14Z))	C42H76NO8P	752.5243301	No
12743	HMDB0007976; C00157	PC(16:0/18:4(6Z,9Z,12Z,15Z))	C42H76NO8P	752.5243301	No
12743	HMDB0008007; C00157	PC(16:1(9Z)/18:3(6Z,9Z,12Z))	C42H76NO8P	752.5243301	No
12743	HMDB0008008; C00157	PC(16:1(9Z)/18:3(9Z,12Z,15Z))	C42H76NO8P	752.5243301	No
12743	HMDB0008167; C00157	PC(18:3(6Z,9Z,12Z)/16:1(9Z))	C42H76NO8P	752.5243301	No
12743	HMDB0008200; C00157	PC(18:3(9Z,12Z,15Z)/16:1(9Z))	C42H76NO8P	752.5243301	No
12743	HMDB0008232; C00157	PC(18:4(6Z,9Z,12Z,15Z)/16:0)	C42H76NO8P	752.5243301	No
12743	HMDB0008362; C00157	PC(20:3(5Z,8Z,11Z)/14:1(9Z))	C42H76NO8P	752.5243301	No
12743	HMDB0008395; C00157	PC(20:3(8Z,11Z,14Z)/14:1(9Z))	C42H76NO8P	752.5243301	No
12743	HMDB0008426; C00157	PC(20:4(5Z,8Z,11Z,14Z)/14:0)	C42H76NO8P	752.5243301	No
12743	HMDB0008459; C00157	PC(20:4(8Z,11Z,14Z,17Z)/14:0)	C42H76NO8P	752.5243301	No
12743	HMDB0008910; C00350	PE(15:0/22:4(7Z,10Z,13Z,16Z))	C42H76NO8P	752.5243301	No
12743	HMDB0009582; C00350	PE(22:4(7Z,10Z,13Z,16Z)/15:0)	C42H76NO8P	752.5243301	No
12743	HMDB0112993	PE-NMe(14:0/22:4(7Z,10Z,13Z,16Z))	C42H76NO8P	752.5243301	No
12743	HMDB0113050	PE-NMe(16:0/20:4(5Z,8Z,11Z,14Z))	C42H76NO8P	752.5243301	No
12743	HMDB0113070	PE-NMe(16:1(9Z)/20:3(5Z,8Z,11Z))	C42H76NO8P	752.5243301	No
12743	HMDB0113071	PE-NMe(16:1(9Z)/20:3(8Z,11Z,14Z))	C42H76NO8P	752.5243301	No
12743	HMDB0113092	PE-NMe(18:0/18:4(6Z,9Z,12Z,15Z))	C42H76NO8P	752.5243301	No
12743	HMDB0113117	PE-NMe(18:1(11Z)/18:3(6Z,9Z,12Z))	C42H76NO8P	752.5243301	No
12743	HMDB0113118	PE-NMe(18:1(11Z)/18:3(9Z,12Z,15Z))	C42H76NO8P	752.5243301	No
12743	HMDB0113144	PE-NMe(18:1(9Z)/18:3(6Z,9Z,12Z))	C42H76NO8P	752.5243301	No
12743	HMDB0113145	PE-NMe(18:1(9Z)/18:3(9Z,12Z,15Z))	C42H76NO8P	752.5243301	No
12743	HMDB0113172	PE-NMe(18:2(9Z,12Z)/18:2(9Z,12Z))	C42H76NO8P	752.5243301	No
12743	HMDB0113199	PE-NMe(18:3(6Z,9Z,12Z)/18:1(11Z))	C42H76NO8P	752.5243301	No
12743	HMDB0113200	PE-NMe(18:3(6Z,9Z,12Z)/18:1(9Z))	C42H76NO8P	752.5243301	No
12743	HMDB0113227	PE-NMe(18:3(9Z,12Z,15Z)/18:1(11Z))	C42H76NO8P	752.5243301	No
12743	HMDB0113228	PE-NMe(18:3(9Z,12Z,15Z)/18:1(9Z))	C42H76NO8P	752.5243301	No
12743	HMDB0113255	PE-NMe(18:4(6Z,9Z,12Z,15Z)/18:0)	C42H76NO8P	752.5243301	No
12743	HMDB0113370	PE-NMe(20:3(5Z,8Z,11Z)/16:1(9Z))	C42H76NO8P	752.5243301	No
12743	HMDB0113398	PE-NMe(20:3(8Z,11Z,14Z)/16:1(9Z))	C42H76NO8P	752.5243301	No
12743	HMDB0113425	PE-NMe(20:4(5Z,8Z,11Z,14Z)/16:0)	C42H76NO8P	752.5243301	No
12743	HMDB0113453	PE-NMe(20:4(8Z,11Z,14Z,17Z)/16:0)	C42H76NO8P	752.5243301	No
12743	HMDB0113592	PE-NMe(22:4(7Z,10Z,13Z,16Z)/14:0)	C42H76NO8P	752.5243301	No
12743	HMDB0113928	PE-NMe2(15:0/20:4(5Z,8Z,11Z,14Z))	C42H76NO8P	752.5243301	No
12743	HMDB0113929	PE-NMe2(15:0/20:4(8Z,11Z,14Z,17Z))	C42H76NO8P	752.5243301	No
12743	HMDB0114326	PE-NMe2(20:4(5Z,8Z,11Z,14Z)/15:0)	C42H76NO8P	752.5243301	No
12743	HMDB0114354	PE-NMe2(20:4(8Z,11Z,14Z,17Z)/15:0)	C42H76NO8P	752.5243301	No
12743	HMDB0116725	PE-NMe(16:0/20:4(8Z,11Z,14Z,17Z))	C42H76NO8P	752.5243301	No
12750	HMDB0034994; C17544	Chikusetsusaponin Ia	C41H70O12	753.4778137	No
12750	HMDB0062593; CHEBI:77491	Ginsenoside Mc	C41H70O12	753.4778137	No
12782	HMDB0009203; C00350	PE(18:4(6Z,9Z,12Z,15Z)/20:5(5Z,8Z,11Z,14Z,17Z))	C43H68NO8P	756.4631955	No
12782	HMDB0009459; C00350	PE(20:5(5Z,8Z,11Z,14Z,17Z)/18:4(6Z,9Z,12Z,15Z))	C43H68NO8P	756.4631955	No
12785	HMDB0007880; C00157	PC(14:0/20:2(11Z,14Z))	C42H80NO8P	756.552258	No
12785	HMDB0007912; C00157	PC(14:1(9Z)/20:1(11Z))	C42H80NO8P	756.552258	No
12785	HMDB0007973; C00157	PC(16:0/18:2(9Z,12Z))	C42H80NO8P	756.552258	No
12785	HMDB0008004; C00157	PC(16:1(9Z)/18:1(11Z))	C42H80NO8P	756.552258	No
12785	HMDB0008005; C00157	PC(16:1(9Z)/18:1(9Z))	C42H80NO8P	756.552258	No
12785	HMDB0008068; C00157	PC(18:1(11Z)/16:1(9Z))	C42H80NO8P	756.552258	No
12785	HMDB0008101; C00157	PC(18:1(9Z)/16:1(9Z))	C42H80NO8P	756.552258	No
12785	HMDB0008133; C00157	PC(18:2(9Z,12Z)/16:0)	C42H80NO8P	756.552258	No
12785	HMDB0008296; C00157	PC(20:1(11Z)/14:1(9Z))	C42H80NO8P	756.552258	No
12785	HMDB0008328; C00157	PC(20:2(11Z,14Z)/14:0)	C42H80NO8P	756.552258	No
12785	HMDB0008909; C00350	PE(15:0/22:2(13Z,16Z))	C42H80NO8P	756.552258	No
12785	HMDB0009549; C00350	PE(22:2(13Z,16Z)/15:0)	C42H80NO8P	756.552258	No
12785	HMDB0010565	PE-NMe(18:1(9Z)/18:1(9Z))	C42H80NO8P	756.552258	No
12785	HMDB0112966	PE-NMe(16:1(9Z)/20:1(11Z))	C42H80NO8P	756.552258	No
12785	HMDB0112967	PE-NMe(18:1(9Z)/18:1(11Z))	C42H80NO8P	756.552258	No
12785	HMDB0112968	PE-NMe(18:1(11Z)/18:1(9Z))	C42H80NO8P	756.552258	No
12785	HMDB0112969	PE-NMe(18:1(11Z)/18:1(11Z))	C42H80NO8P	756.552258	No
12785	HMDB0112992	PE-NMe(14:0/22:2(13Z,16Z))	C42H80NO8P	756.552258	No

12785	HMDB0113010	PE-NMe(14:1(9Z)/22:1(13Z))	C42H80NO8P	756.552258	No
12785	HMDB0113047	PE-NMe(16:0/20:2(11Z,14Z))	C42H80NO8P	756.552258	No
12785	HMDB0113089	PE-NMe(18:0/18:2(9Z,12Z))	C42H80NO8P	756.552258	No
12785	HMDB0113169	PE-NMe(18:2(9Z,12Z)/18:0)	C42H80NO8P	756.552258	No
12785	HMDB0113312	PE-NMe(20:1(11Z)/16:1(9Z))	C42H80NO8P	756.552258	No
12785	HMDB0113340	PE-NMe(20:2(11Z,14Z)/16:0)	C42H80NO8P	756.552258	No
12785	HMDB0113536	PE-NMe(22:1(13Z)/14:1(9Z))	C42H80NO8P	756.552258	No
12785	HMDB0113563	PE-NMe(22:2(13Z,16Z)/14:0)	C42H80NO8P	756.552258	No
12785	HMDB0113925	PE-NMe2(15:0/20:2(11Z,14Z))	C42H80NO8P	756.552258	No
12785	HMDB0114241	PE-NMe2(20:2(11Z,14Z)/15:0)	C42H80NO8P	756.552258	No
12809	HMDB0009137; C00350	PE(18:3(6Z,9Z,12Z)/20:5(5Z,8Z,11Z,14Z,17Z))	C43H70NO8P	758.4757498	No
12809	HMDB0009170; C00350	PE(18:3(9Z,12Z,15Z)/20:5(5Z,8Z,11Z,14Z,17Z))	C43H70NO8P	758.4757498	No
12809	HMDB0009201; C00350	PE(18:4(6Z,9Z,12Z,15Z)/20:4(5Z,8Z,11Z,14Z))	C43H70NO8P	758.4757498	No
12809	HMDB0009202; C00350	PE(18:4(6Z,9Z,12Z,15Z)/20:4(8Z,11Z,14Z,17Z))	C43H70NO8P	758.4757498	No
12809	HMDB0009393; C00350	PE(20:4(5Z,8Z,11Z,14Z)/18:4(6Z,9Z,12Z,15Z))	C43H70NO8P	758.4757498	No
12809	HMDB0009426; C00350	PE(20:4(8Z,11Z,14Z,17Z)/18:4(6Z,9Z,12Z,15Z))	C43H70NO8P	758.4757498	No
12809	HMDB0009457; C00350	PE(20:5(5Z,8Z,11Z,14Z,17Z)/18:3(6Z,9Z,12Z))	C43H70NO8P	758.4757498	No
12809	HMDB0009458; C00350	PE(20:5(5Z,8Z,11Z,14Z,17Z)/18:3(9Z,12Z,15Z))	C43H70NO8P	758.4757498	No
12809	HMDB0114163	PE-NMe2(18:4(6Z,9Z,12Z,15Z)/18:4(6Z,9Z,12Z,15Z))	C43H70NO8P	758.4757498	No
12816	HMDB0007879; C00157	PC(14:0/20:1(11Z))	C42H82NO8P	758.5716296	No
12816	HMDB0007911; C00157	PC(14:1(9Z)/20:0)	C42H82NO8P	758.5716296	No
12816	HMDB0007971; C00157	PC(16:0/18:1(11Z))	C42H82NO8P	758.5716296	No
12816	HMDB0007972; C00157	PC(16:0/18:1(9Z))	C42H82NO8P	758.5716296	No
12816	HMDB0008003; C00157	PC(16:1(9Z)/18:0)	C42H82NO8P	758.5716296	No
12816	HMDB0008035; C00157	PC(18:0/16:1(9Z))	C42H82NO8P	758.5716296	No
12816	HMDB0008067; C00157	PC(18:1(11Z)/16:0)	C42H82NO8P	758.5716296	No
12816	HMDB0008100; C00157	PC(18:1(9Z)/16:0)	C42H82NO8P	758.5716296	No
12816	HMDB0008263; C00157	PC(20:0/14:1(9Z))	C42H82NO8P	758.5716296	No
12816	HMDB0008295; C00157	PC(20:1(11Z)/14:0)	C42H82NO8P	758.5716296	No
12816	HMDB0008908; C00350	PE(15:0/22:1(13Z))	C42H82NO8P	758.5716296	No
12816	HMDB0009516; C00350	PE(22:1(13Z)/15:0)	C42H82NO8P	758.5716296	No
12816	HMDB0112970	PE-NMe(18:0/18:1(9Z))	C42H82NO8P	758.5716296	No
12816	HMDB0112971	PE-NMe(18:0/18:1(11Z))	C42H82NO8P	758.5716296	No
12816	HMDB0112972	PE-NMe(14:1(9Z)/22:0)	C42H82NO8P	758.5716296	No
12816	HMDB0112973	PE-NMe(16:0/20:1(11Z))	C42H82NO8P	758.5716296	No
12816	HMDB0112974	PE-NMe(16:1(9Z)/20:0)	C42H82NO8P	758.5716296	No
12816	HMDB0112991	PE-NMe(14:0/22:1(13Z))	C42H82NO8P	758.5716296	No
12816	HMDB0113115	PE-NMe(18:1(11Z)/18:0)	C42H82NO8P	758.5716296	No
12816	HMDB0113142	PE-NMe(18:1(9Z)/18:0)	C42H82NO8P	758.5716296	No
12816	HMDB0113283	PE-NMe(20:0/16:1(9Z))	C42H82NO8P	758.5716296	No
12816	HMDB0113311	PE-NMe(20:1(11Z)/16:0)	C42H82NO8P	758.5716296	No
12816	HMDB0113509	PE-NMe(22:0/14:1(9Z))	C42H82NO8P	758.5716296	No
12816	HMDB0113535	PE-NMe(22:1(13Z)/14:0)	C42H82NO8P	758.5716296	No
12816	HMDB0113864	PE-NMe2(15:0/20:1(11Z))	C42H82NO8P	758.5716296	No
12816	HMDB0114212	PE-NMe2(20:1(11Z)/15:0)	C42H82NO8P	758.5716296	No
12827	HMDB0007645	DG(22:1(13Z)/24:1(15Z)/0:0)	C49H92O5	759.6858172	No
12827	HMDB0007673	DG(22:2(13Z,16Z)/24:0/0:0)	C49H92O5	759.6858172	No
12827	HMDB0007813	DG(24:0/22:2(13Z,16Z)/0:0)	C49H92O5	759.6858172	No
12827	HMDB0007841	DG(24:1(15Z)/22:1(13Z)/0:0)	C49H92O5	759.6858172	No
12827	HMDB0047907	TG(14:1(9Z)/14:1(9Z)/o-18:0)	C49H92O5	759.6858172	No
12827	HMDB0048391	TG(14:1(9Z)/o-18:0/14:1(9Z))	C49H92O5	759.6858172	No
12827	HMDB0056125	DG(24:0/0:0/22:2n6)	C49H92O5	759.6858172	No
12827	HMDB0056252	DG(22:1n9/0:0/24:1n9)	C49H92O5	759.6858172	No
12840	HMDB0012357; CHEBI:34086	PS(16:0/18:1(9Z))	C40H76NO10P	760.5116753	No
12840	HMDB0012367	PS(16:1(9Z)/18:0)	C40H76NO10P	760.5116753	No
12840	HMDB0012377	PS(18:0/16:1(9Z))	C40H76NO10P	760.5116753	No
12840	HMDB0012387	PS(18:1(9Z)/16:0)	C40H76NO10P	760.5116753	No
12840	HMDB0112267	PS(16:0/18:1(11Z))	C40H76NO10P	760.5116753	No
12840	HMDB0112270	PS(18:1(11Z)/16:0)	C40H76NO10P	760.5116753	No
12840	HMDB0112274	PS(14:0/20:1(11Z))	C40H76NO10P	760.5116753	No
12840	HMDB0112277	PS(14:1(9Z)/20:0)	C40H76NO10P	760.5116753	No

12840	HMDB0112512	PS(20:0/14:1(9Z))	C40H76NO10P	760.5116753	No
12840	HMDB0112540	PS(20:1(11Z)/14:0)	C40H76NO10P	760.5116753	No
12841	HMDB0012357; CHEBI:34086	PS(16:0/18:1(9Z))	C40H76NO10P	760.5153277	No
12841	HMDB0012367	PS(16:1(9Z)/18:0)	C40H76NO10P	760.5153277	No
12841	HMDB0012377	PS(18:0/16:1(9Z))	C40H76NO10P	760.5153277	No
12841	HMDB0012387	PS(18:1(9Z)/16:0)	C40H76NO10P	760.5153277	No
12841	HMDB0112267	PS(16:0/18:1(11Z))	C40H76NO10P	760.5153277	No
12841	HMDB0112270	PS(18:1(11Z)/16:0)	C40H76NO10P	760.5153277	No
12841	HMDB0112274	PS(14:0/20:1(11Z))	C40H76NO10P	760.5153277	No
12841	HMDB0112277	PS(14:1(9Z)/20:0)	C40H76NO10P	760.5153277	No
12841	HMDB0112512	PS(20:0/14:1(9Z))	C40H76NO10P	760.5153277	No
12841	HMDB0112540	PS(20:1(11Z)/14:0)	C40H76NO10P	760.5153277	No
12855	HMDB0007616	DG(22:0/24:1(15Z)/0:0)	C49H94O5	761.7019927	No
12855	HMDB0007644	DG(22:1(13Z)/24:0/0:0)	C49H94O5	761.7019927	No
12855	HMDB0007812	DG(24:0/22:1(13Z)/0:0)	C49H94O5	761.7019927	No
12855	HMDB0007840	DG(24:1(15Z)/22:0/0:0)	C49H94O5	761.7019927	No
12855	HMDB0042300	TG(14:0/14:1(9Z)/o-18:0)	C49H94O5	761.7019927	No
12855	HMDB0042938	TG(14:0/o-18:0/14:1(9Z))	C49H94O5	761.7019927	No
12855	HMDB0047746	TG(14:1(9Z)/14:0/o-18:0)	C49H94O5	761.7019927	No
12855	HMDB0056096	DG(22:0/0:0/24:1n9)	C49H94O5	761.7019927	No
12855	HMDB0056118	DG(24:0/0:0/22:1n9)	C49H94O5	761.7019927	No
12862	HMDB0061019	14-hydroxyclarithromycin	C38H69NO14	762.4637652	No
12906	HMDB0010666	PG(18:3(6Z,9Z,12Z)/18:3(6Z,9Z,12Z))	C42H71O10P	765.4719982	No
12906	HMDB0010667	PG(18:3(6Z,9Z,12Z)/18:3(9Z,12Z,15Z))	C42H71O10P	765.4719982	No
12906	HMDB0010681	PG(18:3(9Z,12Z,15Z)/18:3(6Z,9Z,12Z))	C42H71O10P	765.4719982	No
12906	HMDB0010682	PG(18:3(9Z,12Z,15Z)/18:3(9Z,12Z,15Z))	C42H71O10P	765.4719982	No
12909	HMDB0047902	TG(14:1(9Z)/14:1(9Z)/18:4(6Z,9Z,12Z,15Z))	C49H82O6	765.6024704	No
12909	HMDB0048276	TG(14:1(9Z)/18:4(6Z,9Z,12Z,15Z)/14:1(9Z))	C49H82O6	765.6024704	No
12910	HMDB0031939	Arachisprenol 11	C55H90O	765.6923837	No
12927	HMDB0010595	PG(16:1(9Z)/20:4(5Z,8Z,11Z,14Z))	C42H73O10P	767.4844496	No
12927	HMDB0010651	PG(18:2(9Z,12Z)/18:3(6Z,9Z,12Z))	C42H73O10P	767.4844496	No
12927	HMDB0010652	PG(18:2(9Z,12Z)/18:3(9Z,12Z,15Z))	C42H73O10P	767.4844496	No
12927	HMDB0010665	PG(18:3(6Z,9Z,12Z)/18:2(9Z,12Z))	C42H73O10P	767.4844496	No
12927	HMDB0010680	PG(18:3(9Z,12Z,15Z)/18:2(9Z,12Z))	C42H73O10P	767.4844496	No
12927	HMDB0116608	PG(20:4(5Z,8Z,11Z,14Z)/16:1(9Z))	C42H73O10P	767.4844496	No
12940	HMDB0007947; C00157	PC(15:0/20:3(5Z,8Z,11Z))	C43H80NO8P	768.5565072	No
12940	HMDB0007948; C00157	PC(15:0/20:3(8Z,11Z,14Z))	C43H80NO8P	768.5565072	No
12940	HMDB0008363; C00157	PC(20:3(5Z,8Z,11Z)/15:0)	C43H80NO8P	768.5565072	No
12940	HMDB0008396; C00157	PC(20:3(8Z,11Z,14Z)/15:0)	C43H80NO8P	768.5565072	No
12940	HMDB0008975; C00350	PE(16:1(9Z)/22:2(13Z,16Z))	C43H80NO8P	768.5565072	No
12940	HMDB0009001; C00350	PE(18:0/20:3(5Z,8Z,11Z))	C43H80NO8P	768.5565072	No
12940	HMDB0009002; C00350	PE(18:0/20:3(8Z,11Z,14Z))	C43H80NO8P	768.5565072	No
12940	HMDB0009033; C00350	PE(18:1(11Z)/20:2(11Z,14Z))	C43H80NO8P	768.5565072	No
12940	HMDB0009066; C00350	PE(18:1(9Z)/20:2(11Z,14Z))	C43H80NO8P	768.5565072	No
12940	HMDB0009098; C00350	PE(18:2(9Z,12Z)/20:1(11Z))	C43H80NO8P	768.5565072	No
12940	HMDB0009130; C00350	PE(18:3(6Z,9Z,12Z)/20:0)	C43H80NO8P	768.5565072	No
12940	HMDB0009163; C00350	PE(18:3(9Z,12Z,15Z)/20:0)	C43H80NO8P	768.5565072	No
12940	HMDB0009226; C00350	PE(20:0/18:3(6Z,9Z,12Z))	C43H80NO8P	768.5565072	No
12940	HMDB0009227; C00350	PE(20:0/18:3(9Z,12Z,15Z))	C43H80NO8P	768.5565072	No
12940	HMDB0009258; C00350	PE(20:1(11Z)/18:2(9Z,12Z))	C43H80NO8P	768.5565072	No
12940	HMDB0009289; C00350	PE(20:2(11Z,14Z)/18:1(11Z))	C43H80NO8P	768.5565072	No
12940	HMDB0009290; C00350	PE(20:2(11Z,14Z)/18:1(9Z))	C43H80NO8P	768.5565072	No
12940	HMDB0009321; C00350	PE(20:3(5Z,8Z,11Z)/18:0)	C43H80NO8P	768.5565072	No
12940	HMDB0009354; C00350	PE(20:3(8Z,11Z,14Z)/18:0)	C43H80NO8P	768.5565072	No
12940	HMDB0009551; C00350	PE(22:2(13Z,16Z)/16:1(9Z))	C43H80NO8P	768.5565072	No
12940	HMDB0113911	PE-NMe2(14:1(9Z)/22:2(13Z,16Z))	C43H80NO8P	768.5565072	No
12940	HMDB0113949	PE-NMe2(16:0/20:3(5Z,8Z,11Z))	C43H80NO8P	768.5565072	No
12940	HMDB0113950	PE-NMe2(16:0/20:3(8Z,11Z,14Z))	C43H80NO8P	768.5565072	No
12940	HMDB0113970	PE-NMe2(16:1(9Z)/20:2(11Z,14Z))	C43H80NO8P	768.5565072	No
12940	HMDB0113991	PE-NMe2(18:0/18:3(6Z,9Z,12Z))	C43H80NO8P	768.5565072	No
12940	HMDB0113992	PE-NMe2(18:0/18:3(9Z,12Z,15Z))	C43H80NO8P	768.5565072	No

12940	HMDB0114017	PE-NMe2(18:1(11Z)/18:2(9Z,12Z))	C43H80NO8P	768.5565072	No
12940	HMDB0114044	PE-NMe2(18:1(9Z)/18:2(9Z,12Z))	C43H80NO8P	768.5565072	No
12940	HMDB0114071	PE-NMe2(18:2(9Z,12Z)/18:1(11Z))	C43H80NO8P	768.5565072	No
12940	HMDB0114072	PE-NMe2(18:2(9Z,12Z)/18:1(9Z))	C43H80NO8P	768.5565072	No
12940	HMDB0114099	PE-NMe2(18:3(6Z,9Z,12Z)/18:0)	C43H80NO8P	768.5565072	No
12940	HMDB0114128	PE-NMe2(18:3(9Z,12Z,15Z)/18:0)	C43H80NO8P	768.5565072	No
12940	HMDB0114243	PE-NMe2(20:2(11Z,14Z)/16:1(9Z))	C43H80NO8P	768.5565072	No
12940	HMDB0114271	PE-NMe2(20:3(5Z,8Z,11Z)/16:0)	C43H80NO8P	768.5565072	No
12940	HMDB0114299	PE-NMe2(20:3(8Z,11Z,14Z)/16:0)	C43H80NO8P	768.5565072	No
12940	HMDB0114467	PE-NMe2(22:2(13Z,16Z)/14:1(9Z))	C43H80NO8P	768.5565072	No
12969	HMDB0032008	Kaempferol 3-sophorotrioside	C33H40O21	771.2010566	No
12969	HMDB0037207	Kaempferol 3-sophoroside 7-glucoside	C33H40O21	771.2010566	No
12969	HMDB0037936	Quercetin 3-(2G-rhamnosylgentiobioside)	C33H40O21	771.2010566	No
12969	HMDB0037957	Quercetin 3-rutinoside 7-galactoside	C33H40O21	771.2010566	No
12969	HMDB0037960	Quercetin 3-(2G-glucosylrutinoside)	C33H40O21	771.2010566	No
12969	HMDB0039363	Quercetin 3-(3R-glucosylrutinoside)	C33H40O21	771.2010566	No
12969	HMDB0039375	Hyperin 6''''-[glucosyl-(1->3)-rhamnoside]	C33H40O21	771.2010566	No
12969	HMDB0041771	Quercetin 3-O-glucosyl-rutinoside	C33H40O21	771.2010566	No
12969	HMDB0126861	3,4,5-trihydroxy-6-{[5-hydroxy-2-(4-hydroxyphenyl)-4-	C33H40O21	771.2010566	No
12969	HMDB0126862	6-(4-{5,7-dihydroxy-4-oxo-6,8-bis[3,4,5-trihydroxy-6-(h	C33H40O21	771.2010566	No
12969	HMDB0126863	3,4,5-trihydroxy-6-{[7-hydroxy-2-(4-hydroxyphenyl)-4-	C33H40O21	771.2010566	No
12969	HMDB0135148	3-[(3,4-dihydroxy-6-methyl-5-{[3,4,5-trihydroxy-6-(hyd	C33H40O21	771.2010566	No
12969	HMDB0136152	7-{[3,4-dihydroxy-6-(hydroxymethyl)-5-{[3,4,5-trihydro	C33H40O21	771.2010566	No
12989	HMDB0116515	PGP(a-13:0/a-17:0)	C36H72O13P2	773.4369424	No
12989	HMDB0116523	PGP(a-13:0/i-17:0)	C36H72O13P2	773.4369424	No
12989	HMDB0116542	PGP(i-12:0/i-18:0)	C36H72O13P2	773.4369424	No
12989	HMDB0116551	PGP(i-13:0/a-17:0)	C36H72O13P2	773.4369424	No
12989	HMDB0116559	PGP(i-13:0/i-17:0)	C36H72O13P2	773.4369424	No
12989	HMDB0116573	PGP(i-14:0/i-16:0)	C36H72O13P2	773.4369424	No
13005	HMDB0007925; C00157	PC(14:1(9Z)/22:6(4Z,7Z,10Z,13Z,16Z,19Z))	C44H74NO8P	774.5065271	No
13005	HMDB0008174; C00157	PC(18:3(6Z,9Z,12Z)/18:4(6Z,9Z,12Z,15Z))	C44H74NO8P	774.5065271	No
13005	HMDB0008207; C00157	PC(18:3(9Z,12Z,15Z)/18:4(6Z,9Z,12Z,15Z))	C44H74NO8P	774.5065271	No
13005	HMDB0008238; C00157	PC(18:4(6Z,9Z,12Z,15Z)/18:3(6Z,9Z,12Z))	C44H74NO8P	774.5065271	No
13005	HMDB0008239; C00157	PC(18:4(6Z,9Z,12Z,15Z)/18:3(9Z,12Z,15Z))	C44H74NO8P	774.5065271	No
13005	HMDB0008723; C00157	PC(22:6(4Z,7Z,10Z,13Z,16Z,19Z)/14:1(9Z))	C44H74NO8P	774.5065271	No
13005	HMDB0113081	PE-NMe(16:1(9Z)/22:6(4Z,7Z,10Z,13Z,16Z,19Z))	C44H74NO8P	774.5065271	No
13005	HMDB0113183	PE-NMe(18:2(9Z,12Z)/20:5(5Z,8Z,11Z,14Z,17Z))	C44H74NO8P	774.5065271	No
13005	HMDB0113209	PE-NMe(18:3(6Z,9Z,12Z)/20:4(5Z,8Z,11Z,14Z))	C44H74NO8P	774.5065271	No
13005	HMDB0113210	PE-NMe(18:3(6Z,9Z,12Z)/20:4(8Z,11Z,14Z,17Z))	C44H74NO8P	774.5065271	No
13005	HMDB0113238	PE-NMe(18:3(9Z,12Z,15Z)/20:4(5Z,8Z,11Z,14Z))	C44H74NO8P	774.5065271	No
13005	HMDB0113239	PE-NMe(18:3(9Z,12Z,15Z)/20:4(8Z,11Z,14Z,17Z))	C44H74NO8P	774.5065271	No
13005	HMDB0113265	PE-NMe(18:4(6Z,9Z,12Z,15Z)/20:3(5Z,8Z,11Z))	C44H74NO8P	774.5065271	No
13005	HMDB0113266	PE-NMe(18:4(6Z,9Z,12Z,15Z)/20:3(8Z,11Z,14Z))	C44H74NO8P	774.5065271	No
13005	HMDB0113377	PE-NMe(20:3(5Z,8Z,11Z)/18:4(6Z,9Z,12Z,15Z))	C44H74NO8P	774.5065271	No
13005	HMDB0113405	PE-NMe(20:3(8Z,11Z,14Z)/18:4(6Z,9Z,12Z,15Z))	C44H74NO8P	774.5065271	No
13005	HMDB0113431	PE-NMe(20:4(5Z,8Z,11Z,14Z)/18:3(6Z,9Z,12Z))	C44H74NO8P	774.5065271	No
13005	HMDB0113432	PE-NMe(20:4(5Z,8Z,11Z,14Z)/18:3(9Z,12Z,15Z))	C44H74NO8P	774.5065271	No
13005	HMDB0113459	PE-NMe(20:4(8Z,11Z,14Z,17Z)/18:3(6Z,9Z,12Z))	C44H74NO8P	774.5065271	No
13005	HMDB0113460	PE-NMe(20:4(8Z,11Z,14Z,17Z)/18:3(9Z,12Z,15Z))	C44H74NO8P	774.5065271	No
13005	HMDB0113487	PE-NMe(20:5(5Z,8Z,11Z,14Z,17Z)/18:2(9Z,12Z))	C44H74NO8P	774.5065271	No
13005	HMDB0113677	PE-NMe(22:6(4Z,7Z,10Z,13Z,16Z,19Z)/16:1(9Z))	C44H74NO8P	774.5065271	No
13097	HMDB0040398	1alpha,3beta,22R-Trihydroxyergosta-5,24E-dien-26-oic	C40H64O15	783.4153318	No
13146	HMDB0039118	Delphinidin 3-sophoroside 5-glucoside	C33H41O22	788.2011587	No
13151	HMDB0006796; C06198; CHEBI:27791	P1,P4-Bis(5''-uridyl) tetraphosphate	C18H26N4O23P4	788.9881032	No
13160	HMDB0042100	TG(14:0/15:0/18:1(11Z))	C50H94O6	789.6973161	No
13160	HMDB0042101	TG(14:0/15:0/18:1(9Z))	C50H94O6	789.6973161	No
13160	HMDB0042332	TG(14:0/18:1(11Z)/15:0)	C50H94O6	789.6973161	No
13160	HMDB0042362	TG(14:0/18:1(9Z)/15:0)	C50H94O6	789.6973161	No
13160	HMDB0042968	TG(15:0/14:0/18:1(11Z))	C50H94O6	789.6973161	No
13160	HMDB0042969	TG(15:0/14:0/18:1(9Z))	C50H94O6	789.6973161	No
13160	HMDB0043025	TG(15:0/16:0/16:1(9Z))	C50H94O6	789.6973161	No

13160	HMDB0043053	TG(15:0/18:0/14:1(9Z))	C50H94O6	789.6973161	No
13160	HMDB0043165	TG(15:0/14:1(9Z)/18:0)	C50H94O6	789.6973161	No
13160	HMDB0043193	TG(15:0/16:1(9Z)/16:0)	C50H94O6	789.6973161	No
13160	HMDB0043863	TG(16:0/15:0/16:1(9Z))	C50H94O6	789.6973161	No
13160	HMDB0044699	TG(18:0/15:0/14:1(9Z))	C50H94O6	789.6973161	No
13170	HMDB0007958; C00157	PC(15:0/22:6(4Z,7Z,10Z,13Z,16Z,19Z))	C45H78NO8P	790.5373548	No
13170	HMDB0008724; C00157	PC(22:6(4Z,7Z,10Z,13Z,16Z,19Z)/15:0)	C45H78NO8P	790.5373548	No
13170	HMDB0009012; C00350	PE(18:0/22:6(4Z,7Z,10Z,13Z,16Z,19Z))	C45H78NO8P	790.5373548	No
13170	HMDB0009043; C00350	PE(18:1(11Z)/22:5(4Z,7Z,10Z,13Z,16Z))	C45H78NO8P	790.5373548	No
13170	HMDB0009044; C00350	PE(18:1(11Z)/22:5(7Z,10Z,13Z,16Z,19Z))	C45H78NO8P	790.5373548	No
13170	HMDB0009076; C00350	PE(18:1(9Z)/22:5(4Z,7Z,10Z,13Z,16Z))	C45H78NO8P	790.5373548	No
13170	HMDB0009077; C00350	PE(18:1(9Z)/22:5(7Z,10Z,13Z,16Z,19Z))	C45H78NO8P	790.5373548	No
13170	HMDB0009108; C00350	PE(18:2(9Z,12Z)/22:4(7Z,10Z,13Z,16Z))	C45H78NO8P	790.5373548	No
13170	HMDB0009206; C00350	PE(18:4(6Z,9Z,12Z,15Z)/22:2(13Z,16Z))	C45H78NO8P	790.5373548	No
13170	HMDB0009269; C00350	PE(20:1(11Z)/20:5(5Z,8Z,11Z,14Z,17Z))	C45H78NO8P	790.5373548	No
13170	HMDB0009300; C00350	PE(20:2(11Z,14Z)/20:4(5Z,8Z,11Z,14Z))	C45H78NO8P	790.5373548	No
13170	HMDB0009301; C00350	PE(20:2(11Z,14Z)/20:4(8Z,11Z,14Z,17Z))	C45H78NO8P	790.5373548	No
13170	HMDB0009331; C00350	PE(20:3(5Z,8Z,11Z)/20:3(5Z,8Z,11Z))	C45H78NO8P	790.5373548	No
13170	HMDB0009332; C00350	PE(20:3(5Z,8Z,11Z)/20:3(8Z,11Z,14Z))	C45H78NO8P	790.5373548	No
13170	HMDB0009364; C00350	PE(20:3(8Z,11Z,14Z)/20:3(5Z,8Z,11Z))	C45H78NO8P	790.5373548	No
13170	HMDB0009365; C00350	PE(20:3(8Z,11Z,14Z)/20:3(8Z,11Z,14Z))	C45H78NO8P	790.5373548	No
13170	HMDB0009396; C00350	PE(20:4(5Z,8Z,11Z,14Z)/20:2(11Z,14Z))	C45H78NO8P	790.5373548	No
13170	HMDB0009429; C00350	PE(20:4(8Z,11Z,14Z,17Z)/20:2(11Z,14Z))	C45H78NO8P	790.5373548	No
13170	HMDB0009461; C00350	PE(20:5(5Z,8Z,11Z,14Z,17Z)/20:1(11Z))	C45H78NO8P	790.5373548	No
13170	HMDB0009558; C00350	PE(22:2(13Z,16Z)/18:4(6Z,9Z,12Z,15Z))	C45H78NO8P	790.5373548	No
13170	HMDB0009588; C00350	PE(22:4(7Z,10Z,13Z,16Z)/18:2(9Z,12Z))	C45H78NO8P	790.5373548	No
13170	HMDB0009619; C00350	PE(22:5(4Z,7Z,10Z,13Z,16Z)/18:1(11Z))	C45H78NO8P	790.5373548	No
13170	HMDB0009620; C00350	PE(22:5(4Z,7Z,10Z,13Z,16Z)/18:1(9Z))	C45H78NO8P	790.5373548	No
13170	HMDB0009652; C00350	PE(22:5(7Z,10Z,13Z,16Z,19Z)/18:1(11Z))	C45H78NO8P	790.5373548	No
13170	HMDB0009653; C00350	PE(22:5(7Z,10Z,13Z,16Z,19Z)/18:1(9Z))	C45H78NO8P	790.5373548	No
13170	HMDB0009684; C00350	PE(22:6(4Z,7Z,10Z,13Z,16Z,19Z)/18:0)	C45H78NO8P	790.5373548	No
13170	HMDB0113959	PE-NMe2(16:0/22:6(4Z,7Z,10Z,13Z,16Z,19Z))	C45H78NO8P	790.5373548	No
13170	HMDB0113980	PE-NMe2(16:1(9Z)/22:5(4Z,7Z,10Z,13Z,16Z))	C45H78NO8P	790.5373548	No
13170	HMDB0113981	PE-NMe2(16:1(9Z)/22:5(7Z,10Z,13Z,16Z,19Z))	C45H78NO8P	790.5373548	No
13170	HMDB0114028	PE-NMe2(18:1(11Z)/20:5(5Z,8Z,11Z,14Z,17Z))	C45H78NO8P	790.5373548	No
13170	HMDB0114055	PE-NMe2(18:1(9Z)/20:5(5Z,8Z,11Z,14Z,17Z))	C45H78NO8P	790.5373548	No
13170	HMDB0114082	PE-NMe2(18:2(9Z,12Z)/20:4(5Z,8Z,11Z,14Z))	C45H78NO8P	790.5373548	No
13170	HMDB0114083	PE-NMe2(18:2(9Z,12Z)/20:4(8Z,11Z,14Z,17Z))	C45H78NO8P	790.5373548	No
13170	HMDB0114109	PE-NMe2(18:3(6Z,9Z,12Z)/20:3(5Z,8Z,11Z))	C45H78NO8P	790.5373548	No
13170	HMDB0114110	PE-NMe2(18:3(6Z,9Z,12Z)/20:3(8Z,11Z,14Z))	C45H78NO8P	790.5373548	No
13170	HMDB0114138	PE-NMe2(18:3(9Z,12Z,15Z)/20:3(5Z,8Z,11Z))	C45H78NO8P	790.5373548	No
13170	HMDB0114139	PE-NMe2(18:3(9Z,12Z,15Z)/20:3(8Z,11Z,14Z))	C45H78NO8P	790.5373548	No
13170	HMDB0114166	PE-NMe2(18:4(6Z,9Z,12Z,15Z)/20:2(11Z,14Z))	C45H78NO8P	790.5373548	No
13170	HMDB0114250	PE-NMe2(20:2(11Z,14Z)/18:4(6Z,9Z,12Z,15Z))	C45H78NO8P	790.5373548	No
13170	HMDB0114277	PE-NMe2(20:3(5Z,8Z,11Z)/18:3(6Z,9Z,12Z))	C45H78NO8P	790.5373548	No
13170	HMDB0114278	PE-NMe2(20:3(5Z,8Z,11Z)/18:3(9Z,12Z,15Z))	C45H78NO8P	790.5373548	No
13170	HMDB0114305	PE-NMe2(20:3(8Z,11Z,14Z)/18:3(6Z,9Z,12Z))	C45H78NO8P	790.5373548	No
13170	HMDB0114306	PE-NMe2(20:3(8Z,11Z,14Z)/18:3(9Z,12Z,15Z))	C45H78NO8P	790.5373548	No
13170	HMDB0114332	PE-NMe2(20:4(5Z,8Z,11Z,14Z)/18:2(9Z,12Z))	C45H78NO8P	790.5373548	No
13170	HMDB0114360	PE-NMe2(20:4(8Z,11Z,14Z,17Z)/18:2(9Z,12Z))	C45H78NO8P	790.5373548	No
13170	HMDB0114387	PE-NMe2(20:5(5Z,8Z,11Z,14Z,17Z)/18:1(11Z))	C45H78NO8P	790.5373548	No
13170	HMDB0114388	PE-NMe2(20:5(5Z,8Z,11Z,14Z,17Z)/18:1(9Z))	C45H78NO8P	790.5373548	No
13170	HMDB0114526	PE-NMe2(22:5(4Z,7Z,10Z,13Z,16Z)/16:1(9Z))	C45H78NO8P	790.5373548	No
13170	HMDB0114553	PE-NMe2(22:5(7Z,10Z,13Z,16Z,19Z)/16:1(9Z))	C45H78NO8P	790.5373548	No
13170	HMDB0114579	PE-NMe2(22:6(4Z,7Z,10Z,13Z,16Z,19Z)/16:0)	C45H78NO8P	790.5373548	No
13204	HMDB0007955; C00157	PC(15:0/22:4(7Z,10Z,13Z,16Z))	C45H82NO8P	794.5677637	No
13204	HMDB0008625; C00157	PC(22:4(7Z,10Z,13Z,16Z)/15:0)	C45H82NO8P	794.5677637	No
13204	HMDB0009009; C00350	PE(18:0/22:4(7Z,10Z,13Z,16Z))	C45H82NO8P	794.5677637	No
13204	HMDB0009107; C00350	PE(18:2(9Z,12Z)/22:2(13Z,16Z))	C45H82NO8P	794.5677637	No
13204	HMDB0009139; C00350	PE(18:3(6Z,9Z,12Z)/22:1(13Z))	C45H82NO8P	794.5677637	No
13204	HMDB0009172; C00350	PE(18:3(9Z,12Z,15Z)/22:1(13Z))	C45H82NO8P	794.5677637	No

13204	HMDB0009204; C00350	PE(18:4(6Z,9Z,12Z,15Z)/22:0)	C45H82NO8P	794.5677637	No
13204	HMDB0009234; C00350	PE(20:0/20:4(5Z,8Z,11Z,14Z))	C45H82NO8P	794.5677637	No
13204	HMDB0009235; C00350	PE(20:0/20:4(8Z,11Z,14Z,17Z))	C45H82NO8P	794.5677637	No
13204	HMDB0009265; C00350	PE(20:1(11Z)/20:3(5Z,8Z,11Z))	C45H82NO8P	794.5677637	No
13204	HMDB0009266; C00350	PE(20:1(11Z)/20:3(8Z,11Z,14Z))	C45H82NO8P	794.5677637	No
13204	HMDB0009297; C00350	PE(20:2(11Z,14Z)/20:2(11Z,14Z))	C45H82NO8P	794.5677637	No
13204	HMDB0009329; C00350	PE(20:3(5Z,8Z,11Z)/20:1(11Z))	C45H82NO8P	794.5677637	No
13204	HMDB0009362; C00350	PE(20:3(8Z,11Z,14Z)/20:1(11Z))	C45H82NO8P	794.5677637	No
13204	HMDB0009394; C00350	PE(20:4(5Z,8Z,11Z,14Z)/20:0)	C45H82NO8P	794.5677637	No
13204	HMDB0009427; C00350	PE(20:4(8Z,11Z,14Z,17Z)/20:0)	C45H82NO8P	794.5677637	No
13204	HMDB0009492; C00350	PE(22:0/18:4(6Z,9Z,12Z,15Z))	C45H82NO8P	794.5677637	No
13204	HMDB0009523; C00350	PE(22:1(13Z)/18:3(6Z,9Z,12Z))	C45H82NO8P	794.5677637	No
13204	HMDB0009524; C00350	PE(22:1(13Z)/18:3(9Z,12Z,15Z))	C45H82NO8P	794.5677637	No
13204	HMDB0009555; C00350	PE(22:2(13Z,16Z)/18:2(9Z,12Z))	C45H82NO8P	794.5677637	No
13204	HMDB0009585; C00350	PE(22:4(7Z,10Z,13Z,16Z)/18:0)	C45H82NO8P	794.5677637	No
13204	HMDB0113956	PE-NMe2(16:0/22:4(7Z,10Z,13Z,16Z))	C45H82NO8P	794.5677637	No
13204	HMDB0113999	PE-NMe2(18:0/20:4(5Z,8Z,11Z,14Z))	C45H82NO8P	794.5677637	No
13204	HMDB0114000	PE-NMe2(18:0/20:4(8Z,11Z,14Z,17Z))	C45H82NO8P	794.5677637	No
13204	HMDB0114024	PE-NMe2(18:1(11Z)/20:3(5Z,8Z,11Z))	C45H82NO8P	794.5677637	No
13204	HMDB0114025	PE-NMe2(18:1(11Z)/20:3(8Z,11Z,14Z))	C45H82NO8P	794.5677637	No
13204	HMDB0114051	PE-NMe2(18:1(9Z)/20:3(5Z,8Z,11Z))	C45H82NO8P	794.5677637	No
13204	HMDB0114052	PE-NMe2(18:1(9Z)/20:3(8Z,11Z,14Z))	C45H82NO8P	794.5677637	No
13204	HMDB0114079	PE-NMe2(18:2(9Z,12Z)/20:2(11Z,14Z))	C45H82NO8P	794.5677637	No
13204	HMDB0114107	PE-NMe2(18:3(6Z,9Z,12Z)/20:1(11Z))	C45H82NO8P	794.5677637	No
13204	HMDB0114136	PE-NMe2(18:3(9Z,12Z,15Z)/20:1(11Z))	C45H82NO8P	794.5677637	No
13204	HMDB0114164	PE-NMe2(18:4(6Z,9Z,12Z,15Z)/20:0)	C45H82NO8P	794.5677637	No
13204	HMDB0114192	PE-NMe2(20:0/18:4(6Z,9Z,12Z,15Z))	C45H82NO8P	794.5677637	No
13204	HMDB0114219	PE-NMe2(20:1(11Z)/18:3(6Z,9Z,12Z))	C45H82NO8P	794.5677637	No
13204	HMDB0114220	PE-NMe2(20:1(11Z)/18:3(9Z,12Z,15Z))	C45H82NO8P	794.5677637	No
13204	HMDB0114247	PE-NMe2(20:2(11Z,14Z)/18:2(9Z,12Z))	C45H82NO8P	794.5677637	No
13204	HMDB0114274	PE-NMe2(20:3(5Z,8Z,11Z)/18:1(11Z))	C45H82NO8P	794.5677637	No
13204	HMDB0114275	PE-NMe2(20:3(5Z,8Z,11Z)/18:1(9Z))	C45H82NO8P	794.5677637	No
13204	HMDB0114302	PE-NMe2(20:3(8Z,11Z,14Z)/18:1(11Z))	C45H82NO8P	794.5677637	No
13204	HMDB0114303	PE-NMe2(20:3(8Z,11Z,14Z)/18:1(9Z))	C45H82NO8P	794.5677637	No
13204	HMDB0114329	PE-NMe2(20:4(5Z,8Z,11Z,14Z)/18:0)	C45H82NO8P	794.5677637	No
13204	HMDB0114357	PE-NMe2(20:4(8Z,11Z,14Z,17Z)/18:0)	C45H82NO8P	794.5677637	No
13204	HMDB0114498	PE-NMe2(22:4(7Z,10Z,13Z,16Z)/16:0)	C45H82NO8P	794.5677637	No
13218	HMDB0030465	Mocimycin	C43H60N2O12	795.4068645	No
13219	HMDB0010582	PG(16:0/22:5(4Z,7Z,10Z,13Z,16Z))	C44H77O10P	795.5156612	No
13219	HMDB0010583	PG(16:0/22:5(7Z,10Z,13Z,16Z,19Z))	C44H77O10P	795.5156612	No
13219	HMDB0010596	PG(16:1(9Z)/22:4(7Z,10Z,13Z,16Z))	C44H77O10P	795.5156612	No
13219	HMDB0010625	PG(18:1(11Z)/20:4(5Z,8Z,11Z,14Z))	C44H77O10P	795.5156612	No
13219	HMDB0010640	PG(18:1(9Z)/20:4(5Z,8Z,11Z,14Z))	C44H77O10P	795.5156612	No
13219	HMDB0010653	PG(18:2(9Z,12Z)/20:3(5Z,8Z,11Z))	C44H77O10P	795.5156612	No
13219	HMDB0010654	PG(18:2(9Z,12Z)/20:3(8Z,11Z,14Z))	C44H77O10P	795.5156612	No
13219	HMDB0116578	PG(20:3(5Z,8Z,11Z)/18:2(9Z,12Z))	C44H77O10P	795.5156612	No
13219	HMDB0116581	PG(20:3(8Z,11Z,14Z)/18:2(9Z,12Z))	C44H77O10P	795.5156612	No
13219	HMDB0116609	PG(20:4(5Z,8Z,11Z,14Z)/18:1(11Z))	C44H77O10P	795.5156612	No
13219	HMDB0116610	PG(20:4(5Z,8Z,11Z,14Z)/18:1(9Z))	C44H77O10P	795.5156612	No
13219	HMDB0116621	PG(22:5(7Z,10Z,13Z,16Z,19Z)/16:0)	C44H77O10P	795.5156612	No
13219	HMDB0116743	PG(22:5(4Z,7Z,10Z,13Z,16Z)/16:0)	C44H77O10P	795.5156612	No
13237	HMDB0115344	PA(22:4(7Z,10Z,13Z,16Z)/22:5(4Z,7Z,10Z,13Z,16Z))	C47H75O8P	797.5130446	No
13237	HMDB0115345	PA(22:4(7Z,10Z,13Z,16Z)/22:5(7Z,10Z,13Z,16Z,19Z))	C47H75O8P	797.5130446	No
13237	HMDB0115370	PA(22:5(4Z,7Z,10Z,13Z,16Z)/22:4(7Z,10Z,13Z,16Z))	C47H75O8P	797.5130446	No
13237	HMDB0115395	PA(22:5(7Z,10Z,13Z,16Z,19Z)/22:4(7Z,10Z,13Z,16Z))	C47H75O8P	797.5130446	No
13249	HMDB0008248; C00157	PC(18:4(6Z,9Z,12Z,15Z)/20:5(5Z,8Z,11Z,14Z,17Z))	C46H74NO8P	798.5096334	No
13249	HMDB0008503; C00157	PC(20:5(5Z,8Z,11Z,14Z,17Z)/18:4(6Z,9Z,12Z,15Z))	C46H74NO8P	798.5096334	No
13249	HMDB0113218	PE-NMe(18:3(6Z,9Z,12Z)/22:6(4Z,7Z,10Z,13Z,16Z,19Z)	C46H74NO8P	798.5096334	No
13249	HMDB0113247	PE-NMe(18:3(9Z,12Z,15Z)/22:6(4Z,7Z,10Z,13Z,16Z,19Z	C46H74NO8P	798.5096334	No
13249	HMDB0113274	PE-NMe(18:4(6Z,9Z,12Z,15Z)/22:5(4Z,7Z,10Z,13Z,16Z)	C46H74NO8P	798.5096334	No
13249	HMDB0113275	PE-NMe(18:4(6Z,9Z,12Z,15Z)/22:5(7Z,10Z,13Z,16Z,19Z	C46H74NO8P	798.5096334	No

13249	HMDB0113440	PE-NMe(20:4(5Z,8Z,11Z,14Z)/20:5(5Z,8Z,11Z,14Z,17Z)	C46H74NO8P	798.5096334	No
13249	HMDB0113469	PE-NMe(20:4(8Z,11Z,14Z,17Z)/20:5(5Z,8Z,11Z,14Z,17Z	C46H74NO8P	798.5096334	No
13249	HMDB0113496	PE-NMe(20:5(5Z,8Z,11Z,14Z,17Z)/20:4(5Z,8Z,11Z,14Z)	C46H74NO8P	798.5096334	No
13249	HMDB0113497	PE-NMe(20:5(5Z,8Z,11Z,14Z,17Z)/20:4(8Z,11Z,14Z,17Z	C46H74NO8P	798.5096334	No
13249	HMDB0113630	PE-NMe(22:5(4Z,7Z,10Z,13Z,16Z)/18:4(6Z,9Z,12Z,15Z)	C46H74NO8P	798.5096334	No
13249	HMDB0113657	PE-NMe(22:5(7Z,10Z,13Z,16Z,19Z)/18:4(6Z,9Z,12Z,15Z	C46H74NO8P	798.5096334	No
13249	HMDB0113682	PE-NMe(22:6(4Z,7Z,10Z,13Z,16Z,19Z)/18:3(6Z,9Z,12Z)	C46H74NO8P	798.5096334	No
13249	HMDB0113683	PE-NMe(22:6(4Z,7Z,10Z,13Z,16Z,19Z)/18:3(9Z,12Z,15Z	C46H74NO8P	798.5096334	No
13265	HMDB0115320	PA(22:2(13Z,16Z)/22:6(4Z,7Z,10Z,13Z,16Z,19Z))	C47H77O8P	799.5287507	No
13265	HMDB0115343	PA(22:4(7Z,10Z,13Z,16Z)/22:4(7Z,10Z,13Z,16Z))	C47H77O8P	799.5287507	No
13265	HMDB0115421	PA(22:6(4Z,7Z,10Z,13Z,16Z,19Z)/22:2(13Z,16Z))	C47H77O8P	799.5287507	No
13278	HMDB0008182; C00157	PC(18:3(6Z,9Z,12Z)/20:5(5Z,8Z,11Z,14Z,17Z))	C46H76NO8P	800.5221798	No
13278	HMDB0008215; C00157	PC(18:3(9Z,12Z,15Z)/20:5(5Z,8Z,11Z,14Z,17Z))	C46H76NO8P	800.5221798	No
13278	HMDB0008246; C00157	PC(18:4(6Z,9Z,12Z,15Z)/20:4(5Z,8Z,11Z,14Z))	C46H76NO8P	800.5221798	No
13278	HMDB0008247; C00157	PC(18:4(6Z,9Z,12Z,15Z)/20:4(8Z,11Z,14Z,17Z))	C46H76NO8P	800.5221798	No
13278	HMDB0008437; C00157	PC(20:4(5Z,8Z,11Z,14Z)/18:4(6Z,9Z,12Z,15Z))	C46H76NO8P	800.5221798	No
13278	HMDB0008470; C00157	PC(20:4(8Z,11Z,14Z,17Z)/18:4(6Z,9Z,12Z,15Z))	C46H76NO8P	800.5221798	No
13278	HMDB0008501; C00157	PC(20:5(5Z,8Z,11Z,14Z,17Z)/18:3(6Z,9Z,12Z))	C46H76NO8P	800.5221798	No
13278	HMDB0008502; C00157	PC(20:5(5Z,8Z,11Z,14Z,17Z)/18:3(9Z,12Z,15Z))	C46H76NO8P	800.5221798	No
13278	HMDB0112976	PE-NMe(20:4(5Z,8Z,11Z,14Z)/20:4(5Z,8Z,11Z,14Z))	C46H76NO8P	800.5221798	No
13278	HMDB0113190	PE-NMe(18:2(9Z,12Z)/22:6(4Z,7Z,10Z,13Z,16Z,19Z))	C46H76NO8P	800.5221798	No
13278	HMDB0113216	PE-NMe(18:3(6Z,9Z,12Z)/22:5(4Z,7Z,10Z,13Z,16Z))	C46H76NO8P	800.5221798	No
13278	HMDB0113217	PE-NMe(18:3(6Z,9Z,12Z)/22:5(7Z,10Z,13Z,16Z,19Z))	C46H76NO8P	800.5221798	No
13278	HMDB0113245	PE-NMe(18:3(9Z,12Z,15Z)/22:5(4Z,7Z,10Z,13Z,16Z))	C46H76NO8P	800.5221798	No
13278	HMDB0113246	PE-NMe(18:3(9Z,12Z,15Z)/22:5(7Z,10Z,13Z,16Z,19Z))	C46H76NO8P	800.5221798	No
13278	HMDB0113273	PE-NMe(18:4(6Z,9Z,12Z,15Z)/22:4(7Z,10Z,13Z,16Z))	C46H76NO8P	800.5221798	No
13278	HMDB0113385	PE-NMe(20:3(5Z,8Z,11Z)/20:5(5Z,8Z,11Z,14Z,17Z))	C46H76NO8P	800.5221798	No
13278	HMDB0113413	PE-NMe(20:3(8Z,11Z,14Z)/20:5(5Z,8Z,11Z,14Z,17Z))	C46H76NO8P	800.5221798	No
13278	HMDB0113439	PE-NMe(20:4(5Z,8Z,11Z,14Z)/20:4(8Z,11Z,14Z,17Z))	C46H76NO8P	800.5221798	No
13278	HMDB0113467	PE-NMe(20:4(8Z,11Z,14Z,17Z)/20:4(5Z,8Z,11Z,14Z))	C46H76NO8P	800.5221798	No
13278	HMDB0113468	PE-NMe(20:4(8Z,11Z,14Z,17Z)/20:4(8Z,11Z,14Z,17Z))	C46H76NO8P	800.5221798	No
13278	HMDB0113494	PE-NMe(20:5(5Z,8Z,11Z,14Z,17Z)/20:3(5Z,8Z,11Z))	C46H76NO8P	800.5221798	No
13278	HMDB0113495	PE-NMe(20:5(5Z,8Z,11Z,14Z,17Z)/20:3(8Z,11Z,14Z))	C46H76NO8P	800.5221798	No
13278	HMDB0113603	PE-NMe(22:4(7Z,10Z,13Z,16Z)/18:4(6Z,9Z,12Z,15Z))	C46H76NO8P	800.5221798	No
13278	HMDB0113628	PE-NMe(22:5(4Z,7Z,10Z,13Z,16Z)/18:3(6Z,9Z,12Z))	C46H76NO8P	800.5221798	No
13278	HMDB0113629	PE-NMe(22:5(4Z,7Z,10Z,13Z,16Z)/18:3(9Z,12Z,15Z))	C46H76NO8P	800.5221798	No
13278	HMDB0113655	PE-NMe(22:5(7Z,10Z,13Z,16Z,19Z)/18:3(6Z,9Z,12Z))	C46H76NO8P	800.5221798	No
13278	HMDB0113656	PE-NMe(22:5(7Z,10Z,13Z,16Z,19Z)/18:3(9Z,12Z,15Z))	C46H76NO8P	800.5221798	No
13278	HMDB0113681	PE-NMe(22:6(4Z,7Z,10Z,13Z,16Z,19Z)/18:2(9Z,12Z))	C46H76NO8P	800.5221798	No
13279	HMDB0112763	PS(22:2(13Z,16Z)/15:0)	C43H80NO10P	800.5429396	No
13279	HMDB0116749	PS(15:0/22:2(13Z,16Z))	C43H80NO10P	800.5429396	No
13280	HMDB0008292; C00157	PC(20:0/P-18:0)	C46H92NO7P	800.6512378	No
13280	HMDB0008554; C00157	PC(22:0/P-16:0)	C46H92NO7P	800.6512378	No
13280	HMDB0011223	PC(P-16:0/22:0)	C46H92NO7P	800.6512378	No
13280	HMDB0011248	PC(P-18:0/20:0)	C46H92NO7P	800.6512378	No
13280	HMDB0013416	PC(o-16:1(9Z)/22:0)	C46H92NO7P	800.6512378	No
13280	HMDB0013430	PC(o-18:1(9Z)/20:0)	C46H92NO7P	800.6512378	No
13287	HMDB0013472	PGP(16:0/16:0)	C38H76O13P2	801.4670897	No
13287	HMDB0116525	PGP(a-13:0/i-19:0)	C38H76O13P2	801.4670897	No
13287	HMDB0116544	PGP(i-12:0/i-20:0)	C38H76O13P2	801.4670897	No
13287	HMDB0116561	PGP(i-13:0/i-19:0)	C38H76O13P2	801.4670897	No
13289	HMDB0115293	PA(22:1(13Z)/22:6(4Z,7Z,10Z,13Z,16Z,19Z))	C47H79O8P	801.5465154	No
13289	HMDB0115318	PA(22:2(13Z,16Z)/22:5(4Z,7Z,10Z,13Z,16Z))	C47H79O8P	801.5465154	No
13289	HMDB0115319	PA(22:2(13Z,16Z)/22:5(7Z,10Z,13Z,16Z,19Z))	C47H79O8P	801.5465154	No
13289	HMDB0115369	PA(22:5(4Z,7Z,10Z,13Z,16Z)/22:2(13Z,16Z))	C47H79O8P	801.5465154	No
13289	HMDB0115394	PA(22:5(7Z,10Z,13Z,16Z,19Z)/22:2(13Z,16Z))	C47H79O8P	801.5465154	No
13289	HMDB0115420	PA(22:6(4Z,7Z,10Z,13Z,16Z,19Z)/22:1(13Z))	C47H79O8P	801.5465154	No
13307	HMDB0008023; C00157	PC(16:1(9Z)/22:6(4Z,7Z,10Z,13Z,16Z,19Z))	C46H78NO8P	802.537657	No
13307	HMDB0008149; C00157	PC(18:2(9Z,12Z)/20:5(5Z,8Z,11Z,14Z,17Z))	C46H78NO8P	802.537657	No
13307	HMDB0008180; C00157	PC(18:3(6Z,9Z,12Z)/20:4(5Z,8Z,11Z,14Z))	C46H78NO8P	802.537657	No
13307	HMDB0008181; C00157	PC(18:3(6Z,9Z,12Z)/20:4(8Z,11Z,14Z,17Z))	C46H78NO8P	802.537657	No

13307	HMDB0008213; C00157	PC(18:3(9Z,12Z,15Z)/20:4(5Z,8Z,11Z,14Z))	C46H78NO8P	802.537657	No
13307	HMDB0008214; C00157	PC(18:3(9Z,12Z,15Z)/20:4(8Z,11Z,14Z,17Z))	C46H78NO8P	802.537657	No
13307	HMDB0008244; C00157	PC(18:4(6Z,9Z,12Z,15Z)/20:3(5Z,8Z,11Z))	C46H78NO8P	802.537657	No
13307	HMDB0008245; C00157	PC(18:4(6Z,9Z,12Z,15Z)/20:3(8Z,11Z,14Z))	C46H78NO8P	802.537657	No
13307	HMDB0008372; C00157	PC(20:3(5Z,8Z,11Z)/18:4(6Z,9Z,12Z,15Z))	C46H78NO8P	802.537657	No
13307	HMDB0008405; C00157	PC(20:3(8Z,11Z,14Z)/18:4(6Z,9Z,12Z,15Z))	C46H78NO8P	802.537657	No
13307	HMDB0008435; C00157	PC(20:4(5Z,8Z,11Z,14Z)/18:3(6Z,9Z,12Z))	C46H78NO8P	802.537657	No
13307	HMDB0008436; C00157	PC(20:4(5Z,8Z,11Z,14Z)/18:3(9Z,12Z,15Z))	C46H78NO8P	802.537657	No
13307	HMDB0008468; C00157	PC(20:4(8Z,11Z,14Z,17Z)/18:3(6Z,9Z,12Z))	C46H78NO8P	802.537657	No
13307	HMDB0008469; C00157	PC(20:4(8Z,11Z,14Z,17Z)/18:3(9Z,12Z,15Z))	C46H78NO8P	802.537657	No
13307	HMDB0008500; C00157	PC(20:5(5Z,8Z,11Z,14Z,17Z)/18:2(9Z,12Z))	C46H78NO8P	802.537657	No
13307	HMDB0008726; C00157	PC(22:6(4Z,7Z,10Z,13Z,16Z,19Z)/16:1(9Z))	C46H78NO8P	802.537657	No
13307	HMDB0113134	PE-NMe(18:1(11Z)/22:6(4Z,7Z,10Z,13Z,16Z,19Z))	C46H78NO8P	802.537657	No
13307	HMDB0113161	PE-NMe(18:1(9Z)/22:6(4Z,7Z,10Z,13Z,16Z,19Z))	C46H78NO8P	802.537657	No
13307	HMDB0113188	PE-NMe(18:2(9Z,12Z)/22:5(4Z,7Z,10Z,13Z,16Z))	C46H78NO8P	802.537657	No
13307	HMDB0113189	PE-NMe(18:2(9Z,12Z)/22:5(7Z,10Z,13Z,16Z,19Z))	C46H78NO8P	802.537657	No
13307	HMDB0113215	PE-NMe(18:3(6Z,9Z,12Z)/22:4(7Z,10Z,13Z,16Z))	C46H78NO8P	802.537657	No
13307	HMDB0113244	PE-NMe(18:3(9Z,12Z,15Z)/22:4(7Z,10Z,13Z,16Z))	C46H78NO8P	802.537657	No
13307	HMDB0113356	PE-NMe(20:2(11Z,14Z)/20:5(5Z,8Z,11Z,14Z,17Z))	C46H78NO8P	802.537657	No
13307	HMDB0113383	PE-NMe(20:3(5Z,8Z,11Z)/20:4(5Z,8Z,11Z,14Z))	C46H78NO8P	802.537657	No
13307	HMDB0113384	PE-NMe(20:3(5Z,8Z,11Z)/20:4(8Z,11Z,14Z,17Z))	C46H78NO8P	802.537657	No
13307	HMDB0113411	PE-NMe(20:3(8Z,11Z,14Z)/20:4(5Z,8Z,11Z,14Z))	C46H78NO8P	802.537657	No
13307	HMDB0113412	PE-NMe(20:3(8Z,11Z,14Z)/20:4(8Z,11Z,14Z,17Z))	C46H78NO8P	802.537657	No
13307	HMDB0113437	PE-NMe(20:4(5Z,8Z,11Z,14Z)/20:3(5Z,8Z,11Z))	C46H78NO8P	802.537657	No
13307	HMDB0113438	PE-NMe(20:4(5Z,8Z,11Z,14Z)/20:3(8Z,11Z,14Z))	C46H78NO8P	802.537657	No
13307	HMDB0113465	PE-NMe(20:4(8Z,11Z,14Z,17Z)/20:3(5Z,8Z,11Z))	C46H78NO8P	802.537657	No
13307	HMDB0113466	PE-NMe(20:4(8Z,11Z,14Z,17Z)/20:3(8Z,11Z,14Z))	C46H78NO8P	802.537657	No
13307	HMDB0113493	PE-NMe(20:5(5Z,8Z,11Z,14Z,17Z)/20:2(11Z,14Z))	C46H78NO8P	802.537657	No
13307	HMDB0113601	PE-NMe(22:4(7Z,10Z,13Z,16Z)/18:3(6Z,9Z,12Z))	C46H78NO8P	802.537657	No
13307	HMDB0113602	PE-NMe(22:4(7Z,10Z,13Z,16Z)/18:3(9Z,12Z,15Z))	C46H78NO8P	802.537657	No
13307	HMDB0113627	PE-NMe(22:5(4Z,7Z,10Z,13Z,16Z)/18:2(9Z,12Z))	C46H78NO8P	802.537657	No
13307	HMDB0113654	PE-NMe(22:5(7Z,10Z,13Z,16Z,19Z)/18:2(9Z,12Z))	C46H78NO8P	802.537657	No
13307	HMDB0113679	PE-NMe(22:6(4Z,7Z,10Z,13Z,16Z,19Z)/18:1(11Z))	C46H78NO8P	802.537657	No
13307	HMDB0113680	PE-NMe(22:6(4Z,7Z,10Z,13Z,16Z,19Z)/18:1(9Z))	C46H78NO8P	802.537657	No
13314	HMDB0060250; CHEBI:50774	2-Decaprenyl-6-methoxyphenol	C57H88O2	803.6729597	No
13323	HMDB0014652; CHEBI:53773	Acetyldigitoxin	C43H66O14	805.439976	No
13324	HMDB0009780; C00626	PI(16:0/16:2(9Z,12Z))	C41H75O13P	805.4875877	No
13324	HMDB0009798; C00626	PI(16:1(9Z)/16:1(9Z))	C41H75O13P	805.4875877	No
13324	HMDB0009802; C00626	PI(16:2(9Z,12Z)/16:0)	C41H75O13P	805.4875877	No
13339	HMDB0012314; C06125	3-O-Sulfogalactosylceramide (d18:1/18:0)	C42H81NO11S	806.544053	No
13362	HMDB0009778; C00626	PI(16:0/16:0)	C41H79O13P	809.5182862	No
13369	HMDB0015111; C12009	Telithromycin	C43H65N5O10	810.4657762	No
13384	HMDB0015448; C12753; CHEBI:659106	Troleandomycin	C41H67NO15	812.4454239	No
13415	HMDB0010659	PG(18:2(9Z,12Z)/22:6(4Z,7Z,10Z,13Z,16Z,19Z))	C46H75O10P	817.5026952	No
13415	HMDB0010672	PG(18:3(6Z,9Z,12Z)/22:5(4Z,7Z,10Z,13Z,16Z))	C46H75O10P	817.5026952	No
13415	HMDB0010673	PG(18:3(6Z,9Z,12Z)/22:5(7Z,10Z,13Z,16Z,19Z))	C46H75O10P	817.5026952	No
13415	HMDB0010687	PG(18:3(9Z,12Z,15Z)/22:5(4Z,7Z,10Z,13Z,16Z))	C46H75O10P	817.5026952	No
13415	HMDB0010688	PG(18:3(9Z,12Z,15Z)/22:5(7Z,10Z,13Z,16Z,19Z))	C46H75O10P	817.5026952	No
13415	HMDB0116589	PG(20:4(5Z,8Z,11Z,14Z)/20:4(5Z,8Z,11Z,14Z))	C46H75O10P	817.5026952	No
13415	HMDB0116590	PG(20:4(5Z,8Z,11Z,14Z)/20:4(8Z,11Z,14Z,17Z))	C46H75O10P	817.5026952	No
13415	HMDB0116596	PG(20:4(8Z,11Z,14Z,17Z)/20:4(5Z,8Z,11Z,14Z))	C46H75O10P	817.5026952	No
13415	HMDB0116597	PG(20:4(8Z,11Z,14Z,17Z)/20:4(8Z,11Z,14Z,17Z))	C46H75O10P	817.5026952	No
13415	HMDB0116600	PG(22:6(4Z,7Z,10Z,13Z,16Z,19Z)/18:2(9Z,12Z))	C46H75O10P	817.5026952	No
13448	HMDB0015179; C06688; CHEBI:28077	Rifampin	C43H58N4O12	821.397014	No
13448	HMDB0029843; C02284; CHEBI:15939	Glycyrrhizin	C42H62O16	821.397014	No
13448	HMDB0034522	Periandrin I	C42H62O16	821.397014	No
13448	HMDB0039250	Licoricesaponin K2	C42H62O16	821.397014	No
13448	HMDB0039310	Uralsaponin B	C42H62O16	821.397014	No
13449	HMDB0013493	PGP(16:1(9Z)/18:3(6Z,9Z,12Z))	C40H72O13P2	821.4357681	No
13449	HMDB0013563	PGP(18:3(6Z,9Z,12Z)/16:1(9Z))	C40H72O13P2	821.4357681	No
13449	HMDB0013578	PGP(18:3(9Z,12Z,15Z)/16:1(9Z))	C40H72O13P2	821.4357681	No

13492	HMDB0010611	PG(18:0/22:4(7Z,10Z,13Z,16Z))	C46H83O10P	825.564008	No
13503	HMDB0035449	1beta,2alpha,3beta,19alpha-Tetrahydroxy-12-ursen-28	C42H68O16	827.4444187	No
13503	HMDB0040123	Centellasaponin B	C42H68O16	827.4444187	No
13577	HMDB0009782; C00626	PI(16:0/18:1(11Z))	C43H81O13P	835.5317352	No
13577	HMDB0009783; C00626	PI(16:0/18:1(9Z))	C43H81O13P	835.5317352	No
13577	HMDB0009799; C00626	PI(16:1(9Z)/18:0)	C43H81O13P	835.5317352	No
13577	HMDB0009806; C00626	PI(18:0/16:1(9Z))	C43H81O13P	835.5317352	No
13577	HMDB0009822; C00626	PI(18:1(11Z)/16:0)	C43H81O13P	835.5317352	No
13577	HMDB0009834; C00626	PI(18:1(9Z)/16:0)	C43H81O13P	835.5317352	No
13586	HMDB0006805; C02335; CHEBI:15507	Beta-Alanyl-CoA	C24H41N8O17P3S	837.145214	No
13593	HMDB0042113	TG(14:0/15:0/22:5(4Z,7Z,10Z,13Z,16Z))	C54H94O6	837.6978923	No
13593	HMDB0042118	TG(14:0/15:0/22:5(7Z,10Z,13Z,16Z,19Z))	C54H94O6	837.6978923	No
13593	HMDB0042722	TG(14:0/22:5(4Z,7Z,10Z,13Z,16Z)/15:0)	C54H94O6	837.6978923	No
13593	HMDB0042872	TG(14:0/22:5(7Z,10Z,13Z,16Z,19Z)/15:0)	C54H94O6	837.6978923	No
13593	HMDB0042981	TG(15:0/14:0/22:5(4Z,7Z,10Z,13Z,16Z))	C54H94O6	837.6978923	No
13593	HMDB0042986	TG(15:0/14:0/22:5(7Z,10Z,13Z,16Z,19Z))	C54H94O6	837.6978923	No
13593	HMDB0043043	TG(15:0/16:0/20:5(5Z,8Z,11Z,14Z,17Z))	C54H94O6	837.6978923	No
13593	HMDB0043183	TG(15:0/14:1(9Z)/22:4(7Z,10Z,13Z,16Z))	C54H94O6	837.6978923	No
13593	HMDB0043210	TG(15:0/16:1(9Z)/20:4(5Z,8Z,11Z,14Z))	C54H94O6	837.6978923	No
13593	HMDB0043216	TG(15:0/16:1(9Z)/20:4(8Z,11Z,14Z,17Z))	C54H94O6	837.6978923	No
13593	HMDB0043244	TG(15:0/18:1(11Z)/18:4(6Z,9Z,12Z,15Z))	C54H94O6	837.6978923	No
13593	HMDB0043273	TG(15:0/18:1(9Z)/18:4(6Z,9Z,12Z,15Z))	C54H94O6	837.6978923	No
13593	HMDB0043410	TG(15:0/18:2(9Z,12Z)/18:3(6Z,9Z,12Z))	C54H94O6	837.6978923	No
13593	HMDB0043417	TG(15:0/18:2(9Z,12Z)/18:3(9Z,12Z,15Z))	C54H94O6	837.6978923	No
13593	HMDB0043438	TG(15:0/18:3(6Z,9Z,12Z)/18:2(9Z,12Z))	C54H94O6	837.6978923	No
13593	HMDB0043518	TG(15:0/20:4(5Z,8Z,11Z,14Z)/16:1(9Z))	C54H94O6	837.6978923	No
13593	HMDB0043575	TG(15:0/22:4(7Z,10Z,13Z,16Z)/14:1(9Z))	C54H94O6	837.6978923	No
13593	HMDB0043641	TG(15:0/18:3(9Z,12Z,15Z)/18:2(9Z,12Z))	C54H94O6	837.6978923	No
13593	HMDB0043664	TG(15:0/18:4(6Z,9Z,12Z,15Z)/18:1(11Z))	C54H94O6	837.6978923	No
13593	HMDB0043665	TG(15:0/18:4(6Z,9Z,12Z,15Z)/18:1(9Z))	C54H94O6	837.6978923	No
13593	HMDB0043692	TG(15:0/20:4(8Z,11Z,14Z,17Z)/16:1(9Z))	C54H94O6	837.6978923	No
13593	HMDB0043715	TG(15:0/20:5(5Z,8Z,11Z,14Z,17Z)/16:0)	C54H94O6	837.6978923	No
13593	HMDB0043881	TG(16:0/15:0/20:5(5Z,8Z,11Z,14Z,17Z))	C54H94O6	837.6978923	No
13593	HMDB0047761	TG(14:1(9Z)/15:0/22:4(7Z,10Z,13Z,16Z))	C54H94O6	837.6978923	No
13593	HMDB0048446	TG(16:1(9Z)/15:0/20:4(5Z,8Z,11Z,14Z))	C54H94O6	837.6978923	No
13593	HMDB0048452	TG(16:1(9Z)/15:0/20:4(8Z,11Z,14Z,17Z))	C54H94O6	837.6978923	No
13593	HMDB0049108	TG(18:1(11Z)/15:0/18:4(6Z,9Z,12Z,15Z))	C54H94O6	837.6978923	No
13593	HMDB0049735	TG(18:1(9Z)/15:0/18:4(6Z,9Z,12Z,15Z))	C54H94O6	837.6978923	No
13593	HMDB0052412	TG(18:2(9Z,12Z)/15:0/18:3(6Z,9Z,12Z))	C54H94O6	837.6978923	No
13593	HMDB0052419	TG(18:2(9Z,12Z)/15:0/18:3(9Z,12Z,15Z))	C54H94O6	837.6978923	No
13609	HMDB0032812	Isofucosterol 3-O-[6-O-Octadecanoyl-b-D-glucopyrano	C53H92O7	839.6758662	No
13609	HMDB0034089	Sitoindoside II	C53H92O7	839.6758662	No
13614	HMDB0033805	Azaspiracid	C47H71NO12	840.4901821	No
13615	HMDB0008026; C00157	PC(16:1(9Z)/24:1(15Z))	C48H92NO8P	840.6485432	No
13615	HMDB0008053; C00157	PC(18:0/22:2(13Z,16Z))	C48H92NO8P	840.6485432	No
13615	HMDB0008085; C00157	PC(18:1(11Z)/22:1(13Z))	C48H92NO8P	840.6485432	No
13615	HMDB0008118; C00157	PC(18:1(9Z)/22:1(13Z))	C48H92NO8P	840.6485432	No
13615	HMDB0008150; C00157	PC(18:2(9Z,12Z)/22:0)	C48H92NO8P	840.6485432	No
13615	HMDB0008276; C00157	PC(20:0/20:2(11Z,14Z))	C48H92NO8P	840.6485432	No
13615	HMDB0008308; C00157	PC(20:1(11Z)/20:1(11Z))	C48H92NO8P	840.6485432	No
13615	HMDB0008340; C00157	PC(20:2(11Z,14Z)/20:0)	C48H92NO8P	840.6485432	No
13615	HMDB0008533; C00157	PC(22:0/18:2(9Z,12Z))	C48H92NO8P	840.6485432	No
13615	HMDB0008564; C00157	PC(22:1(13Z)/18:1(9Z))	C48H92NO8P	840.6485432	No
13615	HMDB0008595; C00157	PC(22:2(13Z,16Z)/18:0)	C48H92NO8P	840.6485432	No
13615	HMDB0008792; C00157	PC(24:1(15Z)/16:1(9Z))	C48H92NO8P	840.6485432	No
13615	HMDB0011202	PC(22:1(13Z)/18:1(11Z))	C48H92NO8P	840.6485432	No
13615	HMDB0113136	PE-NMe(18:1(11Z)/24:1(15Z))	C48H92NO8P	840.6485432	No
13615	HMDB0113163	PE-NMe(18:1(9Z)/24:1(15Z))	C48H92NO8P	840.6485432	No
13615	HMDB0113191	PE-NMe(18:2(9Z,12Z)/24:0)	C48H92NO8P	840.6485432	No
13615	HMDB0113301	PE-NMe(20:0/22:2(13Z,16Z))	C48H92NO8P	840.6485432	No
13615	HMDB0113329	PE-NMe(20:1(11Z)/22:1(13Z))	C48H92NO8P	840.6485432	No

13615	HMDB0113357	PE-NMe(20:2(11Z,14Z)/22:0)	C48H92NO8P	840.6485432	No
13615	HMDB0113522	PE-NMe(22:0/20:2(11Z,14Z))	C48H92NO8P	840.6485432	No
13615	HMDB0113548	PE-NMe(22:1(13Z)/20:1(11Z))	C48H92NO8P	840.6485432	No
13615	HMDB0113575	PE-NMe(22:2(13Z,16Z)/20:0)	C48H92NO8P	840.6485432	No
13615	HMDB0113709	PE-NMe(24:0/18:2(9Z,12Z))	C48H92NO8P	840.6485432	No
13615	HMDB0113734	PE-NMe(24:1(15Z)/18:1(11Z))	C48H92NO8P	840.6485432	No
13615	HMDB0113735	PE-NMe(24:1(15Z)/18:1(9Z))	C48H92NO8P	840.6485432	No
13645	HMDB0042600	TG(14:0/20:2n6/o-18:0)	C55H104O5	843.7818335	No
13645	HMDB0042948	TG(14:0/o-18:0/20:2n6)	C55H104O5	843.7818335	No
13645	HMDB0044276	TG(16:0/18:2(9Z,12Z)/o-18:0)	C55H104O5	843.7818335	No
13645	HMDB0044654	TG(16:0/o-18:0/18:2(9Z,12Z))	C55H104O5	843.7818335	No
13645	HMDB0047999	TG(14:1(9Z)/20:1(11Z)/o-18:0)	C55H104O5	843.7818335	No
13645	HMDB0048395	TG(14:1(9Z)/o-18:0/20:1(11Z))	C55H104O5	843.7818335	No
13645	HMDB0048632	TG(16:1(9Z)/18:1(11Z)/o-18:0)	C55H104O5	843.7818335	No
13645	HMDB0048654	TG(16:1(9Z)/18:1(9Z)/o-18:0)	C55H104O5	843.7818335	No
13645	HMDB0049052	TG(16:1(9Z)/o-18:0/18:1(11Z))	C55H104O5	843.7818335	No
13645	HMDB0049053	TG(16:1(9Z)/o-18:0/18:1(9Z))	C55H104O5	843.7818335	No
13645	HMDB0049260	TG(18:1(11Z)/16:1(9Z)/o-18:0)	C55H104O5	843.7818335	No
13645	HMDB0049880	TG(18:1(9Z)/16:1(9Z)/o-18:0)	C55H104O5	843.7818335	No
13645	HMDB0050451	TG(20:1(11Z)/14:1(9Z)/o-18:0)	C55H104O5	843.7818335	No
13645	HMDB0052440	TG(18:2(9Z,12Z)/16:0/o-18:0)	C55H104O5	843.7818335	No
13645	HMDB0053276	TG(20:2n6/14:0/o-18:0)	C55H104O5	843.7818335	No
13664	HMDB0013568	PGP(18:3(6Z,9Z,12Z)/18:3(6Z,9Z,12Z))	C42H72O13P2	845.4358457	No
13664	HMDB0013569	PGP(18:3(6Z,9Z,12Z)/18:3(9Z,12Z,15Z))	C42H72O13P2	845.4358457	No
13664	HMDB0013583	PGP(18:3(9Z,12Z,15Z)/18:3(6Z,9Z,12Z))	C42H72O13P2	845.4358457	No
13664	HMDB0013584	PGP(18:3(9Z,12Z,15Z)/18:3(9Z,12Z,15Z))	C42H72O13P2	845.4358457	No
13664	HMDB0014753; C07235; CHEBI:45367	Rifabutin	C46H62N4O11	845.4358457	No
13671	HMDB0042567	TG(14:0/18:3(6Z,9Z,12Z)/20:5(5Z,8Z,11Z,14Z,17Z))	C55H90O6	845.6655777	No
13671	HMDB0042655	TG(14:0/20:4(5Z,8Z,11Z,14Z)/18:4(6Z,9Z,12Z,15Z))	C55H90O6	845.6655777	No
13671	HMDB0042777	TG(14:0/18:3(9Z,12Z,15Z)/20:5(5Z,8Z,11Z,14Z,17Z))	C55H90O6	845.6655777	No
13671	HMDB0042800	TG(14:0/18:4(6Z,9Z,12Z,15Z)/20:4(5Z,8Z,11Z,14Z))	C55H90O6	845.6655777	No
13671	HMDB0042806	TG(14:0/18:4(6Z,9Z,12Z,15Z)/20:4(8Z,11Z,14Z,17Z))	C55H90O6	845.6655777	No
13671	HMDB0042835	TG(14:0/20:4(8Z,11Z,14Z,17Z)/18:4(6Z,9Z,12Z,15Z))	C55H90O6	845.6655777	No
13671	HMDB0042857	TG(14:0/20:5(5Z,8Z,11Z,14Z,17Z)/18:3(6Z,9Z,12Z))	C55H90O6	845.6655777	No
13671	HMDB0042864	TG(14:0/20:5(5Z,8Z,11Z,14Z,17Z)/18:3(9Z,12Z,15Z))	C55H90O6	845.6655777	No
13671	HMDB0044523	TG(16:0/18:4(6Z,9Z,12Z,15Z)/18:4(6Z,9Z,12Z,15Z))	C55H90O6	845.6655777	No
13671	HMDB0047929	TG(14:1(9Z)/16:1(9Z)/22:6(4Z,7Z,10Z,13Z,16Z,19Z))	C55H90O6	845.6655777	No
13671	HMDB0048017	TG(14:1(9Z)/20:3(5Z,8Z,11Z)/18:4(6Z,9Z,12Z,15Z))	C55H90O6	845.6655777	No
13671	HMDB0048088	TG(14:1(9Z)/18:2(9Z,12Z)/20:5(5Z,8Z,11Z,14Z,17Z))	C55H90O6	845.6655777	No
13671	HMDB0048104	TG(14:1(9Z)/18:3(6Z,9Z,12Z)/20:4(5Z,8Z,11Z,14Z))	C55H90O6	845.6655777	No
13671	HMDB0048110	TG(14:1(9Z)/18:3(6Z,9Z,12Z)/20:4(8Z,11Z,14Z,17Z))	C55H90O6	845.6655777	No
13671	HMDB0048155	TG(14:1(9Z)/20:3n6/18:4(6Z,9Z,12Z,15Z))	C55H90O6	845.6655777	No
13671	HMDB0048170	TG(14:1(9Z)/20:4(5Z,8Z,11Z,14Z)/18:3(6Z,9Z,12Z))	C55H90O6	845.6655777	No
13671	HMDB0048177	TG(14:1(9Z)/20:4(5Z,8Z,11Z,14Z)/18:3(9Z,12Z,15Z))	C55H90O6	845.6655777	No
13671	HMDB0048265	TG(14:1(9Z)/18:3(9Z,12Z,15Z)/20:4(5Z,8Z,11Z,14Z))	C55H90O6	845.6655777	No
13671	HMDB0048271	TG(14:1(9Z)/18:3(9Z,12Z,15Z)/20:4(8Z,11Z,14Z,17Z))	C55H90O6	845.6655777	No
13671	HMDB0048281	TG(14:1(9Z)/18:4(6Z,9Z,12Z,15Z)/20:3(5Z,8Z,11Z))	C55H90O6	845.6655777	No
13671	HMDB0048287	TG(14:1(9Z)/18:4(6Z,9Z,12Z,15Z)/20:3n6)	C55H90O6	845.6655777	No
13671	HMDB0048308	TG(14:1(9Z)/20:4(8Z,11Z,14Z,17Z)/18:3(6Z,9Z,12Z))	C55H90O6	845.6655777	No
13671	HMDB0048315	TG(14:1(9Z)/20:4(8Z,11Z,14Z,17Z)/18:3(9Z,12Z,15Z))	C55H90O6	845.6655777	No
13671	HMDB0048330	TG(14:1(9Z)/20:5(5Z,8Z,11Z,14Z,17Z)/18:2(9Z,12Z))	C55H90O6	845.6655777	No
13671	HMDB0048369	TG(14:1(9Z)/22:6(4Z,7Z,10Z,13Z,16Z,19Z)/16:1(9Z))	C55H90O6	845.6655777	No
13671	HMDB0048587	TG(16:1(9Z)/14:1(9Z)/22:6(4Z,7Z,10Z,13Z,16Z,19Z))	C55H90O6	845.6655777	No
13671	HMDB0048781	TG(16:1(9Z)/18:3(6Z,9Z,12Z)/18:4(6Z,9Z,12Z,15Z))	C55H90O6	845.6655777	No
13671	HMDB0048935	TG(16:1(9Z)/18:3(9Z,12Z,15Z)/18:4(6Z,9Z,12Z,15Z))	C55H90O6	845.6655777	No
13671	HMDB0048949	TG(16:1(9Z)/18:4(6Z,9Z,12Z,15Z)/18:3(6Z,9Z,12Z))	C55H90O6	845.6655777	No
13671	HMDB0048956	TG(16:1(9Z)/18:4(6Z,9Z,12Z,15Z)/18:3(9Z,12Z,15Z))	C55H90O6	845.6655777	No
13671	HMDB0051007	TG(20:3(5Z,8Z,11Z)/14:1(9Z)/18:4(6Z,9Z,12Z,15Z))	C55H90O6	845.6655777	No
13671	HMDB0052512	TG(18:2(9Z,12Z)/14:1(9Z)/20:5(5Z,8Z,11Z,14Z,17Z))	C55H90O6	845.6655777	No
13671	HMDB0052855	TG(18:3(6Z,9Z,12Z)/14:0/20:5(5Z,8Z,11Z,14Z,17Z))	C55H90O6	845.6655777	No
13671	HMDB0052946	TG(18:3(6Z,9Z,12Z)/14:1(9Z)/20:4(5Z,8Z,11Z,14Z))	C55H90O6	845.6655777	No

13671	HMDB0052952	TG(18:3(6Z,9Z,12Z)/14:1(9Z)/20:4(8Z,11Z,14Z,17Z))	C55H90O6	845.6655777	No
13671	HMDB0052965	TG(18:3(6Z,9Z,12Z)/16:1(9Z)/18:4(6Z,9Z,12Z,15Z))	C55H90O6	845.6655777	No
13671	HMDB0053743	TG(20:3n6/14:1(9Z)/18:4(6Z,9Z,12Z,15Z))	C55H90O6	845.6655777	No
13671	HMDB0054017	TG(20:4(5Z,8Z,11Z,14Z)/14:0/18:4(6Z,9Z,12Z,15Z))	C55H90O6	845.6655777	No
13671	HMDB0054093	TG(20:4(5Z,8Z,11Z,14Z)/14:1(9Z)/18:3(9Z,12Z,15Z))	C55H90O6	845.6655777	No
13671	HMDB0055151	TG(18:3(9Z,12Z,15Z)/14:0/20:5(5Z,8Z,11Z,14Z,17Z))	C55H90O6	845.6655777	No
13671	HMDB0055199	TG(18:3(9Z,12Z,15Z)/14:1(9Z)/20:4(8Z,11Z,14Z,17Z))	C55H90O6	845.6655777	No
13671	HMDB0055205	TG(18:3(9Z,12Z,15Z)/16:1(9Z)/18:4(6Z,9Z,12Z,15Z))	C55H90O6	845.6655777	No
13671	HMDB0055358	TG(18:4(6Z,9Z,12Z,15Z)/14:0/20:4(8Z,11Z,14Z,17Z))	C55H90O6	845.6655777	No
13671	HMDB0055369	TG(18:4(6Z,9Z,12Z,15Z)/16:0/18:4(6Z,9Z,12Z,15Z))	C55H90O6	845.6655777	No
13672	HMDB0042104	TG(14:0/15:0/22:1(13Z))	C54H102O6	845.7622773	No
13672	HMDB0042452	TG(14:0/22:1(13Z)/15:0)	C54H102O6	845.7622773	No
13672	HMDB0042972	TG(15:0/14:0/22:1(13Z))	C54H102O6	845.7622773	No
13672	HMDB0043028	TG(15:0/16:0/20:1(11Z))	C54H102O6	845.7622773	No
13672	HMDB0043055	TG(15:0/18:0/18:1(11Z))	C54H102O6	845.7622773	No
13672	HMDB0043056	TG(15:0/18:0/18:1(9Z))	C54H102O6	845.7622773	No
13672	HMDB0043083	TG(15:0/20:0/16:1(9Z))	C54H102O6	845.7622773	No
13672	HMDB0043111	TG(15:0/22:0/14:1(9Z))	C54H102O6	845.7622773	No
13672	HMDB0043167	TG(15:0/14:1(9Z)/22:0)	C54H102O6	845.7622773	No
13672	HMDB0043195	TG(15:0/16:1(9Z)/20:0)	C54H102O6	845.7622773	No
13672	HMDB0043223	TG(15:0/18:1(11Z)/18:0)	C54H102O6	845.7622773	No
13672	HMDB0043252	TG(15:0/18:1(9Z)/18:0)	C54H102O6	845.7622773	No
13672	HMDB0043280	TG(15:0/20:1(11Z)/16:0)	C54H102O6	845.7622773	No
13672	HMDB0043866	TG(16:0/15:0/20:1(11Z))	C54H102O6	845.7622773	No
13672	HMDB0044701	TG(18:0/15:0/18:1(11Z))	C54H102O6	845.7622773	No
13672	HMDB0044702	TG(18:0/15:0/18:1(9Z))	C54H102O6	845.7622773	No
13672	HMDB0045507	TG(20:0/15:0/16:1(9Z))	C54H102O6	845.7622773	No
13672	HMDB0046283	TG(22:0/15:0/14:1(9Z))	C54H102O6	845.7622773	No
13706	HMDB0034601	Hebevinoside XI	C46H74O14	849.4989956	No
13706	HMDB0035981	Hebevinoside V	C46H74O14	849.4989956	No
13708	HMDB0062350; CHEBI:64182	3-demethylubiquinol-10	C58H90O4	849.674775	No
13738	HMDB0041548	Hovenidulcioside B1	C44H70O16	853.4571691	No
13739	HMDB0013507	PGP(18:0/18:2(9Z,12Z))	C42H80O13P2	853.4993728	No
13739	HMDB0013520	PGP(18:1(11Z)/18:1(11Z))	C42H80O13P2	853.4993728	No
13739	HMDB0013521	PGP(18:1(11Z)/18:1(9Z))	C42H80O13P2	853.4993728	No
13739	HMDB0013535	PGP(18:1(9Z)/18:1(11Z))	C42H80O13P2	853.4993728	No
13739	HMDB0013536	PGP(18:1(9Z)/18:1(9Z))	C42H80O13P2	853.4993728	No
13739	HMDB0013549	PGP(18:2(9Z,12Z)/18:0)	C42H80O13P2	853.4993728	No
13745	HMDB0012439	PS(20:4(5Z,8Z,11Z,14Z)/22:6(4Z,7Z,10Z,13Z,16Z,19Z))	C48H74NO10P	854.4998277	No
13745	HMDB0012449	PS(22:6(4Z,7Z,10Z,13Z,16Z,19Z)/20:4(5Z,8Z,11Z,14Z))	C48H74NO10P	854.4998277	No
13745	HMDB0112677	PS(20:4(8Z,11Z,14Z,17Z)/22:6(4Z,7Z,10Z,13Z,16Z,19Z)	C48H74NO10P	854.4998277	No
13745	HMDB0112702	PS(20:5(5Z,8Z,11Z,14Z,17Z)/22:5(4Z,7Z,10Z,13Z,16Z))	C48H74NO10P	854.4998277	No
13745	HMDB0112703	PS(20:5(5Z,8Z,11Z,14Z,17Z)/22:5(7Z,10Z,13Z,16Z,19Z)	C48H74NO10P	854.4998277	No
13745	HMDB0112831	PS(22:5(4Z,7Z,10Z,13Z,16Z)/20:5(5Z,8Z,11Z,14Z,17Z))	C48H74NO10P	854.4998277	No
13745	HMDB0112872	PS(22:6(4Z,7Z,10Z,13Z,16Z,19Z)/20:4(8Z,11Z,14Z,17Z)	C48H74NO10P	854.4998277	No
13745	HMDB0116790	PS(22:5(7Z,10Z,13Z,16Z,19Z)/20:5(5Z,8Z,11Z,14Z,17Z)	C48H74NO10P	854.4998277	No
13769	HMDB0009789; C00626	PI(16:0/20:4(5Z,8Z,11Z,14Z))	C45H79O13P	857.516061	No
13769	HMDB0009790; C00626	PI(16:0/20:4(8Z,11Z,14Z,17Z))	C45H79O13P	857.516061	No
13769	HMDB0009827; C00626	PI(18:1(11Z)/18:3(6Z,9Z,12Z))	C45H79O13P	857.516061	No
13769	HMDB0009828; C00626	PI(18:1(11Z)/18:3(9Z,12Z,15Z))	C45H79O13P	857.516061	No
13769	HMDB0009839; C00626	PI(18:1(9Z)/18:3(6Z,9Z,12Z))	C45H79O13P	857.516061	No
13769	HMDB0009840; C00626	PI(18:1(9Z)/18:3(9Z,12Z,15Z))	C45H79O13P	857.516061	No
13769	HMDB0009850; C00626	PI(18:2(9Z,12Z)/18:2(9Z,12Z))	C45H79O13P	857.516061	No
13769	HMDB0009859; C00626	PI(18:3(6Z,9Z,12Z)/18:1(11Z))	C45H79O13P	857.516061	No
13769	HMDB0009860; C00626	PI(18:3(6Z,9Z,12Z)/18:1(9Z))	C45H79O13P	857.516061	No
13769	HMDB0009863; C00626	PI(18:3(9Z,12Z,15Z)/18:1(11Z))	C45H79O13P	857.516061	No
13769	HMDB0009864; C00626	PI(18:3(9Z,12Z,15Z)/18:1(9Z))	C45H79O13P	857.516061	No
13769	HMDB0009893; C00626	PI(20:4(5Z,8Z,11Z,14Z)/16:0)	C45H79O13P	857.516061	No
13769	HMDB0009899; C00626	PI(20:4(8Z,11Z,14Z,17Z)/16:0)	C45H79O13P	857.516061	No
13769	HMDB0062771	1,2-dilinoleoyl-sn-glycero-3-phospho-1D-myo-inositol	C45H79O13P	857.516061	No
13813	HMDB0043104	TG(15:0/20:0/o-18:0)	C56H110O5	861.8299156	No

13813	HMDB0043804	TG(15:0/o-18:0/20:0)	C56H110O5	861.8299156	No
13813	HMDB0045528	TG(20:0/15:0/o-18:0)	C56H110O5	861.8299156	No
13863	HMDB0041351	Koryoginsenoside R1	C46H76O15	867.5082825	No
13917	HMDB0033490	Pectenotoxin 1	C47H70O15	873.465657	No
13938	HMDB0015332; C08059; CHEBI:45304	Rifapentine	C47H64N4O12	875.4436297	No
13942	HMDB0005447; C00422	TG(16:1(9Z)/18:2(9Z,12Z)/20:4(5Z,8Z,11Z,14Z))	C57H96O6	875.7107486	No
13942	HMDB0010475	TG(18:2(9Z,12Z)/16:0/20:5(5Z,8Z,11Z,14Z,17Z))	C57H96O6	875.7107486	No
13942	HMDB0010489	TG(18:2(9Z,12Z)/18:2(9Z,12Z)/18:3(6Z,9Z,12Z))	C57H96O6	875.7107486	No
13942	HMDB0010490	TG(18:2(9Z,12Z)/18:2(9Z,12Z)/18:3(9Z,12Z,15Z))	C57H96O6	875.7107486	No
13942	HMDB0010502	TG(18:3(9Z,12Z,15Z)/18:1(11Z)/18:3(9Z,12Z,15Z))	C57H96O6	875.7107486	No
13942	HMDB0010503	TG(18:3(9Z,12Z,15Z)/18:1(9Z)/18:3(9Z,12Z,15Z))	C57H96O6	875.7107486	No
13942	HMDB0010519	TG(20:4(5Z,8Z,11Z,14Z)/16:0/18:3(9Z,12Z,15Z))	C57H96O6	875.7107486	No
13942	HMDB0042359	TG(14:0/18:1(11Z)/22:6(4Z,7Z,10Z,13Z,16Z,19Z))	C57H96O6	875.7107486	No
13942	HMDB0042389	TG(14:0/18:1(9Z)/22:6(4Z,7Z,10Z,13Z,16Z,19Z))	C57H96O6	875.7107486	No
13942	HMDB0042440	TG(14:0/20:3(5Z,8Z,11Z)/20:4(5Z,8Z,11Z,14Z))	C57H96O6	875.7107486	No
13942	HMDB0042446	TG(14:0/20:3(5Z,8Z,11Z)/20:4(8Z,11Z,14Z,17Z))	C57H96O6	875.7107486	No
13942	HMDB0042533	TG(14:0/18:2(9Z,12Z)/22:5(4Z,7Z,10Z,13Z,16Z))	C57H96O6	875.7107486	No
13942	HMDB0042538	TG(14:0/18:2(9Z,12Z)/22:5(7Z,10Z,13Z,16Z,19Z))	C57H96O6	875.7107486	No
13942	HMDB0042562	TG(14:0/18:3(6Z,9Z,12Z)/22:4(7Z,10Z,13Z,16Z))	C57H96O6	875.7107486	No
13942	HMDB0042597	TG(14:0/20:2n6/20:5(5Z,8Z,11Z,14Z,17Z))	C57H96O6	875.7107486	No
13942	HMDB0042620	TG(14:0/20:3n6/20:4(5Z,8Z,11Z,14Z))	C57H96O6	875.7107486	No
13942	HMDB0042626	TG(14:0/20:3n6/20:4(8Z,11Z,14Z,17Z))	C57H96O6	875.7107486	No
13942	HMDB0042643	TG(14:0/20:4(5Z,8Z,11Z,14Z)/20:3(5Z,8Z,11Z))	C57H96O6	875.7107486	No
13942	HMDB0042649	TG(14:0/20:4(5Z,8Z,11Z,14Z)/20:3n6)	C57H96O6	875.7107486	No
13942	HMDB0042707	TG(14:0/22:4(7Z,10Z,13Z,16Z)/18:3(6Z,9Z,12Z))	C57H96O6	875.7107486	No
13942	HMDB0042714	TG(14:0/22:4(7Z,10Z,13Z,16Z)/18:3(9Z,12Z,15Z))	C57H96O6	875.7107486	No
13942	HMDB0042736	TG(14:0/22:5(4Z,7Z,10Z,13Z,16Z)/18:2(9Z,12Z))	C57H96O6	875.7107486	No
13942	HMDB0042772	TG(14:0/18:3(9Z,12Z,15Z)/22:4(7Z,10Z,13Z,16Z))	C57H96O6	875.7107486	No
13942	HMDB0042823	TG(14:0/20:4(8Z,11Z,14Z,17Z)/20:3(5Z,8Z,11Z))	C57H96O6	875.7107486	No
13942	HMDB0042829	TG(14:0/20:4(8Z,11Z,14Z,17Z)/20:3n6)	C57H96O6	875.7107486	No
13942	HMDB0042858	TG(14:0/20:5(5Z,8Z,11Z,14Z,17Z)/20:2n6)	C57H96O6	875.7107486	No
13942	HMDB0042886	TG(14:0/22:5(7Z,10Z,13Z,16Z,19Z)/18:2(9Z,12Z))	C57H96O6	875.7107486	No
13942	HMDB0042910	TG(14:0/22:6(4Z,7Z,10Z,13Z,16Z,19Z)/18:1(11Z))	C57H96O6	875.7107486	No
13942	HMDB0042911	TG(14:0/22:6(4Z,7Z,10Z,13Z,16Z,19Z)/18:1(9Z))	C57H96O6	875.7107486	No
13942	HMDB0044079	TG(16:0/16:1(9Z)/22:6(4Z,7Z,10Z,13Z,16Z,19Z))	C57H96O6	875.7107486	No
13942	HMDB0044187	TG(16:0/20:3(5Z,8Z,11Z)/18:4(6Z,9Z,12Z,15Z))	C57H96O6	875.7107486	No
13942	HMDB0044273	TG(16:0/18:2(9Z,12Z)/20:5(5Z,8Z,11Z,14Z,17Z))	C57H96O6	875.7107486	No
13942	HMDB0044294	TG(16:0/18:3(6Z,9Z,12Z)/20:4(5Z,8Z,11Z,14Z))	C57H96O6	875.7107486	No
13942	HMDB0044300	TG(16:0/18:3(6Z,9Z,12Z)/20:4(8Z,11Z,14Z,17Z))	C57H96O6	875.7107486	No
13942	HMDB0044355	TG(16:0/20:3n6/18:4(6Z,9Z,12Z,15Z))	C57H96O6	875.7107486	No
13942	HMDB0044375	TG(16:0/20:4(5Z,8Z,11Z,14Z)/18:3(6Z,9Z,12Z))	C57H96O6	875.7107486	No
13942	HMDB0044382	TG(16:0/20:4(5Z,8Z,11Z,14Z)/18:3(9Z,12Z,15Z))	C57H96O6	875.7107486	No
13942	HMDB0044490	TG(16:0/18:3(9Z,12Z,15Z)/20:4(5Z,8Z,11Z,14Z))	C57H96O6	875.7107486	No
13942	HMDB0044496	TG(16:0/18:3(9Z,12Z,15Z)/20:4(8Z,11Z,14Z,17Z))	C57H96O6	875.7107486	No
13942	HMDB0044511	TG(16:0/18:4(6Z,9Z,12Z,15Z)/20:3(5Z,8Z,11Z))	C57H96O6	875.7107486	No
13942	HMDB0044517	TG(16:0/18:4(6Z,9Z,12Z,15Z)/20:3n6)	C57H96O6	875.7107486	No
13942	HMDB0044543	TG(16:0/20:4(8Z,11Z,14Z,17Z)/18:3(6Z,9Z,12Z))	C57H96O6	875.7107486	No
13942	HMDB0044550	TG(16:0/20:4(8Z,11Z,14Z,17Z)/18:3(9Z,12Z,15Z))	C57H96O6	875.7107486	No
13942	HMDB0044570	TG(16:0/20:5(5Z,8Z,11Z,14Z,17Z)/18:2(9Z,12Z))	C57H96O6	875.7107486	No
13942	HMDB0044619	TG(16:0/22:6(4Z,7Z,10Z,13Z,16Z,19Z)/16:1(9Z))	C57H96O6	875.7107486	No
13942	HMDB0044882	TG(18:0/14:1(9Z)/22:6(4Z,7Z,10Z,13Z,16Z,19Z))	C57H96O6	875.7107486	No
13942	HMDB0045121	TG(18:0/18:3(6Z,9Z,12Z)/18:4(6Z,9Z,12Z,15Z))	C57H96O6	875.7107486	No
13942	HMDB0045310	TG(18:0/18:3(9Z,12Z,15Z)/18:4(6Z,9Z,12Z,15Z))	C57H96O6	875.7107486	No
13942	HMDB0045329	TG(18:0/18:4(6Z,9Z,12Z,15Z)/18:3(6Z,9Z,12Z))	C57H96O6	875.7107486	No
13942	HMDB0045336	TG(18:0/18:4(6Z,9Z,12Z,15Z)/18:3(9Z,12Z,15Z))	C57H96O6	875.7107486	No
13942	HMDB0045428	TG(18:0/22:6(4Z,7Z,10Z,13Z,16Z,19Z)/14:1(9Z))	C57H96O6	875.7107486	No
13942	HMDB0047814	TG(14:1(9Z)/18:0/22:6(4Z,7Z,10Z,13Z,16Z,19Z))	C57H96O6	875.7107486	No
13942	HMDB0047946	TG(14:1(9Z)/18:1(11Z)/22:5(4Z,7Z,10Z,13Z,16Z))	C57H96O6	875.7107486	No
13942	HMDB0047951	TG(14:1(9Z)/18:1(11Z)/22:5(7Z,10Z,13Z,16Z,19Z))	C57H96O6	875.7107486	No
13942	HMDB0047969	TG(14:1(9Z)/18:1(9Z)/22:5(4Z,7Z,10Z,13Z,16Z))	C57H96O6	875.7107486	No
13942	HMDB0047974	TG(14:1(9Z)/18:1(9Z)/22:5(7Z,10Z,13Z,16Z,19Z))	C57H96O6	875.7107486	No

13942	HMDB0047996	TG(14:1(9Z)/20:1(11Z)/20:5(5Z,8Z,11Z,14Z,17Z))	C57H96O6	875.7107486	No
13942	HMDB0048005	TG(14:1(9Z)/20:3(5Z,8Z,11Z)/20:3(5Z,8Z,11Z))	C57H96O6	875.7107486	No
13942	HMDB0048011	TG(14:1(9Z)/20:3(5Z,8Z,11Z)/20:3n6)	C57H96O6	875.7107486	No
13942	HMDB0048083	TG(14:1(9Z)/18:2(9Z,12Z)/22:4(7Z,10Z,13Z,16Z))	C57H96O6	875.7107486	No
13942	HMDB0048127	TG(14:1(9Z)/20:2n6/20:4(5Z,8Z,11Z,14Z))	C57H96O6	875.7107486	No
13942	HMDB0048133	TG(14:1(9Z)/20:2n6/20:4(8Z,11Z,14Z,17Z))	C57H96O6	875.7107486	No
13942	HMDB0048143	TG(14:1(9Z)/20:3n6/20:3(5Z,8Z,11Z))	C57H96O6	875.7107486	No
13942	HMDB0048149	TG(14:1(9Z)/20:3n6/20:3n6)	C57H96O6	875.7107486	No
13942	HMDB0048171	TG(14:1(9Z)/20:4(5Z,8Z,11Z,14Z)/20:2n6)	C57H96O6	875.7107486	No
13942	HMDB0048201	TG(14:1(9Z)/22:2(13Z,16Z)/18:4(6Z,9Z,12Z,15Z))	C57H96O6	875.7107486	No
13942	HMDB0048215	TG(14:1(9Z)/22:4(7Z,10Z,13Z,16Z)/18:2(9Z,12Z))	C57H96O6	875.7107486	No
13942	HMDB0048232	TG(14:1(9Z)/22:5(4Z,7Z,10Z,13Z,16Z)/18:1(11Z))	C57H96O6	875.7107486	No
13942	HMDB0048233	TG(14:1(9Z)/22:5(4Z,7Z,10Z,13Z,16Z)/18:1(9Z))	C57H96O6	875.7107486	No
13942	HMDB0048289	TG(14:1(9Z)/18:4(6Z,9Z,12Z,15Z)/22:2(13Z,16Z))	C57H96O6	875.7107486	No
13942	HMDB0048309	TG(14:1(9Z)/20:4(8Z,11Z,14Z,17Z)/20:2n6)	C57H96O6	875.7107486	No
13942	HMDB0048326	TG(14:1(9Z)/20:5(5Z,8Z,11Z,14Z,17Z)/20:1(11Z))	C57H96O6	875.7107486	No
13942	HMDB0048347	TG(14:1(9Z)/22:5(7Z,10Z,13Z,16Z,19Z)/18:1(11Z))	C57H96O6	875.7107486	No
13942	HMDB0048348	TG(14:1(9Z)/22:5(7Z,10Z,13Z,16Z,19Z)/18:1(9Z))	C57H96O6	875.7107486	No
13942	HMDB0048477	TG(16:1(9Z)/16:0/22:6(4Z,7Z,10Z,13Z,16Z,19Z))	C57H96O6	875.7107486	No
13942	HMDB0048603	TG(16:1(9Z)/16:1(9Z)/22:5(4Z,7Z,10Z,13Z,16Z))	C57H96O6	875.7107486	No
13942	HMDB0048608	TG(16:1(9Z)/16:1(9Z)/22:5(7Z,10Z,13Z,16Z,19Z))	C57H96O6	875.7107486	No
13942	HMDB0048629	TG(16:1(9Z)/18:1(11Z)/20:5(5Z,8Z,11Z,14Z,17Z))	C57H96O6	875.7107486	No
13942	HMDB0048651	TG(16:1(9Z)/18:1(9Z)/20:5(5Z,8Z,11Z,14Z,17Z))	C57H96O6	875.7107486	No
13942	HMDB0048685	TG(16:1(9Z)/20:3(5Z,8Z,11Z)/18:3(6Z,9Z,12Z))	C57H96O6	875.7107486	No
13942	HMDB0048692	TG(16:1(9Z)/20:3(5Z,8Z,11Z)/18:3(9Z,12Z,15Z))	C57H96O6	875.7107486	No
13942	HMDB0048760	TG(16:1(9Z)/18:2(9Z,12Z)/20:4(8Z,11Z,14Z,17Z))	C57H96O6	875.7107486	No
13942	HMDB0048769	TG(16:1(9Z)/18:3(6Z,9Z,12Z)/20:3(5Z,8Z,11Z))	C57H96O6	875.7107486	No
13942	HMDB0048775	TG(16:1(9Z)/18:3(6Z,9Z,12Z)/20:3n6)	C57H96O6	875.7107486	No
13942	HMDB0048803	TG(16:1(9Z)/20:2n6/18:4(6Z,9Z,12Z,15Z))	C57H96O6	875.7107486	No
13942	HMDB0048817	TG(16:1(9Z)/20:3n6/18:3(6Z,9Z,12Z))	C57H96O6	875.7107486	No
13942	HMDB0048824	TG(16:1(9Z)/20:3n6/18:3(9Z,12Z,15Z))	C57H96O6	875.7107486	No
13942	HMDB0048838	TG(16:1(9Z)/20:4(5Z,8Z,11Z,14Z)/18:2(9Z,12Z))	C57H96O6	875.7107486	No
13942	HMDB0048897	TG(16:1(9Z)/22:5(4Z,7Z,10Z,13Z,16Z)/16:1(9Z))	C57H96O6	875.7107486	No
13942	HMDB0048923	TG(16:1(9Z)/18:3(9Z,12Z,15Z)/20:3(5Z,8Z,11Z))	C57H96O6	875.7107486	No
13942	HMDB0048929	TG(16:1(9Z)/18:3(9Z,12Z,15Z)/20:3n6)	C57H96O6	875.7107486	No
13942	HMDB0048950	TG(16:1(9Z)/18:4(6Z,9Z,12Z,15Z)/20:2n6)	C57H96O6	875.7107486	No
13942	HMDB0048970	TG(16:1(9Z)/20:4(8Z,11Z,14Z,17Z)/18:2(9Z,12Z))	C57H96O6	875.7107486	No
13942	HMDB0048986	TG(16:1(9Z)/20:5(5Z,8Z,11Z,14Z,17Z)/18:1(11Z))	C57H96O6	875.7107486	No
13942	HMDB0048987	TG(16:1(9Z)/20:5(5Z,8Z,11Z,14Z,17Z)/18:1(9Z))	C57H96O6	875.7107486	No
13942	HMDB0049007	TG(16:1(9Z)/22:5(7Z,10Z,13Z,16Z,19Z)/16:1(9Z))	C57H96O6	875.7107486	No
13942	HMDB0049091	TG(18:1(11Z)/14:0/22:6(4Z,7Z,10Z,13Z,16Z,19Z))	C57H96O6	875.7107486	No
13942	HMDB0049232	TG(18:1(11Z)/14:1(9Z)/22:5(4Z,7Z,10Z,13Z,16Z))	C57H96O6	875.7107486	No
13942	HMDB0049237	TG(18:1(11Z)/14:1(9Z)/22:5(7Z,10Z,13Z,16Z,19Z))	C57H96O6	875.7107486	No
13942	HMDB0049257	TG(18:1(11Z)/16:1(9Z)/20:5(5Z,8Z,11Z,14Z,17Z))	C57H96O6	875.7107486	No
13942	HMDB0049402	TG(18:1(11Z)/18:2(9Z,12Z)/18:4(6Z,9Z,12Z,15Z))	C57H96O6	875.7107486	No
13942	HMDB0049415	TG(18:1(11Z)/18:3(6Z,9Z,12Z)/18:3(6Z,9Z,12Z))	C57H96O6	875.7107486	No
13942	HMDB0049422	TG(18:1(11Z)/18:3(6Z,9Z,12Z)/18:3(9Z,12Z,15Z))	C57H96O6	875.7107486	No
13942	HMDB0049562	TG(18:1(11Z)/18:3(9Z,12Z,15Z)/18:3(6Z,9Z,12Z))	C57H96O6	875.7107486	No
13942	HMDB0049569	TG(18:1(11Z)/18:3(9Z,12Z,15Z)/18:3(9Z,12Z,15Z))	C57H96O6	875.7107486	No
13942	HMDB0049582	TG(18:1(11Z)/18:4(6Z,9Z,12Z,15Z)/18:2(9Z,12Z))	C57H96O6	875.7107486	No
13942	HMDB0049719	TG(18:1(9Z)/14:0/22:6(4Z,7Z,10Z,13Z,16Z,19Z))	C57H96O6	875.7107486	No
13942	HMDB0049853	TG(18:1(9Z)/14:1(9Z)/22:5(4Z,7Z,10Z,13Z,16Z))	C57H96O6	875.7107486	No
13942	HMDB0049858	TG(18:1(9Z)/14:1(9Z)/22:5(7Z,10Z,13Z,16Z,19Z))	C57H96O6	875.7107486	No
13942	HMDB0049877	TG(18:1(9Z)/16:1(9Z)/20:5(5Z,8Z,11Z,14Z,17Z))	C57H96O6	875.7107486	No
13942	HMDB0050015	TG(18:1(9Z)/18:2(9Z,12Z)/18:4(6Z,9Z,12Z,15Z))	C57H96O6	875.7107486	No
13942	HMDB0050027	TG(18:1(9Z)/18:3(6Z,9Z,12Z)/18:3(6Z,9Z,12Z))	C57H96O6	875.7107486	No
13942	HMDB0050034	TG(18:1(9Z)/18:3(6Z,9Z,12Z)/18:3(9Z,12Z,15Z))	C57H96O6	875.7107486	No
13942	HMDB0050167	TG(18:1(9Z)/18:3(9Z,12Z,15Z)/18:3(6Z,9Z,12Z))	C57H96O6	875.7107486	No
13942	HMDB0050174	TG(18:1(9Z)/18:3(9Z,12Z,15Z)/18:3(9Z,12Z,15Z))	C57H96O6	875.7107486	No
13942	HMDB0050186	TG(18:1(9Z)/18:4(6Z,9Z,12Z,15Z)/18:2(9Z,12Z))	C57H96O6	875.7107486	No
13942	HMDB0050448	TG(20:1(11Z)/14:1(9Z)/20:5(5Z,8Z,11Z,14Z,17Z))	C57H96O6	875.7107486	No

13942	HMDB0050876	TG(20:3(5Z,8Z,11Z)/14:0/20:4(5Z,8Z,11Z,14Z))	C57H96O6	875.7107486	No
13942	HMDB0050882	TG(20:3(5Z,8Z,11Z)/14:0/20:4(8Z,11Z,14Z,17Z))	C57H96O6	875.7107486	No
13942	HMDB0050917	TG(20:3(5Z,8Z,11Z)/16:0/18:4(6Z,9Z,12Z,15Z))	C57H96O6	875.7107486	No
13942	HMDB0050995	TG(20:3(5Z,8Z,11Z)/14:1(9Z)/20:3(5Z,8Z,11Z))	C57H96O6	875.7107486	No
13942	HMDB0051001	TG(20:3(5Z,8Z,11Z)/14:1(9Z)/20:3n6)	C57H96O6	875.7107486	No
13942	HMDB0051017	TG(20:3(5Z,8Z,11Z)/16:1(9Z)/18:3(6Z,9Z,12Z))	C57H96O6	875.7107486	No
13942	HMDB0051024	TG(20:3(5Z,8Z,11Z)/16:1(9Z)/18:3(9Z,12Z,15Z))	C57H96O6	875.7107486	No
13942	HMDB0052403	TG(18:2(9Z,12Z)/14:0/22:5(4Z,7Z,10Z,13Z,16Z))	C57H96O6	875.7107486	No
13942	HMDB0052408	TG(18:2(9Z,12Z)/14:0/22:5(7Z,10Z,13Z,16Z,19Z))	C57H96O6	875.7107486	No
13942	HMDB0052507	TG(18:2(9Z,12Z)/14:1(9Z)/22:4(7Z,10Z,13Z,16Z))	C57H96O6	875.7107486	No
13942	HMDB0052520	TG(18:2(9Z,12Z)/16:1(9Z)/20:4(5Z,8Z,11Z,14Z))	C57H96O6	875.7107486	No
13942	HMDB0052526	TG(18:2(9Z,12Z)/16:1(9Z)/20:4(8Z,11Z,14Z,17Z))	C57H96O6	875.7107486	No
13942	HMDB0052540	TG(18:2(9Z,12Z)/18:1(11Z)/18:4(6Z,9Z,12Z,15Z))	C57H96O6	875.7107486	No
13942	HMDB0052555	TG(18:2(9Z,12Z)/18:1(9Z)/18:4(6Z,9Z,12Z,15Z))	C57H96O6	875.7107486	No
13942	HMDB0052636	TG(18:2(9Z,12Z)/18:3(6Z,9Z,12Z)/18:2(9Z,12Z))	C57H96O6	875.7107486	No
13942	HMDB0052741	TG(18:2(9Z,12Z)/18:3(9Z,12Z,15Z)/18:2(9Z,12Z))	C57H96O6	875.7107486	No
13942	HMDB0052850	TG(18:3(6Z,9Z,12Z)/14:0/22:4(7Z,10Z,13Z,16Z))	C57H96O6	875.7107486	No
13942	HMDB0052876	TG(18:3(6Z,9Z,12Z)/16:0/20:4(5Z,8Z,11Z,14Z))	C57H96O6	875.7107486	No
13942	HMDB0052882	TG(18:3(6Z,9Z,12Z)/16:0/20:4(8Z,11Z,14Z,17Z))	C57H96O6	875.7107486	No
13942	HMDB0052895	TG(18:3(6Z,9Z,12Z)/18:0/18:4(6Z,9Z,12Z,15Z))	C57H96O6	875.7107486	No
13942	HMDB0052959	TG(18:3(6Z,9Z,12Z)/16:1(9Z)/20:3n6)	C57H96O6	875.7107486	No
13942	HMDB0052971	TG(18:3(6Z,9Z,12Z)/18:1(11Z)/18:3(6Z,9Z,12Z))	C57H96O6	875.7107486	No
13942	HMDB0052978	TG(18:3(6Z,9Z,12Z)/18:1(11Z)/18:3(9Z,12Z,15Z))	C57H96O6	875.7107486	No
13942	HMDB0052985	TG(18:3(6Z,9Z,12Z)/18:1(9Z)/18:3(6Z,9Z,12Z))	C57H96O6	875.7107486	No
13942	HMDB0052992	TG(18:3(6Z,9Z,12Z)/18:1(9Z)/18:3(9Z,12Z,15Z))	C57H96O6	875.7107486	No
13942	HMDB0053273	TG(20:2n6/14:0/20:5(5Z,8Z,11Z,14Z,17Z))	C57H96O6	875.7107486	No
13942	HMDB0053357	TG(20:2n6/14:1(9Z)/20:4(5Z,8Z,11Z,14Z))	C57H96O6	875.7107486	No
13942	HMDB0053363	TG(20:2n6/14:1(9Z)/20:4(8Z,11Z,14Z,17Z))	C57H96O6	875.7107486	No
13942	HMDB0053375	TG(20:2n6/16:1(9Z)/18:4(6Z,9Z,12Z,15Z))	C57H96O6	875.7107486	No
13942	HMDB0053654	TG(20:3n6/14:0/20:4(5Z,8Z,11Z,14Z))	C57H96O6	875.7107486	No
13942	HMDB0053660	TG(20:3n6/14:0/20:4(8Z,11Z,14Z,17Z))	C57H96O6	875.7107486	No
13942	HMDB0053683	TG(20:3n6/16:0/18:4(6Z,9Z,12Z,15Z))	C57H96O6	875.7107486	No
13942	HMDB0053737	TG(20:3n6/14:1(9Z)/20:3n6)	C57H96O6	875.7107486	No
13942	HMDB0053754	TG(20:3n6/16:1(9Z)/18:3(9Z,12Z,15Z))	C57H96O6	875.7107486	No
13942	HMDB0054415	TG(22:2(13Z,16Z)/14:1(9Z)/18:4(6Z,9Z,12Z,15Z))	C57H96O6	875.7107486	No
13942	HMDB0054642	TG(22:4(7Z,10Z,13Z,16Z)/14:0/18:3(9Z,12Z,15Z))	C57H96O6	875.7107486	No
13942	HMDB0055164	TG(18:3(9Z,12Z,15Z)/16:0/20:4(8Z,11Z,14Z,17Z))	C57H96O6	875.7107486	No
13942	HMDB0055170	TG(18:3(9Z,12Z,15Z)/18:0/18:4(6Z,9Z,12Z,15Z))	C57H96O6	875.7107486	No
14042	HMDB0009793; C00626	PI(16:0/22:4(10Z,13Z,16Z,19Z))	C47H83O13P	885.5472326	No
14042	HMDB0009794; C00626	PI(16:0/22:4(7Z,10Z,13Z,16Z))	C47H83O13P	885.5472326	No
14042	HMDB0009815; C00626	PI(18:0/20:4(5Z,8Z,11Z,14Z))	C47H83O13P	885.5472326	No
14042	HMDB0009816; C00626	PI(18:0/20:4(8Z,11Z,14Z,17Z))	C47H83O13P	885.5472326	No
14042	HMDB0009830; C00626	PI(18:1(11Z)/20:3(5Z,8Z,11Z))	C47H83O13P	885.5472326	No
14042	HMDB0009831; C00626	PI(18:1(11Z)/20:3(8Z,11Z,14Z))	C47H83O13P	885.5472326	No
14042	HMDB0009842; C00626	PI(18:1(9Z)/20:3(5Z,8Z,11Z))	C47H83O13P	885.5472326	No
14042	HMDB0009843; C00626	PI(18:1(9Z)/20:3(8Z,11Z,14Z))	C47H83O13P	885.5472326	No
14042	HMDB0009853; C00626	PI(18:2(9Z,12Z)/20:2(11Z,14Z))	C47H83O13P	885.5472326	No
14042	HMDB0009877; C00626	PI(20:2(11Z,14Z)/18:2(9Z,12Z))	C47H83O13P	885.5472326	No
14042	HMDB0009881; C00626	PI(20:3(5Z,8Z,11Z)/18:1(11Z))	C47H83O13P	885.5472326	No
14042	HMDB0009882; C00626	PI(20:3(5Z,8Z,11Z)/18:1(9Z))	C47H83O13P	885.5472326	No
14042	HMDB0009888; C00626	PI(20:3(8Z,11Z,14Z)/18:1(11Z))	C47H83O13P	885.5472326	No
14042	HMDB0009889; C00626	PI(20:3(8Z,11Z,14Z)/18:1(9Z))	C47H83O13P	885.5472326	No
14042	HMDB0009894; C00626	PI(20:4(5Z,8Z,11Z,14Z)/18:0)	C47H83O13P	885.5472326	No
14042	HMDB0009900; C00626	PI(20:4(8Z,11Z,14Z,17Z)/18:0)	C47H83O13P	885.5472326	No
14042	HMDB0009912; C00626	PI(22:4(10Z,13Z,16Z,19Z)/16:0)	C47H83O13P	885.5472326	No
14042	HMDB0009914; C00626	PI(22:4(7Z,10Z,13Z,16Z)/16:0)	C47H83O13P	885.5472326	No
14045	HMDB0041479	Tritriacontyl octacosanoate	C61H122O2	885.9368597	No
14066	HMDB0043365	TG(15:0/22:1(13Z)/o-18:0)	C58H112O5	887.8411858	No
14066	HMDB0043813	TG(15:0/o-18:0/22:1(13Z))	C58H112O5	887.8411858	No
14066	HMDB0051441	TG(22:1(13Z)/15:0/o-18:0)	C58H112O5	887.8411858	No
14148	HMDB0042869	TG(14:0/20:5(5Z,8Z,11Z,14Z,17Z)/22:6(4Z,7Z,10Z,13Z,:	C59H92O6	895.6847073	No

14148	HMDB0042927	TG(14:0/22:6(4Z,7Z,10Z,13Z,16Z,19Z)/20:5(5Z,8Z,11Z,1	C59H92O6	895.6847073	No
14148	HMDB0048182	TG(14:1(9Z)/20:4(5Z,8Z,11Z,14Z)/22:6(4Z,7Z,10Z,13Z,1	C59H92O6	895.6847073	No
14148	HMDB0048249	TG(14:1(9Z)/22:5(4Z,7Z,10Z,13Z,16Z)/20:5(5Z,8Z,11Z,1	C59H92O6	895.6847073	No
14148	HMDB0048320	TG(14:1(9Z)/20:4(8Z,11Z,14Z,17Z)/22:6(4Z,7Z,10Z,13Z	C59H92O6	895.6847073	No
14148	HMDB0048337	TG(14:1(9Z)/20:5(5Z,8Z,11Z,14Z,17Z)/22:5(4Z,7Z,10Z,1	C59H92O6	895.6847073	No
14148	HMDB0048342	TG(14:1(9Z)/20:5(5Z,8Z,11Z,14Z,17Z)/22:5(7Z,10Z,13Z	C59H92O6	895.6847073	No
14148	HMDB0048364	TG(14:1(9Z)/22:5(7Z,10Z,13Z,16Z,19Z)/20:5(5Z,8Z,11Z	C59H92O6	895.6847073	No
14148	HMDB0048380	TG(14:1(9Z)/22:6(4Z,7Z,10Z,13Z,16Z,19Z)/20:4(5Z,8Z,1	C59H92O6	895.6847073	No
14148	HMDB0048386	TG(14:1(9Z)/22:6(4Z,7Z,10Z,13Z,16Z,19Z)/20:4(8Z,11Z	C59H92O6	895.6847073	No
14148	HMDB0048961	TG(16:1(9Z)/18:4(6Z,9Z,12Z,15Z)/22:6(4Z,7Z,10Z,13Z,1	C59H92O6	895.6847073	No
14148	HMDB0049003	TG(16:1(9Z)/20:5(5Z,8Z,11Z,14Z,17Z)/20:5(5Z,8Z,11Z,1	C59H92O6	895.6847073	No
14148	HMDB0049045	TG(16:1(9Z)/22:6(4Z,7Z,10Z,13Z,16Z,19Z)/18:4(6Z,9Z,1	C59H92O6	895.6847073	No
14148	HMDB0051313	TG(20:3(5Z,8Z,11Z)/18:4(6Z,9Z,12Z,15Z)/18:4(6Z,9Z,12	C59H92O6	895.6847073	No
14148	HMDB0052767	TG(18:2(9Z,12Z)/18:4(6Z,9Z,12Z,15Z)/20:5(5Z,8Z,11Z,1	C59H92O6	895.6847073	No
14148	HMDB0052795	TG(18:2(9Z,12Z)/20:5(5Z,8Z,11Z,14Z,17Z)/18:4(6Z,9Z,1	C59H92O6	895.6847073	No
14148	HMDB0053079	TG(18:3(6Z,9Z,12Z)/18:3(6Z,9Z,12Z)/20:5(5Z,8Z,11Z,14	C59H92O6	895.6847073	No
14148	HMDB0053119	TG(18:3(6Z,9Z,12Z)/20:4(5Z,8Z,11Z,14Z)/18:4(6Z,9Z,12	C59H92O6	895.6847073	No
14148	HMDB0053177	TG(18:3(6Z,9Z,12Z)/18:3(9Z,12Z,15Z)/20:5(5Z,8Z,11Z,1	C59H92O6	895.6847073	No
14148	HMDB0053184	TG(18:3(6Z,9Z,12Z)/18:4(6Z,9Z,12Z,15Z)/20:4(5Z,8Z,11	C59H92O6	895.6847073	No
14148	HMDB0053190	TG(18:3(6Z,9Z,12Z)/18:4(6Z,9Z,12Z,15Z)/20:4(8Z,11Z,1	C59H92O6	895.6847073	No
14148	HMDB0053203	TG(18:3(6Z,9Z,12Z)/20:4(8Z,11Z,14Z,17Z)/18:4(6Z,9Z,1	C59H92O6	895.6847073	No
14148	HMDB0053209	TG(18:3(6Z,9Z,12Z)/20:5(5Z,8Z,11Z,14Z,17Z)/18:3(6Z,9	C59H92O6	895.6847073	No
14148	HMDB0053216	TG(18:3(6Z,9Z,12Z)/20:5(5Z,8Z,11Z,14Z,17Z)/18:3(9Z,1	C59H92O6	895.6847073	No
14148	HMDB0053947	TG(20:3n6/18:4(6Z,9Z,12Z,15Z)/18:4(6Z,9Z,12Z,15Z))	C59H92O6	895.6847073	No
14148	HMDB0054098	TG(20:4(5Z,8Z,11Z,14Z)/14:1(9Z)/22:6(4Z,7Z,10Z,13Z,1	C59H92O6	895.6847073	No
14148	HMDB0054193	TG(20:4(5Z,8Z,11Z,14Z)/18:3(6Z,9Z,12Z)/18:4(6Z,9Z,12	C59H92O6	895.6847073	No
14148	HMDB0054270	TG(20:4(5Z,8Z,11Z,14Z)/18:3(9Z,12Z,15Z)/18:4(6Z,9Z,1	C59H92O6	895.6847073	No
14148	HMDB0054280	TG(20:4(5Z,8Z,11Z,14Z)/18:4(6Z,9Z,12Z,15Z)/18:3(9Z,1	C59H92O6	895.6847073	No
14148	HMDB0054969	TG(22:5(4Z,7Z,10Z,13Z,16Z)/14:1(9Z)/20:5(5Z,8Z,11Z,1	C59H92O6	895.6847073	No
14148	HMDB0055263	TG(18:3(9Z,12Z,15Z)/18:3(6Z,9Z,12Z)/20:5(5Z,8Z,11Z,1	C59H92O6	895.6847073	No
14148	HMDB0055282	TG(18:3(9Z,12Z,15Z)/20:4(5Z,8Z,11Z,14Z)/18:4(6Z,9Z,1	C59H92O6	895.6847073	No
14148	HMDB0055312	TG(18:3(9Z,12Z,15Z)/18:3(9Z,12Z,15Z)/20:5(5Z,8Z,11Z	C59H92O6	895.6847073	No
14148	HMDB0055318	TG(18:3(9Z,12Z,15Z)/18:4(6Z,9Z,12Z,15Z)/20:4(8Z,11Z	C59H92O6	895.6847073	No
14148	HMDB0055324	TG(18:3(9Z,12Z,15Z)/20:4(8Z,11Z,14Z,17Z)/18:4(6Z,9Z	C59H92O6	895.6847073	No
14148	HMDB0055330	TG(18:3(9Z,12Z,15Z)/20:5(5Z,8Z,11Z,14Z,17Z)/18:3(9Z	C59H92O6	895.6847073	No
14148	HMDB0055409	TG(18:4(6Z,9Z,12Z,15Z)/16:1(9Z)/22:6(4Z,7Z,10Z,13Z,1	C59H92O6	895.6847073	No
14148	HMDB0055429	TG(18:4(6Z,9Z,12Z,15Z)/20:3(5Z,8Z,11Z)/18:4(6Z,9Z,12	C59H92O6	895.6847073	No
14148	HMDB0055449	TG(18:4(6Z,9Z,12Z,15Z)/18:2(9Z,12Z)/20:5(5Z,8Z,11Z,1	C59H92O6	895.6847073	No
14148	HMDB0055454	TG(18:4(6Z,9Z,12Z,15Z)/18:3(6Z,9Z,12Z)/20:4(8Z,11Z,1	C59H92O6	895.6847073	No
14148	HMDB0055465	TG(18:4(6Z,9Z,12Z,15Z)/20:3n6/18:4(6Z,9Z,12Z,15Z))	C59H92O6	895.6847073	No
14148	HMDB0055496	TG(18:4(6Z,9Z,12Z,15Z)/18:3(9Z,12Z,15Z)/20:4(8Z,11Z	C59H92O6	895.6847073	No
14148	HMDB0055574	TG(20:4(8Z,11Z,14Z,17Z)/14:1(9Z)/22:6(4Z,7Z,10Z,13Z	C59H92O6	895.6847073	No
14148	HMDB0055687	TG(20:5(5Z,8Z,11Z,14Z,17Z)/14:0/22:6(4Z,7Z,10Z,13Z,1	C59H92O6	895.6847073	No
14148	HMDB0055714	TG(20:5(5Z,8Z,11Z,14Z,17Z)/14:1(9Z)/22:5(7Z,10Z,13Z	C59H92O6	895.6847073	No
14148	HMDB0055717	TG(20:5(5Z,8Z,11Z,14Z,17Z)/16:1(9Z)/20:5(5Z,8Z,11Z,1	C59H92O6	895.6847073	No
14165	HMDB0013561	PGP(18:2(9Z,12Z)/22:6(4Z,7Z,10Z,13Z,16Z,19Z))	C46H76O13P2	897.4687362	No
14165	HMDB0013574	PGP(18:3(6Z,9Z,12Z)/22:5(4Z,7Z,10Z,13Z,16Z))	C46H76O13P2	897.4687362	No
14165	HMDB0013575	PGP(18:3(6Z,9Z,12Z)/22:5(7Z,10Z,13Z,16Z,19Z))	C46H76O13P2	897.4687362	No
14165	HMDB0013589	PGP(18:3(9Z,12Z,15Z)/22:5(4Z,7Z,10Z,13Z,16Z))	C46H76O13P2	897.4687362	No
14165	HMDB0013590	PGP(18:3(9Z,12Z,15Z)/22:5(7Z,10Z,13Z,16Z,19Z))	C46H76O13P2	897.4687362	No
14165	HMDB0116464	PGP(20:4(5Z,8Z,11Z,14Z)/20:4(5Z,8Z,11Z,14Z))	C46H76O13P2	897.4687362	No
14165	HMDB0116465	PGP(20:4(5Z,8Z,11Z,14Z)/20:4(8Z,11Z,14Z,17Z))	C46H76O13P2	897.4687362	No
14165	HMDB0116471	PGP(20:4(8Z,11Z,14Z,17Z)/20:4(5Z,8Z,11Z,14Z))	C46H76O13P2	897.4687362	No
14165	HMDB0116472	PGP(20:4(8Z,11Z,14Z,17Z)/20:4(8Z,11Z,14Z,17Z))	C46H76O13P2	897.4687362	No
14165	HMDB0116475	PGP(22:6(4Z,7Z,10Z,13Z,16Z,19Z)/18:2(9Z,12Z))	C46H76O13P2	897.4687362	No
14168	HMDB0010508	TG(18:3(9Z,12Z,15Z)/18:2(9Z,12Z)/20:5(5Z,8Z,11Z,14Z	C59H94O6	897.6990899	No
14168	HMDB0010513	TG(18:3(9Z,12Z,15Z)/20:4(5Z,8Z,11Z,14Z)/18:3(9Z,12Z	C59H94O6	897.6990899	No
14168	HMDB0042659	TG(14:0/20:4(5Z,8Z,11Z,14Z)/22:6(4Z,7Z,10Z,13Z,16Z,1	C59H94O6	897.6990899	No
14168	HMDB0042747	TG(14:0/22:5(4Z,7Z,10Z,13Z,16Z)/20:5(5Z,8Z,11Z,14Z,1	C59H94O6	897.6990899	No
14168	HMDB0042839	TG(14:0/20:4(8Z,11Z,14Z,17Z)/22:6(4Z,7Z,10Z,13Z,16Z	C59H94O6	897.6990899	No
14168	HMDB0042863	TG(14:0/20:5(5Z,8Z,11Z,14Z,17Z)/22:5(4Z,7Z,10Z,13Z,1	C59H94O6	897.6990899	No

14168	HMDB0042868	TG(14:0/20:5(5Z,8Z,11Z,14Z,17Z)/22:5(7Z,10Z,13Z,16Z	C59H94O6	897.6990899	No
14168	HMDB0042897	TG(14:0/22:5(7Z,10Z,13Z,16Z,19Z)/20:5(5Z,8Z,11Z,14Z	C59H94O6	897.6990899	No
14168	HMDB0042920	TG(14:0/22:6(4Z,7Z,10Z,13Z,16Z,19Z)/20:4(5Z,8Z,11Z,1	C59H94O6	897.6990899	No
14168	HMDB0042926	TG(14:0/22:6(4Z,7Z,10Z,13Z,16Z,19Z)/20:4(8Z,11Z,14Z	C59H94O6	897.6990899	No
14168	HMDB0044527	TG(16:0/18:4(6Z,9Z,12Z,15Z)/22:6(4Z,7Z,10Z,13Z,16Z,1	C59H94O6	897.6990899	No
14168	HMDB0044581	TG(16:0/20:5(5Z,8Z,11Z,14Z,17Z)/20:5(5Z,8Z,11Z,14Z,1	C59H94O6	897.6990899	No
14168	HMDB0044635	TG(16:0/22:6(4Z,7Z,10Z,13Z,16Z,19Z)/18:4(6Z,9Z,12Z,1	C59H94O6	897.6990899	No
14168	HMDB0048021	TG(14:1(9Z)/20:3(5Z,8Z,11Z)/22:6(4Z,7Z,10Z,13Z,16Z,1	C59H94O6	897.6990899	No
14168	HMDB0048159	TG(14:1(9Z)/20:3n6/22:6(4Z,7Z,10Z,13Z,16Z,19Z))	C59H94O6	897.6990899	No
14168	HMDB0048176	TG(14:1(9Z)/20:4(5Z,8Z,11Z,14Z)/22:5(4Z,7Z,10Z,13Z,1	C59H94O6	897.6990899	No
14168	HMDB0048181	TG(14:1(9Z)/20:4(5Z,8Z,11Z,14Z)/22:5(7Z,10Z,13Z,16Z	C59H94O6	897.6990899	No
14168	HMDB0048226	TG(14:1(9Z)/22:4(7Z,10Z,13Z,16Z)/20:5(5Z,8Z,11Z,14Z	C59H94O6	897.6990899	No
14168	HMDB0048242	TG(14:1(9Z)/22:5(4Z,7Z,10Z,13Z,16Z)/20:4(5Z,8Z,11Z,1	C59H94O6	897.6990899	No
14168	HMDB0048248	TG(14:1(9Z)/22:5(4Z,7Z,10Z,13Z,16Z)/20:4(8Z,11Z,14Z	C59H94O6	897.6990899	No
14168	HMDB0048314	TG(14:1(9Z)/20:4(8Z,11Z,14Z,17Z)/22:5(4Z,7Z,10Z,13Z	C59H94O6	897.6990899	No
14168	HMDB0048319	TG(14:1(9Z)/20:4(8Z,11Z,14Z,17Z)/22:5(7Z,10Z,13Z,16	C59H94O6	897.6990899	No
14168	HMDB0048336	TG(14:1(9Z)/20:5(5Z,8Z,11Z,14Z,17Z)/22:4(7Z,10Z,13Z	C59H94O6	897.6990899	No
14168	HMDB0048357	TG(14:1(9Z)/22:5(7Z,10Z,13Z,16Z,19Z)/20:4(5Z,8Z,11Z	C59H94O6	897.6990899	No
14168	HMDB0048363	TG(14:1(9Z)/22:5(7Z,10Z,13Z,16Z,19Z)/20:4(8Z,11Z,14	C59H94O6	897.6990899	No
14168	HMDB0048373	TG(14:1(9Z)/22:6(4Z,7Z,10Z,13Z,16Z,19Z)/20:3(5Z,8Z,1	C59H94O6	897.6990899	No
14168	HMDB0048379	TG(14:1(9Z)/22:6(4Z,7Z,10Z,13Z,16Z,19Z)/20:3n6)	C59H94O6	897.6990899	No
14168	HMDB0048785	TG(16:1(9Z)/18:3(6Z,9Z,12Z)/22:6(4Z,7Z,10Z,13Z,16Z,1	C59H94O6	897.6990899	No
14168	HMDB0048849	TG(16:1(9Z)/20:4(5Z,8Z,11Z,14Z)/20:5(5Z,8Z,11Z,14Z,1	C59H94O6	897.6990899	No
14168	HMDB0048913	TG(16:1(9Z)/22:5(4Z,7Z,10Z,13Z,16Z)/18:4(6Z,9Z,12Z,1	C59H94O6	897.6990899	No
14168	HMDB0048939	TG(16:1(9Z)/18:3(9Z,12Z,15Z)/22:6(4Z,7Z,10Z,13Z,16Z	C59H94O6	897.6990899	No
14168	HMDB0048955	TG(16:1(9Z)/18:4(6Z,9Z,12Z,15Z)/22:5(4Z,7Z,10Z,13Z,1	C59H94O6	897.6990899	No
14168	HMDB0048960	TG(16:1(9Z)/18:4(6Z,9Z,12Z,15Z)/22:5(7Z,10Z,13Z,16Z	C59H94O6	897.6990899	No
14168	HMDB0048981	TG(16:1(9Z)/20:4(8Z,11Z,14Z,17Z)/20:5(5Z,8Z,11Z,14Z	C59H94O6	897.6990899	No
14168	HMDB0048996	TG(16:1(9Z)/20:5(5Z,8Z,11Z,14Z,17Z)/20:4(5Z,8Z,11Z,1	C59H94O6	897.6990899	No
14168	HMDB0049002	TG(16:1(9Z)/20:5(5Z,8Z,11Z,14Z,17Z)/20:4(8Z,11Z,14Z	C59H94O6	897.6990899	No
14168	HMDB0049023	TG(16:1(9Z)/22:5(7Z,10Z,13Z,16Z,19Z)/18:4(6Z,9Z,12Z	C59H94O6	897.6990899	No
14168	HMDB0049037	TG(16:1(9Z)/22:6(4Z,7Z,10Z,13Z,16Z,19Z)/18:3(6Z,9Z,1	C59H94O6	897.6990899	No
14168	HMDB0049044	TG(16:1(9Z)/22:6(4Z,7Z,10Z,13Z,16Z,19Z)/18:3(9Z,12Z	C59H94O6	897.6990899	No
14168	HMDB0049593	TG(18:1(11Z)/18:4(6Z,9Z,12Z,15Z)/20:5(5Z,8Z,11Z,14Z	C59H94O6	897.6990899	No
14168	HMDB0049633	TG(18:1(11Z)/20:5(5Z,8Z,11Z,14Z,17Z)/18:4(6Z,9Z,12Z	C59H94O6	897.6990899	No
14168	HMDB0050197	TG(18:1(9Z)/18:4(6Z,9Z,12Z,15Z)/20:5(5Z,8Z,11Z,14Z,1	C59H94O6	897.6990899	No
14168	HMDB0050235	TG(18:1(9Z)/20:5(5Z,8Z,11Z,14Z,17Z)/18:4(6Z,9Z,12Z,1	C59H94O6	897.6990899	No
14168	HMDB0051011	TG(20:3(5Z,8Z,11Z)/14:1(9Z)/22:6(4Z,7Z,10Z,13Z,16Z,1	C59H94O6	897.6990899	No
14168	HMDB0051169	TG(20:3(5Z,8Z,11Z)/18:3(6Z,9Z,12Z)/18:4(6Z,9Z,12Z,15	C59H94O6	897.6990899	No
14168	HMDB0051295	TG(20:3(5Z,8Z,11Z)/18:3(9Z,12Z,15Z)/18:4(6Z,9Z,12Z,1	C59H94O6	897.6990899	No
14168	HMDB0051305	TG(20:3(5Z,8Z,11Z)/18:4(6Z,9Z,12Z,15Z)/18:3(6Z,9Z,12	C59H94O6	897.6990899	No
14168	HMDB0051312	TG(20:3(5Z,8Z,11Z)/18:4(6Z,9Z,12Z,15Z)/18:3(9Z,12Z,1	C59H94O6	897.6990899	No
14168	HMDB0052647	TG(18:2(9Z,12Z)/18:3(6Z,9Z,12Z)/20:5(5Z,8Z,11Z,14Z,1	C59H94O6	897.6990899	No
14168	HMDB0052690	TG(18:2(9Z,12Z)/20:4(5Z,8Z,11Z,14Z)/18:4(6Z,9Z,12Z,1	C59H94O6	897.6990899	No
14168	HMDB0052752	TG(18:2(9Z,12Z)/18:3(9Z,12Z,15Z)/20:5(5Z,8Z,11Z,14Z	C59H94O6	897.6990899	No
14168	HMDB0052760	TG(18:2(9Z,12Z)/18:4(6Z,9Z,12Z,15Z)/20:4(5Z,8Z,11Z,1	C59H94O6	897.6990899	No
14168	HMDB0052766	TG(18:2(9Z,12Z)/18:4(6Z,9Z,12Z,15Z)/20:4(8Z,11Z,14Z	C59H94O6	897.6990899	No
14168	HMDB0052780	TG(18:2(9Z,12Z)/20:4(8Z,11Z,14Z,17Z)/18:4(6Z,9Z,12Z	C59H94O6	897.6990899	No
14168	HMDB0052787	TG(18:2(9Z,12Z)/20:5(5Z,8Z,11Z,14Z,17Z)/18:3(6Z,9Z,1	C59H94O6	897.6990899	No
14168	HMDB0052794	TG(18:2(9Z,12Z)/20:5(5Z,8Z,11Z,14Z,17Z)/18:3(9Z,12Z	C59H94O6	897.6990899	No
14168	HMDB0052969	TG(18:3(6Z,9Z,12Z)/16:1(9Z)/22:6(4Z,7Z,10Z,13Z,16Z,1	C59H94O6	897.6990899	No
14168	HMDB0053021	TG(18:3(6Z,9Z,12Z)/20:3(5Z,8Z,11Z)/18:4(6Z,9Z,12Z,15	C59H94O6	897.6990899	No
14168	HMDB0053065	TG(18:3(6Z,9Z,12Z)/18:2(9Z,12Z)/20:5(5Z,8Z,11Z,14Z,1	C59H94O6	897.6990899	No
14168	HMDB0053072	TG(18:3(6Z,9Z,12Z)/18:3(6Z,9Z,12Z)/20:4(5Z,8Z,11Z,14	C59H94O6	897.6990899	No
14168	HMDB0053078	TG(18:3(6Z,9Z,12Z)/18:3(6Z,9Z,12Z)/20:4(8Z,11Z,14Z,1	C59H94O6	897.6990899	No
14168	HMDB0053105	TG(18:3(6Z,9Z,12Z)/20:3n6/18:4(6Z,9Z,12Z,15Z))	C59H94O6	897.6990899	No
14168	HMDB0053111	TG(18:3(6Z,9Z,12Z)/20:4(5Z,8Z,11Z,14Z)/18:3(6Z,9Z,12	C59H94O6	897.6990899	No
14168	HMDB0053118	TG(18:3(6Z,9Z,12Z)/20:4(5Z,8Z,11Z,14Z)/18:3(9Z,12Z,1	C59H94O6	897.6990899	No
14168	HMDB0053170	TG(18:3(6Z,9Z,12Z)/18:3(9Z,12Z,15Z)/20:4(5Z,8Z,11Z,1	C59H94O6	897.6990899	No
14168	HMDB0053176	TG(18:3(6Z,9Z,12Z)/18:3(9Z,12Z,15Z)/20:4(8Z,11Z,14Z	C59H94O6	897.6990899	No
14168	HMDB0053183	TG(18:3(6Z,9Z,12Z)/18:4(6Z,9Z,12Z,15Z)/20:3n6)	C59H94O6	897.6990899	No

14168	HMDB0053195	TG(18:3(6Z,9Z,12Z)/20:4(8Z,11Z,14Z,17Z)/18:3(6Z,9Z,1	C59H94O6	897.6990899	No
14168	HMDB0053202	TG(18:3(6Z,9Z,12Z)/20:4(8Z,11Z,14Z,17Z)/18:3(9Z,12Z	C59H94O6	897.6990899	No
14168	HMDB0053583	TG(20:2n6/18:4(6Z,9Z,12Z,15Z)/18:4(6Z,9Z,12Z,15Z))	C59H94O6	897.6990899	No
14168	HMDB0053747	TG(20:3n6/14:1(9Z)/22:6(4Z,7Z,10Z,13Z,16Z,19Z))	C59H94O6	897.6990899	No
14168	HMDB0053851	TG(20:3n6/18:3(6Z,9Z,12Z)/18:4(6Z,9Z,12Z,15Z))	C59H94O6	897.6990899	No
14168	HMDB0053935	TG(20:3n6/18:3(9Z,12Z,15Z)/18:4(6Z,9Z,12Z,15Z))	C59H94O6	897.6990899	No
14168	HMDB0053946	TG(20:3n6/18:4(6Z,9Z,12Z,15Z)/18:3(9Z,12Z,15Z))	C59H94O6	897.6990899	No
14168	HMDB0054021	TG(20:4(5Z,8Z,11Z,14Z)/14:0/22:6(4Z,7Z,10Z,13Z,16Z,1	C59H94O6	897.6990899	No
14168	HMDB0054092	TG(20:4(5Z,8Z,11Z,14Z)/14:1(9Z)/22:5(4Z,7Z,10Z,13Z,1	C59H94O6	897.6990899	No
14168	HMDB0054097	TG(20:4(5Z,8Z,11Z,14Z)/14:1(9Z)/22:5(7Z,10Z,13Z,16Z	C59H94O6	897.6990899	No
14168	HMDB0054107	TG(20:4(5Z,8Z,11Z,14Z)/16:1(9Z)/20:5(5Z,8Z,11Z,14Z,1	C59H94O6	897.6990899	No
14168	HMDB0054182	TG(20:4(5Z,8Z,11Z,14Z)/18:2(9Z,12Z)/18:4(6Z,9Z,12Z,1	C59H94O6	897.6990899	No
14168	HMDB0054192	TG(20:4(5Z,8Z,11Z,14Z)/18:3(6Z,9Z,12Z)/18:3(9Z,12Z,1	C59H94O6	897.6990899	No
14168	HMDB0054269	TG(20:4(5Z,8Z,11Z,14Z)/18:3(9Z,12Z,15Z)/18:3(9Z,12Z	C59H94O6	897.6990899	No
14168	HMDB0054708	TG(22:4(7Z,10Z,13Z,16Z)/14:1(9Z)/20:5(5Z,8Z,11Z,14Z	C59H94O6	897.6990899	No
14168	HMDB0054913	TG(22:5(4Z,7Z,10Z,13Z,16Z)/14:0/20:5(5Z,8Z,11Z,14Z,1	C59H94O6	897.6990899	No
14168	HMDB0054968	TG(22:5(4Z,7Z,10Z,13Z,16Z)/14:1(9Z)/20:4(8Z,11Z,14Z	C59H94O6	897.6990899	No
14168	HMDB0054975	TG(22:5(4Z,7Z,10Z,13Z,16Z)/16:1(9Z)/18:4(6Z,9Z,12Z,1	C59H94O6	897.6990899	No
14168	HMDB0055209	TG(18:3(9Z,12Z,15Z)/16:1(9Z)/22:6(4Z,7Z,10Z,13Z,16Z	C59H94O6	897.6990899	No
14168	HMDB0055233	TG(18:3(9Z,12Z,15Z)/20:3(5Z,8Z,11Z)/18:4(6Z,9Z,12Z,1	C59H94O6	897.6990899	No
14168	HMDB0055262	TG(18:3(9Z,12Z,15Z)/18:3(6Z,9Z,12Z)/20:4(8Z,11Z,14Z	C59H94O6	897.6990899	No
14168	HMDB0055275	TG(18:3(9Z,12Z,15Z)/20:3n6/18:4(6Z,9Z,12Z,15Z))	C59H94O6	897.6990899	No
14168	HMDB0055311	TG(18:3(9Z,12Z,15Z)/18:3(9Z,12Z,15Z)/20:4(8Z,11Z,14	C59H94O6	897.6990899	No
14168	HMDB0055323	TG(18:3(9Z,12Z,15Z)/20:4(8Z,11Z,14Z,17Z)/18:3(9Z,12	C59H94O6	897.6990899	No
14168	HMDB0055373	TG(18:4(6Z,9Z,12Z,15Z)/16:0/22:6(4Z,7Z,10Z,13Z,16Z,1	C59H94O6	897.6990899	No
14168	HMDB0055408	TG(18:4(6Z,9Z,12Z,15Z)/16:1(9Z)/22:5(7Z,10Z,13Z,16Z	C59H94O6	897.6990899	No
14168	HMDB0055413	TG(18:4(6Z,9Z,12Z,15Z)/18:1(11Z)/20:5(5Z,8Z,11Z,14Z	C59H94O6	897.6990899	No
14168	HMDB0055419	TG(18:4(6Z,9Z,12Z,15Z)/18:1(9Z)/20:5(5Z,8Z,11Z,14Z,1	C59H94O6	897.6990899	No
14168	HMDB0055448	TG(18:4(6Z,9Z,12Z,15Z)/18:2(9Z,12Z)/20:4(8Z,11Z,14Z	C59H94O6	897.6990899	No
14168	HMDB0055459	TG(18:4(6Z,9Z,12Z,15Z)/20:2n6/18:4(6Z,9Z,12Z,15Z))	C59H94O6	897.6990899	No
14168	HMDB0055539	TG(20:4(8Z,11Z,14Z,17Z)/14:0/22:6(4Z,7Z,10Z,13Z,16Z	C59H94O6	897.6990899	No
14168	HMDB0055573	TG(20:4(8Z,11Z,14Z,17Z)/14:1(9Z)/22:5(7Z,10Z,13Z,16	C59H94O6	897.6990899	No
14168	HMDB0055577	TG(20:4(8Z,11Z,14Z,17Z)/16:1(9Z)/20:5(5Z,8Z,11Z,14Z	C59H94O6	897.6990899	No
14168	HMDB0055686	TG(20:5(5Z,8Z,11Z,14Z,17Z)/14:0/22:5(7Z,10Z,13Z,16Z	C59H94O6	897.6990899	No
14168	HMDB0055693	TG(20:5(5Z,8Z,11Z,14Z,17Z)/16:0/20:5(5Z,8Z,11Z,14Z,1	C59H94O6	897.6990899	No
14205	HMDB0116797	CL(8:0/8:0/8:0/8:0)	C41H78O17P2	903.4640384	No
14268	HMDB0125940	{3-[5-(3,4-dihydroxyphenyl)-6,9,17,19,21-pentahydrox	C45H36O19S	911.1472092	No
14268	HMDB0125941	(3-{4-[5-(3,4-dihydroxyphenyl)-6,9,17,19,21-pentahydr	C45H36O19S	911.1472092	No
14268	HMDB0125943	(3-{18-[2-(3,4-dihydroxyphenyl)-3,5,7-trihydroxy-3,4-d	C45H36O19S	911.1472092	No
14268	HMDB0125944	(2-hydroxy-5-{3,5,7-trihydroxy-4-[6,9,17,19,21-pentah	C45H36O19S	911.1472092	No
14268	HMDB0125945	(3-{18-[2-(3,4-dihydroxyphenyl)-3,5,7-trihydroxy-3,4-d	C45H36O19S	911.1472092	No
14268	HMDB0125946	[2-(3,4-dihydroxyphenyl)-5,7-dihydroxy-4-[6,9,17,19,2	C45H36O19S	911.1472092	No
14268	HMDB0125947	{18-[2-(3,4-dihydroxyphenyl)-3,5,7-trihydroxy-3,4-dihy	C45H36O19S	911.1472092	No
14268	HMDB0125948	{18-[2-(3,4-dihydroxyphenyl)-3,5,7-trihydroxy-3,4-dihy	C45H36O19S	911.1472092	No
14268	HMDB0125950	(3-{4-[13-(3,4-dihydroxyphenyl)-6,9,17,19,21-pentahy	C45H36O19S	911.1472092	No
14268	HMDB0125951	{3-[13-(3,4-dihydroxyphenyl)-6,9,17,19,21-pentahydro	C45H36O19S	911.1472092	No
14271	HMDB0039295	Medicagenic acid 28-O-[b-D-xylosyl-(1->4)-a-L-rhamno	C46H72O18	911.4643155	No
14526	HMDB0044248	TG(16:0/24:1(15Z)/o-18:0)	C61H118O5	929.8898366	No
14526	HMDB0044653	TG(16:0/o-18:0/24:1(15Z))	C61H118O5	929.8898366	No
14526	HMDB0045045	TG(18:0/22:1(13Z)/o-18:0)	C61H118O5	929.8898366	No
14526	HMDB0045461	TG(18:0/o-18:0/22:1(13Z))	C61H118O5	929.8898366	No
14526	HMDB0045788	TG(20:0/20:1(11Z)/o-18:0)	C61H118O5	929.8898366	No
14526	HMDB0046238	TG(20:0/o-18:0/20:1(11Z))	C61H118O5	929.8898366	No
14526	HMDB0046505	TG(22:0/18:1(11Z)/o-18:0)	C61H118O5	929.8898366	No
14526	HMDB0046530	TG(22:0/18:1(9Z)/o-18:0)	C61H118O5	929.8898366	No
14526	HMDB0046985	TG(22:0/o-18:0/18:1(11Z))	C61H118O5	929.8898366	No
14526	HMDB0046986	TG(22:0/o-18:0/18:1(9Z))	C61H118O5	929.8898366	No
14526	HMDB0047220	TG(24:0/16:1(9Z)/o-18:0)	C61H118O5	929.8898366	No
14526	HMDB0047703	TG(24:0/o-18:0/16:1(9Z))	C61H118O5	929.8898366	No
14526	HMDB0048566	TG(16:1(9Z)/24:0/o-18:0)	C61H118O5	929.8898366	No
14526	HMDB0049197	TG(18:1(11Z)/22:0/o-18:0)	C61H118O5	929.8898366	No

14526	HMDB0049820	TG(18:1(9Z)/22:0/o-18:0)	C61H118O5	929.8898366	No
14526	HMDB0050394	TG(20:1(11Z)/20:0/o-18:0)	C61H118O5	929.8898366	No
14526	HMDB0051475	TG(22:1(13Z)/18:0/o-18:0)	C61H118O5	929.8898366	No
14526	HMDB0051964	TG(24:1(15Z)/16:0/o-18:0)	C61H118O5	929.8898366	No
14623	HMDB0006403; C12450	Pseudoecgonyl-CoA	C30H49N8O18P3S	933.204308	No
14646	HMDB0003939; C05265; CHEBI:28528	3-Oxodecanoyl-CoA	C31H52N7O18P3S	934.2240308	No
14646	HMDB0004601; C11947	3-Hydroxy-2,6-dimethyl-5-methylene-heptanoyl-CoA	C31H52N7O18P3S	934.2240308	No
14682	HMDB0003938; C05264; CHEBI:28325	(S)-Hydroxydecanoyl-CoA	C31H54N7O18P3S	936.236238	No
14706	HMDB0029950	Punicafolin	C41H30O26	937.0934359	No
14706	HMDB0039265; C10224	Tellimagrandin II	C41H30O26	937.0934359	No
14838	HMDB0043567	TG(15:0/22:2(13Z,16Z)/22:6(4Z,7Z,10Z,13Z,16Z,19Z))	C62H104O6	943.7773583	No
14838	HMDB0043589	TG(15:0/22:4(7Z,10Z,13Z,16Z)/22:4(7Z,10Z,13Z,16Z))	C62H104O6	943.7773583	No
14838	HMDB0043791	TG(15:0/22:6(4Z,7Z,10Z,13Z,16Z,19Z)/22:2(13Z,16Z))	C62H104O6	943.7773583	No
14838	HMDB0054359	TG(22:2(13Z,16Z)/15:0/22:6(4Z,7Z,10Z,13Z,16Z,19Z))	C62H104O6	943.7773583	No
14838	HMDB0054649	TG(22:4(7Z,10Z,13Z,16Z)/15:0/22:4(7Z,10Z,13Z,16Z))	C62H104O6	943.7773583	No
14899	HMDB0001380	Diguanosine pentaphosphate	C20H29N10O24P5	946.9994439	No
14924	HMDB0060160	3-Oxo-4(R),8-dimethyl-nonanoyl-CoA	C32H54N7O18P3S	948.238553	No
14933	HMDB0113796	PE-NMe(13D5/13D5)	C54H96NO10P	948.670929	No
14933	HMDB0114701	PE-NMe2(13D5/13M5)	C54H96NO10P	948.670929	No
14933	HMDB0114709	PE-NMe2(13M5/13D5)	C54H96NO10P	948.670929	No
15056	HMDB0012223; C09818; CHEBI:27639	E-Phenylitaconyl-CoA	C32H44N7O19P3S	954.1576039	No
15086	HMDB0046022	TG(20:0/22:2(13Z,16Z)/o-18:0)	C63H120O5	955.9062061	No
15086	HMDB0046247	TG(20:0/o-18:0/22:2(13Z,16Z))	C63H120O5	955.9062061	No
15086	HMDB0046705	TG(22:0/20:2n6/o-18:0)	C63H120O5	955.9062061	No
15086	HMDB0046993	TG(22:0/o-18:0/20:2n6)	C63H120O5	955.9062061	No
15086	HMDB0047388	TG(24:0/18:2(9Z,12Z)/o-18:0)	C63H120O5	955.9062061	No
15086	HMDB0047710	TG(24:0/o-18:0/18:2(9Z,12Z))	C63H120O5	955.9062061	No
15086	HMDB0049386	TG(18:1(11Z)/24:1(15Z)/o-18:0)	C63H120O5	955.9062061	No
15086	HMDB0049686	TG(18:1(11Z)/o-18:0/24:1(15Z))	C63H120O5	955.9062061	No
15086	HMDB0050000	TG(18:1(9Z)/24:1(15Z)/o-18:0)	C63H120O5	955.9062061	No
15086	HMDB0050285	TG(18:1(9Z)/o-18:0/24:1(15Z))	C63H120O5	955.9062061	No
15086	HMDB0050565	TG(20:1(11Z)/22:1(13Z)/o-18:0)	C63H120O5	955.9062061	No
15086	HMDB0050853	TG(20:1(11Z)/o-18:0/22:1(13Z))	C63H120O5	955.9062061	No
15086	HMDB0051611	TG(22:1(13Z)/20:1(11Z)/o-18:0)	C63H120O5	955.9062061	No
15086	HMDB0052076	TG(24:1(15Z)/18:1(11Z)/o-18:0)	C63H120O5	955.9062061	No
15086	HMDB0052092	TG(24:1(15Z)/18:1(9Z)/o-18:0)	C63H120O5	955.9062061	No
15086	HMDB0052500	TG(18:2(9Z,12Z)/24:0/o-18:0)	C63H120O5	955.9062061	No
15086	HMDB0053341	TG(20:2n6/22:0/o-18:0)	C63H120O5	955.9062061	No
15086	HMDB0054390	TG(22:2(13Z,16Z)/20:0/o-18:0)	C63H120O5	955.9062061	No
15192	HMDB0135051	6-(3-{6,8-bis[3,7-dihydroxy-2-(3-hydroxyphenyl)-3,4-di	C51H46O19	961.2556929	No
15192	HMDB0135052	6-[3-(4-{6-[3,7-dihydroxy-2-(3-hydroxyphenyl)-3,4-dihy	C51H46O19	961.2556929	No
15192	HMDB0135053	6-({6,8-bis[3,7-dihydroxy-2-(3-hydroxyphenyl)-3,4-dihy	C51H46O19	961.2556929	No
15192	HMDB0135054	6-[3-(4-{8-[3,7-dihydroxy-2-(3-hydroxyphenyl)-3,4-dihy	C51H46O19	961.2556929	No
15210	HMDB0116048	CDP-DG(18:2(9Z,11Z)/a-15:0)	C45H79N3O15P2	962.4940439	No
15210	HMDB0116055	CDP-DG(18:2(9Z,11Z)/i-15:0)	C45H79N3O15P2	962.4940439	No
15210	HMDB0116080	CDP-DG(a-15:0/18:2(9Z,11Z))	C45H79N3O15P2	962.4940439	No
15210	HMDB0116205	CDP-DG(i-15:0/18:2(9Z,11Z))	C45H79N3O15P2	962.4940439	No
15225	HMDB0030415	Quercetin 3-(6''''''''-feruloylglucosyl)-(1->2)-galactosyl-(	C43H48O25	963.2438771	No
15225	HMDB0031585	Patuletin 3-(2''''-apiosyl-[2'''''-feruloylgentiobioside])	C43H48O25	963.2438771	No
15225	HMDB0040842	Quercetin 3-(2-feruloylsophoroside) 7-glucoside	C43H48O25	963.2438771	No
15242	HMDB0003936; C05262; CHEBI:27668	(S)-3-Hydroxydodecanoyl-CoA	C33H58N7O18P3S	964.2723751	No
15242	HMDB0062260	3S-hydroxydodecanoyl-CoA	C33H58N7O18P3S	964.2723751	No
15267	HMDB0005416; C00422	TG(20:0/20:0/20:4(5Z,8Z,11Z,14Z))	C63H114O6	965.8569699	No
15267	HMDB0042262	TG(14:0/24:0/22:4(7Z,10Z,13Z,16Z))	C63H114O6	965.8569699	No
15267	HMDB0042697	TG(14:0/22:4(7Z,10Z,13Z,16Z)/24:0)	C63H114O6	965.8569699	No
15267	HMDB0043988	TG(16:0/22:0/22:4(7Z,10Z,13Z,16Z))	C63H114O6	965.8569699	No
15267	HMDB0044014	TG(16:0/24:0/20:4(5Z,8Z,11Z,14Z))	C63H114O6	965.8569699	No
15267	HMDB0044020	TG(16:0/24:0/20:4(8Z,11Z,14Z,17Z))	C63H114O6	965.8569699	No
15267	HMDB0044177	TG(16:0/20:3(5Z,8Z,11Z)/24:1(15Z))	C63H114O6	965.8569699	No
15267	HMDB0044231	TG(16:0/24:1(15Z)/20:3(5Z,8Z,11Z))	C63H114O6	965.8569699	No
15267	HMDB0044237	TG(16:0/24:1(15Z)/20:3n6)	C63H114O6	965.8569699	No

15267	HMDB0044345	TG(16:0/20:3n6/24:1(15Z))	C63H114O6	965.8569699	No
15267	HMDB0044365	TG(16:0/20:4(5Z,8Z,11Z,14Z)/24:0)	C63H114O6	965.8569699	No
15267	HMDB0044407	TG(16:0/22:2(13Z,16Z)/22:2(13Z,16Z))	C63H114O6	965.8569699	No
15267	HMDB0044420	TG(16:0/22:4(7Z,10Z,13Z,16Z)/22:0)	C63H114O6	965.8569699	No
15267	HMDB0044533	TG(16:0/20:4(8Z,11Z,14Z,17Z)/24:0)	C63H114O6	965.8569699	No
15267	HMDB0044794	TG(18:0/20:0/22:4(7Z,10Z,13Z,16Z))	C63H114O6	965.8569699	No
15267	HMDB0044819	TG(18:0/22:0/20:4(5Z,8Z,11Z,14Z))	C63H114O6	965.8569699	No
15267	HMDB0044825	TG(18:0/22:0/20:4(8Z,11Z,14Z,17Z))	C63H114O6	965.8569699	No
15267	HMDB0044851	TG(18:0/24:0/18:4(6Z,9Z,12Z,15Z))	C63H114O6	965.8569699	No
15267	HMDB0045002	TG(18:0/20:3(5Z,8Z,11Z)/22:1(13Z))	C63H114O6	965.8569699	No
15267	HMDB0045028	TG(18:0/22:1(13Z)/20:3(5Z,8Z,11Z))	C63H114O6	965.8569699	No
15267	HMDB0045034	TG(18:0/22:1(13Z)/20:3n6)	C63H114O6	965.8569699	No
15267	HMDB0045059	TG(18:0/24:1(15Z)/18:3(6Z,9Z,12Z))	C63H114O6	965.8569699	No
15267	HMDB0045066	TG(18:0/24:1(15Z)/18:3(9Z,12Z,15Z))	C63H114O6	965.8569699	No
15267	HMDB0045111	TG(18:0/18:3(6Z,9Z,12Z)/24:1(15Z))	C63H114O6	965.8569699	No
15267	HMDB0045144	TG(18:0/20:2n6/22:2(13Z,16Z))	C63H114O6	965.8569699	No
15267	HMDB0045164	TG(18:0/20:3n6/22:1(13Z))	C63H114O6	965.8569699	No
15267	HMDB0045183	TG(18:0/20:4(5Z,8Z,11Z,14Z)/22:0)	C63H114O6	965.8569699	No
15267	HMDB0045222	TG(18:0/22:2(13Z,16Z)/20:2n6)	C63H114O6	965.8569699	No
15267	HMDB0045236	TG(18:0/22:4(7Z,10Z,13Z,16Z)/20:0)	C63H114O6	965.8569699	No
15267	HMDB0045300	TG(18:0/18:3(9Z,12Z,15Z)/24:1(15Z))	C63H114O6	965.8569699	No
15267	HMDB0045319	TG(18:0/18:4(6Z,9Z,12Z,15Z)/24:0)	C63H114O6	965.8569699	No
15267	HMDB0045345	TG(18:0/20:4(8Z,11Z,14Z,17Z)/22:0)	C63H114O6	965.8569699	No
15267	HMDB0045572	TG(20:0/18:0/22:4(7Z,10Z,13Z,16Z))	C63H114O6	965.8569699	No
15267	HMDB0045602	TG(20:0/20:0/20:4(8Z,11Z,14Z,17Z))	C63H114O6	965.8569699	No
15267	HMDB0045627	TG(20:0/22:0/18:4(6Z,9Z,12Z,15Z))	C63H114O6	965.8569699	No
15267	HMDB0045771	TG(20:0/20:1(11Z)/20:3(5Z,8Z,11Z))	C63H114O6	965.8569699	No
15267	HMDB0045777	TG(20:0/20:1(11Z)/20:3n6)	C63H114O6	965.8569699	No
15267	HMDB0045796	TG(20:0/20:3(5Z,8Z,11Z)/20:1(11Z))	C63H114O6	965.8569699	No
15267	HMDB0045827	TG(20:0/22:1(13Z)/18:3(6Z,9Z,12Z))	C63H114O6	965.8569699	No
15267	HMDB0045834	TG(20:0/22:1(13Z)/18:3(9Z,12Z,15Z))	C63H114O6	965.8569699	No
15267	HMDB0045883	TG(20:0/18:2(9Z,12Z)/22:2(13Z,16Z))	C63H114O6	965.8569699	No
15267	HMDB0045902	TG(20:0/18:3(6Z,9Z,12Z)/22:1(13Z))	C63H114O6	965.8569699	No
15267	HMDB0045932	TG(20:0/20:2n6/20:2n6)	C63H114O6	965.8569699	No
15267	HMDB0045952	TG(20:0/20:3n6/20:1(11Z))	C63H114O6	965.8569699	No
15267	HMDB0045971	TG(20:0/20:4(5Z,8Z,11Z,14Z)/20:0)	C63H114O6	965.8569699	No
15267	HMDB0046008	TG(20:0/22:2(13Z,16Z)/18:2(9Z,12Z))	C63H114O6	965.8569699	No
15267	HMDB0046084	TG(20:0/18:3(9Z,12Z,15Z)/22:1(13Z))	C63H114O6	965.8569699	No
15267	HMDB0046102	TG(20:0/18:4(6Z,9Z,12Z,15Z)/22:0)	C63H114O6	965.8569699	No
15267	HMDB0046127	TG(20:0/20:4(8Z,11Z,14Z,17Z)/20:0)	C63H114O6	965.8569699	No
15267	HMDB0046322	TG(22:0/16:0/22:4(7Z,10Z,13Z,16Z))	C63H114O6	965.8569699	No
15267	HMDB0046345	TG(22:0/18:0/20:4(5Z,8Z,11Z,14Z))	C63H114O6	965.8569699	No
15267	HMDB0046351	TG(22:0/18:0/20:4(8Z,11Z,14Z,17Z))	C63H114O6	965.8569699	No
15267	HMDB0046375	TG(22:0/20:0/18:4(6Z,9Z,12Z,15Z))	C63H114O6	965.8569699	No
15267	HMDB0046488	TG(22:0/18:1(11Z)/20:3(5Z,8Z,11Z))	C63H114O6	965.8569699	No
15267	HMDB0046494	TG(22:0/18:1(11Z)/20:3n6)	C63H114O6	965.8569699	No
15267	HMDB0046513	TG(22:0/18:1(9Z)/20:3(5Z,8Z,11Z))	C63H114O6	965.8569699	No
15267	HMDB0046519	TG(22:0/18:1(9Z)/20:3n6)	C63H114O6	965.8569699	No
15267	HMDB0046542	TG(22:0/20:1(11Z)/18:3(6Z,9Z,12Z))	C63H114O6	965.8569699	No
15267	HMDB0046549	TG(22:0/20:1(11Z)/18:3(9Z,12Z,15Z))	C63H114O6	965.8569699	No
15267	HMDB0046560	TG(22:0/20:3(5Z,8Z,11Z)/18:1(11Z))	C63H114O6	965.8569699	No
15267	HMDB0046561	TG(22:0/20:3(5Z,8Z,11Z)/18:1(9Z))	C63H114O6	965.8569699	No
15267	HMDB0046643	TG(22:0/18:2(9Z,12Z)/20:2n6)	C63H114O6	965.8569699	No
15267	HMDB0046662	TG(22:0/18:3(6Z,9Z,12Z)/20:1(11Z))	C63H114O6	965.8569699	No
15267	HMDB0046691	TG(22:0/20:2n6/18:2(9Z,12Z))	C63H114O6	965.8569699	No
15267	HMDB0046710	TG(22:0/20:3n6/18:1(11Z))	C63H114O6	965.8569699	No
15267	HMDB0046711	TG(22:0/20:3n6/18:1(9Z))	C63H114O6	965.8569699	No
15267	HMDB0046837	TG(22:0/18:3(9Z,12Z,15Z)/20:1(11Z))	C63H114O6	965.8569699	No
15267	HMDB0047020	TG(24:0/14:0/22:4(7Z,10Z,13Z,16Z))	C63H114O6	965.8569699	No
15267	HMDB0047066	TG(24:0/16:0/20:4(5Z,8Z,11Z,14Z))	C63H114O6	965.8569699	No
15267	HMDB0047072	TG(24:0/16:0/20:4(8Z,11Z,14Z,17Z))	C63H114O6	965.8569699	No

15267	HMDB0047095	TG(24:0/18:0/18:4(6Z,9Z,12Z,15Z))	C63H114O6	965.8569699	No
15267	HMDB0047203	TG(24:0/16:1(9Z)/20:3(5Z,8Z,11Z))	C63H114O6	965.8569699	No
15267	HMDB0047209	TG(24:0/16:1(9Z)/20:3n6)	C63H114O6	965.8569699	No
15267	HMDB0047231	TG(24:0/18:1(11Z)/18:3(6Z,9Z,12Z))	C63H114O6	965.8569699	No
15267	HMDB0047238	TG(24:0/18:1(11Z)/18:3(9Z,12Z,15Z))	C63H114O6	965.8569699	No
15267	HMDB0047255	TG(24:0/18:1(9Z)/18:3(6Z,9Z,12Z))	C63H114O6	965.8569699	No
15267	HMDB0047262	TG(24:0/18:1(9Z)/18:3(9Z,12Z,15Z))	C63H114O6	965.8569699	No
15267	HMDB0047295	TG(24:0/20:3(5Z,8Z,11Z)/16:1(9Z))	C63H114O6	965.8569699	No
15267	HMDB0047374	TG(24:0/18:2(9Z,12Z)/18:2(9Z,12Z))	C63H114O6	965.8569699	No
15267	HMDB0047392	TG(24:0/18:3(6Z,9Z,12Z)/18:1(11Z))	C63H114O6	965.8569699	No
15267	HMDB0047393	TG(24:0/18:3(6Z,9Z,12Z)/18:1(9Z))	C63H114O6	965.8569699	No
15267	HMDB0047439	TG(24:0/20:3n6/16:1(9Z))	C63H114O6	965.8569699	No
15267	HMDB0047560	TG(24:0/18:3(9Z,12Z,15Z)/18:1(11Z))	C63H114O6	965.8569699	No
15267	HMDB0047561	TG(24:0/18:3(9Z,12Z,15Z)/18:1(9Z))	C63H114O6	965.8569699	No
15267	HMDB0048059	TG(14:1(9Z)/24:1(15Z)/22:2(13Z,16Z))	C63H114O6	965.8569699	No
15267	HMDB0048191	TG(14:1(9Z)/22:2(13Z,16Z)/24:1(15Z))	C63H114O6	965.8569699	No
15267	HMDB0048549	TG(16:1(9Z)/24:0/20:3(5Z,8Z,11Z))	C63H114O6	965.8569699	No
15267	HMDB0048555	TG(16:1(9Z)/24:0/20:3n6)	C63H114O6	965.8569699	No
15267	HMDB0048711	TG(16:1(9Z)/22:1(13Z)/22:2(13Z,16Z))	C63H114O6	965.8569699	No
15267	HMDB0048730	TG(16:1(9Z)/24:1(15Z)/20:2n6)	C63H114O6	965.8569699	No
15267	HMDB0048793	TG(16:1(9Z)/20:2n6/24:1(15Z))	C63H114O6	965.8569699	No
15267	HMDB0048858	TG(16:1(9Z)/22:2(13Z,16Z)/22:1(13Z))	C63H114O6	965.8569699	No
15267	HMDB0049180	TG(18:1(11Z)/22:0/20:3(5Z,8Z,11Z))	C63H114O6	965.8569699	No
15267	HMDB0049186	TG(18:1(11Z)/22:0/20:3n6)	C63H114O6	965.8569699	No
15267	HMDB0049205	TG(18:1(11Z)/24:0/18:3(6Z,9Z,12Z))	C63H114O6	965.8569699	No
15267	HMDB0049212	TG(18:1(11Z)/24:0/18:3(9Z,12Z,15Z))	C63H114O6	965.8569699	No
15267	HMDB0049314	TG(18:1(11Z)/20:1(11Z)/22:2(13Z,16Z))	C63H114O6	965.8569699	No
15267	HMDB0049353	TG(18:1(11Z)/22:1(13Z)/20:2n6)	C63H114O6	965.8569699	No
15267	HMDB0049372	TG(18:1(11Z)/24:1(15Z)/18:2(9Z,12Z))	C63H114O6	965.8569699	No
15267	HMDB0049392	TG(18:1(11Z)/18:2(9Z,12Z)/24:1(15Z))	C63H114O6	965.8569699	No
15267	HMDB0049433	TG(18:1(11Z)/20:2n6/22:1(13Z))	C63H114O6	965.8569699	No
15267	HMDB0049494	TG(18:1(11Z)/22:2(13Z,16Z)/20:1(11Z))	C63H114O6	965.8569699	No
15267	HMDB0049803	TG(18:1(9Z)/22:0/20:3(5Z,8Z,11Z))	C63H114O6	965.8569699	No
15267	HMDB0049809	TG(18:1(9Z)/22:0/20:3n6)	C63H114O6	965.8569699	No
15267	HMDB0049827	TG(18:1(9Z)/24:0/18:3(6Z,9Z,12Z))	C63H114O6	965.8569699	No
15267	HMDB0049834	TG(18:1(9Z)/24:0/18:3(9Z,12Z,15Z))	C63H114O6	965.8569699	No
15267	HMDB0049931	TG(18:1(9Z)/20:1(11Z)/22:2(13Z,16Z))	C63H114O6	965.8569699	No
15267	HMDB0049968	TG(18:1(9Z)/22:1(13Z)/20:2n6)	C63H114O6	965.8569699	No
15267	HMDB0049986	TG(18:1(9Z)/24:1(15Z)/18:2(9Z,12Z))	C63H114O6	965.8569699	No
15267	HMDB0050005	TG(18:1(9Z)/18:2(9Z,12Z)/24:1(15Z))	C63H114O6	965.8569699	No
15267	HMDB0050044	TG(18:1(9Z)/20:2n6/22:1(13Z))	C63H114O6	965.8569699	No
15267	HMDB0050102	TG(18:1(9Z)/22:2(13Z,16Z)/20:1(11Z))	C63H114O6	965.8569699	No
15267	HMDB0050377	TG(20:1(11Z)/20:0/20:3(5Z,8Z,11Z))	C63H114O6	965.8569699	No
15267	HMDB0050383	TG(20:1(11Z)/20:0/20:3n6)	C63H114O6	965.8569699	No
15267	HMDB0050400	TG(20:1(11Z)/22:0/18:3(6Z,9Z,12Z))	C63H114O6	965.8569699	No
15267	HMDB0050407	TG(20:1(11Z)/22:0/18:3(9Z,12Z,15Z))	C63H114O6	965.8569699	No
15267	HMDB0050480	TG(20:1(11Z)/18:1(11Z)/22:2(13Z,16Z))	C63H114O6	965.8569699	No
15267	HMDB0050499	TG(20:1(11Z)/18:1(9Z)/22:2(13Z,16Z))	C63H114O6	965.8569699	No
15267	HMDB0050515	TG(20:1(11Z)/20:1(11Z)/20:2n6)	C63H114O6	965.8569699	No
15267	HMDB0050551	TG(20:1(11Z)/22:1(13Z)/18:2(9Z,12Z))	C63H114O6	965.8569699	No
15267	HMDB0050587	TG(20:1(11Z)/18:2(9Z,12Z)/22:1(13Z))	C63H114O6	965.8569699	No
15267	HMDB0050623	TG(20:1(11Z)/20:2n6/20:1(11Z))	C63H114O6	965.8569699	No
15267	HMDB0050907	TG(20:3(5Z,8Z,11Z)/16:0/24:1(15Z))	C63H114O6	965.8569699	No
15267	HMDB0050924	TG(20:3(5Z,8Z,11Z)/18:0/22:1(13Z))	C63H114O6	965.8569699	No
15267	HMDB0051464	TG(22:1(13Z)/18:0/20:3n6)	C63H114O6	965.8569699	No
15267	HMDB0051479	TG(22:1(13Z)/20:0/18:3(6Z,9Z,12Z))	C63H114O6	965.8569699	No
15267	HMDB0051486	TG(22:1(13Z)/20:0/18:3(9Z,12Z,15Z))	C63H114O6	965.8569699	No
15267	HMDB0051551	TG(22:1(13Z)/16:1(9Z)/22:2(13Z,16Z))	C63H114O6	965.8569699	No
15267	HMDB0051565	TG(22:1(13Z)/18:1(11Z)/20:2n6)	C63H114O6	965.8569699	No
15267	HMDB0051582	TG(22:1(13Z)/18:1(9Z)/20:2n6)	C63H114O6	965.8569699	No
15267	HMDB0051597	TG(22:1(13Z)/20:1(11Z)/18:2(9Z,12Z))	C63H114O6	965.8569699	No

15267	HMDB0051953	TG(24:1(15Z)/16:0/20:3n6)	C63H114O6	965.8569699	No
15267	HMDB0051967	TG(24:1(15Z)/18:0/18:3(6Z,9Z,12Z))	C63H114O6	965.8569699	No
15267	HMDB0051974	TG(24:1(15Z)/18:0/18:3(9Z,12Z,15Z))	C63H114O6	965.8569699	No
15267	HMDB0052035	TG(24:1(15Z)/14:1(9Z)/22:2(13Z,16Z))	C63H114O6	965.8569699	No
15267	HMDB0052048	TG(24:1(15Z)/16:1(9Z)/20:2n6)	C63H114O6	965.8569699	No
15267	HMDB0052062	TG(24:1(15Z)/18:1(11Z)/18:2(9Z,12Z))	C63H114O6	965.8569699	No
15267	HMDB0052078	TG(24:1(15Z)/18:1(9Z)/18:2(9Z,12Z))	C63H114O6	965.8569699	No
15267	HMDB0052461	TG(18:2(9Z,12Z)/20:0/22:2(13Z,16Z))	C63H114O6	965.8569699	No
15267	HMDB0052473	TG(18:2(9Z,12Z)/22:0/20:2n6)	C63H114O6	965.8569699	No
15267	HMDB0052486	TG(18:2(9Z,12Z)/24:0/18:2(9Z,12Z))	C63H114O6	965.8569699	No
15267	HMDB0053306	TG(20:2n6/18:0/22:2(13Z,16Z))	C63H114O6	965.8569699	No
15267	HMDB0053316	TG(20:2n6/20:0/20:2n6)	C63H114O6	965.8569699	No
15267	HMDB0054361	TG(22:2(13Z,16Z)/16:0/22:2(13Z,16Z))	C63H114O6	965.8569699	No
15297	HMDB0005464; C00422	TG(20:1(11Z)/20:1(11Z)/20:1(11Z))	C63H116O6	967.8709673	No
15297	HMDB0042501	TG(14:0/24:1(15Z)/22:2(13Z,16Z))	C63H116O6	967.8709673	No
15297	HMDB0042675	TG(14:0/22:2(13Z,16Z)/24:1(15Z))	C63H116O6	967.8709673	No
15297	HMDB0044007	TG(16:0/24:0/20:3(5Z,8Z,11Z))	C63H116O6	967.8709673	No
15297	HMDB0044013	TG(16:0/24:0/20:3n6)	C63H116O6	967.8709673	No
15297	HMDB0044169	TG(16:0/20:3(5Z,8Z,11Z)/24:0)	C63H116O6	967.8709673	No
15297	HMDB0044211	TG(16:0/22:1(13Z)/22:2(13Z,16Z))	C63H116O6	967.8709673	No
15297	HMDB0044236	TG(16:0/24:1(15Z)/20:2n6)	C63H116O6	967.8709673	No
15297	HMDB0044317	TG(16:0/20:2n6/24:1(15Z))	C63H116O6	967.8709673	No
15297	HMDB0044337	TG(16:0/20:3n6/24:0)	C63H116O6	967.8709673	No
15297	HMDB0044400	TG(16:0/22:2(13Z,16Z)/22:1(13Z))	C63H116O6	967.8709673	No
15297	HMDB0044812	TG(18:0/22:0/20:3(5Z,8Z,11Z))	C63H116O6	967.8709673	No
15297	HMDB0044818	TG(18:0/22:0/20:3n6)	C63H116O6	967.8709673	No
15297	HMDB0044843	TG(18:0/24:0/18:3(6Z,9Z,12Z))	C63H116O6	967.8709673	No
15297	HMDB0044850	TG(18:0/24:0/18:3(9Z,12Z,15Z))	C63H116O6	967.8709673	No
15297	HMDB0044982	TG(18:0/20:1(11Z)/22:2(13Z,16Z))	C63H116O6	967.8709673	No
15297	HMDB0044994	TG(18:0/20:3(5Z,8Z,11Z)/22:0)	C63H116O6	967.8709673	No
15297	HMDB0045033	TG(18:0/22:1(13Z)/20:2n6)	C63H116O6	967.8709673	No
15297	HMDB0045058	TG(18:0/24:1(15Z)/18:2(9Z,12Z))	C63H116O6	967.8709673	No
15297	HMDB0045084	TG(18:0/18:2(9Z,12Z)/24:1(15Z))	C63H116O6	967.8709673	No
15297	HMDB0045103	TG(18:0/18:3(6Z,9Z,12Z)/24:0)	C63H116O6	967.8709673	No
15297	HMDB0045137	TG(18:0/20:2n6/22:1(13Z))	C63H116O6	967.8709673	No
15297	HMDB0045156	TG(18:0/20:3n6/22:0)	C63H116O6	967.8709673	No
15297	HMDB0045216	TG(18:0/22:2(13Z,16Z)/20:1(11Z))	C63H116O6	967.8709673	No
15297	HMDB0045292	TG(18:0/18:3(9Z,12Z,15Z)/24:0)	C63H116O6	967.8709673	No
15297	HMDB0045589	TG(20:0/20:0/20:3(5Z,8Z,11Z))	C63H116O6	967.8709673	No
15297	HMDB0045595	TG(20:0/20:0/20:3n6)	C63H116O6	967.8709673	No
15297	HMDB0045619	TG(20:0/22:0/18:3(6Z,9Z,12Z))	C63H116O6	967.8709673	No
15297	HMDB0045626	TG(20:0/22:0/18:3(9Z,12Z,15Z))	C63H116O6	967.8709673	No
15297	HMDB0045727	TG(20:0/18:1(11Z)/22:2(13Z,16Z))	C63H116O6	967.8709673	No
15297	HMDB0045753	TG(20:0/18:1(9Z)/22:2(13Z,16Z))	C63H116O6	967.8709673	No
15297	HMDB0045776	TG(20:0/20:1(11Z)/20:2n6)	C63H116O6	967.8709673	No
15297	HMDB0045789	TG(20:0/20:3(5Z,8Z,11Z)/20:0)	C63H116O6	967.8709673	No
15297	HMDB0045826	TG(20:0/22:1(13Z)/18:2(9Z,12Z))	C63H116O6	967.8709673	No
15297	HMDB0045876	TG(20:0/18:2(9Z,12Z)/22:1(13Z))	C63H116O6	967.8709673	No
15297	HMDB0045894	TG(20:0/18:3(6Z,9Z,12Z)/22:0)	C63H116O6	967.8709673	No
15297	HMDB0045926	TG(20:0/20:2n6/20:1(11Z))	C63H116O6	967.8709673	No
15297	HMDB0045945	TG(20:0/20:3n6/20:0)	C63H116O6	967.8709673	No
15297	HMDB0046002	TG(20:0/22:2(13Z,16Z)/18:1(11Z))	C63H116O6	967.8709673	No
15297	HMDB0046003	TG(20:0/22:2(13Z,16Z)/18:1(9Z))	C63H116O6	967.8709673	No
15297	HMDB0046076	TG(20:0/18:3(9Z,12Z,15Z)/22:0)	C63H116O6	967.8709673	No
15297	HMDB0046338	TG(22:0/18:0/20:3(5Z,8Z,11Z))	C63H116O6	967.8709673	No
15297	HMDB0046344	TG(22:0/18:0/20:3n6)	C63H116O6	967.8709673	No
15297	HMDB0046367	TG(22:0/20:0/18:3(6Z,9Z,12Z))	C63H116O6	967.8709673	No
15297	HMDB0046374	TG(22:0/20:0/18:3(9Z,12Z,15Z))	C63H116O6	967.8709673	No
15297	HMDB0046471	TG(22:0/16:1(9Z)/22:2(13Z,16Z))	C63H116O6	967.8709673	No
15297	HMDB0046493	TG(22:0/18:1(11Z)/20:2n6)	C63H116O6	967.8709673	No
15297	HMDB0046518	TG(22:0/18:1(9Z)/20:2n6)	C63H116O6	967.8709673	No

15297	HMDB0046541	TG(22:0/20:1(11Z)/18:2(9Z,12Z))	C63H116O6	967.8709673	No
15297	HMDB0046637	TG(22:0/18:2(9Z,12Z)/20:1(11Z))	C63H116O6	967.8709673	No
15297	HMDB0046685	TG(22:0/20:2n6/18:1(11Z))	C63H116O6	967.8709673	No
15297	HMDB0046686	TG(22:0/20:2n6/18:1(9Z))	C63H116O6	967.8709673	No
15297	HMDB0046759	TG(22:0/22:2(13Z,16Z)/16:1(9Z))	C63H116O6	967.8709673	No
15297	HMDB0047059	TG(24:0/16:0/20:3(5Z,8Z,11Z))	C63H116O6	967.8709673	No
15297	HMDB0047065	TG(24:0/16:0/20:3n6)	C63H116O6	967.8709673	No
15297	HMDB0047087	TG(24:0/18:0/18:3(6Z,9Z,12Z))	C63H116O6	967.8709673	No
15297	HMDB0047094	TG(24:0/18:0/18:3(9Z,12Z,15Z))	C63H116O6	967.8709673	No
15297	HMDB0047187	TG(24:0/14:1(9Z)/22:2(13Z,16Z))	C63H116O6	967.8709673	No
15297	HMDB0047208	TG(24:0/16:1(9Z)/20:2n6)	C63H116O6	967.8709673	No
15297	HMDB0047230	TG(24:0/18:1(11Z)/18:2(9Z,12Z))	C63H116O6	967.8709673	No
15297	HMDB0047254	TG(24:0/18:1(9Z)/18:2(9Z,12Z))	C63H116O6	967.8709673	No
15297	HMDB0047368	TG(24:0/18:2(9Z,12Z)/18:1(11Z))	C63H116O6	967.8709673	No
15297	HMDB0047369	TG(24:0/18:2(9Z,12Z)/18:1(9Z))	C63H116O6	967.8709673	No
15297	HMDB0047415	TG(24:0/20:2n6/16:1(9Z))	C63H116O6	967.8709673	No
15297	HMDB0047486	TG(24:0/22:2(13Z,16Z)/14:1(9Z))	C63H116O6	967.8709673	No
15297	HMDB0047875	TG(14:1(9Z)/24:0/22:2(13Z,16Z))	C63H116O6	967.8709673	No
15297	HMDB0048030	TG(14:1(9Z)/22:1(13Z)/24:1(15Z))	C63H116O6	967.8709673	No
15297	HMDB0048052	TG(14:1(9Z)/24:1(15Z)/22:1(13Z))	C63H116O6	967.8709673	No
15297	HMDB0048535	TG(16:1(9Z)/22:0/22:2(13Z,16Z))	C63H116O6	967.8709673	No
15297	HMDB0048554	TG(16:1(9Z)/24:0/20:2n6)	C63H116O6	967.8709673	No
15297	HMDB0048661	TG(16:1(9Z)/20:1(11Z)/24:1(15Z))	C63H116O6	967.8709673	No
15297	HMDB0048704	TG(16:1(9Z)/22:1(13Z)/22:1(13Z))	C63H116O6	967.8709673	No
15297	HMDB0048724	TG(16:1(9Z)/24:1(15Z)/20:1(11Z))	C63H116O6	967.8709673	No
15297	HMDB0049167	TG(18:1(11Z)/20:0/22:2(13Z,16Z))	C63H116O6	967.8709673	No
15297	HMDB0049185	TG(18:1(11Z)/22:0/20:2n6)	C63H116O6	967.8709673	No
15297	HMDB0049204	TG(18:1(11Z)/24:0/18:2(9Z,12Z))	C63H116O6	967.8709673	No
15297	HMDB0049266	TG(18:1(11Z)/18:1(11Z)/24:1(15Z))	C63H116O6	967.8709673	No
15297	HMDB0049287	TG(18:1(11Z)/18:1(9Z)/24:1(15Z))	C63H116O6	967.8709673	No
15297	HMDB0049307	TG(18:1(11Z)/20:1(11Z)/22:1(13Z))	C63H116O6	967.8709673	No
15297	HMDB0049347	TG(18:1(11Z)/22:1(13Z)/20:1(11Z))	C63H116O6	967.8709673	No
15297	HMDB0049366	TG(18:1(11Z)/24:1(15Z)/18:1(11Z))	C63H116O6	967.8709673	No
15297	HMDB0049367	TG(18:1(11Z)/24:1(15Z)/18:1(9Z))	C63H116O6	967.8709673	No
15297	HMDB0049791	TG(18:1(9Z)/20:0/22:2(13Z,16Z))	C63H116O6	967.8709673	No
15297	HMDB0049808	TG(18:1(9Z)/22:0/20:2n6)	C63H116O6	967.8709673	No
15297	HMDB0049826	TG(18:1(9Z)/24:0/18:2(9Z,12Z))	C63H116O6	967.8709673	No
15297	HMDB0049885	TG(18:1(9Z)/18:1(11Z)/24:1(15Z))	C63H116O6	967.8709673	No
15297	HMDB0049905	TG(18:1(9Z)/18:1(9Z)/24:1(15Z))	C63H116O6	967.8709673	No
15297	HMDB0049924	TG(18:1(9Z)/20:1(11Z)/22:1(13Z))	C63H116O6	967.8709673	No
15297	HMDB0049962	TG(18:1(9Z)/22:1(13Z)/20:1(11Z))	C63H116O6	967.8709673	No
15297	HMDB0049981	TG(18:1(9Z)/24:1(15Z)/18:1(9Z))	C63H116O6	967.8709673	No
15297	HMDB0050366	TG(20:1(11Z)/18:0/22:2(13Z,16Z))	C63H116O6	967.8709673	No
15297	HMDB0050382	TG(20:1(11Z)/20:0/20:2n6)	C63H116O6	967.8709673	No
15297	HMDB0050399	TG(20:1(11Z)/22:0/18:2(9Z,12Z))	C63H116O6	967.8709673	No
15297	HMDB0050455	TG(20:1(11Z)/16:1(9Z)/24:1(15Z))	C63H116O6	967.8709673	No
15297	HMDB0050473	TG(20:1(11Z)/18:1(11Z)/22:1(13Z))	C63H116O6	967.8709673	No
15297	HMDB0050492	TG(20:1(11Z)/18:1(9Z)/22:1(13Z))	C63H116O6	967.8709673	No
15297	HMDB0051449	TG(22:1(13Z)/16:0/22:2(13Z,16Z))	C63H116O6	967.8709673	No
15297	HMDB0051463	TG(22:1(13Z)/18:0/20:2n6)	C63H116O6	967.8709673	No
15297	HMDB0051478	TG(22:1(13Z)/20:0/18:2(9Z,12Z))	C63H116O6	967.8709673	No
15297	HMDB0051528	TG(22:1(13Z)/14:1(9Z)/24:1(15Z))	C63H116O6	967.8709673	No
15297	HMDB0051544	TG(22:1(13Z)/16:1(9Z)/22:1(13Z))	C63H116O6	967.8709673	No
15297	HMDB0051923	TG(24:1(15Z)/14:0/22:2(13Z,16Z))	C63H116O6	967.8709673	No
15297	HMDB0051952	TG(24:1(15Z)/16:0/20:2n6)	C63H116O6	967.8709673	No
15297	HMDB0051966	TG(24:1(15Z)/18:0/18:2(9Z,12Z))	C63H116O6	967.8709673	No
15350	HMDB0005181	Dolichol-14	C70H114O	969.8804122	No
15409	HMDB0044023	TG(16:0/24:0/22:6(4Z,7Z,10Z,13Z,16Z,19Z))	C65H114O6	989.8525339	No
15409	HMDB0044241	TG(16:0/24:1(15Z)/22:5(4Z,7Z,10Z,13Z,16Z))	C65H114O6	989.8525339	No
15409	HMDB0044246	TG(16:0/24:1(15Z)/22:5(7Z,10Z,13Z,16Z,19Z))	C65H114O6	989.8525339	No
15409	HMDB0044457	TG(16:0/22:5(4Z,7Z,10Z,13Z,16Z)/24:1(15Z))	C65H114O6	989.8525339	No

15409	HMDB0044597	TG(16:0/22:5(7Z,10Z,13Z,16Z,19Z)/24:1(15Z))	C65H114O6	989.8525339	No
15409	HMDB0044617	TG(16:0/22:6(4Z,7Z,10Z,13Z,16Z,19Z)/24:0)	C65H114O6	989.8525339	No
15409	HMDB0044828	TG(18:0/22:0/22:6(4Z,7Z,10Z,13Z,16Z,19Z))	C65H114O6	989.8525339	No
15409	HMDB0045038	TG(18:0/22:1(13Z)/22:5(4Z,7Z,10Z,13Z,16Z))	C65H114O6	989.8525339	No
15409	HMDB0045043	TG(18:0/22:1(13Z)/22:5(7Z,10Z,13Z,16Z,19Z))	C65H114O6	989.8525339	No
15409	HMDB0045069	TG(18:0/24:1(15Z)/20:5(5Z,8Z,11Z,14Z,17Z))	C65H114O6	989.8525339	No
15409	HMDB0045226	TG(18:0/22:2(13Z,16Z)/22:4(7Z,10Z,13Z,16Z))	C65H114O6	989.8525339	No
15409	HMDB0045252	TG(18:0/22:4(7Z,10Z,13Z,16Z)/22:2(13Z,16Z))	C65H114O6	989.8525339	No
15409	HMDB0045272	TG(18:0/22:5(4Z,7Z,10Z,13Z,16Z)/22:1(13Z))	C65H114O6	989.8525339	No
15409	HMDB0045381	TG(18:0/20:5(5Z,8Z,11Z,14Z,17Z)/24:1(15Z))	C65H114O6	989.8525339	No
15409	HMDB0045407	TG(18:0/22:5(7Z,10Z,13Z,16Z,19Z)/22:1(13Z))	C65H114O6	989.8525339	No
15409	HMDB0045426	TG(18:0/22:6(4Z,7Z,10Z,13Z,16Z,19Z)/22:0)	C65H114O6	989.8525339	No
15409	HMDB0045605	TG(20:0/20:0/22:6(4Z,7Z,10Z,13Z,16Z,19Z))	C65H114O6	989.8525339	No
15409	HMDB0045781	TG(20:0/20:1(11Z)/22:5(4Z,7Z,10Z,13Z,16Z))	C65H114O6	989.8525339	No
15409	HMDB0045786	TG(20:0/20:1(11Z)/22:5(7Z,10Z,13Z,16Z,19Z))	C65H114O6	989.8525339	No
15409	HMDB0045837	TG(20:0/22:1(13Z)/20:5(5Z,8Z,11Z,14Z,17Z))	C65H114O6	989.8525339	No
15409	HMDB0045936	TG(20:0/20:2n6/22:4(7Z,10Z,13Z,16Z))	C65H114O6	989.8525339	No
15409	HMDB0045987	TG(20:0/20:4(5Z,8Z,11Z,14Z)/22:2(13Z,16Z))	C65H114O6	989.8525339	No
15409	HMDB0046012	TG(20:0/22:2(13Z,16Z)/20:4(5Z,8Z,11Z,14Z))	C65H114O6	989.8525339	No
15409	HMDB0046018	TG(20:0/22:2(13Z,16Z)/20:4(8Z,11Z,14Z,17Z))	C65H114O6	989.8525339	No
15409	HMDB0046036	TG(20:0/22:4(7Z,10Z,13Z,16Z)/20:2n6)	C65H114O6	989.8525339	No
15409	HMDB0046056	TG(20:0/22:5(4Z,7Z,10Z,13Z,16Z)/20:1(11Z))	C65H114O6	989.8525339	No
15409	HMDB0046143	TG(20:0/20:4(8Z,11Z,14Z,17Z)/22:2(13Z,16Z))	C65H114O6	989.8525339	No
15409	HMDB0046162	TG(20:0/20:5(5Z,8Z,11Z,14Z,17Z)/22:1(13Z))	C65H114O6	989.8525339	No
15409	HMDB0046186	TG(20:0/22:5(7Z,10Z,13Z,16Z,19Z)/20:1(11Z))	C65H114O6	989.8525339	No
15409	HMDB0046205	TG(20:0/22:6(4Z,7Z,10Z,13Z,16Z,19Z)/20:0)	C65H114O6	989.8525339	No
15409	HMDB0046354	TG(22:0/18:0/22:6(4Z,7Z,10Z,13Z,16Z,19Z))	C65H114O6	989.8525339	No
15409	HMDB0046498	TG(22:0/18:1(11Z)/22:5(4Z,7Z,10Z,13Z,16Z))	C65H114O6	989.8525339	No
15409	HMDB0046503	TG(22:0/18:1(11Z)/22:5(7Z,10Z,13Z,16Z,19Z))	C65H114O6	989.8525339	No
15409	HMDB0046523	TG(22:0/18:1(9Z)/22:5(4Z,7Z,10Z,13Z,16Z))	C65H114O6	989.8525339	No
15409	HMDB0046528	TG(22:0/18:1(9Z)/22:5(7Z,10Z,13Z,16Z,19Z))	C65H114O6	989.8525339	No
15409	HMDB0046552	TG(22:0/20:1(11Z)/20:5(5Z,8Z,11Z,14Z,17Z))	C65H114O6	989.8525339	No
15409	HMDB0046563	TG(22:0/20:3(5Z,8Z,11Z)/20:3(5Z,8Z,11Z))	C65H114O6	989.8525339	No
15409	HMDB0046569	TG(22:0/20:3(5Z,8Z,11Z)/20:3n6)	C65H114O6	989.8525339	No
15409	HMDB0046647	TG(22:0/18:2(9Z,12Z)/22:4(7Z,10Z,13Z,16Z))	C65H114O6	989.8525339	No
15409	HMDB0046695	TG(22:0/20:2n6/20:4(5Z,8Z,11Z,14Z))	C65H114O6	989.8525339	No
15409	HMDB0046701	TG(22:0/20:2n6/20:4(8Z,11Z,14Z,17Z))	C65H114O6	989.8525339	No
15409	HMDB0046713	TG(22:0/20:3n6/20:3(5Z,8Z,11Z))	C65H114O6	989.8525339	No
15409	HMDB0046719	TG(22:0/20:3n6/20:3n6)	C65H114O6	989.8525339	No
15409	HMDB0046743	TG(22:0/20:4(5Z,8Z,11Z,14Z)/20:2n6)	C65H114O6	989.8525339	No
15409	HMDB0046775	TG(22:0/22:2(13Z,16Z)/18:4(6Z,9Z,12Z,15Z))	C65H114O6	989.8525339	No
15409	HMDB0046791	TG(22:0/22:4(7Z,10Z,13Z,16Z)/18:2(9Z,12Z))	C65H114O6	989.8525339	No
15409	HMDB0046810	TG(22:0/22:5(4Z,7Z,10Z,13Z,16Z)/18:1(11Z))	C65H114O6	989.8525339	No
15409	HMDB0046811	TG(22:0/22:5(4Z,7Z,10Z,13Z,16Z)/18:1(9Z))	C65H114O6	989.8525339	No
15409	HMDB0046871	TG(22:0/18:4(6Z,9Z,12Z,15Z)/22:2(13Z,16Z))	C65H114O6	989.8525339	No
15409	HMDB0046893	TG(22:0/20:4(8Z,11Z,14Z,17Z)/20:2n6)	C65H114O6	989.8525339	No
15409	HMDB0046912	TG(22:0/20:5(5Z,8Z,11Z,14Z,17Z)/20:1(11Z))	C65H114O6	989.8525339	No
15409	HMDB0046935	TG(22:0/22:5(7Z,10Z,13Z,16Z,19Z)/18:1(11Z))	C65H114O6	989.8525339	No
15409	HMDB0046936	TG(22:0/22:5(7Z,10Z,13Z,16Z,19Z)/18:1(9Z))	C65H114O6	989.8525339	No
15409	HMDB0047075	TG(24:0/16:0/22:6(4Z,7Z,10Z,13Z,16Z,19Z))	C65H114O6	989.8525339	No
15409	HMDB0047213	TG(24:0/16:1(9Z)/22:5(4Z,7Z,10Z,13Z,16Z))	C65H114O6	989.8525339	No
15409	HMDB0047218	TG(24:0/16:1(9Z)/22:5(7Z,10Z,13Z,16Z,19Z))	C65H114O6	989.8525339	No
15409	HMDB0047241	TG(24:0/18:1(11Z)/20:5(5Z,8Z,11Z,14Z,17Z))	C65H114O6	989.8525339	No
15409	HMDB0047265	TG(24:0/18:1(9Z)/20:5(5Z,8Z,11Z,14Z,17Z))	C65H114O6	989.8525339	No
15409	HMDB0047303	TG(24:0/20:3(5Z,8Z,11Z)/18:3(6Z,9Z,12Z))	C65H114O6	989.8525339	No
15409	HMDB0047310	TG(24:0/20:3(5Z,8Z,11Z)/18:3(9Z,12Z,15Z))	C65H114O6	989.8525339	No
15409	HMDB0047378	TG(24:0/18:2(9Z,12Z)/20:4(5Z,8Z,11Z,14Z))	C65H114O6	989.8525339	No
15409	HMDB0047384	TG(24:0/18:2(9Z,12Z)/20:4(8Z,11Z,14Z,17Z))	C65H114O6	989.8525339	No
15409	HMDB0047395	TG(24:0/18:3(6Z,9Z,12Z)/20:3(5Z,8Z,11Z))	C65H114O6	989.8525339	No
15409	HMDB0047401	TG(24:0/18:3(6Z,9Z,12Z)/20:3n6)	C65H114O6	989.8525339	No
15409	HMDB0047431	TG(24:0/20:2n6/18:4(6Z,9Z,12Z,15Z))	C65H114O6	989.8525339	No

15409	HMDB0047447	TG(24:0/20:3n6/18:3(6Z,9Z,12Z))	C65H114O6	989.8525339	No
15409	HMDB0047454	TG(24:0/20:3n6/18:3(9Z,12Z,15Z))	C65H114O6	989.8525339	No
15409	HMDB0047470	TG(24:0/20:4(5Z,8Z,11Z,14Z)/18:2(9Z,12Z))	C65H114O6	989.8525339	No
15409	HMDB0047535	TG(24:0/22:5(4Z,7Z,10Z,13Z,16Z)/16:1(9Z))	C65H114O6	989.8525339	No
15409	HMDB0047563	TG(24:0/18:3(9Z,12Z,15Z)/20:3(5Z,8Z,11Z))	C65H114O6	989.8525339	No
15409	HMDB0047569	TG(24:0/18:3(9Z,12Z,15Z)/20:3n6)	C65H114O6	989.8525339	No
15409	HMDB0047592	TG(24:0/18:4(6Z,9Z,12Z,15Z)/20:2n6)	C65H114O6	989.8525339	No
15409	HMDB0047614	TG(24:0/20:4(8Z,11Z,14Z,17Z)/18:2(9Z,12Z))	C65H114O6	989.8525339	No
15409	HMDB0047632	TG(24:0/20:5(5Z,8Z,11Z,14Z,17Z)/18:1(11Z))	C65H114O6	989.8525339	No
15409	HMDB0047633	TG(24:0/20:5(5Z,8Z,11Z,14Z,17Z)/18:1(9Z))	C65H114O6	989.8525339	No
15409	HMDB0047655	TG(24:0/22:5(7Z,10Z,13Z,16Z,19Z)/16:1(9Z))	C65H114O6	989.8525339	No
15409	HMDB0048559	TG(16:1(9Z)/24:0/22:5(4Z,7Z,10Z,13Z,16Z))	C65H114O6	989.8525339	No
15409	HMDB0048564	TG(16:1(9Z)/24:0/22:5(7Z,10Z,13Z,16Z,19Z))	C65H114O6	989.8525339	No
15409	HMDB0048734	TG(16:1(9Z)/24:1(15Z)/22:4(7Z,10Z,13Z,16Z))	C65H114O6	989.8525339	No
15409	HMDB0048881	TG(16:1(9Z)/22:4(7Z,10Z,13Z,16Z)/24:1(15Z))	C65H114O6	989.8525339	No
15409	HMDB0049190	TG(18:1(11Z)/22:0/22:5(4Z,7Z,10Z,13Z,16Z))	C65H114O6	989.8525339	No
15409	HMDB0049195	TG(18:1(11Z)/22:0/22:5(7Z,10Z,13Z,16Z,19Z))	C65H114O6	989.8525339	No
15409	HMDB0049215	TG(18:1(11Z)/24:0/20:5(5Z,8Z,11Z,14Z,17Z))	C65H114O6	989.8525339	No
15409	HMDB0049357	TG(18:1(11Z)/22:1(13Z)/22:4(7Z,10Z,13Z,16Z))	C65H114O6	989.8525339	No
15409	HMDB0049376	TG(18:1(11Z)/24:1(15Z)/20:4(5Z,8Z,11Z,14Z))	C65H114O6	989.8525339	No
15409	HMDB0049382	TG(18:1(11Z)/24:1(15Z)/20:4(8Z,11Z,14Z,17Z))	C65H114O6	989.8525339	No
15409	HMDB0049476	TG(18:1(11Z)/20:4(5Z,8Z,11Z,14Z)/24:1(15Z))	C65H114O6	989.8525339	No
15409	HMDB0049517	TG(18:1(11Z)/22:4(7Z,10Z,13Z,16Z)/22:1(13Z))	C65H114O6	989.8525339	No
15409	HMDB0049602	TG(18:1(11Z)/20:4(8Z,11Z,14Z,17Z)/24:1(15Z))	C65H114O6	989.8525339	No
15409	HMDB0049813	TG(18:1(9Z)/22:0/22:5(4Z,7Z,10Z,13Z,16Z))	C65H114O6	989.8525339	No
15409	HMDB0049818	TG(18:1(9Z)/22:0/22:5(7Z,10Z,13Z,16Z,19Z))	C65H114O6	989.8525339	No
15409	HMDB0049837	TG(18:1(9Z)/24:0/20:5(5Z,8Z,11Z,14Z,17Z))	C65H114O6	989.8525339	No
15409	HMDB0049972	TG(18:1(9Z)/22:1(13Z)/22:4(7Z,10Z,13Z,16Z))	C65H114O6	989.8525339	No
15409	HMDB0049990	TG(18:1(9Z)/24:1(15Z)/20:4(5Z,8Z,11Z,14Z))	C65H114O6	989.8525339	No
15409	HMDB0049996	TG(18:1(9Z)/24:1(15Z)/20:4(8Z,11Z,14Z,17Z))	C65H114O6	989.8525339	No
15409	HMDB0050085	TG(18:1(9Z)/20:4(5Z,8Z,11Z,14Z)/24:1(15Z))	C65H114O6	989.8525339	No
15409	HMDB0050124	TG(18:1(9Z)/22:4(7Z,10Z,13Z,16Z)/22:1(13Z))	C65H114O6	989.8525339	No
15409	HMDB0050205	TG(18:1(9Z)/20:4(8Z,11Z,14Z,17Z)/24:1(15Z))	C65H114O6	989.8525339	No
15409	HMDB0050387	TG(20:1(11Z)/20:0/22:5(4Z,7Z,10Z,13Z,16Z))	C65H114O6	989.8525339	No
15409	HMDB0050392	TG(20:1(11Z)/20:0/22:5(7Z,10Z,13Z,16Z,19Z))	C65H114O6	989.8525339	No
15409	HMDB0050410	TG(20:1(11Z)/22:0/20:5(5Z,8Z,11Z,14Z,17Z))	C65H114O6	989.8525339	No
15409	HMDB0050519	TG(20:1(11Z)/20:1(11Z)/22:4(7Z,10Z,13Z,16Z))	C65H114O6	989.8525339	No
15409	HMDB0050537	TG(20:1(11Z)/20:3(5Z,8Z,11Z)/22:2(13Z,16Z))	C65H114O6	989.8525339	No
15409	HMDB0050555	TG(20:1(11Z)/22:1(13Z)/20:4(5Z,8Z,11Z,14Z))	C65H114O6	989.8525339	No
15409	HMDB0050561	TG(20:1(11Z)/22:1(13Z)/20:4(8Z,11Z,14Z,17Z))	C65H114O6	989.8525339	No
15409	HMDB0050579	TG(20:1(11Z)/24:1(15Z)/18:4(6Z,9Z,12Z,15Z))	C65H114O6	989.8525339	No
15409	HMDB0050651	TG(20:1(11Z)/20:3n6/22:2(13Z,16Z))	C65H114O6	989.8525339	No
15409	HMDB0050663	TG(20:1(11Z)/20:4(5Z,8Z,11Z,14Z)/22:1(13Z))	C65H114O6	989.8525339	No
15409	HMDB0050681	TG(20:1(11Z)/22:2(13Z,16Z)/20:3(5Z,8Z,11Z))	C65H114O6	989.8525339	No
15409	HMDB0050687	TG(20:1(11Z)/22:2(13Z,16Z)/20:3n6)	C65H114O6	989.8525339	No
15409	HMDB0050699	TG(20:1(11Z)/22:4(7Z,10Z,13Z,16Z)/20:1(11Z))	C65H114O6	989.8525339	No
15409	HMDB0050759	TG(20:1(11Z)/18:4(6Z,9Z,12Z,15Z)/24:1(15Z))	C65H114O6	989.8525339	No
15409	HMDB0050777	TG(20:1(11Z)/20:4(8Z,11Z,14Z,17Z)/22:1(13Z))	C65H114O6	989.8525339	No
15409	HMDB0050959	TG(20:3(5Z,8Z,11Z)/22:0/20:3(5Z,8Z,11Z))	C65H114O6	989.8525339	No
15409	HMDB0050965	TG(20:3(5Z,8Z,11Z)/22:0/20:3n6)	C65H114O6	989.8525339	No
15409	HMDB0050981	TG(20:3(5Z,8Z,11Z)/24:0/18:3(6Z,9Z,12Z))	C65H114O6	989.8525339	No
15409	HMDB0050988	TG(20:3(5Z,8Z,11Z)/24:0/18:3(9Z,12Z,15Z))	C65H114O6	989.8525339	No
15409	HMDB0051075	TG(20:3(5Z,8Z,11Z)/20:1(11Z)/22:2(13Z,16Z))	C65H114O6	989.8525339	No
15409	HMDB0051108	TG(20:3(5Z,8Z,11Z)/22:1(13Z)/20:2n6)	C65H114O6	989.8525339	No
15409	HMDB0051124	TG(20:3(5Z,8Z,11Z)/24:1(15Z)/18:2(9Z,12Z))	C65H114O6	989.8525339	No
15409	HMDB0051141	TG(20:3(5Z,8Z,11Z)/18:2(9Z,12Z)/24:1(15Z))	C65H114O6	989.8525339	No
15409	HMDB0051176	TG(20:3(5Z,8Z,11Z)/20:2n6/22:1(13Z))	C65H114O6	989.8525339	No
15409	HMDB0051468	TG(22:1(13Z)/18:0/22:5(4Z,7Z,10Z,13Z,16Z))	C65H114O6	989.8525339	No
15409	HMDB0051473	TG(22:1(13Z)/18:0/22:5(7Z,10Z,13Z,16Z,19Z))	C65H114O6	989.8525339	No
15409	HMDB0051489	TG(22:1(13Z)/20:0/20:5(5Z,8Z,11Z,14Z,17Z))	C65H114O6	989.8525339	No
15409	HMDB0051569	TG(22:1(13Z)/18:1(11Z)/22:4(7Z,10Z,13Z,16Z))	C65H114O6	989.8525339	No

15409	HMDB0051586	TG(22:1(13Z)/18:1(9Z)/22:4(7Z,10Z,13Z,16Z))	C65H114O6	989.8525339	No
15409	HMDB0051601	TG(22:1(13Z)/20:1(11Z)/20:4(5Z,8Z,11Z,14Z))	C65H114O6	989.8525339	No
15409	HMDB0051607	TG(22:1(13Z)/20:1(11Z)/20:4(8Z,11Z,14Z,17Z))	C65H114O6	989.8525339	No
15409	HMDB0051616	TG(22:1(13Z)/20:3(5Z,8Z,11Z)/20:2n6)	C65H114O6	989.8525339	No
15409	HMDB0051640	TG(22:1(13Z)/22:1(13Z)/18:4(6Z,9Z,12Z,15Z))	C65H114O6	989.8525339	No
15409	HMDB0051687	TG(22:1(13Z)/18:3(6Z,9Z,12Z)/22:2(13Z,16Z))	C65H114O6	989.8525339	No
15409	HMDB0051702	TG(22:1(13Z)/20:2n6/20:3n6)	C65H114O6	989.8525339	No
15409	HMDB0051718	TG(22:1(13Z)/20:3n6/20:2n6)	C65H114O6	989.8525339	No
15409	HMDB0051751	TG(22:1(13Z)/22:2(13Z,16Z)/18:3(6Z,9Z,12Z))	C65H114O6	989.8525339	No
15409	HMDB0051758	TG(22:1(13Z)/22:2(13Z,16Z)/18:3(9Z,12Z,15Z))	C65H114O6	989.8525339	No
15409	HMDB0051806	TG(22:1(13Z)/18:3(9Z,12Z,15Z)/22:2(13Z,16Z))	C65H114O6	989.8525339	No
15409	HMDB0051816	TG(22:1(13Z)/18:4(6Z,9Z,12Z,15Z)/22:1(13Z))	C65H114O6	989.8525339	No
15409	HMDB0051957	TG(24:1(15Z)/16:0/22:5(4Z,7Z,10Z,13Z,16Z))	C65H114O6	989.8525339	No
15409	HMDB0051962	TG(24:1(15Z)/16:0/22:5(7Z,10Z,13Z,16Z,19Z))	C65H114O6	989.8525339	No
15409	HMDB0051977	TG(24:1(15Z)/18:0/20:5(5Z,8Z,11Z,14Z,17Z))	C65H114O6	989.8525339	No
15409	HMDB0052052	TG(24:1(15Z)/16:1(9Z)/22:4(7Z,10Z,13Z,16Z))	C65H114O6	989.8525339	No
15409	HMDB0052066	TG(24:1(15Z)/18:1(11Z)/20:4(5Z,8Z,11Z,14Z))	C65H114O6	989.8525339	No
15409	HMDB0052072	TG(24:1(15Z)/18:1(11Z)/20:4(8Z,11Z,14Z,17Z))	C65H114O6	989.8525339	No
15409	HMDB0052082	TG(24:1(15Z)/18:1(9Z)/20:4(5Z,8Z,11Z,14Z))	C65H114O6	989.8525339	No
15409	HMDB0052088	TG(24:1(15Z)/18:1(9Z)/20:4(8Z,11Z,14Z,17Z))	C65H114O6	989.8525339	No
15409	HMDB0052103	TG(24:1(15Z)/20:1(11Z)/18:4(6Z,9Z,12Z,15Z))	C65H114O6	989.8525339	No
15409	HMDB0052110	TG(24:1(15Z)/20:3(5Z,8Z,11Z)/18:2(9Z,12Z))	C65H114O6	989.8525339	No
15409	HMDB0052161	TG(24:1(15Z)/18:2(9Z,12Z)/20:3n6)	C65H114O6	989.8525339	No
15409	HMDB0052176	TG(24:1(15Z)/18:3(6Z,9Z,12Z)/20:2n6)	C65H114O6	989.8525339	No
15409	HMDB0052191	TG(24:1(15Z)/20:2n6/18:3(6Z,9Z,12Z))	C65H114O6	989.8525339	No
15409	HMDB0052198	TG(24:1(15Z)/20:2n6/18:3(9Z,12Z,15Z))	C65H114O6	989.8525339	No
15409	HMDB0052206	TG(24:1(15Z)/20:3n6/18:2(9Z,12Z))	C65H114O6	989.8525339	No
15409	HMDB0052288	TG(24:1(15Z)/18:3(9Z,12Z,15Z)/20:2n6)	C65H114O6	989.8525339	No
15409	HMDB0052477	TG(18:2(9Z,12Z)/22:0/22:4(7Z,10Z,13Z,16Z))	C65H114O6	989.8525339	No
15409	HMDB0052490	TG(18:2(9Z,12Z)/24:0/20:4(5Z,8Z,11Z,14Z))	C65H114O6	989.8525339	No
15409	HMDB0052496	TG(18:2(9Z,12Z)/24:0/20:4(8Z,11Z,14Z,17Z))	C65H114O6	989.8525339	No
15409	HMDB0052609	TG(18:2(9Z,12Z)/24:1(15Z)/20:3n6)	C65H114O6	989.8525339	No
15409	HMDB0052701	TG(18:2(9Z,12Z)/22:2(13Z,16Z)/22:2(13Z,16Z))	C65H114O6	989.8525339	No
15409	HMDB0052931	TG(18:3(6Z,9Z,12Z)/24:0/20:3n6)	C65H114O6	989.8525339	No
15409	HMDB0053031	TG(18:3(6Z,9Z,12Z)/22:1(13Z)/22:2(13Z,16Z))	C65H114O6	989.8525339	No
15409	HMDB0053042	TG(18:3(6Z,9Z,12Z)/24:1(15Z)/20:2n6)	C65H114O6	989.8525339	No
15409	HMDB0053320	TG(20:2n6/20:0/22:4(7Z,10Z,13Z,16Z))	C65H114O6	989.8525339	No
15409	HMDB0053331	TG(20:2n6/22:0/20:4(5Z,8Z,11Z,14Z))	C65H114O6	989.8525339	No
15409	HMDB0053337	TG(20:2n6/22:0/20:4(8Z,11Z,14Z,17Z))	C65H114O6	989.8525339	No
15409	HMDB0053349	TG(20:2n6/24:0/18:4(6Z,9Z,12Z,15Z))	C65H114O6	989.8525339	No
15409	HMDB0053434	TG(20:2n6/22:1(13Z)/20:3n6)	C65H114O6	989.8525339	No
15409	HMDB0053452	TG(20:2n6/24:1(15Z)/18:3(9Z,12Z,15Z))	C65H114O6	989.8525339	No
15409	HMDB0053488	TG(20:2n6/20:2n6/22:2(13Z,16Z))	C65H114O6	989.8525339	No
15409	HMDB0053524	TG(20:2n6/22:2(13Z,16Z)/20:2n6)	C65H114O6	989.8525339	No
15409	HMDB0053713	TG(20:3n6/22:0/20:3n6)	C65H114O6	989.8525339	No
15409	HMDB0053730	TG(20:3n6/24:0/18:3(9Z,12Z,15Z))	C65H114O6	989.8525339	No
15409	HMDB0053787	TG(20:3n6/20:1(11Z)/22:2(13Z,16Z))	C65H114O6	989.8525339	No
15409	HMDB0054057	TG(20:4(5Z,8Z,11Z,14Z)/20:0/22:2(13Z,16Z))	C65H114O6	989.8525339	No
15409	HMDB0054372	TG(22:2(13Z,16Z)/18:0/22:4(7Z,10Z,13Z,16Z))	C65H114O6	989.8525339	No
15409	HMDB0054386	TG(22:2(13Z,16Z)/20:0/20:4(8Z,11Z,14Z,17Z))	C65H114O6	989.8525339	No
15409	HMDB0054395	TG(22:2(13Z,16Z)/22:0/18:4(6Z,9Z,12Z,15Z))	C65H114O6	989.8525339	No
15409	HMDB0054474	TG(22:2(13Z,16Z)/22:1(13Z)/18:3(9Z,12Z,15Z))	C65H114O6	989.8525339	No
15409	HMDB0054491	TG(22:2(13Z,16Z)/18:2(9Z,12Z)/22:2(13Z,16Z))	C65H114O6	989.8525339	No
