# Supplementary material for: High-Throughput Metabolomics and Diabetic Kidney Disease Progression: Evidence from the Chronic Renal Insufficiency (CRIC) Study
Source: Am J Nephrol. Author manuscript; Available in PMC 2023 Dec 7. (PMC9116599; doi:10.1159/000521940)
Supplement: Zhang J Neph Methods and Table [file NIHMS1800823-supplement-Zhang_J_Neph_Methods_and_Table.pdf]

Supplementary Material

Untargeted Urine metabolomics assay: sample processing and feature extraction

Frozen 24-hour urine samples from 995 CRIC participants and a healthy control were thawed, centrifuged and precipitate-free supernatants were diluted 1:50 in double distilled water in 96 well polypropylene storage microplates (AB-1058, Abgene) sealed with easy-peel heat sealing foil (AB-0745, Abgene) and kept frozen until analysis. Diluted urines were shipped on dry ice and stored at -80 Celsius up to 2 weeks prior to data acquisition by mass spectrometry. Samples were injected in duplicate (i.e. technical replicates) with an MPS 3 xt autosampler (Gerstel) coupled to an Agilent 6550 Q-TOF mass spectrometer (Agilent Technologies) by non-targeted flow injection analysis (Fuhrer et al., 2011). Briefly, the flow rate was 150 μL/min of mobile phase consisting of isopropanol/water (60:40, v/v) buffered with 5 mM ammonium fluoride, and for online mass axis correction, homo-taurine and hexakis (^{1}H, ^{1}H, ^{3}H tetrafluoropropoxy) phosphazine (HP-0921, Agilent Technologies) were added to the mobile phase. Profile mass spectra (MS1) were recorded in 4Ghz acquisition mode from 50 to 1000 m/z in negative ionization mode with the following source settings: temperature 225° C, drying gas 11 L/min, nebulizer pressure 20 psig, sheath gas temperature 350° C, sheath gas flow 10 L/min, Vcap voltage 3500 V, nozzle voltage 2000 V, fragmentor voltage 350 V and Oct 1 RF Vpp voltage 750V. All steps of data processing and analysis were performed with Matlab R2017b (The Mathworks) using functions embedded in the bioinformatics, statistics, database, and parallel computing toolboxes. After sample alignment and gap-filling, correction for ion intensity drift over time and between plates was performed, and the common mass axis was recalibrated using known frequently occurring ions. Approximately 15k commonly observed ions masses were annotated based on accurate mass comparison using 1 mDa mass tolerance against the Human Metabolome Database HMDBv4.0, resulting in 1899 annotated ion masses assuming single deprotonation (Supplemental Table S6).

Targeted Urine metabolomics assay: sample processing and feature extraction

A subset of 15 candidate metabolites from the untargeted panel were assayed by using a quantitative capillary electrophoresis (CE) coupled to mass spectrometry method (ZipChip-Qexactive) to validate metabolite ion identification and annotations from the untargeted method. For the ZipChip assay, 10 µL of urine sample or calibration standard mixture was mixed with 90 µL of extraction solution containing 80% methanol, 100 mM ammonium acetate and 1 µM of stable isotope labeled internal standards in a 96 well plate. After mixing, samples or calibration standards were kept in a -20°C freezer for at least one hour and then centrifuged at 5000 rpm for 5 minutes. An 50 µL supernatant was transferred to a clean 96 well plate for analysis. Metabolite separation was achieved with a microfluidic chip which integrates capillary electrophoresis (CE) with nano-electrospray ionization through ZipChip interface (908 Devices, Boston, MA). For each sample, 20 µL solution was placed into the sample well and subsequently injected into the HS chip using a 10-s load time. A field strength of 1000V/cm was applied for separation over 4 minutes using a background electrolyte solution consisting of 2% formic acid in 50% methanol in water. The mass spectrometry acquisition was done with Q-Exactive mass spectrometer (Thermo, San Jose, CA). The resolving power was 17,500 with an AGC target of 3×10^6, maximum injection time of 20 ms, and scan range of 75–500 m/z. Thermo Scientific's software Xcalibur-Quan Browser was used for quantitative data processing. Calibration curves for all metabolites were included with each plate. The targeted assay metabolite data were normalized to urine creatinine measured at the CRIC Central Laboratory.

Untargeted metabolomics data: filtering metabolic features.

We leveraged technical replicate data for each sample to develop criteria for filtering out metabolite ions that showed poor reproducibility. A total of 1899 annotated metabolite ions were

measured for the 995 CRIC subjects along with a pooled urine sample from the healthy subject, as quality control (QC). All data were collected in duplicates. The three QC sample per plate were randomly run within each 96 well plate in duplicates leading to 6 measurements per plate and a total of 198 measurements across 33 plates. The filtering process consists of three steps. In step one, only the QC data was used. Three reliability metrics, the correlation coefficient between the technical duplicates (Spearman and Pearson **QC CC**), intraclass correlation (**QC ICC**) and coefficient of variation (**QC CV**) for each metabolite ion abundance were calculated. The QC ICC for each metabolite ion abundance was calculated as the variance between technical duplicates divided by the total variation (including batch and replicate variation); the QC CV was calculated as the standard deviation between technical duplicate abundance divided by the mean of the samples. Low correlations, high ICC or high CV values would indicate poor reliability, i.e., high variability relative to signal. We formalized these criteria and excluded ions for which any of the following was true:

- QC_Spearman < 0.85 or QC_Pearson < 0.85
- QC_CV ≥ 0.05
- QC_ICC ≥ 0.05

These cut offs are based on standard assay reliability metrics. We then implemented filtering step two using the CRIC samples. Here the objective was to filter out metabolite ions which exhibited low biological variability. To this end we calculated an intraclass correlation (CRIC ICC), as the ratio of between subject to total sample variation; ions with ICC <= 0.35 were further excluded.

Thus, in summary, the final set of 698 retained metabolite ions satisfied all criteria: QC Spearman >=0.85 & QC_Pearson >= 0.85 & QC_CV < 0.05 & QC_ICC < 0.05 & CRIC_ICC > 0.35. The technical replicates of the metabolite ion abundances (two per sample) were then averaged. In the

final filtering step, we excluded metabolite ions with low abundance to exclude non-informative ions in the noise range. The ions that passed our filtering criteria and count threshold, constituted the final metabolite ion feature set for modeling.

Variability of 15 targeted metabolites: Of the 13 statistically significant metabolites in the target analysis, 7 had a QC CV of ≤ 5%, 3 had a QC CV of between 5% and up to 10%, and 3 (i.e.,Ornithine, Isoleucine, Betaine), had a QC CV of more than 10%. The 2 non-significant metabolites, DL.Homocystine and Nicotinic acid, were not detected in the QC samples.

Supplemental Table S1. Associations between single metabolite ions* and eGFR slopes, adjusted for 9 clinical variables

**Ion Index**	**Estimate †**	**Lower**	**Upper**	**P value**	**FDR.P value**	Geometric mean (95%Ci)	**Name**
1098	-0.44	-0.68	-0.2	0.000361	0.04198	25511.72 (25009.12, 26024.42)	3-(4-Methyl-3-pentenyl)thiophene
1099	-0.45	-0.66	-0.24	2.90E-05	0.01012	16598.07 (16250.48, 16953.1)	C10:3
281	0.27	0.14	0.41	7.19E-05	0.01672	417279.81 (402283.15, 432835.52)	Furoic acid
30	0.3	0.15	0.46	0.00016	0.02787	10894.52 (10557.28, 11242.53)	Butynal
678	-0.45	-0.7	-0.21	0.000335	0.04198	21007.91 (20618.87, 21404.29)	Cycloheptanecarboxylic acid
9178	0.25	0.16	0.34	1.96E-07	0.000137	18245.82 (17390.49, 19143.22)	3,4-Dicaffeoyl-1,5-quinolactone

*Results for 6 significant metabolites after FDR correction from a total of 698 metabolite ion candidates.

†Estimates are from linear regression models using log2 transformed metabolite ion abundances.

Supplemental Table S2. Associations between single metabolite ion* and time-to-ESKD, adjusted for 9 clinical variables

Ion Index	HR †	lower	upper	p	FDR.p	Geometric Mean (95%Ci)	Name
1019	1.54	1.21	1.97	0	0.01	193443.23 (189589.41, 197375.38)	Hydroxyadipate
1019	1.54	1.21	1.97	0	0.01	193443.23 (189589.41, 197375.38)	(-)-1-Methylpropyl 1-propenyl disulfide
1022	1.47	1.15	1.87	0	0.02	18222.56 (17852.12, 18600.69)	Cymarose
1036	1.38	1.12	1.69	0	0.02	71019.19 (69557.9, 72511.17)	Acetylcysteine
1075	1.27	1.07	1.5	0.01	0.05	46124.24 (44819.74, 47466.71)	4-Pyridoxolactone
1079	1.48	1.21	1.8	0	0.01	50991.66 (49719.96, 52295.89)	Phenylalanine
1098	1.84	1.45	2.32	0	0	25511.72 (25009.12, 26024.42)	3-(4-Methyl-3-pentenyl)thiophene
1127	1.31	1.08	1.59	0.01	0.04	17488.86 (17008.88, 17982.38)	Methyl 1-(methylthio)propyl disulfide
1165	1.41	1.12	1.78	0	0.03	12907.19 (12644.8, 13175.02)	Glycerone sulfate
1167	1.32	1.08	1.62	0.01	0.05	19637.46 (19153.11, 20134.05)	Benzyl methyl disulfide
1167	1.32	1.08	1.62	0.01	0.05	19637.46 (19153.11, 20134.05)	Gallic acid
1293	1.42	1.16	1.74	0	0.01	40878.75 (39843.27, 41941.15)	Berteroin
1293	1.42	1.16	1.74	0	0.01	40878.75 (39843.27, 41941.15)	Acetyl-Asp
1294	1.39	1.14	1.71	0	0.02	24241.14 (23726.58, 24766.87)	Indole-3-acetate

1337	1.32	1.15	1.52	0	0.01	24288.61 (23513.63, 25089.13)	Polyvidone
1337	1.32	1.15	1.52	0	0.01	24288.61 (23513.63, 25089.13)	Formyl-Met
1486	1.12	1.03	1.21	0.01	0.05	1433393.25 (1345812.7, 1526673.22)	4-Pyridoxate
1695	1.38	1.13	1.69	0	0.02	26895.48 (26270.83, 27534.99)	5-Hydroxyindoleacetic acid
1983	1.34	1.09	1.66	0.01	0.05	105402.12 (103059.83, 107797.64)	Sebacic acid
2004	1.49	1.24	1.79	0	0	10527.86 (10267.45, 10794.89)	Indolepyruvate
2028	1.32	1.11	1.58	0	0.02	38218.72 (37123.22, 39346.55)	Tryptophan
2030	1.49	1.15	1.92	0	0.03	17238.22 (16913.82, 17568.85)	Serinyl-Valine
2046	1.59	1.24	2.03	0	0.01	38921.25 (38135.27, 39723.42)	N-Methylcalystegine C1
2046	1.59	1.24	2.03	0	0.01	38921.25 (38135.27, 39723.42)	Dihydro-2,4-dimethyl-6-(2-methylpropyl)-4H-1,3,5-dithiazine
2094	1.29	1.09	1.53	0	0.03	7727.64 (7519.45, 7941.59)	Isoniazid pyruvate
2115	1.45	1.2	1.75	0	0.01	11825.9 (11523.55, 12136.19)	Dihydrolipoate
2115	1.45	1.2	1.75	0	0.01	11825.9 (11523.55, 12136.19)	Aminofurantoin
2115	1.45	1.2	1.75	0	0.01	11825.9 (11523.55, 12136.19)	Methyl 5-(1-Propynyl)-2-thiophenepropanoate
215	1.35	1.12	1.62	0	0.02	31559.9 (30800.84, 32337.67)	Aminobutanoic acid (ABA)
2232	1.47	1.17	1.83	0	0.02	10597.09 (10372.1, 10826.97)	Cucurbic acid

226	1.79	1.37	2.34	0	0	36708.43 (35995.85, 37435.12)	Hydroxybutanoic acid
2360	1.36	1.13	1.65	0	0.02	29428.94 (28673.72, 30204.05)	1-Isothiocyanato-8-(methylthio)octane
2430	1.35	1.13	1.62	0	0.02	15298.62 (14875.87, 15733.39)	1-(5-Acetyl-2-hydroxyphenyl)-3-methyl-1-butanone
2456	1.49	1.21	1.83	0	0.01	29508.77 (28839.54, 30193.54)	Methyl 5-hydroxyoxindole-3-acetate
2456	1.49	1.21	1.83	0	0.01	29508.77 (28839.54, 30193.54)	N-lactoyl-Methionine
2480	1.29	1.1	1.51	0	0.02	27796.64 (26998.94, 28617.92)	3-(6-hydroxy-7-methoxy-2H-1,3-benzodioxol-5-yl)prop-2-enal
2705	1.55	1.28	1.88	0	0	6106.37 (5960.97, 6255.32)	Isoleucyl-Valine
3049	1.23	1.06	1.42	0.01	0.04	7745.56 (7498.43, 8000.84)	Frovatriptan
3078	1.48	1.22	1.8	0	0.01	34343.79 (33610.28, 35093.31)	1-O-Galloylglycerol
3079	1.41	1.12	1.78	0	0.03	478096.9 (467410.77, 489027.34)	Uridine
3248	1.26	1.1	1.44	0	0.02	11376.16 (10996.85, 11768.56)	3,5-dihydroxy-4-(sulfooxy)benzoic acid
3310	1.32	1.09	1.61	0	0.04	8357.32 (8162.09, 8557.23)	5-Hydroxy-N-formylkynurenine
3422	1.33	1.14	1.54	0	0.01	28194.15 (27273.67, 29145.69)	(2R,3S)-Piscidic acid
3425	1.37	1.15	1.62	0	0.01	26614.89 (25857.01, 27394.98)	3-hydroxy-3-(3,4,5-trimethoxyphenyl)propanoic acid
3429	1.46	1.19	1.79	0	0.01	20227.28 (19750.38, 20715.71)	1-Methoxy-1-(2,4,5-trimethoxyphenyl)-2-propanol
343	1.55	1.23	1.96	0	0.01	20768.23 (20346.21, 21199)	Indole

3435	1.53	1.19	1.97	0	0.02	1838727.3 (1803916.21, 1874210.15)	C16:0
344	1.5	1.2	1.86	0	0.01	28340.9 (27753.57, 28940.66)	Valine; Betaine
362	1.42	1.19	1.69	0	0.01	13425.06 (13074.77, 13784.74)	Threonine
3778	1.36	1.16	1.59	0	0.01	9701.78 (9441.69, 9969.03)	3-(6,7-dimethoxy-2H-1,3-benzodioxol-5-yl)oxirane-2-carboxylic acid
3778	1.36	1.16	1.59	0	0.01	9701.78 (9441.69, 9969.03)	DL-Homocystine
4048	1.41	1.16	1.71	0	0.01	9365.57 (9138.14, 9598.67)	Alpha-CEHC
410	1.36	1.16	1.61	0	0.01	36796.27 (35733.18, 37890.99)	Nicotinate
4152	1.39	1.14	1.69	0	0.02	5575.09 (5449.61, 5703.46)	Lactapiperanol C
4156	1.77	1.41	2.22	0	0	58945.49 (57717.85, 60199.25)	Oleic acid
4207	1.51	1.26	1.82	0	0	20553.41 (20062.09, 21056.76)	6-Thioinosinic acid
4220	1.55	1.19	2.02	0	0.02	3479145.07 (3414841.67, 3544659.35)	C18:0
4383	1.22	1.06	1.41	0.01	0.05	11419.74 (11059.39, 11791.84)	Trimethoprim
4406	1.25	1.09	1.43	0	0.02	16400.8 (15850.14, 16970.6)	Hawkinsin
443	1.36	1.11	1.66	0	0.03	14985.02 (14692.13, 15283.75)	2,3,6-Trihydroxypyridine
4489	1.34	1.08	1.67	0.01	0.05	10075.7 (9857.36, 10298.88)	gamma-Glutamylphenylalanine
4674	1.27	1.1	1.46	0	0.02	56745.9 (54792.05, 58769.41)	Promazine 5-sulfoxide

473	1.19	1.05	1.35	0.01	0.04	16055.15 (15487.99, 16643.08)	Pipecolate
4754	1.28	1.09	1.5	0	0.02	13413.73 (13016.12, 13823.49)	Arginyl-Glutamine
490	1.48	1.12	1.94	0	0.04	19693.72 (19340.71, 20053.16)	4-Methyl-2-oxopentanoate
4911	1.38	1.11	1.72	0	0.04	16430.09 (16080.87, 16786.9)	D-Erythroascorbic acid 1''-a-D-glucoside
505	1.33	1.12	1.57	0	0.02	7235.05 (7049.86, 7425.09)	(±)-2-Propylthiazolidine
506	1.28	1.07	1.52	0.01	0.05	44228.25 (43041.67, 45447.54)	(Iso)Leucine
5140	1.24	1.06	1.44	0.01	0.05	9888.5 (9595.22, 10190.75)	10-Hydroxy-8-nor-2-fenchanone glucoside
522	1.32	1.08	1.61	0.01	0.05	12595.79 (12298.74, 12900.01)	Asparagine
526	1.31	1.13	1.51	0	0.01	22896.08 (22182.14, 23633.01)	Ornithine
5302	1.28	1.09	1.52	0	0.03	8421.12 (8211.17, 8636.44)	Zeranol
534	1.65	1.35	2.02	0	0	31057.32 (30386.43, 31743.02)	Aspartate
5392	1.46	1.11	1.93	0.01	0.05	89602.1 (87991.01, 91242.7)	2-Dodecylbenzenesulfonic acid
5392	1.46	1.11	1.93	0.01	0.05	89602.1 (87991.01, 91242.7)	Heptaethylene glycol
548	1.22	1.06	1.41	0.01	0.04	38889.49 (37600.93, 40222.2)	Malate
548	1.22	1.06	1.41	0.01	0.04	38889.49 (37600.93, 40222.2)	3,3-Dimethyl-1,2-dithiolane
5566	1.25	1.08	1.45	0	0.03	8522.47 (8248.01, 8806.07)	(R)-Marmin
561	1.44	1.11	1.87	0.01	0.05	31774.95 (31251.39, 32307.27)	Adenine
576	1.45	1.13	1.87	0	0.03	609099.44 (596985.57, 621459.12)	Threonate

576	1.45	1.13	1.87	0	0.03	609099.44 (596985.57, 621459.12)	1-Pentanesulfenothioic acid
5810	1.22	1.08	1.38	0	0.02	49493.86 (47485.83, 51586.82)	Sulpiride
592	1.34	1.12	1.61	0	0.02	42123.22 (40959.15, 43320.37)	Anthranilate
6153	1.3	1.08	1.57	0.01	0.05	3805.84 (3710.45, 3903.69)	Prostaglandin F2a
628	1.14	1.06	1.24	0	0.02	522340.33 (491410.18, 555217.27)	Gabaculine
6281	1.18	1.05	1.32	0	0.04	15679.07 (15070.75, 16311.94)	Triglochinin
644	1.3	1.13	1.49	0	0.01	99902.18 (96338.97, 103597.17)	3,4-Diethylthiophene
645	1.44	1.11	1.88	0.01	0.05	41976.88 (41179.21, 42789.99)	cis-1,2-Dihydro-3-ethylcatechol
6653	1.23	1.11	1.37	0	0.01	98556.82 (94215.21, 103098.5)	Geniposidic acid
6757	1.42	1.15	1.76	0	0.02	12946.33 (12688.07, 13209.85)	18-Hydroxycortisol
6783	1.27	1.08	1.5	0	0.04	9876.1 (9595.41, 10165.01)	2-(Arabinosylamino)-3-(glucosylamino)propanenitrile
6876	1.28	1.08	1.53	0.01	0.04	8896.2 (8658.52, 9140.39)	Ceftizoxime
6975	1.33	1.1	1.61	0	0.03	8284.61 (8073.53, 8501.22)	5,6-Dihydroxyprostaglandin F1a
718	1.43	1.17	1.75	0	0.01	26400.56 (25817.42, 26996.87)	2-Oxoglutaramate
721	1.51	1.21	1.88	0	0.01	126151.85 (123504.2, 128856.27)	4-Acetamidobutanoate
740	1.23	1.08	1.4	0	0.02	64292.7 (61945.88, 66728.43)	Lysine

753	1.31	1.09	1.57	0	0.04	34166.98 (33287.17, 35070.05)	Indole-3-carbinol
766	1.4	1.13	1.73	0	0.02	221950.22 (216711.34, 227315.74)	Hydroxyglutarate
766	1.4	1.13	1.73	0	0.02	221950.22 (216711.34, 227315.74)	2-Propenyl propyl disulfide
767	1.49	1.17	1.9	0	0.02	20985.39 (20551.63, 21428.32)	Carbamoyl-Ser
7671	1.28	1.08	1.53	0	0.04	18703.77 (18202.6, 19218.75)	Aminophylline
798	1.36	1.11	1.67	0	0.03	360778.44 (352719.19, 369021.83)	Diisopropyl disulfide
798	1.36	1.11	1.67	0	0.03	360778.44 (352719.19, 369021.83)	Pentose
8568	1.16	1.05	1.28	0	0.03	19442.1 (18500.55, 20431.57)	Cholylglycine
8590	1.29	1.1	1.51	0	0.02	4647.21 (4514.03, 4784.31)	Dolichyl b-D-glucosyl phosphate
866	1.49	1.18	1.89	0	0.02	65807.37 (64500.23, 67141)	5-(2-carboxylatoethyl)-4-oxo-4,5-dihydro-1H-imidazol-5-ide
9355	1.35	1.13	1.6	0	0.02	6130.01 (5966.76, 6297.73)	2-(2,4-dihydroxyphenyl)-3-(3,7-dimethylocta-2,6-dien-1-yl)-5,7-dihydroxy-6-(4-hydroxy-3-methylbut-2-en-1-yl)-3,4-dihydro-2H-1-benzopyran-4-one
9355	1.35	1.13	1.6	0	0.02	6130.01 (5966.76, 6297.73)	Argatroban
9464	1.33	1.14	1.57	0	0.01	7841.61 (7614.47, 8075.54)	Capsianoside V
9483	1.28	1.11	1.48	0	0.01	4648.39 (4497.34, 4804.51)	Taurocholic acid
965	1.27	1.1	1.46	0	0.02	22321.34 (21565.53, 23103.63)	L-2-Amino-4-methylenepentanedioic acid
9706	1.2	1.05	1.36	0.01	0.04	7841.06 (7547.01, 8146.56)	N-[(3a,5b,7b)-7-hydroxy-24-oxo-3-

							(sulfooxy)cholan-24-yl]-Glycine
9728	1.53	1.17	1.99	0	0.02	136209 (133639.67, 138827.72)	Sabadelin
999	1.3	1.1	1.55	0	0.03	54960.27 (53547.78, 56410.02)	2-Indolecarboxylic acid

*Results for 99 significant metabolite ions after FDR correction from a total of 698 metabolite ion candidates.
† Hazards ratios are from Cox regression models using log2 transformed metabolite abundances.

Supplemental Table S3. Machine learning and penalized regression models predicting eGFR slope: number of variables selected by each model from among 698 metabolite ions and 9 clinical variables

**Models***	**Inclusion criteria for clinical variables: Forced (F) or Not forced (NF)**	**Number of variables selected**	**Number of Metabolite ions selected**
1-LASSO (λ.1se)	F	9	0
2-LASSO (λ.min)	F	29	20
3-LASSO (λ.min)	NF	30	24
4-LASSO (λ.1se)	NF	12	6
5-Random Forest	NF	30	25
6-Random Forest	NF	12	8

*: Likelihood ratio test p-value < 0.001 when comparing each metabolite model (Models 2- 6) to the clinical-features-only model (Model 1).

Supplemental Table S4. Metabolites (p = 49) selected across six multivariable models for eGFR slope outcome

Ion Index	Tuning parameter choice for Lasso Models				Random Forest		# of times a metabolite ion is selected across the six models	Metabolite Name
	Force		Not Force					
	λ.1se *	λ.min *	λ.min *	λ.1se *	Top 30*	Top 12*		
**Model # (from Table 2)**	**1**	**2**	**3**	**4**	**5**	**6**		
1079	0	0	0	0	1	0	1	Phenylalanine
1098	0	1	1	1	0	0	3	3-(4-Methyl-3-pentenyl)thiophene
1099	0	1	1	1	0	0	3	C10:3
1114	0	0	0	0	1	0	1	Zapotidine
1219	0	0	1	0	0	0	1	Glycerol 3-phosphate
1384	0	0	0	0	1	0	1	Hippurate
2202	0	1	1	1	0	0	3	Zalcitabine
2227	0	0	1	0	0	0	1	Glycyl-Histidine
226	0	0	1	0	0	0	1	Hydroxybutanoic acid
2399	0	0	0	0	1	0	1	4-Nitrophenyl sulfate
2480	0	0	0	0	1	0	1	3-(6-hydroxy-7-methoxy-2H-1,3-benzodioxol-5-yl)prop-2-enal
2513	0	1	1	0	0	0	2	4-(2-Amino-3-hydroxyphenyl)-2,4-dioxobutanoic acid
255	0	1	1	0	0	0	2	Aminophenol
2718	0	0	1	0	0	0	1	Asparaginyl-Valine
2755	0	0	0	0	1	1	2	2,4-Dihydroxy-acetophenone 5-sulfate
281	0	1	1	0	0	0	2	Furoic acid
30	0	1	1	0	1	1	4	Butynal
3117	0	1	1	1	0	0	3	Asparaginyl-Hydroxyproline

3165	0	1	0	0	0	0	1	2-Hydroxy-acetaminophen sulfate
3193	0	0	0	0	1	0	1	Vanillic acid 4-sulfate
3260	0	0	0	0	1	0	1	Heptabarbital
3380	0	0	1	0	0	0	1	Thiamylal
344	0	1	0	0	1	1	3	Valine; Betaine
3756	0	1	1	0	0	0	2	Adenosine
3756	0	1	1	0	0	0	2	Neuraminic acid
3996	0	0	0	0	1	0	1	[4-(3-hydroxybutyl)-2-methoxyphenyl] oxidanesulfonic acid
3996	0	0	0	0	1	0	1	O-Demethylfonsecin
4207	0	1	0	0	0	0	1	6-Thioinosinic acid
4376	0	0	0	0	1	0	1	5-(4-Acetoxy-3-oxo-1-butynyl)-2,2''-bithiophene
4442	0	0	1	0	0	0	1	Pantoyllactone glucoside
473	0	0	1	0	0	0	1	Pipecolate
4754	0	1	1	1	0	0	3	Arginyl-Glutamine
487	0	0	0	0	1	1	2	Itaconate
506	0	0	0	0	1	1	2	(Iso)Leucine
5196	0	0	0	0	1	0	1	Ubiquinone-2
526	0	0	1	0	1	0	2	Ornithine
5302	0	0	1	0	1	0	2	Zeranol
534	0	0	0	0	1	0	1	Aspartate
5388	0	1	1	0	0	0	2	Hydroxyhexamide
6333	0	0	0	0	1	0	1	[(5-oxo-1,7-diphenylheptan-2-yl)oxy]sulfonic acid
6478	0	0	0	0	1	0	1	3,4,5-trihydroxy-6-{[3-(hydroxymethyl)-1-oxo-1H-isochromen-7-yl]oxy}oxane-2-carboxylic acid

6512	0	0	0	0	1	1	2	Amisulpride
7244	0	1	1	0	0	0	2	Pipazethate
7671	0	1	1	0	0	0	2	Aminophylline
798	0	1	0	0	1	1	3	Pentose
798	0	1	0	0	1	1	3	Diisopropyl disulfide
8590	0	1	0	0	0	0	1	Dolichyl b-D-glucosyl phosphate
9178	0	1	1	1	1	1	5	3,4-Dicaffeoyl-1,5-quinolactone
9355	0	1	0	0	0	0	1	2-(2,4-dihydroxy-phenyl)-3-(3,7-dimethylocta-2,6-dien-1-yl)-5,7-dihydroxy-6-(4-hydroxy-3-methylbut-2-en-1-yl)-3,4-dihydro-2H-1-benzopyran-4-one
9355	0	1	0	0	0	0	1	Argatroban
9419	0	0	0	0	1	0	1	3,7,11,15,23-Pentaoxolanost-8-en-26-oic acid
9464	0	0	1	0	0	0	1	Capsianoside V
983	0	0	0	0	1	0	1	Oxoadipate

*0 means not selected and 1 means selected by that model. 49 Metabolites selected across six multivariable models for eGFR slope outcome.

Supplemental Table S5. Pathway Enrichment Analysis for 131 Features

auc (-log10 pval)	max -log10 pval	max -log10 qval (BH)	Pathway	# of hits	ion indices	annotation of hits
924.6201144	9.155605371	2.38192E-09	Transcription Translation	9	344 362 506 522 534 740 1079 2028	HMDB0000159 HMDB0000167 HMDB0000168 HMDB0000172 HMDB0000182 HMDB0000191 HMDB0000687 HMDB0000883
848.6396038	10.79557417	5.91859E-11	Methylmalonic Aciduria	8	215 226 344 490 506	HMDB0000008 HMDB0000023 HMDB0000172 HMDB0000491 HMDB0000687 HMDB0000695 HMDB0000883 HMDB0002166
827.0602089	10.25372887	1.90897E-10	2-Methyl-3-Hydroxybutryl CoA Dehydrogenase Deficiency	7	215 226 344 490 506	HMDB0000023 HMDB0000172 HMDB0000491 HMDB0000687 HMDB0000695 HMDB0000883 HMDB0002166
827.0602089	10.25372887	1.90897E-10	3-Hydroxy-3-Methylglutaryl-CoA Lyase Deficiency	7	215 226 344 490 506	HMDB0000023 HMDB0000172 HMDB0000491 HMDB0000687 HMDB0000695 HMDB0000883 HMDB0002166
827.0602089	10.25372887	1.90897E-10	3-Methylcrotonyl Coa Carboxylase Deficiency	7	215 226 344 490 506	HMDB0000023 HMDB0000172 HMDB0000491 HMDB0000687 HMDB0000695 HMDB0000883 HMDB0002166
827.0602089	10.25372887	1.90897E-10	3-Methylglutaconic Aciduria	7	215 226 344 490 506	HMDB0000023 HMDB0000172 HMDB0000491 HMDB0000687 HMDB0000695

						HMDB0000883 HMDB0002166
827.0602089	10.25372887	1.90897E-10	3-Methylglutaconic AciduriaII	7	215 226 344 490 506	HMDB0000023 HMDB0000172 HMDB0000491 HMDB0000687 HMDB0000695 HMDB0000883 HMDB0002166
827.0602089	10.25372887	1.90897E-10	3-Methylglutaconic AciduriaV	7	215 226 344 490 506	HMDB0000023 HMDB0000172 HMDB0000491 HMDB0000687 HMDB0000695 HMDB0000883 HMDB0002166
827.0602089	10.25372887	1.90897E-10	3-hydroxyisobutyric acid dehydrogenase deficiency	7	215 226 344 490 506	HMDB0000023 HMDB0000172 HMDB0000491 HMDB0000687 HMDB0000695 HMDB0000883 HMDB0002166
827.0602089	10.25372887	1.90897E-10	3-hydroxyisobutyric aciduria	7	215 226 344 490 506	HMDB0000023 HMDB0000172 HMDB0000491 HMDB0000687 HMDB0000695 HMDB0000883 HMDB0002166
827.0602089	10.25372887	1.90897E-10	Beta-Ketothiolase Deficiency	7	215 226 344 490 506	HMDB0000023 HMDB0000172 HMDB0000491 HMDB0000687 HMDB0000695 HMDB0000883 HMDB0002166
827.0602089	10.25372887	1.90897E-10	Isobutyryl-coa dehydrogenase deficiency	7	215 226 344 490 506	HMDB0000023 HMDB0000172 HMDB0000491 HMDB0000687 HMDB0000695 HMDB0000883 HMDB0002166

827.0602089	10.25372887	1.90897E-10	Isovaleric Aciduria	7	215 226 344 490 506	HMDB0000023 HMDB0000172 HMDB0000491 HMDB0000687 HMDB0000695 HMDB0000883 HMDB0002166
827.0602089	10.25372887	1.90897E-10	Isovaleric acidemia	7	215 226 344 490 506	HMDB0000023 HMDB0000172 HMDB0000491 HMDB0000687 HMDB0000695 HMDB0000883 HMDB0002166
827.0602089	10.25372887	1.90897E-10	Maple Syrup Urine Disease	7	215 226 344 490 506	HMDB0000023 HMDB0000172 HMDB0000491 HMDB0000687 HMDB0000695 HMDB0000883 HMDB0002166
827.0602089	10.25372887	1.90897E-10	Methylmalonate Semialdehyde Dehydrogenase Deficiency	7	215 226 344 490 506	HMDB0000023 HMDB0000172 HMDB0000491 HMDB0000687 HMDB0000695 HMDB0000883 HMDB0002166
827.0602089	10.25372887	1.90897E-10	Propionic Acidemia	7	215 226 344 490 506	HMDB0000023 HMDB0000172 HMDB0000491 HMDB0000687 HMDB0000695 HMDB0000883 HMDB0002166
827.0602089	10.25372887	1.90897E-10	Valine Leucine and Isoleucine Degradation	7	215 226 344 490 506	HMDB0000023 HMDB0000172 HMDB0000491 HMDB0000687 HMDB0000695 HMDB0000883 HMDB0002166
608.5556453	7.054608497	2.57957E-07	2-aminoadipic 2-oxoadipic aciduria	5	473 721 740 983	HMDB0000182 HMDB0000225 HMDB0000716 HMDB0001263

						HMDB0003405
608.5556453	7.054608497	2.57957E-07	Hyperlysinemia I Familial	5	473 721 740 983	HMDB0000182 HMDB0000225 HMDB0000716 HMDB0001263 HMDB0003405
608.5556453	7.054608497	2.57957E-07	Hyperlysinemia II or Saccharopinuria	5	473 721 740 983	HMDB0000182 HMDB0000225 HMDB0000716 HMDB0001263 HMDB0003405
608.5556453	7.054608497	2.57957E-07	Pyridoxine dependency with seizures	5	473 721 740 983	HMDB0000182 HMDB0000225 HMDB0000716 HMDB0001263 HMDB0003405
608.5556453	7.054608497	2.57957E-07	Saccharopinuria Hyperlysinemia II	5	473 721 740 983	HMDB0000182 HMDB0000225 HMDB0000716 HMDB0001263 HMDB0003405
606.86437	7.5150756	9.33914E-08	Amikacin Pathway	5	344 362 506 522	HMDB0000167 HMDB0000168 HMDB0000172 HMDB0000687 HMDB0000883
606.86437	7.5150756	9.33914E-08	Chloramphenicol Action Pathway	5	344 362 506 522	HMDB0000167 HMDB0000168 HMDB0000172 HMDB0000687 HMDB0000883
606.86437	7.5150756	9.33914E-08	Clindamycin Pathway	5	344 362 506 522	HMDB0000167 HMDB0000168 HMDB0000172 HMDB0000687 HMDB0000883
606.86437	7.5150756	9.33914E-08	Clomocycline Pathway	5	344 362 506 522	HMDB0000167 HMDB0000168 HMDB0000172 HMDB0000687 HMDB0000883
606.86437	7.5150756	9.33914E-08	Demeclocycline Pathway	5	344 362 506 522	HMDB0000167 HMDB0000168 HMDB0000172 HMDB0000687 HMDB0000883

606.86437	7.5150756	9.33914E-08	Doxycycline Pathway	5	344 362 506 522	HMDB0000167 HMDB0000168 HMDB0000172 HMDB0000687 HMDB0000883
606.86437	7.5150756	9.33914E-08	Erythromycin Pathway	5	344 362 506 522	HMDB0000167 HMDB0000168 HMDB0000172 HMDB0000687 HMDB0000883
606.86437	7.5150756	9.33914E-08	Gentamicin Pathway	5	344 362 506 522	HMDB0000167 HMDB0000168 HMDB0000172 HMDB0000687 HMDB0000883
606.86437	7.5150756	9.33914E-08	Josamycin Action Pathway	5	344 362 506 522	HMDB0000167 HMDB0000168 HMDB0000172 HMDB0000687 HMDB0000883
606.86437	7.5150756	9.33914E-08	Kanamycin Pathway	5	344 362 506 522	HMDB0000167 HMDB0000168 HMDB0000172 HMDB0000687 HMDB0000883
606.86437	7.5150756	9.33914E-08	Lincomycin Action Pathway	5	344 362 506 522	HMDB0000167 HMDB0000168 HMDB0000172 HMDB0000687 HMDB0000883
606.86437	7.5150756	9.33914E-08	Lymecycline Pathway	5	344 362 506 522	HMDB0000167 HMDB0000168 HMDB0000172 HMDB0000687 HMDB0000883
606.86437	7.5150756	9.33914E-08	Methacycline Action Pathway	5	344 362 506 522	HMDB0000167 HMDB0000168 HMDB0000172 HMDB0000687 HMDB0000883
606.86437	7.5150756	9.33914E-08	Minocycline Pathway	5	344 362 506 522	HMDB0000167 HMDB0000168 HMDB0000172 HMDB0000687 HMDB0000883

606.86437	7.5150756	9.33914E-08	Neomycin Pathway	5	344 362 506 522	HMDB0000167 HMDB0000168 HMDB0000172 HMDB0000687 HMDB0000883
606.86437	7.5150756	9.33914E-08	Netilmicin Pathway	5	344 362 506 522	HMDB0000167 HMDB0000168 HMDB0000172 HMDB0000687 HMDB0000883
606.86437	7.5150756	9.33914E-08	Oxytetracycline Pathway	5	344 362 506 522	HMDB0000167 HMDB0000168 HMDB0000172 HMDB0000687 HMDB0000883
606.86437	7.5150756	9.33914E-08	Paromomycin Action Pathway	5	344 362 506 522	HMDB0000167 HMDB0000168 HMDB0000172 HMDB0000687 HMDB0000883
606.86437	7.5150756	9.33914E-08	Rolitetracycline Action Pathway	5	344 362 506 522	HMDB0000167 HMDB0000168 HMDB0000172 HMDB0000687 HMDB0000883
606.86437	7.5150756	9.33914E-08	Roxithromycin Pathway	5	344 362 506 522	HMDB0000167 HMDB0000168 HMDB0000172 HMDB0000687 HMDB0000883
606.86437	7.5150756	9.33914E-08	Spectinomycin Pathway	5	344 362 506 522	HMDB0000167 HMDB0000168 HMDB0000172 HMDB0000687 HMDB0000883
606.86437	7.5150756	9.33914E-08	Streptomycin Pathway	5	344 362 506 522	HMDB0000167 HMDB0000168 HMDB0000172 HMDB0000687 HMDB0000883
606.86437	7.5150756	9.33914E-08	Tetracycline Pathway	5	344 362 506 522	HMDB0000167 HMDB0000168 HMDB0000172 HMDB0000687 HMDB0000883

606.86437	7.5150756	9.33914E-08	Tigecycline Action Pathway	5	344 362 506 522	HMDB0000167 HMDB0000168 HMDB0000172 HMDB0000687 HMDB0000883
606.86437	7.5150756	9.33914E-08	Tobramycin Action Pathway	5	344 362 506 522	HMDB0000167 HMDB0000168 HMDB0000172 HMDB0000687 HMDB0000883
577.5299119	7.256291613	1.66053E-07	Arbekacin Action Pathway	5	344 362 506 522	HMDB0000167 HMDB0000168 HMDB0000172 HMDB0000687 HMDB0000883
577.5299119	7.256291613	1.66053E-07	Azithromycin Pathway	5	344 362 506 522	HMDB0000167 HMDB0000168 HMDB0000172 HMDB0000687 HMDB0000883
577.5299119	7.256291613	1.66053E-07	Clarithromycin Pathway	5	344 362 506 522	HMDB0000167 HMDB0000168 HMDB0000172 HMDB0000687 HMDB0000883
577.5299119	7.256291613	1.66053E-07	Telithromycin Pathway	5	344 362 506 522	HMDB0000167 HMDB0000168 HMDB0000172 HMDB0000687 HMDB0000883
577.5299119	7.256291613	1.66053E-07	Troleandomycin Action Pathway	5	344 362 506 522	HMDB0000167 HMDB0000168 HMDB0000172 HMDB0000687 HMDB0000883
566.2013188	6.637861909	6.7079E-07	Lysine Degradation	5	473 721 740 983	HMDB0000182 HMDB0000225 HMDB0000716 HMDB0001263 HMDB0003405
509.8205521	7.091948012	2.41458E-07	Tryptophan Metabolism	9	592 999 1075 1294 1695 2028	HMDB0000197 HMDB0000763 HMDB0000929 HMDB0001123 HMDB0004073 HMDB0004077

					2513 3310	HMDB0004083 HMDB0004086
507.1681215	5.524222572	8.68013E-06	Glutaric Aciduria Type I	6	473 721 740 983 3435	HMDB0000182 HMDB0000225 HMDB0000716 HMDB0001263 HMDB0003405
427.3014386	4.69903907	5.62789E-05	Methylenetetrahydrofolate Reductase Deficiency	5	215 344 362 1293 3756	HMDB0000043 HMDB0000092 HMDB0000719
407.177883	4.296911545	0.000141524	Betaine Metabolism	3	215 344 3756	HMDB0000043 HMDB0000092
372.94126	4.9584832	3.10849E-05	Cystathionine Beta-Synthase Deficiency	4	215 344 362 3756	HMDB0000043 HMDB0000092 HMDB0000719
372.94126	4.9584832	3.10849E-05	Glycine N-methyltransferase Deficiency	4	215 344 362 3756	HMDB0000043 HMDB0000092 HMDB0000719
372.94126	4.9584832	3.10849E-05	Homocystinuria-megaloblastic anemia due to defect in cobalamin metabolism	4	215 344 362 3756	HMDB0000043 HMDB0000092 HMDB0000719
372.94126	4.9584832	3.10849E-05	Hypermethioninemia	4	215 344 362 3756	HMDB0000043 HMDB0000092 HMDB0000719
372.94126	4.9584832	3.10849E-05	Methionine Adenosyltransferase Deficiency	4	215 344 362 3756	HMDB0000043 HMDB0000092 HMDB0000719
372.94126	4.9584832	3.10849E-05	Methionine Metabolism	4	215 344 362 3756	HMDB0000043 HMDB0000092 HMDB0000719
372.94126	4.9584832	3.10849E-05	S-Adenosylhomocysteine Hydrolase Deficiency	4	215 344 362 3756	HMDB0000043 HMDB0000092 HMDB0000719
354.5800761	4.093274253	0.000224493	Glycine and Serine Metabolism	5	215 344 362 526 2115	HMDB0000043 HMDB0000092 HMDB0000167

						HMDB0000214
348.1722007	3.716543427	0.000528545	Canavan Disease	4	522 534 1293	HMDB0000168 HMDB0000191 HMDB0000812 HMDB0006483
348.1722007	3.716543427	0.000528545	Hypoacetylaspartia	4	522 534 1293	HMDB0000168 HMDB0000191 HMDB0000812 HMDB0006483
331.5949736	3.554061318	0.000765523	Aspartate Metabolism	4	522 534 1293	HMDB0000168 HMDB0000191 HMDB0006483
308.9620164	4.169405331	0.000189104	The oncogenic action of 2-hydroxyglutarate	4	548 766	HMDB0000156 HMDB0000606 HMDB0000694 HMDB0059655
295.2549989	3.997605969	0.000278771	Sarcosine oncometabolite pathway	2	215 344	HMDB0000043 HMDB0000092
293.5754475	3.539817141	0.000782386	Biotin Metabolism	2	740	HMDB0000182 HMDB0003405
293.5754475	3.539817141	0.000782386	Biotinidase Deficiency	2	740	HMDB0000182 HMDB0003405
293.5754475	3.539817141	0.000782386	Multiple carboxylase deficiency	2	740	HMDB0000182 HMDB0003405
276.7865681	3.37440371	0.001140913	Malate-Aspartate Shuttle	2	534 548	HMDB0000191 HMDB0000744
238.2026646	3.369659926	0.001149264	Glutaminolysis and Cancer	3	506 534 548	HMDB0000156 HMDB0000191 HMDB0000687
223.4364272	2.431895244	0.009380499	Ammonia Recycling	3	522 534 2115	HMDB0000168 HMDB0000191 HMDB0012210
219.3383298	2.834073507	0.003888278	The oncogenic action of Fumarate	3	548 766	HMDB0000156 HMDB0000744 HMDB0059655
204.426349	3.228079384	0.001575089	Malonic Aciduria	2	226 344	HMDB0000008 HMDB0000883
204.426349	3.228079384	0.001575089	Malonyl-coa decarboxylase deficiency	2	226 344	HMDB0000008 HMDB0000883

204.426349	3.228079384	0.001575089	Propanoate Metabolism	2	226 344	HMDB0000008 HMDB0000883
181.2841414	2.279090369	0.0131124	Mycophenolic Acid Metabolism Pathway	2	506 721	HMDB0060650 HMDB0061156
179.2126099	2.480279127	0.008420286	Argininemia	2	526 534	HMDB0000191 HMDB0000214
179.2126099	2.480279127	0.008420286	Argininosuccinic Aciduria	2	526 534	HMDB0000191 HMDB0000214
179.2126099	2.480279127	0.008420286	Carbamoyl Phosphate Synthetase Deficiency	2	526 534	HMDB0000191 HMDB0000214
179.2126099	2.480279127	0.008420286	Citrullinemia Type I	2	526 534	HMDB0000191 HMDB0000214
179.2126099	2.480279127	0.008420286	Ornithine Transcarbamylase Deficiency	2	526 534	HMDB0000191 HMDB0000214
179.2126099	2.480279127	0.008420286	Urea Cycle	2	526 534	HMDB0000191 HMDB0000214
179.2033006	2.393233535	0.010218989	Carnitine Synthesis	2	740	HMDB0000182 HMDB0003405
173.9923595	2.524617635	0.007762563	3-Phosphoglycerate dehydrogenase deficiency	3	215 344 2115	HMDB0000043 HMDB0000092
173.9923595	2.524617635	0.007762563	Dihydropyrimidine Dehydrogenase Deficiency	3	215 344 2115	HMDB0000043 HMDB0000092
173.9923595	2.524617635	0.007762563	Dimethylglycine Dehydrogenase Deficiency	3	215 344 2115	HMDB0000043 HMDB0000092
173.9923595	2.524617635	0.007762563	Hyperglycinemia non-ketotic	3	215 344 2115	HMDB0000043 HMDB0000092
173.9923595	2.524617635	0.007762563	Non Ketotic Hyperglycinemia	3	215 344 2115	HMDB0000043 HMDB0000092
173.9923595	2.524617635	0.007762563	Sarcosinemia	3	215 344 2115	HMDB0000043 HMDB0000092

171.3442082	1.991355132	0.023230122	Aminocaproic Acid Pathway	1	506	HMDB0001901
171.3442082	1.991355132	0.023230122	Leucine Stimulation on Insulin Signaling	1	506	HMDB0000687
158.3765722	1.790533482	0.034986209	Beta Ureidopropionase Deficiency	3	215 522 3079	HMDB0000026 HMDB0003911
158.3765722	1.790533482	0.034986209	Dihydropyrimidinase Deficiency	3	215 522 3079	HMDB0000026 HMDB0003911
158.3765722	1.790533482	0.034986209	Mitochondrial Neurogastrointestinal Encephalopathy	3	215 522 3079	HMDB0000026 HMDB0003911
158.3765722	1.790533482	0.034986209	Pyrimidine Metabolism	3	215 522 3079	HMDB0000026 HMDB0003911
158.3765722	1.790533482	0.034986209	UMP Synthase Deficiency Orotic Aciduria	3	215 522 3079	HMDB0000026 HMDB0003911
154.7183257	2.369121032	0.010729416	Mercaptopurine Pathway	5	534 561 3756 4207	HMDB0000034 HMDB0000050 HMDB0000085 HMDB0000191 HMDB0060791
154.7183257	2.369121032	0.010729416	Thioguanine Pathway	5	534 561 3756 4207	HMDB0000034 HMDB0000050 HMDB0000085 HMDB0000191 HMDB0060791
153.4040565	2.09334326	0.019025453	AICA-Ribosiduria	4	534 561 3756	HMDB0000034 HMDB0000050 HMDB0000085 HMDB0000191
153.4040565	2.09334326	0.019025453	Adenine phosphoribosyltransferase deficiency	4	534 561 3756	HMDB0000034 HMDB0000050 HMDB0000085 HMDB0000191

153.40405<br>65	2.0933432<br>6	0.0190254<br>53	Adenosine Deaminase Deficiency	4	534 561<br>3756	HMDB0000034<br>HMDB0000050<br>HMDB0000085<br>HMDB0000191
153.40405<br>65	2.0933432<br>6	0.0190254<br>53	Adenylosucci nate Lyase Deficiency	4	534 561<br>3756	HMDB0000034<br>HMDB0000050<br>HMDB0000085<br>HMDB0000191
153.40405<br>65	2.0933432<br>6	0.0190254<br>53	Gout or Kelley-Seegmiller Syndrome	4	534 561<br>3756	HMDB0000034<br>HMDB0000050<br>HMDB0000085<br>HMDB0000191
153.40405<br>65	2.0933432<br>6	0.0190254<br>53	Lesch-Nyhan Syndrome	4	534 561<br>3756	HMDB0000034<br>HMDB0000050<br>HMDB0000085<br>HMDB0000191
153.40405<br>65	2.0933432<br>6	0.0190254<br>53	Mitochondria l DNA depletion syndrome	4	534 561<br>3756	HMDB0000034<br>HMDB0000050<br>HMDB0000085<br>HMDB0000191
153.40405<br>65	2.0933432<br>6	0.0190254<br>53	Molybdenum Cofactor Deficiency	4	534 561<br>3756	HMDB0000034<br>HMDB0000050<br>HMDB0000085<br>HMDB0000191
153.40405<br>65	2.0933432<br>6	0.0190254<br>53	Myoadenylat e deaminase deficiency	4	534 561<br>3756	HMDB0000034<br>HMDB0000050<br>HMDB0000085<br>HMDB0000191
153.40405<br>65	2.0933432<br>6	0.0190254<br>53	Purine Nucleoside Phosphorylas e Deficiency	4	534 561<br>3756	HMDB0000034<br>HMDB0000050<br>HMDB0000085<br>HMDB0000191
153.40405<br>65	2.0933432<br>6	0.0190254<br>53	Xanthine Dehydrogena se Deficiency	4	534 561<br>3756	HMDB0000034<br>HMDB0000050<br>HMDB0000085<br>HMDB0000191
153.40405<br>65	2.0933432<br>6	0.0190254<br>53	Xanthinuria	4	534 561<br>3756	HMDB0000034<br>HMDB0000050<br>HMDB0000085<br>HMDB0000191
153.40405<br>65	2.0933432<br>6	0.0190254<br>53	XanthinuriaI	4	534 561<br>3756	HMDB0000034<br>HMDB0000050<br>HMDB0000085<br>HMDB0000191
151.44606<br>67	2.0330477<br>83	0.0217898<br>35	Threonine and 2-	1	362	HMDB0000167

			Oxobutanoate Degradation			
150.3024006	2.296714118	0.012633342	Azathioprine Pathway	5	534 561 3756 4207	HMDB0000034 HMDB0000050 HMDB0000085 HMDB0000191 HMDB0060791
150.1629127	1.997491916	0.023136325	2-Hydroxyglutric Aciduria	2	215 534	HMDB0000112 HMDB0000191
150.1629127	1.997491916	0.023136325	4-Hydroxybutyric Aciduria Succinic Semialdehyde Dehydrogenase Deficiency	2	215 534	HMDB0000112 HMDB0000191
150.1629127	1.997491916	0.023136325	Glutamate Metabolism	2	215 534	HMDB0000112 HMDB0000191
150.1629127	1.997491916	0.023136325	Homocarnosinosis	2	215 534	HMDB0000112 HMDB0000191
150.1629127	1.997491916	0.023136325	Hyperinsulinism-Hyperammonemia Syndrome	2	215 534	HMDB0000112 HMDB0000191
150.1629127	1.997491916	0.023136325	Succinic semialdehyde dehydrogenase deficiency	2	215 534	HMDB0000112 HMDB0000191
148.3102905	2.02046685	0.022359532	Purine Metabolism	4	534 561 3756	HMDB0000034 HMDB0000050 HMDB0000085 HMDB0000191
145.3246516	2.127288723	0.018352412	Beta-Alanine Metabolism	2	522 534	HMDB0000026 HMDB0000191
145.3246516	2.127288723	0.018352412	Carnosinuria or carnosinemia	2	522 534	HMDB0000026 HMDB0000191
145.3246516	2.127288723	0.018352412	GABA-Transaminase Deficiency	2	522 534	HMDB0000026 HMDB0000191
145.3246516	2.127288723	0.018352412	Ureidopropionase Deficiency	2	522 534	HMDB0000026 HMDB0000191

129.2333779	1.732037525	0.039914638	The oncogenic action of Succinate	2	548 766	HMDB0000156 HMDB0059655
127.6080507	1.990389575	0.023230122	Ketone Body Metabolism	1	226	HMDB0000011
127.6080507	1.990389575	0.023230122	Succinyl CoA 3-ketoacid CoA transferase deficiency	1	226	HMDB0000011
125.6471327	1.692905677	0.043551893	Fatty Acid Biosynthesis	3	226 490 3435	HMDB0000357 HMDB0010717
116.5644777	1.49510747	0.067505426	Spermidine and Spermine Biosynthesis	1	526	HMDB0000214
114.1847236	1.791248985	0.034986209	Arginine Glycine Amidinotransferase Deficiency	2	526 534	HMDB0000191 HMDB0000214
114.1847236	1.791248985	0.034986209	Arginine and Proline Metabolism	2	526 534	HMDB0000191 HMDB0000214
114.1847236	1.791248985	0.034986209	Creatine deficiency or guanidinoacetate methyltransferase deficiency	2	526 534	HMDB0000191 HMDB0000214
114.1847236	1.791248985	0.034986209	Guanidinoacetate Methyltransferase Deficiency	2	526 534	HMDB0000191 HMDB0000214
114.1847236	1.791248985	0.034986209	Hyperornithinemia with gyrate atrophy	2	526 534	HMDB0000191 HMDB0000214
114.1847236	1.791248985	0.034986209	Hyperornithinemia-hyperammonemia-homocitrullinuria	2	526 534	HMDB0000191 HMDB0000214

114.18472 36	1.7912489 85	0.0349862 09	Hyperproline mia	2	526 534	HMDB0000191 HMDB0000214
114.18472 36	1.7912489 85	0.0349862 09	Hyperproline miaI	2	526 534	HMDB0000191 HMDB0000214
114.18472 36	1.7912489 85	0.0349862 09	L-arginine glycine amidinotransf erase deficiency	2	526 534	HMDB0000191 HMDB0000214
114.18472 36	1.7912489 85	0.0349862 09	Ornithine Aminotransfe rase Deficiency	2	526 534	HMDB0000191 HMDB0000214
114.18472 36	1.7912489 85	0.0349862 09	Prolidase Deficiency	2	526 534	HMDB0000191 HMDB0000214
114.18472 36	1.7912489 85	0.0349862 09	Prolinemia Type II	2	526 534	HMDB0000191 HMDB0000214
104.16567 05	1.3748839 65	0.0885322 67	D-Arginine and D-Ornithine Metabolism	1	526	HMDB0003374
99.317126 24	1.3160677 56	0.1005200 84	Benzocaine Pathway	1	1079	HMDB0004992
98.700605 25	1.5210369 51	0.0639703 33	Nicotinate and Nicotinamide Metabolism	1	410	HMDB0001488
92.807141 9	1.4663732 61	0.0719185 37	Zalcitabine Action Pathway	1	2202	HMDB0015078
92.425781 99	1.2432116 93	0.1175627 99	Transfer of Acetyl Groups into Mitochondria	1	548	HMDB0000156
89.098197 85	1.2461749 88	0.1170877 13	Folate Metabolism	1	1293	HMDB0003470
89.098197 85	1.2461749 88	0.1170877 13	Folate malabsorptio n hereditary	1	1293	HMDB0003470
89.098197 85	1.2461749 88	0.1170877 13	Methotrexate Pathway	1	1293	HMDB0003470
88.212135 75	1.5197030 89	0.0639703 33	D-glyceric acidura	2	1219 3435	HMDB0000126 HMDB0000220
88.212135 75	1.5197030 89	0.0639703 33	Familial lipoprotein	2	1219 3435	HMDB0000126 HMDB0000220

			lipase deficiency			
88.21213575	1.519703089	0.063970333	Glycerol Kinase Deficiency	2	1219 3435	HMDB0000126 HMDB0000220
88.21213575	1.519703089	0.063970333	Glycerolipid Metabolism	2	1219 3435	HMDB0000126 HMDB0000220
77.28960609	1.089900705	0.147334432	Glycerol Phosphate Shuttle	1	1219	HMDB0000126
77.28960609	1.089900705	0.147334432	Mitochondrial Electron Transport Chain	1	1219	HMDB0000126
73.38018341	0.917925298	0.206811072	Tyrosinemia	2	534 1079	HMDB0000159 HMDB0000191
71.29861049	1.008722183	0.172157625	2-ketoglutarate dehydrogenase complex deficiency	1	548	HMDB0000156
71.29861049	1.008722183	0.172157625	Citric Acid Cycle	1	548	HMDB0000156
71.29861049	1.008722183	0.172157625	Congenital lactic acidosis	1	548	HMDB0000156
71.29861049	1.008722183	0.172157625	Fructose-16-diphosphatase deficiency	1	548	HMDB0000744
71.29861049	1.008722183	0.172157625	Fumarase deficiency	1	548	HMDB0000156
71.29861049	1.008722183	0.172157625	Gluconeogenesis	1	548	HMDB0000744
71.29861049	1.008722183	0.172157625	Glycogen Storage Disease Type 1A or Von Gierke Disease	1	548	HMDB0000744
71.29861049	1.008722183	0.172157625	Mitochondrial complex II deficiency	1	548	HMDB0000156
71.29861049	1.008722183	0.172157625	Phosphoenolpyruvate carboxykinase deficiency	1	548	HMDB0000744

71.29861049	1.008722183	0.172157625	Pyruvate dehydrogenase deficiency	1	548	HMDB0000156
71.29861049	1.008722183	0.172157625	Triosephosphate isomerase	1	548	HMDB0000744
68.47735206	0.950670487	0.193126138	Phenylalanine and Tyrosine Metabolism	1	1079	HMDB0000159
68.47735206	0.950670487	0.193126138	Phenylketonuria	1	1079	HMDB0000159
68.0491199	0.968161737	0.187240245	Leigh Syndrome	1	548	HMDB0000156
68.0491199	0.968161737	0.187240245	Pyruvate Decarboxylase E1 Component Deficiency	1	548	HMDB0000156
68.0491199	0.968161737	0.187240245	Pyruvate Dehydrogenase Complex Deficiency	1	548	HMDB0000156
68.0491199	0.968161737	0.187240245	Pyruvate kinase deficiency	1	548	HMDB0000156
65.29086232	0.931966615	0.20115853	Pyruvate Metabolism	1	548	HMDB0000156
63.6736347	0.951274007	0.193126138	Hypophosphatasia	1	1486	HMDB0000017
63.6736347	0.951274007	0.193126138	Vitamin B6 Metabolism	1	1486	HMDB0000017
62.55990175	0.842495833	0.242133228	Glucose-6-phosphate dehydrogenase deficiency	1	798	HMDB0000283
62.55990175	0.842495833	0.242133228	Pentose Phosphate Pathway	1	798	HMDB0000283
62.55990175	0.842495833	0.242133228	Ribose-5-phosphate isomerase deficiency	1	798	HMDB0000283
62.55990175	0.842495833	0.242133228	Transaldolase deficiency	1	798	HMDB0000283
62.04903687	0.893284628	0.218381039	Phospholipid Biosynthesis	1	1219	HMDB0000126

60.92632858	0.869812628	0.229979802	Primary Hyperoxaluria	1	548	HMDB0000156
59.87523419	0.844020111	0.242133228	Valproic Acid Metabolism Pathway	1	645	HMDB0060682
56.47902602	0.795234908	0.268751056	Glycogenosis	1	548	HMDB0000744
55.93997338	1.36770446	0.089754452	Zidovudine Action Pathway	1	3756	HMDB0014638
55.41659506	0.774106644	0.281513483	Warburg Effect	1	548	HMDB0000156
52.46320188	0.643177297	0.365737542	Disulfiram Pathway	1	534	HMDB0000191
52.41711061	0.654667239	0.356960901	Alkaptonuria	1	534	HMDB0000191
52.41711061	0.654667239	0.356960901	Dopamine beta-hydroxylase deficiency	1	534	HMDB0000191
52.41711061	0.654667239	0.356960901	Hawkinsinuria	1	534	HMDB0000191
52.41711061	0.654667239	0.356960901	Monoamine oxidase-a deficiency	1	534	HMDB0000191
52.41711061	0.654667239	0.356960901	Tyrosine Metabolism	1	534	HMDB0000191
48.57732351	0.74536795	0.293297454	Felbamate Metabolism Pathway	1	1695	HMDB0060400
46.48386401	1.332094498	0.097150338	Plasmalogen Synthesis	1	4220	HMDB0000827
37.20167053	0.92856997	0.202269755	Selenoamino Acid Metabolism	1	3756	HMDB0000050
36.36606167	1.051722873	0.160091666	Mitochondrial Beta-Oxidation of Long Chain Saturated Fatty Acids	1	4220	HMDB0000827
33.60310407	0.827878779	0.249854839	Long-chain-3-hydroxyacyl-	1	3435	HMDB0000220

			coa dehydrogenase deficiency			
30.31782951	0.749697321	0.291872576	Carnitine palmitoyl transferase deficiency	1	3435	HMDB0000220
30.31782951	0.749697321	0.291872576	Ethylmalonic Encephalopathy	1	3435	HMDB0000220
30.31782951	0.749697321	0.291872576	Fatty Acid Elongation In Mitochondria	1	3435	HMDB0000220
30.31782951	0.749697321	0.291872576	Fatty Acid Metabolism	1	3435	HMDB0000220
30.31782951	0.749697321	0.291872576	Long chain acyl-CoA dehydrogenase deficiency	1	3435	HMDB0000220
30.31782951	0.749697321	0.291872576	Medium chain acyl-coa dehydrogenase deficiency	1	3435	HMDB0000220
30.31782951	0.749697321	0.291872576	Short Chain Acyl CoA Dehydrogenase Deficiency	1	3435	HMDB0000220
30.31782951	0.749697321	0.291872576	Trifunctional protein deficiency	1	3435	HMDB0000220
30.31782951	0.749697321	0.291872576	Very-long-chain acyl coa dehydrogenase deficiency	1	3435	HMDB0000220
29.56813219	0.744715678	0.293297454	Insulin Signalling	1	3756	HMDB0000050
22.78978613	0.746960113	0.293071001	Hydroxylase deficiency	3	3435 8568 9483	HMDB0000036 HMDB0000138 HMDB0000220
22.54872191	1.162389104	0.129093544	Acetylsalicylic Acid Pathway	4	6153	HMDB0001139 HMDB0004684 HMDB0004694 HMDB0011137

22.548721 91	1.1623891 04	0.1290935 44	Antipyrine Action Pathway	4	6153	HMDB0001139 HMDB0004684 HMDB0004694 HMDB0011137
22.548721 91	1.1623891 04	0.1290935 44	Antrafenine Action Pathway	4	6153	HMDB0001139 HMDB0004684 HMDB0004694 HMDB0011137
22.548721 91	1.1623891 04	0.1290935 44	Bromfenac Pathway	4	6153	HMDB0001139 HMDB0004684 HMDB0004694 HMDB0011137
22.548721 91	1.1623891 04	0.1290935 44	Carprofen Action Pathway	4	6153	HMDB0001139 HMDB0004684 HMDB0004694 HMDB0011137
22.548721 91	1.1623891 04	0.1290935 44	Celecoxib Pathway	4	6153	HMDB0001139 HMDB0004684 HMDB0004694 HMDB0011137
22.548721 91	1.1623891 04	0.1290935 44	Diclofenac Pathway	4	6153	HMDB0001139 HMDB0004684 HMDB0004694 HMDB0011137
22.548721 91	1.1623891 04	0.1290935 44	Diflunisal Pathway	4	6153	HMDB0001139 HMDB0004684 HMDB0004694 HMDB0011137
22.548721 91	1.1623891 04	0.1290935 44	Etoricoxib Action Pathway	4	6153	HMDB0001139 HMDB0004684 HMDB0004694 HMDB0011137
22.548721 91	1.1623891 04	0.1290935 44	Fenoprofen Action Pathway	4	6153	HMDB0001139 HMDB0004684 HMDB0004694 HMDB0011137
22.548721 91	1.1623891 04	0.1290935 44	Flurbiprofen Action Pathway	4	6153	HMDB0001139 HMDB0004684 HMDB0004694 HMDB0011137
22.548721 91	1.1623891 04	0.1290935 44	Indomethacin Pathway	4	6153	HMDB0001139 HMDB0004684 HMDB0004694 HMDB0011137
22.548721 91	1.1623891 04	0.1290935 44	Ketoprofen Pathway	4	6153	HMDB0001139 HMDB0004684

						HMDB0004694<br>HMDB0011137
22.548721<br>91	1.1623891<br>04	0.1290935<br>44	Ketorolac<br>Pathway	4	6153	HMDB0001139<br>HMDB0004684<br>HMDB0004694<br>HMDB0011137
22.548721<br>91	1.1623891<br>04	0.1290935<br>44	Leukotriene<br>C4 Synthesis<br>Deficiency	4	6153	HMDB0001139<br>HMDB0004684<br>HMDB0004694<br>HMDB0011137
22.548721<br>91	1.1623891<br>04	0.1290935<br>44	Lornoxicam<br>Action<br>Pathway	4	6153	HMDB0001139<br>HMDB0004684<br>HMDB0004694<br>HMDB0011137
22.548721<br>91	1.1623891<br>04	0.1290935<br>44	Lumiracoxib<br>Action<br>Pathway	4	6153	HMDB0001139<br>HMDB0004684<br>HMDB0004694<br>HMDB0011137
22.548721<br>91	1.1623891<br>04	0.1290935<br>44	Magnesium<br>salicylate<br>Action<br>Pathway	4	6153	HMDB0001139<br>HMDB0004684<br>HMDB0004694<br>HMDB0011137
22.548721<br>91	1.1623891<br>04	0.1290935<br>44	Mefanamic<br>Acid Pathway	4	6153	HMDB0001139<br>HMDB0004684<br>HMDB0004694<br>HMDB0011137
22.548721<br>91	1.1623891<br>04	0.1290935<br>44	Nabumetone<br>Pathway	4	6153	HMDB0001139<br>HMDB0004684<br>HMDB0004694<br>HMDB0011137
22.548721<br>91	1.1623891<br>04	0.1290935<br>44	Nepafenac<br>Action<br>Pathway	4	6153	HMDB0001139<br>HMDB0004684<br>HMDB0004694<br>HMDB0011137
22.548721<br>91	1.1623891<br>04	0.1290935<br>44	Oxaprozin<br>Pathway	4	6153	HMDB0001139<br>HMDB0004684<br>HMDB0004694<br>HMDB0011137
22.548721<br>91	1.1623891<br>04	0.1290935<br>44	Phenylbutazo<br>ne Action<br>Pathway	4	6153	HMDB0001139<br>HMDB0004684<br>HMDB0004694<br>HMDB0011137
22.548721<br>91	1.1623891<br>04	0.1290935<br>44	Piroxicam<br>Pathway	4	6153	HMDB0001139<br>HMDB0004684<br>HMDB0004694<br>HMDB0011137

22.548721 91	1.1623891 04	0.1290935 44	Rofecoxib Pathway	4	6153	HMDB0001139 HMDB0004684 HMDB0004694 HMDB0011137
22.548721 91	1.1623891 04	0.1290935 44	Salicylate-sodium Action Pathway	4	6153	HMDB0001139 HMDB0004684 HMDB0004694 HMDB0011137
22.548721 91	1.1623891 04	0.1290935 44	Salsalate Action Pathway	4	6153	HMDB0001139 HMDB0004684 HMDB0004694 HMDB0011137
22.548721 91	1.1623891 04	0.1290935 44	Sulindac Pathway	4	6153	HMDB0001139 HMDB0004684 HMDB0004694 HMDB0011137
22.548721 91	1.1623891 04	0.1290935 44	Suprofen Pathway	4	6153	HMDB0001139 HMDB0004684 HMDB0004694 HMDB0011137
22.548721 91	1.1623891 04	0.1290935 44	Tenoxicam Action Pathway	4	6153	HMDB0001139 HMDB0004684 HMDB0004694 HMDB0011137
22.548721 91	1.1623891 04	0.1290935 44	Tiaprofenic Acid Action Pathway	4	6153	HMDB0001139 HMDB0004684 HMDB0004694 HMDB0011137
22.548721 91	1.1623891 04	0.1290935 44	Tolmetin Action Pathway	4	6153	HMDB0001139 HMDB0004684 HMDB0004694 HMDB0011137
22.548721 91	1.1623891 04	0.1290935 44	Trisalicylate-choline Action Pathway	4	6153	HMDB0001139 HMDB0004684 HMDB0004694 HMDB0011137
22.548721 91	1.1623891 04	0.1290935 44	Valdecoxib Pathway	4	6153	HMDB0001139 HMDB0004684 HMDB0004694 HMDB0011137
22.111937 07	1.1063745 41	0.1428959 31	Cerebrotendinous Xanthomatosis	3	3435 8568 9483	HMDB0000036 HMDB0000138 HMDB0000220

22.11193707	1.106374541	0.142895931	Congenital Bile Acid Synthesis Defect Type	3	3435 8568 9483	HMDB0000036 HMDB0000138 HMDB0000220
22.11193707	1.106374541	0.142895931	Congenital Bile Acid Synthesis Defect TypeI	3	3435 8568 9483	HMDB0000036 HMDB0000138 HMDB0000220
22.11193707	1.106374541	0.142895931	Familial Hypercholanemia	3	3435 8568 9483	HMDB0000036 HMDB0000138 HMDB0000220
22.11193707	1.106374541	0.142895931	Zellweger Syndrome	3	3435 8568 9483	HMDB0000036 HMDB0000138 HMDB0000220
21.95777522	1.131937086	0.13640355	Acetaminophen Action Pathway	4	6153	HMDB0001139 HMDB0004684 HMDB0004694 HMDB0011137
21.95777522	1.131937086	0.13640355	Arachidonic Acid Metabolism	4	6153	HMDB0001139 HMDB0004684 HMDB0004694 HMDB0011137
21.95777522	1.131937086	0.13640355	Etodolac Pathway	4	6153	HMDB0001139 HMDB0004684 HMDB0004694 HMDB0011137
21.95777522	1.131937086	0.13640355	Ibuprofen Pathway	4	6153	HMDB0001139 HMDB0004684 HMDB0004694 HMDB0011137
21.95777522	1.131937086	0.13640355	Meloxicam Pathway	4	6153	HMDB0001139 HMDB0004684 HMDB0004694 HMDB0011137
21.95777522	1.131937086	0.13640355	Salicylic Acid Action Pathway	4	6153	HMDB0001139 HMDB0004684 HMDB0004694 HMDB0011137
21.88727716	1.062680283	0.156482836	Bile Acid Biosynthesis	3	3435 8568 9483	HMDB0000036 HMDB0000138 HMDB0000220
21.40073399	1.103138821	0.143611702	Naproxen Pathway	4	6153	HMDB0001139 HMDB0004684 HMDB0004694 HMDB0011137

6.726414512	1.229675391	0.120949716	Argatroban Pathway	1	9355	HMDB0014423
0	0	1	11-beta-hydroxylase deficiency	0		
0	0	1	17-Beta Hydroxysteroid Dehydrogenase III Deficiency	0		
0	0	1	17-alpha-hydroxylase deficiency	0		
0	0	1	3-Beta-Hydroxysteroid Dehydrogenase Deficiency	0		
0	0	1	3-Methylthiofentanyl Action Pathway	0		
0	0	1	5-Oxoprolinuria	0		
0	0	1	5-oxoprolinase deficiency	0		
0	0	1	Acebutolol Pathway	0		
0	0	1	Acetaminophen Metabolism Pathway	0		
0	0	1	Acute Intermittent Porphyria	0		
0	0	1	Adrenal Hyperplasia or Congenital Adrenal Hyperplasia	0		
0	0	1	Adrenoleukodystrophy X-linked	0		

0	0	1	Alanine Metabolism	0		
0	0	1	Alendronate pathway	0		
0	0	1	Alfentanil Pathway	0		
0	0	1	Alimemazine H1-Antihistamine Action	0		
0	0	1	Alpha Linolenic Acid and Linoleic Acid Metabolism	0		
0	0	1	Alprenolol Pathway	0		
0	0	1	Alvimopan Action Pathway	0		
0	0	1	Amiloride Pathway	0		
0	0	1	Amino Sugar Metabolism	0		
0	0	1	Androgen and Estrogen Metabolism	0		
0	0	1	Androstenedione Metabolism	0		
0	0	1	Anileridine Action Pathway	0		
0	0	1	Apparent mineralocorticoid excess syndrome	0		
0	0	1	Aromatase deficiency	0		
0	0	1	Aromatic L-Aminoacid Decarboxylase Deficiency	0		
0	0	1	Artemether Metabolism Pathway	0		

0	0	1	Atenolol Pathway	0		
0	0	1	Atorvastatin Pathway	0		
0	0	1	Bendroflumethiazide Pathway	0		
0	0	1	Beta Oxidation of Very Long Chain Fatty Acids	0		
0	0	1	Beta-mercaptolactate-cysteine disulfiduria	0		
0	0	1	Betahistine H1-Antihistamine Action	0		
0	0	1	Betazole Action Pathway	0		
0	0	1	Bisoprolol Pathway	0		
0	0	1	Blue diaper syndrome	0		
0	0	1	Bumetanide Pathway	0		
0	0	1	Bupivacaine Pathway	0		
0	0	1	Buprenorphine Action Pathway	0		
0	0	1	Butyrate Metabolism	0		
0	0	1	CHILD Syndrome	0		
0	0	1	Caffeine Metabolism	0		
0	0	1	Captopril Pathway	0		
0	0	1	Carbamazepine Metabolism Pathway	0		

0	0	1	Cardiolipin Biosynthesis	0		
0	0	1	Carfentanil Pathway	0		
0	0	1	Carnitine-acylcarnitine translocase deficiency	0		
0	0	1	Catecholamine Biosynthesis	0		
0	0	1	Cerivastatin Pathway	0		
0	0	1	Chloroprocaine Pathway	0		
0	0	1	Chlorothiazide Pathway	0		
0	0	1	Chlorthalidone Pathway	0		
0	0	1	Cholesteryl ester storage disease	0		
0	0	1	Chondrodysplasia Punctata II X Linked Dominant	0		
0	0	1	Cimetidine Pathway	0		
0	0	1	Citalopram Pathway	0		
0	0	1	Cocaine Pathway	0		
0	0	1	Codeine Pathway	0		
0	0	1	Congenital Erythropoietic Porphyria or Gunther Disease	0		
0	0	1	Congenital Lipoid Adrenal Hyperplasia or Lipoid CAH	0		

0	0	1	Congenital disorder of glycosylation CDG-IId	0		
0	0	1	Corticosterone methyl oxidase deficiency	0		
0	0	1	Corticotropin Activation of Cortisol Production	0		
0	0	1	Cyclophosphamide Pathway	0		
0	0	1	Cyclothiazide Pathway	0		
0	0	1	Cysteine Metabolism	0		
0	0	1	Cystinosis ocular nonnephropathic	0		
0	0	1	Cystinuria	0		
0	0	1	De Novo Triacylglycerol Biosynthesis	0		
0	0	1	Desipramine Pathway	0		
0	0	1	Desmosterolosis	0		
0	0	1	Dezocine Action Pathway	0		
0	0	1	Dibucaine Pathway	0		
0	0	1	Dihydromorphine Action Pathway	0		
0	0	1	Dimethylthiambutene Action Pathway	0		

0	0	1	Diphenoxylate Action Pathway	0		
0	0	1	Dopa-responsive dystonia	0		
0	0	1	Dopamine Activation of Neurological Reward System	0		
0	0	1	Doxepin H1-Antihistamine Action	0		
0	0	1	Doxepin Metabolism Pathway	0		
0	0	1	Emedastine H1-Antihistamine Action	0		
0	0	1	Enalapril Metabolism Pathway	0		
0	0	1	Enalapril Pathway	0		
0	0	1	Eplerenone Pathway	0		
0	0	1	Escitalopram Pathway	0		
0	0	1	Estrone Metabolism	0		
0	0	1	Ethacrynic Acid pathway	0		
0	0	1	Ethanol Degradation	0		
0	0	1	Ethylmorphine Action Pathway	0		
0	0	1	Excitatory Neural Signalling Through 5-HTR and Serotonin	0		
0	0	1	Fabry disease	0		

0	0	1	Fanconi-bickel syndrome	0		
0	0	1	Fc Epsilon Receptor I Signaling in Mast Cells	0		
0	0	1	Fentanyl Pathway	0		
0	0	1	Fexofenadine H1-Antihistamine Action	0		
0	0	1	Fluoxetine Pathway	0		
0	0	1	Fluvastatin Pathway	0		
0	0	1	Fosphenytoin Antiarrhythmic Pathway	0		
0	0	1	Fructose and Mannose Degradation	0		
0	0	1	Fructose intolerance hereditary	0		
0	0	1	Fructosuria	0		
0	0	1	Furosemide Pathway	0		
0	0	1	GLUT-1 deficiency syndrome	0		
0	0	1	GM2-Gangliosidosis Variant B Tay-sachs disease	0		
0	0	1	Galactose Metabolism	0		
0	0	1	Galactosemia	0		
0	0	1	Gamma-Glutamyltransferase Deficiency	0		

0	0	1	Gamma-cystathionase deficiency	0		
0	0	1	Gamma-glutamyl-transpeptidase deficiency	0		
0	0	1	Gaucher Disease	0		
0	0	1	Glibenclamide Pathway	0		
0	0	1	Gliclazide Pathway	0		
0	0	1	Globoid Cell Leukodystrophy	0		
0	0	1	Glucose Transporter Defect	0		
0	0	1	Glucose-Alanine Cycle	0		
0	0	1	Glutathione Metabolism	0		
0	0	1	Glutathione Synthetase Deficiency	0		
0	0	1	Glycogen synthetase deficiency	0		
0	0	1	Glycolysis	0		
0	0	1	Hartnup Disorder	0		
0	0	1	Hereditary Coproporphyria	0		
0	0	1	Heroin Pathway	0		
0	0	1	Histidine Metabolism	0		
0	0	1	Histidinemia	0		
0	0	1	Homocysteine Degradation	0		
0	0	1	Homocystinuria cystathionine	0		

			beta-synthase deficiency			
0	0	1	Hydrochlorothiazide Pathway	0		
0	0	1	Hydrocodone Pathway	0		
0	0	1	Hydroflumethiazide Pathway	0		
0	0	1	Hydromorphone Pathway	0		
0	0	1	Hyper-IgD syndrome	0		
0	0	1	Hypercholesterolemia	0		
0	0	1	Hyperphenylalaniemia due to guanosine triphosphate cyclohydrolase deficiency	0		
0	0	1	Hyperphenylalaninemia due to 6-pyruvoyltetrahydropterin synthase deficiency	0		
0	0	1	Hyperphenylalaninemia due to dhpr-deficiency	0		
0	0	1	Ibandronate Pathway	0		
0	0	1	Ifosfamide Pathway	0		
0	0	1	Iminoglycinuria	0		
0	0	1	Imipramine Pathway	0		
0	0	1	Indapamide Pathway	0		
0	0	1	Inositol Metabolism	0		

0	0	1	Inositol Phosphate Metabolism	0		
0	0	1	Irbesartan Action Pathway	0		
0	0	1	Isoprenaline Action Pathway	0		
0	0	1	Joubert syndrome	0		
0	0	1	Ketobemidone Action Pathway	0		
0	0	1	Kidney Function	0		
0	0	1	Krabbe disease	0		
0	0	1	Labetalol Pathway	0		
0	0	1	Lactic Acidemia	0		
0	0	1	Lactose Degradation	0		
0	0	1	Lactose Intolerance	0		
0	0	1	Lactose Synthesis	0		
0	0	1	Lamivudine Action Pathway	0		
0	0	1	Lamivudine Metabolism Pathway	0		
0	0	1	Levallorphan Action Pathway	0		
0	0	1	Levobunolol Action Pathway	0		
0	0	1	Levobupivacaine Pathway	0		
0	0	1	Levomethadyl Acetate Action Pathway	0		

0	0	1	Levorphanol Action Pathway	0		
0	0	1	Lidocaine Pathway	0		
0	0	1	Lisinopril Pathway	0		
0	0	1	Lovastatin Pathway	0		
0	0	1	Lysinuric Protein Intolerance	0		
0	0	1	Lysosomal Acid Lipase Deficiency	0		
0	0	1	Mepivacaine Pathway	0		
0	0	1	Metachromatic Leukodystrophy	0		
0	0	1	Methadone Pathway	0		
0	0	1	Methadyl Acetate Action Pathway	0		
0	0	1	Methyclothiazide Pathway	0		
0	0	1	Methylhistidine Metabolism	0		
0	0	1	Metolazone Pathway	0		
0	0	1	Mevalonic aciduria	0		
0	0	1	Mitochondrial Beta-Oxidation of Medium Chain Saturated Fatty Acids	0		
0	0	1	Mitochondrial Beta-Oxidation of	0		

			Short Chain Saturated Fatty Acids			
0	0	1	Morphine Pathway	0		
0	0	1	Mucopolysaccharidosis Sly syndrome	0		
0	0	1	Nalbuphine Action Pathway	0		
0	0	1	Naloxone Action Pathway	0		
0	0	1	Naltrexone Action Pathway	0		
0	0	1	Nateglinide Pathway	0		
0	0	1	Nevirapine Action Pathway	0		
0	0	1	Nevirapine Metabolism Pathway	0		
0	0	1	Nicotine Pathway	0		
0	0	1	Nifedipine Pathway	0		
0	0	1	Nucleotide Sugars Metabolism	0		
0	0	1	Omeprazole Metabolism Pathway	0		
0	0	1	Omeprazole Pathway	0		
0	0	1	Orphenadrine H1-Antihistamine Action	0		
0	0	1	Oxidation of Branched Chain Fatty Acids	0		

0	0	1	Oxybuprocaine Pathway	0		
0	0	1	Oxycodone Pathway	0		
0	0	1	Oxymorphone Pathway	0		
0	0	1	PE Test	0		
0	0	1	Pamidronate Pathway	0		
0	0	1	Pancreas Function	0		
0	0	1	Pantothenate and CoA Biosynthesis	0		
0	0	1	Pentazocine Action Pathway	0		
0	0	1	Perindopril Pathway	0		
0	0	1	Phenindamine H1-Antihistamine Action	0		
0	0	1	Phenylacetate Metabolism	0		
0	0	1	Phenytoin Antiarrhythmic Pathway	0		
0	0	1	Phosphatidylcholine Biosynthesis	0		
0	0	1	Phosphatidylethanolamine Biosynthesis	0		
0	0	1	Phosphatidylinositol Phosphate Metabolism	0		
0	0	1	Phytanic Acid Peroxisomal Oxidation	0		
0	0	1	Pindolol Pathway	0		
0	0	1	Polythiazide Pathway	0		

0	0	1	Porphyria Variegata	0		
0	0	1	Porphyrin Metabolism	0		
0	0	1	Practolol Action Pathway	0		
0	0	1	Pravastatin Pathway	0		
0	0	1	Prilocaine Pathway	0		
0	0	1	Procaine Pathway	0		
0	0	1	Promethazine H1-Antihistamine Action	0		
0	0	1	Proparacaine Pathway	0		
0	0	1	Propoxyphene Action Pathway	0		
0	0	1	Propranolol Pathway	0		
0	0	1	Pterine Biosynthesis	0		
0	0	1	Pyruvaldehyde Degradation	0		
0	0	1	Pyruvate Carboxylase Deficiency	0		
0	0	1	Quetiapine H1-Antihistamine Action	0		
0	0	1	Quinapril Metabolism Pathway	0		
0	0	1	Quinapril Pathway	0		
0	0	1	Quinethazone Pathway	0		
0	0	1	Quinidine Pathway	0		
0	0	1	Ranitidine Pathway	0		

0	0	1	Refsum Disease	0		
0	0	1	Remifentanil Pathway	0		
0	0	1	Repaglinide Pathway	0		
0	0	1	Retinol Metabolism	0		
0	0	1	Riboflavin Metabolism	0		
0	0	1	Risedronate Pathway	0		
0	0	1	Ropivacaine Pathway	0		
0	0	1	Rosiglitazone Metabolism Pathway	0		
0	0	1	Rosuvastatin Pathway	0		
0	0	1	Roxatidine acetate Action Pathway	0		
0	0	1	Salla Disease Infantile Sialic Acid Storage Disease	0		
0	0	1	Segawa syndrome	0		
0	0	1	Sepiapterin reductase deficiency	0		
0	0	1	Short-chain 3-hydroxyacyl-CoA dehydrogenase deficiency	0		
0	0	1	Sialuria or French Type Sialuria	0		
0	0	1	Simvastatin Action Pathway	0		

0	0	1	Smith-Lemli-Opitz Syndrome	0		
0	0	1	Sotalol Action Pathway	0		
0	0	1	Sphingolipid Metabolism	0		
0	0	1	Spironolactone Pathway	0		
0	0	1	Starch and Sucrose Metabolism	0		
0	0	1	Stavudine Action Pathway	0		
0	0	1	Steroid Biosynthesis	0		
0	0	1	Steroidogenesis	0		
0	0	1	Sucrase-isomaltase deficiency	0		
0	0	1	Sufentanil Pathway	0		
0	0	1	Sulfate Sulfite Metabolism	0		
0	0	1	Sulfite oxidase deficiency	0		
0	0	1	Tamoxifen Pathway	0		
0	0	1	Taurine and Hypotaurine Metabolism	0		
0	0	1	Tay-Sachs Disease	0		
0	0	1	Thiamine Metabolism	0		
0	0	1	Thioguanine Metabolism Pathway	0		
0	0	1	Thyroid hormone synthesis	0		

0	0	1	Torsemide Pathway	0		
0	0	1	Tramadol Action Pathway	0		
0	0	1	Tramadol Metabolism Pathway	0		
0	0	1	Tranexamic Acid Pathway	0		
0	0	1	Trehalose Degradation	0		
0	0	1	Triamterene Pathway	0		
0	0	1	Trichlormethiazide Pathway	0		
0	0	1	Triprolidine H1-Antihistamine Action	0		
0	0	1	Tyrosine hydroxylase deficiency	0		
0	0	1	Ubiquinone Biosynthesis	0		
0	0	1	Valsartan Action Pathway	0		
0	0	1	Vasopressin Regulation of Water Homeostasis	0		
0	0	1	Venlafaxine Metabolism Pathway	0		
0	0	1	Verapamil Pathway	0		
0	0	1	Vitamin A Deficiency	0		
0	0	1	Vitamin K Metabolism	0		
0	0	1	Warfarin Pathway	0		
0	0	1	Wolman disease	0		

0	0	1	Zoledronate Pathway	0		

Supplemental Figure S1. Boxplot of cross-validated concordance (C-statistics) for time-to-ESKD for each model in Supplemental Table S3

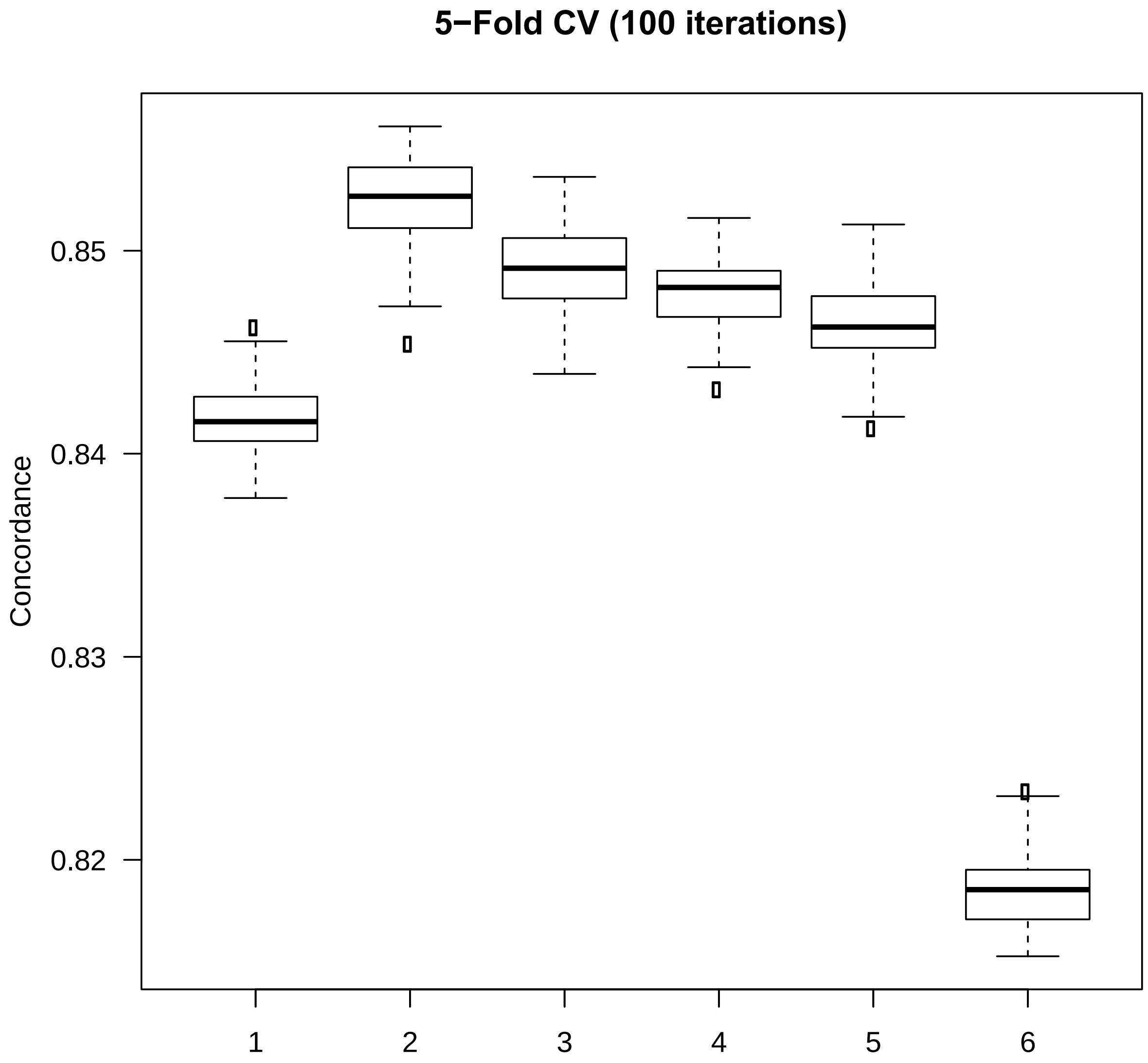

The numbers on X axis denote the fitted model as specified in Supplemental Table S3. Model 1 is the clinical- variable only model.

Note: R^2 of 6 survival models are 0.46, 0.50, 0.50, 0.47, 0.50, 0.41, respectively.

Supplemental Figure S2. Pathway enrichment analysis using metabolite ions selected in adjusted single metabolite and/or multiple metabolite models

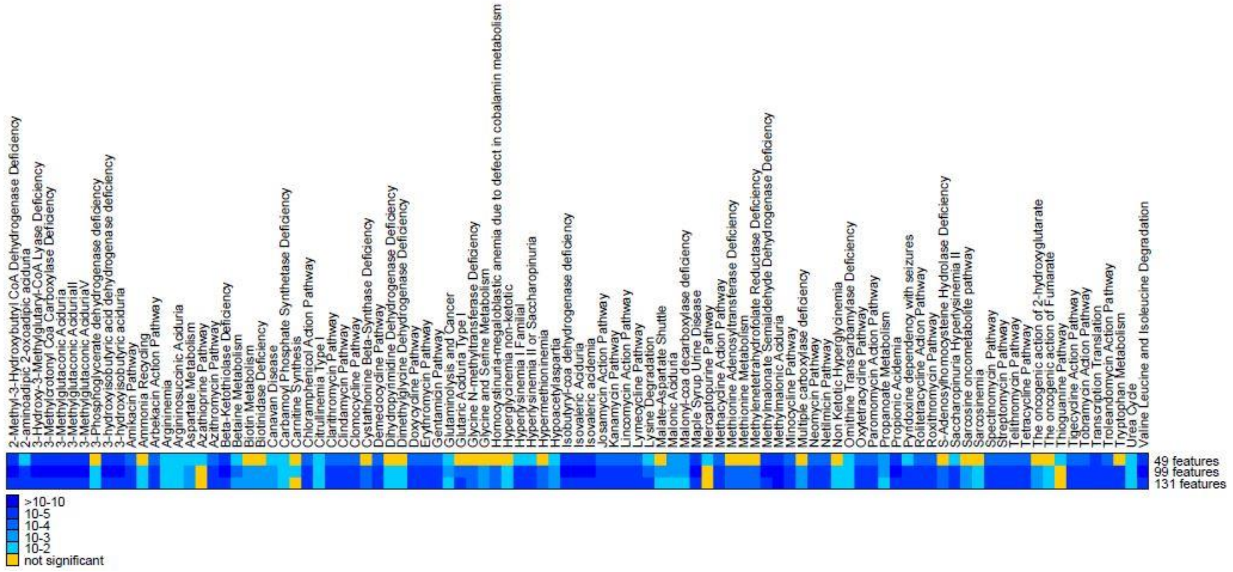

Top row: 49 metabolite ions selected in multivariable models for eGFR slope outcome (Supplemental Table S4);

Middle row: 99 metabolite ions significant in adjusted Cox model, testing associations between single metabolite ion and time-to-ESKD, adjusted for 9 clinical variables (Supplemental Table S2);

Bottom row: 131 metabolites merged from the 49-metabolite-ion and 99-metabolite-ion sets.

Supplementary References

Fuhrer, T, Heer D, Begemann B, et al. High-throughput, accurate mass metabolome profiling of cellular extracts by flow injection-time-of-flight mass spectrometry. Anal Chem, 2011. **83**(18): p. 7074-80.
